# Pyridinamide Ion Pairs: Design Principles for Super-Nucleophiles in Apolar Organic Solvents

**DOI:** 10.1021/acs.joc.4c02668

**Published:** 2025-01-31

**Authors:** Veronika Burger, Maximilian Franta, AnnMarie C. O‘Donoghue, Armin R. Ofial, Ruth M. Gschwind, Hendrik Zipse

**Affiliations:** † Department of Chemistry, Ludwig-Maximilians Universität München, Butenandtstr. 5-13, 81377 München, Germany; ‡ Institute for Organic Chemistry, University Regensburg, Universitätsstr. 31, 93053 Regensburg, Germany; § Department of Chemistry, Durham University, South Road, Durham DH1 3LE, United Kingdom

## Abstract

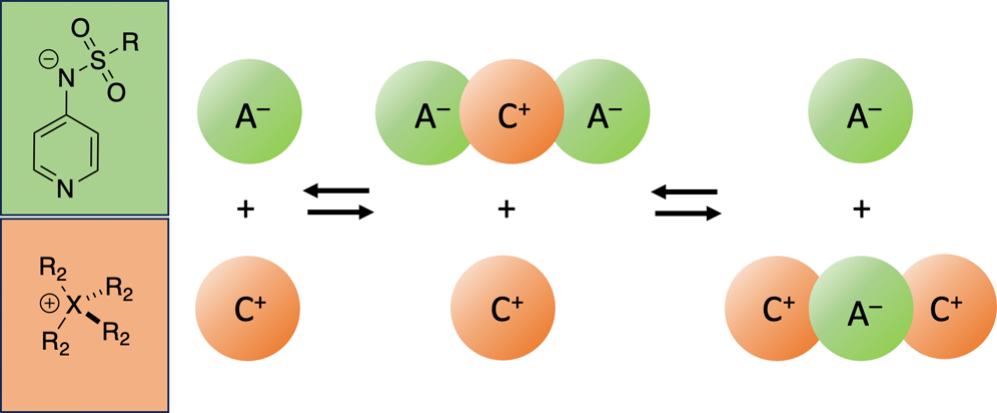

A comprehensive analytical protocol combining conductivity, diffusion-ordered NMR (DOSY), and photometric kinetic measurements is employed to analyze the nucleophilic reactivity of pyridinamide ion pairs in low-polarity organic solvents. The association patterns of these systems are found to strongly depend on cation size, with larger cations favoring the formation of cationic triple ion sandwich complexes together with free and highly nucleophilic anions. Kinetic studies using the ionic strength-controlled benzhydrylium method demonstrate that pyridinamide ions exhibit significantly higher nucleophilicities as compared to established organocatalysts, particularly in low-polarity solvents. Nucleophilicities are furthermore found to correlate well with Brønsted basicities measured in water and with Lewis basicities calculated in dichloromethane. Taken together, these findings provide quantitative guidelines for the future design of highly active Lewis base catalysts.

## Introduction

**Chart 1:**
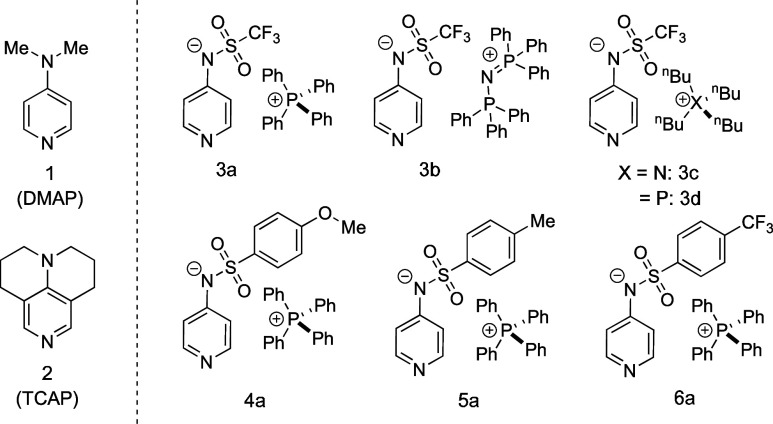
Structures of Neutral Organocatalysts DMAP (1), TCAP (2), and the Pyridinamide Ion Pair Library

In the field of organic synthesis, ion pair catalysis has emerged as a powerful tool that leverages the interaction between charged species to enhance catalytic activity and selectivity to facilitate various chemical transformations.^1−4^ Its application extends from classical phase-transfer (PT) catalysis^5^ to asymmetric synthesis.^6−9^ Recent studies have shown that ion pair catalysis offers unique opportunities for selectivity control and overcomes challenges where conventional methods may fall short.^10,11^ Pyridinamide ion pairs have recently been introduced as a new class of Lewis base catalysts capable of outperforming established structurally related neutral organocatalysts such as 4-(dimethylamino)pyridine (DMAP, **1**)^12,13^ and the more reactive 9-azajulolidine (TCAP, **2**)^14^ in selected benchmark reactions.^15,16^ In contrast to neutral pyridine-based catalysts **1** and **2**, the actual state of the ion pair catalysts shown in Chart 1 depends on a variety of factors, such as the solvent polarity, the choice of additives, and the concentration regime used in catalytic processes. For pyridinamide phosphonium salts, such as **3a** and **4a** (Chart 1), we have recently outlined a protocol for quantifying their concentration-dependent speciation and the nucleophilicity of the free anion component toward reference electrophiles of known reactivity in organic solvents of low polarity such as dichloromethane (DCM).^17^ Most kinetic studies aiming to quantify the reactivity of anionic nucleophiles rely on highly polar solvents such as water, DMSO, and acetonitrile (MeCN), often in combination with additives such as crown ethers to reduce interactions between the cationic counterion and the reacting anion.^18^ The intrinsic nucleophilicity of a free anion is, however, expected to be higher in organic solvents of low polarity (DCM, THF, and toluene) commonly used in organocatalytic transformations. Ion pairs tend to associate in apolar media in a concentration-dependent manner, causing nonlinear effects and thus complicating systematic kinetic studies.

**Figure 1:**
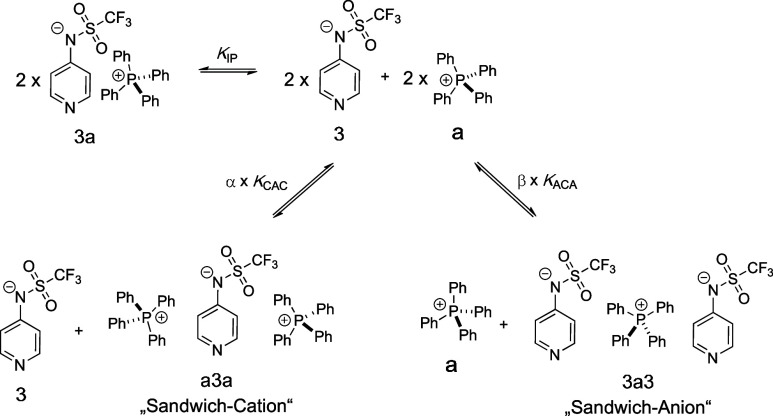
Association types of anion **3** and cation **a** leading to either the 1:1 ion pair **3a**, the sandwich cation **a3a**, or the sandwich anion **3a3** with their respective association constants *K*_IP_, *K*_CAC_, and *K*_ACA_.

Our recently introduced analytical protocol combines conductivity measurements, diffusion-ordered NMR spectroscopy (DOSY) measurements, and photometric kinetic measurements utilizing an ionic strength-controlled benzhydrylium ion methodology and enables us to uniquely link insights into the concentration of ions with their association state and nucleophilicity. For **3a** as a reference system, the results from conductivity and DOSY experiments can best be rationalized by assuming the concentration-dependent formation of the “sandwich”-type cations (**a3a**) and anions (**3a3**) shown in Figure 1, with little interference from the respective 1:1 ion pair (**3a**). Key properties obtained from DOSY experiments in DCM are the concentration-dependent “effective” cation and anion volumes, whose combination with conductivity data then yields the equilibrium constants for all three aggregates **3a**, **a3a**, and **3a3** shown in Figure 1.

While it is, in principle, possible to fit concentration-dependent conductivity and DOSY profiles to only one of the equilibria shown in Figure 1, the best possible fit is obtained when there is simultaneous formation of both sandwich ion species **a3a** and **3a3**. In practical terms, this requires optimization of the scaling factors α (contribution of sandwich cation **a3a**) and β (contribution of sandwich anion **3a3**) shown in Figure 1. In the following, we will refer to this model as the “mixed sandwich ion model” (see the Supporting Information). The accurate description of concentration profiles for all species was then combined with the ionic strength-controlled benzhydrylium method to determine the nucleophilicity of free pyridinamide anions in DCM and MeCN. Based on the results obtained for pyridinamide ion pairs **3a**–**d** and **4a–6a** (Chart 1), we aim to extract key design principles for the synthesis of highly reactive pyridinamide ion pair systems.

## Results and Discussion

### Conductivity

Conductivity measurements were performed in DCM for salt concentrations ranging from 0.02 to 1.0 mM. At low electrolyte concentrations and for the case of noninteracting ions, the experimentally determined conductivity κ depends on the specific molar conductivity Λ_m_ and the ion concentration [IP] as expressed in eq 1.
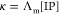
1

**Figure 2:**
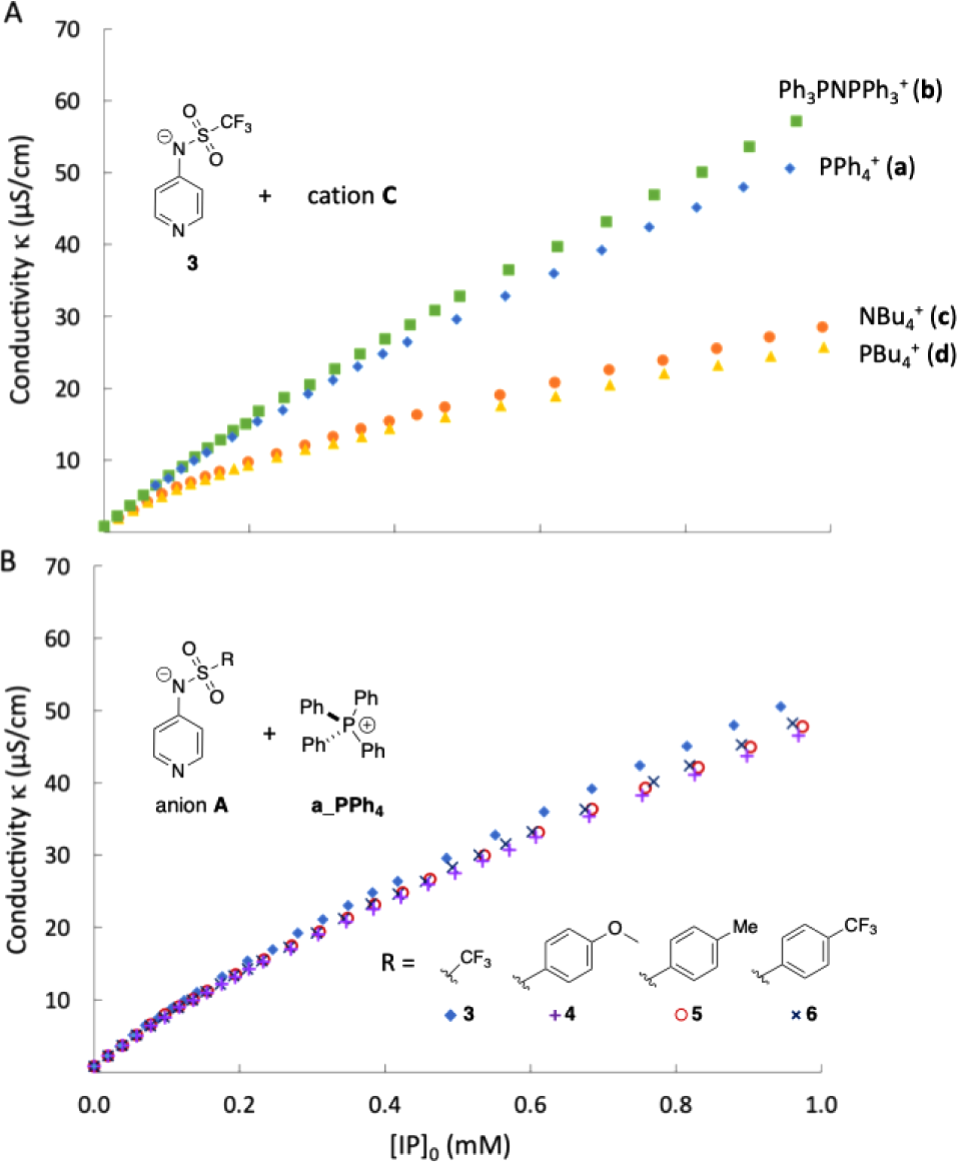
(A) Conductivity profiles for **3a**, **3b**, **3c**, and **3d** measured in DCM at 20 °C, (B) conductivity profiles for **3a**, **4a**, **5a**, and **6a** measured in DCM at 20 °C.

The conductivity curves for ion pairs composed of anion **3** and cations **a–d** shown in Figure 2A appear to fall into two separate groups. The first of these includes cations **a** (PPh_4_^+^) and **b** (PPh_3_NPPh_3_^+^) carrying aromatic substituents and displays systematically higher conductivities compared to the second group with cations **c** (NBu_4_^+^) and **d** (PBu_4_^+^) carrying unbranched aliphatic side chains. For simplicity, we focus our discussion on the cationic sandwich association constants *K*_CAC_. Pyridinamide ion pair **3b** shows the weakest degree of association of all ion pairs in DCM with *K*_CAC_(**3b**) = 4.65 × 10^6^ M^–2^, closely followed by **3a** with *K*_CAC_(**3a**) = 6.38 × 10^6^ M^–2^. In contrast, ion pairs **3c** and **3d** show systematically higher values with *K*_CAC_(**3c**) = 1.01 × 10^7^ M^–2^ and *K*_CAC_(**3d**) = 1.07 × 10^7^ M^–2^, respectively, indicating a higher degree of aggregation in DCM.

Variation of the anion substitution pattern leads, in contrast, to only minor variations in the association behavior, as is easily seen from the similar conductivity curves displayed in Figure 2B. In quantitative terms, this is reflected in the respective association constants of *K*_CAC_(**4a**) = 6.50 × 10^6^ M^–2^, *K*_CAC_(**5a**) = 5.15 × 10^6^ M^–2^, and *K*_CAC_(**6a**) = 6.75 × 10^6^ M^–2^, all of which are in close proximity to one another and quite similar to that of **3a**. The conductivity measurements thus clearly document that the lowest degree of aggregation (and thus the highest concentration of free ions) will be obtained with the two phosphonium ions **a** and **b** carrying aromatic substituents.

### DOSY NMR

Since conductivity data alone cannot determine which sandwich association type predominates for a given pyridinamide ion pair, DOSY NMR measurements were performed in DCM-d_2_ at concentrations ranging from 0.005 to 1.0 mM (see Supporting Information, Chapter S4). DOSY measurements at these low concentrations require a 600 MHz spectrometer with a helium cryo probe and measurement times of up to 16 h per sample. The DOSY results were compared to calculated volumes of ions and associated complexes of the respective pyridinamide ion pair which are based on the van der Waals cavities employed in the SMD continuum solvation model at the SMD(DCM)/B3LYP-D3/6–31+G(d) level of theory. Recent DOSY measurements of pyridinamide ion pair **3a** and **4a** revealed a prevailing cationic sandwich association for **3a** and a mainly anionic sandwich association for **4a**.^17^ The cationic sandwich association is indicated by a higher increase in the hydrodynamic cation volume in comparison to the hydrodynamic anion volume, while the anionic sandwich association displays a distinct crossing point of both ionic volumes when the anion volume surpasses the cation volume of the respective salt in the DOSY measurement.

The concentration-dependent DOSY plots of ion pair **3b**, **3c**, and **3d** display a similar curve with cation volumes larger than expected as shown for **3a**. Here, the effective cation volume increases more strongly with increasing concentration than the anion volume, which is most easily rationalized with predominant formation of cationic sandwich complexes of “**a3a**”-type (see the Supporting Information for details). In contrast, for ion pairs **4a**, **5a**, and **6a**, a crossing point of anion and cation volumes is observed where the anion volume exceeds the cation volume, which is typical for the anionic sandwich complexes of “4a4”-type.

Combined analysis of the conductivity and DOSY NMR data points to a predominance of cation sandwich formation for **3a**–**d** and a predominance of anion sandwich formation for pyridinamide ion pairs **4–6a**. This is quantitatively reflected in the scaling factors α and β collected in Table 1. For ion pair **3b** and **3d,** all experimental observables can be rationalized with excellent accuracy with the cation sandwich model alone; that is, α/β = 100/0.

**Table 1:** List of Pyridinamide Ion Pairs with Their Optimized Scale Factors α and β and Association Constants *K*_CAC_ and *K*_ACA_ in the Mixed Sandwich Ion Model

IP	α/β	α × *K*_CAC_ [M^–2^]	β × *K*_ACA_ [M^–2^]	RMSE
**3a**	44/21	2.81 × 10^6^	1.34 × 10^6^	0.43
**3b**	100/0	4.65 × 10^6^	0.00	0.11
**3c**	33/23	3.33 × 10^6^	2.32 × 10^6^	0.61
**3d**	100/0	1.07 × 10^7^	0.00	0.10
**4a**	12/61	7.80 × 10^5^	3.97 × 10^6^	0.35
**5a**	11/67	5.67 × 10^5^	3.45 × 10^6^	0.29
**6a**	16/52	1.08 × 10^6^	3.51 × 10^6^	0.38

In summary, the conductivity data demonstrate that the association pattern is more influenced by the choice of cation than anion, while the DOSY data reveal a switch from cation sandwich to anion sandwich association when moving from small anion **3** (215 Å^3^) to larger anions such as **4** (285 Å^3^), **5** (266 Å^3^), or **6** (295 Å^3^) when using **a** (362 Å^3^) as the cation component. The size difference between the chosen anion and cation components, as listed in Table 2 thus appears to represent a controlling factor for the speciation of ion pairs in organic solvents of low polarity. For the first two entries **3a** and **3b** combining aryl phosphonium cations **a** and **b** with the comparatively small anion **3,** we find a predominance of cationic sandwich association. For the combinations of larger anions **4** −**6** with phosphonium cation **a**, the volume difference is significantly smaller (<100 Å^3^) and all experiments point to a mixture of cation and anion sandwich association.

**Table 2:** Analysis of Volume Difference between Anions and Cations in Pyridinamide Ion Pairs with Aryl Substituted Cations **a** and **b**

IP	Cation volume (Å^3^)	Anion volume (Å^3^)	Volume difference^a^
**3a**	362	215	147
**3b**	559	215	344
**4a**	362	285	77
**5a**	362	266	96
**6a**	362	295	67

a Volume difference = cation volume – anion volume.

While the difference between anion and cation volume may not be the only factor that determines the association pattern of pyridinamide ion pairs in organic solvents of low polarity, it is a feature that can be adjusted in a straightforward manner to shift the association equilibria toward the formation of cationic sandwich aggregates (accompanied by an increase in the free anion concentration in solution).

### Kinetics

**Chart 2:**
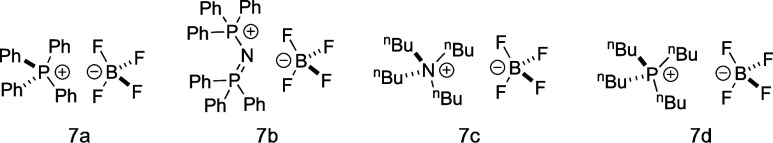
Structures of Non-nucleophilic Additives **7a**–**d** Employed in Ionic Strength-Controlled Benzhydrylium Measurements

To characterize the nucleophilic reactivity of pyridinamide ion pairs, we used the established Mayr benzhydrylium ion method suitable for the quantification of the reactivity of carbon-, nitrogen-, oxygen-, sulfur-, and phosphorus-based nucleophiles in different solvents,^19,20^ including DMAP (**1**) and TCAP (**2**).^21−23^ In order to separate the effects of increasing nucleophile concentration from those of increasing solution ionic strength, we employed the ionic strength-controlled methodology described earlier by adding the non-nucleophilic salts **7a**–**d** composed of  and the respective cation components (see Chart 2) to the reaction mixture to keep the ionic strength (*I*) constant at *I* = 1.0 mM as the upper concentration limit chosen in conductivity, DOSY, and kinetics measurements.

The benzhydrylium ion method involves the photometric monitoring of the reactions of colored benzhydrylium salts, such as **8a**–**8c** (Table 3) whose electrophilic reactivities are characterized by the solvent-independent parameters *E*, with nucleophiles used in excess concentration to achieve kinetics under pseudo-first-order conditions. The first-order rate constants *k*_obs_ (s^–1^) can then be obtained by fitting a monoexponential decay function to the decreasing absorption of **8** during the reaction with the nucleophile which is assumed to be the anion of the respective ion pair. *Kinetics in MeCN*. Conductivity measurements showed that pyridinamide salts fully dissociate into anions and cations when dissolved in MeCN. Accordingly, a linear increase of pseudo-first-order rate constants *k*_obs_ with nucleophile concentrations [**A**] (= anion concentration or total salt concentration) was observed in the kinetics of reactions of pyridinamide ion pairs with **8** (eq 2).
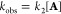
2
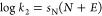
3

**Table 3:**
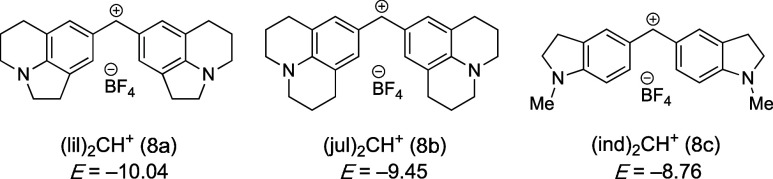
Second-Order Rate Constants *k*_2_ for the Reactions of DMAP (1), TCAP (2), and Pyridinamide Salts **3a–d**, **4a**, **5a**, and **6a** with Reference Electrophiles **8a**, **8b**, and **8c** in MeCN (at 20 °C) Analyzed by Eq 3 to Give the Nucleophile-Specific Reactivity Parameters *N* and *s*_N_

	*k***_2_** (M^–1^ s^–1^)			
IP	8a	8b	8c	*N*(*s*_N_)
**1**^a^	2.11 × 10^3^	5.30 × 10^3^	1.29 × 10^4^	15.51 (0.62)^b^
**2**^c^	6.30 × 10^3^	–	4.17 × 10^4^	15.60 (0.68)^b^
**3a**^d^	7.16 × 10^3^	1.53 × 10^4^	4.13 × 10^4^	16.38 (0.60)
**3b**	8.37 × 10^3^	1.37 × 10^4^	4.74 × 10^4^	16.68 (0.59)
**3c**	8.45 × 10^3^	1.37 × 10^4^	4.79 × 10^4^	16.68 (0.59)
**3d**	8.79 × 10^3^	1.63 × 10^4^	5.03 × 10^4^	16.48 (0.60)
**4a**^d^	5.11 × 10^4^	1.36 × 10^5^	3.47 × 10^5^	17.28 (0.65)
**5a**	3.45 × 10^4^	9.36 × 10^4^	2.30 × 10^5^	17.19 (0.64)
**6a**	2.92 × 10^4^	5.95 × 10^4^	1.16 × 10^5^	19.51 (0.47)

a Second-order rate constants *k*_2_ from Refs. (21, 22)

b Additional *k*_2_ values from Refs.^21−23^ were used to determine *N* (and *s*_N_).

c Second-order rate constants *k*_2_ from Ref (23).

d Second-order rate constants *k*_2_ from Ref (17).

Equation 2 thus yields the second-order rate constants *k*_2_ (M^–1^ s^–1^) for the reactions of pyridinamide ion pair **3a**–**d**, **4–6a** with **8a–8c** in acetonitrile (Table 3). All salts containing anion **3** display largely similar bimolecular rate constants *k*_2_, the spread of individual values amounting to ±10%. From all pyridinamide anions studied here, anion **3** is the least reactive species, but still reacts about three times faster with electrophiles **8** than DMAP (**1**) and similarly fast as TCAP (**2**). The highest rate constants *k*_2_ were measured for anion **4** with an electron-donating aryl substituent (4-MeO). These *k*_2_ values are approximately eight times larger than those for analogous reactions of **8** with TCAP (**2**) and closely followed by the rate constants *k*_2_ for anion **5** and anion **6**. The nucleophilicity parameters for pyridinamide ion pairs in MeCN were obtained by analyzing the kinetic data according to Mayr–Patz eq 3. They range between *N* = 16.38 (*s*_N_ = 0.60) for anion **3** to *N* = 17.28 (*s*_N_ = 0.65) for anion **4** with the only outlier being *N* = 19.51 for anion **6** due to its relatively low nucleophile-specific sensitivity parameter *s*_N_ = 0.46. This makes direct comparison of *N* values less convenient than the inspection of the second-order rate constants, which amount to *k*_2_ = 2.92 × 10^4^ M^–1^ s^–1^ for anion **6** and *k*_2_ = 3.45 × 10^4^ M^–1^ s^–1^ for anion **5** in reactions with **8a**.

**Figure 3:**
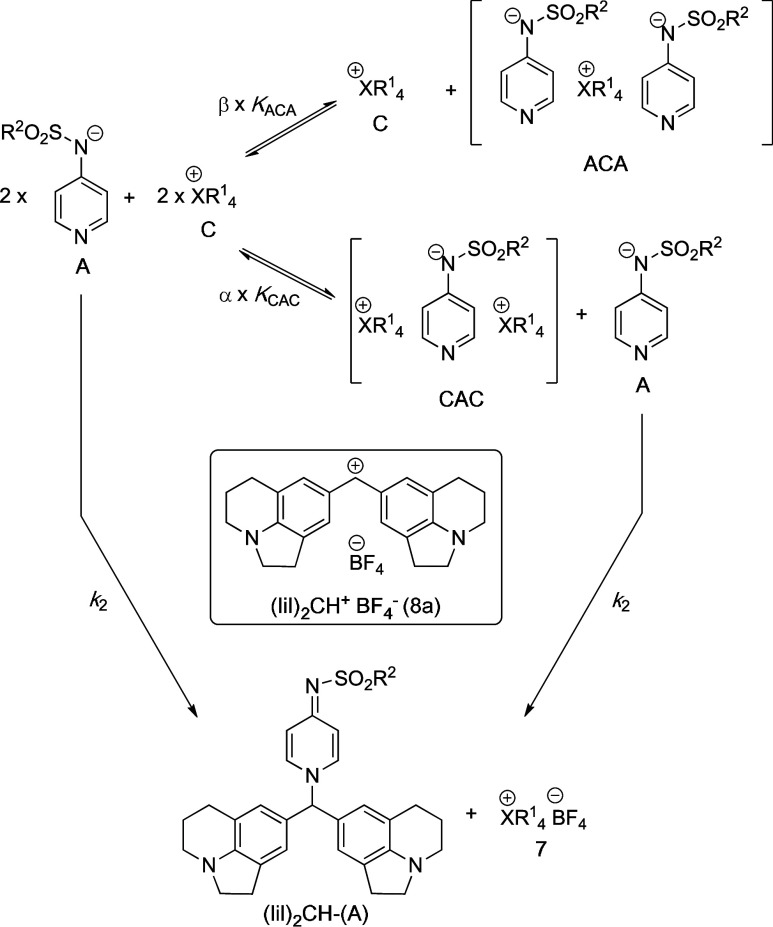
Benzhydrylium ion reaction employed for the quantification of nucleophilicities of anion **A**.

*Kinetics in DCM*. Reactions of pyridinamide ion pairs **3a**–**d**, **4–6a** with electrophiles **8** in DCM are more complex and require a slightly different approach to determine reliable reactivity parameters (for details, see Ref. (17)). We recently developed the ionic strength-controlled method where a non-nucleophilic and structurally related additive salt **7** is added to fulfill the condition of [**IP**]_0_ + [**7**] + [**8**] = 1.0 mM in each kinetic measurement. This yields a linear correlation of *k*_obs_ for **IP** + **8** reactions in DCM total ion pair concentrations ranging from 0.01 to 1.0 mM. Numerical analysis of all kinetic data accounts for the fact that variable fractions of the nucleophilic pyridinamide anions **3**–**6** are caught in unreactive cationic sandwich complexes CAC and/or in less reactive anionic sandwich complexes ACA (see Figure 3). The rate constants *k*_*2*_ collected in Table 4 for the reaction of pyridinamide ion pairs **3a**–**d**, **4–6a** with benzhydrylium ions **8a–8c** thus reflect the reaction rates of the free anion component at *I* = 1.0 mM. In DCM as the solvent, the bimolecular rate constant *k*_2_ increases moderately when moving from DMAP (**1**) to TCAP (**2**). Meanwhile, we observe a significantly larger increase for pyridinamide ion pairs, which exceed those for **2** by at least one order of magnitude and the respective *k*_2_ values obtained in MeCN by approximately two orders of magnitude.

**Table 4:** Second-Order Rate Constants *k*_2_ of the Reactions of DMAP (1), TCAP (2), and Pyridinamide Salts with Reference Electrophiles **8a**, **8b**, and **8c** in DCM (at 20 °C).

	*k***_2_** (M^–1^ s^–1^)		
**IP**	**8a**	**8b**	**8c**
**1**^a^	6.45 × 10^3^	9.84 × 10^3^	4.96 × 10^4^
**2**^b^	1.42 × 10^4^	3.11 × 10^4^	1.28 × 10^5^
**3a**^b^^,^^c^	5.42 × 10^5^	1.25 × 10^6^	4.64 × 10^6^
**3b**^c^	4.92 × 10^5^	1.30 × 10^6^	4.51 × 10^6^
**3c**^c^	3.38 × 10^5^	8.32 × 10^5^	3.17 × 10^6^
**3d**^c^	3.57 × 10^5^	9.06 × 10^5^	3.45 × 10^6^
**4a**^b^^,^^c^	1.69 × 10^6^	4.19 × 10^6^	1.15 × 10^7^
**5a**^c^	1.79 × 10^6^	3.99 × 10^6^	1.23 × 10^7^
**6a**^c^	1.14 × 10^6^	3.08 × 10^6^	9.48 × 10^6^

a Second-order rate constants *k*_2_ from Ref (21).

b Second-order rate constants *k*_2_ from Ref (17).

c Determined at constant ionic strength *I* = 1.0 mM with the mixed sandwich association model.

**Figure 4:**
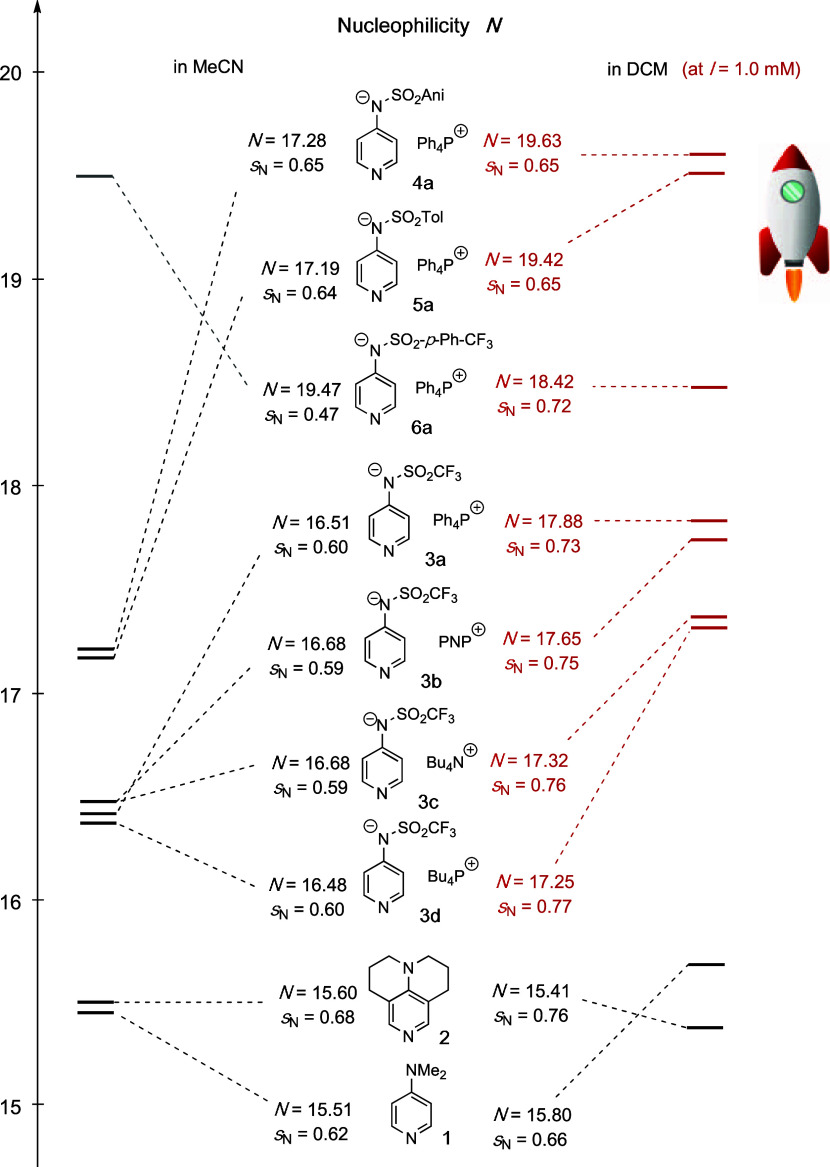
Mayr nucleophilicity parameter *N* (and *s*_N_) of DMAP (**1**), TCAP (**2**), and pyridinamide ion pairs in MeCN and DCM, where all nucleophilicities for ion pairs were obtained by applying the mixed sandwich ion model.

Following the same mode of analysis as in Burger et al.^17^ for our library of pyridinamide ion pairs, we find the four measured rate constants *k*_2_ for anion **3** to be again largely similar for the systems **3a–3d**, the spread of individual values now being somewhat larger at ±23% as compared to acetonitrile. Closer inspection shows the bimolecular rate constants for anion **3** in ion pairs **3a** and **3b** to be quite similar, as are the rate constants for ion pairs **3c** and **3d**. As already found in MeCN, the anion reactivity order in DCM is anion **3** < anion **6** < anion **5** ≈ anion **4**. These measurements establish pyridinamide ion pairs as potent and exceedingly nucleophilic pyridine derivatives in solvents of low polarity (Figure 4). Pyridinamide ion pair **4a** displays the highest nucleophilicity, which is in full agreement with the results for selected benchmark reactions performed in CDCl_3_ as solvent.^15,16^ The pyridinamide ion pairs studied here are thus significantly more reactive in DCM as the reaction medium compared to cyclic guanidines such as 1,5,7-triazabicyclo[4.4.0]dec-5-ene (TBD) identified in earlier studies as the most reactive N-nucleophiles with *N*/*s*_*N*_ = 16.16/0.75.^34^

### Testing for Catalytic Efficiency

The reaction of *n*-butanol (**10**) with *p*-tolyl isocyanate (**9**) to urethane **11** was used as a primary benchmark reaction in earlier studies.^15^ The procedure established by Helberg et al.^15^ was followed to determine the effective rate constants *k*_eff_ for the newly synthesized pyridinamide ion pairs. The role of Lewis base catalysts in this reaction may involve both, the initial Lewis base addition to the isocyanate, or the Lewis base complexation of reactant alcohols.^24−27^

**Figure 5:**
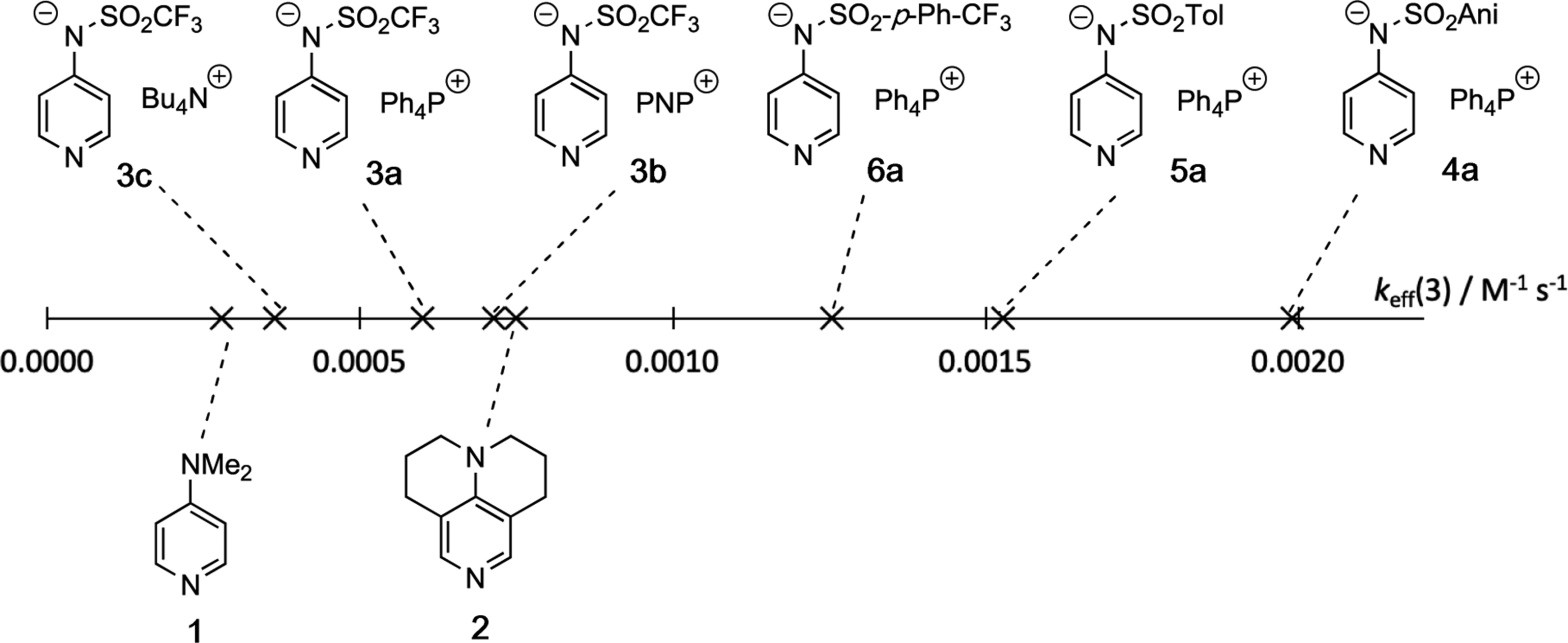
Effective rate constants *k*_eff_ for the benchmark reaction shown in Scheme 1 with 3.0 mol % of catalyst. Data for **1**, **2**, **3c**, and **4a** were taken from Ref (15).

Reaction progress was quantified by ^1^H NMR spectroscopy via the integration of well-separated signals. Effective rate constants were obtained by numerical simulations of the turnover curves based on an effective second-order mechanism as described by Helberg et al.^15^ TCAP (**2**) is the most reactive neutral organocatalyst and, as expected based on the nucleophilicity measurements, pyridinamide salt **4a** catalyzes the urethane reaction three times faster than TCAP (**2**), followed by salts **5a** and **6a** on the scale of effective rate constants *k*_eff_ (Figure 5). As previous kinetic measurements indicated, **3c** is the least catalytically active ion pair catalyst and is close to DMAP (**1**).

**Scheme 1:**
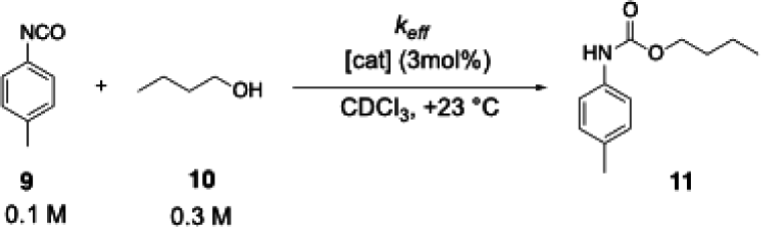
Reaction of *n*-Butanol (10) with *p*-Tolyl Isocyanate (**9**) Employed as Catalytic Benchmark Reaction

**Figure 6:**
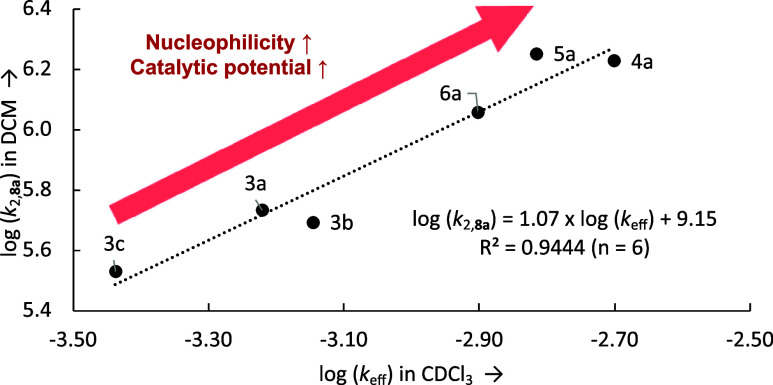
Correlation of log *k*_eff_ vs log *k*_2_(**8a**) for pyridinamide ion pair catalysts.

The effective rate constants *k*_eff_ obtained in the catalytic benchmark reaction for pyridinamide salts correlate linearly with the rate constants *k*_2_ determined for the reaction with benzhydryl cation **8a** as shown in the double logarithmic plot in Figure 6. The good fidelity of this correlation implies that neither the change in solvent from DCM to CDCl_3_ nor the change in the concentration regime is of major importance for the systems studied here. The reactivity parameters obtained from the ionic strength-controlled benzhydrylium method can, either in the form of the individual *k*_*2*_ values or in their condensed form as *N*/*s_N_* parameters, thus be expected to be highly useful in the development and analysis of Lewis base-mediated catalytic reactions involving pyridinamide ion pair catalysts (Scheme 1).

### p*K*_a_ Values

In order to test whether the reactivities of pyridinamide anions **3–6** toward electrophiles correlate with their Brønsted basicities, their p*K*_*a*_ values were determined in three water/cosolvent mixtures using a spectrophotometric titration method at 25 °C (Table 5, for details see the Supporting Information). The ionic strength was controlled with KCl in the buffer solution and set to *I* = 0.3 M. A first p*K*_a_ value (referred to as p*K*_a1_) can be determined for deprotonation of the protonated pyridine sulfonamide (**PA_H**), while the second p*K*_a_ value (referred to as p*K*_a2_) describes deprotonation of pyridine sulfonamide (**PA**) towards anions **3**-**6**. That neutral sulfonamides **PA** have a preference for the imino tautomeric form shown in Table 5 is supported by X-ray crystal structures of these systems.^15^ The assignment of the tautomeric form **PA_H** is only tentative and rests on X-ray crystal structures of complexes of **PA** with protic solvents. The UV/Vis absorbance of the substrate is measured across the full pH range. For p*K*_a2_, the pH-absorbance plot is fitted to eq 4, where *A*_obs_ is the observed absorbance, *A*_max_ is the maximum absorbance of the neutral compound, *A*_min_ is the minimum absorbance (at which the substrate is fully deprotonated), pH is that of the used buffer, and *K*_a_ is the acid dissociation constant for the substrate.
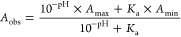
4

**Table 5:**
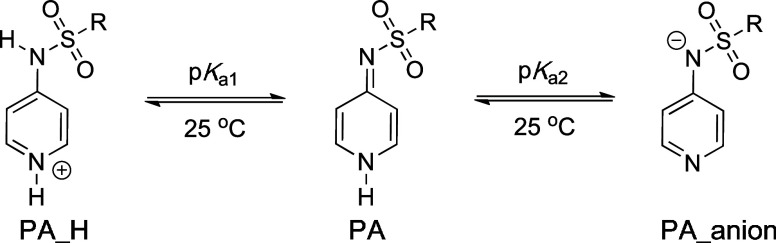
List of Measured p*K*_a_ Values for Pyridinamides (PA) in Selected Aqueous Solvent Mixtures at 25 °C

**PA**	p*K*_a1_	p*K*_a2_	Solvent mixture (v/v)
**1 (DMAP)**	9.85	–	H_2_O + 2.5% MeCN
**3**	–	7.62^a^	H_2_O + 2.5% MeCN
**4**	3.57^a^	8.85^a^	H_2_O + 2.5% MeCN
**5**	3.44^a^	8.83^a^	H_2_O + 2.5% MeCN
**6**	2.69^a^	8.65^a^	H_2_O + 2.5% MeCN
**1 (DMAP)**	8.73^a^^,^^b^	–	H_2_O/MeCN = 1:1
IP_3a	–	7.31^a^^,^^b^	H_2_O/MeCN = 1:1
**IP_4a**	3.46^a^^,^^b^	8.70^a^^,^^b^	H_2_O/MeCN = 1:1
**IP_5a**	3.32^a^^,^^b^	8.67^a^^,^^b^	H_2_O/MeCN = 1:1
**IP_6a**	2.45^a^^,^^b^	8.35^a^^,^^b^	H_2_O/MeCN = 1:1
**3**	7.46^c^	–	H_2_O/DMSO = 1:1
**4**	n.d	8.97^c^	H_2_O/DMSO = 1:1
**5**	n.d	8.92^c^	H_2_O/DMSO = 1:1
**6**	n.d	8.57^c^	H_2_O/DMSO = 1:1

a Averaged p*K*_a_ values over two measurement series.

b Conversion factor −0.257 for 50% MeCN^29^ content applied on pH of the baseline.

c Single measurement series.

The p*K*_a_ of the pyridinamide compounds was determined in aqueous solutions, H_2_O/MeCN mixtures (volume ratio = 1:1), and H_2_O/DMSO mixtures (volume ratio = 1:1). The p*K*_a_ of DMAP was chosen as a reference and is in agreement with literature values.^28^ To exclude a potential interference of the countercation when using pyridinamide salts instead of neutral sulfonamide for the p*K*_a_ determination, pyridinamide ion pair **5a** was measured under the same conditions (see , Chapter S6).

**Figure 7:**
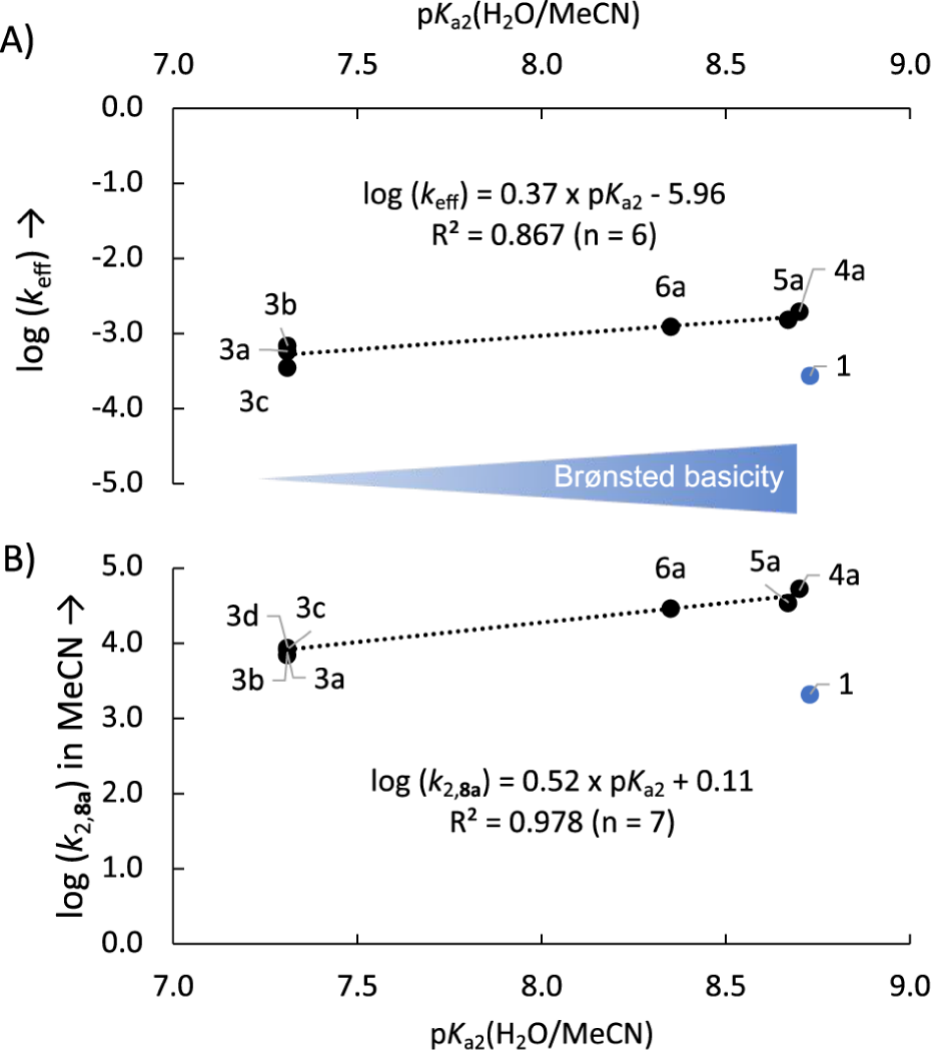
(A) Correlation of log(*k*_eff_) vs p*K*_a2_ values in H_2_O/MeCN = 1:1 for pyridinamide ion pairs **3a-c, 4–6a** and p*K*_a1_ of DMAP (**1**). (B) Correlation of log(*k*_2_,**_8a_**) in MeCN vs. p*K*_a2_ values in H_2_O/MeCN = 1:1 for pyridinamide ion pairs **3a–d, 4–6a** and p*K*_a1_ of DMAP (**1**). The results for DMAP were excluded from the correlation in both cases.

The observed p*K*_a_ values of sulfonamides were similar in all employed solvent mixtures with sulfonamide **4** being the most basic one in all cases. The overall basicity trend **3** < **6** < **5** < **4** also remains the same when switching solvents. Contrary to the sulfonamides, the p*K*_a_ value of DMAP responds strongly to changes in solvent: moving from almost pure water to the 1:1 water/MeCN mixture lowers the p*K*_a_ value from +9.85 to +8.73. Correlating the measured p*K*_a_ values of the sulfonamide anions in the 1:1 H_2_O/MeCN solvent mixture with the logarithmic rate constant for reactions of pyridinamide salts with electrophile **8a** in MeCN shows a good correlation. However, this correlation appears not to be valid for neutral pyridine Lewis bases such as DMAP (**1**) (Figure 7).

### Computational Studies

To quantify the Lewis basicity of pyridinamide ion pairs, we calculated methyl cation affinities (MCA) at the SMD(DCM)/B3LYP-D3/6-31+G(d) level of theory. This type of relative affinity value has previously been shown to correlate well with experimentally determined rate constants.^15,30,31^ In agreement with those studies, pyridine was chosen as the reference base for the group transfer reaction shown in eq 5 and the relative Lewis basicity toward Me^+^ (ΔMCA) was calculated as the free reaction energy at 298.15 K.

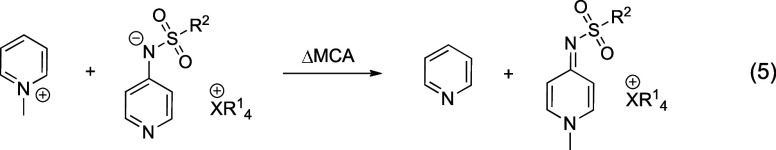



**Figure 8:**
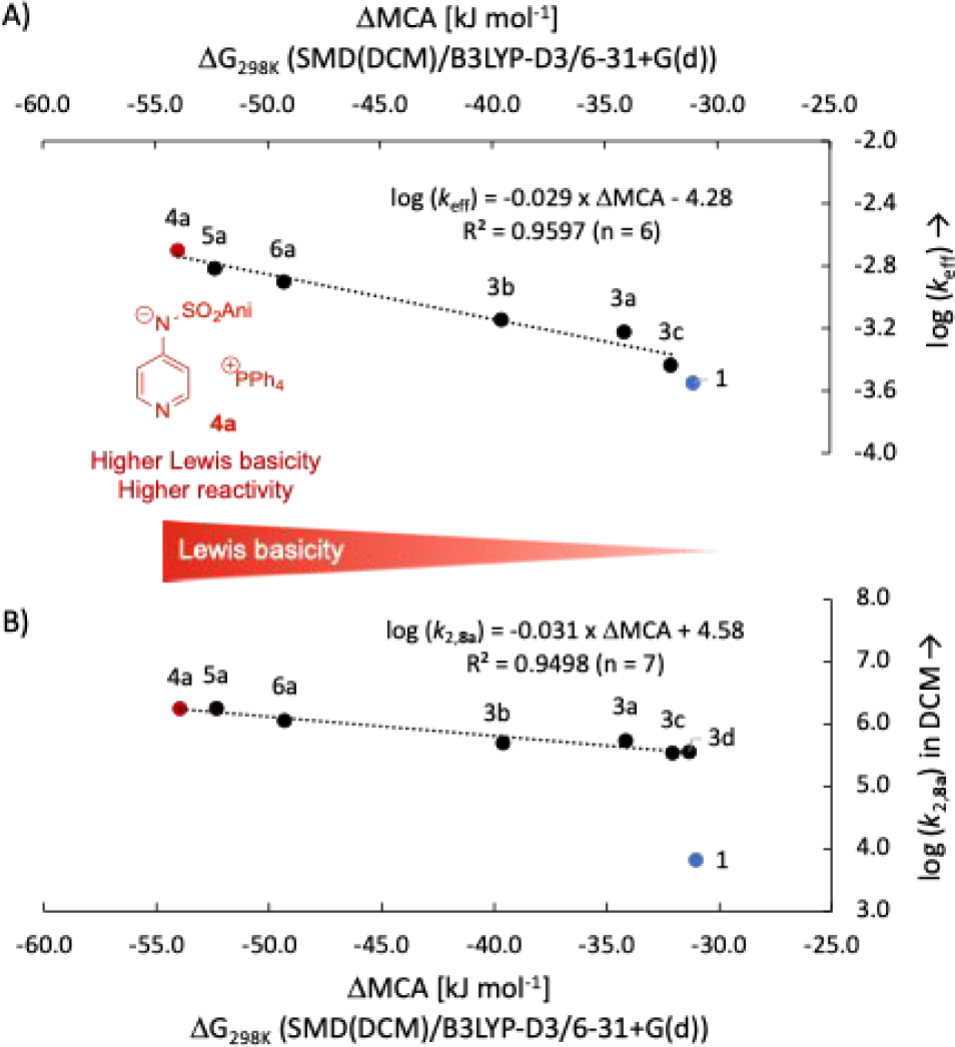
(A) Correlation of effective rate constant log(*k*_eff_) for urethane synthesis (3.0 mol % catalyst loading) with Lewis basicity parameter ΔMCA, and (B) correlation of bimolecular rate constant log(*k*_2,**8a**_) with Lewis basicity parameter ΔMCA calculated at the SMD(DCM)/B3LYP-D3/6-31+G(d) level of theory. The results for DMAP were excluded from the correlation in both cases.

The resulting ΔMCA values are plotted against the experimentally obtained effective rate constant *k*_eff_ for the synthesis of urethane **11** with a catalyst load of 3.0 mol % in Figure 8, which shows a strong correlation between these two parameters. The relative Lewis basicity parameter ΔMCA displays the same general trend observed for the nucleophilicity and p*K*_a_ values and in the effective rate constant *k*_eff_ in the urethane benchmark reaction. In all measurements, the most basic pyridinamide ion pair **4a** achieves the highest value. It should be added that higher catalyst basicity may^32^ or may not^33^ translate into higher reaction rates of catalytic transformations with pyridine-derived Lewis bases.

## Conclusion

By employing a combination of conductivity, DOSY NMR, and kinetic measurements for a focused library of pyridinamide ion pairs, we were able to elucidate the complex asymmetric association behavior of these ions, which impacts their catalytic performance. Key insights include the strong dependence of the association pattern on the size and structure of the cation, which significantly influences the balance between cationic and anionic sandwich association. The association constants of ion pair **3a**–**d** derived from conductivity data revealed a lower degree of association in ion pairs with larger aryl-substituted cations, which also correlates with their catalytic activity. DOSY NMR results further support this theory and show a predominant cationic sandwich association in ion pairs with small anion and large cation volumes, which is essential for maintaining a higher concentration of free nucleophilic anions in solution.

Kinetic studies using the ionic strength-controlled benzhydrylium method confirmed a superior nucleophilicity of pyridinamide anions in DCM compared to neutral organocatalysts such as TCAP (**2**). Comparing the bimolecular rate constants *k*_2_ for reactions with cationic reference electrophiles reveals the most nucleophilic pyridinamide compound being anion **4** with a 90 times higher rate constant than TCAP (**2**). This is also reflected in the determined reaction rate of the urethane benchmark reaction, where **4a** catalyzes the reaction seven times more effectively than TCAP (**2**). Kinetic data for the single step nucleophilicity measurements as well as the multistep catalytic benchmark experiments correlate well with the Brønsted and Lewis basicities of the respective ion pair systems. These latter quantities thus represent, together with the volume parameters obtained from PCM-type calculations, valuable guidelines for the future design of highly reactive ion pair systems.

## Data Availability

The data underlying this study are available in the published article and its Supporting Information.

## Supporting Information Available

Additional experimental and computational details, single crystal X-ray data, analysis procedures and methods, including step by step descriptions (PDF)

## Abbreviations

DCM: dichloromethane
DMAP: 4-dimethylaminopyridine
DMSO: dimethyl sulfoxide
DOSY: diffusion-ordered NMR spectroscopy
IP: ion pair
MCA: methyl cation affinity
MeCN: acetonitrile
PA: pyridinamide
TCAP: 9-azajulolidine
