# Supplementary material for: Pyridinamide Ion Pairs: Design Principles for Super-Nucleophiles in Apolar Organic Solvents
Source: J Org Chem. 2025 Jan 31;90(6):2298–306. doi: 10.1021/acs.joc.4c02668 (PMC11833877; doi:10.1021/acs.joc.4c02668)
Supplement: Supplementary file 1 — jo4c02668_si_001.pdf [file jo4c02668_si_001.pdf]

Supporting information

Pyridinamide Ion Pairs – Design Principles for Super-Nucleophiles in Apolar Organic Solvents

Veronika Burger[a], Maximilian Franta[b], AnnMarie C. O'Donoghue*[c], Armin R. Ofial*[a], Ruth M. Gschwind*[b], and Hendrik Zipse[*a]

[a]Department of Chemistry, Ludwig-Maximilians-Universität München, Butenandtstr. 5-13, 81377 München, Germany.

[b]Institute for Organic Chemistry, University Regensburg, Universitätsstr. 31, 93053 Regensburg, Germany.

[c] Department of Chemistry, Durham University, South Road, Durham DH1 3LE, United Kingdom.

Table of contents

1. Additional Figures and Correlations S3

2. Synthesis of Compounds S7

2.1 Synthesis of Neutral Pyridinamides S7

2.2 Synthesis of Ion Pair Catalysts S8

2.3 Synthesis of Additives S12

3. Conductometric Measurements S14

3.1 Sample Preparation and Data Acquirement S14

3.2 Conductivity Data Analysis S14

3.3 Model I – Double 1:1 Ion Pair Association S16

3.4 Model IIa – Cationic Sandwich Association S17

3.5 Model IIb – Anionic Sandwich Association S19

3.6 Model III – Mixed Sandwich Association S20

3.7 Workflow summary S23

3.8 Conductivity data in MeCN S24

3.9 Pyridinamide ion Pairs – Conductivity data in DCM S29

3.10 Additive Salts – Conductivity data in DCM S36

4. DOSY NMR Spectroscopy S40

5. Nucleophilicity Data S46

5.1 Kinetic Data Analysis S46

5.2 Nucleophilicity data in MeCN S48

5.3 Nucleophilicity data in DCM at 0.01 – 0.03 mM S53

5.4 Nucleophilicity data in DCM at constant ionic strength I = 1.0 mM S59

5.5 Effects of Ion Association on Nucleophilicity S65

6. pK_a measurements S68

6.1 General Information – pK_a measurements in aqueous solutions S68

6.2 General Information – pK_a measurements in MeCN mixtures S77

6.3 General Information – pK_a measurements in DMSO mixtures S83

7. NMR Kinetics S87

8. Crystallographic Data S108

9. NMR Spectra S112

10. DOSY Fits S125

11. Computational General Information S145

11.1 Optimized Conformers of Pyridinamide Ion Pairs in DCM S150

11.2 Calculations of Methyl Cation Affinity (MCA) values S165

11.3 Conformers of Pyridinamide Triple Ion Complexes in DCM S174

11.4 Optimized Conformers of Additive Salts in DCM S182

11.5 Optimized Conformers and Methyl Cation Affinities of Neutral Lewis Base Catalysts in DCM S187

11.6 XYZ-Coordinates of Most Stable Compounds S189

References S252

General

All reagents were purchased from Sigma Aldrich, TCI, or Acros and used without further purification unless otherwise noted. Solvents were obtained from Acros Organics, Sigma Aldrich, or Merck and purified by simple distillation in a rotary evaporator, unless otherwise specified.

All air- and moisture-sensitive reactions were performed under a nitrogen atmosphere, the glassware, and magnetic stirrers were dried in a dry oven at 110 °C overnight. $CDCl_3$ and NEt_3 were dried over CaH_2 and distilled prior to use. MeCN-d_3, CD_2Cl_2 and $CDCl_3$ were dried over 4Å MS.

CH_2Cl_2 for nucleophilicity measurements was stirred over concentrated H_2SO_4 for two weeks before extraction with water (1 x 1.0 L), $NaHCO_3$ (1 x 1.0 L), and again water (1 x 1.0 L). CaH_2 was added as a drying agent, and the solvent was freshly distilled over CaH_2 prior to use.

Melting points were acquired using Büchi Melting Point M-560 devices and are uncorrected.

Nuclear magnetic resonance (NMR) spectra were recorded on a Bruker 400 MHz or INOVA 400 and 600 MHz machines. The following abbreviations were used in the analysis of NMR spectra: s = singlet, d = doublet, t = triplet, q = quartet, m = multiplet, br s = broad singlet. NMR signals were assigned based on 2D spectra (COSY, HSQC, HMBC, NOESY) experiment analysis. Chemical shifts are given in ppm. The internal reference was set to the residual solvent signals (CD_2Cl_2, $CDCl_3$, DMSO-d_6). The ^{13}C NMR spectra (101 or 151 MHz) were recorded under broadband proton-decoupling. ^{19}F spectra were referenced using the solvent signal.[1] The spectra were imported and processed in the program MestreNova (version 14.1.1).

Infrared (IR) spectra were measured on a Perkin Elmer Spectrum BX-59343 instrument with a Smith Detection DuraSamplIR II Diamond ATR sensor for liquids or neat for solids. Intensities are described as vs = very strong, m = medium, w = weak, br = broad.

High-resolution mass spectra (HRMS) were recorded on a Thermo Finnigan LTQ FT Ultra Fourier Transform Ion Cyclotron Resonance mass spectrometer with electrospray ionization (ESI) for sample ionization. For EI (70 eV) measurements a Thermo Finnigan of the MAT 95 type with a direct exposure probe (DEP) was used.

Crystal structures were recorded using an Oxford Diffraction XCalibur with Sapphire CCD-detector and a molybdenum-K_α-source (λ = 0.71073) with a concentric circle kappa-device. The structures were resolved using the program SHELXS or SIR97 and refined with SHELXS.

1. Additional Figures and Correlations

Conductivity profiles in MeCN for all pyridinamide ion pairs are depicted in Figure S1.

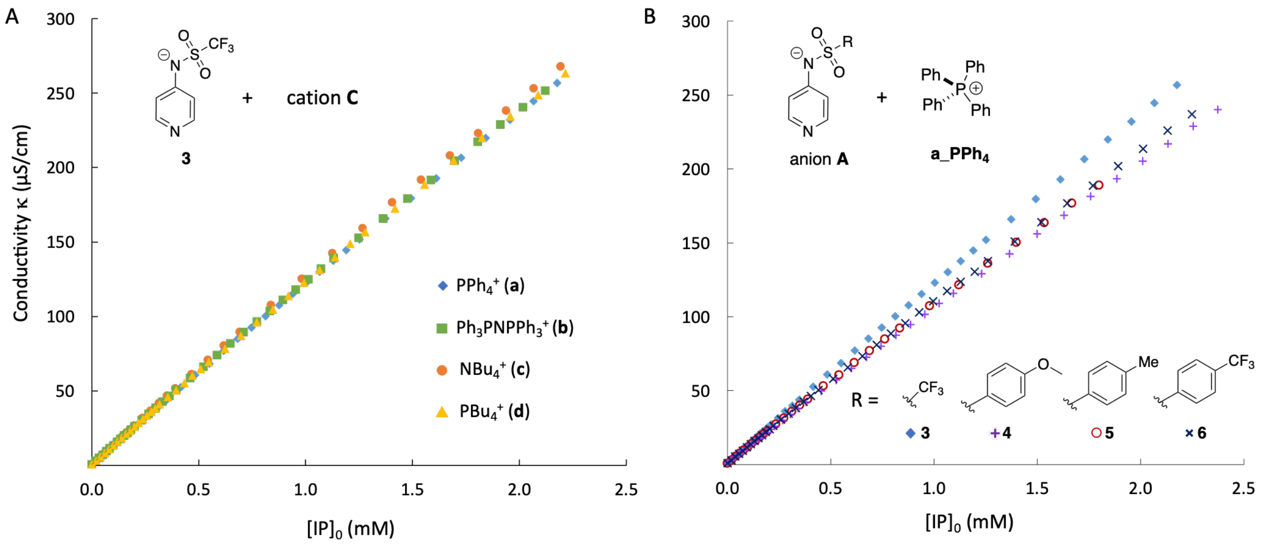

Figure S1. A) Conductivity profiles for **3a-d** measured in MeCN at 20 °C, B) Conductivity profiles for **3-6a** measured in MeCN at 20 °C.

The association constant K_{IP} for the association in MeCN was determined according to the simple 1:1 ion association model described in Zipse *et al.*[2] The resulting association constants K_{IP} (M^{-1}) are summarized in Table S1.

Table S1. Association constants K_{IP} (M^{-1}) of pyridinamide ion catalysts **3a-d,4-6a** at 20 °C in MeCN.

System	Λ_m (S cm^2 mol^{-1})	K_{IP} (M^{-1})
**3a**	126	34.6 ± 0.29
**3b**	130	50.6 ± 0.50
**3c**	133	44.0 ± 0.63
**3d**	129	44.0 ± 0.34
**4a**	110	42.9 ± 0.33
**5a**	113	44.5 ± 0.80
**6a**	115	49.3 ± 0.55

Additional correlations were performed for pK_a values with the respective effective rate constants of the urethane benchmark reaction. Both experimental values correlate strongly with each other in Figure S2, however, adding the results of DMAP to the data set demonstrated that this is not a universal correlation for pyridine-based compounds.

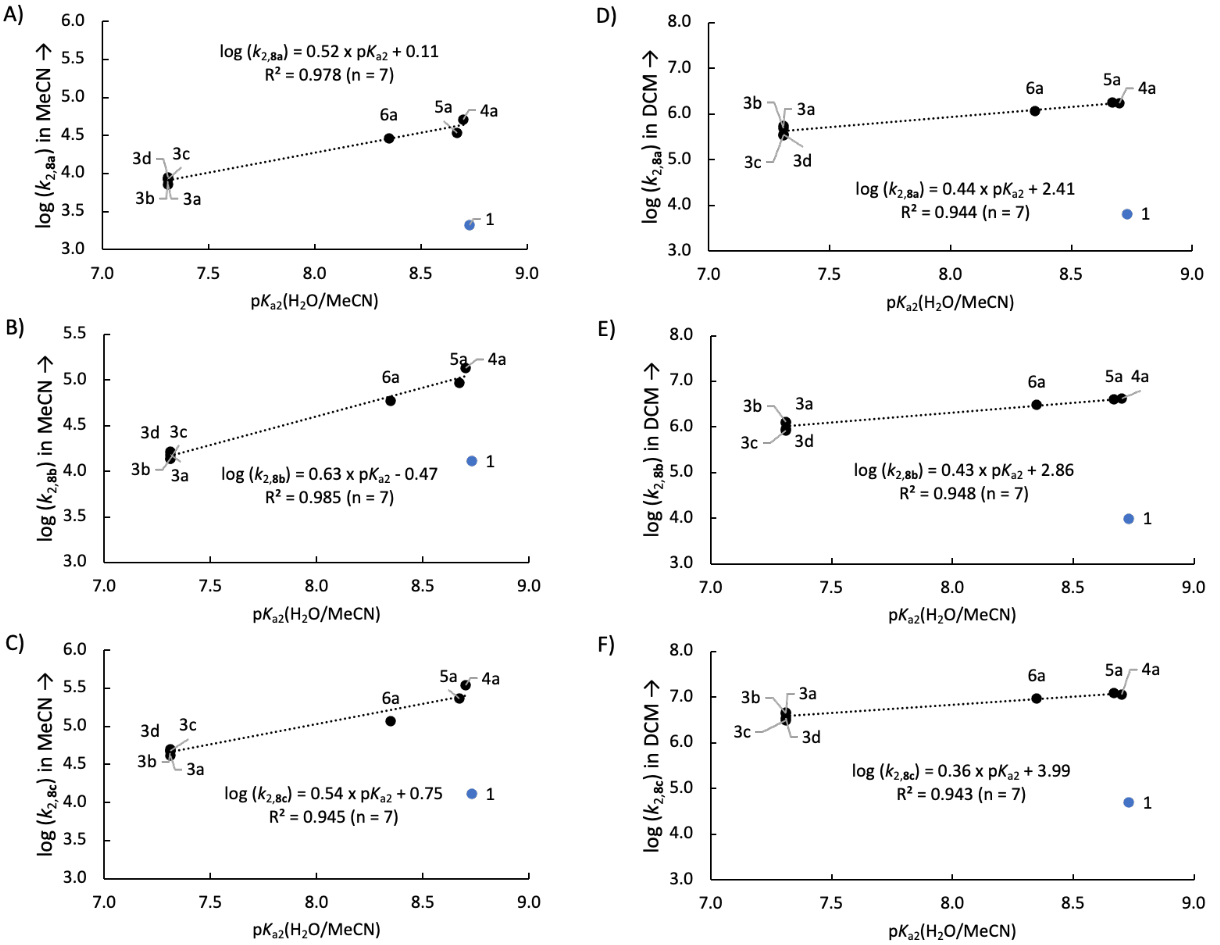

Figure S2. Correlation of A) log ($k_{2,\mathbf{8a}}$) in MeCN, B) log ($k_{2,\mathbf{8b}}$) in MeCN, C) log ($k_{2,\mathbf{8c}}$) in MeCN, D) log ($k_{2,\mathbf{8a}}$) in DCM, E) log ($k_{2,\mathbf{8b}}$) in DCM, and F) log ($k_{2,\mathbf{8c}}$) in DCM with pK_a values measured in H_2O/MeCN = 1:1 for pyridinamide salts **3-6a**.

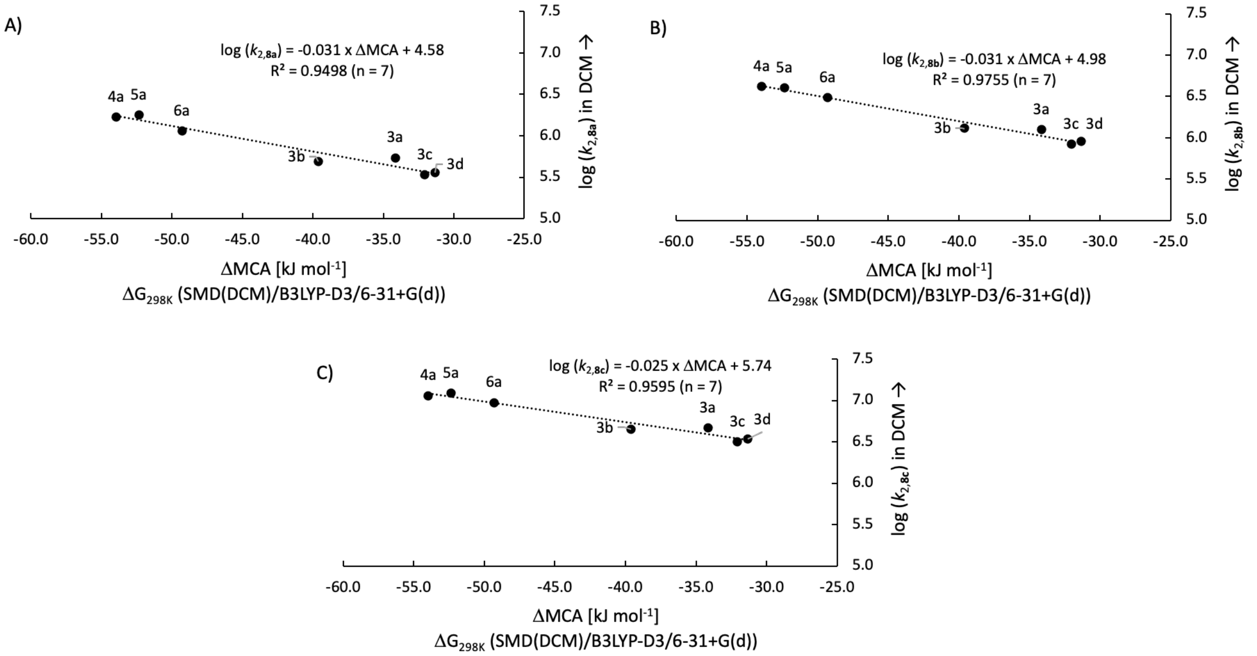

Figure S3. Correlation of A) bimolecular rate constant log ($k_{2,\mathbf{8a}}$), B) bimolecular rate constant log ($k_{2,\mathbf{8b}}$), C) bimolecular rate constant log ($k_{2,\mathbf{8c}}$) with Lewis basicity parameter ΔMCA calculated at the SMD(DCM)/B3LYP-D3/6-31+G(d) level of theory.

In the Tables S2 and S3 below the most important results of the conductivity data analysis is summarized to enable a quick and easy overview over the specific molar conductivities Λ_m (S cm^2 mol^{-1}), the limited ion conductivities λ_i (S cm^2 mol^{-1}) of all charged species, their scaling factors δ_i and the resulting association constant of all applied model (see Chapter 3 for details).

Table S2. Specific molar conductivities Λ_m (S cm^2 mol^{-1}) with the corresponding limited ion conductivities λ_i (S cm^2 mol^{-1}) with their scaling factor δ_i of all charged species and the resulting ion association constant K_{IP} (M^{-2}), the cationic and anionic sandwich association constants K_{CAC} and K_{ACA} (M^{-2}) with the respective RMSE values for all pyridinamide ion pairs and additives.

Ion Pair	Λ_m (S cm^2 mol^{-1})	λ_A (S cm^2 mol^{-1})	λ_C (S cm^2 mol^{-1})	λ_{CAC} (S cm^2 mol^{-1})	λ_{ACA} (S cm^2 mol^{-1})	δ_A /δ_C /δ_{CAC}/δ_{ACA}	K_{IP} (M^{-2}) (RMSE)	K_{CAC} (M^{-2}) (RMSE)	K_{ACA} (M^{-2}) (RMSE)
**3d**	56.0	22.4	33.6	8.40	–	40/60/15/–	7.35×10^6 (0.33)	1.07×10^7 (0.09)	–
**3c**	57.9	23.7	34.2	12.7	2.32	41/59/22/4	5.00×10^6 (0.26)	1.01×10^7 (0.10)	1.01×10^7 (0.10)
**3b**	80.3	22.4	57.8	73.1	37.8	28/72/91/47	2.96×10^5 (0.32)	4.65×10^6 (0.11)	4.65×10^6 (0.11)
**3a**	79.2	29.3	49.9	53.1	32.5	37/63/67/41	6.86×10^5 (0.30)	6.38×10^6 (0.17)	6.38×10^6 (0.17)
**5a**	73.2	30.7	42.5	41.7	30.0	42/58/57/41	6.92×10^5 (0.23)	5.15×10^6 (0.13)	5.15×10^6 (0.13)
**4a**	73.6	32.4	41.2	39.7	30.9	44/56/54/42	8.79×10^5 (0.27)	6.50×10^6 (0.12)	6.50×10^6 (0.12)
**6a**	74.2	33.4	40.8	44.5	37.1	45/55/60/50	6.89×10^5 (0.32)	6.75×10^6 (0.12)	6.75×10^6 (0.12)
**7a**	95.2	15.2	80.0	76.2	–	16/84/80/–	1.01×10^6 (0.33)	7.05×10^6 (0.16)	–
**7b**	91.3	10.0	81.3	90.4	–	11/89/99/–	3.98×10^5 (0.47)	4.61×10^6 (0.24)	–
**7c**	68.2	12.3	55.9	23.2	–	18/82/34/–	2.49×10^7 (0.22)	3.51×10^7 (0.15)	–
**7d**	59.9	10.8	49.1	18.0	–	18/82/30/–	3.04×10^7 (0.21)	3.46×10^7 (0.12)	–

Table S3. Cationic and anionic sandwich association constants K_{CAC} and K_{ACA} (M^{-2}) with the respective RMSE values as result of the separated models and the mixed sandwich association model with their scaling factors α and β as well as the reference DOSY ion volumes and the calculated ion volumes with their percentual residual at c = 1.0 mM for all pyridinamide ion pairs.

Ion Pair	K_{CAC} (M^{-2}) (RMSE)	K_{ACA} (M^{-2}) (RMSE)	α/β	$\alpha \times K_{CAC}$ (M^{-2})	$\beta \times K_{ACA}$ (M^{-2})	RMSE	V_{an} (Å^3)	V_{cat} (Å^3)	Residual (%) Anion	Residual (%) Cation
**DOSY ref(3c)**							**571**	**660**		
**3c**	1.01×10^7 (0.10)	1.01×10^7 (0.10)	33/23	3.33×10^6	2.32×10^6	0.61	535	619	-6.21	-6.23
**DOSY ref(3b)**							**473**	**944**		
**3b**	4.65×10^6 (0.11)	4.65×10^6 (0.11)	100/0	4.65×10^6	0.00	0.11	590	1076	+24.7	+14.0
**DOSY ref(3a)**							**505**	**642**		
**3a**	6.38×10^6 (0.17)	6.38×10^6 (0.17)	44/21	2.81×10^6	1.34×10^6	0.43	533	679	+5.61	+5.80
**DOSY ref(5a)**							**727**	**652**		
**5a**	5.15×10^6 (0.13)	5.15×10^6 (0.13)	11/67	5.67×10^5	3.45×10^6	0.29	670	601	-7.90	-7.79
**DOSY ref(4a)**							**765**	**682**		
**4a**	6.50×10^6 (0.12)	6.50×10^6 (0.12)	12/61	7.80×10^5	3.97×10^6	0.35	706	630	-7.79	-7.67

**DOSY ref(6a)**							**772**	**714**		
**6a**	6.75×10^6 (0.12)	6.75×10^6 (0.12)	16/52	1.08×10^6	3.51×10^6	0.38	711	657	-7.90	-7.93

2. Synthesis of Compounds

In this supporting information the data for the following ion pair (see Chart S1) will be discussed. Pyridinamide ion pair **3a** will be used as reference system.

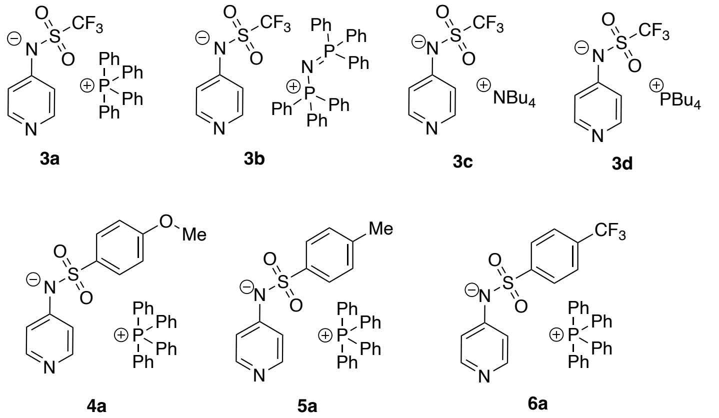

Chart S1. Pyridinamide ion pair library.

2.1 Synthesis of Neutral Pyridinamides

1,1,1-Trifluoro-*N*-(pyridin-4(1*H*)-ylidene)methanesulfonamide (PA_**3**)[2]

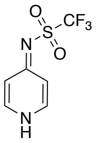

PA_3

4-Aminopyridine (2.37 g, 25.2 mmol, 1.0 eq) was dissolved in dry pyridine (30.0 mL) under nitrogen atmosphere. Triethylamine (8.00 mL, 57.4 mmol, 2.3 eq) was added and stirred for 10 min. The reaction mixture was cooled to 0 °C and trifluoromethanesulfonyl chloride (5.00 g, 29.7 mmol, 1.2 eq) was added. After stirring for 10 min at 0 °C, the mixture was refluxed for 3.5 h under nitrogen atmosphere before the solvent was removed. The crude product was suspended in H_2O, refluxed for 20 min and filtered while hot. This process was repeated with acetone and MTBE, yielding pyridinamide **3** (3.55 g, 15.7 mmol, 62%) as a light brown solid.

^{1}H NMR (400 MHz, DMSO-*d*$_6$): δ [ppm] = 13.60 (s, 1H), 8.28 (d, *J* = 7.3 Hz, 2H), 7.27 (d, *J* = 7.3 Hz, 2H).

^{13}C{^{1}H} NMR (101 MHz, DMSO-*d*$_6$): δ [ppm] = 163.7, 140.2, 120.7 (q, *J* = 325.6 Hz), 116.9.

^{19}F NMR (377 MHz, DMSO-*d*$_6$): δ [ppm] = -77.69.

HRMS (ESI): calc. for $C_6H_5F_3N_2O_2S^+$ [M$^+$]: 226.0018, found 226.0019.

4-Methoxy-*N*-(pyridin-4(1*H*)-ylidene)benzenesulfonamide (PA_**4**)[2]

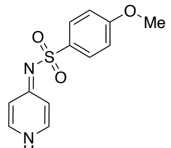

PA_4

4-Aminopyridine (2.03 g, 21.6 mmol, 1.0 eq) and NEt_3 (8.50 mL, 61.0 mmol, 2.8 eq) were dissolved in pyridine (22.0 mL). The mixture was cooled to 0 °C and 4-methoxybenzenesulfonyl chloride (5.35 g, 25.9 mmol, 1.2 eq) was added and stirred for 20 min. The reaction mixture was refluxed for 3 h before being cooled down to rt. The solvent was removed, and the resulting precipitate was repeatedly refluxed in H_2O, acetone, and MTBE. The final product was dried *in vacuo*. Pyridinamide **4** (4.78 g, 18.1 mmol, 84%) was obtained as an off-white solid.

^{1}H NMR (400 MHz, DMSO-*d*$_6$): δ [ppm] = 12.1 (s, 1H), 8.04 (d, *J* = 6.5 Hz, 2H), 7.74 (d, *J* = 8.9 Hz, 1H), 7.03 (d, *J* = 8.9 Hz, 1H), 6.95 – 6.89 (m, 2H), 3.79 (s, 3H).

^{13}C{^{1}H} NMR (101 MHz, DMSO-*d*$_6$): δ [ppm] = 161.7, 151.1, 141.7, 134.4, 128.3, 114.1, 113.8, 55.5.

HRMS (ESI): calc. for $C_{12}H_{12}N_2O_3S^+$ [M$^+$]: 264.0563, found 264.0567.

4-Methyl-*N*-(pyridin-4(1*H*)-ylidene)benzenesulfonamide (PA_**5**)

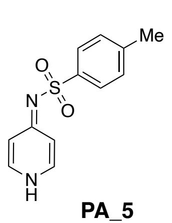

PA_5

Following a modified procedure of Helberg and Zipse,[3] 4-aminopyridine (2.00 g, 1.0 eq, 21.3 mmol) was dissolved in pyridine (22.0 mL). Triethylamine (8.00 mL, 57.4 mmol, 2.3 eq) was added and the mixture was stirred for 10 min. After cooling to 0 °C, 4-methylbenzenesulfonyl chloride (4.86 g, 1.2 eq, 25.5 mmol) was added and the mixture was stirred at 0 °C for 30 min. Subsequently, the reaction mixture was refluxed for 3 h before being cooled down to rt. The solvent was evaporated, and the crude product was suspended in H_2O, refluxed for 20 min and filtered while hot. This process was repeated with ethanol and MTBE, yielding pyridinamide **5** (4.78 g, 19.59 mmol, 91%) as an off-white solid.

^{1}H NMR (400 MHz, DMSO-*d6*): δ [ppm] = 12.3 (s, 1H), 8.01 (d, *J* = 6.7 Hz, 2H), 7.71 – 7.65 (m, 2H), 7.30 (d, *J* = 8.0 Hz, 2H), 6.95 – 6.86 (m, 2H), 2.33 (s, 2H).

^{13}C{^{1}H} NMR (101 MHz, DMSO-*d6*): δ [ppm] = 141.6, 140.2, 129.3, 126.2, 114.1, 20.9.

HRMS (ESI): calc. for $C_{12}H_{12}N_2O_2S^+$ [M$^+$]: 248.0614, found: 248.0614.

The spectroscopic data is consistent with the literature.[3]

N-(pyridin-4(1*H*)-ylidene)-4-(trifluoromethyl)benzenesulfonamide (PA_**6**)

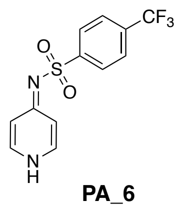

PA_6

4-Aminopyridine (1.00 g, 10.6 mmol, 1.0 eq) was dissolved in dry pyridine (12.0 mL) under nitrogen atmosphere. Triethylamine (3.30 mL, 23.4 mmol, 2.2 eq) was added and the reaction mixture was stirred for 10 min before being cooled down to 0 °C. 4-Trifluoromethylbenzenesulfonyl chloride (2.86 g, 11.7 mmol, 1.1 eq) was added and the mixture was stirred for 20 min before being refluxed for 2.5 h. The solvent was evaporated, and the crude product was suspended in H_2O, refluxed for 20 min and filtered while hot. This process was repeated with ethanol and MTBE, yielding pyridinamide **6** (2.64 g, 8.73 mmol, 82%) was an off-white solid.

^{1}H NMR (400 MHz, DMSO-*d6*): δ [ppm] = 12.8 (s, 1H), 8.00 (dd, *J* = 7.6, 5.4 Hz, 4H), 7.87 (d, *J* = 8.2 Hz, 2H), 6.94 (d, *J* = 7.3 Hz, 2H).

^{13}C{^{1}H} NMR (101 MHz, DMSO-*d6*): δ [ppm] = 162.5, 147.8, 139.2, 131.0 (q, *J* = 32.1 Hz), 126.9, 125.9 (q, *J* = 3.8 Hz), 123.7 (q, *J* = 272.6 Hz), 114.9.

^{19}F NMR (377 MHz, $CDCl_3$): δ [ppm] = -61.4

IR (ATR): ν (cm^{-1}) = 3049 (w), 2660 (w), 1635 (m), 1617 (m), 1477 (s). 1330 (s), 1194 (m), 1140 (vs), 1086 (s), 950 (s), 834 (s), 766 (m), 706 (m).

HRMS (ESI): calc. for $C_{12}H_9F_3N_2O_2S^+$ [M$^+$]: 302.0331, found 302.0335.

m. p: 298-301 °C

2.2 Synthesis of Ion Pair Catalysts

Tetraphenylphosphonium pyridin-4-yl((trifluoromethyl)sulfonyl)amide (**3a**)[2]

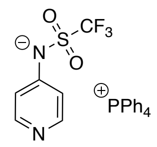

3a

Pyridinamide **3** (402 mg, 1.78 mmol, 1.0 eq) was added to a solution of NaOH (78.2 mg, 1.96 mmol, 1.1 eq) in H_2O (5.0 mL). Tetraphenyl bromide (746 mg, 1.78 mmol, 1.0 eq) was added, and the reaction mixture was stirred at rt. for 1 h. DCM (5.0 mL) was added, and the two phases were separated. The aqueous phase was extracted with DCM (3 x 15 mL). The collected organic phase was dried over $MgSO_4$, filtered and the solvent was evaporated. The crude product was crystallized from DCM overlayered with toluene. Pyridinamide ion pair **3a** (787 mg, 1.39 mmol, 78%) was obtained in form of colorless needles.

^{1}H NMR (400 MHz $CDCl_3$): δ [ppm] = 8.05 – 8.00 (m, 2H), 7.88 – 7.81 (m, 4H), 7.71 (td, *J* = 7.9, 3.6 Hz, 4H), 7.55 (ddd, *J* = 13.0, 8.4, 1.3 Hz, 4H), 7.02 – 6.97 (m, 2H).

$^{13}C\{^1H\}$ NMR (101 MHz, $CDCl_3$): δ [ppm] = 156.1, 149.5, 135.9 (d, *J* = 3.1 Hz), 134.5 (d, *J* = 10.3 Hz), 130.9 (d, *J* = 12.9 Hz), 122.2 (q, *J* = 328.6 Hz), 118.0 (d, *J* = 6.4 Hz), 117.1.

^{19}F NMR (377 MHz, $CDCl_3$): δ [ppm] = -76.8.

^{31}P NMR (162 MHz, $CDCl_3$): δ [ppm] = 23.19.

Elemental Analysis: Anal. calcd. for $C_{30}H_{24}F_3N_2O_2PS$: C, 63.82; H, 4.29; N, 4.96; S, 5.68. Found: C, 63.79; H, 4.32; N, 5.00; S, 5.45.

HRMS (ESI): calc. for $C_6H_4F_3N_2O_2S^-$ [A^-]: 224.9951; found 224.9948; calc. for $C_{24}H_{20}P^+$ [C^+]: 339.1297; found: 339.1289.

Triphenyl ((triphenyl-λ5-phosphaneylidene)amino) phosphonium pyridin-4-yl (trifluoromethyl) sulfonyl) amide (**3b**)

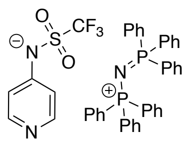

3b

Pyridinamide **3** (349 mg, 1.55 mol, 1.0 eq) was added to a solution of NaOH (67.0 mg, 1.70 mmol, 1.1 eq) in H_2O (5.00 mL). Bis-(triphenylphosphoranyliden)-ammonium chloride (886 mg, 1.55 mmol, 1.0 eq) was added, and the reaction mixture was stirred at rt. overnight. DCM (5.00 mL) was added, and the two phases were separated. The aqueous phase was extracted with DCM (3 x 15 mL). The collected organic phase was dried over $MgSO_4$, filtered and the solvent was evaporated. The crude product was crystallized from DCM overlayered with toluene in a 250 mL flask closed with a septum with one inserted needle, cooled to -40 °C (CO_2/MeCN) and left in the cooling bath overnight. Ion Pair **3b** (952 mg, 1.25 mmol, 81%) was obtained in form of colorless crystals.

^1H NMR (400 MHz, $CDCl_3$): δ [ppm] = 8.10 (d, *J* = 6.6 Hz, 2H), 7.63 (td, *J* = 5.7, 2.6 Hz, 6H), 7.49 – 7.38 (m, 24H), 7.12 (d, *J* = 6.5 Hz, 2H).

$^{13}C\{^1H\}$ NMR (101 MHz, $CDCl_3$): δ [ppm] = 156.3, 149.4, 134.08 – 133.82 (m), 132.41 – 131.92 (m), 129.99 – 129.37 (m), 127.1 (dd, *J* = 107.9, 1.9 Hz), 122.3 (q, *J* = 328.7 Hz), 118.1.

^{19}F NMR (376 MHz, $CDCl_3$): δ [ppm] = -76.8.

^{31}P NMR (162 MHz, $CDCl_3$): δ [ppm] = 21.03.

IR (ATR): ν (cm^{-1}) = 3058 (br), 1588 (m), 1438 (m), 1319 (s), 1288 (vs), 1198 (s), 1155 (s), 1115 (s), 999 (m), 832 (w), 723 (s), 692 (m).

Elemental Analysis: Anal. calcd. for $C_{42}H_{34}F_3N_3O_2P_2S$: C, 66.05; H, 4.49; N, 5.50; S, 4.20. Found: C, 65.84; H, 4.56; N, 5.43; S, 4.21.

HRMS (ESI): calc. for $C_6H_4F_3N_2O_2S^-$ [A^-]: 224.9951; found 224.9952; calc. for $C_{36}H_{30}NP_2^+$ [C^+]: 538.1848; found: 538.1849.

m. p: 139-140 °C

Tetrabutylammonium pyridin-4-yl((trifluoromethyl)sulfonyl)amide (**3c**)

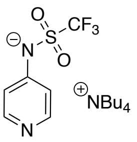

3c

Following a modified procedure of Helberg and Zipse,[3] pyridinamide **3** (400 mg, 1.77 mmol, 1.0 eq) was added to a solution of NaOH (76.3 mg, 1.91 mmol, 1.0 eq) in H_2O (6.00 mL). Tetrabutylammonium bromide (570 mg, 1.77 mmol, 1.0 eq) was added portion-wise. The resulting reaction mixture was stirred at rt. overnight. DCM (6 mL) was added, and the two phases were separated. The aqueous phase was extracted with DCM (3 x 15 mL) and the collected organic phase was dried over $MgSO_4$, filtered, and concentrated *in situ*. The crude product was crystallized from DCM overlayered with toluene and heptane. Pyridinamide ion pair **3c** (710 mg, 1.52 mmol, 86%) were obtained in form of off-white crystals.

^1H NMR (600 MHz, $CDCl_3$): δ [ppm] = 8.16 (d, *J* = 6.4 Hz, 2H), 7.04 (d, *J* = 6.4 Hz, 2H), 3.16 – 3.08 (m, 8H), 1.58 – 1.51 (m, 8H), 1.36 (h, *J* = 7.3 Hz, 8H), 0.95 (t, *J* = 7.4 Hz, 12H).

$^{13}C\{^{1}H\}$ NMR (151 MHz, $CDCl_3$): δ [ppm] = 155.8, 149.6, 122.1 (q, *J* = 328.2 Hz), 118.1 58.8, 24.0, 19.7, 13.7.

^{19}F NMR (377 MHz, $CDCl_3$): δ [ppm] = -76.8.

Elemental Analysis: Anal. calcd. for $C_{22}H_{40}F_3N_3O_2S$: C, 56.51; H, 8.62; N, 8.99; S, 6.86. Found: C, 56.34; H, 8.45; N, 9.00; S, 6.63.

The spectroscopic data is consistent with the literature.[3]

Tetrabutylphosphonium pyridin-4-yl((trifluoromethyl)sulfonyl)amide (**3d**)[3]

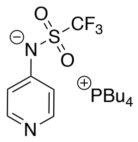

3d

Pyridinamide **3** (402 mg, 1.78 mmol, 1.0 eq) was suspended in tetrabutylphosphonium hydroxide (1.25 mL, 40% in H_2O, 1.78 mmol, 1.0 eq) and H_2O (5.00 mL) was added. The reaction mixture was stirred at rt. for 2 h. DCM (5.00 mL) was added, and the two phases were separated. The aqueous phase was extracted with DCM (3 x 15 mL). The collected organic phase was dried over $MgSO_4$, filtered and the solvent was evaporated. The crude product was crystallized from DCM overlayered with toluene, *iso*-hexane (1.00 mL), and heptane (2.50 mL). The mixture was stored in the fridge overnight. Ion Pair **3d** (718 mg, 1.48 mmol, 88%) was obtained in form of off-white crystals.

^{1}H NMR (600 MHz, $CDCl_3$): δ [ppm] = 8.17 (d, *J* = 6.4 Hz, 2H), 7.04 (d, *J* = 6.5 Hz, 2H), 2.18 – 2.08 (m, 8H), 1.49 – 1.43 (m, 16H), 0.96 – 0.90 (m, 12H).

$^{13}C\{^{1}H\}$ NMR (151 MHz, , $CDCl_3$): δ [ppm] = 155.6, 149.7, 122.1 (q, *J* = 328.1 Hz), 118.1, 23.9 (d, *J* = 15.3 Hz), 23.7 (d, *J* = 4.8 Hz), 18.7 (d, *J* = 47.5 Hz), 13.4.

^{19}F NMR (376 MHz, $CDCl_3$): δ [ppm] = -76.84.

^{31}P NMR (162 MHz, $CDCl_3$): δ [ppm] = 32.98.

Elemental Analysis: Anal. calcd. for $C_{22}H_{40}F_3N_2O_2PS$: C, 54.53; H, 8.32; N, 5.78; S, 6.62. Found: C, 54.41; H, 8.41; N, 5.67; S, 6.89.

The spectroscopic data is consistent with the literature.[3]

Tetraphenylphosphonium ((4-methoxyphenyl)sulfonyl)(pyridin-4-yl)amide (**4a**)[2]

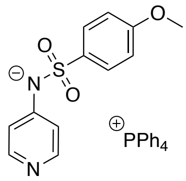

4a

Pyridinamide **4** (501 mg, 1.90 mmol, 1.0 eq) was added to a solution of NaOH (78.9 mg, 1.97 mmol, 1.1 eq) in H_2O (5.0 mL). Tetraphenylphosphonium bromide (795 mg, 1.90 mmol, 1.0 eq) was dissolved in DCM and added dropwise over 30 min through a dropping funnel. The reaction mixture was stirred at rt. for 2 h. More DCM (5.0 mL) was added, and the two phases were separated. The aqueous phase was extracted with DCM (3 x 25 mL). The collected organic phase was dried over $MgSO_4$, filtered and the solvent was evaporated. The crude product was crystallized from DCM overlayered with toluene and heptane (0.5 mL) while stored in the fridge. Ion Pair **4a** (782 mg, 1.30 mmol, 69%) was obtained in form of colorless crystals.

^{1}H NMR (600 MHz, $CDCl_3$): δ [ppm] = 7.90 – 7.79 (m, 1H), 7.77 – 7.69 (m, 1H), 7.61 – 7.53 (m, 1H), 6.79 – 6.72 (m, 4H), 3.74 (s, 3H).

$^{13}C\{^{1}H\}$ NMR (151 MHz, $CDCl_3$): δ [ppm] = 160.5, 157.9, 148.8, 138.8, 138.8, 135.9 (d, *J* = 3.1 Hz), 134.5 (d, *J* = 10.3 Hz), 130.9 (d, *J* = 12.9 Hz), 128.7, 117.5 (d, *J* = 89.5 Hz), 116.3, 113.2, 55.4.

^{31}P NMR (162 MHz, $CDCl_3$): δ [ppm] = 23.03.

Elemental Analysis: Anal. calcd. for $C_{36}H_{31}N_2O_3PS$: C, 71.74; H, 5.18; N, 4.65; S, 5.32. Found: C, 71.35; H, 5.24; N, 4.62; S, 5.77.

Tetraphenylphosphonium pyridin-4-yl(tosyl)amide (**5a**)

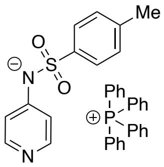

5a

Pyridinamide **5** (449 mg, 1.81 mmol, 1.0 eq) was added to a solution of NaOH (74.3 mg, 1.86 mmol, 1.1 eq) in H_2O (5.00 mL). Tetraphenylphosphonium bromide (746 mg, 1.78 mmol, 1.0 eq) was dissolved in DCM and added dropwise over 10 min through a dropping funnel. The reaction mixture was stirred at rt. for 2 h. DCM (5.00 mL) was added, and the two phases were separated. The aqueous phase was extracted with DCM (3 x 15 mL). The collected organic phase was dried over $MgSO_4$, filtered and the solvent was evaporated. The crude product was crystallized from DCM overlayered with toluene while stored in the fridge. Ion Pair **5a** (905 mg, 1.54 mmol, 85%) was obtained in form of colorless crystals.

^{1}H NMR (400 MHz, $CDCl_3$): δ [ppm] = 7.90 – 7.80 (m, 8H), 7.75 (td, *J* = 7.8, 3.6 Hz, 8H), 7.64 – 7.54 (m, 8H), 7.08 (d, *J* = 7.8 Hz, 2H), 6.76 – 6.72 (m, 2H), 2.28 (s, 3H)

^{13}C{^{1}H} NMR (101 MHz, $CDCl_3$): δ [ppm] = 157.9, 148.6, 143.4, 139.5, 135.91 (d, J = 3.0 Hz), 134.48 (d, J = 10.3 Hz), 130.91 (d, J = 12.9 Hz), 128.7, 126.9, 117.5 (d, *J* = 89.6 Hz), 116.2, 21.4.

^{31}P NMR (162 MHz, $CDCl_3$): δ [ppm] = 23.08.

IR (ATR): ν (cm^{-1}) = 3958 (br), 2177 (br), 1586 (m), 1483 (m), 1437 (m), 1314 (m), 1246 (m), 1127 (s), 1107 (s), 1083 (s), 990 (m), 816 (w), 722 (vs), 689 (s).

Elemental Analysis: Anal. calcd. for $C_{36}H_{31}N_2O_2PS$: C, 73.70; H, 5.33; N, 4.77; S, 5.46. Found: C, 73.02; H, 5.45; N, 4.59; S, 5.66.

HRMS (ESI): calc. for $C_{12}H_{11}N_2O_2S^-$ [A^-]: 247.0547; found 247.0547; calc. for $C_{24}H_{20}P^+$ [C^+]: 339.1297; found: 339.1297.

m. p: 160-165 °C

Tetraphenylphosphonium pyridin-4-yl((4-(trifluoromethyl)phenyl)sulfonyl)amide (**6a**)

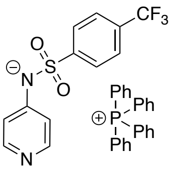

6a

Pyridinamide **6** (299 mg, 0.99 mmol, 1.0 eq) was added to a solution of NaOH (40.0 mg, 1.00 mmol, 1.1 eq) in H_2O (5.00 mL). Tetraphenylphosphonium bromide (415 mg, 0.99 mmol, 1.0 eq) was added and the reaction mixture was stirred at rt. overnight. DCM (5.00 mL) was added, and the two phases were separated. The aqueous phase was extracted with DCM (3 x 10 mL). The collected organic phase was dried over $MgSO_4$, filtered and the solvent was evaporated. The crude product was crystallized from DCM overlayered with toluene and heptane (0.50 mL) while stored in the fridge. The obtained crystals were dissolved in little DCM and the solvent was removed *in vacuum*, yielding a colorless foam that was dried under N_2 for two days. Ion Pair **6a** (537 mg, 0.84 mmol, 85%) was obtained as a colorless solid.

^{1}H NMR (400 MHz, $CDCl_3$): δ [ppm] = 8.06 – 8.01 (m, 1H), 7.92 – 7.83 (m, 3H), 7.73 (td, *J* = 7.9, 3.6 Hz, 4H), 7.61 – 7.49 (m, 5H), 6.81 (d, *J* = 5.5 Hz, 1H).

^{13}C{^{1}H} NMR (101 MHz, $CDCl_3$): δ [ppm] = 157.5, 150.3, 148.9,8 135.95 (d, *J* = 3.1 Hz), 134.45 (d, *J* = 10.3 Hz), 130.9 (d, *J* = 12.9 Hz), 127.3, 125.15 (q, *J* = 3.8 Hz), 124.1 (*virt*. q, *J* = 272.3 Hz, *C*F_3), 117.55 (d, *J* = 89.5 Hz), 166.4.

^{31}P NMR (162 MHz, $CDCl_3$): δ [ppm] = 23.09.

^{19}F NMR (376 MHz, $CDCl_3$): δ [ppm] = -62.61.

IR (ATR): ν (cm^{-1}) = 3060 (bs), 1587 (m), 1483 (m), 1438 (m), 1322 (vs), 1253 (w), 1128 (s), 1108 (s), 1060 (m), 992 (m), 970 (w), 757 (w), 723 (m), 690 (m).

Elemental Analysis: Anal. calcd. for $C_{36}H_{28}F_3N_2O_2PS$: C, 67.49; H, 4.41; N, 4.37; S, 5.00. Found: C, 64.34; H, 4.43; N, 4.09; S, 4.74.

HRMS (ESI): calc. for $C_{12}H_8F_3N_2O_2S^-$ [A^-]: 301.0264; found 301.0264; calc. for $C_{24}H_{20}P^+$ [C^+]: 339.1297; found: 339.1297.

m. p: 75-78 °C

2.3 Synthesis of Additives

Tetraphenylphosphonium tetrafluoroborate (**7a**)[2]

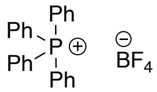

7a

Tetraphenylphosphonium bromide (1.05 g, 2.50 mmol, 1.0 eq) was dissolved in water and sodium tetrafluoroborate (276 mg, 2.50 mmol, 1.0 eq) was added in portions. The resulting cloudy suspension was stirred for 2 h at rt. DCM (10 mL) was added, and the two phases were separated. The aqueous phase was extracted with DCM (3 x 20 mL). The collected organic phase was dried over $MgSO_4$, filtered and the solvent was evaporated. The crude product was dissolved in DCM and precipitated by adding toluene. Product **6** (940 mg, 2.21 mmol, 88%) was obtained as colorless needles.

^{1}H NMR (400 MHz CD_2Cl_2): δ [ppm] = 7.98 – 7.87 (m, 4 H), 7.81 – 7.72 (m, 8 H), 7.66 – 7.56 (m, 8 H).

^{13}C{^{1}H} NMR (101 MHz, CD_2Cl_2): δ [ppm] = 36.2 (d, *J* = 3.3 Hz), 134.8 (d, *J* = 10.4 Hz), 131.0 (d, *J* = 13.0 Hz).

^{19}F NMR (377 MHz, CD_2Cl_2): δ [ppm] = -153.43.

^{31}P NMR (162 MHz, CD_2Cl_2): δ [ppm] = 23.12.

Elemental Analysis: Anal. calcd. for $C_{24}H_{20}BF_4P$: C, 67.64; H, 4.73. Found: C, 67.58; H, 4.62.

HRMS (ESI): calc. for BF_4^- [A^-]: 87.0035; found: 87.0034; calc. for $C_{24}H_{20}P^+$ [C^+]: 339.1297; found: 339.1292.

Triphenyl((triphenyl-λ5-phosphaneylidene)amino)phosphonium tetrafluoroborate (**7b**)

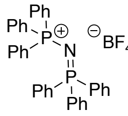

7b

Bis-(triphenylphosphoranyliden)-ammonium chloride (804 mg, 1.50 mmol, 1.0 eq) was suspended in H_2O and sodium tetrafluoroborate (154 mg, 1.40 mmol, 1.0 eq) was added in portions. The resulting cloudy suspension was stirred for 2 h at rt. DCM (10.0 mL) was added, and the two phases were separated. The aqueous phase was extracted with DCM (3 x 20 mL). The collected organic phase was dried over $MgSO_4$, filtered and the solvent was evaporated. The crude product was dissolved in DCM and the solvent was removed *in vacuum* until dryness. Product **7b** (804 mg, 1.29 mmol, 92%) was obtained as colorless platelet.

^{1}H NMR (400 MHz CD_2Cl_2): δ [ppm] = 7.69 – 7.61 (m, 6 H), 7.54 – 7.39 (m, 24 H).

^{13}C{^{1}H} NMR (101 MHz, CD_2Cl_2): δ [ppm] = 134.1 (t, *J* = 1.1 Hz), 132.8 – 132.4 (m), 130.1 – 129.6 (m), 127.4 (dd, *J* = 108.0, 2.0 Hz).

^{19}F NMR (377 MHz, CD_2Cl_2): δ [ppm] = -153.59.

^{31}P NMR (162 MHz, CD_2Cl_2): δ [ppm] = 21.06

IR (ATR): ν (cm^{-1}) = 3058 (w), 1588 (w), 1482 (m), 1437 (m), 1263 (m), 1184 (w), 1113 (m), 1052 (s), 995 (m), 955 (w), 798 (w), 761 (w), 747 (m), 723 (s), 691 (s).

Elemental Analysis: Anal. calcd. for $C_{36}H_{30}BF_4NP_2$: C, 69.14; H, 4.84; N, 2.24. Found: C, 65.07; H, 4.77; N, 1.96.

HRMS (ESI): calc. for BF_4^- [A^-]: 87.0035; found: 87.0034; calc. for $C_{36}H_{30}NP_2^+$ [C^+]: 538.1848; found: 538.1835.

m. p: 350-352 °C.

Tetrabutylammonium tetrafluoroborate (**7c**)

$^{\oplus}NBu_4$ $^{\ominus}BF_4$
7c

Tetrabutylammonium tetrafluoroborate was purchased from Sigma Aldrich (99% purity) and used without further purification after analysis.

^{1}H NMR (400 MHz CD_2Cl_2): δ [ppm] = 3.24 – 3.06 (m, 8 H), 1.70 – 1.52 (m, 8 H), 1.41 (h, *J* = 7.3 Hz, 8 H), 1.00 (t, *J* = 7.3 Hz, 12 H).

^{13}C{^{1}H} NMR (101 MHz, CD_2Cl_2): δ [ppm] = 59.0, 24.1, 20.0, 13.7.

^{19}F NMR (376 MHz, CD_2Cl_2): δ [ppm] = -151.98

Elemental Analysis: Anal. calcd. for $C_{16}H_{36}BF_4N$: C, 58.36; H, 11.02; N, 4.25. Found: C, 58.41; H, 10.81; N, 4.20.

HRMS (ESI): calc. for BF_4^- [A$^-$]: 87.0035; found: 87.0034; calc. for $C_{16}H_{36}N^+$ [C$^+$]: 242.2842; found: 242.2842.

Tetrabutylphosphonium tetrafluoroborate (**7d**)

$^{\oplus}PBu_4$ $^{\ominus}BF_4$
7d

Tetrabutylphosphonium hydroxide (1.91 g, 40% in H_2O, 1.77 mmol, 1.0 eq) was added to a solution of sodium tetrafluoroborate (303 mg, 2.77 mmol, 1.0 eq) in H_2O (10.0 mL). The resulting cloudy suspension was stirred for 2 h at rt. DCM (10.0 mL) was added, and the two phases were separated. The aqueous phase was extracted with DCM (3 x 20 mL). The collected organic phase was dried over $MgSO_4$, filtered and the solvent was evaporated. The crude product was dissolved in DCM and the solvent evaporated until dryness. Product **7d** (958 mg, 1.45 mmol, 52%) was further dried *in vacuum*, resulting in a colorless solid.

^{1}H NMR (400 MHz CD_2Cl_2): δ [ppm] = 2.22 – 2.01 (m, 8 H), 1.60 – 1.43 (m, 16 H), 1.05 – 0.92 (m, 12 H).

^{13}C{^{1}H} NMR (101 MHz, CD_2Cl_2): δ [ppm] = 24.3 (d, *J* = 15.4 Hz), 23.8 (d, *J* = 4.8 Hz), 18.9 (d, *J* = 47.7 Hz), 13.5.

^{19}F NMR (376 MHz, CD_2Cl_2): δ [ppm] = -151.65

^{31}P NMR (162 MHz, CD_2Cl_2): δ [ppm] = 33.10

IR (ATR): ν (cm^{-1}) = 2960 (m), 2934 (m), 2874 (w), 1467 (w), 1417 (w), 1382 (w), 1284 (w), 1234 (w), 1096 (m), 1049 (s), 1036 (s), 906 (w), 831 (w), 719 (w).

Elemental Analysis: Anal. calcd. for $C_{16}H_{36}BF_4P$: C, 55.50; H, 10.48. Found: C, 55.58; H, 10.54.

HRMS (ESI): calc. for BF_4^- [A$^-$]: 87.0035; found: 87.0034; calc. for $C_{16}H_{36}P^+$ [C$^+$]: 259.2549; found: 259.2548.

m. p: 95-96 °C

3. Conductometric Measurements

3.1 Sample Preparation and Data Acquirement

For the measurement an up to 0.02 M solution of the respective catalyst is prepared in a 10 mL volumetric flask and is given portion wise to the pure solvent in a measuring cell at 20 °C. Conductivity measurements were done using a WTW LF53 conductometer with a Pt electrode LTA 1/NS in MeCN and DCM. Calibration was done as described in ref [4]. Temperature control (20.0 ± 0.1 °C) was achieved by using a circulating bath cryostat. The conductivity is measured in volt [V] and depicted in "ME-REDLab Data Acquisition V1.1, developed by Dr. B. Kempf 2010, where the conductivity is plotted against the time. After adding a portion of ion pair stock solution, one must wait until the conductivity value reaches a stable plateau (min 30 sec up to 100 sec.) form which the average conductivity value is read off. In Excel this value [V] is then converted into [μS/cm] by multiplying it with the cell parameter of the used electrode (z = 160). The cell parameter was obtained by calibrating the conductometric set up with aqueous KCl solutions (0.007 M, 0.0145 M, and 0.0375 M; commercial conductivity standard solutions purchased from Alfa Aesar) referring to the conductivity of 1273 μS/cm for an 0.01 M solution of aq. KCl at 20 °C.[5]

3.2 Conductivity Data Analysis

The conductivity of a substance is defined as its ability to conduct electricity and depends on the number of charge carrier in solution. Therefore, it can be expressed as a molar quantity where κ is the measured conductivity, Λ_m is the molar conductivity and *c* is the measured concentration of electrolyte.

$$\kappa = \Lambda_\mathrm{m} \mathrm{c} \tag{S1}$$

The experimentally determined conductivity κ depends on the specific molar conductivity Λ_m of the measured salt and the ion concentration [X] as expressed in eq. S2.

$$\kappa = \Lambda_\mathrm{m}[\mathrm{X}] \tag{S2}$$

The molar conductivity Λ_m consists of the sum of all limited ionic conductivities λ_i of all ionic species *i* in solution according to eq. S3.

$$\Lambda_\mathrm{m} = \sum \lambda_i \tag{S3}$$

The value for the specific molar conductivity Λ_m for the combined ions is derived from the extrapolated linear regression line of the red marked conductivity values (first three data points) assuming that the respective measured salt is fully dissociated in this concentration area (see Figure S4). Therefore, the specific molar conductivity Λ_m can be divided into the limited ionic conductivity of the cation λ_cat and of the anion λ_an. The limited ionic conductivities are determined by applying Fuoss' theory that the molar conductivity λ of each ion is proportional to their volume.[6,7] By using the calculated volumes of each anion, cation, and ion pair based on the Van der Waals cavities employed in the SMD continuum solvation model at the SMD(DCM)/B3LYP-D3/6-31+G(d) level of theory the scaling factor δ_i for the conversion of Λ_m into λ_i of the respective anion **A** and cation **C** was obtained (see eq. S4).

$$\delta_\mathrm{A} = \frac{\mathrm{vol(A)}}{\mathrm{vol(IP)}} \tag{S4a}$$

$$\delta_\mathrm{C} = \frac{\mathrm{vol(C)}}{\mathrm{vol(IP)}} \tag{S4b}$$

The background conductivity (BG) included here is the conductivity of the used solvent (BG = 0.8 μS cm^{-1}).

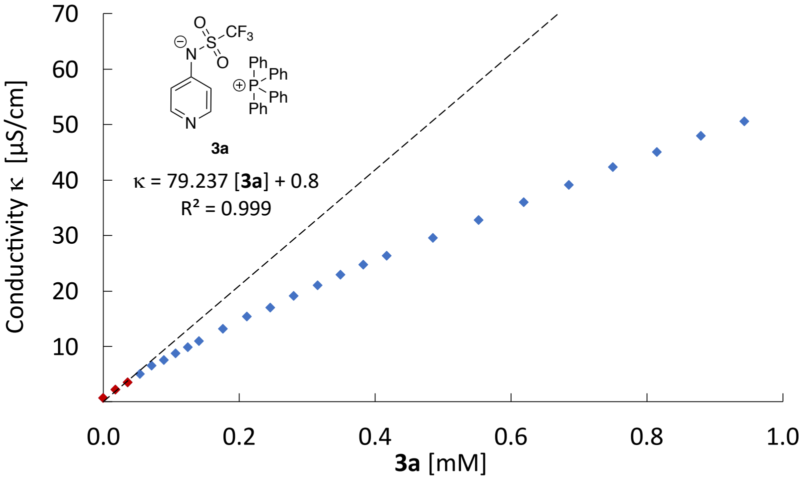

Figure S4. Concentration-dependent conductivity profile for salt **3a** in DCM with Λ_m = 79.2 S cm^2 mol^{-1} and solvent background BG = 0.8 µS cm^{-1}at 20 °C.

Determination of the respective association constants were achieved by numerical simulation utilizing the complex pathway simulation program, COPASI[8], for the analysis of the conductivity data.

Therefore, a biochemical model was opened, and the to be investigated association type was described as a reaction. The reaction was set to be reversible, which is also indicated by the equal sign "=" in the reaction equation. In the "Symbol Definition" section the starting compounds are listed with rate constant k_1 as well as the product with rate constant k_2, defining the association constant as $k_1/k_2 = K$.

For the optimization of the association constant K the reaction constant k_2 was set to be 1, while the rate constant k_1 was changed until the best fit between the simulated and experimental conductivity curve was found. The "root-mean-square-error" RMSE was used as a quality control measure.

In the section "Species" the individual concentration for all compounds were added. This was zero for the product(s) and the total salt concentration for both educts (e.g. for the anion **A** and for the cation **C**).

Next, in the section "Tasks" menu the "Steady-State" option was selected. By clicking the "Run" button at the bottom of the window, COPASI ran through a steady state analysis and then reported the concentration of each species of the biochemical model.

The obtained concentrations were then transferred to an excel sheet where the concentrations were converted into conductivity values according to eq. S5 with λ_i being the limited ionic conductivity of a charged species, δ_i the scaling factor of a charged species for the conversion of the specific molar conductivity Λ_m into the respective λ_i, and $[X]_i$ being the concentration of this respective charged species. The scaling factor $\delta_i = \lambda_i/\Lambda_m$ was introduced to simplify the conversion process in excel.

$$\kappa = \sum \lambda_i \times [X]_i \qquad \text{(S5a)}$$

$$\kappa = \sum \delta_i \times \Lambda_m \times [X]_i \qquad \text{(S5b)}$$

These simulated conductivity values were then compared to the experimental data. The conductivity background of the solvents was subtracted from the data points giving the conductivity of the pure salt and the RMSE was calculated as a quality control measure. The rate constant k_1 was optimized until the minimum of the RMSE was found.

This procedure was semi-automated by using a phyton script that automatically performs the "steady state" function for a given numbers of concentrations.[9]

The following association types were analyzed by employing numerical simulations:

3.3 Model I – Double 1:1 Ion Pair Association

The 1:1 association model included two anions **A** and two cation **C** combining into two charge-neutral non-conducting ion pairs **IP** is described in Scheme S1.

$$2 * \mathrm{A} \quad + \; 2 * \mathrm{C} \; \overset{K_{\mathrm{IP}}}{\rightleftharpoons} \; 2 * \mathrm{IP}$$

Scheme S1. Equation for numerical simulations of double 1:1 ion association for pyridinamide ion pairs.

The 1:1 ion pair association constant is defined as $k_1/k_2 = K_{IP}$ with $k_2 = 1$ being a fixed value. The limited ionic conductivities for the anion and cation were calculated according to eq. S4, while it was assumed that the neutral ion pair does not contribute towards the overall conductivity (λ_{IP} = 0.0 S cm^2 mol^{-1}). The results of this model for all ion pairs systems are summarized below in Table S4.

Table S4. Double ion association constants K_{IP} (M^{-2}) with the specific molar conductivities Λ_m (S cm^2 mol^{-1}) and limited ionic conductivities λ_i (S cm^2 mol^{-1}) and the scaling factor δ_i for pyridinamide ion pairs.

Ion Pair	Λ_m (S cm^2 mol^{-1})	δ_A	δ_C	λ_A (S cm^2 mol^{-1})	λ_C (S cm^2 mol^{-1})	K_{IP} (M^{-2})	RMSE
**3d**	56.0	0.40	0.60	22.4	33.6	7.35 x 10^6	0.329
**3c**	57.9	0.41	0.59	23.7	34.2	5.00 x 10^6	0.261
**3b**	80.3	0.28	0.72	22.4	57.8	2.96 x 10^5	0.318
**3a**	79.2	0.37	0.63	29.3	49.9	6.86 x 10^5	0.304
**5a**	73.2	0.42	0.58	30.7	42.5	6.92 x 10^5	0.228
**4a**	73.6	0.44	0.56	32.4	41.2	8.79 x 10^5	0.274
**6a**	74.2	0.45	0.55	33.4	40.8	6.89 x 10^5	0.320

While this model fits the conductivity data reasonably well, it does not concur with the DOSY NMR data. Instead of observing a convergence of the diffusion coefficients D_i (m^2 s^{-1}) and hence the corresponding ion volumes, the measurements show a clear deviation between both ions. The resulting DOSY volumes for exemplary **3a** were plotted against the used concentrations for both cation and anion (see Figure S5, experimental data see SI, Chapter 4 of ref [2]) and compared to simulated ion volumes calculated according to eq. S6 and based on the concentration obtained by numerical simulation of the 1:1 ion pair association model I.

$$\mathrm{vol_{cat}} = \left(\frac{[\mathrm{C}]}{[\mathrm{IP}]_{\mathrm{tot}}}\right) \times 362 + \left(\frac{[\mathrm{IP}]}{[\mathrm{IP}]_{\mathrm{tot}}}\right) \times 570 \qquad \text{(S6a)}$$

$$\mathrm{vol_{an}} = \left(\frac{[\mathrm{A}]}{[\mathrm{IP}]_{\mathrm{tot}}}\right) \times 215 + \left(\frac{[\mathrm{IP}]}{[\mathrm{IP}]_{\mathrm{tot}}}\right) \times 570 \qquad \text{(S6b)}$$

For the lowest concentrations the measured volumes are close to the calculated ones for the free ions (cation: 362 Å^3 and anion: 215 Å^3). For concentrations higher than 0.01 mM, a clear increase of the volumes for the anion **3** and the cation **a** can be observed with a large off-set between the anion and cation volumes. If the association follows an 1:1 ion pair association the volumes of anion and cation should converge and approach 570 Å^3 for a monomeric ion pair **3a**. Instead with increasing salt concentration the volume of the cation even exceeds the calculated volume for the 1:1 monomeric ion pair **3a** (641 Å^3 versus 570 Å^3 at 1.0 mM). On the other hand, the simulated volume of the cation based on the concentrations of the conductivity evaluation lie far below the calculated volume the 1:1 monomeric ion pair **3a** (435 Å^3 versus 570 Å^3 at 1.0 mM).

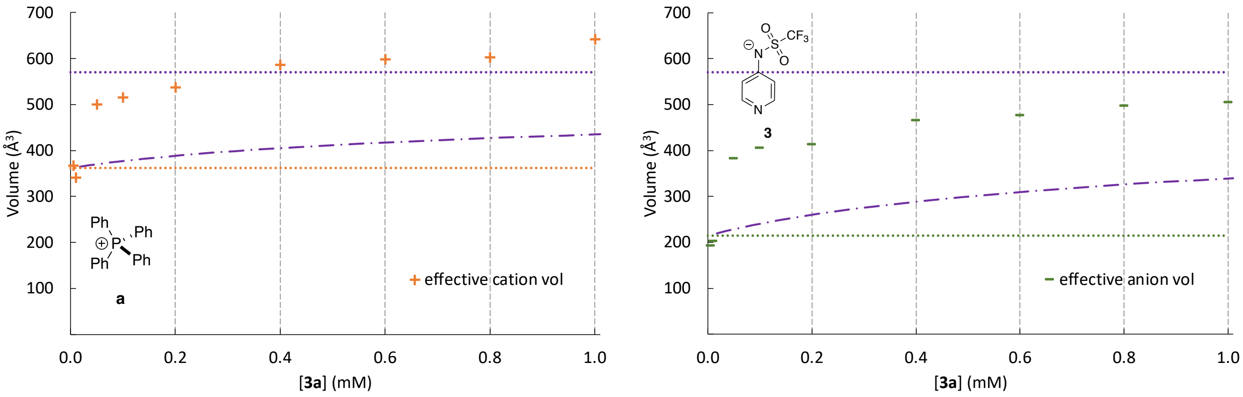

Figure S5. Concentration-dependent volumes of cations **a** (orange "+" symbols) and anions **3** (green "–" symbols) of salt **3a** in CD_2Cl_2 as calculated from DOSY experiments (with data from ref [2]) with the respective free ion volumes (dotted line; cation in orange; anion in green) and the calculated trend for 1:1 ion association for each ion volume (purple dotted line).

All these observations suggested the involvement of an additional charged species.

3.4 Model IIa – Cationic Sandwich Association

Based on the finding of DOSY NMR measurements further association types were investigated. One was a cationic sandwich association consisting of four ions, two anions **A** and two cations **C** associating into a formally positive charged sandwich complex **CAC** which leaves one free anion **A** (see Scheme S2).

$$2 * \mathrm{A} \; + \; 2 * \mathrm{C} \; \underset{}{\overset{K_{\mathrm{CAC}}}{\rightleftharpoons}} \; \mathrm{A} \; + \; \mathrm{CAC}$$

Scheme S2. General cationic sandwich association equilibrium for pyridinamide ion pairs.

The cationic sandwich association constant is defined as $k_1/k_2 = K_{\mathrm{CAC}}$ with $k_2 = 1$ being a fixed value. The limited ionic conductivities for the anion and cation were again calculated according to eq. S4. However, there was no established way to determine the limited ionic conductivity λ_{CAC} for the cation sandwich **CAC**. Therefore, instead of assigning all sandwich cations a fixed value, it was decided to treat λ_{CAC} as another variable. This required a slight modification of the optimization process since instead of one variable, the association constant K, there were two variables to be optimized, the sandwich association constant K_{CAC} (M^{-2}) and λ_{CAC} (S cm^2 mol^{-1}).

To simplify the process, the scaling factor δ_{CAC} was optimized rather than λ_{CAC} for the sandwich cation. Since the sandwich complex is formally positively charged, it is recommended to start the optimization process with $\delta_{\mathrm{CAC}} = \delta_{\mathrm{C}} = 0.63$ (exemplary for ion pair **3a**). The aim was to find a global minimum based on the sandwich association constant K_{CAC} with the RMSE being the quality control measure (see Table S5).

Table S5. List of cationic sandwich association constants K_{CAC} (M^{-2}) and scaling factor δ_{CAC} of the optimization process to find the global minimum for both parameters for pyridinamide ion pair **3a** with its specific molar conductivity Λ_m (S cm^2 mol^{-1}) and the limited ionic conductivities λ_i (S cm^2 mol^{-1}) for anion **3** and cation **a**.

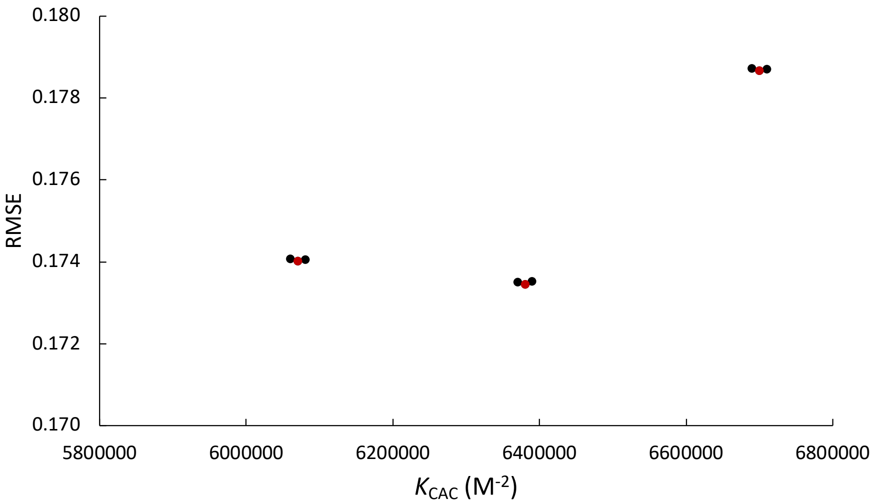

Ion Pair	Λ_m (S cm^2 mol^{-1})	δ_A/δ_C	δ_{CAC}	K_{CAC} (M^{-2})	RMSE
**3a**	79.2	37/63	0.66	6.06×10^6	0.1741
				6.07×10^6	0.1740
				6.08×10^6	0.1741
			0.67	6.37×10^6	0.1735
				**6.38×10^6**	**0.1734**
				6.39×10^6	0.1735
			0.68	6.69×10^6	0.1787
				6.70×10^6	0.1786
				6.71×10^6	0.1787

The obtained sandwich association constants for all ion pair systems are summarized in Table S6.

Table S6. Cationic sandwich association constants K_{CAC} (M^{-2}) with the specific molar conductivities Λ_m (S cm^2 mol^{-1}) and the scaling factor δ_i of all charged species for pyridinamide ion pairs.

Ion Pair	Λ_m (S cm^2 mol^{-1})	δ_A /δ_C /δ_{CAC} ratio	K_{CAC} (M^{-2})	RMSE
**3d**	56.0	40/60/15	1.07×10^7	0.094
**3c**	57.9	41/59/22	1.01×10^7	0.100
**3b**	80.3	28/72/91	4.65×10^6	0.105
**3a**	79.2	37/63/67	6.38×10^6	0.173
**5a**	73.2	42/58/57	5.15×10^6	0.130
**4a**	73.6	44/56/54	6.50×10^6	0.122
**6a**	74.2	45/55/60	6.75×10^6	0.161

Comparing the RMSE value obtained in the 1:1 ion pair association and the cationic sandwich association, a better fit for the cationic sandwich association model can be observed. As a secondary quality control measure, the ion volumes based on DOSY NMR were compared to ion volumes calculated based on the numerical simulation data. Therefore, the anion and cation volumes were calculated according to eq. S7.

$$\mathrm{vol_{cat}} = \left(\frac{[\mathrm{C}]}{[\mathrm{IP}]_{\mathrm{tot}}}\right) \times 362 + \left(\frac{2 \times [\mathrm{CAC}]}{[\mathrm{IP}]_{\mathrm{tot}}}\right) \times 925 \qquad \text{(S7a)}$$

$$\mathrm{vol_{an}} = \left(\frac{[\mathrm{A}]}{[\mathrm{IP}]_{\mathrm{tot}}}\right) \times 215 + \left(\frac{[\mathrm{CAC}]}{[\mathrm{IP}]_{\mathrm{tot}}}\right) \times 925 \quad \text{(S7b)}$$

Figure S6 shows the data and model comparison for ion pair **3a** as an example.

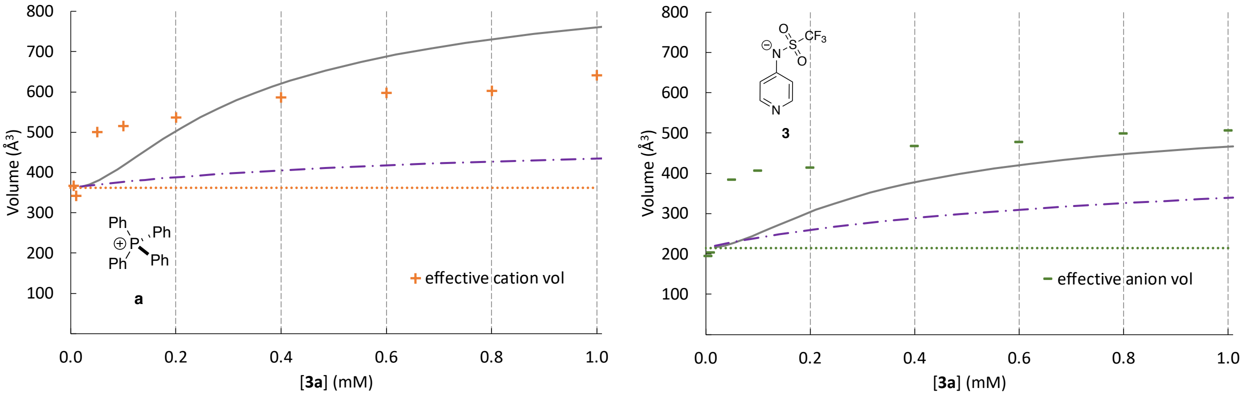

Figure S6. Concentration-dependent volumes of cations **a** (orange "+" symbols) and anions **3** (green "–" symbols) of salt **3a** in CD_2Cl_2 as calculated from DOSY experiments (with data from ref [2]) with the respective free ion volumes (dotted line; cation in orange; anion in green), the calculated trend for 1:1 ion association for each ion volume (purple dotted line), and for the cationic sandwich model (grey line).

In this case the simulated volume curves for the cation and anion (orange and green lines, respectively) are closer to the experimental data than the 1:1 ion pair association model. First indications are that cationic sandwich association gives indeed an excellent fit for both the conductivity as well as the DOSY NMR data.

3.5 Model IIb – Anionic Sandwich Association

Further comprehensive DOSY studies of all ion pair systems showed that the more nucleophilic ion pairs **4-6a** showed results that indicate an anionic sandwich association rather than a cationic sandwich association (see SI of ref [2]). Adjustment of the cationic sandwich association to incorporate an anion sandwich consists of four ions, two anions **A** and two cations **C** associating into a formally negative charged sandwich complex **ACA** which leaves one free cation **C** (see Scheme S3).

$$2 * \mathrm{A} \; + \; 2 * \mathrm{C} \;\xrightleftharpoons{K_{\mathrm{ACA}}}\; \mathrm{C} \; + \; \mathrm{ACA}$$

Scheme S3. General anionic sandwich association equilibrium for pyridinamide ion pairs.

The anion sandwich association constant is defined as $k_1/k_2 = K_{\mathrm{ACA}}$ with $k_2 = 1$ being a fixed value. The limited ionic conductivities for the anion and cation were again calculated according to eq. S4. The limited ionic conductivity λ_{ACA} of the anionic sandwich complex was treated as another variable. To find the global minimum for both the limited ionic conductivity λ_{ACA} and the anionic sandwich association constant K_{ACA} the same fitting process as described above for the cationic sandwich association model was followed. Since the sandwich complex is formally negatively charged, it is recommended to start the optimization process with $\delta_{\mathrm{ACA}} = \delta_{\mathrm{A}}$. The results are summarized in Table S7.

Table S7. Anionic sandwich association constants K_{ACA} (M^{-2}) with the specific molar conductivities Λ_m (S cm^2 mol^{-1}) and the scaling factor δ_i of all charged species for pyridinamide ion pairs.

Ion Pair	Λ_m (S cm^2 mol^{-1})	δ_A /δ_C /δ_{ACA} ratio	K_{ACA} (M^{-2})	RMSE
**3d**	56.0	40/60/–	–	–
**3c**	57.9	41/59/4	1.01×10^7	0.100
**3b**	80.3	28/72/47	4.65×10^6	0.105
**3a**	79.2	37/63/41	6.38×10^6	0.173

**5a**	73.2	42/58/41	5.15 x 10^6	0.130
**4a**	73.6	44/56/42	6.50 x 10^6	0.122
**6a**	74.2	45/55/50	6.75 x 10^6	0.161

Numerical simulation of both sandwich association types gave identical association constants and RMSE values. Thus, based on conductivity data alone, those two association types cannot be distinguished. The sole exception is ion pair **3d**. Here the global minimum could not be found in the anionic sandwich association without allowing negative values for the limited ionic conductivity λ_{ACA}. This indicates either that for this ion pair, an anionic sandwich association is not feasible or that model II ran into a numerical boundary. In both cases, no optimized anionic sandwich association constant K_{ACA} could be found for ion pair **3d**.

For the comparison of the experimental and simulated ion volumes, the simulated volumes were calculated according to eq. S8 for the anionic sandwich association model.

$$\mathrm{vol_{cat}} = \left(\frac{[\mathrm{C}]}{[\mathrm{IP}]_{\mathrm{tot}}}\right) \times 362 + \left(\frac{[\mathrm{ACA}]}{[\mathrm{IP}]_{\mathrm{tot}}}\right) \times 782 \quad \text{(S8a)}$$

$$\mathrm{vol_{an}} = \left(\frac{[\mathrm{A}]}{[\mathrm{IP}]_{\mathrm{tot}}}\right) \times 215 + \left(\frac{2 \times [\mathrm{ACA}]}{[\mathrm{IP}]_{\mathrm{tot}}}\right) \times 782 \quad \text{(S8b)}$$

The comparison showed that the anionic sandwich association model does not offer a better overlap between the experimentally obtained and simulated ion volumes. Figure S7 shows the simulated ion volumes for both the cationic and the anionic sandwich association model exemplary for pyridinamide ion pair **3a**.

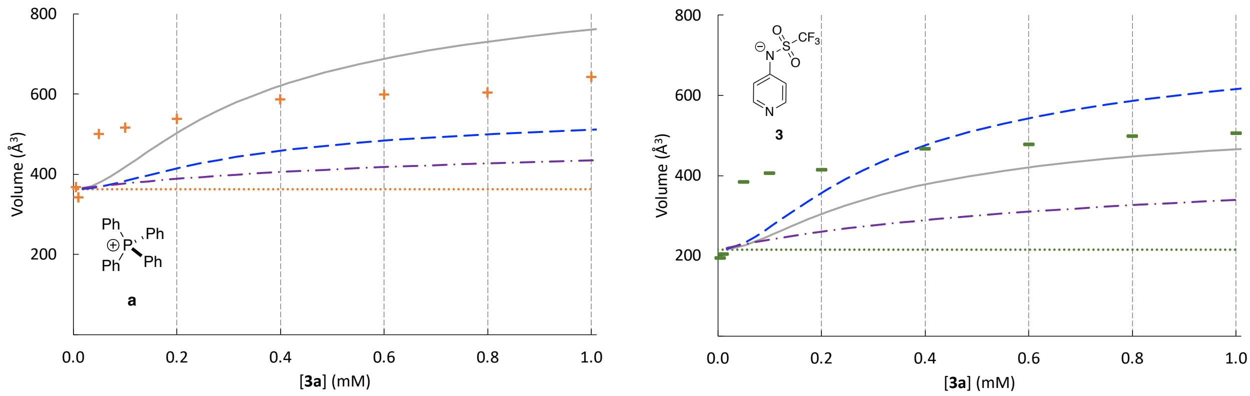

Figure S7. Concentration-dependent volumes of cations **a** (orange "+" symbols) and anions **3** (green "–" symbols) of salt **3a** in CD_2Cl_2 as calculated from DOSY experiments (with data from ref [2]) with the respective free ion volumes (dotted line; cation in orange; anion in green), the calculated trend for 1:1 ion association for each ion volume (purple dotted line), and for the cationic (grey line) and anionic (blue dashed line) sandwich model.

While the simulated cation volumes based on the cationic sandwich association model are larger than the experimental values, the simulated cation volumes based on the anionic sandwich association are smaller than them. For the simulated anion volumes, the opposite holds true. For pyridinamide ion pair **3a** the cationic sandwich model is preferred since the increase in anion volumes should be obvious in the DOSY measurements, with the anion volumes surpassing the cation volumes as it can be seen for pyridinamide ion pair **4a** (for further details see SI of ref [2]).

3.6 Model III – Mixed Sandwich Association

Since the separate analysis of the conductivity data for both cationic and anionic sandwich association still revealed significant deviations in comparing the experimental and simulated ion volumes, the combination of both sandwich association types in the *mixed sandwich association model* was the next step.

Therefore, both sandwich association constants, K_{CAC} and K_{ACA}, were assigned a *scaling factor* α and β, respectively (see Scheme S4).

$$2 * A + 2 * C \xrightleftharpoons{\alpha \times K_{CAC}} A + CAC$$

$$2 * A + 2 * C \xrightleftharpoons{\beta \times K_{ACA}} C + ACA$$

Scheme S4. Association equations for mixed sandwich association model.

To find the right ratio between cationic and anionic sandwich association one contribution factor would be set to a fixed value, while the other one would be treated as a variable and be optimized by using numerical simulation to find the minimum of the RMSE value. The optimization of the contribution factor was limited to two relevant decimals.

Cation and anion volumes obtained by DOSY NMR measurements at 1.0 mM salt concentration were used as reference to determine the optimal ratio for the factors, α and β. The simulated ion volumes were calculated according to eq. S9.

$$\mathrm{vol}_{\mathrm{cat}} = \frac{[\mathrm{C}]}{[\mathrm{IP}]_{\mathrm{tot}}} \times 362 + \left(\frac{2 \times [\mathrm{CAC}]}{[\mathrm{IP}]_{\mathrm{tot}}}\right) \times 925 + \left(\frac{[\mathrm{ACA}]}{[\mathrm{IP}]_{\mathrm{tot}}}\right) \times 782 \quad \text{(S9a)}$$

$$\mathrm{vol}_{\mathrm{an}} = \frac{[\mathrm{A}]}{[\mathrm{IP}]_{\mathrm{tot}}} \times 215 + \left(\frac{[\mathrm{CAC}]}{[\mathrm{IP}]_{\mathrm{tot}}}\right) \times 925 + \left(\frac{2 \times [\mathrm{ACA}]}{[\mathrm{IP}]_{\mathrm{tot}}}\right) \times 782 \quad \text{(S9b)}$$

The percentual residual between experimental and simulated ion volumes determined the optimal ratio for the factors, α and β in the mixed sandwich association. For more details see SI of ref [2].

Examining the simulated ion volumes based on Model II for the cationic and anionic sandwich association against the newly introduced mixed model showed a significantly improved overlap for the experimental data than any other association model (see Figure S8, exemplary for ion pair **3a**).

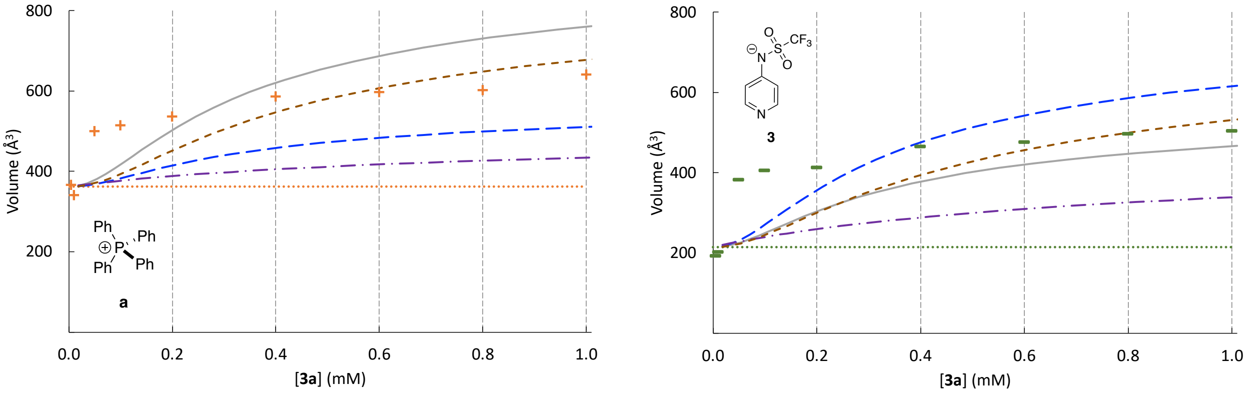

Figure S8. Concentration-dependent volumes of cations **a** (orange "+" symbols) and anions **3** (green "–" symbols) of salt **3a** in CD_2Cl_2 as calculated from DOSY experiments (with data from ref [2]) with the respective free ion volumes (dotted line; cation in orange; anion in green), the calculated trend for 1:1 ion association for each ion volume (purple dotted line), for the cationic (grey line) and anionic (blue dashed line) sandwich model and the mixed sandwich association model (brown short-dash line).

The mixed model gave the best results when checking the experimental and simulated ion volumes against each other. The results of model 3 for all ion pairs are summarized in Table S8.

Table S8. Final cationic and anionic sandwich association constants K_{CAC} (M^{-2}) and K_{ACA} (M^{-2}) with the specific molar conductivities Λ_m (S cm^2 mol^{-1}) and the scaling factors δ_i of all charged species with their respective scaling factors α and β for pyridinamide ion pairs.

Ion Pair	Λ_m (S cm^2 mol^{-1})	$\delta_A/\delta_C/\delta_{CAC}/\delta_{ACA}$ (%)	$\alpha \times K_{CAC}$ [M^{-2}]	$\beta \times K_{ACA}$ [M^{-2}]	α/β (%)	RMSE
**3d**	56.0	40/60/15/–	–	–	–	–
**3c**	57.9	41/59/22/4	3.33×10^6	2.32×10^6	33/23	0.61

**3b**	80.3	28/72/91/47	4.65 x 10^6	0.00	100/0	0.11
**3a**	79.2	37/63/67/41	2.81 x 10^6	1.34 x 10^6	44/21	0.43
**5a**	73.2	42/58/57/41	5.67 x 10^5	3.45 x 10^6	11/67	0.29
**4a**	73.6	44/56/54/42	7.80 x 10^5	3.97 x 10^6	12/61	0.35
**6a**	74.2	45/55/60/50	1.08 x 10^6	3.51 x 10^6	16/52	0.38

Notable is the result of the analysis of pyridinamide ion pair **3b** in the mixed model. For the **3b** the best result was achieved for the purely cationic sandwich association without any participation of the anionic sandwich association. However, the percentual residual for both cation and anion volume are rather large with +25% for the anion volume and +14% for the cation volume (see Figure S9A) compared to the results of the other ion pairs, e.g. ± 6.0% for ion volume in pyridinamide ion pair **3a** (see Figure S9B).

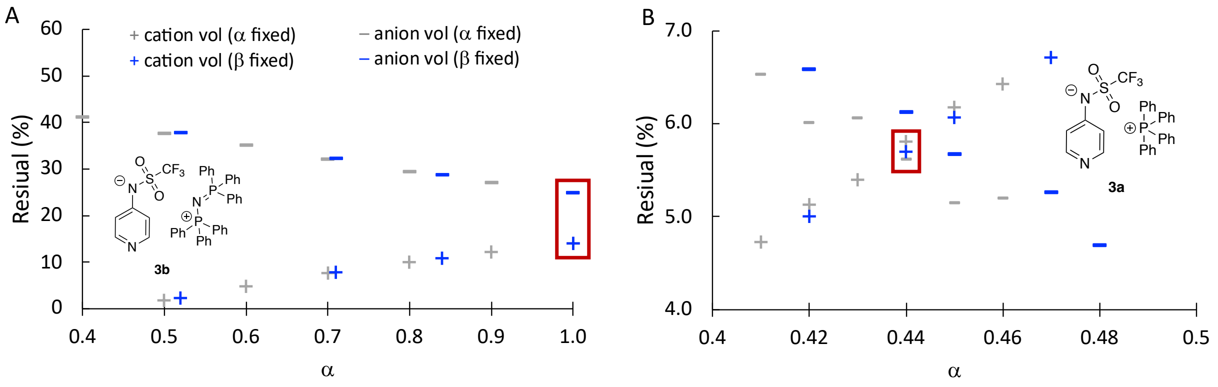

Figure S9. A) Percentual residue for calculated cation (blue (for β fixed) and grey (for α fixed) "+") and anion (blue (for β fixed) and grey (for α fixed) "–") volumes in mixed sandwich association model for pyridinamide ion pair **3b**. B) Percentual residue for calculated cation (blue (for β fixed) and grey (for α fixed) "+") and anion (blue (for β fixed) and grey (for α fixed) "–") volumes in mixed sandwich association model for pyridinamide ion pair **3a**.

This might be an indication for other ionic species or ionic effects taking place in solution for **3b** that are not yet accounted for in the current conductivity model. Moving forward, pyridinamide ion pairs **3b** as well as **3d** will be analyzed with the cationic sandwich association model.

3.7 Workflow summary

For the determination of ion pairing constant K_{IP} and sandwich association constant K_{CAC} for ionic compounds like **3a** based on conductivity measurements in DCM, the following steps were carried out:

Determination of Ion Pairing constant K_{IP} for salt **3a**

a) Measure conductivity for **3a**.
b) Determine the molar conductivity Λ_m by linear extrapolation of the first three data points.
c) Use eq. S3 or the respective biochemical model (for details see Scheme S1 or S2) concentrations for all compound involved in the model, copy the concentrations into excel to convert them into conductivity values by assigning both ion 50% contribution towards Λ_m.
d) Compare sum of theoretical conductivity with experimental values (without solvent background conductivity).
e) Use the RMSE value as a quality control measure.
f) Adjust k_1 in $K_{CAC} = k_1/k_2$ until the minimum RMSE is found, to obtain K_{IP}.

Determination of sandwich association constant K_{CAC} for salt **3a**

g) Measure conductivity for **3a**.
h) Determine the molar conductivity Λ_m by linear extrapolation of the first three data points.
i) Set the limited molar ionic conductivity for the anion λ_A and cation λ_C to the calculated values for the scaling factor δ_i based on eq. S8. The sum of $\delta_A+\delta_C$ cannot exceed 1.
j) Treat $\delta_{CAC} = \lambda_{CAC}/\Lambda_m$(**3a**) as a second variable. Select at starting value e.g. $\delta_{CAC} = 0.67$ and start the optimization process for K_{CAC}.
k) Use the respective sandwich association model (Model 3) to obtain concentrations for all species involved, copy them into Excel to convert them into conductivity values using eq. S5.
l) Compare sum of theoretical conductivity with experimental values (without solvent background conductivity).
m) Use the RMSE values as a quality control measure.
n) Adjust k_1 in $K_{CAC} = k_1/k_2$ until minimum RMSE is found, to obtain the final K_{CAC}.
o) Adjust the molar ionic conductivity for the sandwich ion λ_{CAC} to find the percentual distribution that fits the conductivity data the best. For each new percentual distribution, repeat step k)-n) until the global minimum RMSE is found.

For analysis of the additive **7a-d** the same steps are followed, only step q deviates since the determination of the specific molar conductivity Λ_m of BF_4^- is done slightly differently:

p) Measure conductivity for salt **7a-d**.
q) Determine the molar conductivity Λ_m by linear extrapolation of the first three data points.
r) Follow step i) – o) as described above to obtain K_{CAC} for additive **7a-d**.

Determination of composition of cationic and anionic sandwich association for salt **3a** (Model 3)

After determination of the cationic sandwich association constant K_{CAC} and the anionic sandwich association K_{ACA} the mix of both association types that fits the experimental data best is ascertained.

s) Assign each association constant a *scaling factor*: $K_{CAC} \times \alpha$ and $K_{ACA} \times \beta$.
t) Set α to a fixed value starting at 1.0 and going down in steps of 0.1. Optimize β to achieve the smallest RMSE value for the conductivity data using numerical simulations. Limit each factor to two relevant decimals.
u) Repeat step s) vice versa for factor β.
v) Determine the composition of cationic and anionic sandwich association in reference to the measured DOSY volume at 1.0 mM by calculating the percentual residual between experimental and simulated ion volumes.
w) Take further optimization steps until finding the α/β ratio with the smallest percentual residual for both cation and anion volume.

3.8 Conductivity data in MeCN

Concentration dependent conductivity data of **3b** in MeCN at 20 °C

[3b]/M	Conductivity κ (μS/cm)
0.00	0.80
1.68×10^{-5}	3.36
3.36×10^{-5}	5.28
5.04×10^{-5}	7.20
6.71×10^{-5}	9.76
8.38×10^{-5}	11.7
1.00×10^{-4}	13.6
1.17×10^{-4}	16.0
1.34×10^{-4}	17.9
1.50×10^{-4}	20.2
1.67×10^{-4}	22.1
1.83×10^{-4}	24.2
2.00×10^{-4}	26.4
2.33×10^{-4}	30.6
2.65×10^{-4}	34.7
2.98×10^{-4}	38.7
3.30×10^{-4}	42.9
3.63×10^{-4}	46.7
3.95×10^{-4}	50.7
4.59×10^{-4}	58.7
5.22×10^{-4}	66.4
5.85×10^{-4}	74.2
6.48×10^{-4}	81.9
7.10×10^{-4}	89.6
7.71×10^{-4}	96.8
8.32×10^{-4}	104
8.93×10^{-4}	111
9.53×10^{-4}	118
1.01×10^{-3}	125
1.07×10^{-3}	132
1.13×10^{-3}	139
1.25×10^{-3}	153
1.36×10^{-3}	166
1.48×10^{-3}	179
1.59×10^{-3}	192
1.70×10^{-3}	205
1.80×10^{-3}	217
1.91×10^{-3}	229
2.02×10^{-3}	240
2.12×10^{-3}	252

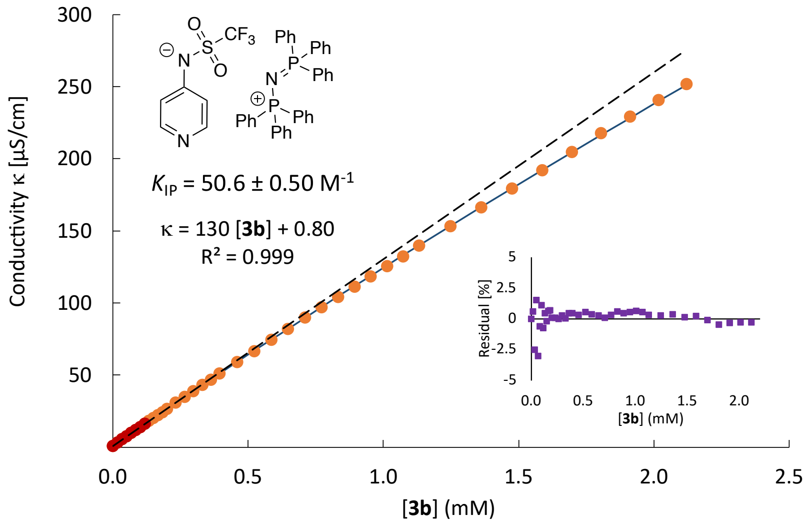

Concentration dependent conductivity data of **3c** in MeCN at 20 °C

[3c]/M	Conductivity κ (μS/cm)
0.00	0.80
1.99×10^{-5}	3.36
3.97×10^{-5}	6.24
5.94×10^{-5}	8.80
7.92×10^{-5}	11.4
9.89×10^{-5}	14.0
1.19×10^{-4}	16.4
1.38×10^{-4}	19.1
1.58×10^{-4}	21.8
1.77×10^{-4}	24.2
1.97×10^{-4}	26.9
2.36×10^{-4}	32.0
2.74×10^{-4}	37.0
3.13×10^{-4}	42.0
3.51×10^{-4}	47.0
3.90×10^{-4}	51.8
4.66×10^{-4}	61.4
5.41×10^{-4}	71.1
6.16×10^{-4}	80.6
6.90×10^{-4}	89.8
8.37×10^{-4}	108
9.82×10^{-4}	126
1.12×10^{-3}	143
1.27×10^{-3}	159
1.40×10^{-3}	177
1.54×10^{-3}	192
1.67×10^{-3}	208
1.81×10^{-3}	223
1.94×10^{-3}	238
2.07×10^{-3}	253
2.19×10^{-3}	268
2.32×10^{-3}	282
2.44×10^{-3}	296

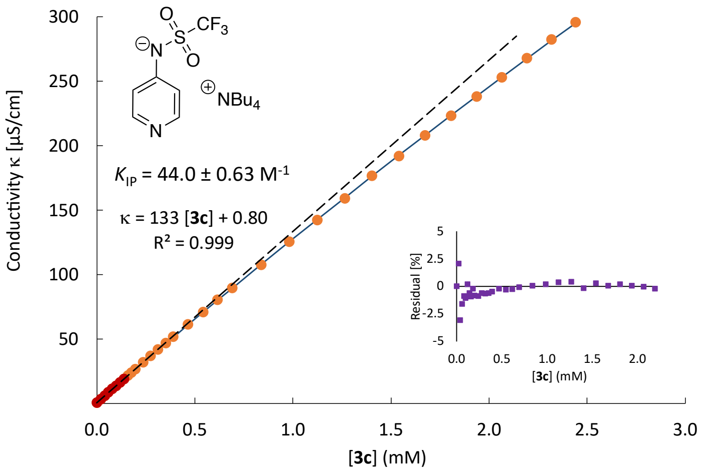

Concentration dependent conductivity data of **3d** in MeCN at 20 °C

[3d]/M	Conductivity κ (μS/cm)
0.00	0.80
2.01×10^{-5}	3.20
4.01×10^{-5}	5.92
6.01×10^{-5}	8.48
8.00×10^{-5}	11.0
9.99×10^{-5}	13.8
1.20×10^{-4}	16.3
1.40×10^{-4}	18.7
1.59×10^{-4}	21.3
1.79×10^{-4}	23.7
1.99×10^{-4}	26.2
2.18×10^{-4}	28.8
2.38×10^{-4}	31.2
2.58×10^{-4}	33.6
2.77×10^{-4}	36.2
2.97×10^{-4}	38.4
3.16×10^{-4}	41.1
3.56×10^{-4}	46.1
3.94×10^{-4}	50.6
4.32×10^{-4}	55.4
4.71×10^{-4}	60.3
5.09×10^{-4}	64.8
5.47×10^{-4}	69.6
6.23×10^{-4}	78.4
6.98×10^{-4}	87.2
7.72×10^{-4}	96.3
8.46×10^{-4}	105
9.20×10^{-4}	114
9.93×10^{-4}	123
1.06×10^{-3}	131
1.14×10^{-3}	140
1.21×10^{-3}	149
1.28×10^{-3}	157
1.42×10^{-3}	173
1.56×10^{-3}	189
1.69×10^{-3}	205
1.83×10^{-3}	220
1.96×10^{-3}	234
2.09×10^{-3}	249
2.22×10^{-3}	263
2.34×10^{-3}	277
2.47×10^{-3}	291

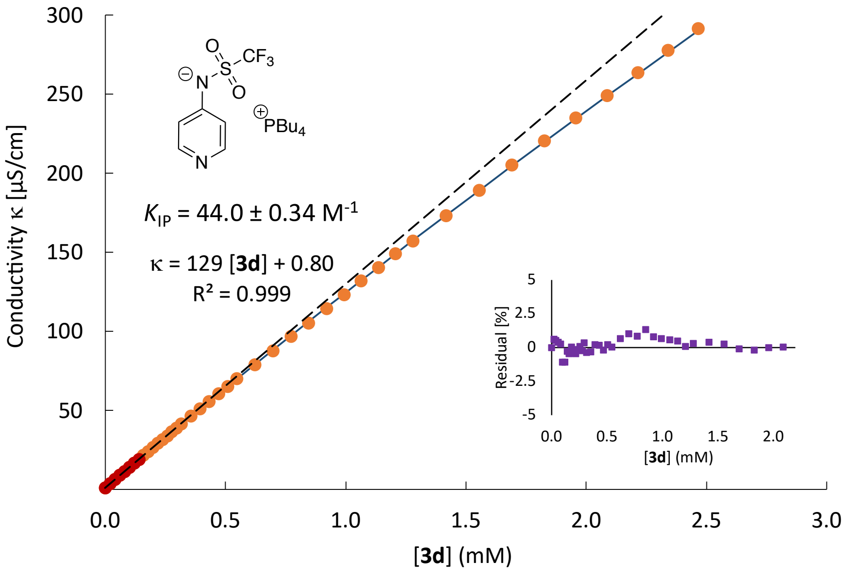

Concentration dependent conductivity data of **5a** in MeCN at 20 °C

[5a]/M	Conductivity κ (μS/cm)
0.00	0.80
1.98 x 10^{-5}	2.96
3.95 x 10^{-5}	5.36
5.92 x 10^{-5}	7.52
7.89 x 10^{-5}	9.68
9.85 x 10^{-5}	11.8
1.18 x 10^{-4}	14.2
1.38 x 10^{-4}	16.3
1.57 x 10^{-4}	18.4
1.77 x 10^{-4}	20.7
1.96 x 10^{-4}	22.9
2.35 x 10^{-4}	27.2
2.73 x 10^{-4}	31.4
3.12 x 10^{-4}	35.8
3.50 x 10^{-4}	40.0
3.88 x 10^{-4}	44.2
4.64 x 10^{-4}	53.1
5.39 x 10^{-4}	60.6
6.14 x 10^{-4}	68.8
6.88 x 10^{-4}	76.9
7.61 x 10^{-4}	84.7
8.34 x 10^{-4}	92.3
9.78 x 10^{-4}	107
1.12 x 10^{-3}	121
1.26 x 10^{-3}	136
1.40 x 10^{-3}	150
1.53 x 10^{-3}	164
1.67 x 10^{-3}	177
1.80 x 10^{-3}	189

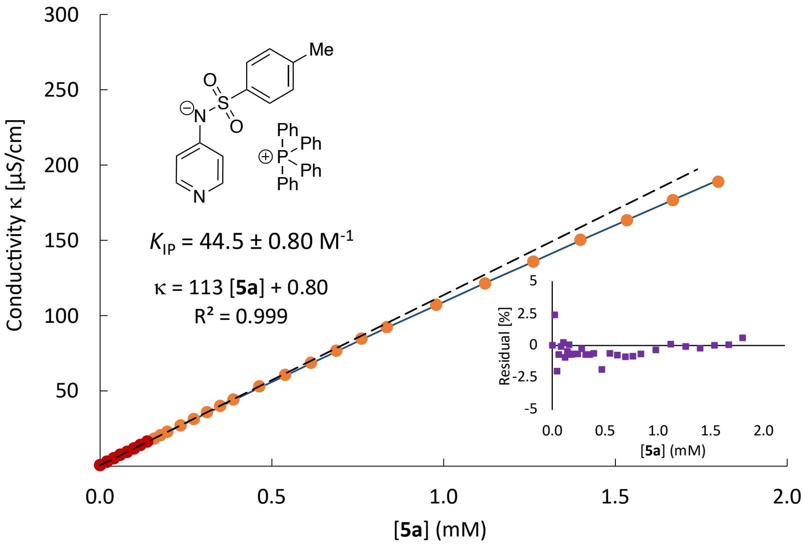

Concentration dependent conductivity data of **6a** in MeCN at 20 °C

[6a]/M	Conductivity κ (μS/cm)
0.00	8.00×10^{-1}
1.88×10^{-5}	2.72×10^{0}
3.75×10^{-5}	5.28×10^{0}
5.62×10^{-5}	7.20×10^{0}
7.48×10^{-5}	9.28×10^{0}
9.35×10^{-5}	1.17×10^{1}
1.12×10^{-4}	1.38×10^{1}
1.31×10^{-4}	1.58×10^{1}
1.49×10^{-4}	1.78×10^{1}
1.68×10^{-4}	1.97×10^{1}
1.86×10^{-4}	2.19×10^{1}
2.04×10^{-4}	2.38×10^{1}
2.23×10^{-4}	2.61×10^{1}
2.59×10^{-4}	3.02×10^{1}
2.96×10^{-4}	3.42×10^{1}
3.32×10^{-4}	3.82×10^{1}
3.68×10^{-4}	4.24×10^{1}
4.04×10^{-4}	4.62×10^{1}
4.40×10^{-4}	5.04×10^{1}
5.12×10^{-4}	5.80×10^{1}
5.82×10^{-4}	6.56×10^{1}
6.53×10^{-4}	7.33×10^{1}
7.22×10^{-4}	8.08×10^{1}
7.92×10^{-4}	8.86×10^{1}
8.60×10^{-4}	9.56×10^{1}
9.28×10^{-4}	1.03×10^{2}
9.96×10^{-4}	1.10×10^{2}
1.06×10^{-3}	1.17×10^{2}
1.13×10^{-3}	1.24×10^{2}
1.20×10^{-3}	1.30×10^{2}
1.26×10^{-3}	1.37×10^{2}
1.39×10^{-3}	1.51×10^{2}
1.52×10^{-3}	1.64×10^{2}
1.65×10^{-3}	1.77×10^{2}
1.77×10^{-3}	1.89×10^{2}
1.89×10^{-3}	2.02×10^{2}
2.01×10^{-3}	2.14×10^{2}
2.13×10^{-3}	2.26×10^{2}
2.25×10^{-3}	2.37×10^{2}
2.36×10^{-3}	2.48×10^{2}

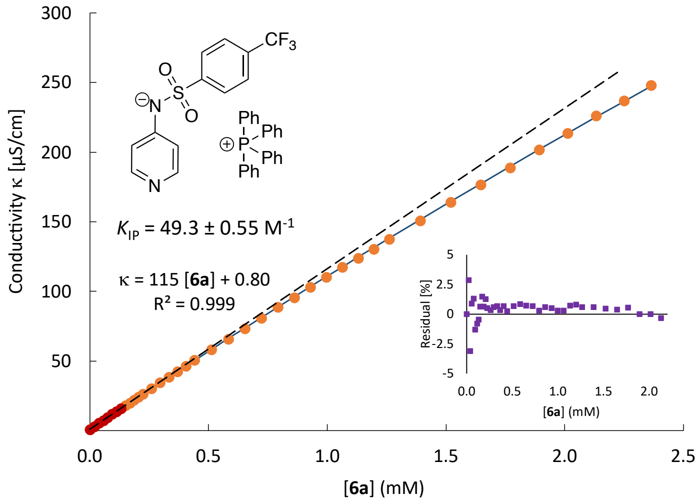

3.9 Pyridinamide ion Pairs – Conductivity data in DCM

Concentration dependent conductivity data of **3a** in DCM at 20 °C

[3a]/M	Conductivity κ (μS/cm)
0.00	0.80
1.78×10^{-5}	2.24
3.55×10^{-5}	3.60
5.40×10^{-5}	5.12
7.09×10^{-5}	6.48
8.86×10^{-5}	7.44
1.06×10^{-4}	8.80
1.24×10^{-4}	9.92
1.41×10^{-4}	11.0
1.76×10^{-4}	13.2
2.11×10^{-4}	15.4
2.46×10^{-4}	17.0
2.80×10^{-4}	19.2
3.15×10^{-4}	21.1
3.49×10^{-4}	23.0
3.83×10^{-4}	24.8
4.17×10^{-4}	26.4
4.85×10^{-4}	29.6
5.52×10^{-4}	32.8
6.19×10^{-4}	36.0
6.85×10^{-4}	39.2
7.50×10^{-4}	42.4
8.15×10^{-4}	45.1
8.80×10^{-4}	48.0
9.44×10^{-4}	50.6
1.01×10^{-3}	53.1
1.07×10^{-3}	56.2
1.13×10^{-3}	58.4
1.20×10^{-3}	60.8
1.32×10^{-3}	66.4
1.44×10^{-3}	71.2
1.56×10^{-3}	76.0
1.68×10^{-3}	80.3
1.79×10^{-3}	84.0
2.02×10^{-3}	92.8
2.24×10^{-3}	101
2.51×10^{-3}	111
2.77×10^{-3}	120
3.26×10^{-3}	138
3.72×10^{-3}	153
4.15×10^{-3}	167
4.56×10^{-3}	181
4.94×10^{-3}	194

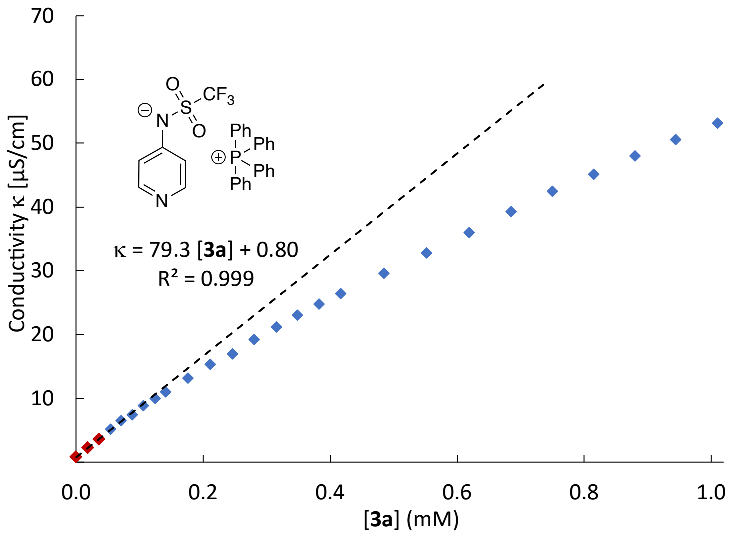

Concentration dependent conductivity data of **3b** in DCM at 20 °C

[3b]/M	Conductivity κ (μS/cm)
0.00	0.80
1.79 x 10^{-5}	2.24
3.58 x 10^{-5}	3.68
5.37 x 10^{-5}	5.12
7.16 x 10^{-5}	6.48
8.94 x 10^{-5}	7.84
1.08 x 10^{-4}	9.12
1.25 x 10^{-4}	10.4
1.43 x 10^{-4}	11.7
1.60 x 10^{-4}	12.8
1.78 x 10^{-4}	14.0
1.95 x 10^{-4}	15.1
2.13 x 10^{-4}	16.3
2.48 x 10^{-4}	18.6
2.83 x 10^{-4}	20.6
3.18 x 10^{-4}	22.7
3.52 x 10^{-4}	24.8
3.87 x 10^{-4}	26.9
4.21 x 10^{-4}	28.8
4.55 x 10^{-4}	30.7
4.89 x 10^{-4}	32.6
5.57 x 10^{-4}	36.5
6.24 x 10^{-4}	39.8
6.91 x 10^{-4}	43.2
7.57 x 10^{-4}	46.9
8.23 x 10^{-4}	50.1
8.88 x 10^{-4}	53.6
9.52 x 10^{-4}	57.1
1.02 x 10^{-3}	60.2
1.08 x 10^{-3}	63.4
1.14 x 10^{-3}	66.4
1.27 x 10^{-3}	72.8
1.39 x 10^{-3}	78.4
1.51 x 10^{-3}	84.5
1.63 x 10^{-3}	90.1
1.75 x 10^{-3}	95.4
1.87 x 10^{-3}	100
2.09 x 10^{-3}	111
2.32 x 10^{-3}	121
2.58 x 10^{-3}	132
2.84 x 10^{-3}	143
3.33 x 10^{-3}	162
3.80 x 10^{-3}	182
4.23 x 10^{-3}	201
4.64 x 10^{-3}	217

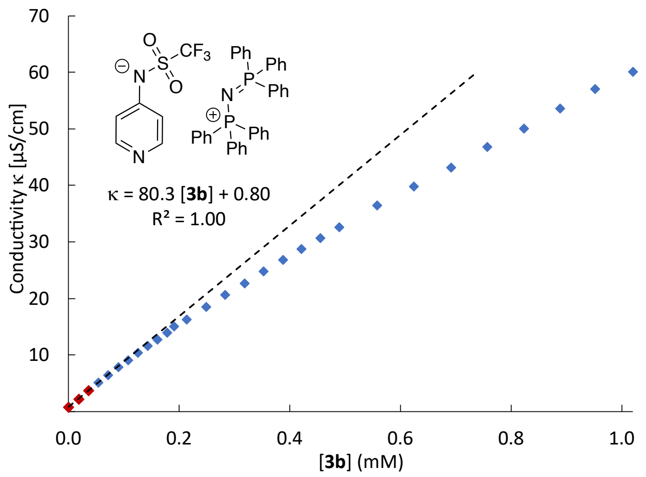

Concentration dependent conductivity data of **3c** in DCM at 20 °C

[3c]/M	Conductivity κ (μS/cm)
0.00	0.80
2.00×10^{-5}	2.00
3.99×10^{-5}	3.09
5.98×10^{-5}	4.24
7.97×10^{-5}	5.28
9.95×10^{-5}	6.24
1.19×10^{-4}	6.96
1.39×10^{-4}	7.68
1.59×10^{-4}	8.40
1.98×10^{-4}	9.76
2.37×10^{-4}	10.9
2.76×10^{-4}	12.1
3.15×10^{-4}	13.3
3.54×10^{-4}	14.3
3.92×10^{-4}	15.4
4.30×10^{-4}	16.3
4.69×10^{-4}	17.4
5.45×10^{-4}	19.1
6.20×10^{-4}	20.8
6.95×10^{-4}	22.6
7.69×10^{-4}	23.9
8.43×10^{-4}	25.5
9.16×10^{-4}	27.1
9.88×10^{-4}	28.5
1.06×10^{-3}	29.8
1.20×10^{-3}	32.3
1.34×10^{-3}	35.0
1.48×10^{-3}	37.4
1.62×10^{-3}	39.6
1.75×10^{-3}	41.9
1.88×10^{-3}	43.8
2.14×10^{-3}	48.2
2.39×10^{-3}	52.0
2.70×10^{-3}	56.3
2.99×10^{-3}	60.8
3.55×10^{-3}	68.1
4.08×10^{-3}	75.5
4.57×10^{-3}	82.7
5.03×10^{-3}	88.8

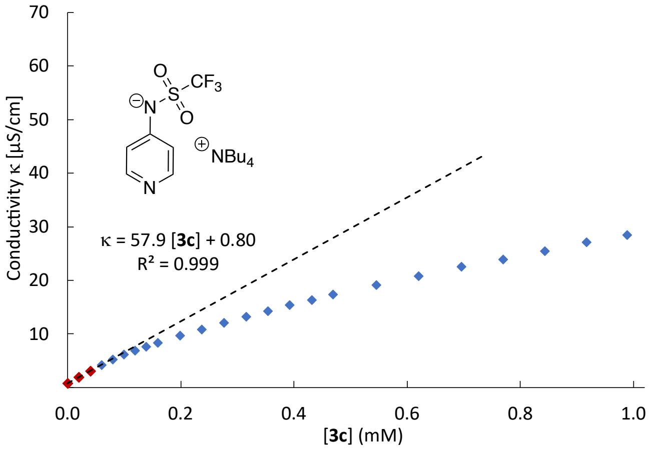

Concentration dependent conductivity data of **3d** in DCM at 20 °C

[3d]/M	Conductivity κ (μS/cm)
0.00	0.80
2.00 x 10^{-5}	1.92
4.00 x 10^{-5}	3.04
5.99 x 10^{-5}	4.16
7.98 x 10^{-5}	4.96
9.97 x 10^{-5}	5.92
1.20 x 10^{-4}	6.72
1.39 x 10^{-4}	7.36
1.59 x 10^{-4}	8.00
1.79 x 10^{-4}	8.80
1.98 x 10^{-4}	9.28
2.38 x 10^{-4}	10.4
2.77 x 10^{-4}	11.5
3.16 x 10^{-4}	12.3
3.54 x 10^{-4}	13.3
3.93 x 10^{-4}	14.4
4.70 x 10^{-4}	16.0
5.46 x 10^{-4}	17.6
6.21 x 10^{-4}	18.9
6.96 x 10^{-4}	20.5
7.71 x 10^{-4}	22.1
8.44 x 10^{-4}	23.2
9.18 x 10^{-4}	24.5
9.90 x 10^{-4}	25.8
1.06 x 10^{-3}	26.9
1.13 x 10^{-3}	28.0
1.28 x 10^{-3}	30.2
1.42 x 10^{-3}	32.3
1.55 x 10^{-3}	34.6
1.69 x 10^{-3}	35.7
1.82 x 10^{-3}	38.4
1.95 x 10^{-3}	40.5
2.08 x 10^{-3}	42.1
2.27 x 10^{-3}	44.5
2.46 x 10^{-3}	47.2
2.70 x 10^{-3}	50.1
2.94 x 10^{-3}	52.8
3.23 x 10^{-3}	56.5
3.50 x 10^{-3}	59.8
4.03 x 10^{-3}	65.6
4.53 x 10^{-3}	71.5
4.99 x 10^{-3}	77.3

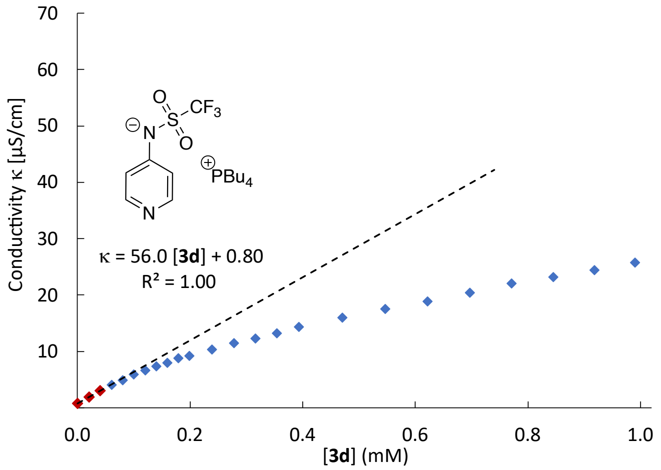

Concentration dependent conductivity data of **4a** in DCM at 20 °C

[4a]/M	Conductivity κ (μS/cm)
0.00	0.80
1.96×10^{-5}	2.24
3.91×10^{-5}	3.68
5.86×10^{-5}	5.04
7.81×10^{-5}	6.40
9.75×10^{-5}	7.52
1.17×10^{-4}	8.96
1.36×10^{-4}	9.92
1.56×10^{-4}	11.0
1.76×10^{-4}	12.2
1.94×10^{-4}	13.1
2.13×10^{-4}	14.2
2.32×10^{-4}	15.3
2.71×10^{-4}	17.0
3.09×10^{-4}	19.0
3.47×10^{-4}	20.8
3.84×10^{-4}	22.6
4.22×10^{-4}	24.2
4.59×10^{-4}	25.9
4.97×10^{-4}	27.5
5.34×10^{-4}	29.2
5.71×10^{-4}	30.7
6.08×10^{-4}	32.5
6.81×10^{-4}	35.4
7.54×10^{-4}	38.2
8.26×10^{-4}	41.1
8.98×10^{-4}	43.7
9.69×10^{-4}	46.6
1.04×10^{-3}	49.1
1.11×10^{-3}	51.5
1.18×10^{-3}	54.6
1.25×10^{-3}	57.0
1.32×10^{-3}	59.5
1.45×10^{-3}	64.3
1.59×10^{-3}	69.0
1.72×10^{-3}	73.3
1.85×10^{-3}	77.6
1.97×10^{-3}	82.1
2.10×10^{-3}	86.6
2.35×10^{-3}	94.2
2.59×10^{-3}	102
2.88×10^{-3}	111
3.16×10^{-3}	120
3.69×10^{-3}	136
4.19×10^{-3}	151
4.66×10^{-3}	165
5.10×10^{-3}	178

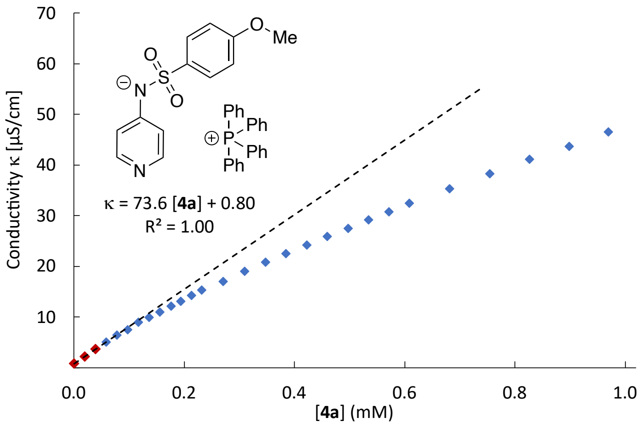

Concentration dependent conductivity data of **5a** in DCM at 20 °C

[5a]/M	Conductivity κ (μS/cm)
0.00	0.80
1.97×10^{-5}	2.24
3.94×10^{-5}	3.68
5.90×10^{-5}	5.12
7.85×10^{-5}	6.56
9.81×10^{-5}	7.92
1.18×10^{-4}	9.04
1.37×10^{-4}	10.1
1.56×10^{-4}	11.2
1.95×10^{-4}	13.4
2.34×10^{-4}	15.5
2.72×10^{-4}	17.4
3.10×10^{-4}	19.4
3.49×10^{-4}	21.3
3.87×10^{-4}	23.1
4.24×10^{-4}	24.8
4.62×10^{-4}	26.6
5.37×10^{-4}	29.9
6.11×10^{-4}	33.1
6.85×10^{-4}	36.3
7.58×10^{-4}	39.3
8.31×10^{-4}	42.1
9.03×10^{-4}	45.0
9.75×10^{-4}	47.8
1.05×10^{-3}	50.4
1.12×10^{-3}	53.0
1.19×10^{-3}	55.7
1.32×10^{-3}	60.6
1.46×10^{-3}	65.4
1.59×10^{-3}	70.3
1.73×10^{-3}	74.9
1.86×10^{-3}	79.2
1.99×10^{-3}	83.5
2.24×10^{-3}	91.8
2.48×10^{-3}	100
2.78×10^{-3}	109
3.06×10^{-3}	118
3.61×10^{-3}	135
4.12×10^{-3}	150
4.60×10^{-3}	164
5.05×10^{-3}	177

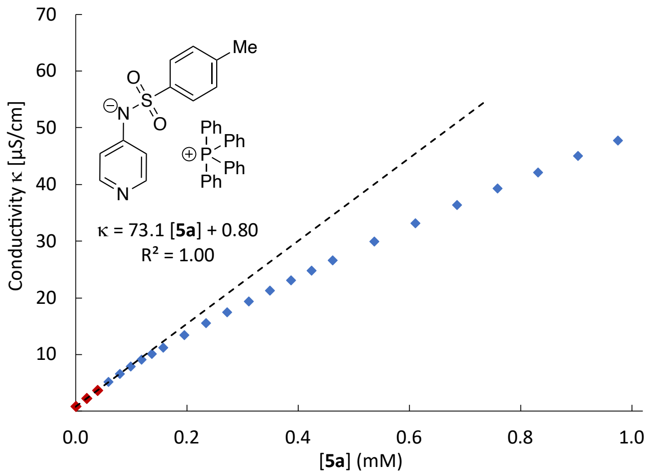

Concentration dependent conductivity data of **6a** in DCM at 20 °C

[6a]/M	Conductivity κ (μS/cm)
0.00	0.80
1.94×10^{-5}	2.24
3.88×10^{-5}	3.68
5.81×10^{-5}	5.12
7.74×10^{-5}	6.40
9.67×10^{-5}	7.60
1.16×10^{-4}	8.88
1.35×10^{-4}	10.0
1.54×10^{-4}	11.1
1.73×10^{-4}	12.2
1.92×10^{-4}	13.3
2.11×10^{-4}	14.2
2.30×10^{-4}	15.4
2.68×10^{-4}	17.3
3.06×10^{-4}	19.2
3.44×10^{-4}	21.2
3.81×10^{-4}	23.2
4.18×10^{-4}	24.6
4.56×10^{-4}	26.4
4.93×10^{-4}	28.3
5.29×10^{-4}	30.0
5.66×10^{-4}	31.5
6.03×10^{-4}	33.2
6.75×10^{-4}	36.3
7.69×10^{-4}	40.2
8.19×10^{-4}	42.4
8.90×10^{-4}	45.3
9.61×10^{-4}	48.2
1.03×10^{-3}	50.9
1.10×10^{-3}	53.6
1.17×10^{-3}	56.3
1.24×10^{-3}	58.7
1.31×10^{-3}	61.4
1.44×10^{-3}	66.4
1.57×10^{-3}	71.4
1.70×10^{-3}	76.0
1.83×10^{-3}	80.6
1.96×10^{-3}	85.0
2.14×10^{-3}	91.5
2.33×10^{-3}	97.6
2.56×10^{-3}	106
2.85×10^{-3}	115
3.13×10^{-3}	124
3.66×10^{-3}	141
4.16×10^{-3}	157

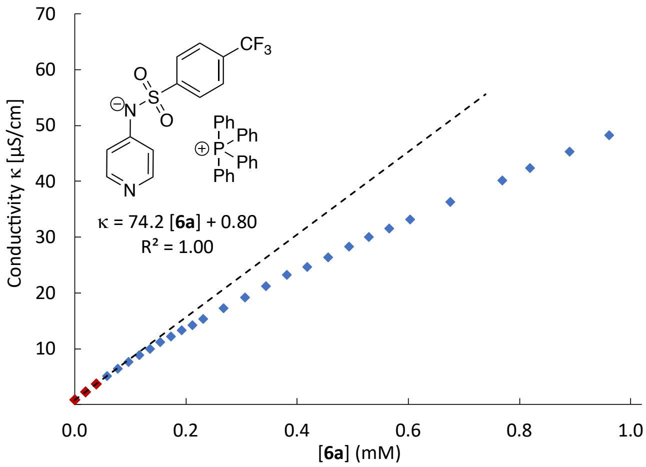

3.10 Additive Salts – Conductivity data in DCM

Concentration dependent conductivity data of **7a** in DCM at 20 °C

[**7a**]/M	Conductivity κ (μS/cm)
0.00	0.80
1.93×10^{-5}	2.64
3.86×10^{-5}	4.48
5.79×10^{-5}	6.24
7.71×10^{-5}	7.84
9.63×10^{-5}	9.36
1.15×10^{-4}	10.6
1.35×10^{-4}	12.3
1.54×10^{-4}	13.8
1.92×10^{-4}	16.4
2.30×10^{-4}	19.0
2.67×10^{-4}	21.4
3.05×10^{-4}	23.9
3.42×10^{-4}	26.1
3.80×10^{-4}	28.4
4.17×10^{-4}	30.5
4.54×10^{-4}	32.6
5.27×10^{-4}	36.6
6.00×10^{-4}	40.6
6.73×10^{-4}	44.1
7.45×10^{-4}	47.9
8.16×10^{-4}	51.4
8.87×10^{-4}	55.0
9.57×10^{-4}	58.2
1.03×10^{-3}	61.7
1.10×10^{-3}	64.6
1.16×10^{-3}	67.9
1.23×10^{-3}	71.1
1.30×10^{-3}	74.1
1.43×10^{-3}	79.9
1.57×10^{-3}	85.8
1.70×10^{-3}	91.3
1.82×10^{-3}	96.6
1.95×10^{-3}	102
2.20×10^{-3}	112
2.44×10^{-3}	122
2.73×10^{-3}	133
3.01×10^{-3}	145
3.54×10^{-3}	165
4.04×10^{-3}	184
4.51×10^{-3}	201

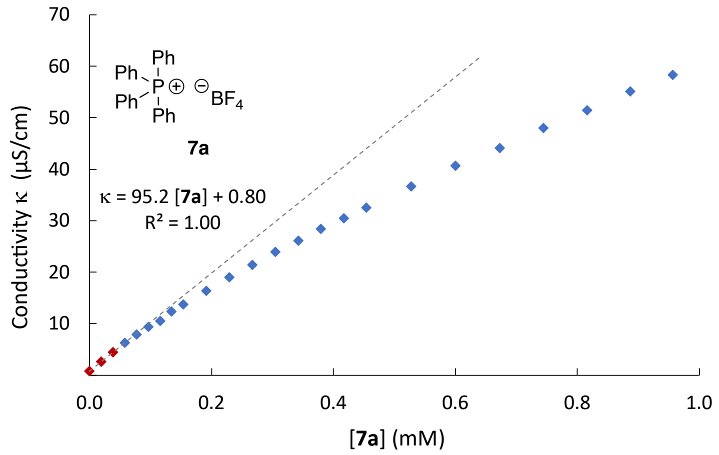

Concentration dependent conductivity data of **7b** in DCM at 20 °C

[**7b**]/M	Conductivity κ (μS/cm)
0.00	0.80
1.93×10^{-5}	2.56
3.85×10^{-5}	4.32
5.77×10^{-5}	6.08
7.69×10^{-5}	7.68
9.61×10^{-5}	9.36
1.15×10^{-4}	10.6
1.34×10^{-4}	12.2
1.53×10^{-4}	13.8
1.91×10^{-4}	16.4
2.29×10^{-4}	19.0
2.67×10^{-4}	21.8
3.04×10^{-4}	24.2
3.41×10^{-4}	26.6
3.79×10^{-4}	29.3
4.16×10^{-4}	31.4
4.52×10^{-4}	33.8
5.26×10^{-4}	38.1
5.99×10^{-4}	42.6
6.71×10^{-4}	46.7
7.43×10^{-4}	50.6
8.14×10^{-4}	54.9
8.84×10^{-4}	58.8
9.54×10^{-4}	62.6
1.02×10^{-3}	66.6
1.09×10^{-3}	70.2
1.16×10^{-3}	73.8
1.23×10^{-3}	77.2
1.30×10^{-3}	80.8
1.43×10^{-3}	87.7
1.56×10^{-3}	94.2
1.69×10^{-3}	101
1.82×10^{-3}	107
1.94×10^{-3}	113
2.19×10^{-3}	125
2.43×10^{-3}	136
2.72×10^{-3}	150
3.00×10^{-3}	163
3.53×10^{-3}	187
4.03×10^{-3}	210
4.50×10^{-3}	231
4.94×10^{-3}	250

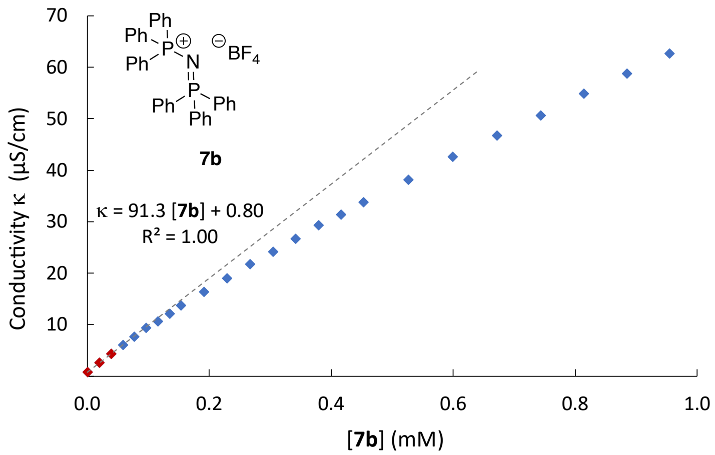

Concentration dependent conductivity data of **7c** in DCM at 20 °C

[7c]/M	Conductivity κ (µS/cm)
0.00	0.80
2.02×10^{-5}	2.24
4.03×10^{-5}	3.52
6.04×10^{-5}	4.32
8.05×10^{-5}	5.28
1.01×10^{-4}	6.24
1.21×10^{-4}	6.72
1.40×10^{-4}	7.36
1.60×10^{-4}	8.08
2.00×10^{-4}	9.20
2.40×10^{-4}	10.4
2.79×10^{-4}	11.2
3.18×10^{-4}	12.3
3.57×10^{-4}	13.1
3.96×10^{-4}	14.2
4.74×10^{-4}	16.0
5.50×10^{-4}	17.3
6.27×10^{-4}	18.9
7.02×10^{-4}	20.2
7.77×10^{-4}	21.8
8.52×10^{-4}	23.1
9.25×10^{-4}	24.3
9.99×10^{-4}	25.6
1.07×10^{-3}	26.9
1.14×10^{-3}	28.0
1.22×10^{-3}	29.2
1.29×10^{-3}	30.2
1.43×10^{-3}	32.3
1.57×10^{-3}	34.4
1.70×10^{-3}	36.5
1.84×10^{-3}	38.2
1.97×10^{-3}	40.2
2.23×10^{-3}	43.7
2.48×10^{-3}	46.9
2.79×10^{-3}	50.6
3.08×10^{-3}	54.4
3.64×10^{-3}	61.1
4.17×10^{-3}	67.1
4.66×10^{-3}	73.0
5.13×10^{-3}	78.2

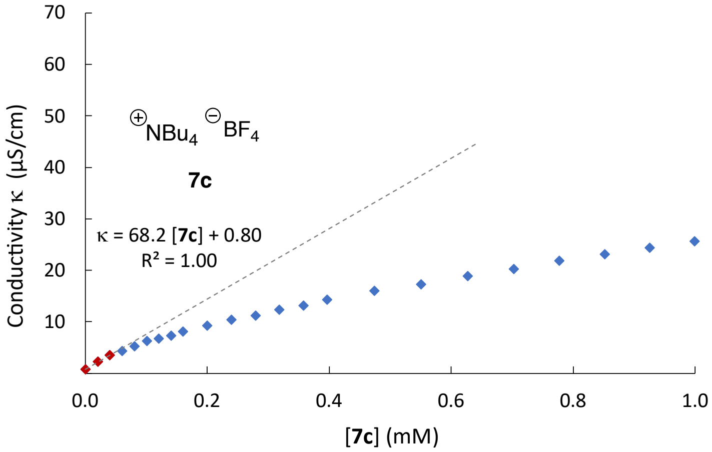

Concentration dependent conductivity data of **7d** in DCM at 20 °C

**[7d]**/M	Conductivity κ (μS/cm)
0.00	0.80
2.03×10^{-5}	2.08
4.06×10^{-5}	3.20
6.08×10^{-5}	4.08
8.10×10^{-5}	4.80
1.01×10^{-4}	5.44
1.21×10^{-4}	6.08
1.61×10^{-4}	7.12
2.01×10^{-4}	8.16
2.41×10^{-4}	9.04
2.81×10^{-4}	9.92
3.20×10^{-4}	10.6
3.60×10^{-4}	11.4
3.99×10^{-4}	12.2
4.77×10^{-4}	13.7
5.54×10^{-4}	15.0
6.31×10^{-4}	16.3
7.07×10^{-4}	17.4
7.82×10^{-4}	18.4
8.57×10^{-4}	19.7
9.32×10^{-4}	20.7
1.01×10^{-3}	21.8
1.08×10^{-3}	22.8
1.15×10^{-3}	23.7
1.29×10^{-3}	25.4
1.44×10^{-3}	27.4
1.58×10^{-3}	29.1
1.71×10^{-3}	30.6
1.85×10^{-3}	32.3
1.98×10^{-3}	33.8
2.24×10^{-3}	36.5
2.50×10^{-3}	39.2
2.81×10^{-3}	42.6
3.10×10^{-3}	45.4
3.67×10^{-3}	50.9
4.20×10^{-3}	56.0
4.69×10^{-3}	60.6
5.16×10^{-3}	65.0

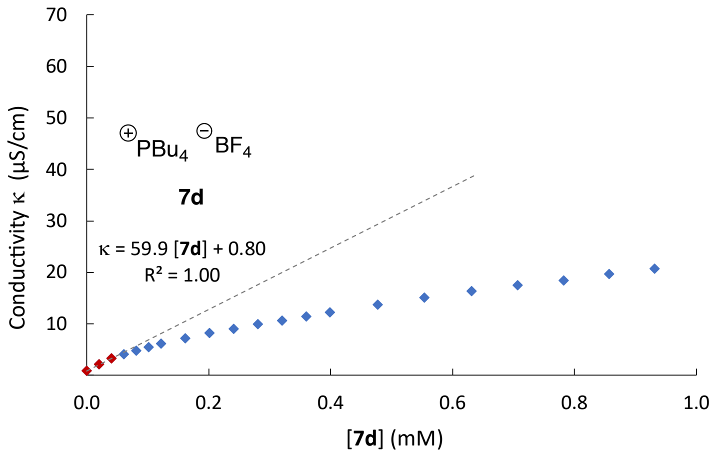

4. DOSY NMR Spectroscopy

Diffusion ordered NMR spectroscopy (DOSY) experiments were performed using a Bruker® Avance III 600 MHz operating at 600.25 MHz for protons, equipped with a 5-mm high-resolution TCI cryoprobe and with pulsed gradient units, capable of producing magnetic field pulsed gradients in the z-direction of 0.64 $T.m^{-1}$. All measurements were performed at 298 K. Temperature was certified by internal NMR calibration samples from Bruker®. NMR Data was processed, evaluated, and plotted with TopSpin 3.2 software and with a python script. Further analysis of the measurements was performed with Microsoft Excel (Version 16.0.10359.20023 64 Bit) and a python script.

Sample preparation for diffusion-ordered spectroscopy

For the sample preparation 1.0 mL stock solutions with a concentration of 1/5 mM were prepared of ion pair **3a** in freshly distilled deuterated solvents (CD_2Cl_2). These stock solutions were then diluted to the respective concentrations. Thereafter the stock solution was put in an ultrasonic bath for one minute before it was used. Before the addition of the stock solutions, the NMR tubes were evacuated and flushed with Argon three times. Then the stock solution was added to the NMR tube under Argon flow. Tetramethyl silane (TMS) was added as a reference by withdrawing 0.5 mL from the atmosphere of the TMS bottle, just above the surface of the liquid. Afterwards, the NMR tube was sealed and again put in an ultrasonic bath for one minute before use.

Diffusion ordered spectroscopy (DOSY)

All DOSY measurements were performed with the convection suppressing DSTE (double stimulated echo) pulse sequence developed by Jerschow and Müller in a pseudo 2D mode.[10,11] Therefore, TMS was added to the sample and used as reference for the ^1H chemical shifts and for hydrodynamic radius as well as the viscosity of the solvent. For the measurement a set of 4 dummy scans and 32 scans was used for samples with concentration ≥ 0.5 mM of the ion pair. A set of 4 dummy scans and 64 scans were used for samples with concentrations ≥ 0.1 mM of the ion pair. A set of 4 dummy scans and 256 scans was used for samples with less than 0.05 mM of the ion pair. A relaxation delay of 3.5 s was used for all samples. The diffusion time delay was set to 40 ms and the gradient pulse lengths (p30, SINE.100 pulse shape) were optimized for each species to give a sigmoidal signal decay for varying gradient strengths between 5% and 95%. Optimal pulse lengths of 1.12 – 1.3 ms were used at 298 K for TMS and ion pairs. For each species, 32 spectra with linear varying gradient strength from 5% to 95% were measured. Thereby, no line broadening occurred for increased gradient strengths. For integration the corresponding signals of **3a** were used except if there was an overlap with another signal (see Figure S10).

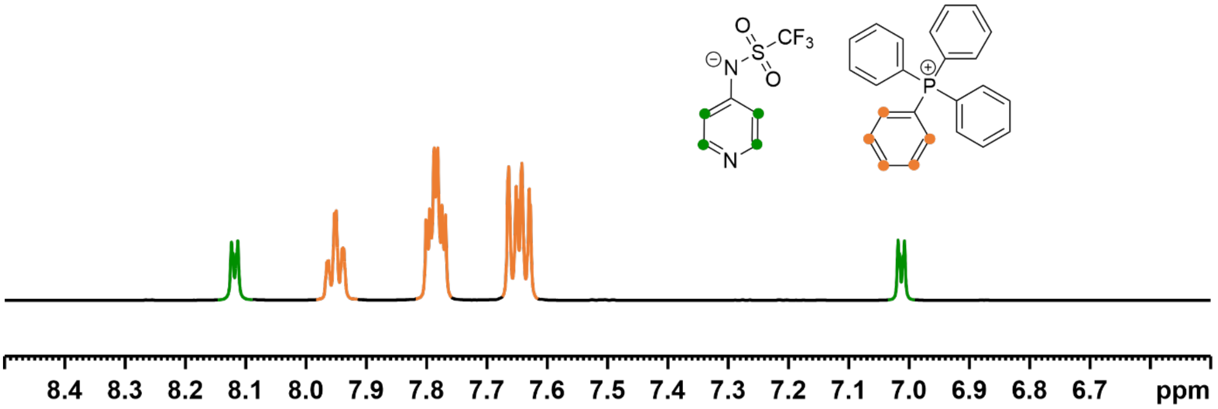

Figure S10. Signals used for DOSY evaluation of **3a**. Anion signals are marked in green and cation signals are marked in orange. All corresponding signals were used for DOSY evaluation and averaged for each ion.

One possible concern at experimental durations of 16 h and CD_2Cl_2 as solvent is a concentration change due to evaporation. Therefore, both parafilm sealed normal tubes and Young tubes were tested using one-dimensional spectra prior and after the DOSY experiments. No change was observed for both kinds of tubes. Convection has to be eliminated even at room temperature because we used a cryo probe.[11] The pulse sequence developed by Jerschow and Mueller[10] in a pseudo 2D mode addresses this issue, but is known to reduce signal to noise down to 40 % compared to non-diffusion compensated pulse sequences. Selective

repetitions showed the reproducibility using this NMR setup. We found that the sample preparation is more critical. Sonification was necessary to break up the ionic aggregates existing in the stock solutions. Tests showed that sonification over 5 min is sufficient since no change between sonification over 5 or 30 min was observed.

In general, the transformation of diffusion coefficients into hydrodynamic radii via the Stejskal-Tanner equation is applicable for neutral molecules. In the case of ions, the additional electrostatic interactions between counter ions should be included and are expected to reduce the mobility of the ions at higher concentrations. In this manuscript extremely low concentrations of ions between 0.005 mM and 1.0 mM are applied, therefore the electrostatic interactions should be negligible. Indeed, DOSY/Steijskal-Tanner/Stokes-Einstein approach for measurements at 0.005 mM were in good agreement with the calculated volumes of the free ions. Furthermore, upon concentration increase the DOSY data don't show any direct correlation to the concentration but a concentration dependence which in combination with the conductivity data and the nucleophilicity data is only consistent with the sandwich formation (see main text). In addition, for the interpretation mainly the relative values of cations and anions i.e. the offset is important, and this shouldn't be affected by the electrostatic interactions. Therefore, the signal intensities of these groups in the DOSY spectra were classically analyzed as a function of the gradient strength by the in Bruker TopSpin 3.2 included software T1/T2 relaxation package by employing the Stejskal-Tanner equation.[12] Based on the obtained translational diffusion coefficients, the hydrodynamic radii of the analytes r_H were estimated following the Stokes-Einstein equation (S10), with D_i = self-diffusion coefficient k_B = Boltzmann constant, T = temperature, η = viscosity of the sample, c = correcting factor, F = shape factor:[13]

$$D_i = \frac{k_B T}{F c \pi \eta r_H} \qquad \text{(S10)}$$

The shape factor F was set to 1 for a spherical shape. The semi-empirical modification by Chen (S11) was used to calculate the correction factor c. Therefore, a from literature known value for the radius of the corresponding solvent was used ($r_{CD_2Cl_2}$ = 2.46 Å[14]).[15]

$$c_{Chen} = \frac{6F}{1 + 0.695\,(\frac{r_{solv}}{r_{ref}})^{2.234}} \qquad \text{(S11)}$$

Viscosity calibration of the derived D_i values was performed with literature known values for the radii of TMS (r_{ref} = 2.96 Å, calculated from hard-sphere increments[16]) and the experimentally determined diffusion coefficient D_{ref} of TMS, which is determined individually for each sample.

$$\eta\ [kg/ms] = \frac{k_B T\ (1 + 0.695 \left(\frac{r_{solv}}{r_{ref}}\right)^{2.234})}{6\pi D_{ref}\ r_{ref}} \qquad \text{(S12)}$$

After including all correction and calibration equations in the Stokes equation (S10), the equation was rearranged for the hydrodynamic radii r_H (S13). For easier imagination, the corresponding volumes V_A were calculated with the assumption of a spherical shape.

$$D = \frac{k_B T\ (1 + 0.695 \left(\frac{r_{solv}}{r_{ref}}\right)^{2.234})}{6\pi \eta r_H} \qquad \text{(S13)}$$

The experimental self-diffusion coefficients D_i, the viscosity corrected hydrodynamic radii r_H and the resulting volumes V_A of all samples are depicted in Table S9 -13. TMS was used as viscosity reference in each sample separately with the variation in its experimental diffusion coefficients reflecting the different viscosities depending on the ion pair concentration (see Table S9-13). The average D_i values were derived by using all baseline separated signals that were referring to the same species. As stated above the hydrodynamic values

and the volumes are viscosity corrected and therefore the only values, which can be directly compared with each other.

Table S9. Experimental self-diffusion coefficients D_i, viscosity corrected hydrodynamic radii r_H and resulting volumes V_A of the ion pair **3c** in varying concentrations. TMS was used as viscosity reference for the experimental self-diffusion coefficients D_i to allow for a comparison of hydrodynamic radii r_H and resulting volumes V_A. The corresponding self-diffusion coefficients D_i of TMS are given for each sample. Entry 1-3: SW = 22 Hz, O1P = 10.0 ppm, gradient strength 5-95% linear. Samples were measured at room temperature.

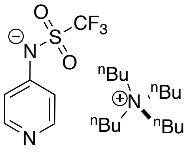

3c

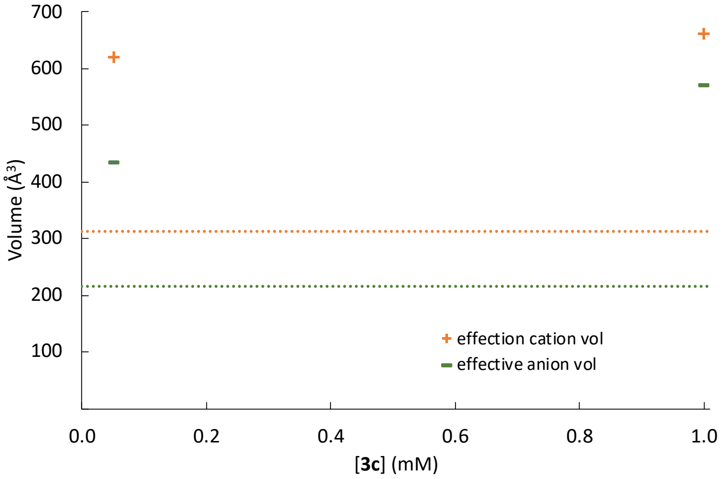

Sample	solvent	Species inside sample	Concentration [mM]	Diffusion coefficient D_i [$m^2.s^{-1}$]	Hydrodynamic radius r_H [Å]	Volume V_A [$Å^3$]	R^2
**1**	CD_2Cl_2	**3c** Anion<br>**3c** Cation<br>TMS	0.05<br>0.05	1.30 x 10^{-9}<br>1.11 x 10^{-9}<br>2.58 x 10^{-9}	4.70<br>5.32	434<br>630	0.999<br>0.997
**2**	CD_2Cl_2	**3c** Anion<br>**3c** Cation<br>TMS	1.0<br>1.0	1.14 x 10^{-9}<br>1.07 x 10^{-9}<br>2.55 x 10^{-9}	5.15<br>5.4	571<br>660	0.998<br>0.998
**3**	CD_2Cl_2	**3c** Anion<br>**3c** Cation<br>TMS	5.0<br>5.0	1.05 x 10^{-9}<br>1.04 x 10^{-9}<br>2.46 x 10^{-9}	5.34<br>5.34	640<br>651	0.999<br>0.999

Table S10. Experimental self-diffusion coefficients D_i, viscosity corrected hydrodynamic radii r_H and resulting volumes V_A of the ion pair **3b** in varying concentrations. TMS was used as viscosity reference for the experimental self-diffusion coefficients D_i to allow for a comparison of hydrodynamic radii r_H and resulting volumes V_A. The corresponding self-diffusion coefficients D_i of TMS are given for each sample. Entry 4-7: SW = 22 Hz, O1P = 10.0 ppm, gradient strength 5-95% linear. Samples were measured at room temperature.

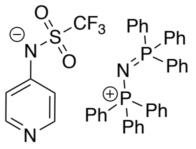

3b

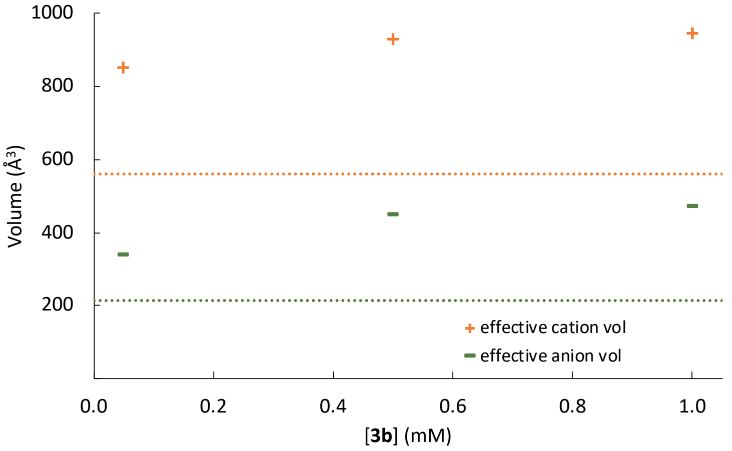

Sample	solvent	Species inside sample	Concentration [mM]	Diffusion coefficient D_i [$m^2.s^{-1}$]	Hydrodynamic radius r_H [Å]	Volume V_A [$Å^3$]	R^2
4	CD_2Cl_2	**3b** Anion	0.05	1.45×10^{-9}	4.32	337	0.998
		**3b** Cation	0.05	9.82×10^{-10}	5.88	850	0.999
		TMS		2.59×10^{-9}			
5	CD_2Cl_2	**3b** Anion	0.5	1.25×10^{-9}	4.74	447	0.998
		**3b** Cation	0.5	9.24×10^{-10}	6.05	927	0.999
		TMS		2.52×10^{-9}			
6	CD_2Cl_2	**3b** Anion	1.0	1.22×10^{-9}	4.83	473	0.999
		**3b** Cation	1.0	9.18×10^{-10}	6.09	944	0.999
		TMS		2.52×10^{-9}			
7	CD_2Cl_2	**3b** Anion	5.0	1.11×10^{-9}	5.16	577	0.999
		**3b** Cation	5.0	8.99×10^{-10}	6.14	969	0.999
		TMS		2.49×10^{-9}			

Table S11. Experimental self-diffusion coefficients D_i, viscosity corrected hydrodynamic radii r_H and resulting volumes V_A of the ion pair **3d** in varying concentrations. TMS was used as viscosity reference for the experimental self-diffusion coefficients D_i to allow for a comparison of hydrodynamic radii r_H and resulting volumes V_A. The corresponding self-diffusion coefficients D_i of TMS are given for each sample. Entry 8-12: SW = 22 Hz, O1P = 10.0 ppm, gradient strength 5-95% linear. Samples were measured at room temperature.

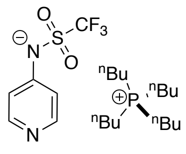

3d

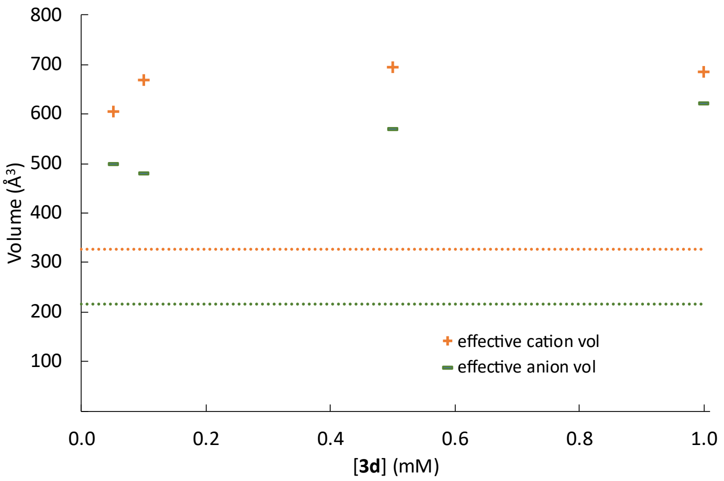

Sample	solvent	Species inside sample	Concentration [mM]	Diffusion coefficient D_i [$m^2.s^{-1}$]	Hydrodynamic radius r_H [Å]	Volume V_A [$Å^3$]	R^2
8	CD_2Cl_2	**3d** Anion	0.05	1.00×10^{-9}	5.85	497	0.999
		**3d** Cation	0.05	1.03×10^{-9}	5.71	603	0.999
		TMS		2.63×10^{-9}			
9	CD_2Cl_2	**3d** Anion	0.1	1.24×10^{-9}	4.85	479	0.998
		**3d** Cation	0.1	1.08×10^{-9}	5.42	668	0.999
		TMS		2.58×10^{-9}			
10	CD_2Cl_2	**3d** Anion	0.5	1.15×10^{-9}	5.14	569	0.999
		**3d** Cation	0.5	1.06×10^{-9}	5.49	694	0.999
		TMS		2.57×10^{-9}			
11	CD_2Cl_2	**3d** Anion	1.0	1.09×10^{-9}	5.29	621	0.999
		**3d** Cation	1.0	1.05×10^{-9}	5.47	684	0.999
		TMS		2.53×10^{-9}			
12	CD_2Cl_2	**3d** Anion	5.0	1.04×10^{-9}	5.34	639	0.999
		**3d** Cation	5.0	1.01×10^{-9}	5.50	696	0.999
		TMS		2.45×10^{-9}			

Table S12. Experimental self-diffusion coefficients D_i, viscosity corrected hydrodynamic radii r_H and resulting volumes V_A of the ion pair **5a** in varying concentrations. TMS was used as viscosity reference for the experimental self-diffusion coefficients D_i to allow for a comparison of hydrodynamic radii r_H and resulting volumes V_A. The corresponding self-diffusion coefficients D_i of TMS are given for each sample. Entry 13-16: SW = 22 Hz, O1P = 10.0 ppm, gradient strength 5-95% linear. Samples were measured at room temperature.

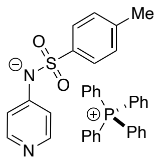

5a

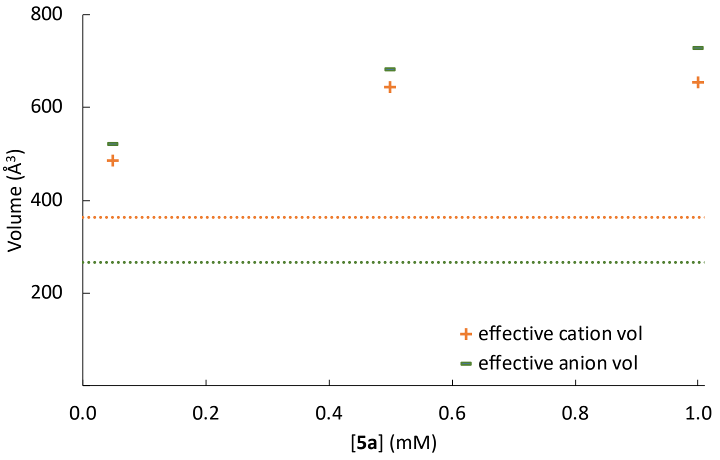

Sample	solvent	Species inside sample	Concentration [mM]	Diffusion coefficient D_i [$m^2.s^{-1}$]	Hydrodynamic radius r_H [Å]	Volume V_A [Å^3]	R^2
**13**	CD_2Cl_2	**5a** Anion	0.05	1.18×10^{-9}	4.99	519	0.999
		**5a** Cation	0.05	1.22×10^{-9}	4.87	485	0.997
		TMS		2.54×10^{-9}			
**14**	CD_2Cl_2	**5a** Anion	0.5	1.04×10^{-9}	5.46	681	0.999
		**5a** Cation	0.5	1.06×10^{-9}	5.36	643	0.999
		TMS		2.50×10^{-9}			
**15**	CD_2Cl_2	**5a** Anion	1.0	1.00×10^{-9}	5.58	727	0.999
		**5a** Cation	1.0	1.05×10^{-9}	4.38	652	0.999
		TMS		2.49×10^{-9}			
**16**	CD_2Cl_2	**5a** Anion	5.0	9.31×10^{-10}	5.96	888	0.999
		**5a** Cation	5.0	9.95×10^{-10}	5.64	753	0.999
		TMS		2.50×10^{-9}			

Table S13. Experimental self-diffusion coefficients D_i, viscosity corrected hydrodynamic radii r_H and resulting volumes V_A of the ion pair **6a** in varying concentrations. TMS was used as viscosity reference for the experimental self-diffusion coefficients D_i to allow for a comparison of hydrodynamic radii r_H and resulting volumes V_A. The corresponding self-diffusion coefficients D_i of TMS are given for each sample. Entry 17-20: SW = 22 Hz, O1P = 10.0 ppm, gradient strength 5-95% linear. Samples were measured at room temperature.

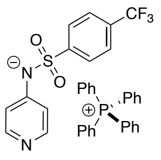

6a

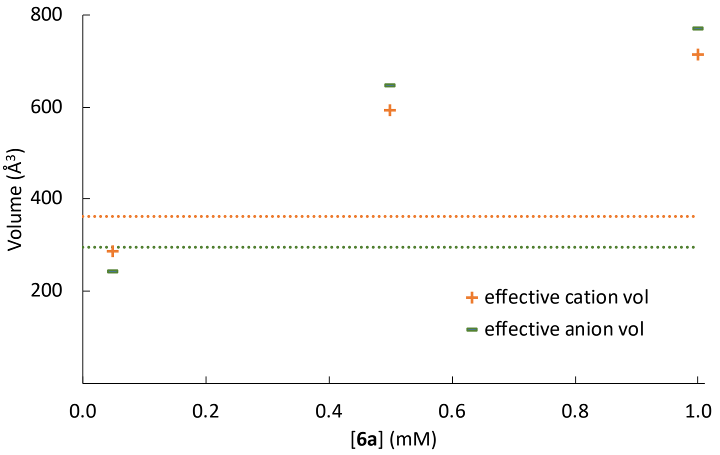

Sample	solvent	Species inside sample	Concentration [mM]	Diffusion coefficient D_i [$m^2.s^{-1}$]	Hydrodynamic radius r_H [Å]	Volume V_A [Å^3]	R^2
**17**	CD_2Cl_2	**6a** Anion	0.05	1.19×10^{-9}	3.87	243	0.999

		**6a** Cation	0.05	1.10 x 10^{-9}	4.09	286	0.999
		TMS		1.81 x 10^{-9}			
**18**	CD_2Cl_2	**6a** Anion	0.5	1.03 x 10^{-9}	5.36	646	0.999
		**6a** Cation	0.5	1.06 x 10^{-9}	5.21	594	0.999
		TMS		2.42 x 10^{-9}			
**19**	CD_2Cl_2	**6a** Anion	1.0	1.01 x 10^{-9}	5.69	772	0.999
		**6a** Cation	1.0	1.04 x 10^{-9}	5.54	714	0.999
		TMS		2.55 x 10^{-9}			
**20**	CD_2Cl_2	**6a** Anion	5.0	9.66 x 10^{-10}	5.86	841	0.999
		**6a** Cation	5.0	1.00 x 10^{-9}	5.68	769	0.999
		TMS		2.54 x 10^{-9}			

5. Nucleophilicity Data

The reaction rates of ion pair catalysts and **TCAP** and the reference electrophiles **8** were measured photometrically on a stop-flow spectrophotometer. The temperature was controlled with a circulating bath thermostat. The kinetic reactions were carried out under pseudo-first-order conditions (ion pair = nucleophile, excess compound) at the absorption maximum of benzhydrylium ion in the respective solvent as described previously by the H. Mayr group.[17] First-order rate constants k_{obs} (s^{-1}) were derived by fitting the absorbance according to the mono-exponential curve $A_t = A_0 \exp(-k_{obs}\, t) + C$ (see Figure S11).

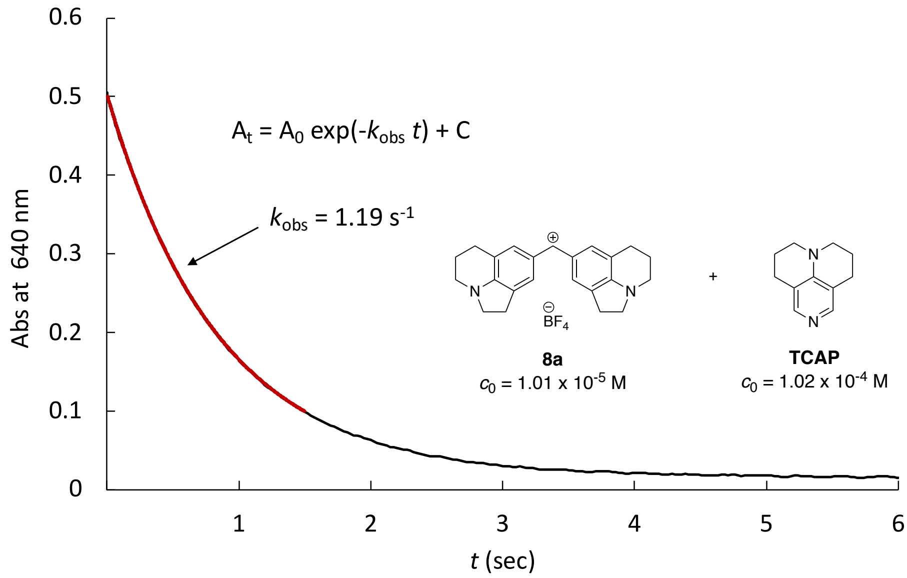

Figure S11. Absorbance decay of benzhydrylium salt **8a** reacting with nucleophile TCAP (**2**) in DCM at 20°C, fitted by mono-exponential decay function resulting in $k_{obs} = 1.19\ s^{-1}$.

Then the second-order rate constant k_2 ($L\ mol^{-1}\ s^{-1}$) is obtained from the slope of the linear plots of k_{obs} (s^{-1}) vs [Nu] since $k_{obs} = k_2$[Nu]. The subsequent characterization is based on the Mayr-Patz eq. S14 where the reaction rates for the reaction of the nucleophile with the electrophile is expressed as a function of the nucleophilicity parameter N, the associated sensitivity parameter s_N, and the electrophilicity parameter E. Both, N parameter and nucleophile-specific s_N parameter are solvent dependent.[17–19]

$$\log k_2^{20\,°C} = s_N(N + E) \qquad \text{(S14)}$$

5.1 Kinetic Data Analysis

Measurements of ionic compounds performed in non-polar solvents require more consideration due to the influence of ion association on the first-order rate constant k_{obs}. To negate the effect of ion association in solution, the ionic strength controlled benzhydrylium method was developed where a non-nucleophilic, structurally related salt was added as additive to keep ionic strength I constant throughout the kinetic.

Kinetic data gathered under conditions of ionic strength control I = 1.0 mM, can be analyzed in multiple ways (for full discussion see SI, Chapter 6 of ref [2]).

The most practical way would be to assume that the amount of weighted salt equals the amount of nucleophile in solution. Here, the standard Mayr's method would be employed to obtain the second-order rate constant k_2 and subsequently gain the N parameter and s_N parameter.

The combination of conductivity and DOSY NMR, however, revealed a complex ion association taking place in solution, where the single ions associate into distinctive triple ion complexes of the type, **CAC**, and **ACA**. It is assumed that the remaining free anion **A** is the only reactive nucleophile in solution.

To determine the concentration of free nucleophile **A** in solution, the conductivity model III was used as basis. Since the association of the pyridinamide ion pair is controlled *via* the addition of the additive salt, the

association of the additive itself has to be considered. The existing model **III** was extended by adding the association equation for the additive to it (see Scheme S5).

$$2 * \mathrm{A} + 2 * \mathrm{C} \xrightleftharpoons{\alpha \times K_{\mathrm{CAC}}} \mathrm{A} + \mathrm{CAC}$$

$$2 * \mathrm{A} + 2 * \mathrm{C} \xrightleftharpoons{\beta \times K_{\mathrm{ACA}}} \mathrm{C} + \mathrm{ACA}$$

$$2 * \mathrm{C} + 2 * \mathrm{B} \xrightleftharpoons{K_{\mathrm{CAC}}(\mathrm{add})} \mathrm{B} + \mathrm{CBC}$$

Scheme S5. Numerical simulation equations of mixed sandwich association model extension for evaluation of ionic strength controlled kinetic data.

Since there are two pyridinamide ion pairs, **3b** and **3d**, whose association pattern could not be analyzed with the mixed sandwich association model, an alternative extension for the nucleophilicity data evaluation will be included here. DOSY NMR data revelated the cationic sandwich association as a suitable association model for pyridinamide ion pairs with the deprotonated 1,1,1-trifluoro-*N*-(pyridin-4(1*H*)-ylidene)methanesulfonamide as anion (see Chapter 4). As an alternative, model **II**a was used as basis for another extension for the analysis of the ionic strength-controlled benzhydrylium method (see Scheme S6).

$$2 * \mathrm{A} + 2 * \mathrm{C} \xrightleftharpoons{K_{\mathrm{CAC}}} \mathrm{A} + \mathrm{CAC}$$

$$2 * \mathrm{C} + 2 * \mathrm{B} \xrightleftharpoons{K_{\mathrm{CAC}}(\mathrm{add})} \mathrm{B} + \mathrm{CBC}$$

Scheme S6. Numerical simulation equations of cationic sandwich association model extension for evaluation of ionic strength controlled kinetic data.

Now, both models were used to evaluate the gathered kinetic data. For completeness, all kinetic data sets were analyzed utilizing both association extensions to obtain the respective nucleophilicity parameters N and s_N. Numerical simulation to obtain the relevant ion concentrations were performed using COPASI, whereas the individual starting concentrations are defined as:

– [**A**] = concentration of pyridinamide salt

– [**C**] = concentration of pyridinamide salt + concentration of additive

– [**B**] = concentration of additive + concentration of the respective benzhydrylium salt.

The resulting N- and s_N-parameter of the ionic-strength controlled benzhydrylium method for pyridinamide ion pairs in DCM are summarized in Table S14. The applied models are distinguished by superscripts: "HC" = [**A**]$_{tot}$ with I = 1.0 mM, "HC,sw" = [**A**] obtained by model **II**a extension with I = 1.0 mM, and "HC,mix" = [**A**] obtained by model **III** extension with I = 1.0 mM.

Table S14. List of N- and s_N-parameter obtained with different evaluation models for pyridinamide ion pairs in DCM.

**Ion Pair**	**N/s_N parameter**		
	**HC**	**HC,sw**	**HC,mix**
**3d**	17.12/0.77	17.25/0.77	–
**3c**	17.20/0.76	17.33/0.76	17.32/0.76
**3b**	17.41/0.75	17.65/0.75	17.65/0.75
**3a**	17.40/0.75	17.61/0.75	17.88/0.73
**5a**	19.24/0.66	19.47/0.66	19.42/0.66
**4a**	19.35/0.65	19.65/0.65	19.63/0.65
**6a**	17.66/0.72	18.53/0.72	18.43/0.72

5.2 Nucleophilicity data in MeCN

Nucleophilicity of ion pair 3b in MeCN at 20 °C

Reaction of **3b** with (lil)$_2$CH$^+$BF$_4^-$ (stopped-flow, λ = 632 nm)

[8a] (mol L^{-1})	[3b] (mol L^{-1})	k_{obs} (s^{-1})
	9.81 x 10^{-6}	0.38
	1.47 x 10^{-5}	0.42
9.41 x 10^{-7}	1.96 x 10^{-5}	0.46
	2.45 x 10^{-5}	0.51
	2.94 x 10^{-5}	0.54

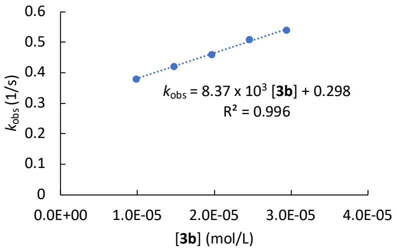

k_2 = 8.37 x 10^3 L mol^{-1} s^{-1}

Reaction of **3b** with (jul)$_2$CH$^+$BF$_4^-$ (stopped-flow, λ = 635 nm)

[8b] (mol L^{-1})	[3b] (mol L^{-1})	k_{obs} (s^{-1})
	9.81 x 10^{-6}	0.82
	1.47 x 10^{-5}	0.89
9.67 x 10^{-7}	1.96 x 10^{-5}	0.95
	2.45 x 10^{-5}	–
	2.94 x 10^{-5}	1.09

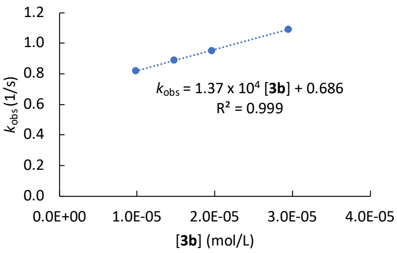

k_2 = 1.37 x 10^4 L mol^{-1} s^{-1}

Reaction of **3b** with (ind)$_2$CH$^+$BF$_4^-$ (stopped-flow, λ = 616 nm)

[8c] (mol L^{-1})	[3b] (mol L^{-1})	k_{obs} (s^{-1})
	9.81 x 10^{-6}	0.56
	1.47 x 10^{-5}	0.86
1.07 x 10^{-6}	1.96 x 10^{-5}	1.08
	2.45 x 10^{-5}	1.30
	2.94 x 10^{-5}	1.50

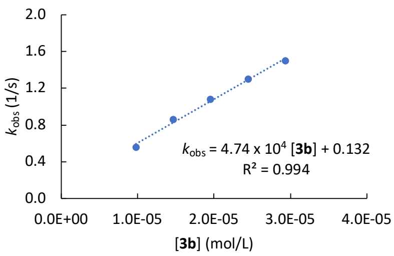

k_2 = 4.74 x 10^4 L mol^{-1} s^{-1}

Determination of N and s_N parameter for **3b** in MeCN

Electrophile	E	k_2 (M^{-1} s^{-1})
8a	-10.04	8.37 x 10^3
8b	-9.45	1.37 x 10^4
8c	-8.76	4.74 x 10^4

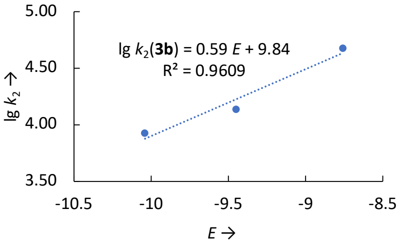

N = 16.68 **s_N = 0.59**

Nucleophilicity of ion pair 3c in MeCN at 20 °C

Reaction of **3c** with $(lil)_2CH^+BF_4^-$ (stopped-flow, λ = 632 nm)

[8a] (mol L^{-1})	[3c] (mol L^{-1})	k_{obs} (s^{-1})
	9.94×10^{-6}	0.33
	1.49×10^{-5}	0.38
1.09×10^{-6}	1.99×10^{-5}	0.41
	2.49×10^{-5}	0.46
	2.98×10^{-5}	0.50

$k_2 = 8.45 \times 10^3$ L mol^{-1} s^{-1}

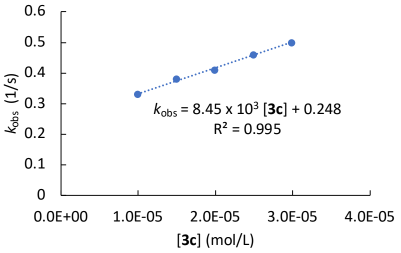

Reaction of **3c** with $(jul)_2CH^+BF_4^-$ (stopped-flow, λ = 635 nm)

[8b] (mol L^{-1})	[3c] (mol L^{-1})	k_{obs} (s^{-1})
	9.94×10^{-6}	0.82
	1.49×10^{-5}	0.89
1.04×10^{-6}	1.99×10^{-5}	0.92
	2.49×10^{-5}	1.01
	2.98×10^{-5}	1.10

$k_2 = 1.37 \times 10^4$ L mol^{-1} s^{-1}

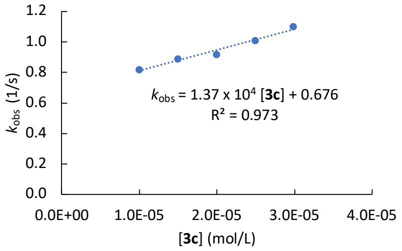

Reaction of **3c** with $(ind)_2CH^+BF_4^-$ (stopped-flow, λ = 616 nm)

[8c] (mol L^{-1})	[3c] (mol L^{-1})	k_{obs} (s^{-1})
	9.94×10^{-6}	0.54
	1.49×10^{-5}	0.86
9.27×10^{-7}	1.99×10^{-5}	1.06
	2.49×10^{-5}	1.34
	2.98×10^{-5}	1.49

$k_2 = 4.97 \times 10^4$ L mol^{-1} s^{-1}

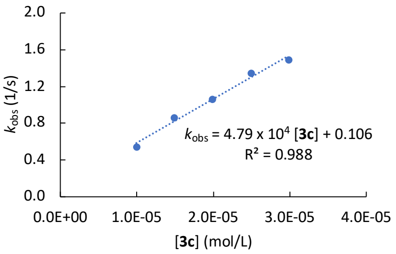

Determination of *N* and s_N parameter for **3c** in MeCN

Electrophile	*E*	k_2 (m^{-1} s^{-1})
8a	-10.04	8.45×10^3
8b	-9.45	1.37×10^4
8c	-8.76	4.79×10^4

N = 16.68 **s_N = 0.59**

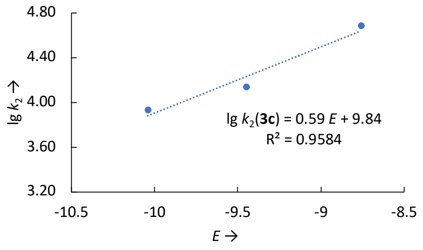

Nucleophilicity of ion pair 3d in MeCN at 20 °C

Reaction of **3d** with $(lil)_2CH^+BF_4^-$ (stopped-flow, λ = 632 nm)

[8a] (mol L^{-1})	[3d] (mol L^{-1})	k_{obs} (s^{-1})
	9.82 x 10^{-6}	0.33
	1.47 x 10^{-5}	0.38
9.69 x 10^{-7}	1.96 x 10^{-5}	0.42
	2.45 x 10^{-5}	0.46
	2.95 x 10^{-5}	–

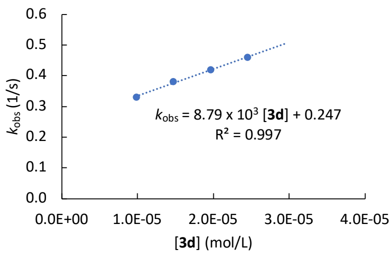

k_2 = 8.79 x 10^3 L mol^{-1} s^{-1}

Reaction of **3d** with $(jul)_2CH^+BF_4^-$ (stopped-flow, λ = 635 nm)

[8b] (mol L^{-1})	[3d] (mol L^{-1})	k_{obs} (s^{-1})
	9.82 x 10^{-6}	0.81
	1.47 x 10^{-5}	0.88
9.56 x 10^{-7}	1.96 x 10^{-5}	0.96
	2.45 x 10^{-5}	1.05
	2.95 x 10^{-5}	–

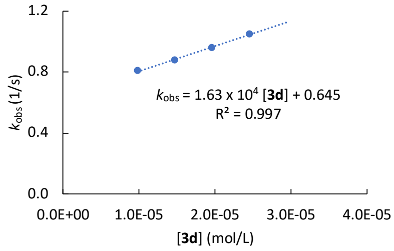

k_2 = 1.63 x 10^4 L mol^{-1} s^{-1}

Reaction of **3d** with $(ind)_2CH^+BF_4^-$ (stopped-flow, λ = 616 nm)

[8c] (mol L^{-1})	[3d] (mol L^{-1})	k_{obs} (s^{-1})
	9.82 x 10^{-6}	0.56
	1.47 x 10^{-5}	0.86
8.98 x 10^{-7}	1.96 x 10^{-5}	1.10
	2.45 x 10^{-5}	1.30
	2.95 x 10^{-5}	–

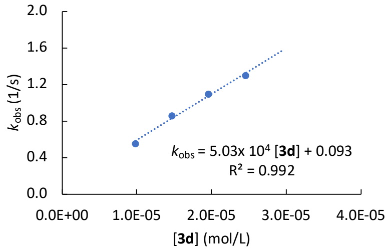

k_2 = 5.03 x 10^4 L mol^{-1} s^{-1}

Determination of *N* and s_N parameter for **3d** in MeCN

Electrophile	*E*	k_2 (M^{-1} s^{-1})
8a	-10.04	8.79 x 10^3
8b	-9.45	1.63 x 10^4
8c	-8.76	5.03 x 10^4

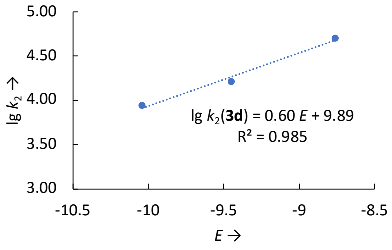

N = 16.48 **s_N = 0.60**

Nucleophilicity of ion pair 5a in MeCN at 20 °C

Reaction of **5a** with $(lil)_2CH^+BF_4^-$ (stopped-flow, λ = 632 nm)

[8a] (mol L^{-1})	[5a] (mol L^{-1})	k_{obs} (s^{-1})
	1.00×10^{-5}	–
	2.01×10^{-5}	0.64
9.41×10^{-7}	3.01×10^{-5}	1.08
	4.02×10^{-5}	1.42
	5.02×10^{-5}	1.68

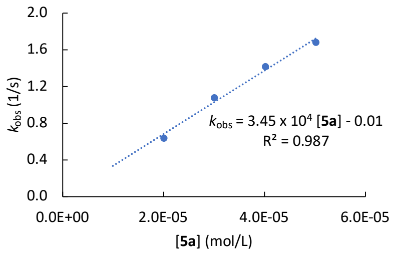

$k_2 = 3.45 \times 10^4$ L mol^{-1} s^{-1}

Reaction of **5a** with $(jul)_2CH^+BF_4^-$ (stopped-flow, λ = 635 nm)

[8b] (mol L^{-1})	[5a] (mol L^{-1})	k_{obs} (s^{-1})
	1.00×10^{-5}	0.38
	2.01×10^{-5}	1.54
1.17×10^{-6}	3.01×10^{-5}	2.51
	4.02×10^{-5}	3.31
	5.02×10^{-5}	4.20

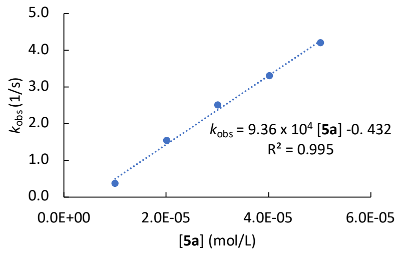

$k_2 = 9.36 \times 10^4$ L mol^{-1} s^{-1}

Reaction of **5a** with $(ind)_2CH^+BF_4^-$ (stopped-flow, λ = 616 nm)

[8c] (mol L^{-1})	[5a] (mol L^{-1})	k_{obs} (s^{-1})
	1.00×10^{-5}	1.46
	2.01×10^{-5}	3.6
1.14×10^{-6}	3.01×10^{-5}	5.91
	4.02×10^{-5}	8.42
	5.02×10^{-5}	10.6

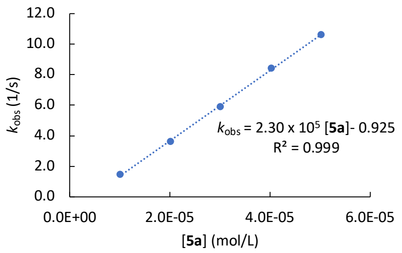

$k_2 = 2.30 \times 10^5$ L mol^{-1} s^{-1}

Determination of N and s_N parameter for **5a** in MeCN

Electrophile	E	k_2 (M^{-1} s^{-1})
8a	-10.04	3.45×10^4
8b	-9.45	9.36×10^4
8c	-8.76	2.30×10^5

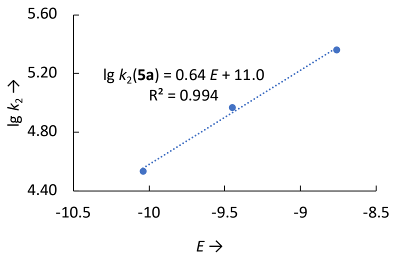

N = 17.19 **s_N = 0.64**

Nucleophilicity of ion pair 6a in MeCN at 20 °C

Reaction of **6a** with $(lil)_2CH^+BF_4^-$ (stopped-flow, λ = 632 nm)

[8a] (mol L^{-1})	[6a] (mol L^{-1})	k_{obs} (s^{-1})
	9.92×10^{-6}	0.16
	1.49×10^{-5}	0.32
9.96×10^{-7}	1.98×10^{-5}	0.48
	2.48×10^{-5}	0.64
	2.98×10^{-5}	0.75

$k_2 = 3.02 \times 10^4$ L mol^{-1} s^{-1}

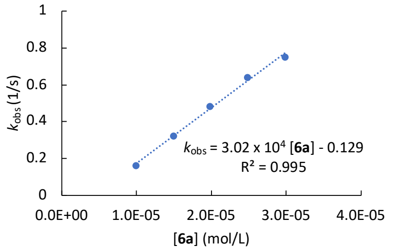

Reaction of **6a** with $(jul)_2CH^+BF_4^-$ (stopped-flow, λ = 635 nm)

[8b] (mol L^{-1})	[6a] (mol L^{-1})	k_{obs} (s^{-1})
	9.92×10^{-6}	0.58
	1.49×10^{-5}	0.85
1.02×10^{-6}	1.98×10^{-5}	1.18
	2.48×10^{-5}	1.50
	2.98×10^{-5}	1.73

$k_2 = 5.94 \times 10^4$ L mol^{-1} s^{-1}

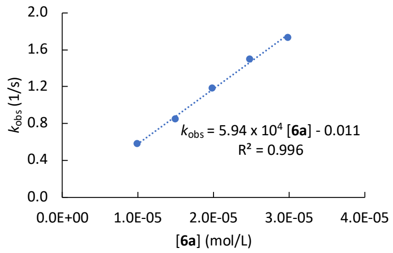

Reaction of **6a** with $(ind)_2CH^+BF_4^-$ (stopped-flow, λ = 616 nm)

[8c] (mol L^{-1})	[6a] (mol L^{-1})	k_{obs} (s^{-1})
	9.92×10^{-6}	1.22
	1.49×10^{-5}	1.97
9.13×10^{-7}	1.98×10^{-5}	2.50
	2.48×10^{-5}	3.12
	2.98×10^{-5}	3.61

$k_2 = 1.19 \times 10^5$ L mol^{-1} s^{-1}

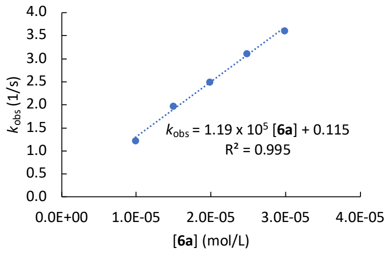

Determination of N and s_N parameter for **6a** in MeCN

Electrophile	E	k_2 (M^{-1} s^{-1})
8a	-10.04	3.02×10^4
8b	-9.45	6.26×10^4
8c	-8.76	1.19×10^5

N = 19.47 **s_N = 0.47**

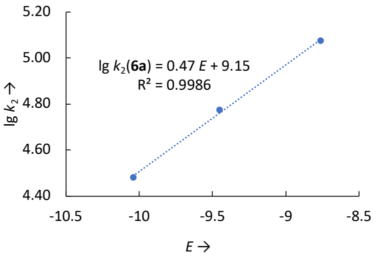

5.3 Nucleophilicity data in DCM at 0.01 – 0.03 mM

Nucleophilicity of ion pair 3b in DCM at 20 °C

Reaction of **3b** with $(lil)_2CH^+BF_4^-$ (stopped-flow, λ = 640 nm)

[8a] (mol L^{-1})	[3b] (mol L^{-1})	k_{obs} (s^{-1})
	9.88×10^{-6}	–
	1.48×10^{-5}	21.0
1.05×10^{-6}	1.98×10^{-5}	27.6
	2.47×10^{-5}	35.8
	2.96×10^{-5}	41.9

$k_2 = 1.44 \times 10^6$ L mol^{-1} s^{-1}

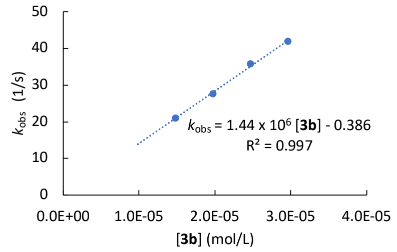

Reaction of **3b** with $(jul)_2CH^+BF_4^-$ (stopped-flow, λ = 643 nm)

[8b] (mol L^{-1})	[3b] (mol L^{-1})	k_{obs} (s^{-1})
	9.88×10^{-6}	29.7
	1.48×10^{-5}	–
9.80×10^{-7}	1.98×10^{-5}	60.4
	2.47×10^{-5}	78.0
	2.96×10^{-5}	90.5

$k_2 = 3.13 \times 10^6$ L mol^{-1} s^{-1}

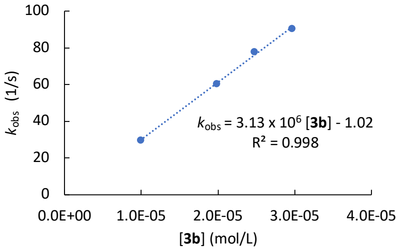

Reaction of **3b** with $(ind)_2CH^+BF_4^-$ (stopped-flow, λ = 626 nm)

[8c] (mol L^{-1})	[3b] (mol L^{-1})	k_{obs} (s^{-1})
	9.88×10^{-6}	119
	1.48×10^{-5}	202
1.01×10^{-6}	1.98×10^{-5}	266
	2.47×10^{-5}	342
	2.96×10^{-5}	–

$k_2 = 1.48 \times 10^7$ L mol^{-1} s^{-1}

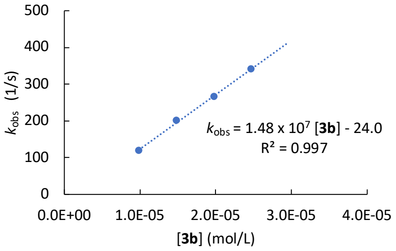

Determination of N and s_N parameter for **3b** in DCM

Electrophile	E	k_2 (M^{-1} s^{-1})
8a	-10.04	1.44×10^6
8b	-9.45	3.13×10^6
8c	-8.76	1.48×10^7

***N* = 17.63** **s_N = 0.80**

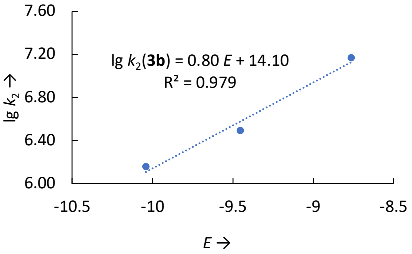

Nucleophilicity of ion pair 3c in DCM at 20 °C

Reaction of **3c** with $(lil)_2CH^+BF_4^-$ (stopped-flow, λ = 640 nm)

[8a] (mol L^{-1})	[3c] (mol L^{-1})	k_{obs} (s^{-1})
1.11 x 10^{-6}	9.49 x 10^{-6}	14.1
	1.42 x 10^{-5}	23.8
	1.90 x 10^{-5}	29.4
	2.37 x 10^{-5}	–
	2.85 x 10^{-5}	–

k_2 = 1.61 x 10^6 L mol^{-1} s^{-1}

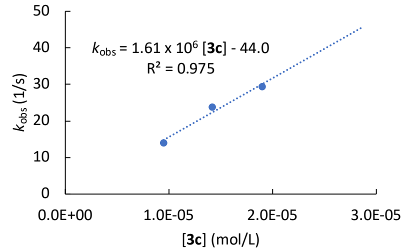

Reaction of **3c** with $(jul)_2CH^+BF_4^-$ (stopped-flow, λ = 643 nm)

[8b] (mol L^{-1})	[3c] (mol L^{-1})	k_{obs} (s^{-1})
9.80 x 10^{-7}	9.49 x 10^{-6}	–
	1.42 x 10^{-5}	38.1
	1.90 x 10^{-5}	48.6
	2.37 x 10^{-5}	62.6
	2.85 x 10^{-5}	78.6

k_2 = 2.85 x 10^6 L mol^{-1} s^{-1}

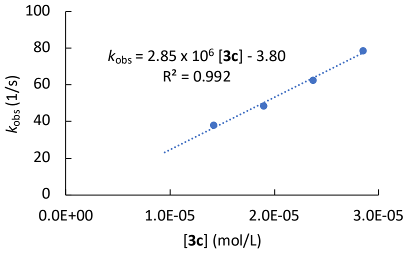

Reaction of **3c** with $(ind)_2CH^+BF_4^-$ (stopped-flow, λ = 626 nm)

[8c] (mol L^{-1})	[3c] (mol L^{-1})	k_{obs} (s^{-1})
8.02 x 10^{-7}	9.49 x 10^{-6}	–
	1.42 x 10^{-5}	170
	1.90 x 10^{-5}	285
	2.37 x 10^{-5}	359
	2.85 x 10^{-5}	435

k_2 = 1.83 x 10^7 L mol^{-1} s^{-1}

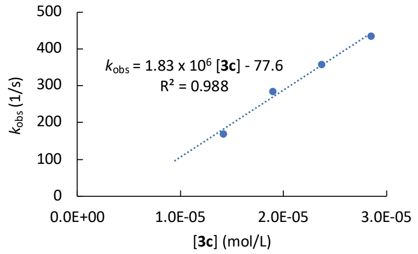

Determination of *N* and s_N parameter for **3c** in DCM

Electrophile	*E*	k_2 (M^{-1} s^{-1})
8a	-10.04	1.61 x 10^6
8b	-9.45	2.85 x 10^6
8c	-8.76	1.83 x 10^7

***N* = 17.47** **s_N = 0.83**

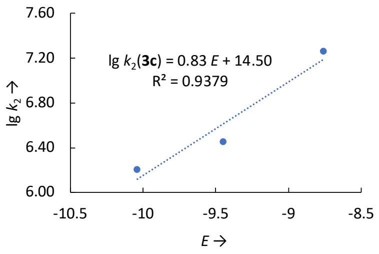

Nucleophilicity of ion pair 3d in DCM at 20 °C

Reaction of **3d** with $(lil)_2CH^+BF_4^-$ (stopped-flow, λ = 640 nm)

[8a] (mol L^{-1})	[3d] (mol L^{-1})	k_{obs} (s^{-1})
	9.98×10^{-6}	–
	1.48×10^{-5}	24.6
9.50×10^{-7}	2.00×10^{-5}	32.6
	2.48×10^{-5}	37.4
	2.99×10^{-5}	45.3

$k_2 = 1.33 \times 10^6$ L mol^{-1} s^{-1}

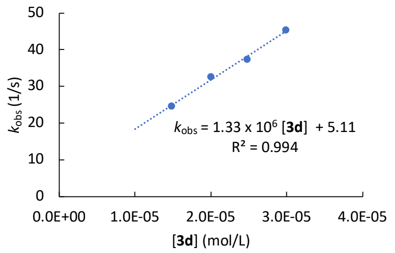

Reaction of **3d** with $(jul)_2CH^+BF_4^-$ (stopped-flow, λ = 643 nm)

[8b] (mol L^{-1})	[3d] (mol L^{-1})	k_{obs} (s^{-1})
	9.98×10^{-6}	28.8
	1.48×10^{-5}	47.7
1.03×10^{-6}	2.00×10^{-5}	–
	2.48×10^{-5}	74.5
	2.99×10^{-5}	92.7

$k_2 = 3.10 \times 10^6$ L mol^{-1} s^{-1}

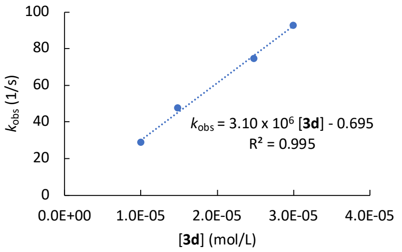

Reaction of **3d** with $(ind)_2CH^+BF_4^-$ (stopped-flow, λ = 626 nm)

[8c] (mol L^{-1})	[3d] (mol L^{-1})	k_{obs} (s^{-1})
	9.98×10^{-6}	111
	1.48×10^{-5}	188
9.56×10^{-7}	2.00×10^{-5}	265
	2.48×10^{-5}	–
	2.99×10^{-5}	411

$k_2 = 1.50 \times 10^7$ L mol^{-1} s^{-1}

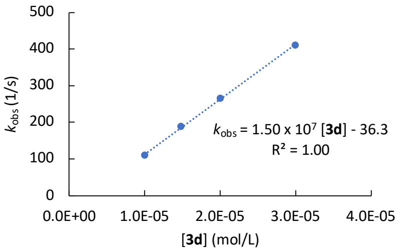

Determination of N and s_N parameter for **3d** in DCM

Electrophile	E	k_2 (M^{-1} s^{-1})
8a	-10.04	1.33×10^6
8b	-9.45	3.10×10^6
8c	-8.76	1.50×10^7

***N* = 17.33** **s_N = 0.83**

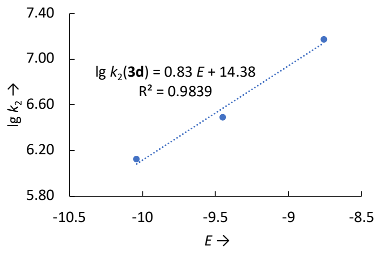

Nucleophilicity of ion pair 4a in DCM at 20 °C

Reaction of **4a** with $(lil)_2CH^+BF_4^-$ (stopped-flow, λ = 640 nm)

[8a] (mol L^{-1})	[4a] (mol L^{-1})	k_{obs} (s^{-1})
	1.01 x 10^{-5}	83.6
	1.49 x 10^{-5}	137
9.87 x 10^{-7}	2.01 x 10^{-5}	193
	2.49 x 10^{-5}	218
	3.02 x 10^{-5}	–

k_2 = 9.24 x 10^7 L mol^{-1} s^{-1}

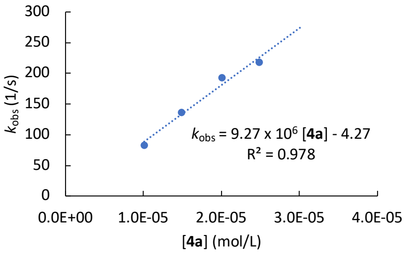

Reaction of **4a** with $(jul)_2CH^+BF_4^-$ (stopped-flow, λ = 643 nm)

[8b] (mol L^{-1})	[4a] (mol L^{-1})	k_{obs} (s^{-1})
	1.01 x 10^{-5}	–
	1.49 x 10^{-5}	257
9.80 x 10^{-7}	2.01 x 10^{-5}	347
	2.49 x 10^{-5}	394
	3.02 x 10^{-5}	477

k_2 = 1.40 x 10^7 L mol^{-1} s^{-1}

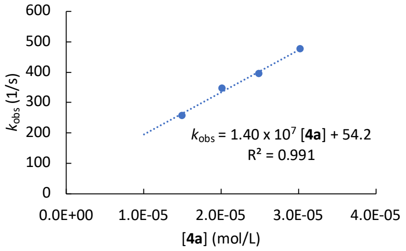

Determination of *N* and s_N parameter for **4a** in DCM

Electrophile	*E*	k_2 (M^{-1} s^{-1})
8a	-10.04	9.24 x 10^6
8b	-9.45	1.40 x 10^7
8c	-8.76	

***N* = 18.05** **s_N = 0.87[a]** (assumption based on **5a**)

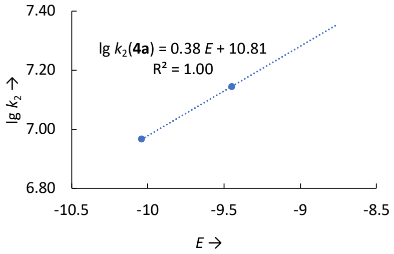

Nucleophilicity of ion pair 5a in DCM at 20 °C

Reaction of **5a** with $(lil)_2CH^+BF_4^-$ (stopped-flow, λ = 640 nm)

[8a] (mol L^{-1})	[5a] (mol L^{-1})	k_{obs} (s^{-1})
	9.96×10^{-6}	–
	1.47×10^{-5}	111
9.87×10^{-7}	1.99×10^{-5}	–
	2.47×10^{-5}	250
	2.99×10^{-5}	310

$k_2 = 1.32 \times 10^7$ L mol^{-1} s^{-1}

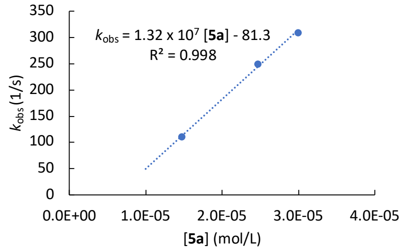

Reaction of **5a** with $(jul)_2CH^+BF_4^-$ (stopped-flow, λ = 643 nm)

[8b] (mol L^{-1})	[5a] (mol L^{-1})	k_{obs} (s^{-1})
	9.96×10^{-6}	—
	1.47×10^{-5}	195
9.80×10^{-7}	1.99×10^{-5}	
	2.47×10^{-5}	432
	2.99×10^{-5}	564

$k_2 = 2.42 \times 10^7$ L mol^{-1} s^{-1}

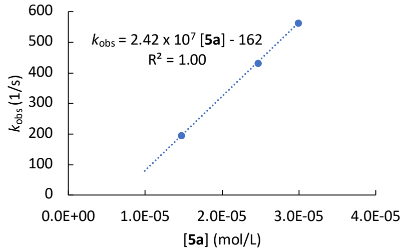

Reaction of **5a** with $(ind)_2CH^+BF_4^-$ (stopped-flow, λ = 626 nm)

[8c] (mol L^{-1})	[5a] (mol L^{-1})	k_{obs} (s^{-1})
	9.96×10^{-6}	186
	1.47×10^{-5}	969
1.01×10^{-6}	1.99×10^{-5}	–
	2.47×10^{-5}	–
	2.99×10^{-5}	–

$k_2 = 1.65 \times 10^8$ L mol^{-1} s^{-1}

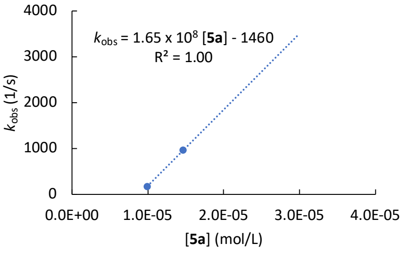

Determination of N and s_N parameter for **5a** in DCM

Electrophile	E	k_2 (M^{-1} s^{-1})
8a	-10.04	1.32×10^7
8b	-9.45	2.42×10^7
8c	-8.76	1.65×10^8

N = 18.05 **s_N = 0.84**

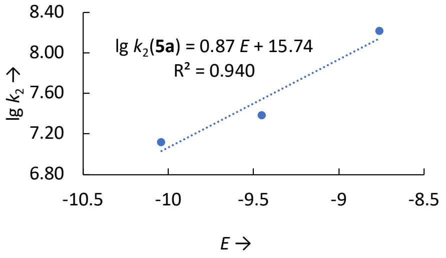

Nucleophilicity of ion pair 6a in DCM at 20 °C

Reaction of **6a** with $(lil)_2CH^+BF_4^-$ (stopped-flow, λ = 640 nm)

[8a] (mol L^{-1})	[6a] (mol L^{-1})	k_{obs} (s^{-1})
	9.93 x 10^{-6}	–
	1.49 x 10^{-5}	54.2
9.00 x 10^{-7}	1.99 x 10^{-5}	82.7
	2.48 x 10^{-5}	99.1
	2.98 x 10^{-5}	126

k_2 = 4.68 x 10^6 L mol^{-1} s^{-1}

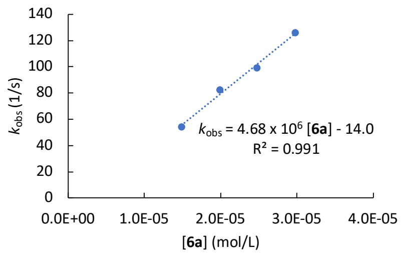

Reaction of **6a** with $(jul)_2CH^+BF_4^-$ (stopped-flow, λ = 643 nm)

[8b] (mol L^{-1})	[6a] (mol L^{-1})	k_{obs} (s^{-1})
	9.93 x 10^{-6}	–
	1.49 x 10^{-5}	106
9.80 x 10^{-7}	1.99 x 10^{-5}	150
	2.48 x 10^{-5}	189
	2.98 x 10^{-5}	232

k_2 = 8.41 x 10^6 L mol^{-1} s^{-1}

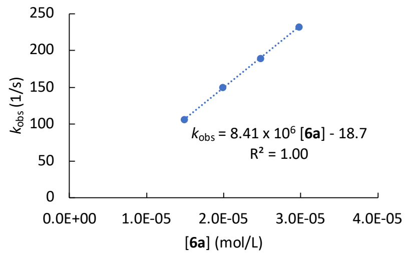

Reaction of **6a** with $(ind)_2CH^+BF_4^-$ (stopped-flow, λ = 626 nm)

[8c] (mol L^{-1})	[6a] (mol L^{-1})	k_{obs} (s^{-1})
	9.93 x 10^{-6}	133
	1.49 x 10^{-5}	346
9.00 x 10^{-7}	1.99 x 10^{-5}	486
	2.48 x 10^{-5}	629
	2.98 x 10^{-5}	–

k_2 = 3.28 x 10^7 L mol^{-1} s^{-1}

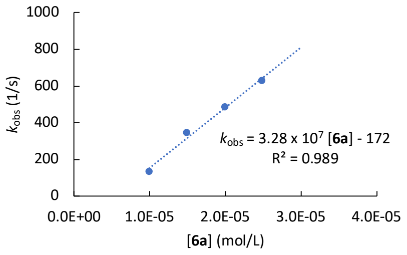

Determination of N and s_N parameter for **6a** in DCM

Electrophile	E	k_2 (M^{-1} s^{-1})
8a	-10.04	4.66 x 10^6
8b	-9.45	8.41 x 10^6
8c	-8.76	3.28 x 10^7

***N* = 19.87** ***s_N* = 0.67**

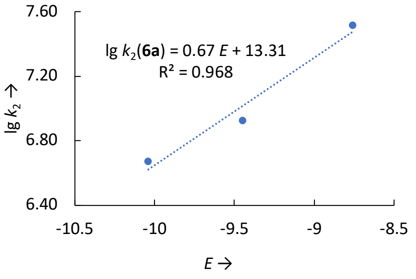

5.4 Nucleophilicity data in DCM at constant ionic strength *I* = 1.0 mM

Kinetic data of ion pair 3b in DCM at 20 °C

Association constants for numerical simulations: K_{CAC}(**3b**) = 4.65 x 10^6 M^{-2}, K_{CAC}(**7c**) = 4.61 x 10^6 M^{-2}.

Reaction of **3b + 7b** with $(lil)_2CH^+BF_4^-$ (stopped-flow, λ = 640 nm)

[8a] (mol L^{-1})	$[3b]_{tot}$ (mol L^{-1})	[C] (mol L^{-1})	$[3]^{HC,sw}$ (mol L^{-1})	$[3]^{HC,mix}$ (mol L^{-1})	k_{obs} (s^{-1})
	4.02 x 10^{-5}	9.57 x 10^{-4}	2.67 x 10^{-5}	–	15.0
	8.04 x 10^{-5}	9.18 x 10^{-4}	5.34 x 10^{-5}	–	28.8
4.04 x 10^{-6}	1.21 x 10^{-4}	8.79 x 10^{-4}	8.04 x 10^{-5}	–	43.2
	1.61 x 10^{-4}	8.41 x 10^{-4}	1.07 x 10^{-4}	–	55.1
	2.01 x 10^{-4}	8.02 x 10^{-4}	1.34 x 10^{-4}	–	67.8

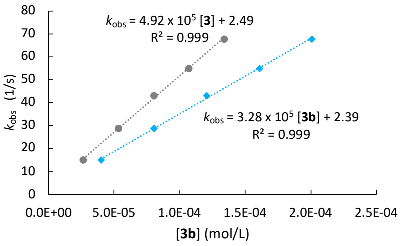

k_2^{HC} **= 3.28 x 10^5 L mol^{-1} s^{-1}** for $[\mathbf{3b}]_{tot}$

$k_2^{HC,sw}$ **= 4.92 x 10^5 L mol^{-1} s^{-1}** for [**3**]

Reaction of **3b + 7b** with $(jul)_2CH^+BF_4^-$ (stopped-flow, λ = 643 nm)

[8b] (mol L^{-1})	$[3b]_{tot}$ (mol L^{-1})	[C] (mol L^{-1})	$[3]^{HC,sw}$ (mol L^{-1})	$[3]^{HC,mix}$ (mol L^{-1})	k_{obs} (s^{-1})
	4.02 x 10^{-5}	9.57 x 10^{-4}	2.67 x 10^{-5}	–	31.5
	8.04 x 10^{-5}	9.18 x 10^{-4}	5.34 x 10^{-5}	–	60.5
4.03 x 10^{-6}	1.21 x 10^{-4}	8.79 x 10^{-4}	8.04 x 10^{-5}	–	95.6
	1.61 x 10^{-4}	8.41 x 10^{-4}	1.07 x 10^{-4}	–	133
	2.01 x 10^{-4}	8.02 x 10^{-4}	1.34 x 10^{-4}	–	170

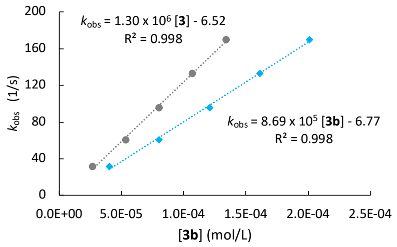

k_2^{HC} **= 8.69 x 10^5 L mol^{-1} s^{-1}** for $[\mathbf{3b}]_{tot}$

$k_2^{HC,sw}$ **= 1.30 x 10^6 L mol^{-1} s^{-1}** for [**3**]

Reaction of **3b + 7b** with $(jul)_2CH^+BF_4^-$ (stopped-flow, λ = 643 nm)

[8c] (mol L^{-1})	$[3b]_{tot}$ (mol L^{-1})	[C] (mol L^{-1})	$[3]^{HC,sw}$ (mol L^{-1})	$[3]^{HC,mix}$ (mol L^{-1})	k_{obs} (s^{-1})
	4.02 x 10^{-5}	9.57 x 10^{-4}	2.67 x 10^{-5}	–	88.7
	8.04 x 10^{-5}	9.18 x 10^{-4}	5.34 x 10^{-5}	–	199
3.71 x 10^{-6}	1.21 x 10^{-4}	8.79 x 10^{-4}	8.04 x 10^{-5}	–	318
	1.61 x 10^{-4}	8.41 x 10^{-4}	1.07 x 10^{-4}	–	445
	2.01 x 10^{-4}	8.02 x 10^{-4}	1.34 x 10^{-4}	–	570

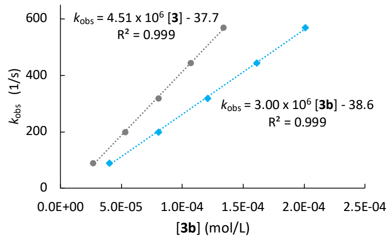

k_2^{HC} **= 3.00 x 10^6 L mol^{-1} s^{-1}** for $[\mathbf{3b}]_{tot}$

$k_2^{HC,sw}$ **= 4.51 x 10^6 L mol^{-1} s^{-1}** for [**3**]

Electrophile	*E*	k_2^{HC} (M^{-1} s^{-1}) for $[3b]_{tot}$	$k_2^{HC,sw}$ (M^{-1} s^{-1}) for [3]	$k_2^{HC,mix}$ (M^{-1} s^{-1}) for [3]
8a	-10.04	3.28 x 10^5	4.92 x 10^5	–
8b	-9.45	8.69 x 10^5	1.30 x 10^6	–
8c	-8.76	3.00 x 10^6	4.51 x 10^6	–

***N* = 17.41** $\quad$ s_N **= 0.75** for $[\mathbf{3b}]_{tot}$

***N* = 17.65** $\quad$ s_N **= 0.75** for $[\mathbf{3}]^{HC,sw}$

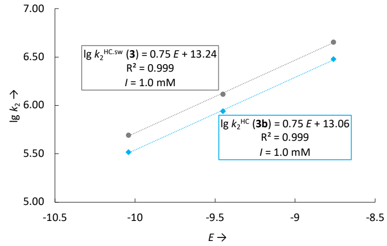

Data of ion pair 3c in DCM at 20 °C

Association constants for numerical simulations: K_{CAC}(**3c**) = 1.01 x 10^7 M^{-2}, K_{CAC}(**7c**) = 3.51 x 10^7 M^{-2},α x K_{CAC} = 3.33 x 10^6 M^{-2}, and β x K_{ACA} = 2.32 x 10^6 M^{-2}.

Reaction of **3c + 7c** with (lil)$_2$CH$^+$BF$_4^-$ (stopped-flow, λ = 640 nm)

**[8a] (mol L^{-1})**	**[3c]$_{tot}$ (mol L^{-1})**	**[7c] (mol L^{-1})**	**[3]^{HC,sw} (mol L^{-1})**	**[3]^{HC,mix} (mol L^{-1})**	**k_{obs} (s^{-1})**
	3.96 x 10^{-5}	9.550 x 10^{-}	1.80 x 10^{-5}	3.60 x 10^{-5}	10.9
	7.91 x 10^{-5}	9.160 x 10^{-}	3.70 x 10^{-5}	7.01 x 10^{-5}	23.4
3.90 x 10^{-6}	1.19 x 10^{-}	8.760 x 10^{-}	5.74 x 10^{-5}	1.03 x 10^{-}	34.2
	1.58 x 10^{-}	8.360 x 10^{-}	7.80 x 10^{-5}	1.33 x 10^{-}	45.1
	1.98 x 10^{-}	7.960 x 10^{-}	1.00 x 10^{-}	1.63 x 10^{-}	53.5

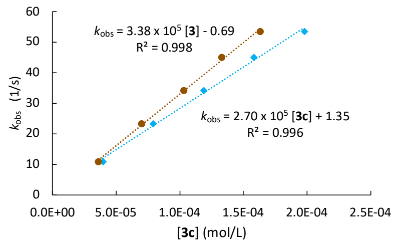

k_2^{HC} = 2.70 x 10^5 L mol^{-1} s^{-1} for [**3c**]$_{tot}$

$k_2^{HC,sw}$ = 3.41 x 10^5 L mol^{-1} s^{-1} for [**3**]

$k_2^{HC,mix}$ = 3.38 x 10^5 L mol^{-1} s^{-1} for [**3**]

Reaction of **3c + 7c** with (jul)$_2$CH$^+$BF$_4^-$ (stopped-flow, λ = 643 nm)

**[8b] (mol L^{-1})**	**[3c]$_{tot}$ (mol L^{-1})**	**[7c] (mol L^{-1})**	**[3]^{HC,sw} (mol L^{-1})**	**[3]^{HC,mix} (mol L^{-1})**	**k_{obs} (s^{-1})**
	3.96 x 10^{-5}	9.550 x 10^{-}	1.80 x 10^{-5}	3.60 x 10^{-5}	24.7
	7.91 x 10^{-5}	9.160 x 10^{-}	3.70 x 10^{-5}	7.01 x 10^{-5}	49.1
3.92 x 10^{-6}	1.19 x 10^{-}	8.760 x 10^{-}	5.74 x 10^{-5}	1.03 x 10^{-}	74.0
	1.58 x 10^{-}	8.360 x 10^{-}	7.80 x 10^{-5}	1.33 x 10^{-}	98.8
	1.98 x 10^{-}	7.960 x 10^{-}	1.00 x 10^{-}	1.63 x 10^{-}	132

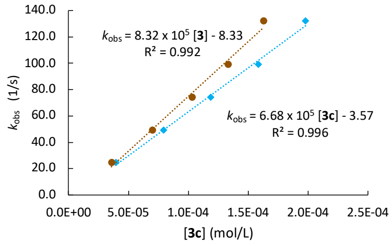

k_2^{HC} = 6.68 x 10^5 L mol^{-1} s^{-1} for [**3c**]$_{tot}$

$k_2^{HC,sw}$ = 8.42 x 10^5 L mol^{-1} s^{-1} for [**3**]

$k_2^{HC,mix}$ = 8.32 x 10^5 L mol^{-1} s^{-1} for [**3**]

Reaction of **3c + 7c** with (jul)$_2$CH$^+$BF$_4^-$ (stopped-flow, λ = 643 nm)

**[8c] (mol L^{-1})**	**[3c]$_{tot}$ (mol L^{-1})**	**[7c] (mol L^{-1})**	**[3]^{HC,sw} (mol L^{-1})**	**[3]^{HC,mix} (mol L^{-1})**	**k_{obs} (s^{-1})**
	3.96 x 10^{-5}	9.550 x 10^{-}	1.80 x 10^{-5}	3.60 x 10^{-5}	79.2
	7.91 x 10^{-5}	9.160 x 10^{-}	3.70 x 10^{-5}	7.01 x 10^{-5}	180
3.97 x 10^{-6}	1.19 x 10^{-}	8.760 x 10^{-}	5.74 x 10^{-5}	1.03 x 10^{-}	278
	1.58 x 10^{-}	8.360 x 10^{-}	7.80 x 10^{-5}	1.33 x 10^{-}	375
	1.98 x 10^{-}	7.960 x 10^{-}	1.00 x 10^{-}	1.63 x 10^{-}	485

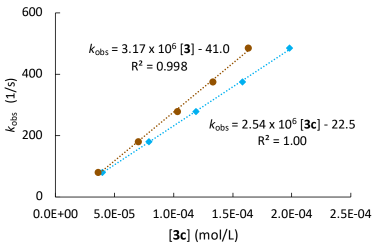

k_2^{HC} = 2.45 x 10^6 L mol^{-1} s^{-1} for [**3c**]$_{tot}$

$k_2^{HC,sw}$ = 3.21 x 10^6 L mol^{-1} s^{-1} for [**3**]

$k_2^{HC,mix}$ = 3.17 x 10^6 L mol^{-1} s^{-1} for [**3**]

**Electrophile**	***E***	**k_2^{HC} (M^{-1} s^{-1})** for [**3c**]$_{tot}$	**$k_2^{HC,sw}$ (M^{-1} s^{-1})** for [**3**]	**$k_2^{HC,mix}$ (M^{-1} s^{-1})** for [**3**]
8a	-10.04	2.70 x 10^5	3.41 x 10^5	3.38 x 10^5
8b	-9.45	6.68 x 10^5	8.42 x 10^5	8.32 x 10^5
8c	-8.76	2.45 x 10^6	3.21 x 10^6	3.17 x 10^6

***N* = 17.20** ***s$_N$* = 0.76** for [**3c**]$_{tot}$

***N* = 17.33** ***s*$_N$ = 0.76** for [3]^{HC,sw}

***N* = 17.32** ***s*$_N$ = 0.76** for [3]^{HC,mix}

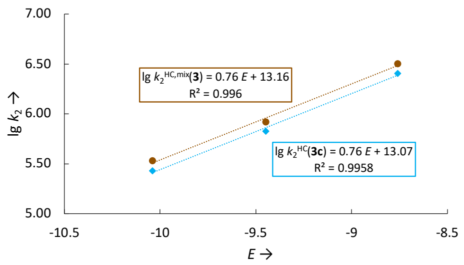

Kinetic data of ion pair 3d in DCM at 20 °C

Association constants for numerical simulations: K_{CAC}(**3d**) = 1.07 x 10^7 M^{-2}, K_{CAC}(**7d**) = 3.74 x 10^7 M^{-2}.

Reaction of **3d + 7d** with (lil)$_2$CH$^+$BF$_4^-$ (stopped-flow, λ = 640 nm)

[8a] (mol L^{-1})	[3d]$_{tot}$ (mol L^{-1})	[7d] (mol L^{-1})	[3]^{HC,sw} (mol L^{-1})	[3]^{HC,mix} (mol L^{-1})	k_{obs} (s^{-1})
	4.00 x 10^{-5}	9.51 x 10^{-4}	3.24 x 10^{-5}	–	12.0
	7.79 x 10^{-5}	9.21 x 10^{-4}	6.26 x 10^{-5}	–	25.0
4.03 x 10^{-6}	1.20 x 10^{-4}	8.77 x 10^{-4}	9.58 x 10^{-5}	–	36.6
	1.60 x 10^{-4}	8.32 x 10^{-4}	1.27 x 10^{-4}	–	45.8
	2.00 x 10^{-4}	8.02 x 10^{-4}	1.57 x 10^{-4}	–	57.6

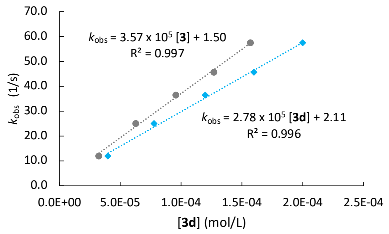

k_2^{HC} = 2.78 x 10^5 L mol^{-1} s^{-1} for [**3d**]$_{tot}$

$k_2^{HC,sw}$ = 3.57 x 10^5 L mol^{-1} s^{-1} for [**3**]

Reaction of **3d + 7d** with (jul)$_2$CH$^+$BF$_4^-$ (stopped-flow, λ = 643 nm)

[8b] (mol L^{-1})	[3d]$_{tot}$ (mol L^{-1})	[7d] (mol L^{-1})	[3]^{HC,sw} (mol L^{-1})	[3]^{HC,mix} (mol L^{-1})	k_{obs} (s^{-1})
	4.00 x 10^{-5}	9.51 x 10^{-4}	3.24 x 10^{-5}	–	24.8
	7.79 x 10^{-5}	9.21 x 10^{-4}	6.26 x 10^{-5}	–	50.0
4.02 x 10^{-6}	1.20 x 10^{-4}	8.77 x 10^{-4}	9.58 x 10^{-5}	–	76.7
	1.60 x 10^{-4}	8.32 x 10^{-4}	1.27 x 10^{-4}	–	108
	2.00 x 10^{-4}	8.02 x 10^{-4}	1.57 x 10^{-4}	–	138

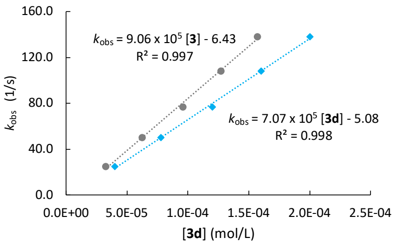

k_2^{HC} = 7.07 x 10^5 L mol^{-1} s^{-1} for [**3d**]$_{tot}$

$k_2^{HC,sw}$ = 9.06 x 10^5 L mol^{-1} s^{-1} for [**3**]

Reaction of **3d + 7d** with (jul)$_2$CH$^+$BF$_4^-$ (stopped-flow, λ = 643 nm)

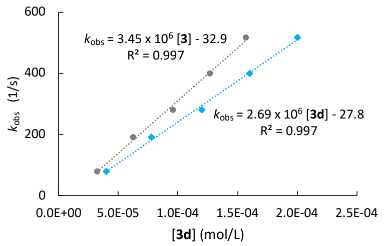

[8c] (mol L^{-1})	[3d]$_{tot}$ (mol L^{-1})	[7d] (mol L^{-1})	[3]^{HC,sw} (mol L^{-1})	[3]^{HC,mix} (mol L^{-1})	k_{obs} (s^{-1})
	4.00 x 10^{-5}	9.51 x 10^{-4}	3.24 x 10^{-5}	–	80.2
	7.79 x 10^{-5}	9.21 x 10^{-4}	6.26 x 10^{-5}	–	191
4.14 x 10^{-6}	1.20 x 10^{-4}	8.77 x 10^{-4}	9.58 x 10^{-5}	–	282
	1.60 x 10^{-4}	8.32 x 10^{-4}	1.27 x 10^{-4}	–	400
	2.00 x 10^{-4}	8.02 x 10^{-4}	1.57 x 10^{-4}	–	517

k_2^{HC} = 2.69 x 10^6 L mol^{-1} s^{-1} for [**3d**]$_{tot}$

$k_2^{HC,sw}$ = 3.45 x 10^6 L mol^{-1} s^{-1} for [**3**]

Electrophile	*E*	k_2^{HC} (M^{-1} s^{-1}) for [3d]$_{tot}$	$k_2^{HC,sw}$ (M^{-1} s^{-1}) for [3]	$k_2^{HC,mix}$ (M^{-1} s^{-1}) for [3]
8a	-10.04	2.78 x 10^5	3.57 x 10^5	–
8b	-9.45	7.07 x 10^5	9.06 x 10^5	–

8c	-8.76	2.69 x 10^6	3.45 x 10^6	–

***N* = 17.12** $\quad$ ***s*$_N$ = 0.77** for **[3d]**$_{tot}$

***N* = 17.25** $\quad$ ***s*$_N$ = 0.77** for **[3]**^{HC,sw}

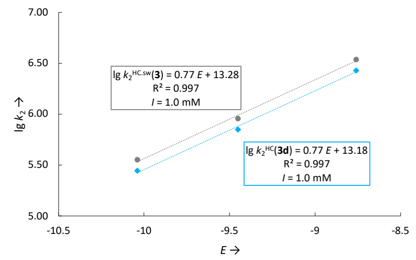

Kinetic data of ion pair 5a in DCM at 20 °C

Association constants for numerical simulations: K_{CAC}(**5a**) = 5.15 x 10^6 M^{-2}, K_{CAC}(**7a**) = 7.05 x 10^6 M^{-2},α x K_{CAC} = 5.67 x 10^5 M^{-2}, and β x K_{ACA} = 3.45 x 10^6 M^{-2}.

Reaction of **5a + 7a** with (lil)$_2$CH$^+$BF$_4^-$ (stopped-flow, λ = 640 nm)

[8a] (mol L^{-1})	[5a]$_{tot}$ (mol L^{-1})	[7a] (mol L^{-1})	[5]^{HC,sw} (mol L^{-1})	[5]^{HC,mix} (mol L^{-1})	k_{obs} (s^{-1})
	4.02 x 10^{-5}	9.59 x 10^{-4}	2.85 x 10^{-5}	3.60 x 10^{-5}	35.2
	6.02 x 10^{-5}	9.45 x 10^{-4}	4.25 x 10^{-5}	5.22 x 10^{-5}	55.3
4.00 x 10^{-6}	8.03 x 10^{-5}	9.18 x 10^{-4}	5.68 x 10^{-5}	6.77 x 10^{-5}	80.5
	1.00 x 10^{-4}	9.04 x 10^{-4}	7.07 x 10^{-5}	8.22 x 10^{-5}	113
	1.20 x 10^{-4}	8.76 x 10^{-4}	8.46 x 10^{-5}	9.61 x 10^{-5}	141

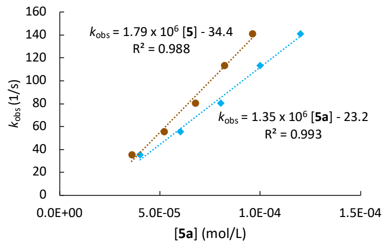

$\boldsymbol{k_2^{HC}}$ **= 1.35 x 10^6 L mol^{-1} s^{-1}** for **[5a]**$_{tot}$

$\boldsymbol{k_2^{HC,sw}}$ **= 1.92 x 10^6 L mol^{-1} s^{-1}** for [**5**]

$\boldsymbol{k_2^{HC,mix}}$ **= 1.79 x 10^6 L mol^{-1} s^{-1}** for [**5**]

Reaction of **5a + 7a** with (jul)$_2$CH$^+$BF$_4^-$ (stopped-flow, λ = 643 nm)

[8b] (mol L^{-1})	[5a]$_{tot}$ (mol L^{-1})	[7a] (mol L^{-1})	[5]^{HC,sw} (mol L^{-1})	[5]^{HC,mix} (mol L^{-1})	k_{obs} (s^{-1})
	4.02 x 10^{-5}	9.59 x 10^{-4}	2.85 x 10^{-5}	3.60 x 10^{-5}	74
	6.02 x 10^{-5}	9.45 x 10^{-4}	4.25 x 10^{-5}	5.22 x 10^{-5}	137
3.82 x 10^{-6}	8.03 x 10^{-5}	9.18 x 10^{-4}	5.68 x 10^{-5}	6.77 x 10^{-5}	196
	1.00 x 10^{-4}	9.04 x 10^{-4}	7.07 x 10^{-5}	8.22 x 10^{-5}	251
	1.20 x 10^{-4}	8.76 x 10^{-4}	8.46 x 10^{-5}	9.61 x 10^{-5}	317

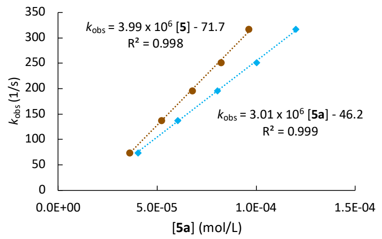

$\boldsymbol{k_2^{HC}}$ **= 3.01 x 10^6 L mol^{-1} s^{-1}** for **[5a]**$_{tot}$

$\boldsymbol{k_2^{HC,sw}}$ **= 4.27 x 10^6 L mol^{-1} s^{-1}** for [**5**]

$\boldsymbol{k_2^{HC,mix}}$ **= 3.99 x 10^6 L mol^{-1} s^{-1}** for [**5**]

Reaction of **5a + 7a** with (jul)$_2$CH$^+$BF$_4^-$ (stopped-flow, λ = 643 nm)

[8c] (mol L^{-1})	[5a]$_{tot}$ (mol L^{-1})	[7a] (mol L^{-1})	[5]^{HC,sw} (mol L^{-1})	[5]^{HC,mix} (mol L^{-1})	k_{obs} (s^{-1})
	4.02 x 10^{-5}	9.59 x 10^{-4}	2.85 x 10^{-5}	3.60 x 10^{-5}	270
	6.02 x 10^{-5}	9.45 x 10^{-4}	4.25 x 10^{-5}	5.22 x 10^{-5}	430
4.08 x 10^{-6}	8.03 x 10^{-5}	9.18 x 10^{-4}	5.68 x 10^{-5}	6.77 x 10^{-5}	608
	1.00 x 10^{-4}	9.04 x 10^{-4}	7.07 x 10^{-5}	8.22 x 10^{-5}	842
	1.20 x 10^{-4}	8.76 x 10^{-4}	8.46 x 10^{-5}	9.61 x 10^{-5}	989

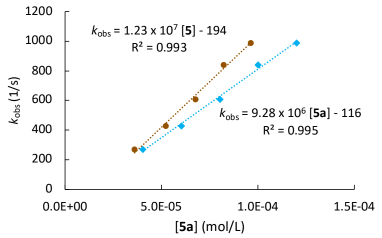

$\boldsymbol{k_2^{HC}}$ **= 9.28 x 10^6 L mol^{-1} s^{-1}** for **[5a]**$_{tot}$

$k_2^{HC,sw}$ **= 1.32 x 10^7 L mol^-1 s^-1** for [**5**]

$k_2^{HC,mix}$ **= 1.23 x 10^7 L mol^-1 s^-1** for [**5**]

Electrophile	*E*	k_2^{HC} (M^{-1} s^{-1}) for [5a]$_{tot}$	$k_2^{HC,sw}$ (M^{-1} s^{-1}) for [5]	$k_2^{HC,mix}$ (M^{-1} s^{-1}) for [5]
8a	-10.04	1.35 x 10^6	1.92 x 10^6	1.79 x 10^6
8b	-9.45	3.01 x 10^6	4.27 x 10^6	3.99 x 10^6
8c	-8.76	9.28 x 10^6	1.32 x 10^7	1.23 x 10^7

***N* = 19.24** s_N **= 0.66** for [**5a**]$_{tot}$

***N* = 19.47** s_N **= 0.66** for [**5**]^{HC,sw}

***N* = 19.42** s_N **= 0.66** for [**5**]^{HC,mix}

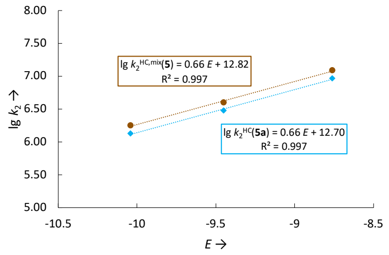

Kinetic data of ion pair 6a in DCM at 20 °C

Association constants for numerical simulations: K_{CAC}(**6a**) = 6.75 x 10^6 M^{-2}, K_{CAC}(**7a**) = 7.05 x 10^6 M^{-2},α x K_{CAC} = 1.08 x 10^6 M^{-2}, and β x K_{ACA} = 3.51 x 10^6 M^{-2}.

Reaction of **6a + 7a** with $(lil)_2CH^+BF_4^-$ (stopped-flow, λ = 640 nm)

[8a] (mol L^{-1})	[6a]$_{tot}$ (mol L^{-1})	[7a] (mol L^{-1})	[6]^{HC,sw} (mol L^{-1})	[6]^{HC,mix} (mol L^{-1})	k_{obs} (s^{-1})
	3.98 x 10^{-5}	9.59 x 10^{-4}	2.60 x 10^{-5}	3.44 x 10^{-5}	27.9
	5.97 x 10^{-5}	9.44 x 10^{-4}	3.90 x 10^{-5}	5.01 x 10^{-5}	44.4
3.82 x 10^{-6}	7.96 x 10^{-5}	9.16 x 10^{-4}	5.19 x 10^{-5}	6.50 x 10^{-5}	57.9
	9.95 x 10^{-5}	9.02 x 10^{-4}	6.48 x 10^{-5}	7.92 x 10^{-5}	75.5
	1.19 x 10^{-4}	8.74 x 10^{-4}	7.76 x 10^{-5}	9.25 x 10^{-5}	95.1

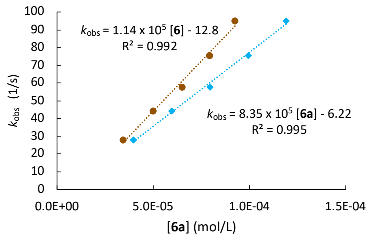

k_2^{HC} **= 8.35 x 10^5 L mol^-1 s^-1** for [**6a**]$_{tot}$

$k_2^{HC,sw}$ **= 1.28 x 10^6 L mol^-1 s^-1** for [**6**]

$k_2^{HC,mix}$ **= 1.14 x 10^6 L mol^-1 s^-1** for [**6**]

Reaction of **6a + 7a** with $(jul)_2CH^+BF_4^-$ (stopped-flow, λ = 643 nm)

[8b] (mol L^{-1})	[6a]$_{tot}$ (mol L^{-1})	[7a] (mol L^{-1})	[6]^{HC,sw} (mol L^{-1})	[6]^{HC,mix} (mol L^{-1})	k_{obs} (s^{-1})
	3.98 x 10^{-5}	9.59 x 10^{-4}	2.60 x 10^{-5}	3.44 x 10^{-5}	58.3
	5.97 x 10^{-5}	9.44 x 10^{-4}	3.90 x 10^{-5}	5.01 x 10^{-5}	102
4.04 x 10^{-6}	7.96 x 10^{-5}	9.16 x 10^{-4}	5.19 x 10^{-5}	6.50 x 10^{-5}	153
	9.95 x 10^{-5}	9.02 x 10^{-4}	6.48 x 10^{-5}	7.92 x 10^{-5}	193
	1.19 x 10^{-4}	8.74 x 10^{-4}	7.76 x 10^{-5}	9.25 x 10^{-5}	237

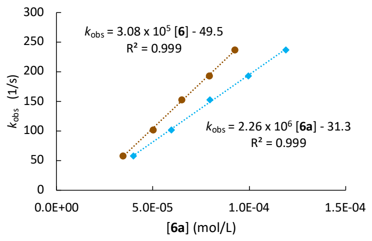

k_2^{HC} **= 2.26 x 10^6 L mol^-1 s^-1** for [**6a**]$_{tot}$

$k_2^{HC,sw}$ **= 3.48 x 10^6 L mol^-1 s^-1** for [6]

$k_2^{HC,mix}$ **= 3.08 x 10^6 L mol^-1 s^-1** for [6]

Reaction of **6a + 7a** with $(jul)_2CH^+BF_4^-$ (stopped-flow, λ = 643 nm)

[8c]	[6a]$_{tot}$	[7a]	[6]^{HC,sw}	[6]^{HC,mix}	k_{obs} (s^{-1})

(mol L^{-1})	(mol L^{-1})	(mol L^{-1})	(mol L^{-1})	(mol L^{-1})	
	3.98 x 10^{-5}	9.59 x 10^{-4}	2.60 x 10^{-5}	3.44 x 10^{-5}	198
	5.97 x 10^{-5}	9.44 x 10^{-4}	3.90 x 10^{-5}	5.01 x 10^{-5}	350
4.02 x 10^{-6}	7.96 x 10^{-5}	9.16 x 10^{-4}	5.19 x 10^{-5}	6.50 x 10^{-5}	514
	9.95 x 10^{-5}	9.02 x 10^{-4}	6.48 x 10^{-5}	7.92 x 10^{-5}	626
	1.19 x 10^{-4}	8.74 x 10^{-4}	7.76 x 10^{-5}	9.25 x 10^{-5}	748

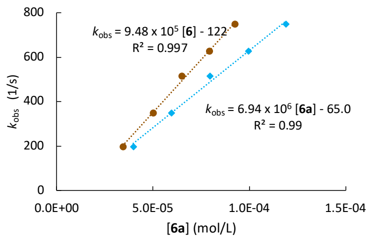

k_2^{HC} = **6.94 x 10^6 L mol^{-1} s^{-1}** for [**6a**]$_{tot}$

$k_2^{HC,sw}$ = **1.07 x 10^7 L mol^{-1} s^{-1}** for [**6**]

$k_2^{HC,mix}$ = **9.48 x 10^6 L mol^{-1} s^{-1}** for [**6**]

Electrophile	*E*	k_2^{HC} (M^{-1} s^{-1}) for [**6a**]$_{tot}$	$k_2^{HC,sw}$ (M^{-1} s^{-1}) for [**6**]	$k_2^{HC,mix}$ (M^{-1} s^{-1}) for [**6**]
8a	-10.04	8.35 x 10^5	1.28 x 10^6	1.14 x 10^6
8b	-9.45	2.26 x 10^6	3.48 x 10^6	3.08 x 10^6
8c	-8.76	6.94 x 10^6	1.07 x 10^7	9.48 x 10^6

***N* = 18.25**   ***s$_N$* = 0.72** for [**6a**]$_{tot}$

***N* = 18.53**   ***s$_N$* = 0.72** for [**6**]^{HC,sw}

***N* = 18.43**   ***s$_N$* = 0.72** for [**6**]^{HC,mix}

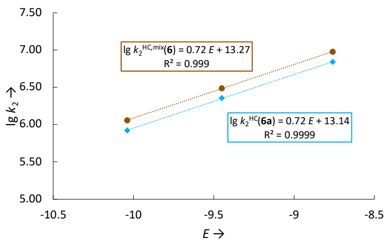

5.5 Effects of Ion Association on Nucleophilicity

The so-called wide range measurements employ the same principle as the benzhydrylium kinetics except for covering a much larger concentration range (1 x 10^{-5} to 4 x 10^{-3}). The aim of these measurement to illustrate the influence of ion pair formation with increasing concentration on the first-order reaction rate k_{obs} (s^{-1}) and obtained second order rate constants k_2 (M^{-1} s^{-1}) are generally not included in the N and s_N parameter determination. The benzhydrylium salt $(lil)_2CH^+BF_4^-$ (**8a**) was used as electrophile at λ = 640 nm.

Measurement of 3b at 20°C

Reaction of **3b** with $(lil)_2CH^+BF_4^-$ in DCM (stopped-flow, λ = 640 nm)

[8a] (mol L^{-1})	[3b] (mol L^{-1})	k_{obs} (s^{-1})
1.23 x 10^{-6}	1.00 x 10^{-5}	11.0
	2.00 x 10^{-5}	34.5
	4.01 x 10^{-5}	64.5
	6.21 x 10^{-5}	92.7
	8.01 x 10^{-5}	112
	1.00 x 10^{-4}	133
	2.00 x 10^{-4}	212
	4.01 x 10^{-4}	303
	6.01 x 10^{-4}	356
	8.01 x 10^{-4}	380
	1.00 x 10^{-3}	417
	2.00 x 10^{-3}	460
	4.01 x 10^{-3}	541

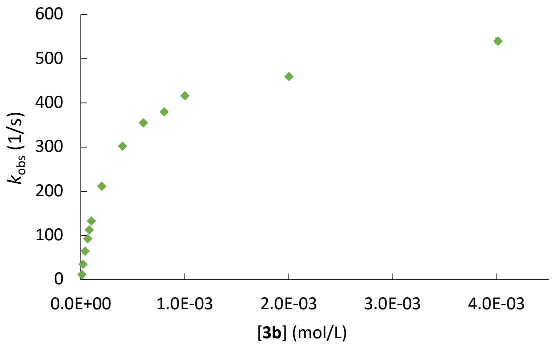

Measurement of 3c at 20°C

Reaction of **3c** with $(lil)_2CH^+BF_4^-$ in DCM (stopped-flow, λ = 640 nm)

[8a] (mol L^{-1})	[3c] (mol L^{-1})	k_{obs} (s^{-1})
1.06 x 10^{-6}	1.00 x 10^{-5}	15.0
	2.00 x 10^{-5}	28.9
	4.01 x 10^{-5}	52.1
	6.01 x 10^{-5}	71.9
	8.02 x 10^{-5}	88.1
	1.00 x 10^{-4}	100
	2.00 x 10^{-4}	142
	4.01 x 10^{-4}	196
	6.01 x 10^{-4}	238
	8.02 x 10^{-4}	266
	1.00 x 10^{-3}	294
	2.00 x 10^{-3}	367
	4.01 x 10^{-3}	469

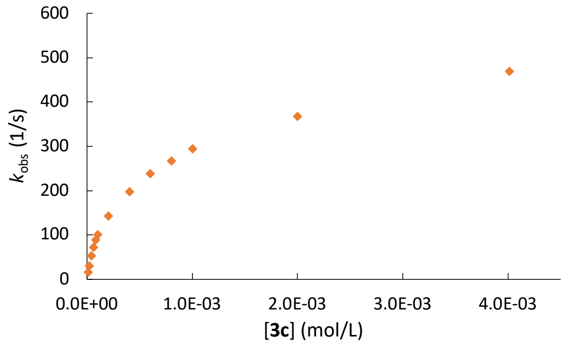

Measurement of 3d at 20°C

Reaction of **3d** with (lil)$_2$CH$^+$BF$_4^-$ in DCM (stopped-flow, λ = 640 nm)

[8a] (mol L^{-1})	[3d] (mol L^{-1})	k_{obs} (s^{-1})
1.03 x 10^{-6}	9.98 x 10^{-6}	14.0
	2.00 x 10^{-5}	31.1
	3.99 x 10^{-5}	50.3
	5.99 x 10^{-5}	72.6
	7.99 x 10^{-5}	86.1
	9.98 x 10^{-5}	98.2
	2.00 x 10^{-4}	149
	3.99 x 10^{-4}	188
	5.99 x 10^{-4}	242
	7.99 x 10^{-4}	269
	9.98 x 10^{-4}	294
	2.00 x 10^{-3}	379
	3.99 x 10^{-3}	475

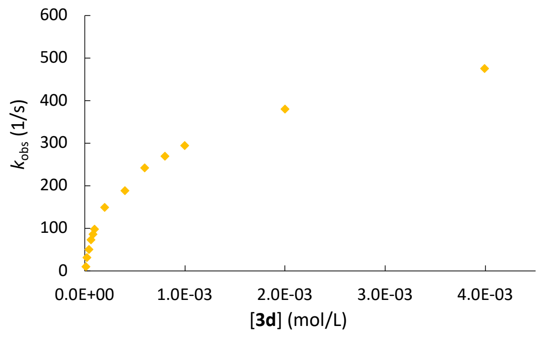

Reaction of **3d** with (lil)$_2$CH$^+$BF$_4^-$ in MeCN (stopped-flow, λ = 632 nm)

[8a] (mol L^{-1})	[3d] (mol L^{-1})	k_{obs} (s^{-1})
1.26 x 10^{-6}	9.91 x 10^{-6}	0.31
	1.98 x 10^{-5}	0.40
	3.96 x 10^{-5}	0.50
	5.94 x 10^{-5}	0.65
	7.92 x 10^{-5}	0.78
	9.91 x 10^{-5}	0.92
	1.98 x 10^{-4}	1.43
	3.96 x 10^{-4}	2.43
	5.94 x 10^{-4}	3.51
	7.92 x 10^{-4}	4.48
	9.91 x 10^{-4}	5.63
	1.98 x 10^{-3}	10.4
	3.96 x 10^{-3}	25.5

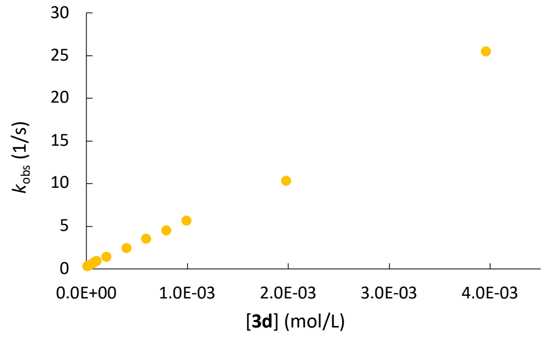

Measurement of 5a at 20°C

Reaction of **5a** with (lil)$_2$CH$^+$BF$_4^-$ in DCM (stopped-flow, λ = 640 nm)

[8a] (mol L^{-1})	[5a] (mol L^{-1})	k_{obs} (s^{-1})
9.74 x 10^{-7}	9.96 x 10^{-6}	49.5
	1.99 x 10^{-5}	205
	3.98 x 10^{-5}	354
	5.98 x 10^{-5}	502
	7.97 x 10^{-5}	672
	9.96 x 10^{-5}	828

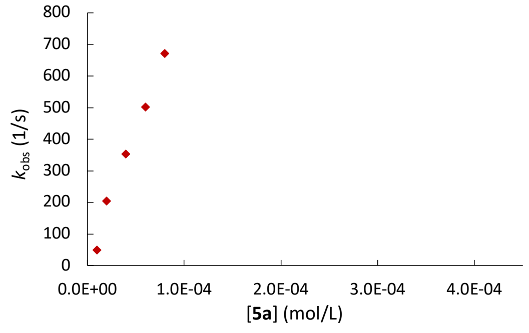

Measurement of 6a at 20°C

Reaction of **6a** with $(lil)_2CH^+BF_4^-$ in DCM (stopped-flow, λ = 640 nm)

[8a] (mol L^{-1})	[6a] (mol L^{-1})	k_{obs} (s^{-1})
1.04 x 10^{-6}	9.72 x 10^{-6}	20.7
	1.94 x 10^{-5}	71.6
	3.89 x 10^{-5}	173
	5.83 x 10^{-5}	228
	7.77 x 10^{-5}	291
	9.72 x 10^{-5}	358
	1.99 x 10^{-4}	535
	3.98 x 10^{-4}	680

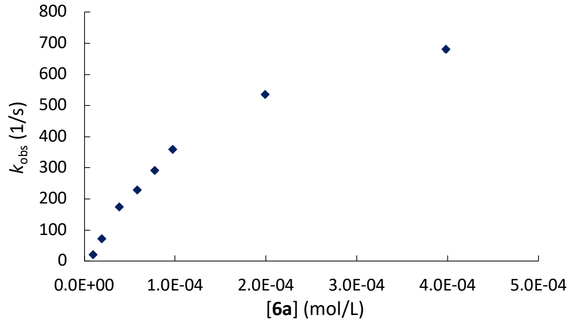

6. pK_a measurements

6.1 General Information – pK_a measurements in aqueous solutions

The neutral pyridinamides as well as the respective ion pairs were stored under nitrogen atmosphere. Hygroscopic salts were dried *in vacuo* for 12 h prior to being used in the buffer preparation. Pure water was used for the preparation of buffer solutions and stock solutions. For pipetting the buffer and stock solutions *Eppendorf* pipettes were used. All calibration solutions and the baseline solutions were tempered at 25 °C using a circulating water bath.

UV-Vis Spectra were recorded on a Varian Cary 100 Bio Spectrophotometer (version 9.00) maintained at 25 °C using the “scan software” (version 3.00(182)) and the “kinetics software” (version 3.00(182)). The scan software was used to record the full UV-Vis spectrum of the respective compound between 200 - 400 nm, while the kinetics software was used to validate the stability of the compound at a fixed wavelength, whereas the absorbance was measured every 0.1 min for 1.0 min. Before starting the measurement, the respective wavelength was selected, and the instrument was “zeroed” to air. The baseline function of the program was not used. Instead, a baseline spectrum would be measured for each data point where the solution had the same composition as the actual measurement but without the compound in it. The baseline spectrum was then subtracted from the measurement spectrum using Excel. The p*H* value of each baseline mixture was determined using a Radiometer Analytical RadioLab p*H* 210 standard p*H* meter connected to a radiometer electrode filled with saturated KCl solution. The electrode was stored in 3.0 M KCl solution in between measurements. Calibration of the setup was done at intervals of p*H* = 1.4 – 4.0, 4.0 – 7.0, and 7.0 – 10.0 shortly before measuring the respective baseline mixtures, which were tempered to 25 °C using a circulating water bath for at least 30 min prior to measuring.

For the aqueous pK_a measurements the neutral pyridinamides were dissolved in premixed MeCN/pure water mixtures (ratio = 50/50 of volume) and added to aqueous buffer solutions (c = 0.05 M or 0.1 M, *I* = 0.3 M) to give measurement solutions with a compound concentration of *c* = 0.05 mM. The absorbance of the neutral pyridinamide or respective anion was measured across the p*H* range and the resulting absorbance-p*H* plots were fitted using Origin. To obtain the necessary equation for the fitting, equations S15-19 are used, with eq. S16-18 being inserted into eq. S15 to give eq. S19 with K_a being the acid dissociation constant for the compound, p*H* is the one of the used buffers, A_{obs} being the observed absorbance, A_{max} the maximum absorbance (where the compound is either protonated or deprotonated) and A_{min} is the minimum absorbance (where the compound is either fully protonated or fully deprotonated).

When the chosen wavelength is following the neutral pyridinamide, e.g. 285 nm for pyridinamide **5**, the absorbance is increasing with the p*H* (p*H* < 6), thus A_{max} reflects the neutral pyridinamide (one proton at the pyridine nitrogen) and A_{min} the fully protonated pyridinamide form, which is depicted in eq. S17 and S18.

$$K_a = \frac{[H^+][A^-]}{[HA]} \qquad \text{S15}$$

$$[H^+] = 10^{-pH} \qquad \text{S16}$$

$$[A^-] = A_{obs} - A_{min} \qquad \text{S17}$$

$$[HA] = A_{max} - A_{obs} \qquad \text{S18}$$

$$K_a = \frac{10^{-pH}(A_{obs} - A_{min})}{(A_{max} - A_{obs})} \qquad \text{S19}$$

Increase in absorbance:

$$A_{obs} = \frac{10^{-pH} \times A_{min} + K_a \times A_{max}}{10^{-pH} \times K_a} \qquad \text{S20}$$

When further increasing p*H* (p*H* > 6) the absorbance of the neutral pyridinamide decreases due to deprotonation of the neutral pyridinamide towards the fully deprotonated anion. Here, A_{max} reflects the neutral

pyridinamide and A_{min} the fully deprotonated anion. Therefore, eq. S17 and S18 are rewritten into S21 and S22. Inserting those equations into eq. S15 and solving it for A_{obs} results in eq. S23 with which the decrease in absorbance can be fitted in Origin to obtain K_a. In Origin A_{max} and A_{min} are treated as variables.

$$[A^-] = A_{max} - A_{obs} \qquad \text{S21}$$

$$[HA] = A_{obs} - A_{min} \qquad \text{S22}$$

Decrease in absorbance:

$$A_{obs} = \frac{10^{-pH} \times A_{max} + K_a \times A_{min}}{K_a + 10^{-pH}} \qquad \text{S23}$$

Taking the logarithm of K_a (see eq. S24) gives the final value for pK_a.

$$pK_a = -log_{10} K_a \qquad \text{S24}$$

Preparation of buffer solutions

The buffer solutions are prepared according to the Henderson-Hasselbalch equation (see eq. S25). Several acid-base pairs with variating amounts of free base were chosen to obtain the wanted p*H* value in the final buffer solution (see Table S15).

$$pH = pK_a + log\frac{A^-}{HA} \qquad \text{S25}$$

The ionic strength *I* of the buffer solution is calculated according to equation S26 with c_i being the concentration of ions in solution and z_i^2 the charge of ions squared.

$$I = \frac{1}{2}\sum c_i z_i^2 \qquad \text{S26}$$

For comparable results, all buffer solutions must have to the same ionic strength *I* independent of the concentration and composition regarding the acid and base of the final buffer solutions.

All prepared buffer solutions for the aqueous p*H*-titration with their acid-base pairs, amount of free base (fb) and measured p*H* value are listed below. All buffer solutions are prepared to have a concentration of 0.1 M and an ionic strength *I* of 0.3 M which is achieved by addition of potassium chloride (KCl).

Table S15. List of prepared aqueous buffer solution with the involved acid and bases, the pK_a value of the respective acid, the amount of free base (fb) in percentage, the final buffer concentration, and the resulting p*H*.

No.	Buffer System	Acid	Base	pK_a of acid	%fb	Concentration (M)	p*H*
1	HCl	HCl	–	–	–	0.1	1.08
2	HCl	HCl	–	–	–	0.03	1.60
3	HCl	HCl	–	–	–	0.01	2.10
4	Formate	HCl	$KCHCO_2$	3.75	10	0.1	2.51
5		HCl	$KCHCO_2$		50	0.1	3.55
6	Acetate	HCl	$KMeCO_2$	4.76	30	0.1	4.17
7		HCl	$KMeCO_2$		70	0.1	4.94
8	Phosphate	KH_2PO_4	K_2HPO_4	6.86	10	0.1	5.68
9		KH_2PO_4	K_2HPO_4		50	0.1	6.72
10	Triethanolamine-HCl	TEOA-HCl	KOH	7.76	10	0.1	6.94
11		TEOA-HCl	KOH		20	0.05	7.37
12		TEOA-HCl	KOH		30	0.1	7.55
13		TEOA-HCl	KOH		40	0.05	7.81
14		TEOA-HCl	KOH		50	0.1	7.95
15		TEOA-HCl	KOH		70	0.05/0.1	8.37
16		TEOA-HCl	KOH		80	0.05	8.62
17		TEOA-HCl	KOH		90	0.1	8.96

18	Carbonate	$KHCO_3$	K_2CO_3	10.3	10	0.1	9.04
19		$KHCO_3$	K_2CO_3		25	0.1	9.46
20		$KHCO_3$	K_2CO_3		50	0.1	9.97
21	Piperidine-HCl	PIP-HCl	KOH	11.2	20	0.1	10.71
22		PIP-HCl	KOH		60	0.1	11.47

pK_a measurement data in aqueous solution

DMAP – aqueous pK_a

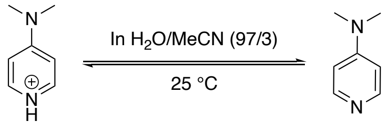

DMAP-H **DMAP**

VB24. Stock solution A with 3.04 mg DMAP (**1**) in 25 mL H_2O/MeCN mix (1:1), diluted to c = 0.05 mM, measured at λ_{max} = 280 nm.

Buffer	%fb	pH_{buffer}	p$H_{baseline}$	Abs (280nm)
Phosphate	50	6.72	6.75	0.87
TEOA	20	7.37	7.35	0.85
	40	7.81	7.79	0.89
	70	8.37	8.35	0.85
	80	8.62	8.59	0.87
Carbonate	10	9.04	9.09	0.79
	25	9.49	9.53	0.67
	50	9.97	10.03	0.51
PIP	20	10.71	10.71	0.34
	60	11.47	11.48	0.28

$K_a = 1.42 \times 10^{-10}$ → pK_a = 9.85, Lit: pK_a = 9.66[20]

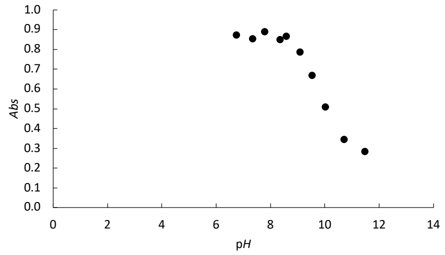

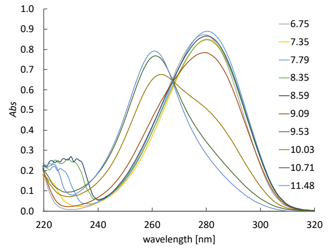

Pyridinamide 3 – aqueous pK_a

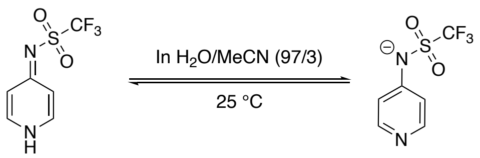

PA_3 **an3**

VB09. Stock solution A with 2.30 mg PA_**3** in 10 mL H_2O/MeCN mix (1:1), diluted to c = 0.05 mM, measured at λ_{max} = 272 nm. Not included in pK_a determination.

Buffer	%fb	pH_{buffer}	p$H_{baseline}$	Abs (272nm)
HCl	–	2.1	2.14	1.33
Formate	10	2.51	2.55	1.33
	50	3.55	3.59	1.30
Acetate	30	4.17	4.23	1.30
	70	4.94	5.00	1.24
Phosphate	10	5.68	5.74	1.31

TEOA-HCl	10	6.94	6.98	1.11
	30	7.55	7.58	0.70
	50	7.95	7.97	0.44
	70	8.36	8.36	0.25
	90	8.96	8.96	0.09
Carbonate	10	9.02	9.13	0.07
	25	9.45	9.54	0.04
	50	9.93	9.99	0.03
PIP-HCl	20	10.74	10.71	0.03
	60	11.50	11.47	0.04

$K_a = 2.24 \times 10^{-8}$ → $pK_a = 7.65$

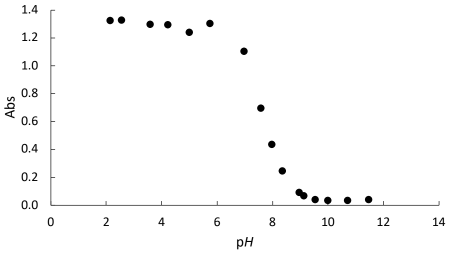

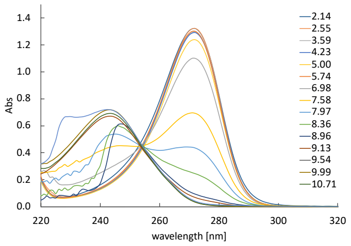

VB13. Stock solution A with 2.25 mg PA_**3** in 10 mL H_2O/MeCN mix (1:1), diluted to c = 0.05 mM, measured at λ_{max} = 272 nm.

**Buffer**	**%fb**	**p*H*$_{buffer}$**	**p*H*$_{baseline}$**	**Abs (272nm)**
Acetate	30	4.17	4.19	1.72
Phosphate	10	5.68	5.72	1.71
TEOA-HCl	10	6.94	6.96	1.42
	30	7.55	7.55	0.95
	50	7.95	7.93	0.61
	70	8.36	8.33	0.34
	90	8.96	8.91	0.13
Carbonate	10	9.02	9.1	0.11
	25	9.45	9.49	0.07
	50	9.93	9.95	0.05

$K_a = 2.38 \times 10^{-8}$ → $pK_a = 7.62$

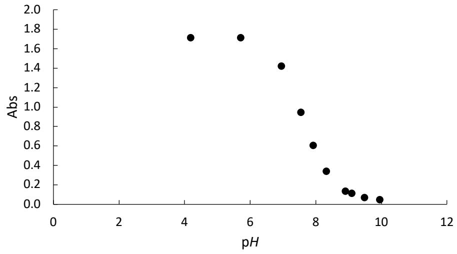

VB16. Stock solution A with 2.26 mg PA_**3** in 10 mL H_2O/MeCN mix (1:1), diluted to c = 0.05 mM, measured at λ_{max} = 272 nm.

**Buffer**	**%fb**	**p*H*$_{buffer}$**	**p*H*$_{baseline}$**	**Abs (272nm)**
HCl	–	1.09	1.12	1.09
HCl	–	1.6	1.64	1.10
HCl	–	2.1	2.12	1.09
Formate	10	2.51	2.52	1.13
	50	3.55	3.59	1.12
Acetat	30	4.17	4.24	1.11
	70	4.94	4.99	1.13
Phosphate	10	5.68	5.75	1.09
TEOA-HCl	10	6.94	6.95	0.98
	30	7.55	7.55	0.53
	50	7.95	7.93	0.38
	70	8.36	8.33	0.22

	90	8.96	8.89	0.13
Carbonate	10	9.02	9.12	0.06
	25	9.45	9.53	0.04
	50	9.93	9.98	0.02
PIP-HCl	20	10.74	10.72	0.03
	60	11.5	11.5	0.03

$K_a = 2.60 \times 10^{-8}$ → pK_a = 7.59

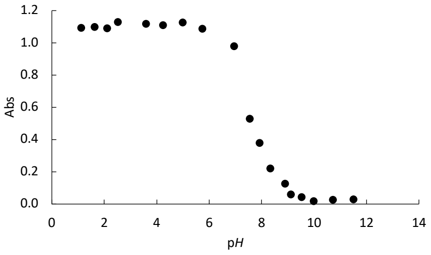

Pyridinamide 3 – aqueous pK_a with I = 0.15 M

Stock solutions were prepared according to procedure for pK_a measurements in 1:1 water/MeCN mixtures to verify that the change in overall ionic strength I by dilutions has no significant influence on the pK_a value.

VB35. Stock solution A with 2.31 mg PA_**3** in 10 mL H_2O/MeCN mix (1:1), diluted to c = 0.05 mM, measured at λ_{max} = 272 nm.

Buffer	%fb	pH_{buffer}	p$H_{baseline}$	Abs (272nm)
Acetat	30	4.17	4.23	1.23
	70	4.94	5.01	1.23
Phosphate	10	5.68	5.96	1.20
	50	6.72	6.87	1.07
TEOA	20	7.37	7.30	0.87
	40	7.81	7.75	0.60
	70	8.37	8.29	0.31
	80	8.62	8.53	0.24
Carbonate	10	9.04	9.25	0.13
	25	9.46	9.65	0.11
	50	9.97	10.12	0.11
PIP	20	10.71	10.59	0.11

$K_a = 2.29 \times 10^{-8}$ → pK_a = 7.64

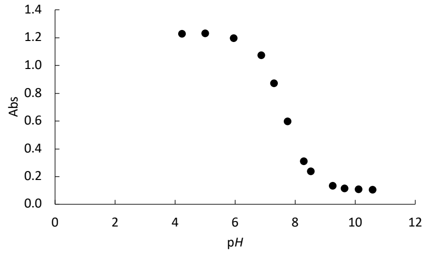

Pyridinamide 5 – aqueous pK_a

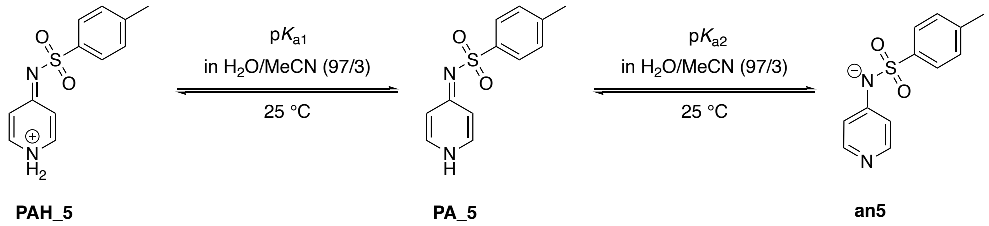

VB12. Stock solution A with 2.48 mg PA_**5** in 10 mL H_2O/MeCN mix (1:1), diluted to c = 0.05 mM, measured at λ_{max} = 285 nm.

Buffer	%fb	pH_{buffer}	p$H_{baseline}$	Abs (285nm)	pKa_1	pKa_2
HCl	–	1.09	1.12	0.05	X	
HCl	–	1.6	1.64	0.07	X	
HCl	–	2.1	2.14	0.16	X	
Formate	10	2.51	2.55	0.21	X	
	50	3.55	3.59	0.79	X	
Acetat	30	4.17	4.24	1.20	X	
	70	4.94	5.00	1.33	X	
Phosphate	10	5.68	5.75	1.33	X	X
TEOA	10	6.94	6.96	1.35		X
	30	7.55	7.56	1.28		X
	50	7.95	7.93	1.22		X
	70	8.36	8.34	1.04		X
	90	8.96	8.91	0.68		X
Carbonate	10	9.02	9.2	0.52		X
	25	9.45	9.51	0.36		X
	50	9.93	9.98	0.23		X
PIP	20	10.74	10.71	0.16		X
	60	11.5	11.47	0.16		X

$K_{a1} = 3.72 \times 10^{-4}$ → pK_{a1} = 3.43

$K_{a2} = 1.51 \times 10^{-9}$ → pK_{a2} = 8.82

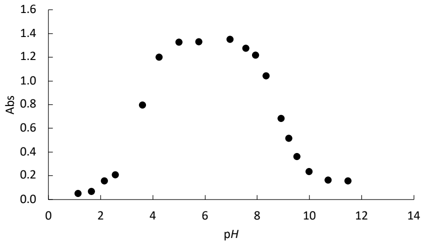

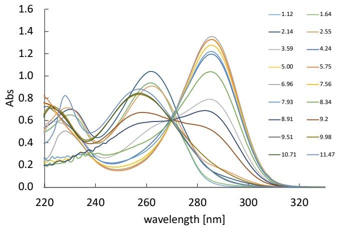

VB17. Stock solution A with 2.50 mg PA_**5** in 10 mL H_2O/MeCN mix (1:1), diluted to c = 0.05 mM, measured at λ_{max} = 285 nm.

Buffer	%fb	pH_{buffer}	p$H_{baseline}$	Abs (285nm)	pKa_1	pKa_2
HCl	–	1.09	1.12	0.05	X	
HCl	–	1.6	1.64	0.07	X	
HCl	–	2.1	2.12	0.13	X	
Formate	10	2.51	2.52	0.21	X	
	50	3.55	3.59	0.85	X	
Acetat	30	4.17	4.24	1.24	X	
	70	4.94	4.99	1.37	X	
Phosphate	10	5.68	5.75	1.43	X	X
TEOA	10	6.94	6.95	1.41		X
	30	7.55	7.55	1.30		X
	50	7.95	7.93	1.26		X
	70	8.36	8.33	1.11		X
	90	8.96	8.89	0.75		X
Carbonate	10	9.02	9.12	0.56		X
	25	9.45	9.53	0.36		X
	50	9.93	9.98	0.23		X
PIP	20	10.74	10.72	0.16		X
	60	11.50	11.50	0.15		X

$K_{a1} = 3.63 \times 10^{-4}$ → pK_{a1} = 3.44

$K_{a2} = 1.48 \times 10^{-9}$ → pK_{a2} = 8.83

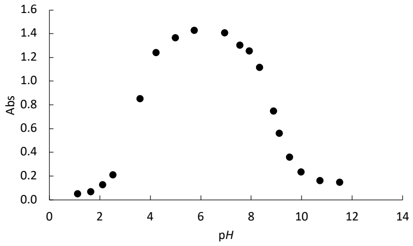

Pyridinamide ion pair 5a – aqueous pK_a

Stock solution prepared using pyridinamide ion pair **5a** rather than pyridinamide **5** to examine the potential influence of the counter-cation **a** on the measurement. The measurement was performed following two wavelength: at 285 nm for the pyridinamide and at 258 nm for the pyridinamide anion **5**.

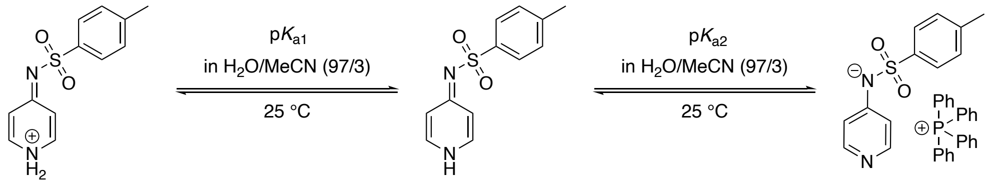

PAH_5 **PA_5** **5a**

VB25. Stock solution A with 5.89 mg **5a** in 10 mL H_2O/MeCN mix (1:1), diluted to c = 0.05 mM, measured at λ_{max}(PA_**5**) = 285 nm and λ_{max}(**5a**) = 258 nm.

Buffer	%fb	pH_{buffer}	p$H_{baseline}$	Abs (258nm)	Abs (285nm)	pKa_1	pKa_2
HCl	0.1	1.09	1.11	1.41	0.18	X	
HCl	0.03	1.60	1.62	1.42	0.19	X	
HCl	0.01	2.10	2.10	–	0.24	X	
Formate	10	2.51	2.51	1.31	0.34	X	
	50	3.55	3.59	0.83	1.15	X	
Acetat	30	4.17	4.21	0.58	1.57	X	
	70	4.94	5.00	0.51	1.78	X	
Phosphate	10	5.68	5.86	0.47	1.78	X	X
	50	6.72	6.75	0.48	1.78		X
TEOA	20	7.37	7.35	0.49	1.72		X
	40	7.81	7.81	0.54	1.64		X
	70	8.37	8.37	0.68	1.39		X
	80	8.62	8.62	0.77	1.20		X
Carbonate	10	9.04	9.13	1.00	0.78		X
	25	9.46	9.57	1.15	0.51		X
	50	9.97	10.05	1.26	0.37		X
PIP	20	10.71	10.72	1.28	0.29		X
	60	11.47	11.51	1.30	0.27		X

258 nm: $K_{a1} = 4.36 \times 10^{-4}$ → pK_{a1} = 3.36 285 nm: $K_{a1} = 3.85 \times 10^{-4}$ → pK_{a1} = 3.41

$K_{a2} = 1.35 \times 10^{-9}$ → pK_{a2} = 8.87 $K_{a2} = 1.48 \times 10^{-9}$ → pK_{a2} = 8.83

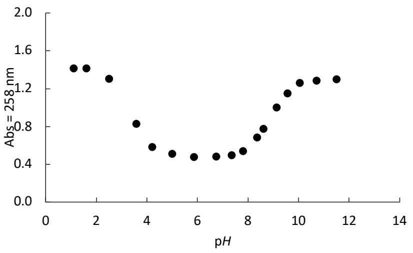

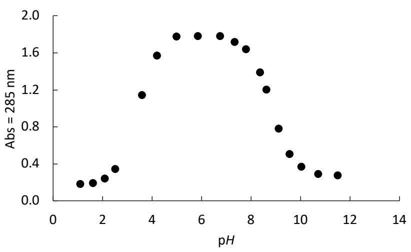

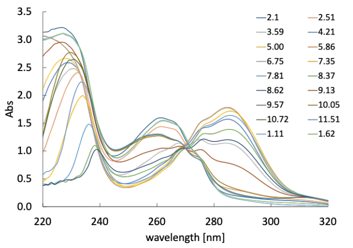

Pyridinamide 4 – aqueous pK_a

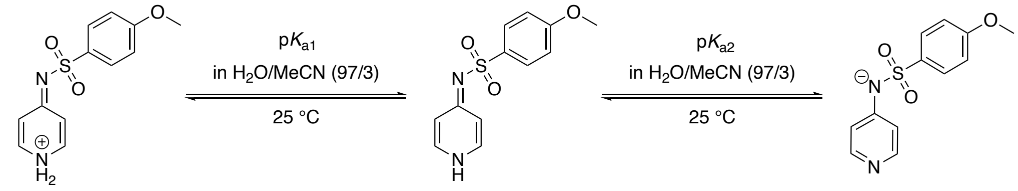

PAH_4 **PA_4** **an4**

VB10. Stock solution A with 2.65 mg PA_**4** in 10 mL H_2O/MeCN mix (1:1), diluted to c = 0.05 mM, measured at λ_{max} = 286 nm.

Buffer	%fb	pH_{buffer}	p$H_{baseline}$	Abs (286nm)	pKa_1	pKa_2
HCl	–	1.09	1.12	0.33	X	
HCl	–	1.6	1.64	0.34	X	
HCl	–	2.1	2.14	0.42	X	
Formate	10	2.51	2.55	0.47	X	
	50	3.55	3.59	1.10	X	
Acetat	30	4.17	4.23	1.46	X	
	70	4.94	5.00	1.72	X	
Phosphate	10	5.68	5.75	1.75	X	
TEOA	10	6.94	6.96	1.73		X
	30	7.55	7.57	1.67		X
	50	7.95	7.95	1.57		X
	70	8.36	8.35	1.46		X
	90	8.96	8.93	0.90		X
Carbonate	10	9.02	9.16	0.71		X
	25	9.45	9.51	0.48		X
	50	9.93	9.97	0.32		X
PIP	20	10.74	10.71	0.22		X
	60	11.5	11.47	0.21		X

$K_{a1} = 2.84 \times 10^{-4}$ → pK_{a1} = 3.55

$K_{a2} = 1.35 \times 10^{-9}$ → pK_{a2} = 8.87

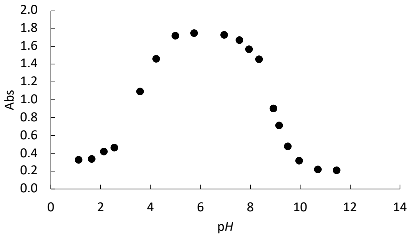

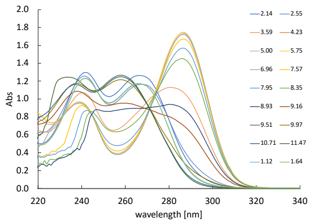

VB18. Stock solution A with 2.62 mg PA_**4** in 10 mL H_2O/MeCN mix (1:1), diluted to c = 0.05 mM, measured at λ_{max} = 285 nm.

Buffer	%fb	pH_{buffer}	p$H_{baseline}$	Abs (285nm)	pKa_1	pKa_2
HCl	–	1.09	1.12	0.26	X	
HCl	–	1.6	1.64	0.27	X	
HCl	–	2.1	2.12	0.31	X	
Formate	10	2.51	2.52	0.36	X	
	50	3.55	3.59	0.78	X	
Acetat	30	4.17	4.24	1.08	X	
	70	4.94	4.99	1.24	X	
Phosphate	10	5.68	5.75	1.28	X	X
TEOA	10	6.94	6.95	1.25		X
	30	7.55	7.55	1.15		X
	50	7.95	7.93	1.12		X
	70	8.36	8.33	0.95		X
	90	8.96	8.89	0.68		X
Carbonate	10	9.02	9.12	0.53		X

	25	9.45	9.53	0.35		X
	50	9.97	10.03	0.22		X
PIP	20	10.74	10.72	0.17		X
	60	11.5	11.5	0.17		X

$K_{a1} = 2.60 \times 10^{-4}$ → $pK_{a1} = 3.59$

$K_{a2} = 1.48 \times 10^{-9}$ → $pK_{a2} = 8.83$

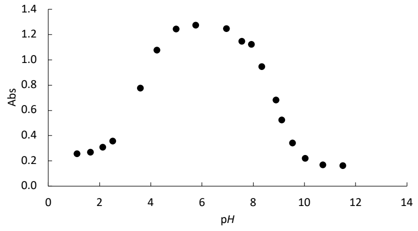

Pyridinamide 6 – aqueous p*K*a

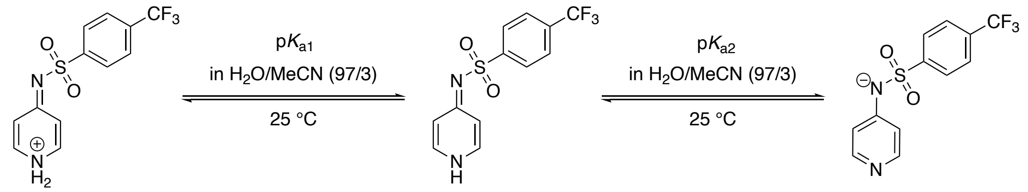

PAH_6 **PA_6** **an6**

VB11. Stock solution A with 3.03 mg PA_**6** in 10 mL H_2O/MeCN mix (1:1), diluted to $c = 0.05$ mM, measured at λ_{max} = 284 nm.

Buffer	%fb	p*H*buffer	p*H*baseline	Abs (284nm)	p*K*a1	p*K*a2
HCl	–	1.09	1.12	0.07	X	
HCl	–	1.6	1.64	0.15	X	
HCl	–	2.1	2.14	0.37	X	
Formate	10	2.51	2.55	0.62	X	
	50	3.55	3.59	1.34	X	
Acetat	30	4.17	4.17	1.44	X	
	70	4.94	5.00	1.49	X	
Phosphate	10	5.68	5.76	1.53	X	X
TEOA	10	6.94	6.95	1.46		X
	30	7.55	7.56	1.52		X
	50	7.95	7.94	1.30		X
	70	8.36	8.35	1.06		X
	90	8.96	8.96	0.66		X
Carbonate	10	9.02	9.19	0.52		X
	25	9.45	9.54	0.38		X
	50	9.93	9.98	0.29		X
PIP	20	10.74	10.71	0.23		X
	60	11.5	11.47	0.23		X

$K_{a1} = 2.91 \times 10^{-3}$ → $pK_{a1} = 2.72$

$K_{a2} = 2.31 \times 10^{-9}$ → $pK_{a2} = 8.64$

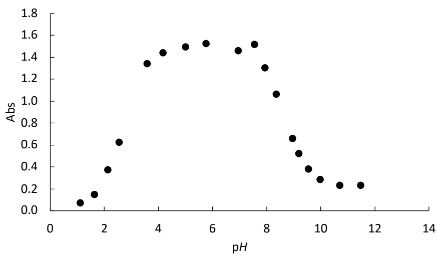

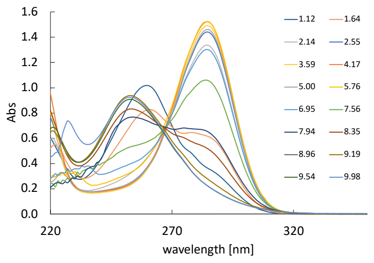

VB19. Stock solution A with 3.01 mg PA_**6** in 10 mL H_2O/MeCN mix (1:1), diluted to c = 0.05 mM, measured at λ_{max} = 285 nm.

Buffer	%fb	pH_{buffer}	p$H_{baseline}$	Abs (285nm)	pKa_1	pKa_2
HCl	–	1.09	1.12	0.04	X	
HCl	–	1.6	1.64	0.11	X	
HCl	–	2.1	2.12	0.27	X	
Formate	10	2.51	2.52	0.54	X	
	50	3.55	3.59	1.10	X	
Acetat	30	4.17	4.24	1.22	X	
	70	4.94	4.99	1.21	X	
Phosphate	10	5.68	5.75	1.26	X	X
TEOA	10	6.94	6.95	1.20		X
	30	7.55	7.55	1.10		X
	50	7.95	7.93	1.03		X
	70	8.36	8.33	0.84		X
	90	8.96	8.89	0.65		X
Carbonate	10	9.02	9.12	0.40		X
	25	9.45	9.53	0.28		X
	50	9.97	10.03	0.21		X
PIP	20	10.74	10.72	0.19		X
	60	11.5	11.5	0.18		X

$K_{a1} = 2.24 \times 10^{-3}$ → pK_{a1} = 2.65

$K_{a2} = 2.25 \times 10^{-9}$ → pK_{a2} = 8.65

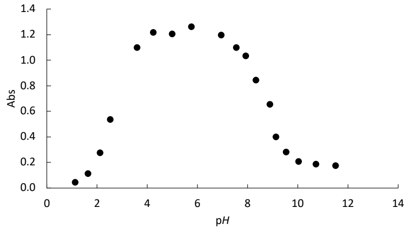

6.2 General Information – pK_a measurements in MeCN mixtures

The respective ion pairs **3-6a** were used for stock solutions due to solubility issues of the pyridinamides for the pK_a determination in water/MeCN mixtures with a ratio of 50/50. Stock solution A with c = 1.0 mM of the respective neutral pyridinamides in MeCN was prepared and diluted with MeCN to give stock solution B with c = 0.1 mM. Subsequently, 0.5 mL of stock solution B was combined with 0.5 mL of buffer solution to give the final measurement mixture with a compound concentration of c = 0.05 mM.

However, due to dilution the final ionic strength of the measurement solution was reduced to I = 0.15 M. To verify that the reduced ionic strength would not influence the final pK_a value a test measurement with neutral pyridinamide SA**3** was performed with the same conditions as for the aqueous pK_a measurement with the exception that the stock solution was diluted in a way that the ionic strength of the measurement mixture would be I = 0.15 M. The resulting pK_a value of this measurement did not deviate from the pK_a value obtained with I = 0.3 M. To the observed pH_{obs} of the water/MeCN mixtures a correction factor δ_{MeCN} must be applied since the pH_{obs} was determined with an electrode calibrated in aqueous solutions. Therefore, a correction of -0.257 was added to the measured output of the pH probe.[21]

DMAP –pK_a in MeCN mixture

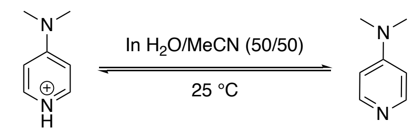

DMAP-H **DMAP**

VB30. Stock solution A with 12.2 mg DMAP (**1**) in 20 mL MeCN mix, diluted to c = 0.05 mM, measured at λ_{max} = 280 nm.

Buffer	%fb	pH_{buffer}	p$H_{baseline}$	pH(corr)	Abs (280nm)
Phosphate	50	6.72	7.52	7.263	0.94
TEOA	20	7.37	7.25	6.993	0.95
	40	7.81	7.68	7.423	0.94
	70	8.37	8.22	7.963	0.87
	80	8.62	8.49	8.233	0.81
Carbonate	10	9.04	10.12	9.863	0.31
	25	9.46	10.56	10.303	0.28
	50	9.97	11.03	10.773	0.27
PIP	20	10.71	10.19	9.933	0.31
	60	11.47	11	10.743	0.27

K_a = 1.65 x 10^{-9} → pK_a = 8.78

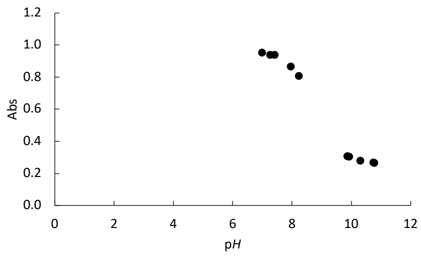

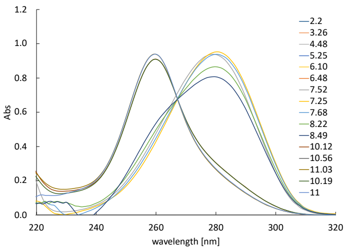

VB37. Stock solution A with 12.0 mg DMAP (**1**) in 20 mL MeCN mix, diluted to c = 0.05 mM, measured at λ_{max} = 280 nm.

Buffer	%fb	pH_{buffer}	p$H_{baseline}$	pH(corr)	Abs (280nm)
Acetat	30	4.17	5.29	5.033	1.06
	70	4.94	6.09	5.833	1.05
Phosphate	10	5.68	6.49	6.233	1.05
	50	6.72	7.52	7.263	1.02
TEOA	20	7.37	7.19	6.933	1.03
	40	7.81	7.64	7.383	1.01
	70	8.37	8.18	7.923	0.93
	80	8.62	8.42	8.163	0.87
Carbonate	10	9.04	10.16	9.903	0.33
	25	9.46	10.49	10.233	0.30
	50	9.97	11.04	10.783	0.29
PIP	20	10.71	10.11	9.853	0.34
	60	11.47	10.96	10.703	0.29

K_a = 2.16 x 10^{-9} → pK_a = 8.67

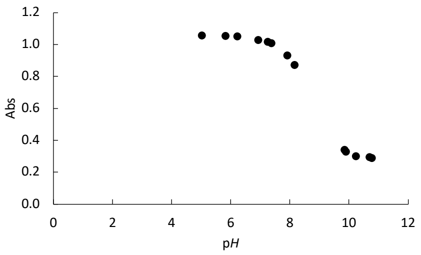

Ion Pair 3a –pK_a in MeCN mixture

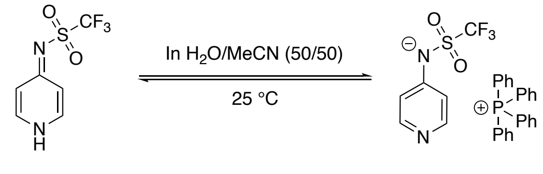

PA_3 **3a**

VB26. Stock solution A with 5.65 mg **3a** in 10 mL MeCN, diluted to c = 0.05 mM, measured at λ_{max} = 272 nm.

Buffer	%fb	pH_{buffer}	p$H_{baseline}$	pH(corr)	Abs (272nm)	pKa
HCl	0.1	1.08	1.17	0.913	1.65	
HCl	0.03	1.6	1.71	1.453	1.67	
HCl	0.01	2.1	2.2	1.943	1.66	
Formate	10	2.51	3.26	3.003	1.66	X
	50	3.55	4.48	4.223	1.66	X
Acetat	30	4.17	5.25	4.993	1.64	X
	70	4.94	6.10	5.84	1.62	X
Phosphate	10	5.68	6.48	6.223	1.56	X
	50	6.72	7.52	7.263	1.10	X
TEOA	20	7.37	7.18	6.923	1.30	X
	40	7.81	7.66	7.403	1.01	X
	70	8.37	8.2	7.943	0.69	X
	80	8.62	8.46	8.203	0.60	X
Carbonate	10	9.04	10.13	9.873	0.46	X
	25	9.46	10.56	10.303	0.44	X
	50	9.97	11.03	10.773	0.44	X
PIP	20	10.71	10.19	9.933	0.44	X
	60	11.47	11	10.743	0.44	X

$K_a = 4.60 \times 10^{-8}$ → pK_a = 7.34

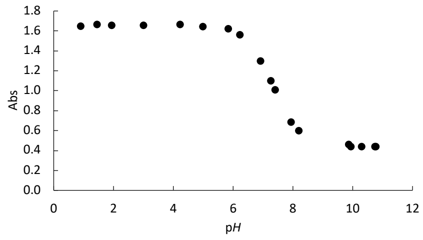

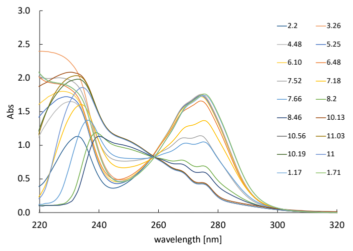

VB36. Stock solution A with 11.2 mg **3a** in 10 mL MeCN, diluted to c = 0.05 mM, measured at λ_{max} = 272 nm.

Buffer	%fb	pH_{buffer}	p$H_{baseline}$	pH(corr)	Abs (272nm)
Formate	10	2.51	3.24	2.983	1.33
	50	3.55	4.48	4.223	1.33
Acetat	30	4.17	5.29	5.033	1.33
	70	4.94	6.09	5.833	1.30
Phosphate	10	5.68	6.49	6.233	1.24
	50	6.72	7.52	7.263	0.80
TEOA	20	7.37	7.19	6.933	0.98
	40	7.81	7.64	7.383	0.70
	70	8.37	8.18	7.923	0.43
	80	8.62	8.42	8.163	0.38
Carbonate	10	9.04	10.16	9.903	0.22
	25	9.46	10.49	10.233	0.22
	50	9.97	11.04	10.783	0.22
PIP	20	10.71	10.11	9.853	0.22

$K_a = 5.22 \times 10^{-8}$ → pK_a = 7.28

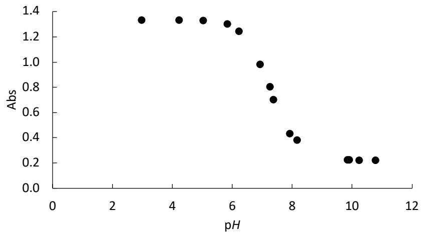

Ion Pair 5a –pK_a in MeCN mixture

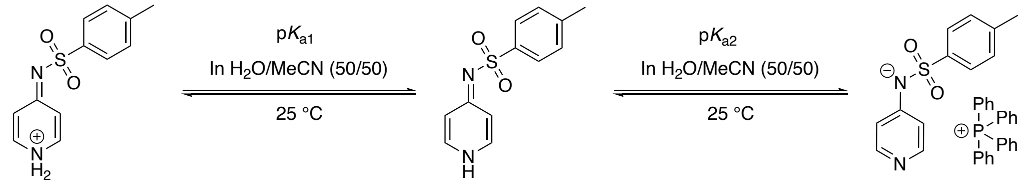

PAH_5 **PA_5** **5a**

VB27. Stock solution A with 5.85 mg **5a** in 10 mL MeCN, diluted to c = 0.05 mM, measured at λ_{max} = 290 nm.

Buffer	%fb	pH_{buffer}	p$H_{baseline}$	pH(corr)	Abs (290nm)	pKa1	pKa2
HCl	0.1	1.08	1.17	0.913	0.08	X	
HCl	0.03	1.6	1.71	1.453	0.08	X	
HCl	0.01	2.1	2.2	1.943	0.11	X	
Formate	10	2.51	3.26	3.003	0.45	X	
	50	3.55	4.48	4.223	1.17	X	
Acetat	30	4.17	5.25	4.993	1.26	X	
	70	4.94	6.10	5.843	1.29	X	X
Phosphate	10	5.68	6.48	6.223	1.28	X	X
	50	6.72	7.52	7.263	1.23		X
TEOA	20	7.37	7.18	6.923	1.27		X
	40	7.81	7.66	7.403	1.23		X
	70	8.37	8.2	7.943	1.10		X
	80	8.62	8.46	8.203	1.01		X
Carbonate	10	9.04	10.13	9.873	0.26		X
	25	9.46	10.56	10.303	0.21		X
	50	9.97	11.03	10.773	0.19		X
PIP	20	10.71	10.19	9.933	0.24		X
	60	11.47	11	10.743	0.19		X

$K_{a1} = 4.65 \times 10^{-4}$ → pK_{a1} = 3.33

$K_{a2} = 2.14 \times 10^{-9}$ → pK_{a2} = 8.67

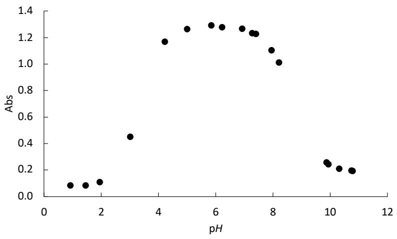

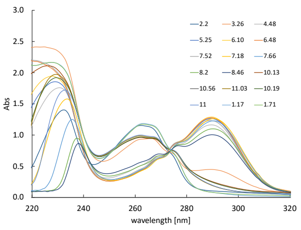

VB38. Stock solution A with 12.0 mg **5a** in 10 mL MeCN, diluted to c = 0.05 mM, measured at λ_{max} = 290 nm.

Buffer	%fb	pH_{buffer}	p$H_{baseline}$	pH(corr)	Abs (290nm)	pKa_1	pKa_2
HCl	0.1	1.08	1.2	0.943	0.07	X	
HCl	0.03	1.6	1.73	1.473	0.07	X	
HCl	0.01	2.1	2.21	1.953	0.10	X	
Formate	10	2.51	3.24	2.983	0.43	X	
	50	3.55	4.48	4.223	1.13	X	
Acetat	30	4.17	5.29	5.033	1.23	X	
	70	4.94	6.07	5.813	1.24	X	
Phosphate	10	5.68	6.5	6.243	1.23	X	X
	50	6.72	7.52	7.263	1.21		X
TEOA	20	7.37	7.21	6.953	1.21		X
	40	7.81	7.67	7.413	1.18		X
	70	8.37	8.21	7.953	1.07		X
	80	8.62	8.45	8.193	0.97		X
Carbonate	10	9.04	10.21	9.953	0.23		X
	25	9.46	10.48	10.223	0.20		X

	50	9.97	11.02	10.763	0.19		X
PIP	20	10.71	10.08	9.823	0.24		X
	60	11.47	10.95	10.693	0.19		X

$K_{a1} = 4.88 \times 10^{-4}$ → $pK_{a1} = 3.31$

$K_{a2} = 2.14 \times 10^{-9}$ → $pK_{a2} = 8.67$

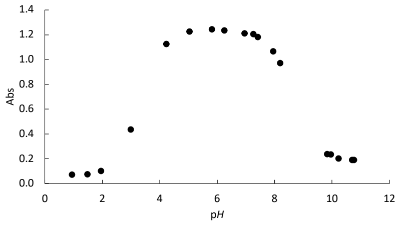

Ion Pair 4a –pK_a in MeCN mixture

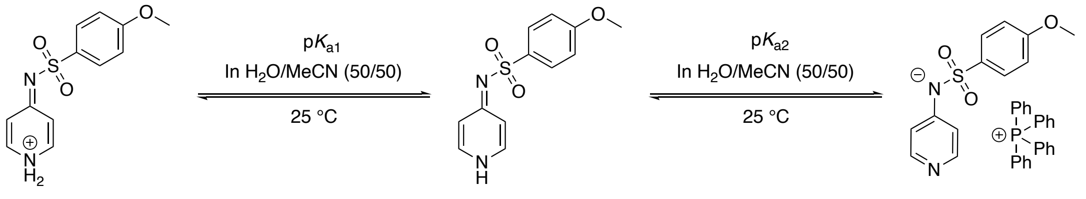

PAH_4 **PA_4** **4a**

VB28. Stock solution A with 6.02 mg **4a** in 10 mL MeCN, diluted to c = 0.05 mM, measured at λ_{max} = 290 nm.

Buffer	%fb	pH_{buffer}	p$H_{baseline}$	pH(corr)	Abs (290nm)	pKa_1	pKa_2
HCl	0.1	1.08	1.17	0.913	0.16	X	
HCl	0.03	1.6	1.71	1.453	0.17	X	
HCl	0.01	2.1	2.2	1.943	0.19	X	
Formate	10	2.51	3.26	3.003	0.41	X	
	50	3.55	4.48	4.223	1.03	X	
Acetat	30	4.17	5.25	4.993	1.15	X	
	70	4.94	6.10	5.843	1.17	X	X
Phosphate	10	5.68	6.48	6.223	1.17	X	X
	50	6.72	7.52	7.263	1.14		X
TEOA	20	7.37	7.18	6.923	1.15		X
	40	7.81	7.66	7.403	1.12		X
	70	8.37	8.2	7.943	1.01		X
	80	8.62	8.46	8.203	0.93		X
Carbonate	10	9.04	10.13	9.873	0.23		X
	25	9.46	10.56	10.303	0.19		X
	50	9.97	11.03	10.773	0.18		X
PIP	20	10.71	10.19	9.933	0.22		X
	60	11.47	11	10.743	0.17		X

$K_{a1} = 3.36 \times 10^{-4}$ → $pK_{a1} = 3.47$

$K_{a2} = 2.04 \times 10^{-9}$ → $pK_{a2} = 8.69$

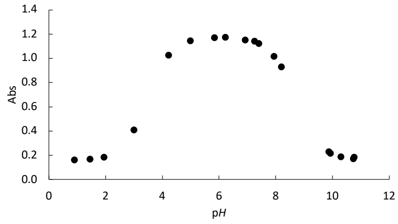

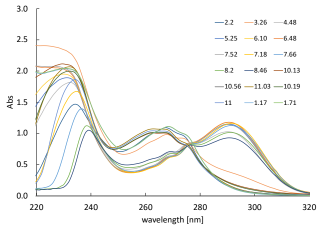

VB39. Stock solution A with 12.3 mg **4a** in 10 mL MeCN, diluted to *c* = 0.05 mM, measured at λ_{max} = 290 nm.

Buffer	%fb	p*H*$_{buffer}$	p*H*$_{baseline}$	pH(corr)	Abs (290nm)	p*K*a$_1$	p*K*a$_2$
HCl	0.1	1.08	1.2	0.943	0.18	X	
HCl	0.03	1.6	1.73	1.473	0.19	X	
HCl	0.01	2.1	2.21	1.953	0.21	X	
Formate	10	2.51	3.24	2.983	0.45	X	
	50	3.55	4.48	4.223	1.10	X	
Acetat	30	4.17	5.29	5.033	1.22	X	
	70	4.94	6.07	5.813	1.24	X	X
Phosphate	10	5.68	6.5	6.243	1.24	X	X
	50	6.72	7.52	7.263	1.20		X
TEOA	20	7.37	7.21	6.953	1.23		X
	40	7.81	7.67	7.413	1.19		X
	70	8.37	8.21	7.953	1.08		X
	80	8.62	8.45	8.193	0.99		X
Carbonate	10	9.04	10.21	9.953	0.24		X
	25	9.46	10.48	10.223	0.20		X
	50	9.97	11.02	10.763	0.19		X
PIP	20	10.71	10.08	9.823	0.29		X
	60	11.47	10.95	10.693	0.19		X

$K_{a1} = 3.64 \times 10^{-4}$ → pK_{a1} = 3.44

$K_{a2} = 1.95 \times 10^{-9}$ → pK_{a2} = 8.71

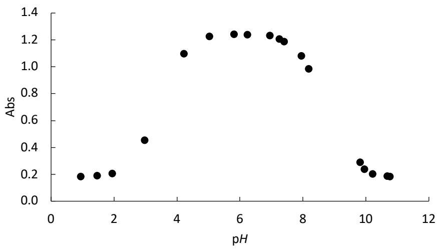

Ion Pair 6a –pK_a in MeCN mixture

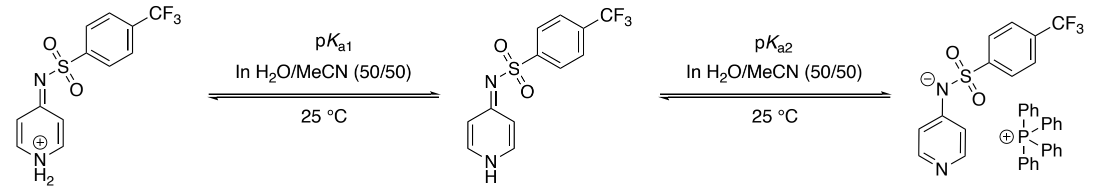

PAH_6 **PA_6** **6a**

VB29. Stock solution A with 6.41 mg **4a** in 10 mL MeCN, diluted to *c* = 0.05 mM, measured at λ_{max} = 290 nm.

Buffer	%fb	p*H*$_{buffer}$	p*H*$_{baseline}$	pH(corr)	Abs (290nm)	p*K*a$_1$	p*K*a$_2$
HCl	0.1	1.08	1.17	0.913	0.08	X	
HCl	0.03	1.6	1.71	1.453	0.16	X	
HCl	0.01	2.1	2.2	1.943	0.33	X	
Formate	10	2.51	3.26	3.003	0.98	X	
	50	3.55	4.48	4.223	1.22	X	
Acetat	30	4.17	5.25	4.993	1.24	X	X
	70	4.94	6.10	5.843	1.24	X	X
Phosphate	10	5.68	6.48	6.223	1.23		X
	50	6.72	7.52	7.263	1.16		X
TEOA	20	7.37	7.18	6.923	1.20		X
	40	7.81	7.66	7.403	1.14		X
	70	8.37	8.2	7.943	0.95		X
	80	8.62	8.46	8.203	0.83		X
Carbonate	10	9.04	10.13	9.873	0.27		X
	25	9.46	10.56	10.303	0.25		X
	50	9.97	11.03	10.773	0.24		X

PIP	20	10.71	10.19	9.933	0.27		X
	60	11.47	11	10.743	0.25		X

K_{a1} = 3.54 x 10^{-3} → pK_{a1} = 2.45

K_{a2} = 4.47 x 10^{-9} → pK_{a2} = 8.35

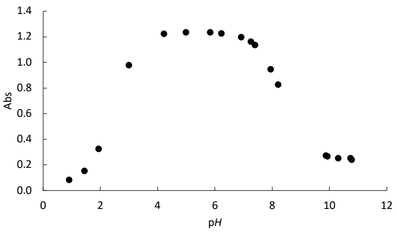

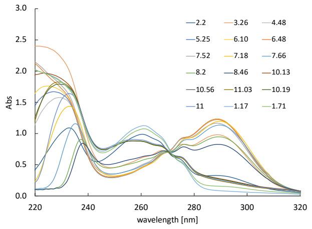

VB40. Stock solution A with 12.9 mg **4a** in 10 mL MeCN, diluted to *c* = 0.05 mM, measured at λ_{max} = 290 nm.

**Buffer**	**%fb**	**p*H*$_{buffer}$**	**p*H*$_{baseline}$**	**pH(corr)**	**Abs (290nm)**	**p*K*a$_1$**	**p*K*a$_2$**
HCl	0.1	1.09	1.2	0.943	0.07	X	
HCl	0.03	1.6	1.73	1.473	0.15	X	
HCl	0.01	2.1	2.21	1.953	0.32	X	
Formate	10	2.51	3.24	2.983	0.99	X	
	50	3.55	4.48	4.223	1.24	X	
Acetat	30	4.17	5.29	5.033	1.26	X	X
	70	4.94	6.07	5.813	1.26	X	X
Phosphate	10	5.68	6.5	6.243	1.23		X
	50	6.72	7.52	7.263	1.17		X
TEOA	20	7.37	7.21	6.953	1.20		X
	40	7.81	7.67	7.413	1.13		X
	70	8.37	8.21	7.953	0.95		X
	80	8.62	8.45	8.193	0.83		X
Carbonate	10	9.04	10.21	9.953	0.26		X
	25	9.46	10.48	10.223	0.24		X
	50	9.97	11.02	10.763	0.23		X
PIP	20	10.71	10.08	9.823	0.26		X
	60	11.47	10.95	10.693	0.23		X

K_{a1} = 3.54 x 10^{-3} → pK_{a1} = 2.45

K_{a2} = 4.58 x 10^{-9} → pK_{a2} = 8.34

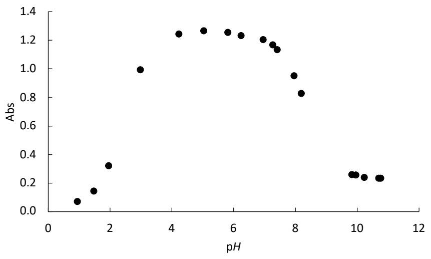

6.3 General Information – pK_a measurements in DMSO mixtures

Stock solution A with c = 1.0 mM of the respective neutral pyridinamides in DMSO was prepared and diluted with DMSO to give stock solution B with c = 0.1 mM. 0.5 mL of stock solution B was combined with 0.5 mL of buffer solution to give the final measurement mixture with a compound concentration of c = 0.05 mM. In this chapter only observed p*H* values are reported for the deprotonation of the neutral pyridinamide to the fully deprotonated anion.

Pyridinamide 3 –pK_a in DMSO

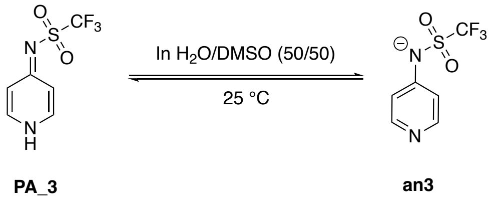

VB31. Stock solution A with 2.29 mg PA_**3** in 10 mL DMSO, diluted to c = 0.05 mM, measured at λ_{max} = 275 nm.

Buffer	%fb	pH_{buffer}	p$H_{baseline}$	Abs (275nm)
Acetat	30	4.17	5.9	1.39
	70	4.94	6.75	1.25
Phosphate	50	6.94	9	0.14
TEOA	20	7.55	7.54	0.71
	40	7.95	7.9	0.39
	70	8.36	8.51	0.18
	80	8.96	8.75	0.14
Carbonate	10	9.02	11.79	0.06
PIP	20	10.74	10.63	0.06
	60	11.5	11.46	0.06

K_a = 3.47 x 10^{-8} → pK_a = 7.46

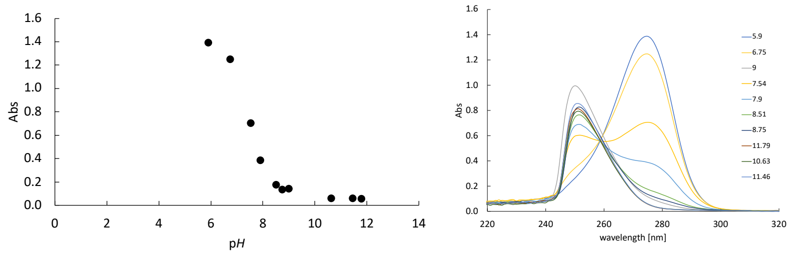

Pyridinamide 5 –pK_a in DMSO

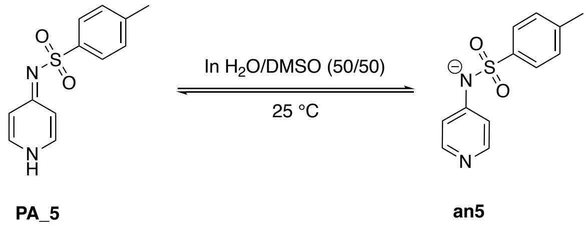

VB32. Stock solution A with 2.47 mg PA_**5** in 10 mL DMSO, diluted to c = 0.05 mM, measured at λ_{max} = 292 nm.

Buffer	%fb	pH_{buffer}	p$H_{baseline}$	Abs (292nm)
Acetat	30	4.17	5.9	1.53
	70	4.94	6.75	1.54
Phosphate	50	6.94	9	0.81
TEOA	20	7.55	7.54	1.50
	40	7.95	7.9	1.40
	70	8.36	8.51	1.17
	80	8.96	8.75	1.00
Carbonate	10	9.02	11.79	0.21
PIP	20	10.74	10.63	0.24
	60	11.5	11.46	0.20

K_a = 1.21 x 10^{-9} → pK_a = 8.92

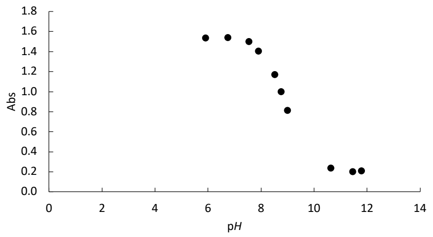

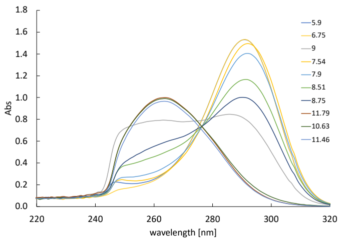

Pyridinamide 4 –pK_a in DMSO

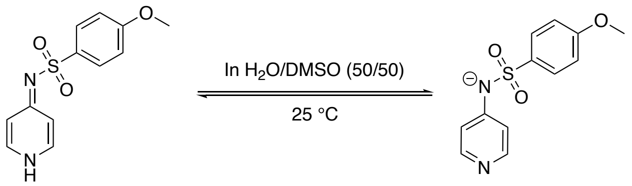

PA_4 **an4**

VB33. Stock solution A with 3.63 mg PA_**4** in 10 mL DMSO, diluted to c = 0.05 mM, measured at λ_{max} = 292 nm.

Buffer	%fb	pH_{buffer}	p$H_{baseline}$	Abs (292nm)
Acetat	30	4.17	5.9	1.57
	70	4.94	6.75	1.57
Phosphate	50	6.94	9	0.88
TEOA	20	7.55	7.54	1.54
	40	7.95	7.9	1.45
	70	8.36	8.51	1.23
	80	8.96	8.75	1.08
Carbonate	10	9.02	11.79	0.24
	25	9.45	12.25	0.25
PIP	20	10.74	10.63	0.28
	60	11.5	11.46	0.25

K_a = 1.08 x 10^{-9} → pK_a = 8.97

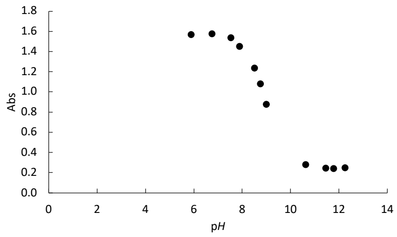

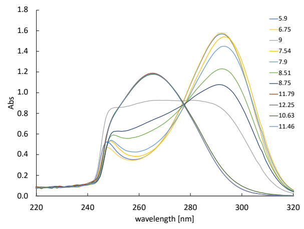

Pyridinamide 6 –pK_a in DMSO

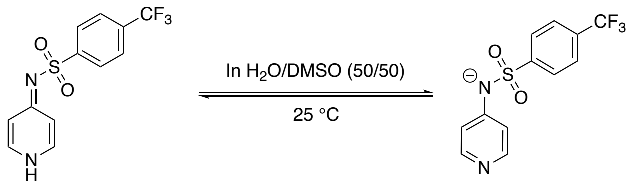

PA_6 **an6**

VB34. Stock solution A with 3.04 mg PA_**6** in 10 mL DMSO, diluted to c = 0.05 mM, measured at λ_{max} = 292 nm.

Buffer	%fb	pH_{buffer}	p$H_{baseline}$	Abs (292nm)
Acetat	30	4.17	5.90	1.26
	70	4.94	6.75	1.27
Phosphate	50	6.94	9.00	0.51

TEOA	20	7.55	7.54	1.20
	40	7.95	7.90	1.08
	70	8.36	8.51	0.80
	80	8.96	8.75	0.65
Carbonate	10	9.02	11.79	0.24
	25	9.45	12.25	0.23
PIP	20	10.74	10.63	0.25
	60	11.5	11.46	0.24

$K_a = 2.70 \times 10^{-9}$ → $pK_a = 8.57$

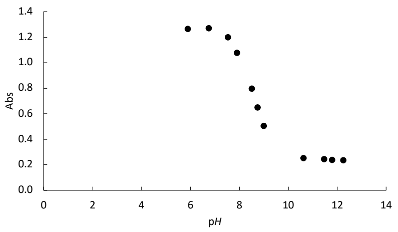

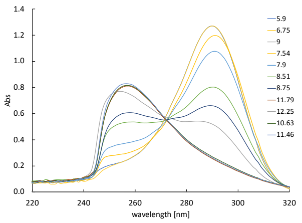

7. NMR Kinetics

The urethane synthesis was chosen as benchmark reaction to insert the newly designed ion pair catalyst into to existing reactivity library and compare their reactivity with already known ion pair catalysts. The benchmark reaction was performed with pyridinamide ion pair **3a**, **3b**, **5a,** and **6a**. The effective rate constants for 1, 2, **3c** and **4a** were taken from Helberg and Zipse.[3]

The reactant *p*-tosyl isocyanate (**9**, reagent grade) was distilled prior to use. The reactant 1-butanol (**10**, reagent grade) was distilled and stored over 4Å MS and under N_2 prior to use.

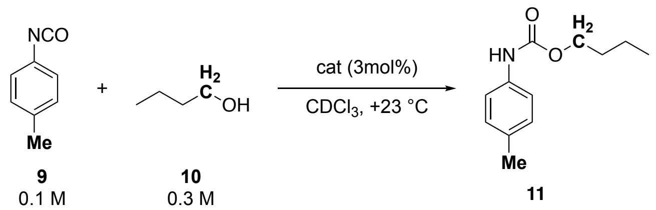

Scheme S7. Urethane synthesis of 1-butanol and tosyl isocyanate.

The experimental procedure and evaluation of the urethane synthesis was done as described as in Helberg and Zipse[3] There the conversion was calculated according to equation S27.

$$\text{Conversion [\%]} = \frac{3 \times \text{product(CH2)}}{2 \times (\text{prod(me)} + \text{isocyanat(Me)})} \times 100 \tag{S27}$$

The conversion is then used to calculate the concentration of urethane at the time t c_t[product] based on the initial concentration c_0 (eq. S28).

$$c_t = c(\mathbf{9})_0 - \left(\frac{c(\mathbf{9})_0 \times (100 - \text{conversion})}{100}\right) \tag{S28}$$

Numerical simulation according to equation S30 were performed using the program COPASI to obtain the respective effective rate constants k_{eff}.

$$[\mathbf{9}] + [\mathbf{10}] = [\text{urethane } \mathbf{11}] \tag{S30}$$

The benchmark reaction was performed with three different catalyst loadings and in two independent runs each. The resulting averaged effective rate constants k_{eff} are summarized in Table S16.

Table S16. List of averaged k_{eff} values obtained for the urethane synthesis with 1.0 mol%, 3.0 mol%, and 6.0 mol% catalyst loading.

IP	$k_{eff}(1)$ / M^{-1} s^{-1}	$k_{eff}(3)$ / M^{-1} s^{-1}	$k_{eff}(6)$ / M^{-1} s^{-1}	k_{cat} / M^{-2} s^{-1}
**1**[a]		2.80 x 10^{-4}		0.086
**2**[a]	2.78 x 10^{-4}	7.50 x 10^{-4}		0.364
**3a**	2.26 x 10^{-4} ± 3.67 x 10^{-6}	6.01 x 10^{-4} ± 1.50 x 10^{-6}	1.20 x 10^{-3} ± 2.19 x 10^{-5}	0.196
**3b**	2.45 x 10^{-4} ± 1.86 x 10^{-6}	7.15 x 10^{-4} ± 1.56 x 10^{-5}	1.27 x 10^{-3} ± 1.61 x 10^{-5}	0.205
**3c**[a]	–	3.65 x 10^{-4}	–	0.115
**4a**[a]	8.04 x 10^{-4}[b]	1.99 x 10^{-3}	3.96 x 10^{-3}	0.661
**5a**	5.28 x 10^{-4} ± 1.95 x 10^{-6}	1.53 x 10^{-3} ± 8.03 x 10^{-6}	2.90 x 10^{-3} ± 1.20 x 10^{-4}	0.477
**6a**	4.30 x 10^{-4} ± 3.14 x 10^{-6}	1.25 x 10^{-3} ± 2.50 x 10^{-6}	2.58 x 10^{-3} ± 4.34 x 10^{-5}	0.433

[a] values taken from ref. 3. [b] measured with 1.2 mol% catalyst load.

Subsequently the effective rate constants can be used to determine the concentration-independent rate constant of each catalyst according to equation S31.

$$k_{eff} = k_{cat} \times [\text{catalyst}] \quad \text{(S31)}$$

Plotting the effective rate constant k_{eff} against the respective catalyst load [catalyst] reveals a clear linear correlation for all investigated systems with a very small Y-intercept, thus, indicating that any potential uncatalyzed background reaction is negligible.

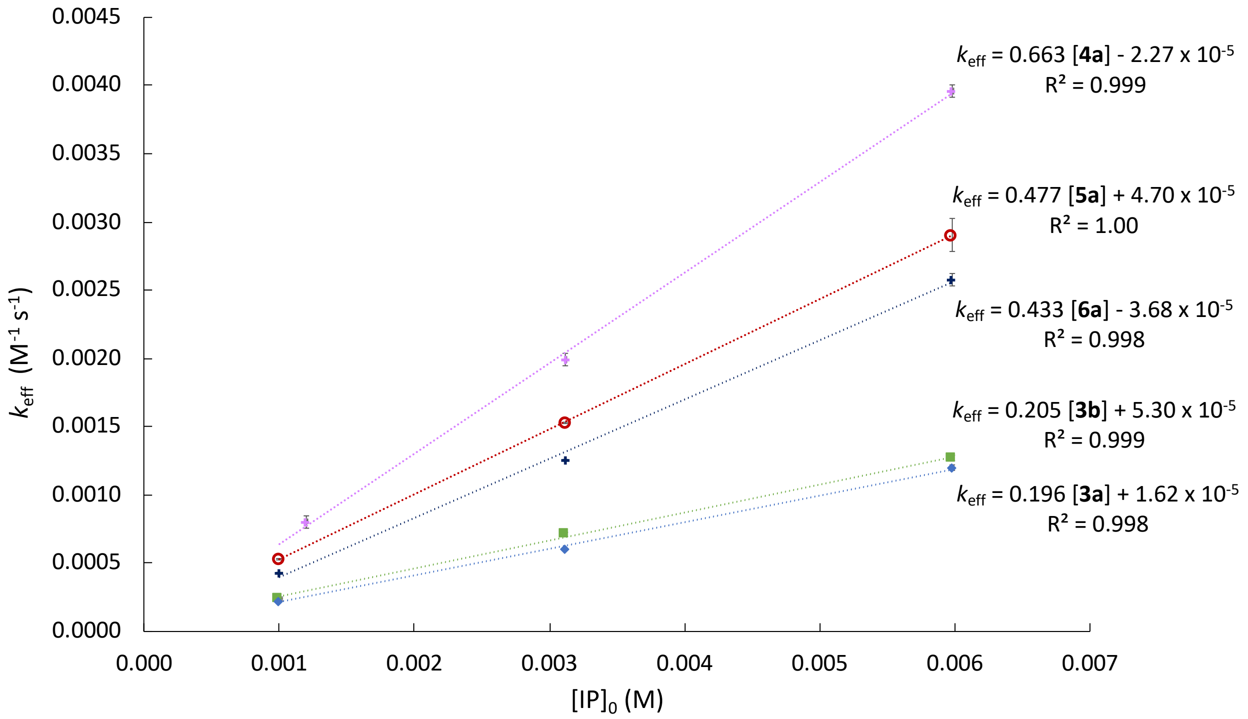

Figure S12. Correlation of catalytic activity at increasing catalyst loadings for the urethane synthesis with data from ref 20 for **4a**.

The relative catalytic performance of the pyridinamide ion pair is reflected in the slope of the correlation as the concentration-independent rate constant k_{cat} ($M^{-2}\,s^{-1}$).

The fitted values of the effective rate constants k_{eff} by CoPaSi with a catalyst load of 3.0 mol% are summarized in Table S17.

Table S17. List of k_{eff} values obtained for the urethane synthesis (3.0 mol%).

Catalyst	Run	Yield [%]	k_{eff}	Average k_{eff}
**3a**	1	99	6.00×10^{-4}	$6.01 \times 10^{-4} \pm 1.50 \times 10^{-6}$
	2	99	6.03×10^{-4}	
**3b**	1	99	7.30×10^{-4}	$7.15 \times 10^{-4} \pm 1.56 \times 10^{-5}$
	2	99	6.99×10^{-4}	
**5a**	1	98	1.54×10^{-3}	$1.53 \times 10^{-3} \pm 8.03 \times 10^{-6}$
	2	98	1.52×10^{-3}	
**6a**	1	99	1.26×10^{-3}	$1.25 \times 10^{-3} \pm 2.50 \times 10^{-6}$
	2	99	1.25×10^{-3}	

The raw data of the absolute kinetics with 3.0 mol% catalyst load are summarized in Table S18-21.

Table S18. Raw data of absolute kinetic of urethane synthesis with **5a** (3.0 mol%) in $CDCl_3$.

Time (min)	Integ. Limits (ppm)		Integ	Absol.	Integ. Limits (ppm)		Integ	Absol.	Integ. Limits (ppm)		Integ	Absol.	$Conv_{exp}$ (%)
**vb178a**	**Product**				**Educt**				**Product+Educt**				**$Conv_{exp}$**
12	4.08	4.20	1.0	49294.1	3.56	3.71	9.7	476294.3	2.23	2.37	5.86	288770.5	25.6
15	4.09	4.20	1.0	55914.8	3.55	3.71	7.7	430175.9	2.22	2.39	4.88	273068.0	30.7
17	4.07	4.20	1.0	62846.1	3.55	3.70	6.7	423653.9	2.22	2.39	4.34	272685.0	34.6
34	4.07	4.20	1.0	98334.9	3.56	3.70	3.8	376780.0	2.25	2.37	2.68	263525.0	56.0
36	4.08	4.21	1.0	102344.9	3.56	3.69	3.6	372393.1	2.25	2.36	2.57	262921.2	58.4
50	4.07	4.20	1.0	122784.0	3.54	3.71	2.9	355569.3	2.21	2.38	2.18	267832.6	68.8
56	4.06	4.21	1.0	129615.9	3.54	3.70	2.7	350760.2	2.23	2.36	2.05	265973.5	73.1
69	4.07	4.21	1.0	138972.5	3.55	3.72	2.4	336015.9	2.24	2.37	1.89	262415.3	79.4
76	4.07	4.21	1.0	146500.3	3.53	3.71	2.3	337052.4	2.24	2.36	1.82	266753.2	82.4
94	4.00	4.16	1.0	156155.9	3.47	3.64	2.1	330238.6	2.19	2.30	1.71	267455.4	87.6
106	4.06	4.22	1.0	160783.5	3.57	3.70	2.0	325588.1	2.24	2.37	1.67	268897.9	89.7
136	4.07	4.23	1.0	168225.4	3.55	3.71	1.9	320733.0	2.24	2.37	1.60	269475.2	93.6
181	4.08	4.21	1.0	111998.1	3.57	3.71	1.8	205954.2	2.24	2.36	1.57	175523.0	95.7
204	4.07	4.21	1.0	171467.4	3.56	3.71	1.8	313087.9	2.25	2.36	1.55	266295.1	96.6
259	4.06	4.23	1.0	174991.4	3.54	3.71	1.8	315640.4	2.25	2.37	1.54	269731.8	97.3
319	4.06	4.23	1.0	175396.0	3.57	3.70	1.8	315181.3	2.25	2.37	1.54	270341.7	97.3
397	4.07	4.21	1.0	192169.9	3.56	3.71	1.8	347963.4	2.25	2.34	1.53	294564.8	97.9

454	4.06	4.22	1.0	172974.0	3.56	3.70	1.8	311679.1	2.25	2.34	1.54	265550.6	97.7
**vb178b**	**Product**				**Educt**				**Product+Educt**				**$Conv_{exp}$**
10	4.09	4.19	1.0	37893.4	3.56	3.70	11.41	432371.0	2.24	2.36	6.86	259805.7	21.9
13	4.08	4.19	1.0	47001.4	3.54	3.69	9.25	434932.5	2.25	2.35	5.64	265131.7	26.6
23	4.08	4.20	1.0	76538.2	3.56	3.70	5.33	407883.4	2.25	2.35	3.48	266070.6	43.1
42	4.07	4.21	1.0	112354.8	3.55	3.69	3.34	374759.4	2.24	2.36	2.38	267505.2	63.0
50	4.08	4.20	1.0	122211.4	3.56	3.69	3.00	366277.5	2.24	2.37	2.20	268516.7	68.3
72	4.07	4.20	1.0	93844.7	3.56	3.69	2.43	227976.5	2.24	2.35	1.88	176461.3	79.8
82	4.07	4.20	1.0	147132.3	3.55	3.70	2.28	335092.8	2.24	2.36	1.79	263607.9	83.7
118	4.07	4.20	1.0	161421.5	3.56	3.69	2.02	325616.8	2.25	2.36	1.65	265795.5	91.1
144	4.08	4.20	1.0	166541.9	3.54	3.69	1.94	322736.7	2.25	2.34	1.59	265081.5	94.2
173	4.07	4.20	1.0	170024.8	3.55	3.71	1.89	321623.8	2.24	2.34	1.57	267074.8	95.5
206	4.06	4.21	1.0	171642.3	3.56	3.69	1.86	318640.6	2.24	2.34	1.55	266157.9	96.7
244	4.07	4.20	1.0	171051.2	3.55	3.69	1.85	317160.7	2.25	2.34	1.55	264423.0	97.0
265	4.08	4.20	1.0	171900.8	3.56	3.69	1.85	317940.4	2.23	2.34	1.55	266886.2	96.6
323	4.06	4.21	1.0	172777.9	3.56	3.70	1.84	317965.8	2.24	2.35	1.54	266884.4	97.1
386	4.07	4.20	1.0	171132.6	3.56	3.70	1.85	315950.8	2.23	2.35	1.55	265461.2	96.7
448	4.06	4.21	1.0	196983.9	3.56	3.70	1.84	362400.7	2.25	2.34	1.54	302637.4	97.6
509	4.07	4.20	1.0	196566.2	3.55	3.70	1.85	363477.6	2.25	2.33	1.54	302865.7	97.4
570	4.08	4.19	1.0	198972.9	3.54	3.70	1.86	369253.6	2.25	2.35	1.54	307097.3	97.2
654	4.08	4.19	1.0	171821.6	3.55	3.70	1.85	318272.0	2.23	2.35	1.56	267421.0	96.4
728	4.06	4.23	1.0	172803.0	3.55	3.70	1.84	317268.7	2.23	2.35	1.54	266391.1	97.3

Table S19. Raw data of absolute kinetic of urethane synthesis with **6a** (3.0 mol%) in $CDCl_3$.

**Time (min)**	**Integ. Limits (ppm)**		**Integ**	**Absol.**	**Integ. Limits (ppm)**		**Integ**	**Absol.**	**Integ. Limits (ppm)**		**Integ**	**Absol.**	**$Conv_{exp}$ (%)**
**vb179a**	**Product**				**Educt**				**Product+Educt**				**$Conv_{exp}$**
7	4.10	4.19	1.0	21119.5	3.54	3.70	21.03	444081.1	2.24	2.37	12.10	255583.1	12.4
13	4.10	4.19	1.0	38513.7	3.55	3.70	11.13	428476.9	2.24	2.37	6.66	256415.1	22.5
23	4.07	4.20	1.0	64161.0	3.55	3.70	6.35	407122.9	2.25	2.36	4.01	257123.4	37.4
35	4.08	4.20	1.0	88042.4	3.55	3.70	4.39	386299.8	2.25	2.36	2.94	258846.2	51.0
53	4.08	4.20	1.0	112183.9	3.57	3.69	3.25	364042.0	2.26	2.35	2.31	258768.2	65.0
58	4.08	4.20	1.0	116953.6	3.56	3.69	3.07	358822.3	2.25	2.35	2.22	259096.4	67.7
93	4.07	4.20	1.0	146224.2	3.54	3.70	2.33	340964.5	2.25	2.35	1.80	263350.4	83.3
128	4.07	4.20	1.0	154824.2	3.54	3.70	2.05	317812.4	2.25	2.34	1.64	254342.1	91.3

151	4.07	4.20	1.0	160536.5	3.55	3.70	1.95	312903.5	2.25	2.35	1.59	256039.8	94.0
184	4.06	4.20	1.0	168818.8	3.55	3.70	1.86	314146.1	2.25	2.35	1.55	261326.1	96.9
210	4.07	4.22	1.0	171490.6	3.55	3.71	1.83	313455.0	2.26	2.34	1.52	260572.1	98.7
269	4.07	4.20	1.0	170644.1	3.57	3.69	1.79	304736.0	2.25	2.34	1.50	256627.1	99.7
330	4.08	4.20	1.0	170195.9	3.56	3.69	1.78	302412.0	2.25	2.34	1.50	254918.7	100.1
394	4.07	4.21	1.0	174777.5	3.55	3.70	1.77	308676.8	2.26	2.35	1.49	260655.1	100.6
455	4.08	4.21	1.0	169826.6	3.55	3.71	1.77	300827.9	2.25	2.35	1.49	253125.1	100.6
529	4.05	4.21	1.0	173616.0	3.54	3.71	1.76	306288.9	2.25	2.35	1.49	258404.8	100.8
601	4.08	4.20	1.0	173080.7	3.56	3.71	1.77	306534.7	2.24	2.34	1.50	259269.6	100.1
737	4.08	4.22	1.0	174241.5	3.55	3.70	1.77	308299.3	2.25	2.35	1.49	259882.2	100.6
**vb179b**	**Product**				**Educt**				**Product+Educt**				**Conv$_{exp}$**
8	4.08	4.20	1.0	24630.2	3.54	3.71	18.84	464151.3	2.24	2.36	10.57	260311.6	14.2
10	4.09	4.20	1.0	31628.5	3.54	3.71	14.48	457851.2	2.22	2.37	8.28	261910.8	18.1
25	4.07	4.21	1.0	69395.3	3.53	3.71	6.13	425178.0	2.22	2.39	3.81	264609.5	39.3
40	4.08	4.20	1.0	96043.3	3.56	3.71	4.16	399441.7	2.26	2.36	2.73	262339.7	54.9
58	4.08	4.21	1.0	118526.6	3.56	3.71	3.17	376155.2	2.24	2.37	2.21	262533.0	67.7
70	4.08	4.21	1.0	130089.9	3.54	3.71	2.83	367516.1	2.25	2.36	2.02	262345.0	74.4
87	4.08	4.20	1.0	143368.3	3.56	3.71	2.49	357482.1	2.24	2.37	1.85	265717.8	80.9
118	4.07	4.22	1.0	153762.7	3.56	3.71	2.19	335982.7	2.24	2.36	1.69	259428.1	88.9
145	4.08	4.20	1.0	160291.4	3.57	3.69	2.05	328527.5	2.24	2.35	1.62	259755.3	92.6
175	4.07	4.22	1.0	166496.1	3.56	3.71	1.95	325310.9	2.24	2.35	1.57	261136.9	95.6
207	4.07	4.22	1.0	169453.4	3.57	3.71	1.90	322588.5	2.25	2.35	1.53	260054.9	97.7
270	4.04	4.22	1.0	174752.1	3.55	3.72	1.86	324492.4	2.24	2.35	1.51	263627.8	99.4
333	4.05	4.22	1.0	173673.4	3.54	3.70	1.85	320440.0	2.24	2.36	1.51	262786.7	99.1
398	4.06	4.21	1.0	174802.4	3.55	3.70	1.84	322096.1	2.24	2.34	1.50	262549.3	99.9
459	4.07	4.21	1.0	172237.9	3.55	3.70	1.84	317275.6	2.24	2.35	1.50	258657.8	99.9
518	4.07	4.22	1.0	171481.0	3.55	3.71	1.84	315688.2	2.24	2.35	1.50	257702.8	99.8

Table S20. Raw data of absolute kinetic of urethane synthesis with **3a** (3.0 mol%) in $CDCl_3$.

**Time (min)**	**Integ. Limits (ppm)**		**Integ**	**Absol.**	**Integ. Limits (ppm)**		**Integ**	**Absol.**	**Integ. Limits (ppm)**		**Integ**	**Absol.**	**Conv$_{exp}$ (%)**
**vb180a**	**Product**				**Educt**				**Product+Educt**				**Conv$_{exp}$**
8	4.09	4.19	1.0	12106.7	3.51	3.72	37.62	455470.7	2.24	2.37	21.05	254885.6	7.1
23	4.06	4.21	1.0	34660.7	3.53	3.72	12.42	430344.0	2.21	2.38	7.35	254677.0	20.4
44	4.06	4.20	1.0	61201.0	3.54	3.71	6.71	410922.0	2.23	2.37	4.23	258719.7	35.5

59	4.08	4.20	1.0	77216.4	3.53	3.72	5.23	403646.8	2.22	2.36	3.41	263495.9	44.0
70	4.08	4.20	1.0	86814.1	3.55	3.71	4.56	395843.8	2.22	2.38	3.06	265873.9	49.0
96	4.08	4.20	1.0	105104.0	3.54	3.71	3.57	374777.2	2.24	2.36	2.50	263257.0	59.9
127	4.06	4.21	1.0	121294.5	3.54	3.72	2.95	357899.8	2.24	2.36	2.16	261926.2	69.5
191	4.08	4.21	1.0	140897.3	3.54	3.72	2.37	333724.4	2.22	2.37	1.85	261289.1	80.9
246	4.06	4.20	1.0	155473.6	3.54	3.70	2.12	329236.5	2.22	2.36	1.72	267487.1	87.2
311	4.06	4.22	1.0	168453.1	3.54	3.71	1.96	330192.5	2.22	2.35	1.63	274495.2	92.1
375	4.07	4.21	1.0	174820.0	3.55	3.70	1.88	328765.2	2.24	2.35	1.58	276855.8	94.7
428	4.06	4.22	1.0	173903.6	3.54	3.72	1.84	320645.1	2.22	2.35	1.57	272185.2	95.8
490	4.06	4.23	1.0	175021.6	3.55	3.72	1.80	315648.3	2.22	2.35	1.54	269905.5	97.3
549	4.07	4.21	1.0	174387.5	3.54	3.73	1.80	313152.8	2.22	2.36	1.54	268564.2	97.4
610	4.08	4.21	1.0	173563.7	3.56	3.71	1.79	310251.8	2.22	2.35	1.54	266766.7	97.6
676	4.08	4.21	1.0	208267.6	3.56	3.71	1.78	371527.8	2.24	2.35	1.52	317593.6	98.4
737	4.07	4.22	1.0	198501.8	3.57	3.71	1.77	352000.2	2.23	2.36	1.53	303099.3	98.2
855	4.08	4.22	1.0	201673.4	3.55	3.71	1.77	357559.8	2.23	2.35	1.52	307452.2	98.4
**vb180b**	**Product**				**Educt**				**Product+Educt**				**Conv$_{exp}$**
8	4.10	4.19	1.0	10202.0	3.55	3.70	43.50	443824.4	2.24	2.37	23.32	237942.0	6.4
21	4.09	4.19	1.0	31387.3	3.56	3.69	14.21	445884.5	2.24	2.36	7.99	250754.4	18.8
31	4.07	4.21	1.0	45353.7	3.55	3.70	9.66	438069.9	2.23	2.36	5.60	254067.6	26.8
35	4.08	4.20	1.0	49194.4	3.55	3.71	8.85	435173.7	2.24	2.36	5.17	254116.6	29.0
56	4.08	4.20	1.0	46588.9	3.56	3.70	5.70	265557.5	2.22	2.37	3.53	164491.9	42.5
71	4.08	4.20	1.0	54869.3	3.57	3.70	4.71	258373.3	2.24	2.36	3.01	165081.9	49.9
97	4.08	4.21	1.0	67253.2	3.56	3.71	3.70	248698.5	2.24	2.36	2.47	166118.5	60.7
112	4.09	4.20	1.0	71585.6	3.55	3.71	3.37	241389.1	2.22	2.36	2.31	165109.7	65.0
148	4.07	4.21	1.0	127575.3	3.56	3.71	2.82	359489.7	2.23	2.36	2.01	256172.2	74.7
173	4.08	4.21	1.0	152312.3	3.54	3.72	2.60	396249.6	2.22	2.36	1.89	288615.0	79.2
211	4.08	4.21	1.0	144549.4	3.56	3.70	2.36	340705.2	2.22	2.36	1.78	256707.3	84.5
237	4.07	4.20	1.0	150186.8	3.54	3.71	2.25	337989.8	2.23	2.35	1.71	256934.8	87.7
270	4.09	4.20	1.0	152319.3	3.57	3.70	2.16	328817.8	2.22	2.36	1.67	254604.0	89.7
290	4.07	4.22	1.0	155610.2	3.55	3.71	2.10	327249.1	2.23	2.36	1.63	254297.4	91.8
320	4.07	4.21	1.0	157869.5	3.55	3.71	2.05	324154.9	2.22	2.37	1.61	254235.3	93.1
356	4.08	4.21	1.0	163634.7	3.55	3.71	2.00	328020.6	2.22	2.35	1.59	259391.6	94.6
415	4.08	4.21	1.0	179040.6	3.56	3.71	1.97	352363.3	2.22	2.36	1.56	278514.3	96.4
472	4.07	4.21	1.0	189082.0	3.55	3.71	1.93	364100.1	2.23	2.35	1.54	290916.0	97.5
533	4.07	4.22	1.0	163454.0	3.55	3.72	1.90	310119.0	2.23	2.35	1.52	248915.8	98.5
593	4.06	4.21	1.0	166964.1	3.58	3.71	1.87	312704.2	2.23	2.35	1.52	253385.2	98.8
655	4.07	4.21	1.0	165202.8	3.56	3.71	1.88	309988.3	2.23	2.35	1.51	250084.6	99.1

716	4.08	4.21	1.0	166852.0	3.55	3.71	1.88	312914.5	2.23	2.36	1.52	252851.0	99.0
777	4.09	4.21	1.0	165015.3	3.56	3.71	1.87	309295.2	2.22	2.36	1.52	250642.9	98.8
898	4.07	4.21	1.0	166530.3	3.55	3.70	1.87	310898.4	2.23	2.36	1.51	251766.7	99.2
1019	4.09	4.20	1.0	111250.7	3.56	3.70	1.87	207821.0	2.23	2.35	1.51	168346.4	99.1

Table S21. Raw data of absolute kinetic of urethane synthesis with **3b** (3.0 mol%) in $CDCl_3$.

Time (min)	Integ. Limits (ppm)		Integ	Absol.	Integ. Limits (ppm)		Integ	Absol.	Integ. Limits (ppm)		Integ	Absol.	$Conv_{exp}$ (%)
**vb181a**	**Productu**				**Educt**				**Product+Educt**				$Conv_{exp}$
10	4.09	4.19	1.0	22448.5	3.55	3.69	24.33	546069.3	2.23	2.35	13.25	297484.3	11.3
19	4.09	4.19	1.0	42333.5	3.55	3.70	12.49	528830.4	2.23	2.37	7.07	299283.2	21.2
31	4.08	4.20	1.0	62820.1	3.55	3.70	8.13	510560.6	2.22	2.37	4.79	300876.9	31.3
46	4.08	4.20	1.0	86236.3	3.55	3.71	5.68	490049.6	2.22	2.36	3.51	302259.1	42.8
61	4.06	4.20	1.0	104143.7	3.56	3.71	4.54	472966.0	2.22	2.37	2.91	303300.2	51.5
78	4.09	4.20	1.0	70978.8	3.55	3.71	3.80	269475.2	2.23	2.36	2.52	179064.3	59.5
99	4.08	4.21	1.0	80825.3	3.56	3.70	3.24	261786.2	2.22	2.36	2.23	180470.6	67.2
133	4.08	4.20	1.0	90538.9	3.55	3.70	2.71	245261.7	2.23	2.36	1.95	176774.5	76.8
161	4.07	4.21	1.0	98101.9	3.56	3.70	2.47	241854.5	2.23	2.36	1.83	179056.8	82.2
192	4.07	4.22	1.0	104311.6	3.55	3.70	2.29	238487.5	2.23	2.35	1.73	180480.5	86.7
225	4.08	4.21	1.0	108755.7	3.55	3.70	2.16	235182.8	2.21	2.36	1.67	181717.2	89.8
253	4.07	4.21	1.0	111600.7	3.56	3.71	2.09	233003.4	2.22	2.36	1.63	182073.9	91.9
281	4.07	4.21	1.0	113808.2	3.56	3.70	2.03	231558.6	2.23	2.35	1.60	182103.9	93.7
312	4.06	4.22	1.0	113997.5	3.55	3.71	1.99	226852.6	2.22	2.35	1.58	179580.2	95.2
377	4.07	4.23	1.0	116117.7	3.56	3.70	1.94	224689.5	2.23	2.36	1.55	179839.1	96.9
438	4.08	4.21	1.0	117570.3	3.55	3.70	1.92	225247.5	2.23	2.34	1.54	180721.1	97.6
498	4.07	4.20	1.0	114485.8	3.56	3.70	1.90	218083.3	2.23	2.35	1.53	175251.6	98.0
553	4.07	4.21	1.0	114461.0	3.56	3.70	1.89	216567.8	2.22	2.36	1.53	174887.5	98.2
615	4.07	4.21	1.0	115384.7	3.56	3.70	1.89	217794.2	2.22	2.35	1.52	175794.7	98.5
675	4.06	4.22	1.0	114030.0	3.56	3.71	1.88	214640.1	2.22	2.35	1.52	172944.6	98.9
**vb181b**	**Productu**				**Educt**				**Product+Educt**				$Conv_{exp}$
7	4.09	4.19	1.0	13710.0	3.56	3.71	34.08	467245.9	2.24	2.37	19.65	269351.0	7.6
23	4.09	4.20	1.0	41798.3	3.55	3.71	10.55	440800.3	2.25	2.36	6.44	269355.0	23.3
43	4.09	4.20	1.0	46273.2	3.53	3.71	5.88	271873.6	2.23	2.36	3.85	177993.9	39.0
58	4.09	4.19	1.0	87975.9	3.56	3.69	4.59	403953.5	2.22	2.37	3.14	276296.3	47.8
79	4.08	4.20	1.0	106883.8	3.55	3.71	3.60	384683.5	2.23	2.36	2.57	275093.8	58.3

108	4.08	4.20	1.0	123349.0	3.57	3.71	2.88	355121.5	2.23	2.36	2.17	268172.1	69.0
139	4.07	4.22	1.0	138162.9	3.55	3.71	2.47	341350.7	2.23	2.36	1.94	268601.5	77.2
167	4.08	4.20	1.0	146150.0	3.56	3.70	2.25	328996.5	2.22	2.36	1.83	266786.6	82.2
195	4.07	4.21	1.0	155650.7	3.57	3.71	2.09	324777.0	2.23	2.36	1.73	269502.7	86.6
215	4.07	4.21	1.0	160956.8	3.56	3.71	2.02	324641.0	2.22	2.37	1.70	272898.2	88.5
257	4.08	4.21	1.0	164760.7	3.56	3.71	1.91	314618.1	2.22	2.36	1.63	269276.9	91.8
275	4.07	4.21	1.0	165753.8	3.56	3.71	1.88	311138.6	2.22	2.36	1.62	267854.9	92.8
318	4.08	4.22	1.0	199454.8	3.55	3.70	1.82	362315.5	2.23	2.35	1.58	315214.5	94.9
379	4.07	4.21	1.0	171945.1	3.56	3.71	1.76	303430.8	2.22	2.36	1.55	267060.1	96.6
441	4.08	4.20	1.0	202047.0	3.54	3.70	1.74	352121.4	2.22	2.37	1.54	311594.4	97.3
502	4.07	4.20	1.0	202205.8	3.55	3.71	1.73	349564.1	2.20	2.35	1.53	310244.9	97.8
579	4.07	4.21	1.0	204730.1	3.55	3.71	1.71	350597.1	2.22	2.35	1.52	311548.7	98.6
624	4.06	4.21	1.0	203172.8	3.56	3.71	1.71	347389.4	2.21	2.36	1.52	309359.7	98.5
684	4.06	4.22	1.0	202490.7	3.56	3.71	1.71	345301.6	2.22	2.36	1.52	307293.7	98.8
746	4.07	4.21	1.0	203387.3	3.56	3.71	1.71	346977.5	2.22	2.35	1.52	308443.1	98.9

The absolute kinetics of the urethane reaction were done with 6.0 mol% catalyst. The results are summarized in Table S22.

Table S22. List of k_{eff} values obtained for the urethane synthesis (6.0 mol%).

**Catalyst**	**Run**	**Yield [%]**	$\boldsymbol{k_{eff}}$	Average $\boldsymbol{k_{eff}}$
**3a**	1	99	1.17×10^{-3}	$1.20 \times 10^{-3} \pm 2.19 \times 10^{-5}$
	2	99	1.22×10^{-3}	
**3b**	1	99	1.25×10^{-3}	$1.27 \times 10^{-3} \pm 1.61 \times 10^{-5}$
	2	99	1.29×10^{-3}	
**5a**	1	94	2.78×10^{-3}	$2.90 \times 10^{-3} \pm 1.20 \times 10^{-4}$
	2	94	3.02×10^{-3}	
**6a**	1	99	2.62×10^{-3}	$2.58 \times 10^{-3} \pm 4.34 \times 10^{-5}$
	2	99	2.53×10^{-3}	

The raw data of absolute kinetics with 6.0 mol% catalyst are summarized in Table S23-26**.**

Table S23. Raw data of absolute kinetic of urethane synthesis with **5a** (6.0 mol%) in $CDCl_3$.

**Time (min)**	**Integ. Limits (ppm)**		**Integ**	**Absol.**	**Integ. Limits (ppm)**		**Integ**	**Absol.**	**Integ. Limits (ppm)**		**Integ**	**Absol.**	**Conv$_{exp}$ (%)**
**Vb148a**	**Product**				**Edukt**				**Product+Educt**				**Conv$_{exp}$**
8	4.09	4.18	1.0	29185.0	3.56	3.69	9.30	271306.7	2.24	2.35	5.69	166208.1	26.3

10	4.09	4.18	1.0	59227.1	3.56	3.71	6.98	413205.8	2.24	2.36	4.40	260880.1	34.1
12	4.08	4.19	1.0	71187.4	3.54	3.70	5.66	402809.9	2.23	2.37	3.68	262096.6	40.7
17	4.09	4.19	1.0	58203.6	3.55	3.69	4.25	247297.0	2.23	2.35	2.89	168302.2	51.9
22	4.08	4.19	1.0	69779.6	3.56	3.68	3.41	238296.2	2.23	2.35	2.43	169707.5	61.7
27	4.08	4.20	1.0	78221.0	3.55	3.70	2.96	231383.6	2.23	2.35	2.17	169958.2	69.0
45	4.07	4.20	1.0	148303.5	3.56	3.70	2.23	330735.0	2.24	2.35	1.76	261273.4	85.1
49	4.07	4.20	1.0	150486.5	3.55	3.70	2.18	328273.0	2.23	2.34	1.73	261053.3	86.5
57	4.07	4.21	1.0	155900.6	3.56	3.69	2.08	324180.4	2.24	2.35	1.68	261781.6	89.3
67	4.06	4.20	1.0	160640.7	3.56	3.69	2.01	322507.9	2.24	2.34	1.64	263201.6	91.6
87	4.07	4.21	1.0	163389.5	3.55	3.69	1.95	317842.8	2.24	2.35	1.60	261982.7	93.5
97	4.07	4.21	1.0	164771.4	3.56	3.69	1.93	317761.2	2.24	2.35	1.60	263296.9	93.9
114	4.07	4.20	1.0	164387.3	3.56	3.70	1.93	316867.2	2.23	2.35	1.60	263070.8	93.7
137	4.06	4.22	1.0	166393.9	3.55	3.70	1.91	318151.6	2.23	2.35	1.59	264277.0	94.4
205	4.07	4.20	1.0	166060.0	3.54	3.69	1.92	318604.0	2.23	2.35	1.60	264971.5	94.0
232	4.07	4.20	1.0	165119.6	3.57	3.69	1.92	316459.3	2.24	2.34	1.59	261878.0	94.6
259	4.08	4.20	1.0	165620.4	3.55	3.70	1.92	317924.4	2.24	2.34	1.59	263065.6	94.4
292	4.08	4.20	1.0	165821.4	3.56	3.70	1.92	318132.1	2.24	2.34	1.59	263490.2	94.4
**Vb148b**	**Product**				**Edukt**				**Product+Educt**				**Conv$_{exp}$**
8	4.08	4.19	1.0	47747.8	3.56	3.70	8.68	414514.0	2.25	2.37	5.43	259419.5	27.6
19	4.07	4.20	1.0	103050.3	3.56	3.69	3.59	369901.9	2.25	2.35	2.56	263556.5	58.6
22	4.07	4.20	1.0	110270.1	3.56	3.69	3.30	363801.4	2.25	2.35	2.39	264007.5	62.7
24	4.09	4.19	1.0	115854.2	3.57	3.70	3.09	358255.6	2.24	2.35	2.28	264336.0	65.7
26	4.07	4.20	1.0	121937.8	3.57	3.69	2.90	353083.0	2.23	2.35	2.17	264921.5	69.0
36	4.08	4.20	1.0	139135.3	3.56	3.70	2.42	336126.5	2.23	2.35	1.90	263977.5	79.1
38	4.07	4.20	1.0	142251.9	3.58	3.69	2.34	332825.0	2.24	2.35	1.85	263857.6	80.9
48	4.08	4.20	1.0	152991.0	3.56	3.70	2.15	328785.7	2.23	2.35	1.75	267110.8	85.9
63	4.07	4.20	1.0	159359.0	3.56	3.69	1.99	317805.2	2.24	2.34	1.65	263423.6	90.7
73	4.07	4.20	1.0	162087.7	3.57	3.69	1.94	315235.1	2.24	2.34	1.62	263239.7	92.4
88	4.08	4.20	1.0	164490.2	3.57	3.69	1.91	314633.4	2.25	2.35	1.61	264561.5	93.3
98	4.08	4.20	1.0	165074.1	3.57	3.69	1.90	313714.9	2.23	2.33	1.60	264202.9	93.7
131	4.08	4.20	1.0	166136.5	3.57	3.69	1.88	313109.0	2.24	2.33	1.59	264379.8	94.3
161	4.09	4.20	1.0	167512.0	3.57	3.69	1.89	316039.8	2.25	2.33	1.59	266051.2	94.4
190	4.09	4.19	1.0	167308.2	3.55	3.68	1.89	315590.2	2.24	2.33	1.59	266756.4	94.1
221	4.06	4.20	1.0	168539.3	3.56	3.69	1.87	315961.5	2.25	2.33	1.58	266660.7	94.8
277	4.08	4.19	1.0	166523.6	3.56	3.69	1.89	313924.4	2.24	2.33	1.59	264813.4	94.3
339	4.06	4.20	1.0	166788.4	3.56	3.70	1.88	313455.6	2.23	2.35	1.59	265520.8	94.2
404	4.07	4.20	1.0	165905.3	3.57	3.70	1.88	311843.9	2.23	2.34	1.59	263533.8	94.4

464	4.08	4.20	1.0	167065.1	3.56	3.70	1.88	314615.3	2.23	2.35	1.60	266675.3	94.0
582	4.08	4.20	1.0	166197.5	3.55	3.69	1.88	313076.0	2.22	2.33	1.59	265011.1	94.1
703	4.08	4.20	1.0	166849.9	3.57	3.69	1.88	313756.0	2.24	2.34	1.59	265064.7	94.4

Table S24. Raw data of absolute kinetic of urethane synthesis with **6a** (6.0 mol%) in $CDCl_3$

Time (min)	Integ. Limits (ppm)		Integ	Absol.	Integ. Limits (ppm)		Integ	Absol.	Integ. Limits (ppm)		Integ	Absol.	$Conv_{exp}$ (%)
**Vb149b**	**Product**				**Edukt**				**Product+Educt**				**$Conv_{exp}$**
8	4.10	4.18	1.0	42807.5	3.53	3.70	9.45	404583.4	2.24	2.35	5.56	238155.5	27.0
11	4.08	4.19	1.0	54341.0	3.56	3.69	7.25	394059.7	2.24	2.36	4.40	238883.1	34.1
20	4.08	4.19	1.0	90368.1	3.55	3.70	4.00	361602.8	2.23	2.37	2.66	240357.1	56.4
23	4.07	4.20	1.0	96708.7	3.55	3.71	3.69	356485.2	2.24	2.37	2.49	240666.5	60.3
25	4.08	4.21	1.0	102716.9	3.56	3.70	3.42	351085.0	2.21	2.36	2.36	242262.4	63.6
36	4.08	4.20	1.0	123801.3	3.55	3.70	2.69	332410.3	2.24	2.36	1.95	241698.4	76.8
38	4.07	4.20	1.0	149671.3	3.55	3.70	2.59	387700.9	2.24	2.36	1.90	284964.1	78.8
49	4.07	4.20	1.0	139718.0	3.56	3.70	2.30	320853.3	2.24	2.36	1.75	244059.1	85.9
51	4.08	4.20	1.0	141802.0	3.56	3.70	2.25	319471.4	2.23	2.36	1.73	244888.7	86.9
54	4.07	4.21	1.0	144471.4	3.56	3.70	2.20	317838.5	2.23	2.35	1.70	244889.4	88.5
68	4.07	4.20	1.0	152312.1	3.56	3.71	2.04	309962.6	2.24	2.35	1.60	244227.6	93.5
78	4.07	4.20	1.0	155162.5	3.57	3.70	1.97	305499.3	2.25	2.34	1.56	242781.7	95.9
93	4.08	4.20	1.0	157954.3	3.56	3.70	1.91	302330.6	2.24	2.34	1.54	243087.0	97.5
95	4.07	4.21	1.0	186571.0	3.57	3.69	1.90	354687.5	2.24	2.34	1.53	285760.7	97.9
111	4.07	4.20	1.0	161009.5	3.57	3.69	1.87	300984.5	2.24	2.34	1.52	243932.6	99.0
132	4.07	4.20	1.0	161771.6	3.56	3.69	1.85	299937.3	2.25	2.34	1.50	243285.0	99.7
160	4.07	4.20	1.0	164362.5	3.56	3.69	1.84	302820.0	2.24	2.33	1.50	246339.4	100.1
187	4.07	4.20	1.0	164685.3	3.56	3.70	1.84	302388.8	2.25	2.34	1.49	245888.3	100.5
245	4.08	4.20	1.0	163030.5	3.56	3.70	1.84	299643.5	2.24	2.34	1.50	244430.7	100.0
**Vb149c**	**Product**				**Edukt**				**Product+Educt**				**$Conv_{exp}$**
10	4.08	4.19	1.0	46050.9	3.55	3.70	8.88	408956.5	2.25	2.36	5.25	241937.7	28.6
21	4.09	4.19	1.0	91277.2	3.57	3.69	4.09	373301.4	2.23	2.35	2.70	246878.5	55.5
23	4.09	4.19	1.0	97854.8	3.57	3.69	3.76	367793.0	2.25	2.35	2.52	246782.4	59.5
25	4.08	4.19	1.0	103814.3	3.56	3.69	3.49	362742.3	2.23	2.35	2.39	247833.5	62.8
27	4.09	4.19	1.0	108987.2	3.56	3.70	3.29	358586.2	2.24	2.35	2.27	247795.3	66.0
38	4.08	4.19	1.0	129136.3	3.57	3.69	2.64	341432.5	2.25	2.35	1.93	249602.9	77.6
48	4.02	4.13	1.0	143426.4	3.51	3.63	2.35	336603.8	2.18	2.29	1.77	254406.5	84.6
50	4.08	4.19	1.0	145282.3	3.58	3.69	2.30	334284.8	2.24	2.34	1.75	253867.0	85.8

57	4.09	4.20	1.0	151155.3	3.58	3.69	2.18	329770.6	2.24	2.34	1.68	254399.9	89.1
74	4.07	4.20	1.0	160457.2	3.57	3.69	2.00	320635.5	2.26	2.33	1.57	252526.9	95.3
90	4.06	4.20	1.0	164379.9	3.57	3.69	1.92	316103.9	2.24	2.33	1.54	252808.0	97.5
108	4.07	4.20	1.0	160969.2	3.57	3.69	1.88	303151.4	2.26	2.33	1.51	242862.9	99.4
117	4.07	4.20	1.0	161798.8	3.57	3.69	1.87	302581.1	2.25	2.33	1.51	243921.6	99.5
158	4.08	4.20	1.0	163303.9	3.58	3.69	1.85	301648.9	2.25	2.33	1.50	244479.4	100.2
177	4.08	4.20	1.0	163460.0	3.57	3.70	1.85	301688.8	2.24	2.33	1.50	245247.6	100.0
210	4.08	4.20	1.0	163795.1	3.56	3.69	1.84	301684.9	2.25	2.33	1.49	244616.0	100.4
240	4.09	4.19	1.0	163494.8	3.58	3.69	1.84	301356.7	2.25	2.33	1.50	244846.4	100.2
299	4.06	4.20	1.0	163912.3	3.56	3.69	1.84	301021.6	2.25	2.33	1.49	244513.7	100.6
363	4.08	4.20	1.0	163587.4	3.58	3.70	1.84	301007.6	2.24	2.33	1.50	244717.4	100.3
425	4.07	4.20	1.0	163892.2	3.58	3.69	1.84	301066.0	2.24	2.33	1.49	244930.8	100.4

Table S25. Raw data of absolute kinetic of urethane synthesis with **3a** (6.0 mol%) in $CDCl_3$.

**Time (min)**	**Integ. Limits (ppm)**		**Integ**	**Absol.**	**Integ. Limits (ppm)**		**Integ**	**Absol.**	**Integ. Limits (ppm)**		**Integ**	**Absol.**	**$Conv_{exp}$ (%)**
**Vb150b**	**Product**				**Edukt**				**Product+Educt**				**$Conv_{exp}$**
7	4.10	4.18	1.0	16975.7	3.57	3.69	23.60	400550.1	2.26	2.35	12.88	218569.1	11.7
9	4.10	4.18	1.0	22743.8	3.56	3.70	17.41	395871.3	2.25	2.36	9.65	219396.1	15.5
14	4.10	4.18	1.0	33728.6	3.57	3.69	11.42	385024.3	2.25	2.36	6.52	219916.2	23.0
19	4.09	4.19	1.0	44866.6	3.56	3.70	8.41	377201.5	2.25	2.36	4.93	221260.3	30.4
24	4.09	4.19	1.0	55034.5	3.57	3.69	6.72	369620.8	2.24	2.36	4.06	223499.5	36.9
29	4.10	4.19	1.0	63898.5	3.56	3.70	5.69	363632.9	2.25	2.35	3.51	224263.4	42.7
34	4.09	4.19	1.0	72009.0	3.56	3.70	4.97	357675.4	2.24	2.36	3.14	226364.2	47.7
51	4.10	4.19	1.0	90964.2	3.56	3.70	3.64	331436.5	2.23	2.35	2.45	222920.0	61.2
60	4.07	4.20	1.0	100193.8	3.56	3.69	3.21	321730.0	2.23	2.35	2.22	222283.8	67.6
64	4.10	4.20	1.0	102327.1	3.56	3.70	3.12	318812.4	2.23	2.37	2.17	222471.7	69.0
84	4.07	4.20	1.0	120684.9	3.55	3.71	2.63	317398.6	2.23	2.35	1.91	230951.6	78.4
112	4.09	4.20	1.0	124247.3	3.57	3.70	2.31	286982.0	2.25	2.35	1.73	215539.6	86.5
141	4.08	4.20	1.0	143192.5	3.57	3.69	2.11	302664.9	2.25	2.35	1.63	233950.3	91.8
172	4.08	4.20	1.0	144498.7	3.56	3.70	2.01	290303.5	2.24	2.35	1.58	228917.6	94.7
205	4.09	4.20	1.0	147750.5	3.57	3.71	1.95	288467.7	2.24	2.35	1.56	230195.8	96.3
235	4.08	4.20	1.0	154688.5	3.57	3.70	1.91	295719.1	2.23	2.35	1.54	238367.9	97.3
359	4.09	4.20	1.0	167525.7	3.56	3.70	1.87	313036.4	2.24	2.34	1.51	253762.2	99.0
420	4.08	4.20	1.0	167099.2	3.56	3.70	1.86	310884.2	2.24	2.33	1.51	251979.5	99.5

482	4.08	4.20	1.0	166607.4	3.56	3.70	1.86	309469.7	2.23	2.33	1.51	251046.4	99.5
533	4.08	4.20	1.0	164303.9	3.57	3.70	1.86	305123.5	2.23	2.33	1.51	247375.5	99.6
599	4.07	4.20	1.0	170235.8	3.57	3.69	1.85	315358.7	2.23	2.34	1.51	256223.6	99.7
659	4.08	4.20	1.0	165639.7	3.57	3.69	1.86	307591.4	2.22	2.34	1.51	250266.8	99.3
**Vb150c**	**Product**				**Edukt**				**Product+Educt**				**Conv$_{exp}$**
6	4.09	4.18	1.0	20151.8	3.53	3.73	24.3	490320.0	2.24	2.37	13.56	273187.3	11.1
16	4.08	4.19	1.0	49860.8	3.55	3.72	9.31	464309.3	2.24	2.37	5.55	276667.0	27.0
18	4.09	4.19	1.0	55754.9	3.54	3.72	8.24	459227.5	2.23	2.38	4.97	277283.7	30.2
20	4.08	4.20	1.0	61729.0	3.56	3.72	7.35	453735.4	2.22	2.39	4.51	278519.4	33.2
30	4.09	4.20	1.0	84272.2	3.55	3.71	5.14	433369.2	2.24	2.36	3.30	278341.5	45.4
33	4.08	4.19	1.0	88920.2	3.54	3.69	4.82	428764.2	2.22	2.35	3.14	279331.4	47.7
35	4.09	4.20	1.0	92922.6	3.55	3.71	4.57	424963.8	2.23	2.38	3.01	280085.7	49.8
41	4.08	4.20	1.0	102791.3	3.56	3.70	4.04	415188.2	2.22	2.37	2.73	280363.1	55.0
53	4.08	4.20	1.0	120675.6	3.57	3.70	3.33	401345.5	2.24	2.36	2.34	281894.8	64.2
64	4.08	4.20	1.0	115425.7	3.56	3.70	2.94	339027.1	2.23	2.36	2.13	245928.0	70.4
84	4.09	4.20	1.0	151515.1	3.57	3.70	2.50	379055.4	2.21	2.36	1.90	287791.9	79.0
94	4.08	4.21	1.0	157817.5	3.56	3.70	2.37	373773.0	2.23	2.36	1.83	288156.3	82.2
126	4.08	4.21	1.0	167218.9	3.55	3.70	2.11	352184.0	2.24	2.35	1.67	280062.2	89.6
157	4.09	4.20	1.0	176820.4	3.57	3.70	1.97	348924.7	2.23	2.35	1.61	284733.3	93.2
188	4.09	4.20	1.0	181696.9	3.57	3.70	1.90	344527.2	2.24	2.36	1.57	284948.4	95.6
220	4.09	4.20	1.0	160988.9	3.57	3.69	1.85	297860.2	2.23	2.35	1.55	248820.9	97.1
289	4.08	4.20	1.0	185655.0	3.57	3.71	1.81	336780.2	2.22	2.35	1.53	283602.1	98.2
334	4.08	4.20	1.0	175196.0	3.56	3.70	1.81	317013.2	2.24	2.34	1.51	264951.1	99.2
394	4.08	4.20	1.0	161682.9	3.57	3.70	1.80	290507.4	2.22	2.35	1.52	245212.9	98.9
454	4.07	4.21	1.0	190082.3	3.54	3.71	1.79	340744.2	2.20	2.34	1.51	287011.4	99.3
514	4.07	4.21	1.0	190528.2	3.57	3.70	1.79	340183.4	2.23	2.36	1.51	287437.7	99.4
576	4.08	4.20	1.0	188852.2	3.57	3.70	1.79	338501.1	2.25	2.34	1.50	283272.4	100.0
644	4.06	4.21	1.0	187939.8	3.57	3.70	1.79	336237.9	2.21	2.35	1.51	283847.0	99.3
705	4.07	4.20	1.0	187200.9	3.56	3.70	1.79	335462.7	2.23	2.35	1.51	282801.5	99.3

Table S26. Raw data of absolute kinetic of urethane synthesis with **3b** (6.0 mol%) in $CDCl_3$.

**Time (min)**	**Integ. Limits (ppm)**		**Integ**	**Absol.**	**Integ. Limits (ppm)**		**Integ**	**Absol.**	**Integ. Limits (ppm)**		**Integ**	**Absol.**	**Conv$_{exp}$ (%)**
**Vb151a**	**Product**				**Edukt**				**Product+Educt**				**Conv$_{exp}$**
7	4.09	4.19	1.0	19017.1	3.55	3.71	23.2	440423.3	2.24	2.36	12.78	243094.3	11.7

9	4.10	4.19	1.0	24022.1	3.55	3.69	18.0	431570.6	2.25	2.36	10.04	241240.6	14.9
12	4.09	4.20	1.0	33803.6	3.54	3.70	12.7	429442.2	2.25	2.36	7.25	245108.3	20.7
17	4.10	4.19	1.0	47243.6	3.56	3.70	8.87	418899.0	2.24	2.37	5.23	247242.9	28.7
27	4.09	4.20	1.0	69016.2	3.56	3.69	5.63	388310.4	2.23	2.36	3.52	243095.8	42.6
29	4.09	4.20	1.0	73232.3	3.56	3.69	5.25	384158.3	2.22	2.37	3.32	243435.6	45.1
32	4.09	4.20	1.0	77101.1	3.57	3.70	4.93	380001.7	2.23	2.36	3.15	242873.2	47.6
42	4.08	4.20	1.0	93920.6	3.55	3.71	3.94	369804.8	2.23	2.37	2.62	246325.2	57.2
52	4.07	4.21	1.0	107040.0	3.56	3.70	3.35	358868.4	2.24	2.37	2.31	247545.6	64.9
54	4.06	4.20	1.0	109431.2	3.55	3.70	3.26	356332.1	2.23	2.35	2.26	247487.5	66.3
62	4.08	4.20	1.0	116738.5	3.56	3.70	2.99	348945.6	2.23	2.35	2.12	247488.2	70.8
72	4.09	4.19	1.0	124082.7	3.54	3.70	2.75	341564.1	2.24	2.36	2.00	247661.1	75.2
87	4.08	4.20	1.0	133953.4	3.56	3.69	2.47	331507.8	2.23	2.35	1.85	247975.8	81.0
102	4.08	4.21	1.0	142432.0	3.56	3.71	2.30	326884.5	2.23	2.36	1.75	249895.3	85.5
134	4.08	4.20	1.0	151650.9	3.55	3.70	2.08	315627.5	2.24	2.35	1.64	248030.3	91.7
195	4.09	4.20	1.0	158526.6	3.57	3.70	1.93	305635.0	2.24	2.34	1.55	246447.5	96.5
222	4.09	4.20	1.0	160994.2	3.55	3.70	1.90	305233.5	2.23	2.35	1.54	248598.5	97.1
257	4.08	4.19	1.0	161266.4	3.57	3.70	1.87	301507.2	2.22	2.35	1.53	246917.1	98.0
282	4.08	4.20	1.0	160862.1	3.57	3.70	1.86	298989.3	2.23	2.35	1.52	244876.0	98.5
346	4.08	4.21	1.0	166365.8	3.55	3.70	1.84	306246.5	2.22	2.34	1.51	251547.2	99.2
407	4.07	4.20	1.0	165204.2	3.57	3.70	1.84	303574.8	2.21	2.35	1.51	250220.0	99.0
467	4.08	4.20	1.0	160596.4	3.56	3.70	1.85	296374.0	2.22	2.35	1.52	243735.0	98.8
528	4.07	4.21	1.0	160677.1	3.56	3.70	1.84	295400.3	2.22	2.34	1.51	242429.7	99.4
590	4.09	4.20	1.0	160363.1	3.57	3.70	1.84	295591.3	2.22	2.35	1.52	243093.3	99.0
651	4.07	4.20	1.0	160307.5	3.56	3.70	1.84	294893.4	2.22	2.34	1.51	241933.9	99.4
711	4.09	4.20	1.0	158890.1	3.56	3.70	1.84	293066.7	2.22	2.34	1.51	240611.7	99.1
**Vb151b**	**Product**				**Edukt**				**Product+Educt**				**Conv$_{exp}$**
11	4.10	4.18	1.0	31178.5	3.55	3.70	13.1	408195.5	2.24	2.36	7.61	237358.2	19.7
13	4.09	4.19	1.0	44174.4	3.56	3.70	10.7	474147.3	2.24	2.36	6.34	279939.4	23.7
21	4.08	4.20	1.0	55885.2	3.54	3.72	6.91	386346.9	2.25	2.37	4.25	237719.6	35.3
31	4.09	4.19	1.0	75614.9	3.57	3.69	4.87	368239.3	2.25	2.35	3.17	239833.4	47.3
40	4.10	4.19	1.0	90430.9	3.56	3.70	3.94	356417.2	2.24	2.35	2.68	242127.6	56.0
43	4.08	4.19	1.0	110242.7	3.57	3.70	3.77	416012.7	2.24	2.35	2.58	284826.3	58.1
45	4.10	4.19	1.0	113582.7	3.58	3.70	3.63	412456.2	2.25	2.35	2.50	283932.8	60.0
47	4.09	4.19	1.0	116688.9	3.57	3.70	3.51	409807.6	2.25	2.35	2.43	283756.2	61.7
63	4.09	4.19	1.0	118778.9	3.58	3.69	2.87	341373.1	2.25	2.35	2.09	248578.6	71.7
65	4.08	4.20	1.0	121231.5	3.57	3.70	2.81	340961.2	2.25	2.35	2.06	249661.6	72.8
80	4.09	4.19	1.0	132074.7	3.57	3.69	2.50	330434.3	2.24	2.34	1.90	250501.5	79.1

112	4.08	4.20	1.0	141949.4	3.57	3.70	2.15	304524.6	2.24	2.35	1.71	242487.1	87.8
122	4.09	4.20	1.0	145845.5	3.58	3.70	2.08	303274.5	2.24	2.35	1.67	243647.6	89.8
132	4.09	4.20	1.0	148250.9	3.57	3.70	2.03	300307.1	2.24	2.35	1.64	243691.2	91.3
147	4.03	4.14	1.0	152969.4	3.51	3.63	1.97	301299.2	2.16	2.28	1.62	247463.7	92.7
177	4.09	4.21	1.0	162094.9	3.57	3.70	1.89	305911.0	2.22	2.35	1.57	255100.8	95.3
201	4.09	4.20	1.0	164280.0	3.57	3.70	1.85	304229.9	2.24	2.34	1.55	254778.2	96.7
237	4.08	4.21	1.0	163332.8	3.57	3.70	1.81	296299.2	2.23	2.34	1.53	249844.7	98.1
263	4.08	4.20	1.0	163736.6	3.57	3.69	1.80	294760.7	2.23	2.34	1.52	249435.8	98.5
296	4.08	4.19	1.0	163723.4	3.57	3.69	1.79	293616.9	2.23	2.33	1.52	248622.2	98.8
357	4.08	4.19	1.0	164008.4	3.57	3.69	1.79	292938.3	2.23	2.34	1.52	248779.3	98.9
419	4.08	4.20	1.0	163654.8	3.58	3.69	1.78	291186.5	2.24	2.33	1.51	246346.5	99.6
487	4.09	4.19	1.0	190682.2	3.57	3.69	1.79	340553.9	2.22	2.34	1.52	289407.1	98.8
548	4.10	4.20	1.0	190691.0	3.58	3.70	1.78	340099.0	2.24	2.33	1.51	287927.7	99.3
599	4.09	4.20	1.0	189898.1	3.58	3.69	1.78	338163.2	2.24	2.33	1.51	286409.2	99.5
665	4.08	4.20	1.0	180069.0	3.58	3.69	1.79	321510.8	2.24	2.33	1.51	271479.6	99.5

The absolute kinetics of the urethane reaction were done with 1.0 mol% catalyst. The results are summarized in Table S27.

Table S27. List of k_{eff} values obtained for the urethane synthesis (1.0 mol%).

**Catalyst**	**Run**	**Yield [%]**	$\boldsymbol{k_{eff}}$	Average $\boldsymbol{k_{eff}}$
**3a**	1	99	2.23×10^{-4}	$2.26 \times 10^{-4} \pm 3.67 \times 10^{-6}$
	2	99	2.30×10^{-4}	
**3b**	1	99	2.43×10^{-4}	$2.45 \times 10^{-4} \pm 1.86 \times 10^{-6}$
	2	99	2.47×10^{-4}	
**5a**	1	97	5.26×10^{-4}	$5.28 \times 10^{-4} \pm 1.95 \times 10^{-6}$
	2	98	5.30×10^{-4}	
**6a**	1	98	4.33×10^{-4}	$4.30 \times 10^{-4} \pm 3.14 \times 10^{-6}$
	2	98	4.27×10^{-4}	

The raw data of absolute kinetics with 1.0 mol% catalyst is summarized in Table S28-31.

Table S28. Raw data of absolute kinetic of urethane synthesis with **5a** (1.0 mol%) in $CDCl_3$.

**Time (min)**	**Integ. Limits (ppm)**		**Integ**	**Absol.**	**Integ. Limits (ppm)**		**Integ**	**Absol.**	**Integ. Limits (ppm)**		**Integ**	**Absol.**	**Conv$_{exp}$ (%)**
**vb229a**	**Product**				**Educt**				**Product+Educt**				**Conv$_{exp}$**
6	4.11	4.19	1.0	9517.4	3.53	3.70	40.8	388705.2	2.22	2.38	23.1	220155.6	6.5

8	4.09	4.20	1.0	12138.0	3.53	3.71	31.9	386786.2	2.23	2.38	18.1	220157.0	8.3
15	4.08	4.19	1.0	19520.3	3.52	3.71	19.5	381095.0	2.22	2.38	11.3	221394.3	13.2
25	4.09	4.19	1.0	29774.7	3.54	3.71	12.5	372002.5	2.24	2.37	7.45	221700.4	20.1
35	4.09	4.20	1.0	39190.5	3.54	3.71	9.28	363859.8	2.24	2.36	5.67	222336.4	26.4
55	4.08	4.20	1.0	57560.0	3.56	3.71	6.18	355654.7	2.22	2.37	3.98	228934.0	37.7
68	4.08	4.20	1.0	67136.0	3.54	3.71	5.20	348930.4	2.20	2.38	3.44	230729.4	43.6
99	4.08	4.20	1.0	84246.7	3.57	3.70	3.83	322627.6	2.24	2.37	2.67	225086.0	56.1
127	4.09	4.20	1.0	96337.5	3.54	3.70	3.26	313659.6	2.22	2.35	2.35	226711.4	63.7
191	4.06	4.20	1.0	117625.6	3.56	3.70	2.51	295219.5	2.23	2.36	1.94	228307.4	77.3
259	4.08	4.22	1.0	130258.4	3.56	3.69	2.18	283687.6	2.21	2.35	1.76	229818.7	85.0
319	4.08	4.21	1.0	135787.0	3.57	3.71	2.03	275873.0	2.22	2.37	1.68	228165.2	89.3
373	4.07	4.22	1.0	143944.6	3.53	3.70	1.95	280740.1	2.21	2.35	1.63	235150.3	91.8
434	4.05	4.23	1.0	147826.5	3.55	3.71	1.89	279116.4	2.22	2.35	1.60	236226.5	93.9
495	4.01	4.16	1.0	192545.2	3.49	3.64	1.85	356939.0	2.16	2.29	1.58	304056.9	95.0
557	4.06	4.21	1.0	191386.1	3.55	3.71	1.83	350576.0	2.22	2.35	1.57	300045.9	95.7
617	4.06	4.22	1.0	192679.0	3.55	3.71	1.82	350758.9	2.21	2.35	1.56	301332.9	95.9
679	4.07	4.22	1.0	187290.9	3.56	3.70	1.82	340269.2	2.21	2.37	1.56	292418.5	96.1
729	4.06	4.22	1.0	194953.1	3.54	3.70	1.81	352439.1	2.21	2.35	1.55	303145.9	96.5
848	4.07	4.22	1.0	168555.7	3.54	3.72	1.80	303378.6	2.20	2.36	1.55	261692.3	96.6
970	4.08	4.22	1.0	193918.4	3.52	3.71	1.81	350314.5	2.21	2.35	1.55	301052.1	96.6
1090	4.06	4.22	1.0	169184.6	3.56	3.72	1.79	303395.9	2.23	2.37	1.54	261305.8	97.1
1216	4.08	4.21	1.0	194798.6	3.56	3.70	1.80	350549.1	2.21	2.35	1.55	302558.9	96.6
1333	4.05	4.23	1.0	194271.0	3.55	3.70	1.79	348164.4	2.18	2.35	1.55	300832.1	96.9
1453	4.02	4.14	1.0	165813.2	3.50	3.64	1.80	298902.8	2.15	2.28	1.55	257828.9	96.5
1574	4.05	4.23	1.0	167347.2	3.53	3.70	1.79	300342.3	2.20	2.35	1.55	259004.0	96.9
**vb229b**	**Product**				**Educt**				**Product+Educt**				**Conv$_{exp}$**
6	4.10	4.18	1.0	11351.0	3.55	3.70	44.6	506743.2	2.26	2.37	24.6	278814.4	6.1
22	4.09	4.18	1.0	33760.8	3.56	3.71	14.3	484114.4	2.24	2.37	8.29	279928.3	18.1
24	4.03	4.14	1.0	36790.5	3.49	3.64	13.1	481479.0	2.16	2.30	7.63	280714.8	19.7
44	4.09	4.20	1.0	60773.0	3.54	3.71	7.63	463442.3	2.23	2.37	4.66	283386.5	32.2
57	4.08	4.20	1.0	73048.7	3.55	3.70	6.19	451881.5	2.25	2.36	3.88	283077.4	38.7
68	4.08	4.20	1.0	83214.3	3.56	3.70	5.30	441260.0	2.24	2.36	3.40	283230.3	44.1
96	4.08	4.20	1.0	102807.1	3.56	3.70	4.00	410721.3	2.23	2.36	2.70	277781.6	55.5
124	4.08	4.20	1.0	119550.8	3.55	3.70	3.37	402306.3	2.23	2.37	2.37	282841.0	63.4
188	4.07	4.20	1.0	142930.0	3.57	3.70	2.60	371297.1	2.24	2.36	1.94	277542.2	77.2
247	4.09	4.20	1.0	159996.6	3.56	3.70	2.29	365874.2	2.23	2.35	1.78	284811.7	84.3
313	4.09	4.20	1.0	173349.3	3.56	3.70	2.10	364448.5	2.24	2.35	1.68	290823.4	89.4

367	4.07	4.23	1.0	182233.1	3.58	3.70	2.00	365132.5	2.22	2.36	1.63	296433.5	92.2
428	4.08	4.21	1.0	177320.9	3.57	3.70	1.94	344816.1	2.22	2.35	1.59	282518.9	94.1
488	4.08	4.21	1.0	193534.6	3.57	3.71	1.91	370372.9	2.23	2.35	1.57	304662.8	95.3
549	4.08	4.21	1.0	179373.8	3.56	3.71	1.89	339445.0	2.22	2.34	1.56	280462.2	95.9
609	4.08	4.23	1.0	180607.5	3.56	3.70	1.87	338196.1	2.24	2.34	1.54	278902.5	97.1
671	4.07	4.21	1.0	200253.2	3.56	3.70	1.87	373900.1	2.23	2.35	1.55	310178.3	96.8
733	4.09	4.20	1.0	166837.8	3.57	3.70	1.88	313056.4	2.23	2.36	1.56	259626.1	96.4
854	4.08	4.22	1.0	191221.4	3.56	3.71	1.85	353471.6	2.23	2.35	1.54	295258.7	97.1
968	4.07	4.20	1.0	185569.1	3.56	3.70	1.85	343373.2	2.24	2.35	1.54	285920.4	97.4
1088	4.07	4.21	1.0	186093.2	3.55	3.70	1.85	343617.1	2.22	2.36	1.55	287697.3	97.0
1210	4.08	4.21	1.0	184869.5	3.57	3.70	1.85	341673.6	2.23	2.35	1.54	285308.2	97.2
1330	4.08	4.21	1.0	187723.9	3.55	3.70	1.85	347441.6	2.23	2.35	1.54	289291.1	97.3
1453	4.07	4.20	1.0	189093.3	3.56	3.70	1.85	348977.3	2.22	2.36	1.55	292393.6	97.0

Table S29. Raw data of absolute kinetic of urethane synthesis with **6a** (1.0 mol%) in $CDCl_3$.

Time (min)	Integ. Limits (ppm)		Integ	Absol.	Integ. Limits (ppm)		Integ	Absol.	Integ. Limits (ppm)		Integ	Absol.	$Conv_{exp}$ (%)
**vb230a**	**Product**				**Educt**				**Product+Educt**				**$Conv_{exp}$**
10	4.10	4.19	1.0	12717.5	3.56	3.71	30.7	389892.1	2.24	2.37	17.2	219324.1	8.7
12	4.10	4.19	1.0	14623.3	3.57	3.71	26.4	386709.0	2.24	2.37	15.0	219305.5	10.0
27	4.09	4.19	1.0	28146.7	3.53	3.71	13.4	376967.3	2.23	2.36	7.84	220606.9	19.1
37	4.09	4.19	1.0	36486.6	3.56	3.70	10.1	368385.6	2.23	2.36	6.05	220837.5	24.8
47	4.09	4.20	1.0	43743.4	3.56	3.70	8.28	362292.1	2.23	2.36	5.07	221712.9	29.6
68	4.09	4.20	1.0	57734.8	3.55	3.71	6.09	351826.3	2.22	2.37	3.87	223614.1	38.7
91	4.09	4.20	1.0	73080.3	3.55	3.71	4.80	351038.3	2.23	2.36	3.17	231384.8	47.4
139	4.09	4.20	1.0	89800.4	3.54	3.71	3.54	317837.6	2.23	2.36	2.47	221885.1	60.7
192	4.09	4.20	1.0	108911.6	3.56	3.70	2.87	312683.2	2.23	2.35	2.12	230485.5	70.9
253	4.09	4.20	1.0	122596.8	3.56	3.70	2.47	303139.9	2.23	2.36	1.90	232604.6	79.1
314	4.08	4.21	1.0	144488.3	3.55	3.71	2.24	323453.0	2.24	2.36	1.77	255242.3	84.9
375	4.07	4.21	1.0	175320.6	3.53	3.70	2.10	367959.8	2.21	2.36	1.69	296739.9	88.6
437	4.07	4.21	1.0	181169.9	3.56	3.71	2.00	363190.6	2.24	2.34	1.64	296264.7	91.7
498	4.09	4.20	1.0	178316.0	3.57	3.69	1.95	348539.3	2.22	2.35	1.62	288511.7	92.7
558	4.08	4.21	1.0	162220.6	3.56	3.71	1.90	308028.1	2.23	2.35	1.58	256630.2	94.8
619	4.06	4.21	1.0	165173.9	3.56	3.71	1.87	308235.3	2.22	2.36	1.57	258926.8	95.7
679	4.07	4.21	1.0	167093.2	3.55	3.70	1.85	308296.3	2.22	2.35	1.56	259886.2	96.4

741	4.08	4.21	1.0	192719.8	3.57	3.71	1.84	353746.0	2.23	2.34	1.55	298310.2	96.9
862	4.07	4.21	1.0	168563.7	3.56	3.71	1.81	305401.2	2.20	2.36	1.54	260055.1	97.2
971	4.09	4.20	1.0	168732.3	3.56	3.70	1.81	305452.1	2.23	2.34	1.54	259170.5	97.7
1098	4.08	4.21	1.0	187166.4	3.56	3.70	1.81	338226.9	2.21	2.36	1.53	287162.9	97.8
1215	4.07	4.20	1.0	191171.5	3.55	3.71	1.80	344999.3	2.21	2.36	1.53	292980.4	97.9
1337	4.09	4.21	1.0	167458.7	3.56	3.70	1.80	301536.6	2.24	2.35	1.53	255733.8	98.2
1457	4.08	4.21	1.0	167998.9	3.57	3.70	1.80	301697.7	2.23	2.36	1.53	256338.7	98.3
1572	4.08	4.23	1.0	168468.5	3.55	3.71	1.80	302612.6	2.23	2.36	1.52	256859.3	98.4
**vb230b**	**Product**				**Educt**				**Product+Educt**				**Conv$_{exp}$**
10	4.09	4.19	1.0	15012.1	3.54	3.70	34.6	519985.9	2.24	2.36	18.7	279992.1	8.0
20	4.09	4.19	1.0	26562.7	3.54	3.71	19.2	510582.9	2.23	2.36	10.6	281736.1	14.1
22	4.09	4.20	1.0	29260.3	3.55	3.71	17.4	508036.7	2.24	2.37	9.63	281826.7	15.6
46	4.09	4.20	1.0	54493.7	3.55	3.71	9.06	493571.4	2.23	2.37	5.28	287875.5	28.4
61	4.08	4.20	1.0	68017.5	3.56	3.71	7.06	480252.4	2.23	2.38	4.24	288588.2	35.4
81	4.09	4.20	1.0	84020.7	3.55	3.71	5.55	466669.3	2.23	2.37	3.45	289635.1	43.5
101	4.09	4.20	1.0	92579.8	3.55	3.71	4.66	431723.3	2.22	2.38	2.98	276115.5	50.3
130	4.08	4.21	1.0	114044.3	3.55	3.71	3.91	445437.8	2.23	2.37	2.58	293915.1	58.2
207	4.08	4.21	1.0	148451.7	3.55	3.71	2.90	430075.1	2.22	2.36	2.05	304527.3	73.1
262	4.08	4.21	1.0	163190.0	3.56	3.72	2.56	417610.8	2.23	2.35	1.87	305370.2	80.2
317	4.07	4.21	1.0	160034.3	3.55	3.71	2.35	375380.8	2.21	2.37	1.76	282243.6	85.1
379	4.08	4.21	1.0	184516.8	3.55	3.71	2.20	406695.6	2.22	2.37	1.69	311842.8	88.8
434	4.05	4.22	1.0	172706.8	3.56	3.72	2.11	364603.0	2.21	2.36	1.64	283014.8	91.5
496	4.06	4.22	1.0	162313.5	3.55	3.71	2.05	332890.6	2.22	2.36	1.61	260917.2	93.3
557	4.06	4.22	1.0	187089.8	3.55	3.71	2.00	373341.2	2.22	2.37	1.58	296022.0	94.8
617	4.08	4.23	1.0	189967.1	3.55	3.71	1.97	374178.8	2.23	2.35	1.57	297376.7	95.8
676	4.06	4.22	1.0	163587.9	3.55	3.72	1.94	317985.1	2.23	2.36	1.55	253500.9	96.8
738	4.07	4.22	1.0	185517.8	3.55	3.71	1.93	358265.9	2.23	2.36	1.54	286435.7	97.2
858	4.08	4.20	1.0	188398.7	3.56	3.70	1.92	361114.7	2.21	2.35	1.54	290540.9	97.3
980	4.08	4.21	1.0	186455.2	3.56	3.71	1.91	355971.1	2.22	2.35	1.53	285859.8	97.8
1094	4.07	4.21	1.0	191379.1	3.55	3.71	1.90	363698.2	2.23	2.35	1.53	292415.8	98.2
1216	4.06	4.23	1.0	192969.6	3.55	3.70	1.89	364546.1	2.22	2.34	1.52	294266.7	98.4
1335	4.07	4.21	1.0	191797.7	3.56	3.70	1.90	363491.4	2.22	2.34	1.53	292731.9	98.3
1457	4.08	4.21	1.0	191807.1	3.56	3.71	1.90	363624.4	2.23	2.34	1.53	292889.3	98.2
1575	4.08	4.21	1.0	191224.3	3.56	3.70	1.90	362792.7	2.22	2.35	1.53	292704.3	98.0

Table S30. Raw data of absolute kinetic of urethane synthesis with **3a** (1.0 mol%) in $CDCl_3$.

Time (min)	Integ. Limits (ppm)		Integ	Absol.	Integ. Limits (ppm)		Integ	Absol.	Integ. Limits (ppm)		Integ	Absol.	$Conv_{exp}$ (%)
**vb227a**	**Product**				**Educt**				**Product+Educt**				**$Conv_{exp}$**
5	4.10	4.19	1.0	3929.2	3.53	3.73	113.8	447214.5	2.23	2.38	62.7	246385.3	2.4
28	4.09	4.19	1.0	17551.3	3.53	3.73	24.9	436583.1	2.22	2.37	14.1	248195.5	10.6
33	4.07	4.20	1.0	20512.5	3.54	3.72	21.1	433740.4	2.22	2.38	12.1	248444.0	12.4
48	4.08	4.21	1.0	28371.3	3.54	3.73	15.1	427186.6	2.20	2.38	8.80	249601.0	17.0
63	4.02	4.15	1.0	35704.2	3.48	3.66	11.8	421135.0	2.16	2.32	7.00	249906.2	21.4
95	4.08	4.20	1.0	50425.1	3.53	3.72	8.09	407904.0	2.22	2.37	4.97	250533.3	30.2
128	4.06	4.21	1.0	63311.3	3.53	3.73	6.27	396912.4	2.21	2.38	3.98	251973.5	37.7
186	4.01	4.14	1.0	82268.5	3.47	3.66	4.62	380085.0	2.15	2.30	3.08	253299.2	48.7
246	4.08	4.22	1.0	98353.5	3.56	3.71	3.71	365160.4	2.22	2.38	2.59	254414.6	58.0
307	4.04	4.22	1.0	111236.5	3.54	3.71	3.18	353187.1	2.21	2.37	2.29	254279.2	65.6
369	4.06	4.21	1.0	121736.3	3.53	3.72	2.84	345633.5	2.21	2.36	2.10	256129.5	71.3
429	4.05	4.23	1.0	130674.2	3.52	3.72	2.60	339408.5	2.22	2.36	1.97	257350.0	76.2
491	4.07	4.22	1.0	135646.4	3.55	3.72	2.43	329417.2	2.22	2.37	1.88	254920.5	79.8
546	4.06	4.22	1.0	163320.2	3.54	3.71	2.31	377062.0	2.20	2.35	1.81	295890.3	82.8
606	4.06	4.22	1.0	167817.5	3.53	3.72	2.21	370359.6	2.21	2.36	1.75	294281.8	85.5
665	4.05	4.22	1.0	171460.3	3.56	3.74	2.12	363662.2	2.20	2.36	1.71	292881.4	87.8
727	4.05	4.22	1.0	174760.6	3.54	3.73	2.06	359152.0	2.20	2.37	1.67	292142.7	89.7
846	4.07	4.22	1.0	184868.8	3.53	3.72	1.96	361589.8	2.22	2.36	1.62	299052.1	92.7
966	4.04	4.22	1.0	163046.6	3.53	3.72	1.89	307481.4	2.22	2.36	1.58	257519.2	95.0
1087	4.05	4.23	1.0	163628.3	3.54	3.71	1.85	303077.8	2.21	2.36	1.56	255560.1	96.0
1225	4.06	4.23	1.0	144252.4	3.55	3.71	1.82	263091.8	2.21	2.37	1.55	222920.9	97.1
1330	4.06	4.22	1.0	130121.2	3.56	3.72	1.81	235849.2	2.20	2.37	1.54	200232.6	97.5
1445	4.05	4.22	1.0	143255.7	3.53	3.71	1.79	256651.8	2.21	2.35	1.53	218498.2	98.3
1581	4.06	4.21	1.0	147706.8	3.56	3.72	1.78	263113.4	2.20	2.36	1.53	225327.4	98.3
1812	4.05	4.23	1.0	152622.9	3.55	3.73	1.76	268937.8	2.21	2.36	1.51	230812.0	99.2
1994	4.05	4.24	1.0	193812.7	3.52	3.74	1.76	340360.2	2.20	2.36	1.51	292462.1	99.4
**vb227b**	**Product**				**Educt**				**Product+Educt**				**$Conv_{exp}$**
5	4.09	4.19	1.0	4626.8	3.54	3.72	110.0	508927.5	2.23	2.37	60.3	278789.9	2.5
15	4.10	4.19	1.0	11772.9	3.54	3.71	42.7	503045.3	2.24	2.37	23.7	279468.7	6.3
31	4.10	4.19	1.0	22232.8	3.55	3.72	22.3	494983.7	2.22	2.37	12.7	281709.0	11.8
38	4.09	4.19	1.0	26592.0	3.55	3.72	18.5	491850.0	2.23	2.37	10.6	281921.7	14.1
62	4.08	4.21	1.0	40520.3	3.55	3.71	11.7	474354.7	2.23	2.37	6.91	279981.1	21.7

75	4.08	4.20	1.0	46775.2	3.53	3.72	9.90	462999.8	2.23	2.38	5.93	277256.5	25.3
101	4.09	4.20	1.0	59597.0	3.55	3.72	7.61	453244.9	2.21	2.38	4.69	279380.7	32.0
132	4.08	4.21	1.0	73681.5	3.55	3.71	5.98	440740.6	2.22	2.38	3.80	280151.5	39.5
192	4.08	4.21	1.0	96907.8	3.55	3.70	4.41	427440.1	2.22	2.37	2.95	285618.8	50.9
254	4.08	4.20	1.0	115324.7	3.55	3.72	3.57	412071.3	2.22	2.37	2.49	287263.1	60.2
315	4.07	4.21	1.0	129653.1	3.54	3.73	3.08	399768.3	2.21	2.38	2.22	288414.2	67.4
380	4.06	4.22	1.0	141767.5	3.55	3.72	2.74	388063.4	2.20	2.37	2.04	288697.6	73.7
436	4.08	4.22	1.0	150827.9	3.55	3.72	2.53	381706.2	2.21	2.37	1.92	290172.4	78.0
497	4.06	4.21	1.0	158641.1	3.55	3.72	2.37	375263.0	2.22	2.36	1.83	290672.3	81.9
564	4.05	4.21	1.0	164750.8	3.54	3.72	2.24	368817.1	2.22	2.37	1.76	290283.2	85.1
610	4.06	4.23	1.0	169501.8	3.53	3.72	2.16	366547.8	2.19	2.37	1.73	292417.6	86.9
670	4.06	4.22	1.0	172321.8	3.55	3.72	2.09	360065.6	2.22	2.36	1.68	290019.2	89.1
741	4.06	4.23	1.0	176677.8	3.55	3.72	2.02	357076.6	2.22	2.36	1.64	290111.8	91.3
852	4.08	4.21	1.0	180632.5	3.54	3.72	1.95	353021.1	2.22	2.36	1.61	290736.6	93.2
973	4.06	4.22	1.0	187691.5	3.54	3.72	1.89	354577.1	2.19	2.36	1.58	296268.0	95.0
1093	3.98	4.16	1.0	188788.3	3.49	3.65	1.85	349175.9	2.15	2.31	1.55	292736.2	96.7
1216	4.07	4.21	1.0	191280.7	3.54	3.71	1.83	349770.9	2.22	2.35	1.54	294854.0	97.3
1331	4.06	4.23	1.0	164903.5	3.55	3.71	1.81	297956.7	2.21	2.35	1.53	252277.1	98.0
1457	4.00	4.16	1.0	189227.8	3.48	3.65	1.80	340425.1	2.14	2.30	1.52	287959.3	98.6
1702	4.05	4.23	1.0	191959.9	3.54	3.73	1.78	342190.7	2.22	2.35	1.51	289726.2	99.4
1938	4.05	4.23	1.0	204185.3	3.56	3.73	1.78	362741.5	2.21	2.35	1.51	307796.4	99.5
2178	4.07	4.22	1.0	193553.9	3.55	3.72	1.77	342398.5	2.20	2.37	1.51	292336.0	99.3
2415	4.08	4.23	1.0	183498.1	3.54	3.72	1.78	326171.6	2.21	2.35	1.51	276624.9	99.5

Table S31. Raw data of absolute kinetic of urethane synthesis with **3b** (1.0 mol%) in $CDCl_3$.

Time (min)	Integ. Limits (ppm)		Integ	Absol.	Integ. Limits (ppm)		Integ	Absol.	Integ. Limits (ppm)		Integ	Absol.	$Conv_{exp}$ (%)
**vb228a**	**Product**				**Educt**				**Product+Educt**				**$Conv_{exp}$**
5	4.09	4.20	1.0	4643.0	3.56	3.72	97.3	451622.2	2.23	2.38	54.3	251915.5	2.8
16	4.10	4.19	1.0	11511.3	3.54	3.72	38.8	446083.7	2.23	2.38	21.9	252138.6	6.8
36	4.07	4.20	1.0	24810.5	3.54	3.72	17.5	434843.9	2.23	2.37	10.2	252917.8	14.7
46	4.09	4.19	1.0	29983.2	3.54	3.71	14.3	430233.6	2.22	2.37	8.46	253682.0	17.7
64	4.08	4.20	1.0	39729.8	3.53	3.72	10.6	420722.1	2.23	2.38	6.38	253358.5	23.5
93	4.09	4.19	1.0	53929.7	3.55	3.71	7.59	409242.8	2.23	2.36	4.74	255464.9	31.7
124	4.07	4.21	1.0	67115.5	3.55	3.71	5.92	397304.0	2.22	2.37	3.82	256409.4	39.3

183	4.09	4.21	1.0	87481.8	3.54	3.72	4.33	378391.5	2.21	2.37	2.94	257143.5	51.0
245	4.08	4.21	1.0	105211.2	3.53	3.72	3.47	364603.8	2.22	2.36	2.46	259093.7	60.9
310	4.07	4.21	1.0	118694.6	3.54	3.71	2.96	351170.6	2.23	2.36	2.18	259095.3	68.7
370	4.06	4.21	1.0	129238.8	3.54	3.71	2.65	342491.2	2.22	2.36	2.01	260053.2	74.5
424	4.08	4.21	1.0	134017.1	3.52	3.72	2.47	331499.3	2.21	2.36	1.92	256657.1	78.3
491	4.07	4.22	1.0	165012.6	3.54	3.72	2.29	378695.6	2.23	2.36	1.81	299074.4	82.8
547	4.08	4.22	1.0	168610.0	3.56	3.72	2.19	369352.2	2.21	2.36	1.76	296524.5	85.3
605	4.05	4.23	1.0	173236.0	3.53	3.72	2.10	363621.2	2.21	2.38	1.71	295431.2	88.0
668	4.08	4.21	1.0	176028.0	3.53	3.71	2.04	358521.2	2.22	2.36	1.67	294207.5	89.7
722	4.07	4.22	1.0	182772.0	3.54	3.71	1.98	361608.9	2.21	2.36	1.64	300516.9	91.2
846	4.07	4.22	1.0	164235.8	3.55	3.71	1.89	309895.0	2.23	2.35	1.59	261674.5	94.1
967	4.07	4.21	1.0	167706.1	3.55	3.73	1.84	308744.6	2.21	2.36	1.57	263560.1	95.4
1123	4.08	4.20	1.0	171157.4	3.54	3.71	1.84	314528.2	2.22	2.35	1.55	265132.5	96.8
1204	4.07	4.21	1.0	149226.9	3.53	3.72	1.79	267395.7	2.21	2.36	1.54	229942.0	97.3
1326	4.07	4.21	1.0	152878.8	3.54	3.72	1.77	271070.3	2.21	2.35	1.53	233694.8	98.1
1443	4.07	4.23	1.0	151022.3	3.55	3.73	1.76	265414.1	2.22	2.35	1.52	229133.7	98.9
1564	4.08	4.21	1.0	156143.0	3.56	3.73	1.75	273851.8	2.23	2.36	1.52	237137.1	98.8
1805	4.08	4.22	1.0	195715.1	3.55	3.71	1.75	341838.3	2.21	2.36	1.52	297217.1	98.8
2049	4.07	4.22	1.0	200730.4	3.54	3.71	1.74	348682.8	2.21	2.36	1.51	303278.6	99.3
**vb228b**	**Product**				**Educt**				**Product+Educt**				**Conv$_{exp}$**
5	4.09	4.19	1.0	5184.1	3.52	3.73	102.3	530218.9	2.21	2.39	54.9	284855.9	2.7
9	4.10	4.20	1.0	7770.8	3.54	3.71	67.8	526864.0	2.22	2.37	36.6	284421.7	4.1
19	4.08	4.20	1.0	15263.9	3.54	3.72	34.1	521142.1	2.22	2.38	18.7	285514.3	8.0
36	4.09	4.20	1.0	27814.9	3.55	3.71	18.3	509968.1	2.22	2.36	10.3	286217.6	14.6
49	4.08	4.20	1.0	35653.6	3.53	3.71	14.1	503038.7	2.21	2.38	8.05	286967.7	18.6
72	4.08	4.20	1.0	49189.4	3.55	3.71	10.0	489760.3	2.23	2.36	5.83	287009.3	25.7
96	4.08	4.21	1.0	62427.5	3.55	3.72	7.66	478184.5	2.21	2.37	4.62	288497.5	32.5
126	4.08	4.21	1.0	76661.8	3.51	3.72	6.09	467038.2	2.21	2.37	3.78	289765.2	39.7
191	4.08	4.22	1.0	102749.0	3.55	3.71	4.29	441135.8	2.23	2.38	2.83	290405.4	53.1
246	4.08	4.21	1.0	119491.3	3.55	3.71	3.57	426131.9	2.23	2.37	2.44	291362.9	61.5
307	4.05	4.21	1.0	134573.2	3.54	3.71	3.07	413342.0	2.22	2.36	2.17	292601.7	69.0
366	4.06	4.22	1.0	145942.8	3.54	3.72	2.77	403560.7	2.22	2.36	2.01	293251.2	74.7
427	4.06	4.21	1.0	154296.5	3.55	3.72	2.54	391905.3	2.23	2.36	1.89	291294.1	79.5
488	4.07	4.22	1.0	161117.3	3.55	3.73	2.38	383970.8	2.23	2.37	1.80	290800.9	83.1
549	4.06	4.21	1.0	167574.2	3.55	3.72	2.27	379939.7	2.23	2.36	1.75	292683.2	85.9
607	4.07	4.22	1.0	172069.5	3.57	3.71	2.18	375104.7	2.24	2.36	1.70	292485.1	88.2
667	4.07	4.22	1.0	175248.6	3.54	3.71	2.12	371388.9	2.22	2.36	1.67	292019.7	90.0

727	4.08	4.22	1.0	181721.1	3.55	3.71	2.06	375236.5	2.21	2.36	1.64	298727.3	91.2
846	4.07	4.22	1.0	158597.6	3.56	3.71	1.98	314655.2	2.21	2.37	1.60	253786.1	93.7
968	4.07	4.22	1.0	160980.6	3.55	3.71	1.93	311273.7	2.22	2.36	1.57	252734.1	95.5
1086	4.07	4.21	1.0	163661.5	3.56	3.71	1.90	311255.4	2.23	2.35	1.55	254220.6	96.6
1207	4.06	4.21	1.0	189361.5	3.55	3.71	1.88	356773.6	2.21	2.36	1.54	292054.1	97.3
1328	4.07	4.21	1.0	190116.1	3.56	3.71	1.87	355558.4	2.22	2.36	1.54	291836.0	97.7
1453	4.07	4.22	1.0	191263.9	3.55	3.70	1.86	355621.3	2.23	2.34	1.52	291648.6	98.4
1567	4.07	4.22	1.0	205200.0	3.56	3.71	1.85	380436.2	2.23	2.36	1.52	312490.2	98.5
1807	4.07	4.21	1.0	203826.6	3.54	3.71	1.85	376804.8	2.22	2.34	1.52	309123.3	98.9
2052	4.08	4.21	1.0	194504.9	3.56	3.70	1.84	357286.8	2.23	2.36	1.52	294868.6	98.9
2290	4.07	4.20	1.0	192043.4	3.55	3.71	1.84	352656.8	2.22	2.35	1.52	291384.5	98.9

8. Crystallographic Data

Triphenyl ((triphenyl-λ5-phosphaneylidene)amino) phosphonium pyridin-4-yl (trifluoromethyl) sulfonyl) amide (**3b**)

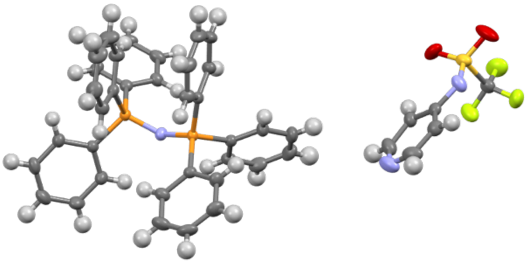

Table S32. Crystallographic details for **3b** obtained with crystallization by precipitation from CH_2Cl_2 overlayered with toluene. Ellipsoids are drawn at 25% probability level.

net formula	$C_{42}H_{34}F_3N_3O_2P_2S$
M_r/g mol^{-1}	763.72
crystal size/mm	0.170 × 0.140 × 0.120
T/K	173.(2)
radiation	MoKα
diffractometer	'Bruker D8 Venture TXS'
crystal system	triclinic
space group	'P -1'
a/Å	10.3726(12)
b/Å	11.4051(12)
c/Å	16.5612(19)
α/°	97.926(4)
β/°	106.882(4)
γ/°	90.907(4)
V/Å^3	1853.6(4)
Z	2
calc. density/g cm^{-3}	1.368
μ/mm^{-1}	0.230
absorption correction	Multi-Scan
transmission factor range	0.93–0.97
refls. measured	32931
R_{int}	0.0504
mean σ(I)/I	0.0487
θ range	2.377–27.485
observed refls.	7124
x, y (weighting scheme)	0.0419, 0.9450
hydrogen refinement	constr
Flack parameter	?
refls in refinement	8480
parameters	478
restraints	0
$R(F_{obs})$	0.0412
$R_w(F^2)$	0.1105
S	1.027
shift/error$_{max}$	0.001
max electron density/e Å^{-3}	0.405
min electron density/e Å^{-3}	−0.367

Tetraphenylphosphonium pyridin-4-yl(tosyl)amide (**5a**)

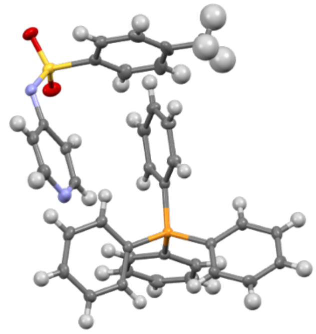

Table S33. Crystallographic details for **5a** obtained with crystallization by precipitation from CH_2Cl_2 overlayered with toluene and heptane. Ellipsoids are drawn at 25% probability level.

net formula	$C_{36}H_{31}N_2O_2PS$
M_r/g mol^{-1}	586.66
crystal size/mm	0.120 × 0.100 × 0.090
T/K	103.(2)
radiation	MoKα
diffractometer	'Bruker D8 Venture TXS'
crystal system	triclinic
space group	'P -1'
a/Å	10.7903(5)
b/Å	12.0043(5)
c/Å	13.0598(5)
α/°	98.1020(10)
β/°	105.4840(10)
γ/°	107.2340(10)
V/Å^3	1511.90(11)
Z	2
calc. density/g cm^{-3}	1.289
μ/mm^{-1}	0.196
absorption correction	Multi-Scan
transmission factor range	0.96–0.98
refls. measured	37059
R_{int}	0.0493
mean σ(I)/I	0.0411
θ range	2.754–28.278
observed refls.	5838
x, y (weighting scheme)	0.0284, 1.1454
hydrogen refinement	constr
Flack parameter	?
refls in refinement	7485
parameters	380
restraints	0
$R(F_{obs})$	0.0429
$R_w(F^2)$	0.1037
S	1.054
shift/error$_{max}$	0.001
max electron density/e Å^{-3}	0.375
min electron density/e Å^{-3}	−0.412

Tetraphenylphosphonium pyridin-4-yl((4-(trifluoromethyl)phenyl)sulfonyl)amide (**6a**)

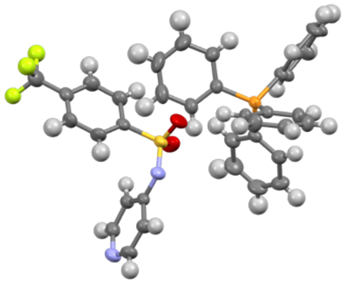

Table S34. Crystallographic details for **6a** obtained with crystallization by precipitation from CH_2Cl_2 overlayered with toluene. Ellipsoids are drawn at 25% probability level.

net formula	$C_{36}H_{28}F_3N_2O_2PS$
M_r/g mol^{-1}	640.63
crystal size/mm	0.110 × 0.050 × 0.040
T/K	173.(2)
radiation	MoKα
diffractometer	'Bruker D8 Venture TXS'
crystal system	triclinic
space group	'P -1'
a/Å	10.5191(6)
b/Å	12.8560(7)
c/Å	14.7858(7)
α/°	66.648(2)
β/°	81.861(2)
γ/°	73.088(2)
V/Å^3	1755.57(16)
Z	2
calc. density/g cm^{-3}	1.212
μ/mm^{-1}	0.186
absorption correction	Multi-Scan
transmission factor range	0.96–0.99
refls. measured	31372
R_{int}	0.0509
mean σ(I)/I	0.0464
θ range	2.861–27.102
observed refls.	5983
x, y (weighting scheme)	0.0792, 1.0326
hydrogen refinement	constr
Flack parameter	?
refls in refinement	7726
parameters	431
restraints	133
$R(F_{obs})$	0.0572
$R_w(F^2)$	0.1681
S	1.044
shift/error$_{max}$	0.001
max electron density/e Å^{-3}	0.393
min electron density/e Å^{-3}	−0.545

Triphenyl((triphenyl-λ5-phosphaneylidene)amino)phosphonium tetrafluoroborate (**7b**)

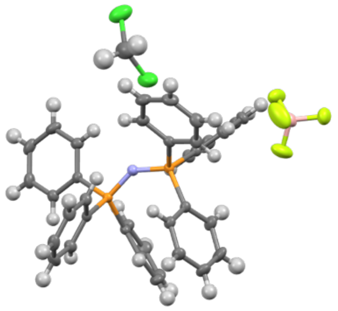

Table S35. Crystallographic details for additive **7b** ($PNPBF_4$ x CH_2Cl_2) obtained with crystallization by precipitation from CH_2Cl_2 overlayered with toluene. Ellipsoids are drawn at 25% probability level.

net formula	$C_{37}H_{32}BCl_2F_4NP_2$
M_r/g mol^{-1}	710.28
crystal size/mm	0.100 × 0.060 × 0.050
T/K	173.(2)
radiation	MoKα
diffractometer	'Bruker D8 Venture TXS'
crystal system	triclinic
space group	'P -1'
a/Å	9.5185(5)
b/Å	10.6730(6)
c/Å	17.0550(9)
α/°	90.353(2)
β/°	94.638(2)
γ/°	93.483(2)
V/Å^3	1723.66(16)
Z	2
calc. density/g cm^{-3}	1.369
μ/mm^{-1}	0.331
absorption correction	Multi-Scan
transmission factor range	0.95–0.98
refls. measured	29933
R_{int}	0.0464
mean σ(I)/I	0.0408
θ range	2.965–26.372
observed refls.	6153
x, y (weighting scheme)	0.0362, 1.2966
hydrogen refinement	constr
Flack parameter	?
refls in refinement	7035
parameters	424
restraints	0
$R(F_{obs})$	0.0398
$R_w(F^2)$	0.1016
S	1.011
shift/error$_{max}$	0.001
max electron density/e Å^{-3}	0.399
min electron density/e Å^{-3}	−0.638

The X-ray crystal structure of compound **7a** has previously been reported (without the DCM solvate).[22–24]

9. NMR Spectra

The recorded NMR spectra of compounds **3a** and **7a** were identical to the ones published in ref. 2. The recorded NMR spectra of compounds PA_**3**, PA_**4**, PA_**5**, **3c**, **3d**, and **4a** were identical to the ones published in ref. 3.

N-(pyridin-4(1*H*)-ylidene)-4-(trifluoromethyl)benzenesulfonamide (PA_**6**)

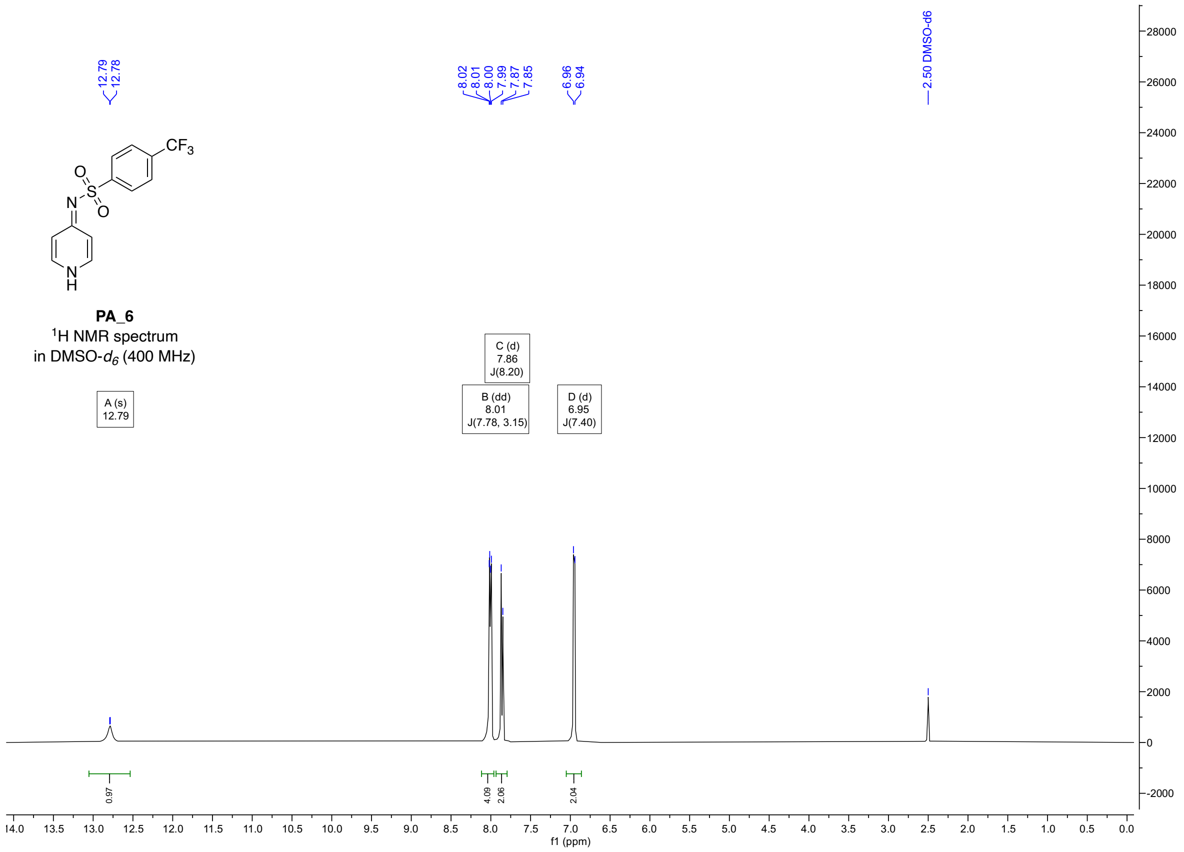

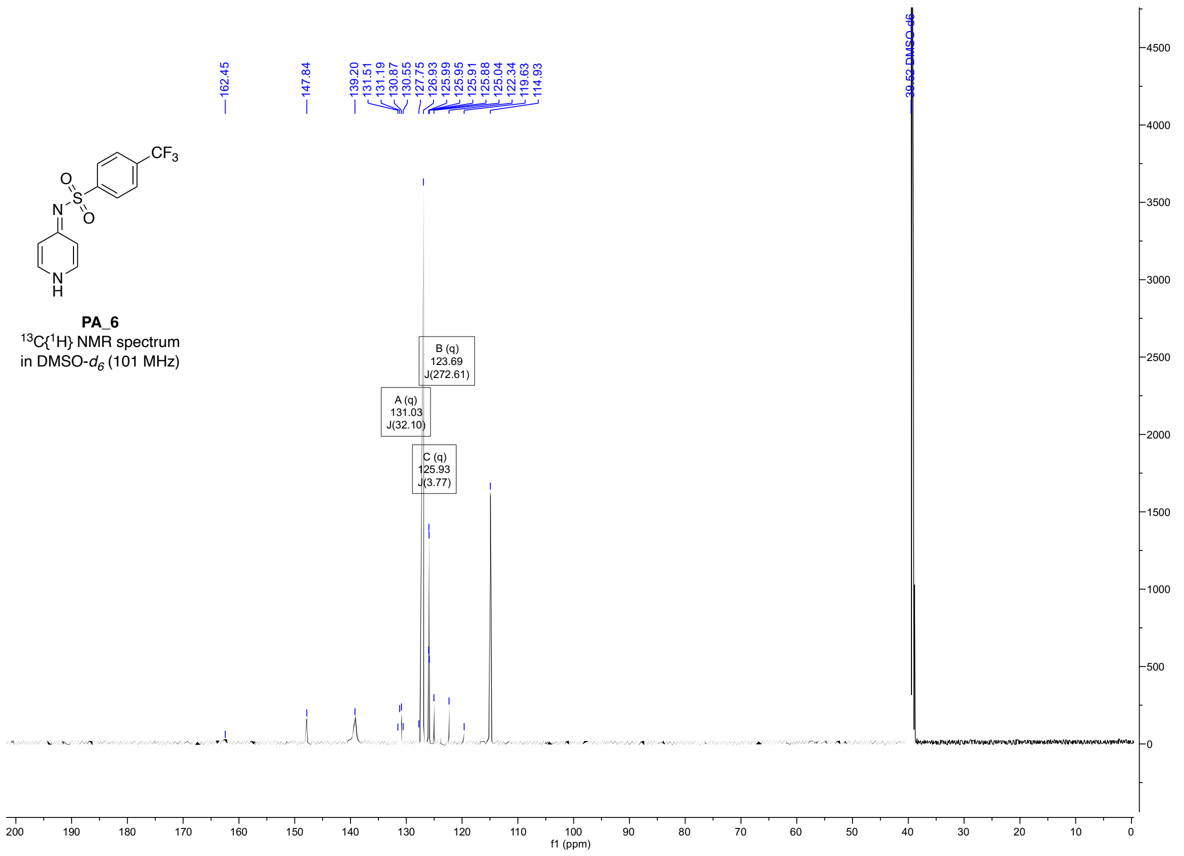

PA_6
$^{13}C\{^{1}H\}$ NMR spectrum
in DMSO-d_6 (101 MHz)

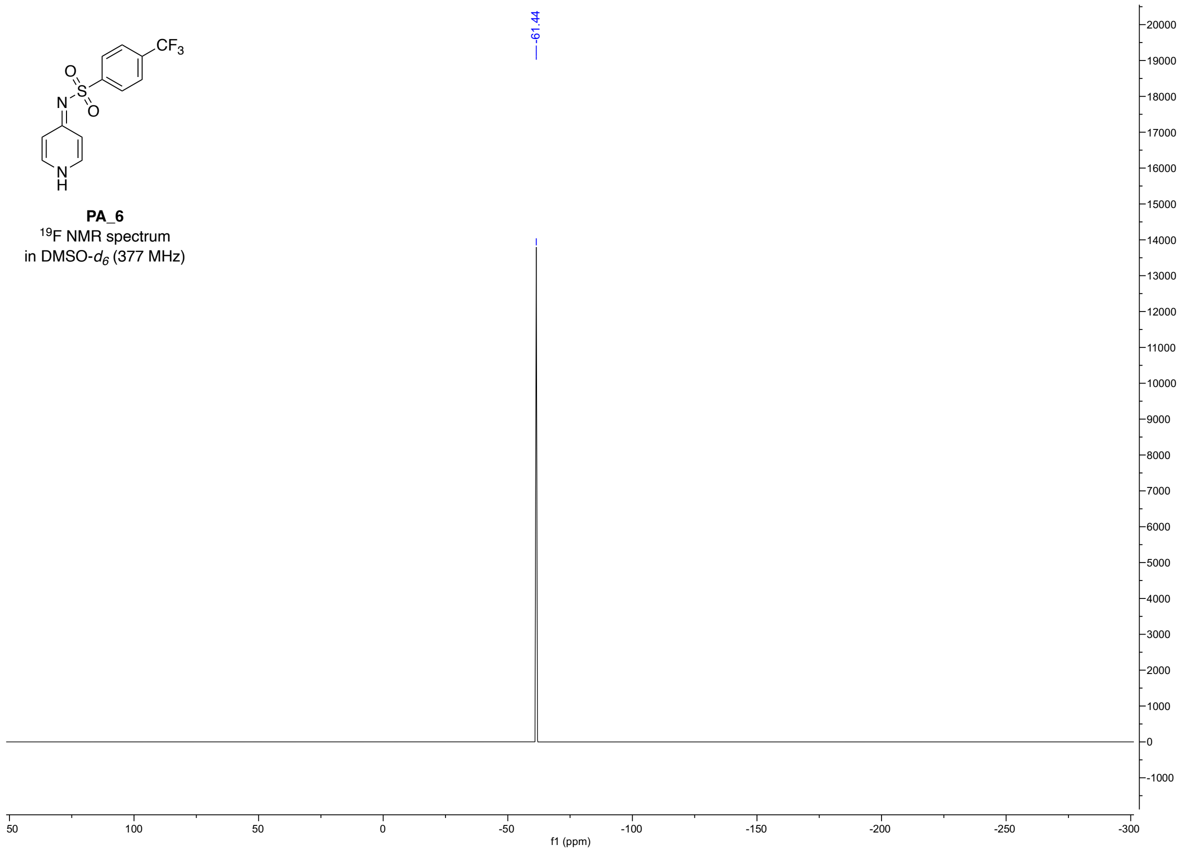

PA_6
^{19}F NMR spectrum
in DMSO-d_6 (377 MHz)

Triphenyl ((triphenyl-λ5-phosphaneylidene)amino) phosphonium pyridin-4-yl (trifluoromethyl) sulfonyl) amide (**3b**)

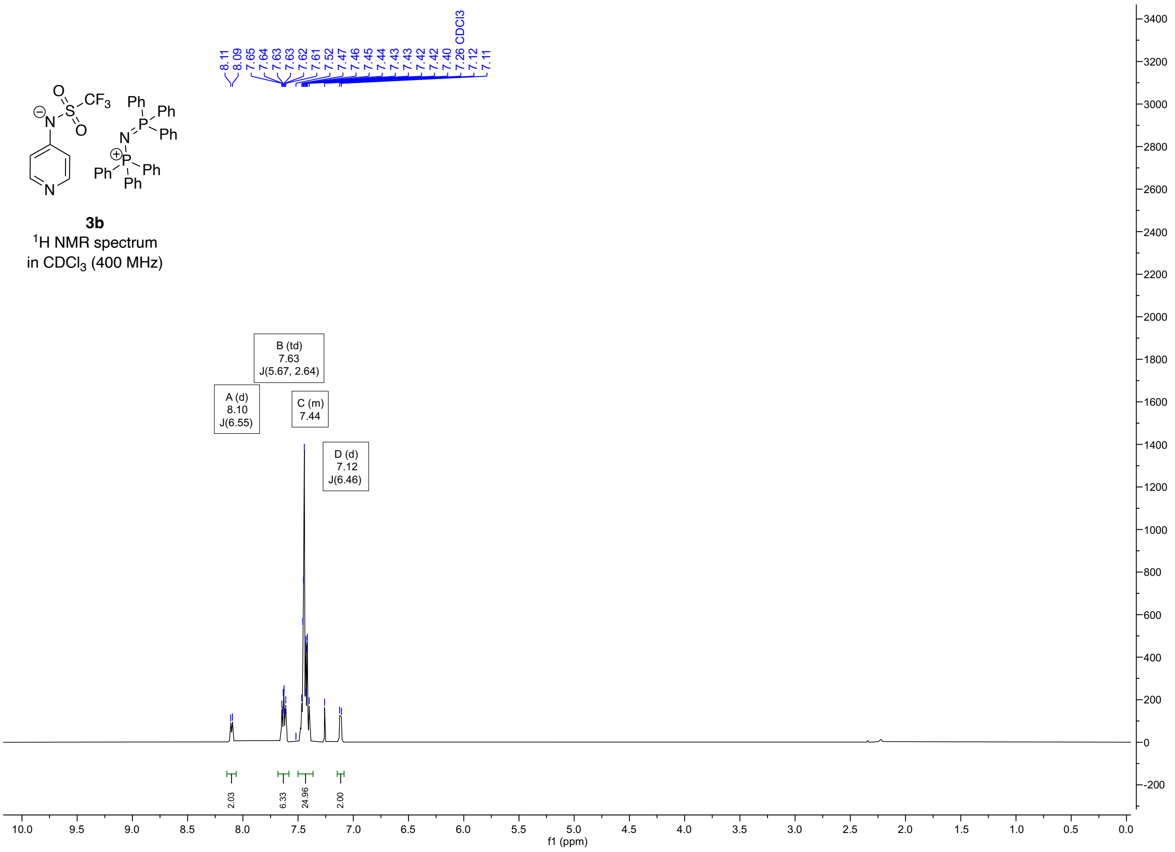

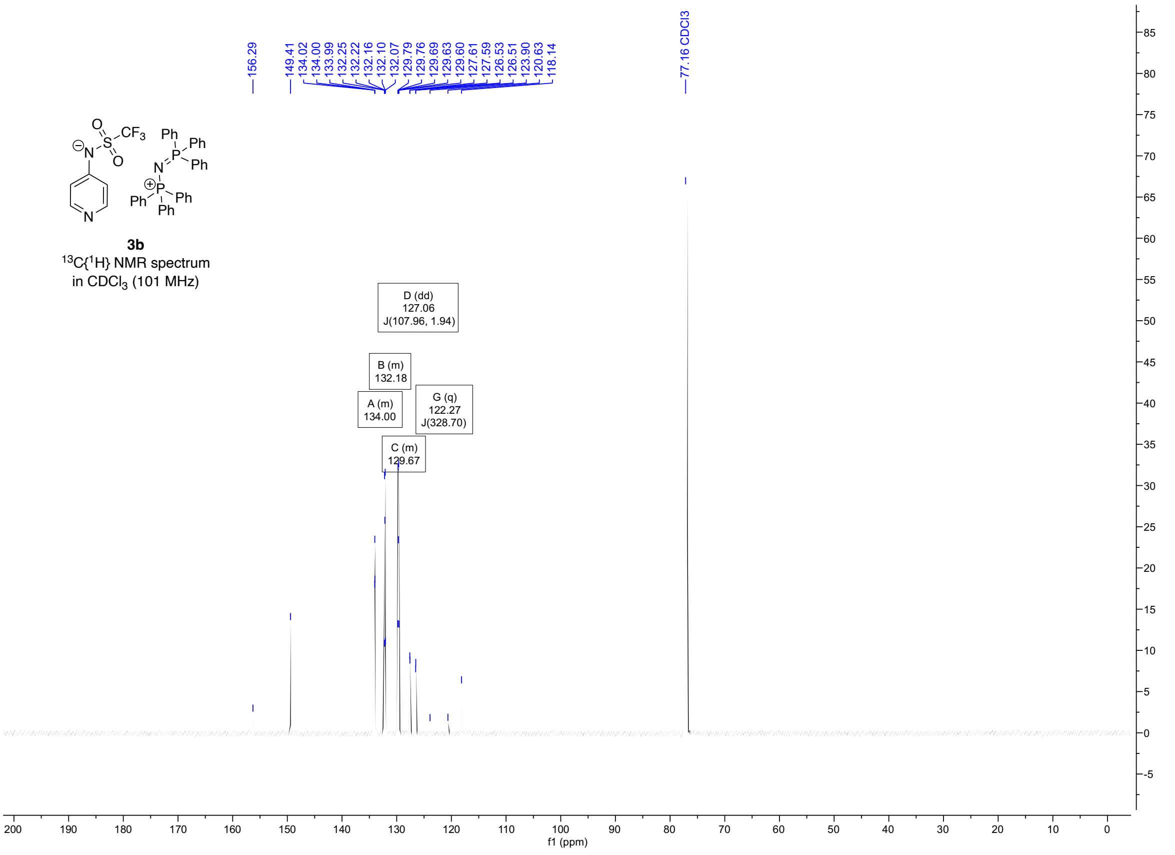

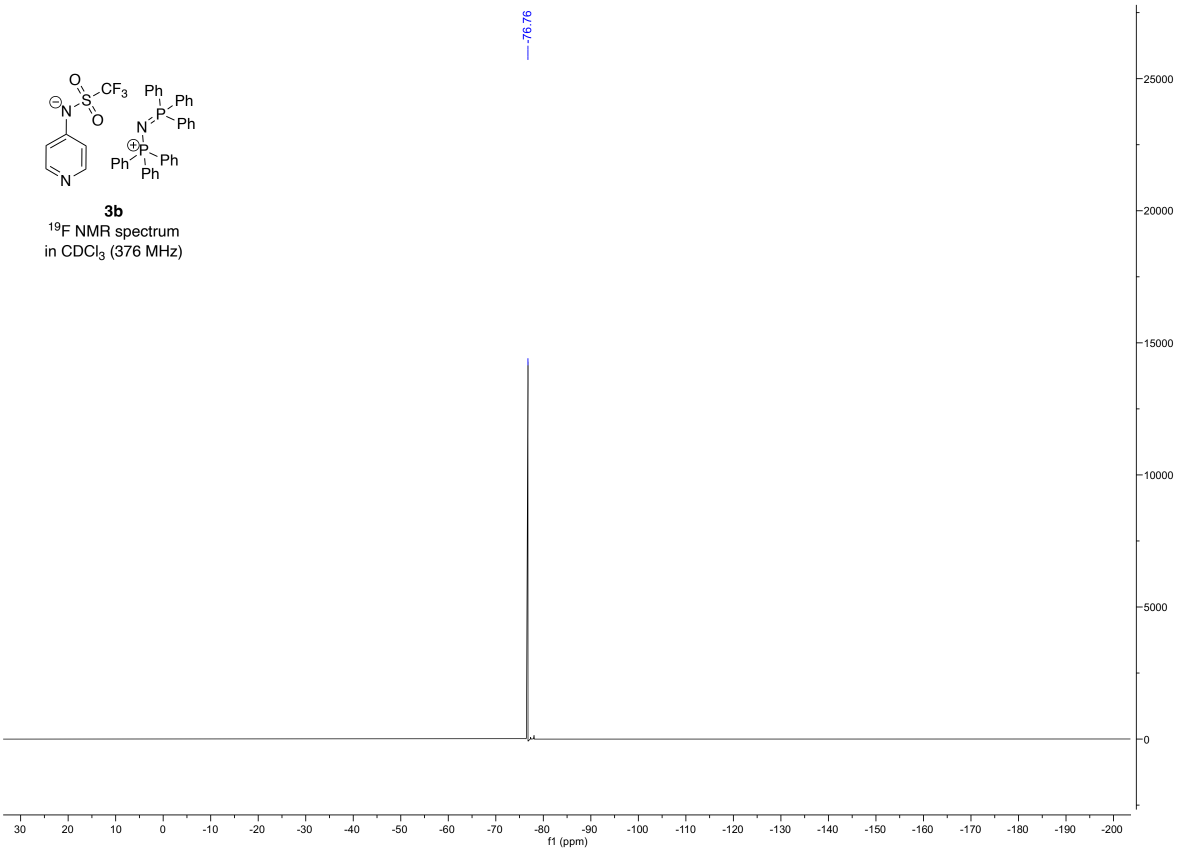
3b
^{19}F NMR spectrum
in $CDCl_3$ (376 MHz)
-76.76
f1 (ppm)

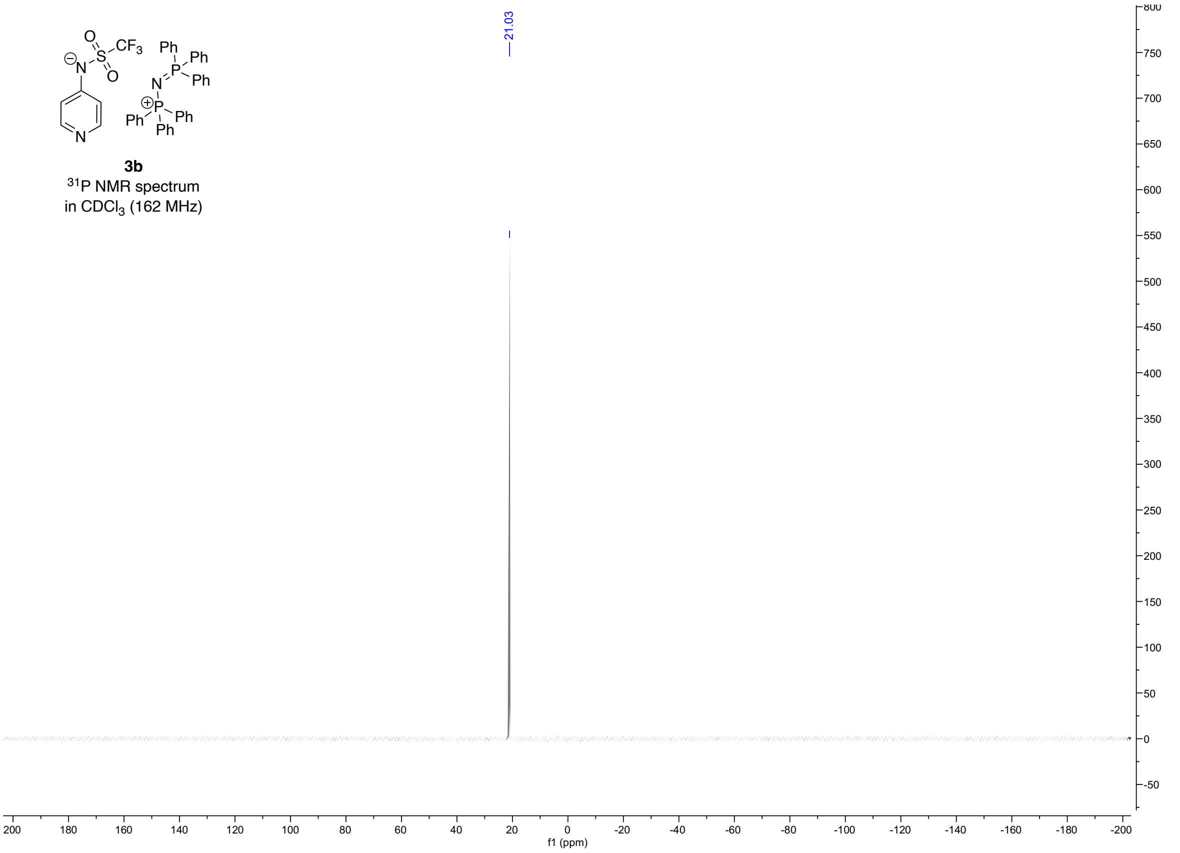
3b
^{31}P NMR spectrum
in $CDCl_3$ (162 MHz)
21.03
f1 (ppm)

Tetraphenylphosphonium pyridin-4-yl(tosyl)amide (**5a**)

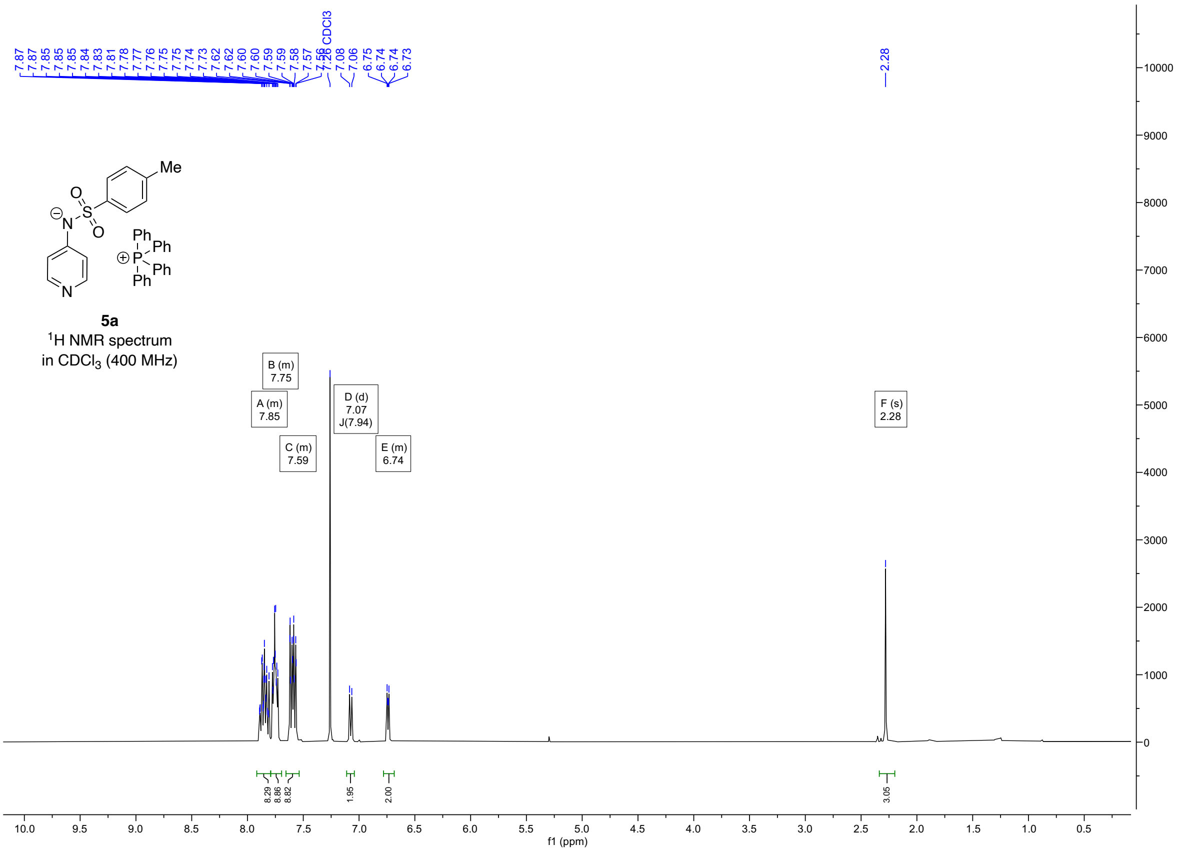

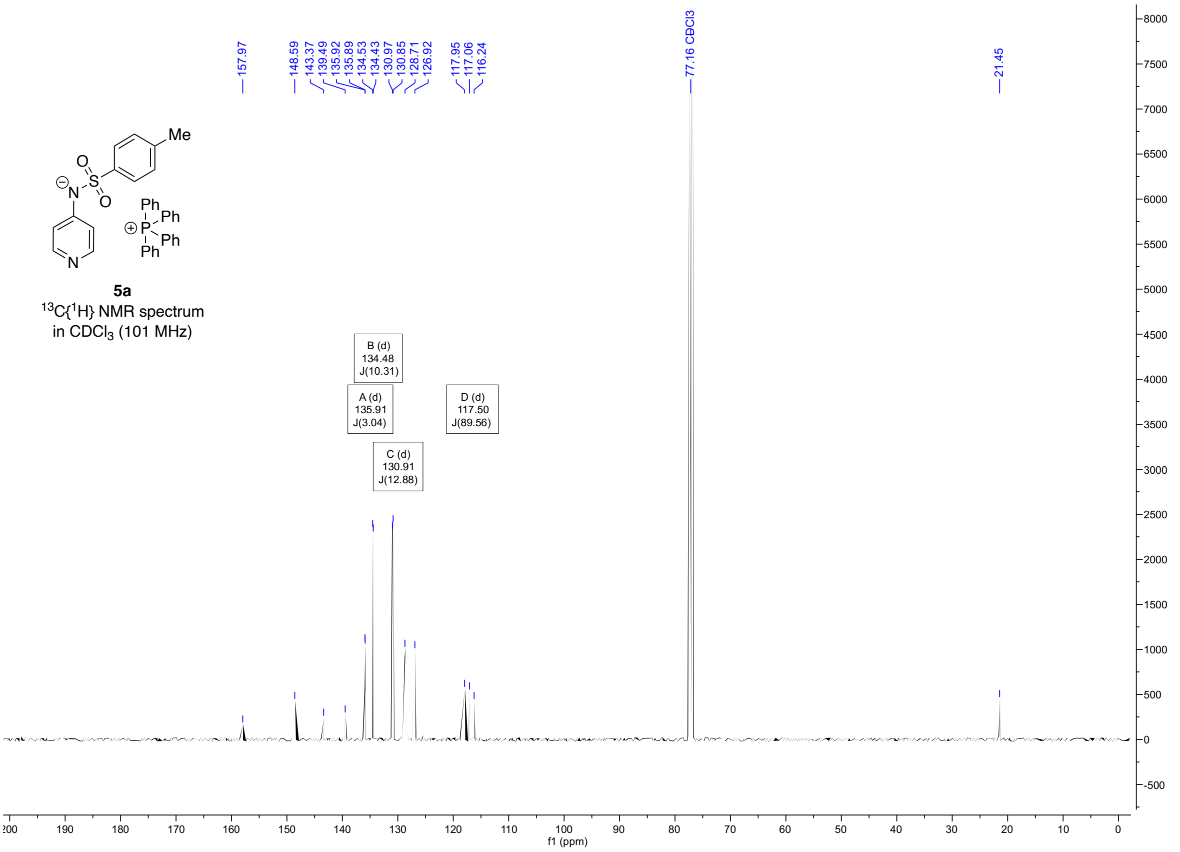

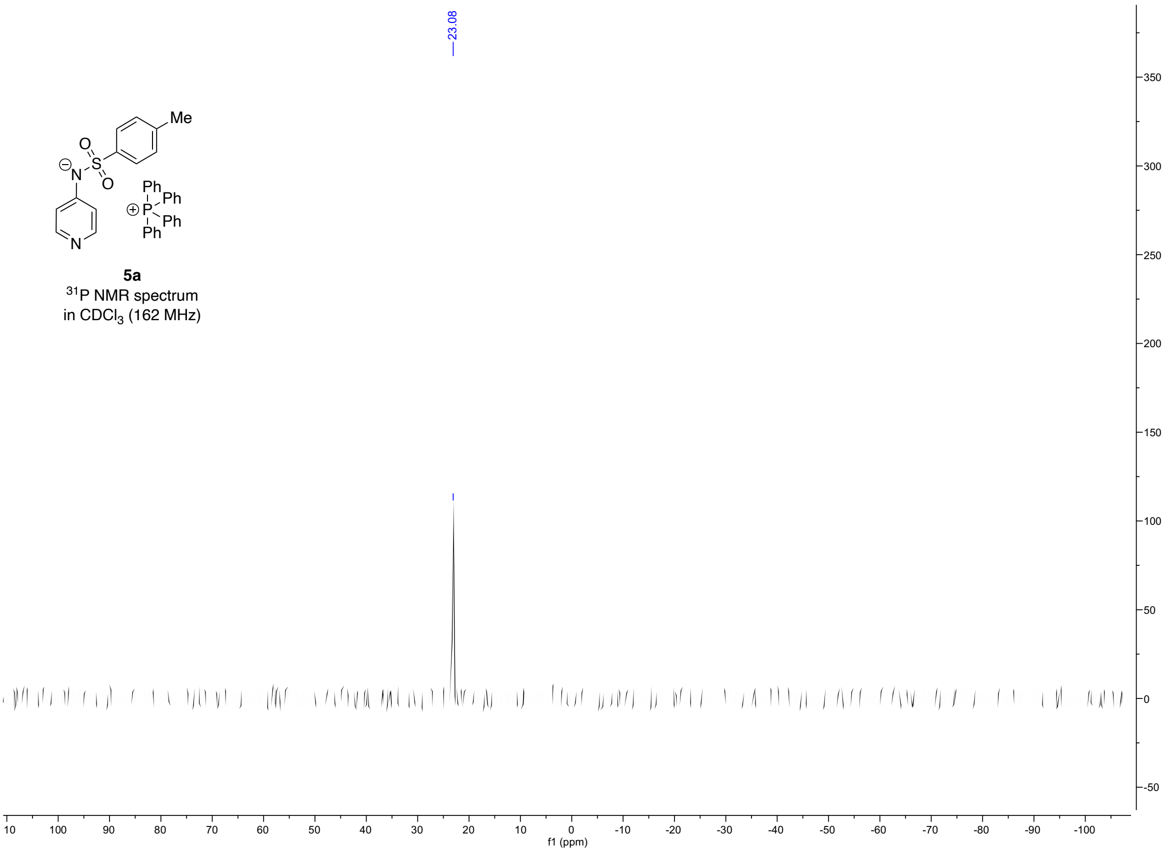

Tetraphenylphosphonium pyridin-4-yl((4-(trifluoromethyl)phenyl)sulfonyl)amide (**6a**)

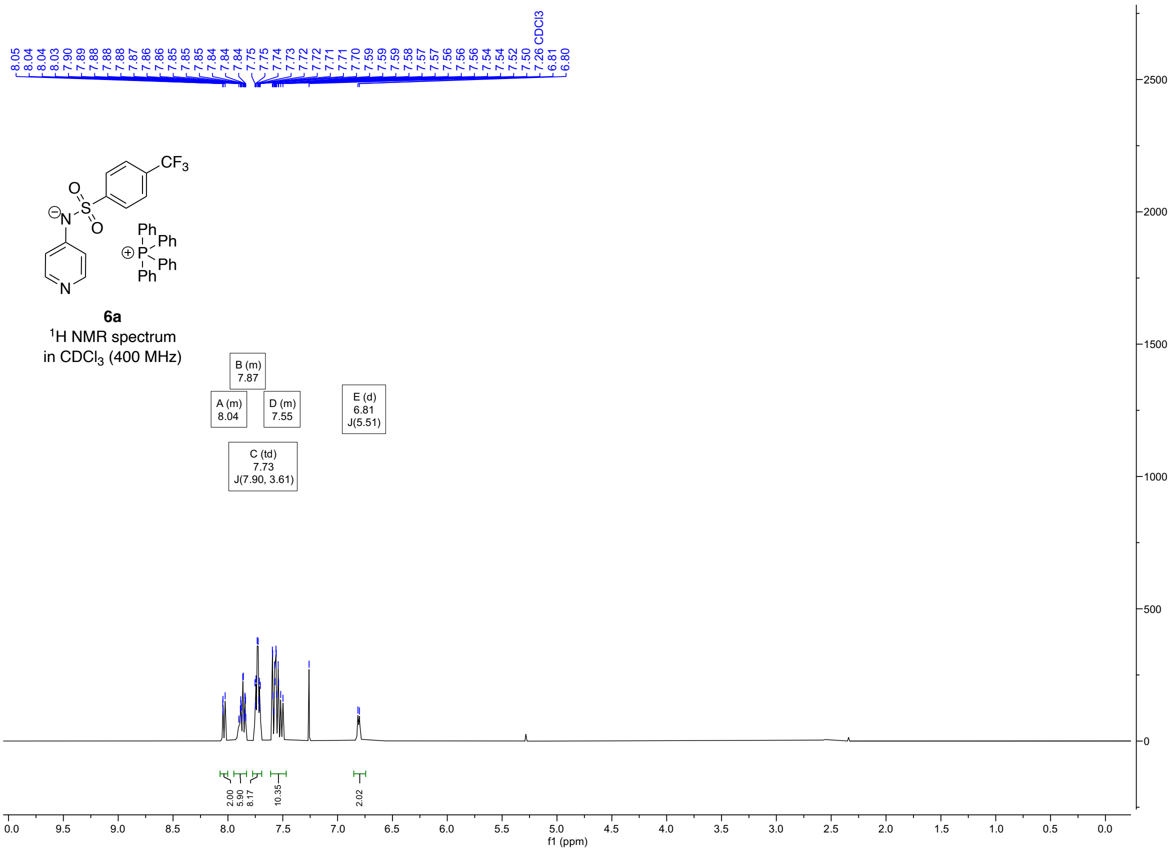

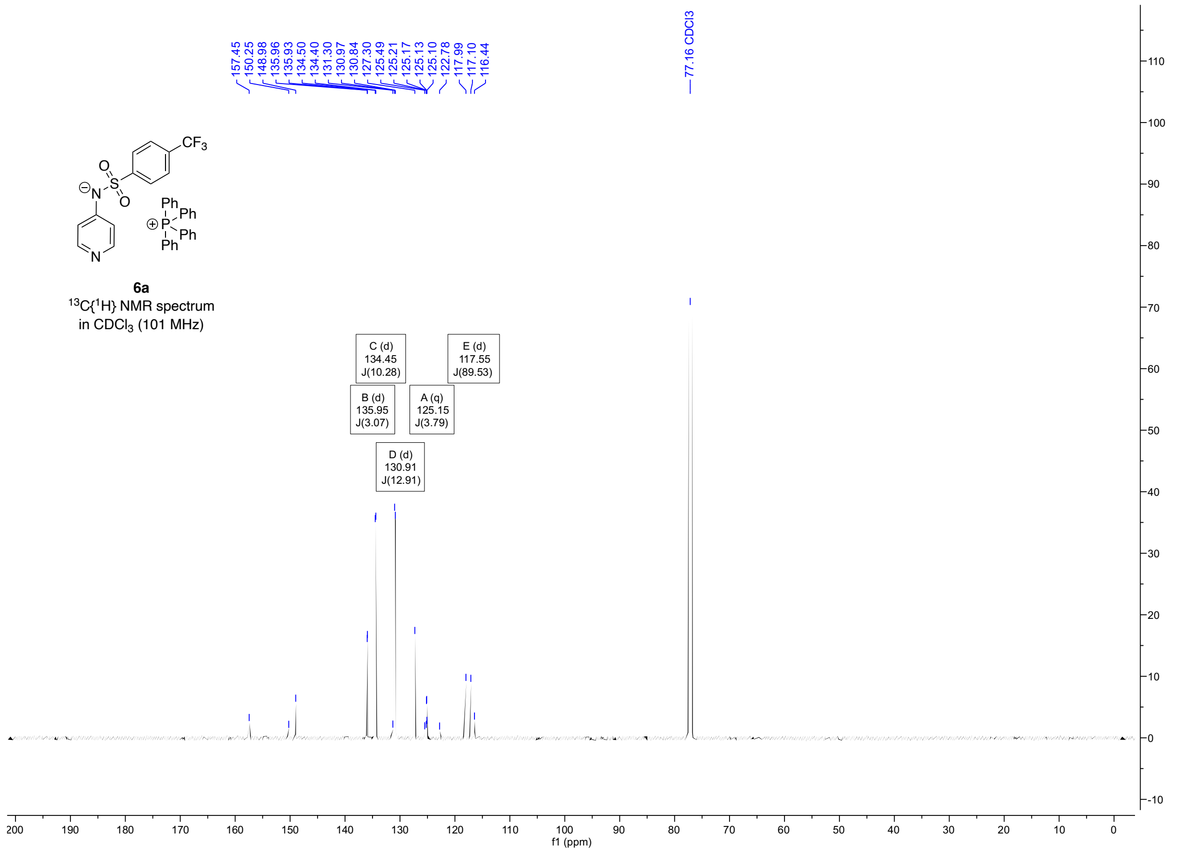
157.45
150.25
148.98
135.96
135.93
134.50
134.40
131.30
130.97
130.84
127.30
125.49
125.21
125.17
125.13
125.10
122.78
117.99
117.10
116.44
77.16 CDCl3
CF3
Ph
6a
$^{13}C\{^{1}H\}$ NMR spectrum
in $CDCl_3$ (101 MHz)
C (d) 134.45 J(10.28)
B (d) 135.95 J(3.07)
E (d) 117.55 J(89.53)
A (q) 125.15 J(3.79)
D (d) 130.91 J(12.91)
f1 (ppm)

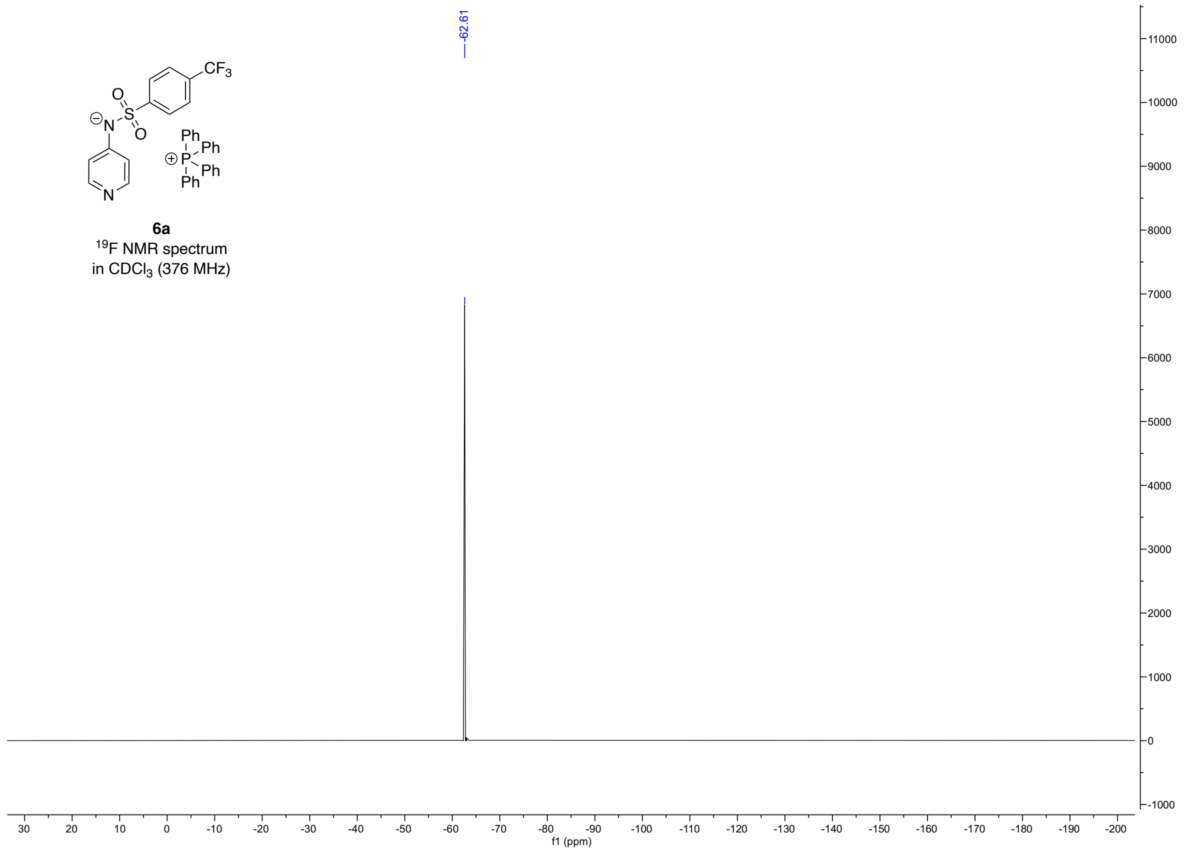
-62.61
CF3
Ph
6a
^{19}F NMR spectrum
in $CDCl_3$ (376 MHz)
f1 (ppm)

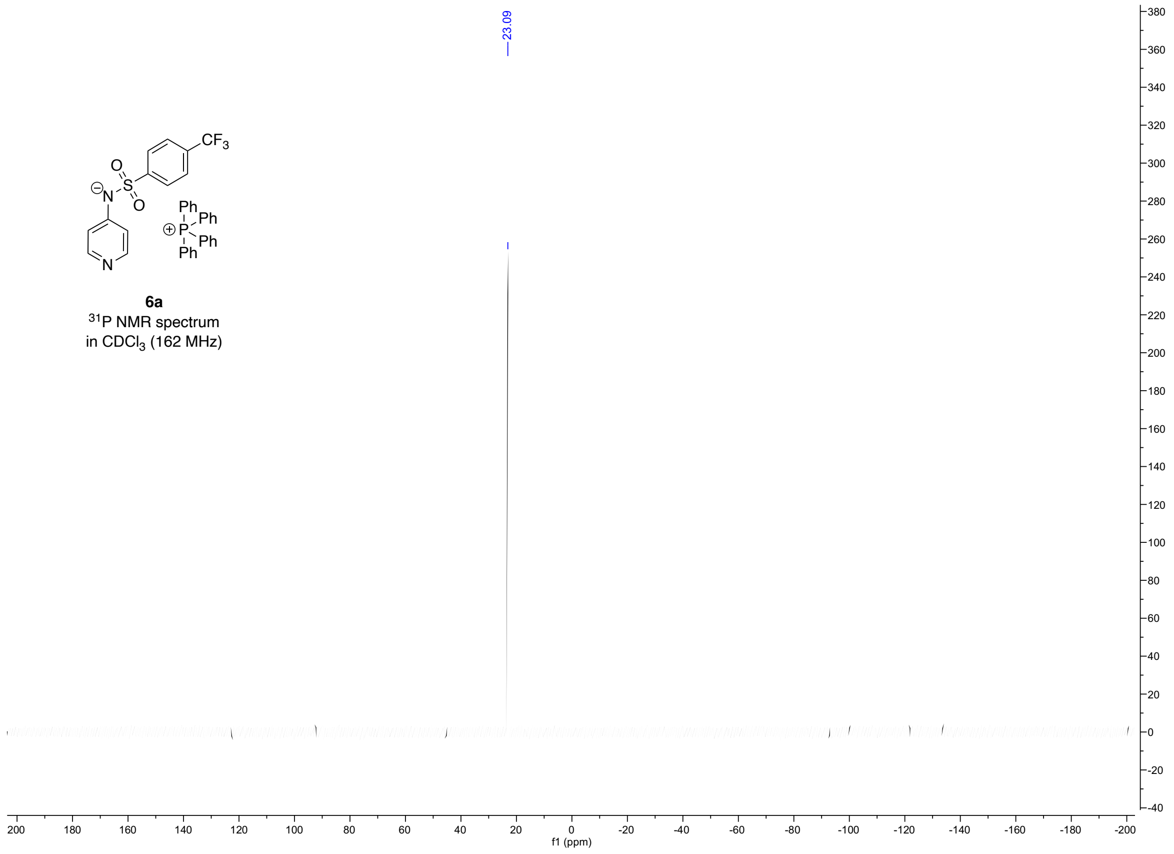

Triphenyl((triphenyl-λ5-phosphaneylidene)amino)phosphonium tetrafluoroborate (**7b**)

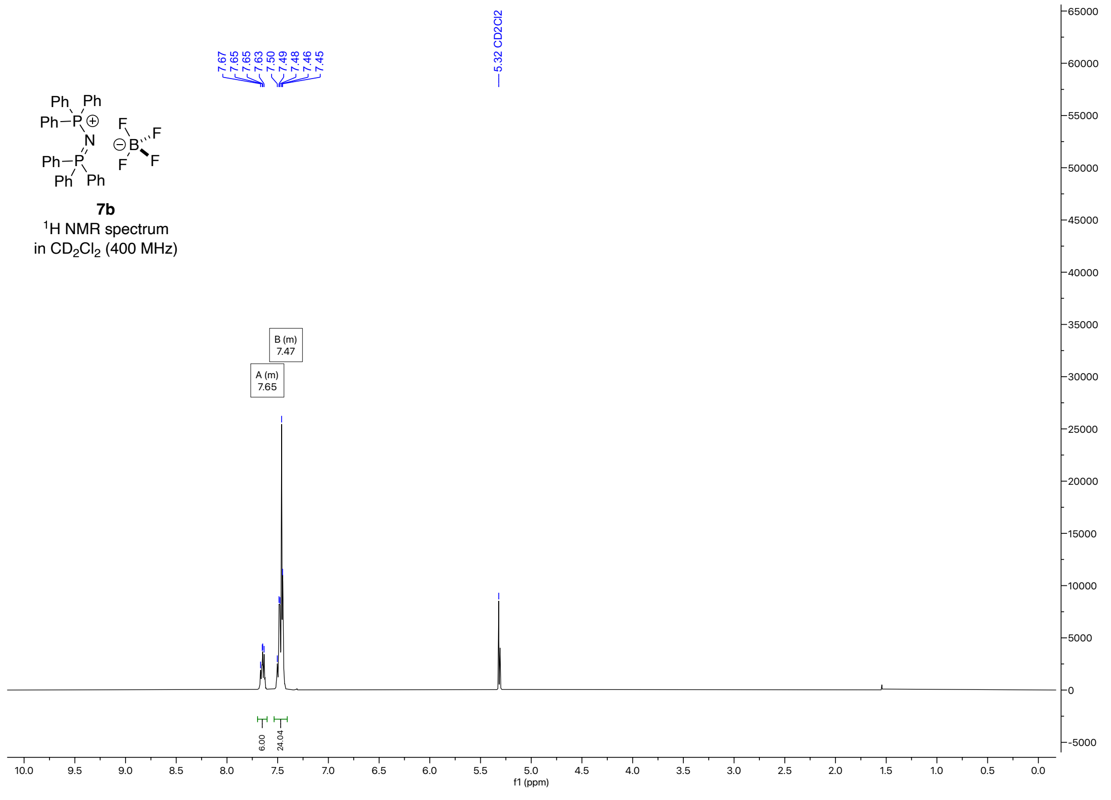

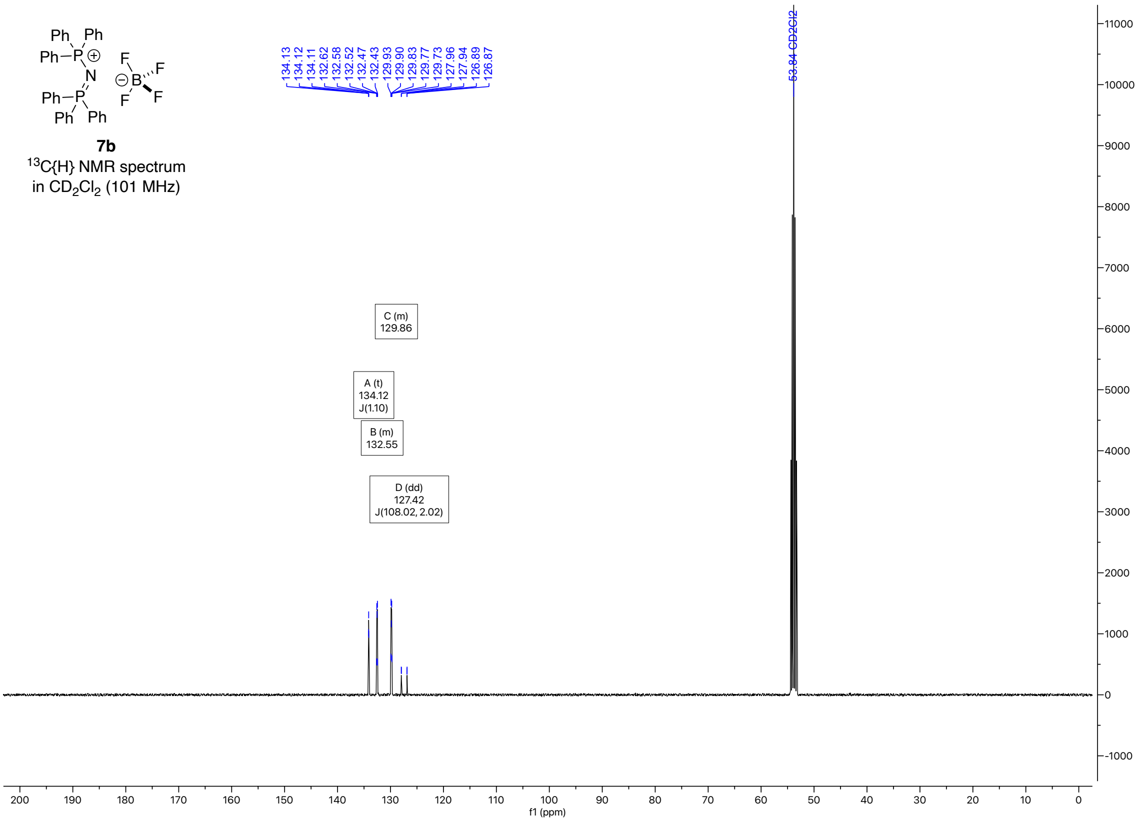

7b
^{13}C{H} NMR spectrum
in CD_2Cl_2 (101 MHz)
134.13
134.12
134.11
132.62
132.58
132.52
132.47
132.43
129.93
129.90
129.83
129.77
129.73
127.96
127.94
126.89
126.87
53.84 CD2Cl2
C (m)
129.86
A (t)
134.12
J(1.10)
B (m)
132.55
D (dd)
127.42
J(108.02, 2.02)
f1 (ppm)

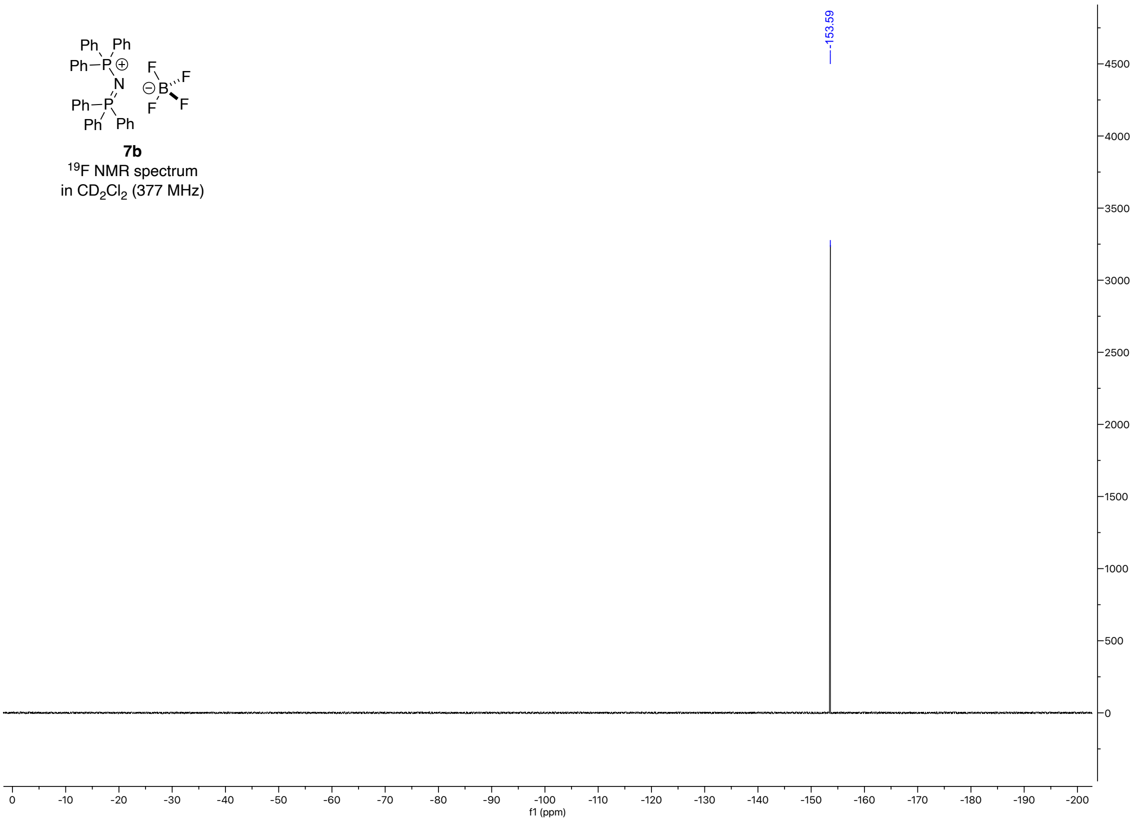

7b
^{19}F NMR spectrum
in CD_2Cl_2 (377 MHz)
-153.59
f1 (ppm)

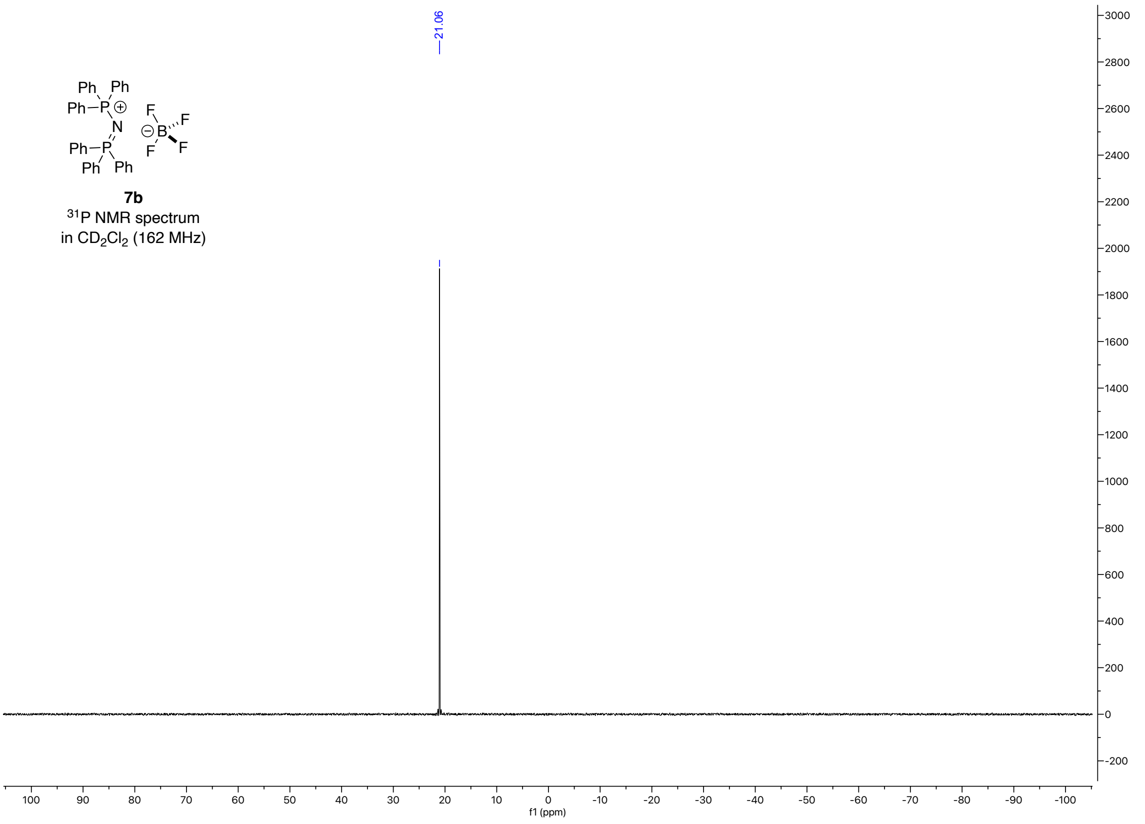

<u>Tetrabutylammonium tetrafluoroborate (**7c**)</u>

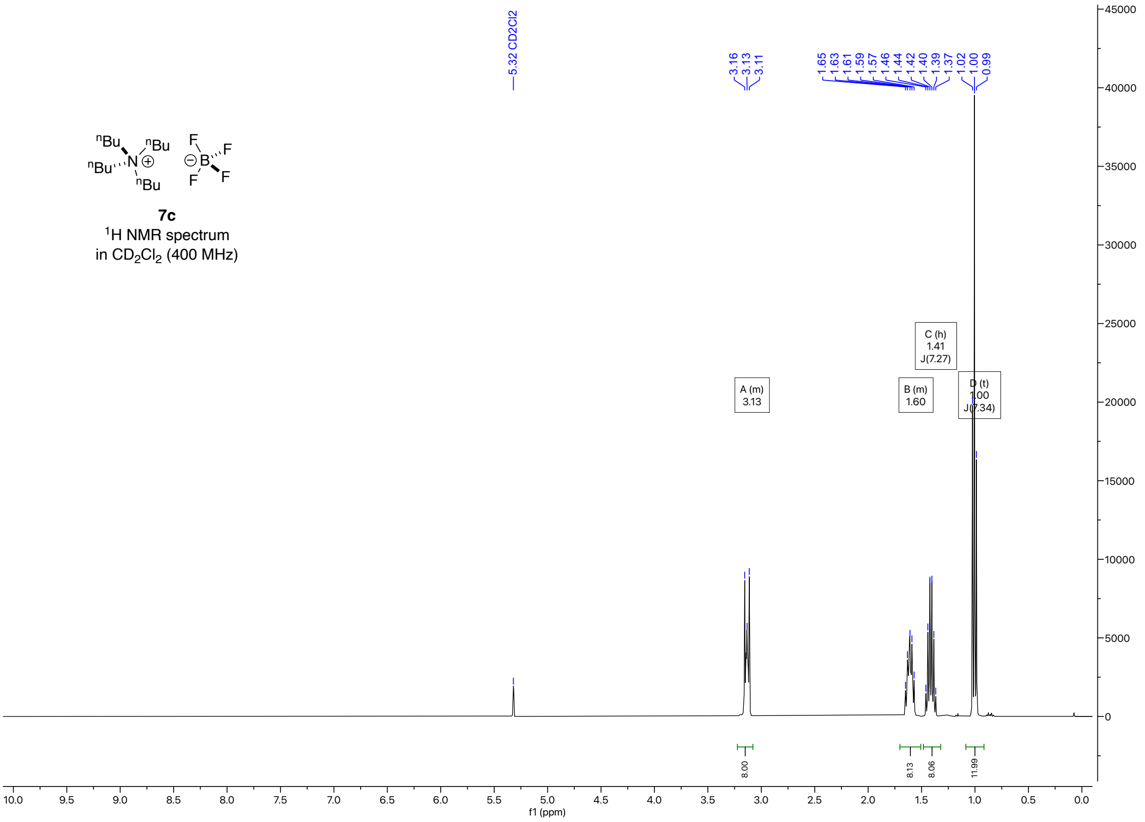

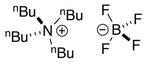

7c
$^{13}C\{^{1}H\}$ NMR spectrum
in CD_2Cl_2 (101 MHz)

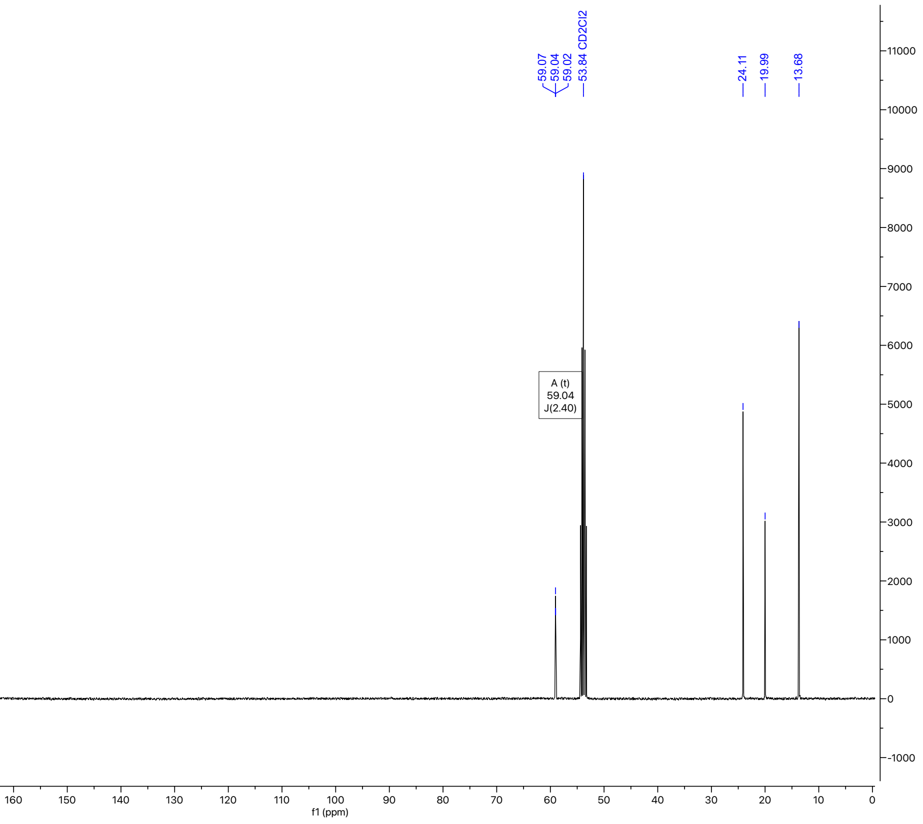

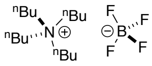

7c
^{19}F NMR spectrum
in CD_2Cl_2 (376 MHz)

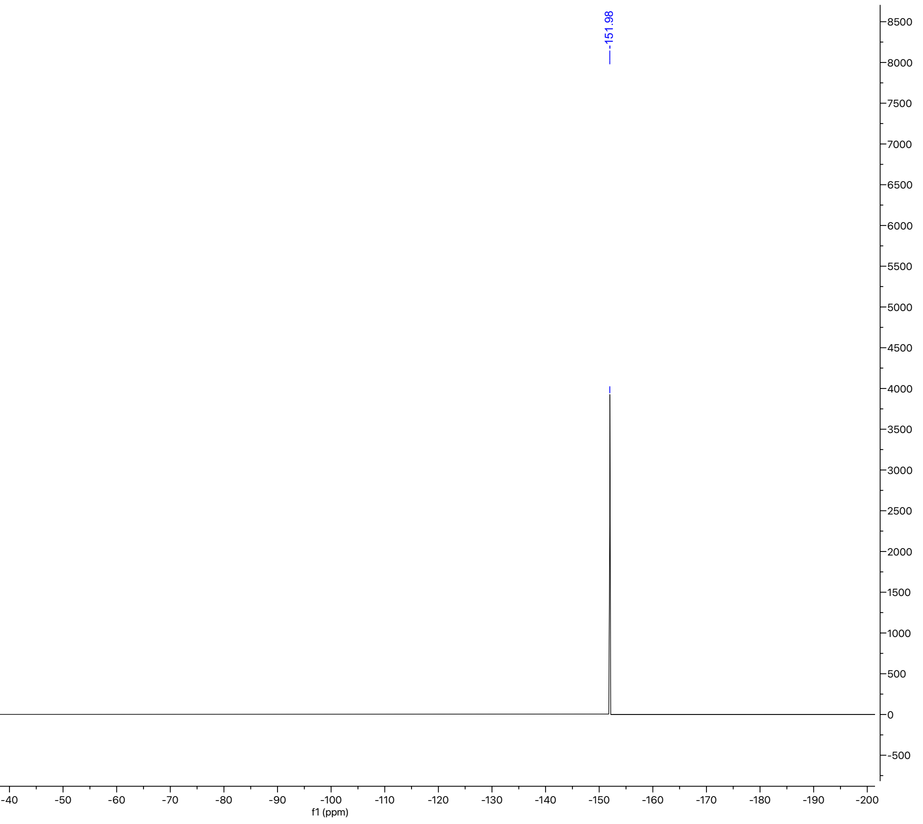

Tetrabutylphosphonium tetrafluoroborate (**7d**)

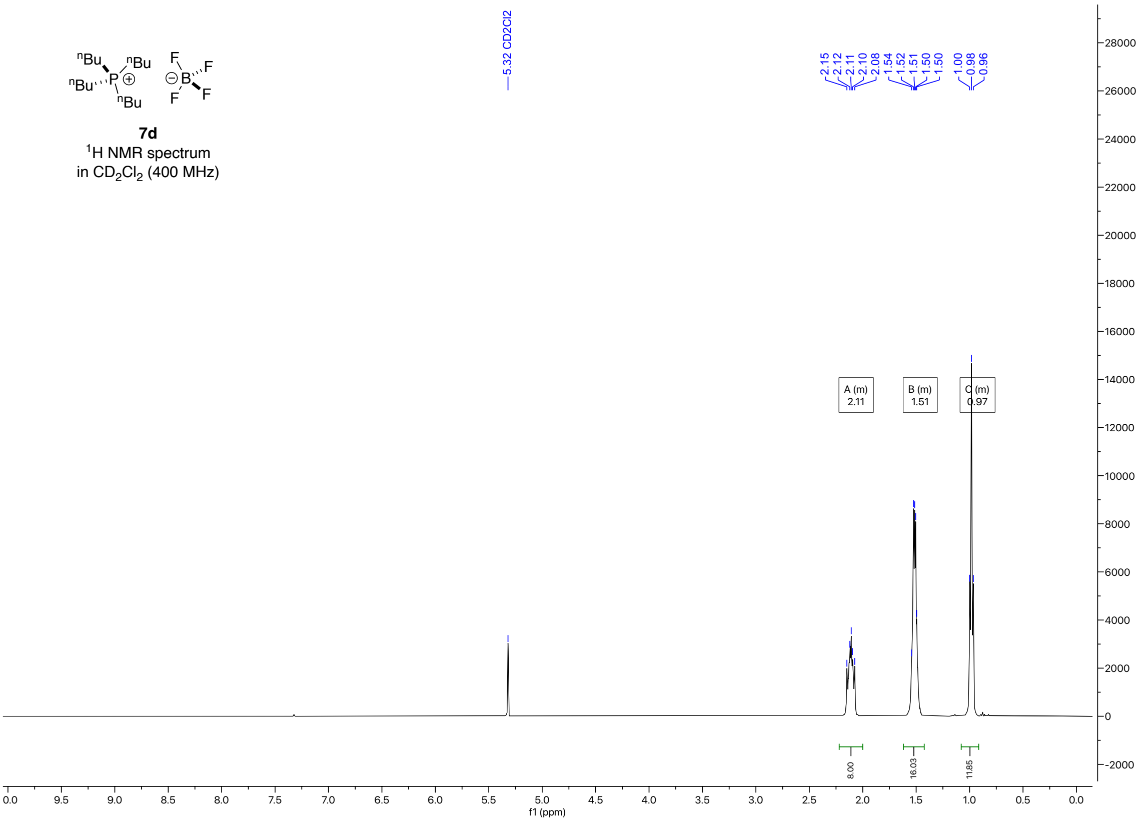

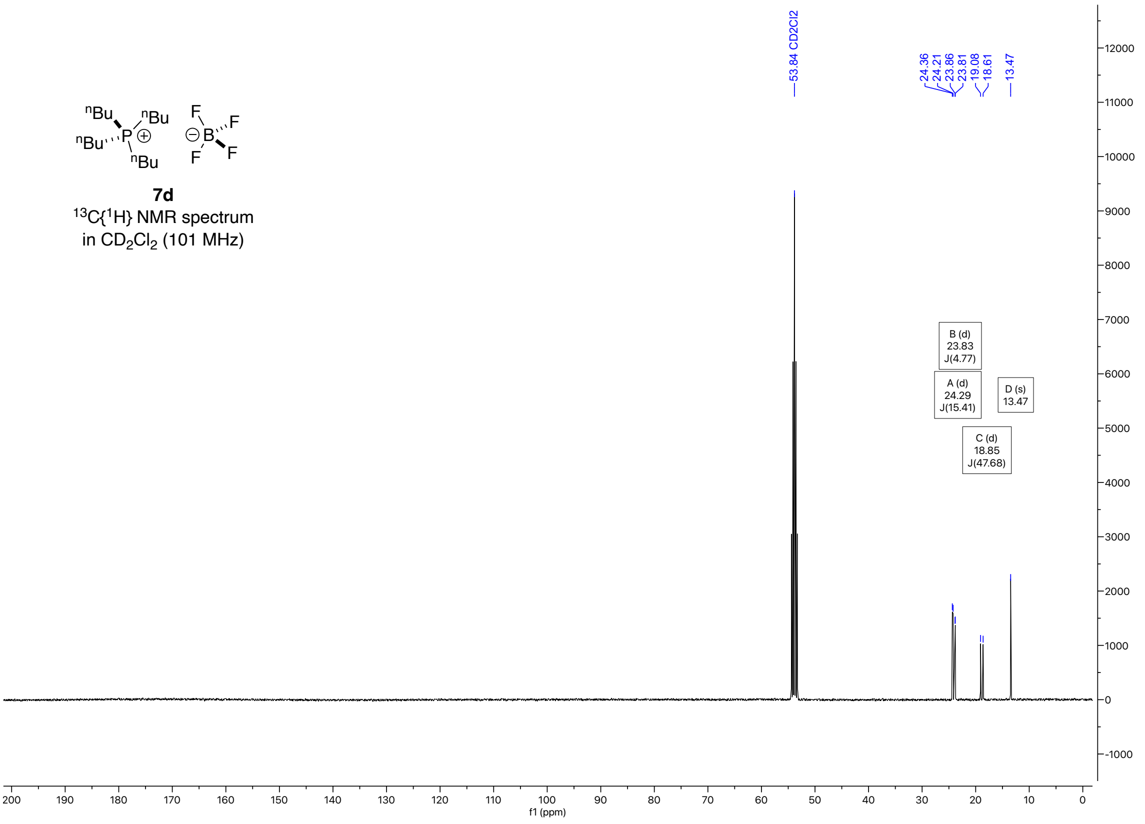

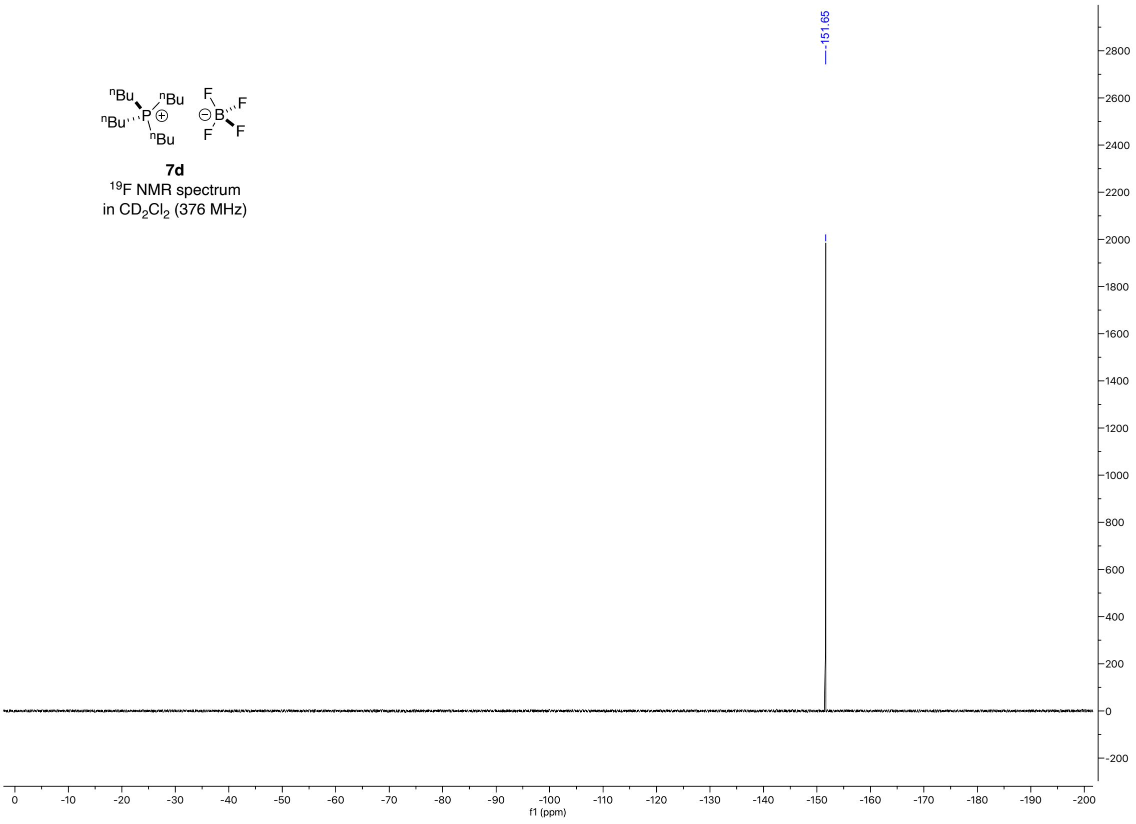
-151.65
7d
^{19}F NMR spectrum
in CD_2Cl_2 (376 MHz)
f1 (ppm)

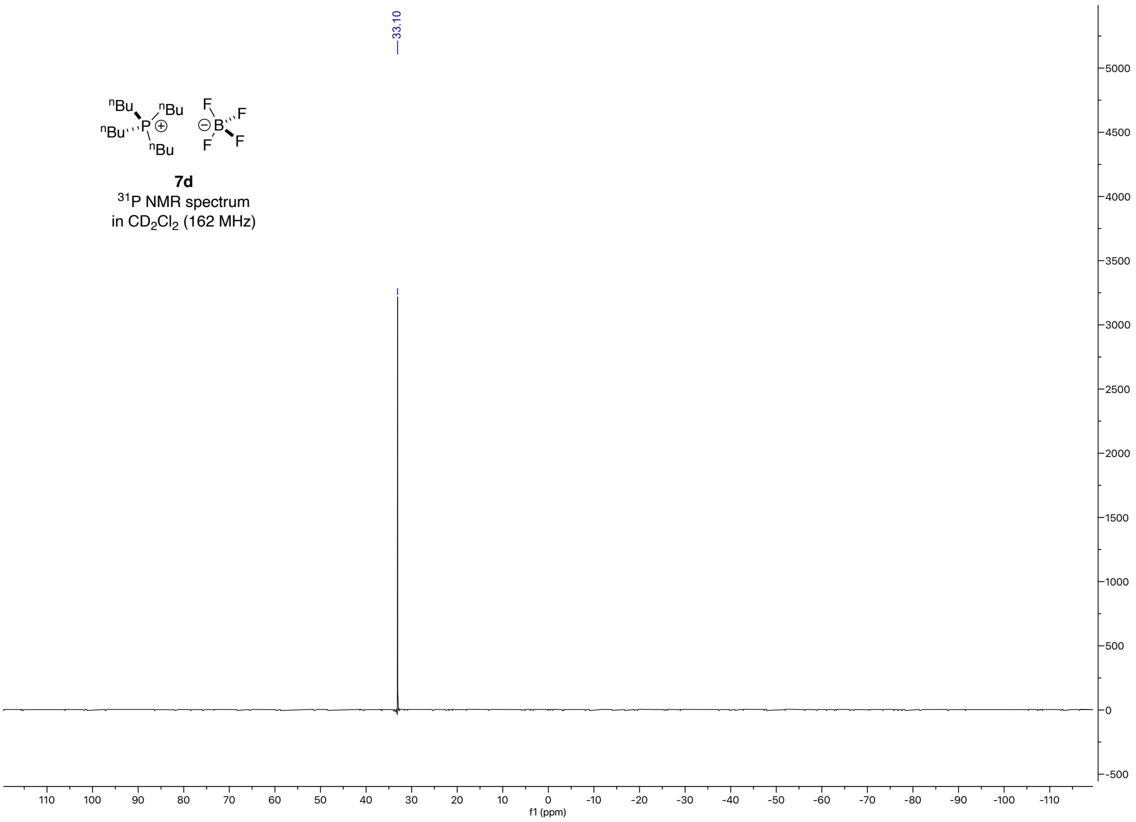
33.10
7d
^{31}P NMR spectrum
in CD_2Cl_2 (162 MHz)
f1 (ppm)

10. DOSY Fits

DOSY curve fits for one signal of each ion are displayed in the following. In addition, the DOSY curve fits of TMS, which was used as reference is also given for each sample. Fitting of the data points was conducted according to the Stejskal-Tanner equation.[12,25] The corresponding R^2 of all measurements are given in the tables of chapter 4.

Ion Pair 3b, 0.05mM, CH_2Cl_2

Anion

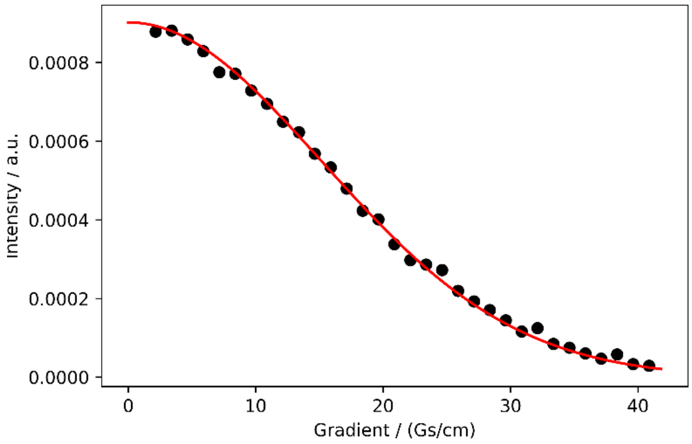

Cation

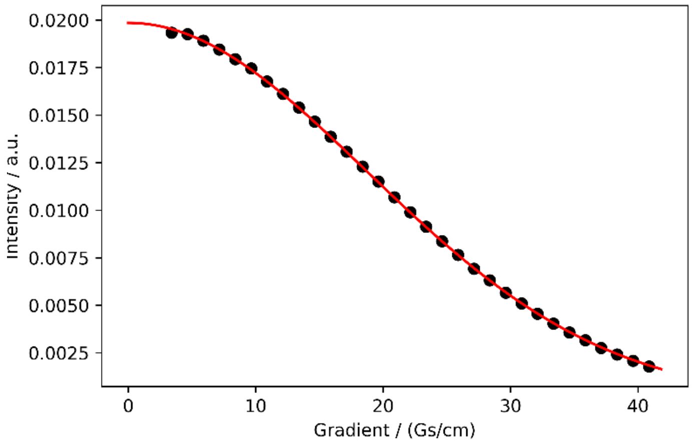

TMS

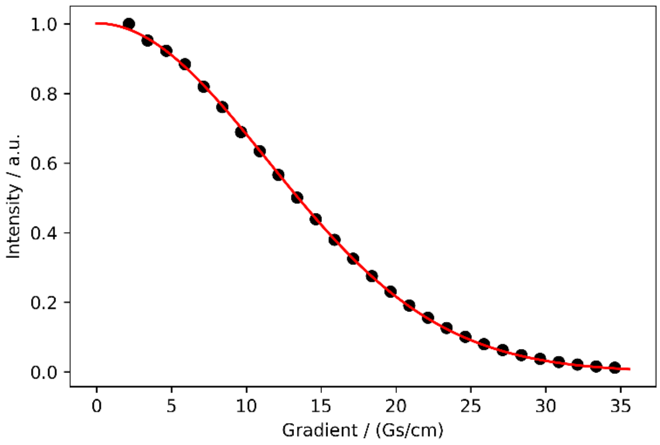

Ion Pair 3b, 0.5 mM, CD_2Cl_2

Anion

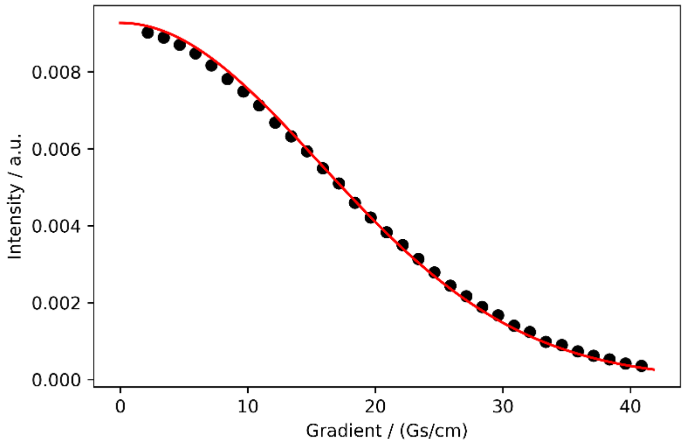

Cation

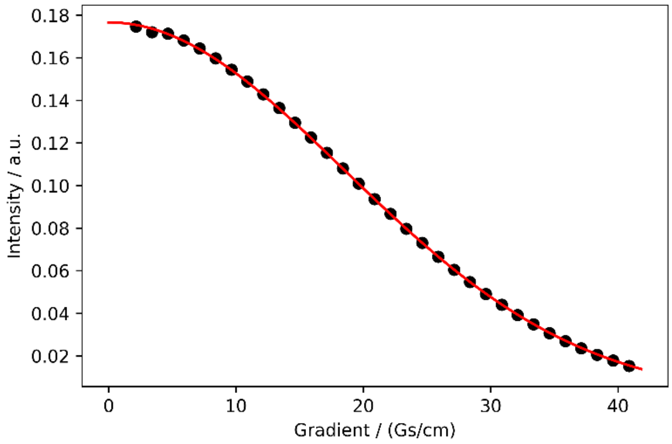

TMS

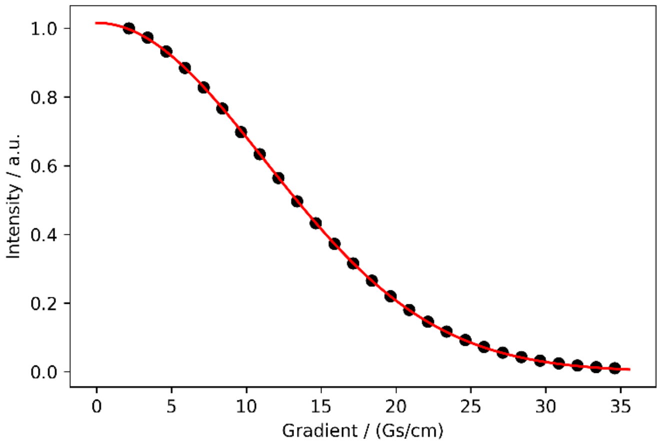

Ion Pair 3b, 1.0 mM, CD_2Cl_2

Anion

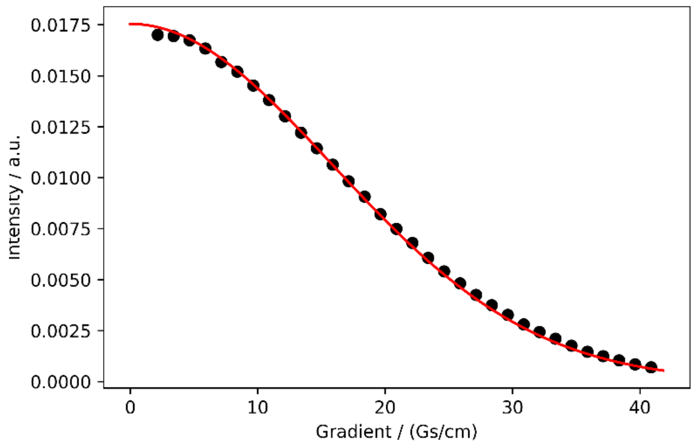

Cation

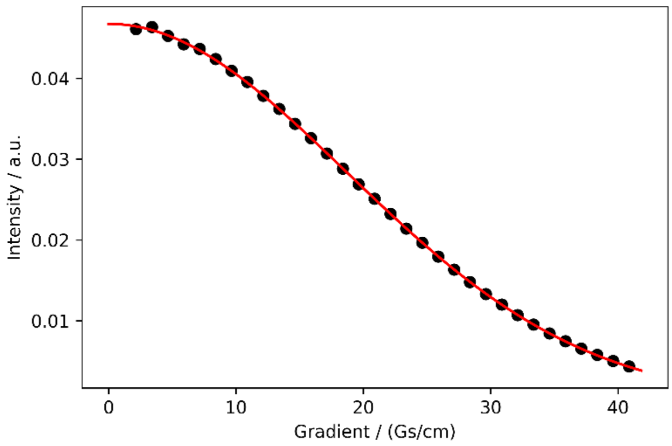

TMS

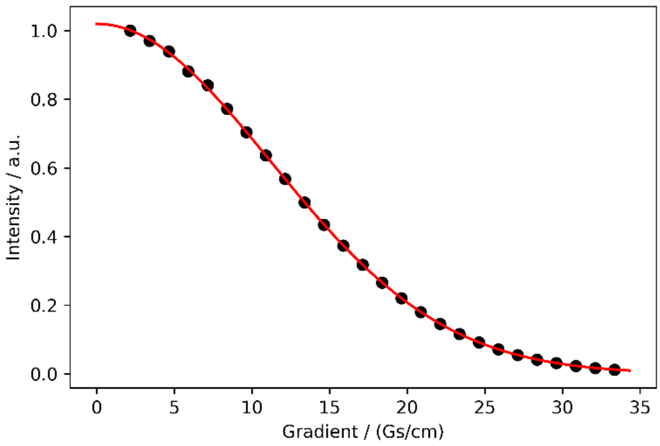

Ion Pair 3b, 5.0 mM, CD_2Cl_2

Anion

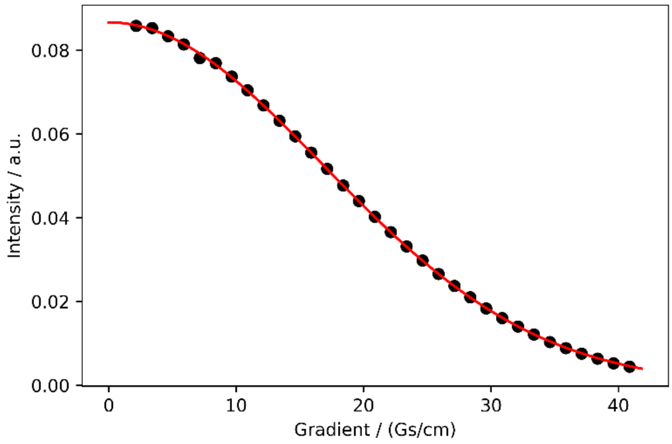

Cation

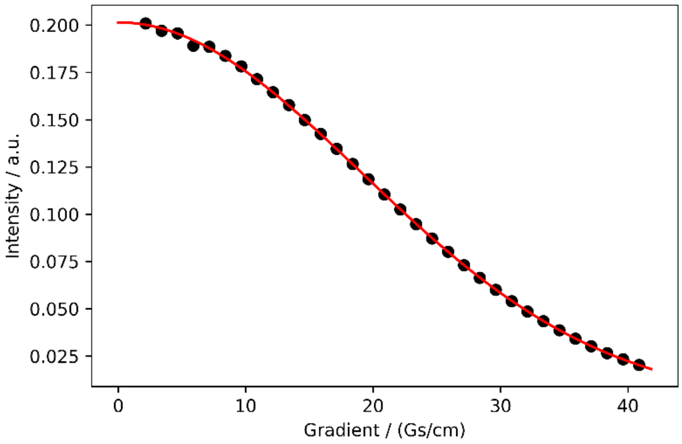

TMS

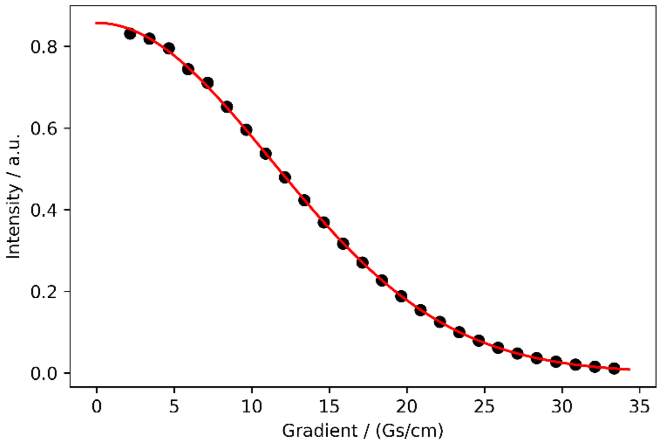

Ion Pair 3c, 0.05 mM, CD_2Cl_2

Anion

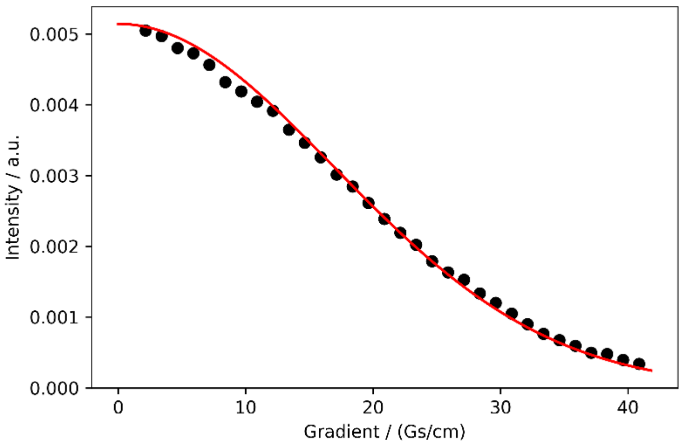

Cation

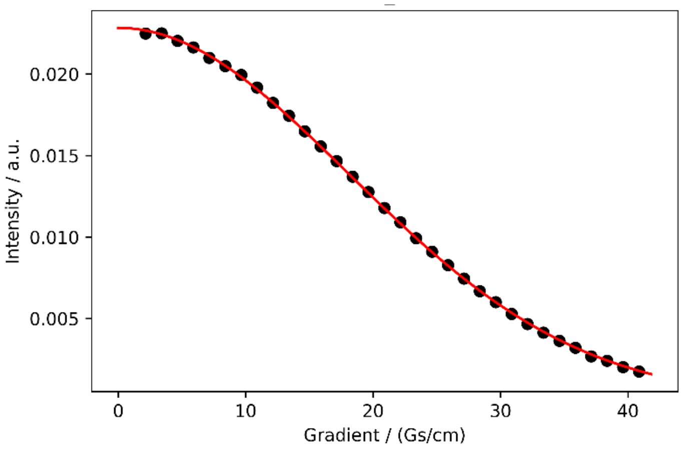

TMS

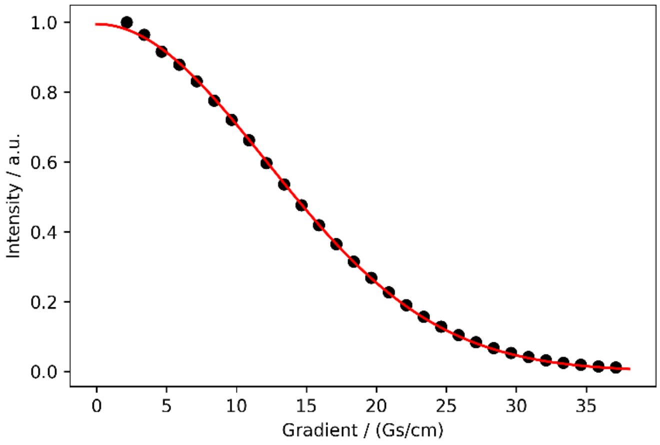

Ion Pair 3c, 1.0 mM, CD_2Cl_2

Anion

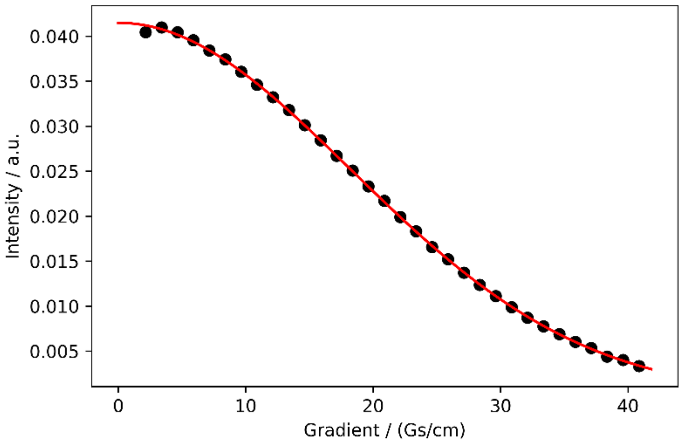

Cation

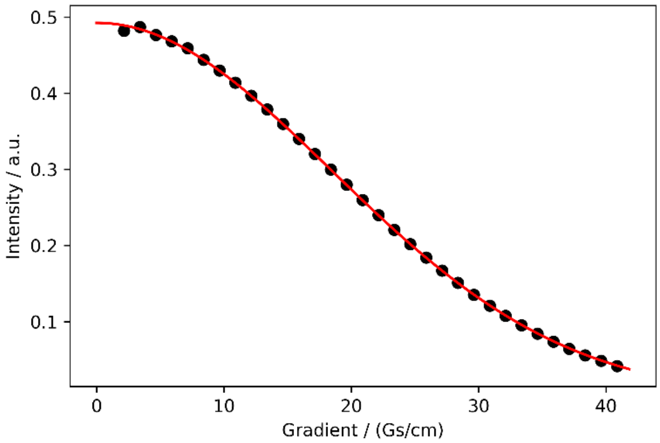

TMS

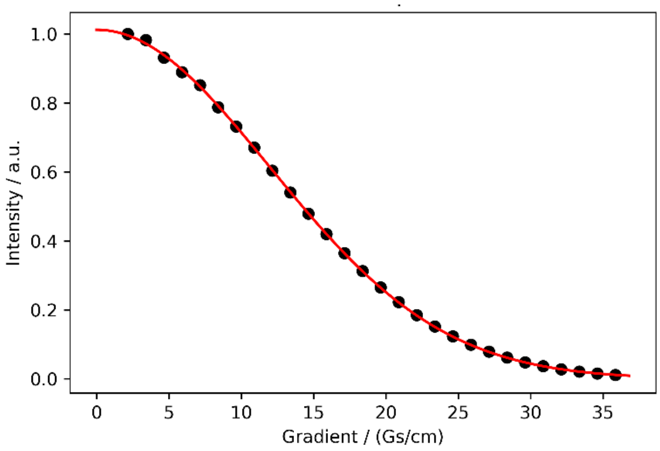

Ion Pair 3c, 5.0 mM, CD_2Cl_2

Anion

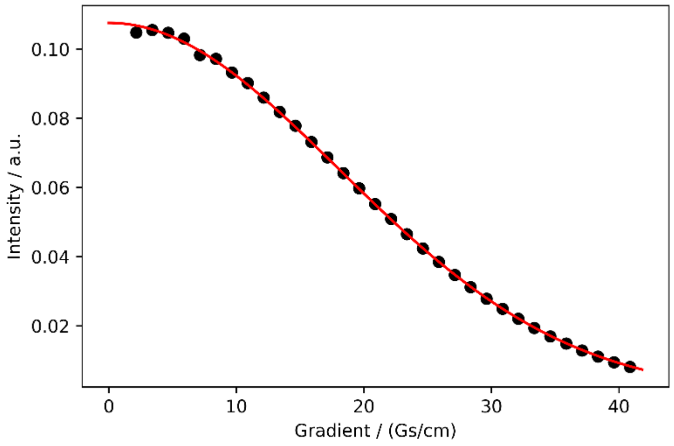

Cation

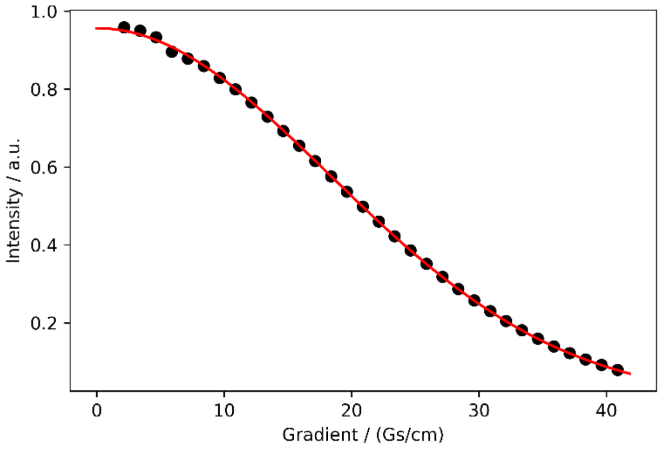

TMS

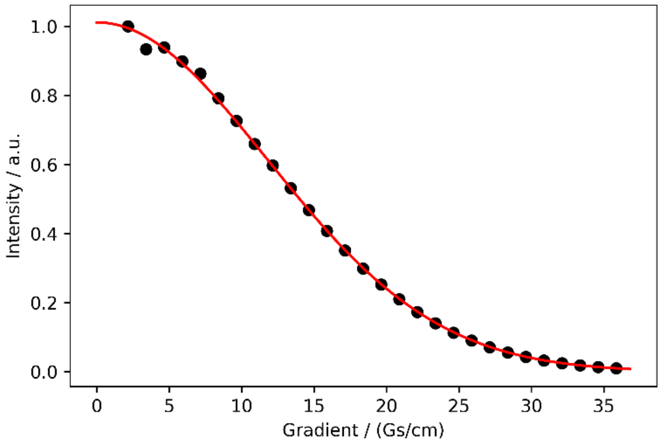

Ion Pair 3d, 0.05 mM, CD_2Cl_2

Anion

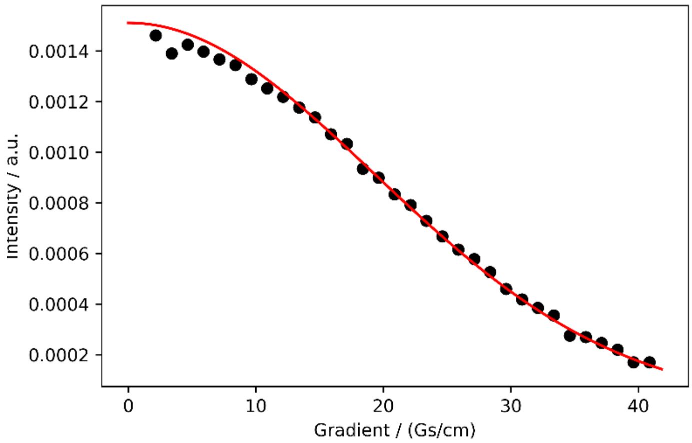

Cation

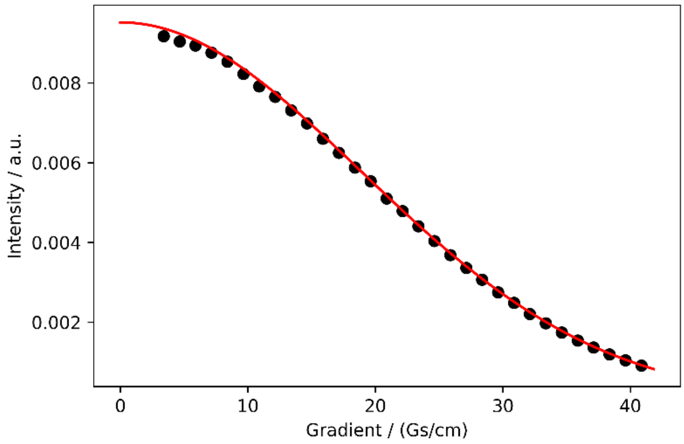

TMS

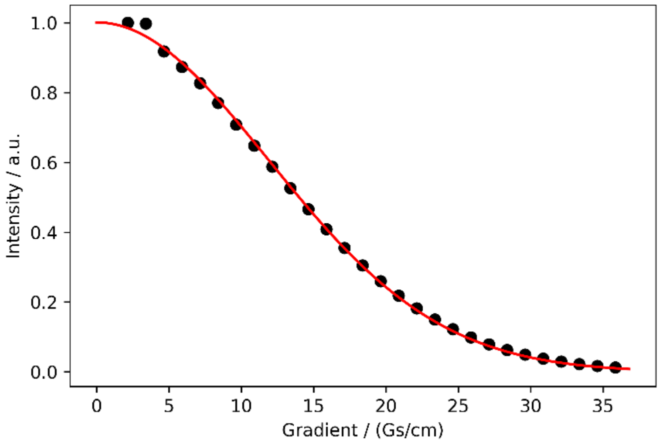

Ion Pair 3d, 0.1 mM, CD_2Cl_2

Anion

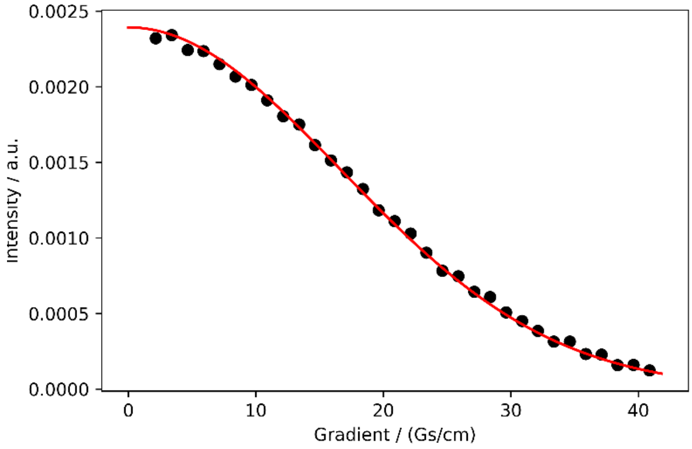

Cation

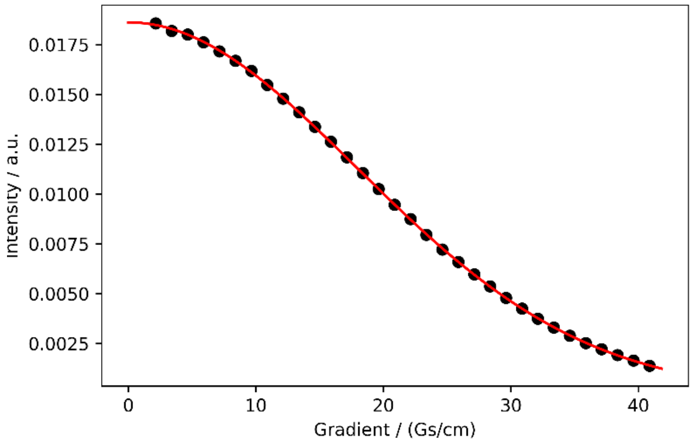

TMS

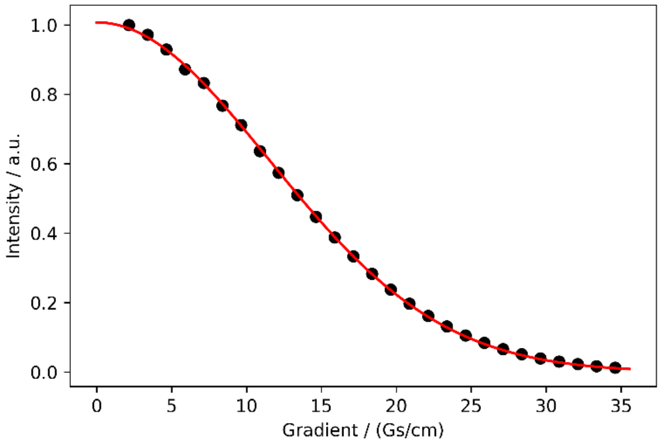

Ion Pair 3d, 0.5 mM, CD_2Cl_2

Anion

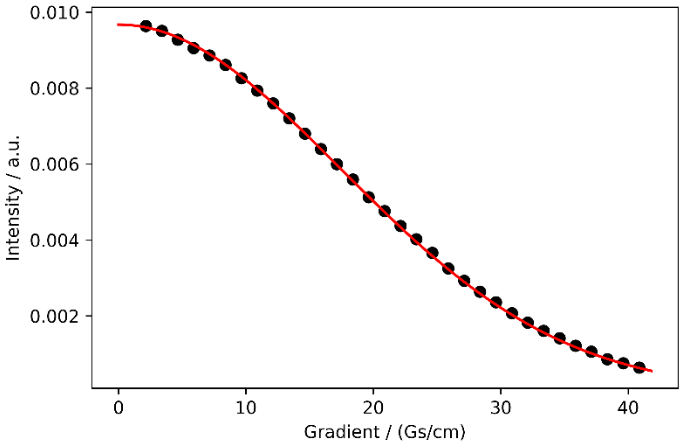

Cation

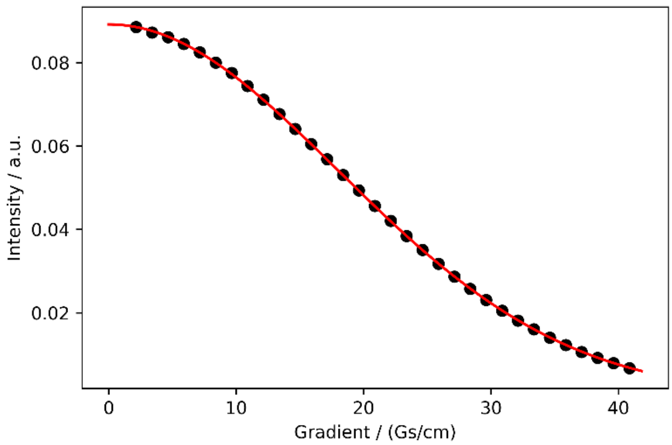

TMS

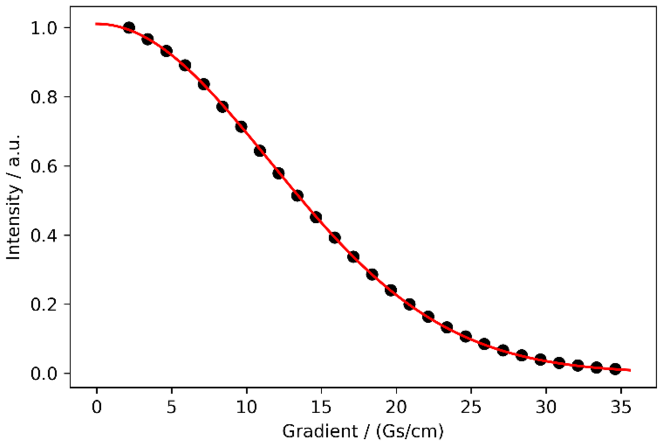

Ion Pair 3d, 1.0 mM, CD_2Cl_2

Anion

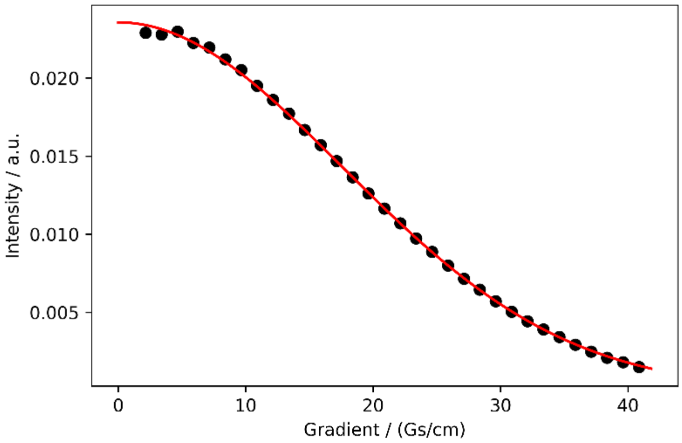

Cation

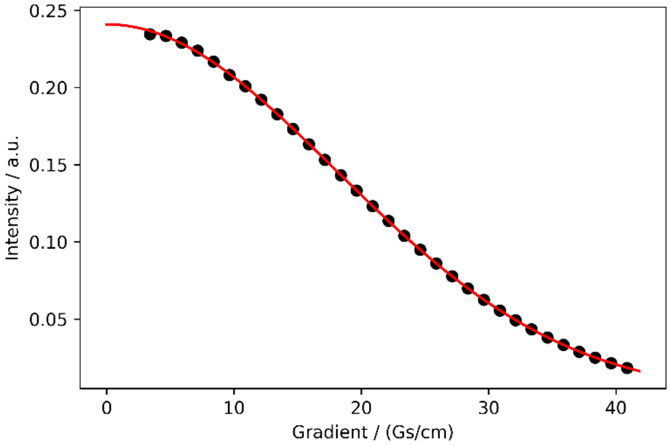

TMS

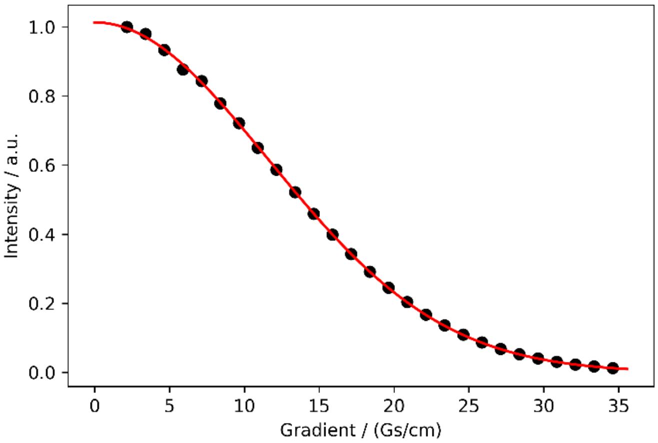

Ion Pair 3d, 5.0 mM, CD_2Cl_2

Anion

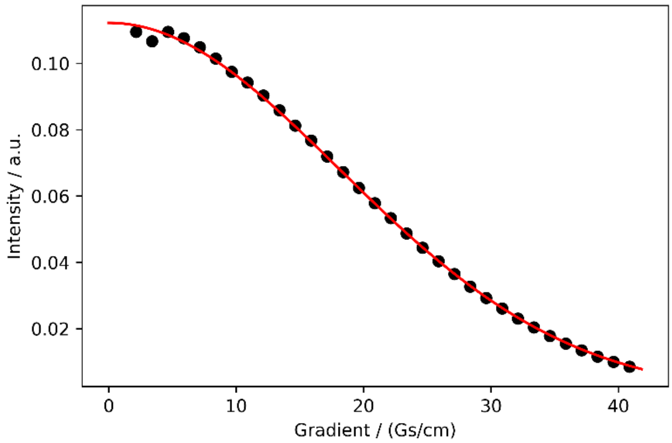

Cation

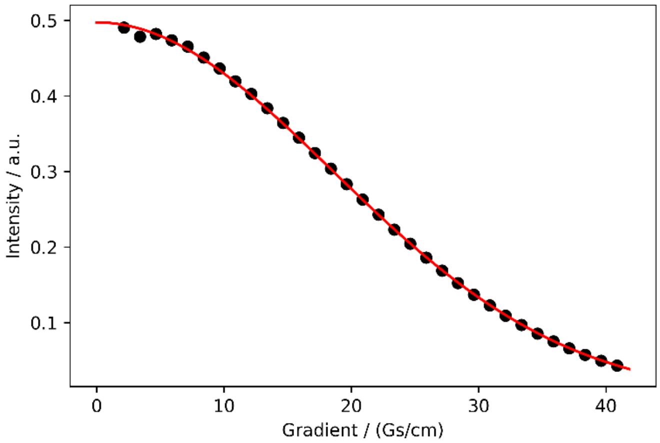

TMS

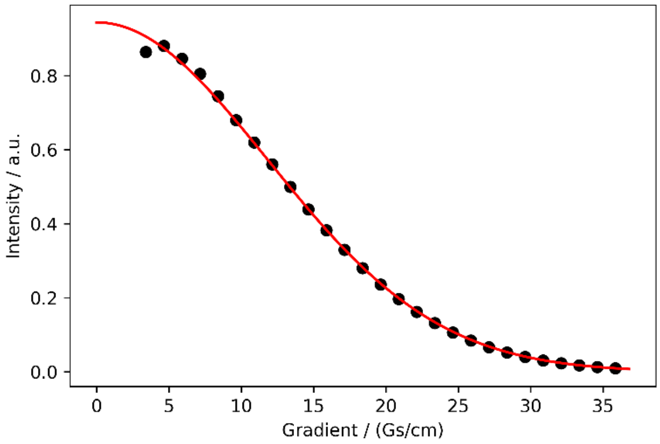

Ion Pair 5a, 0.05 mM, CD_2Cl_2

Anion

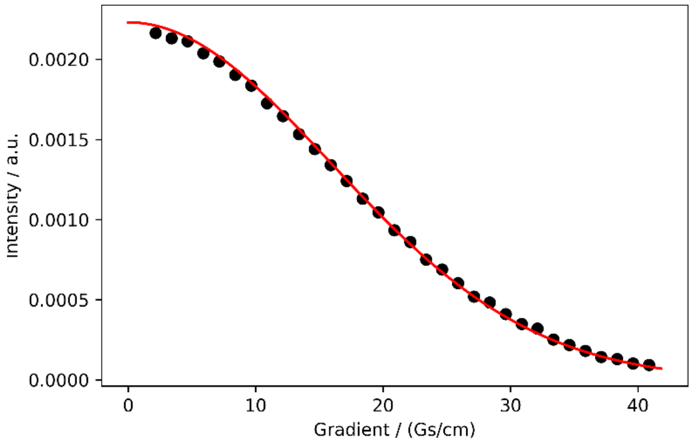

Cation

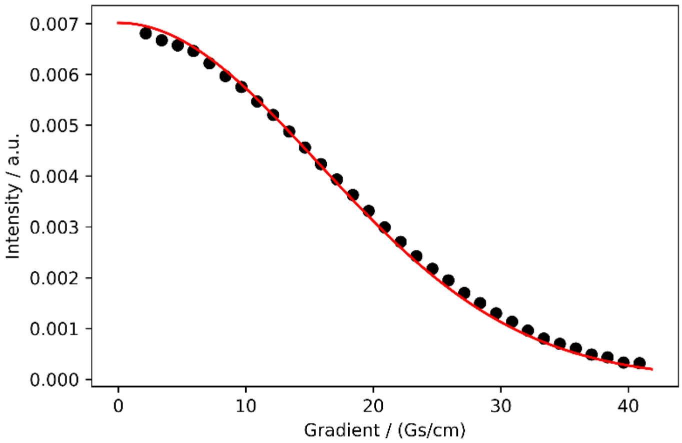

TMS

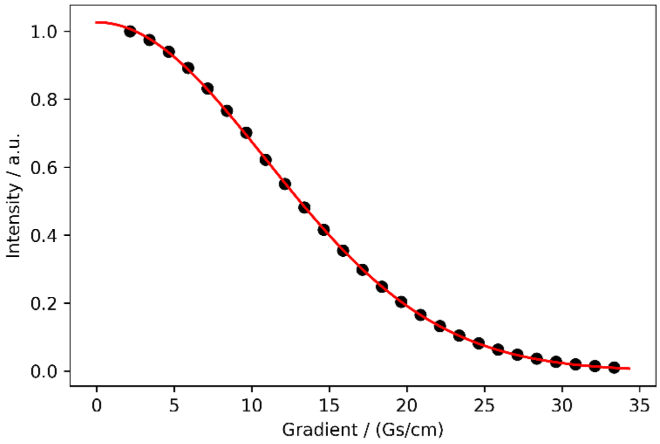

Ion Pair 5a, 0.5 mM, CD_2Cl_2

Anion

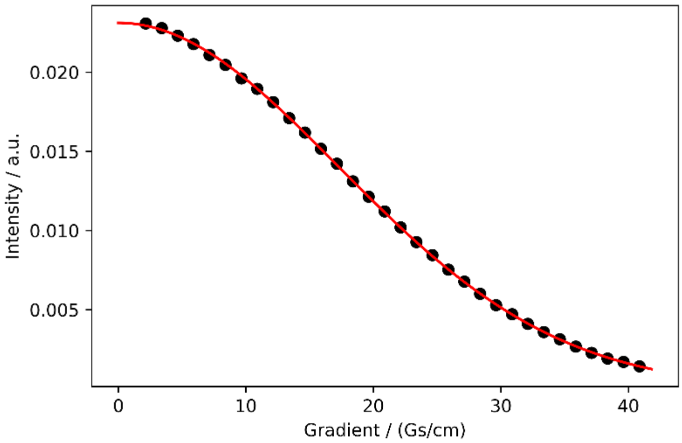

Cation

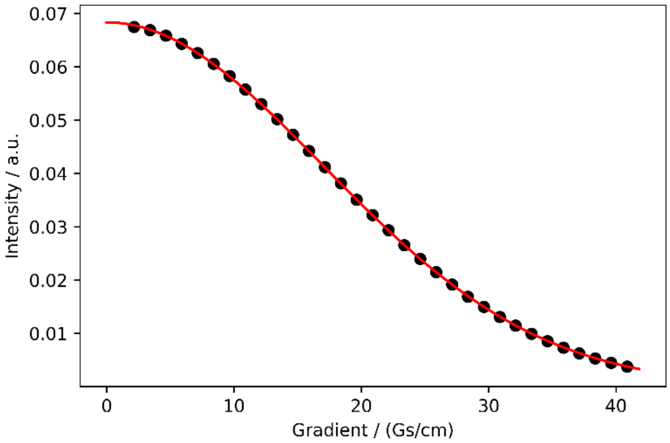

TMS

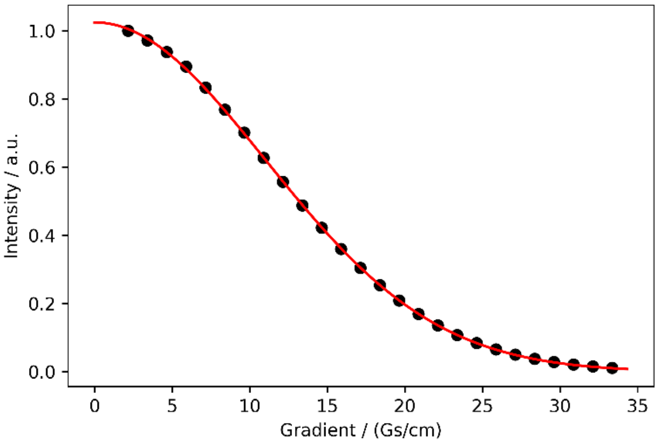

Ion Pair 5a, 1.0 mM, CD_2Cl_2

Anion

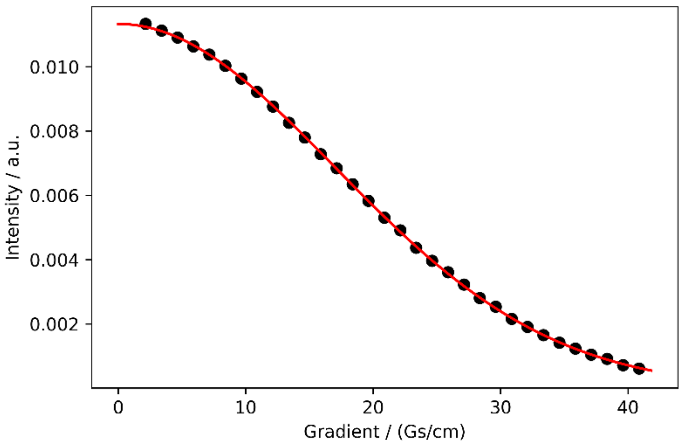

Cation

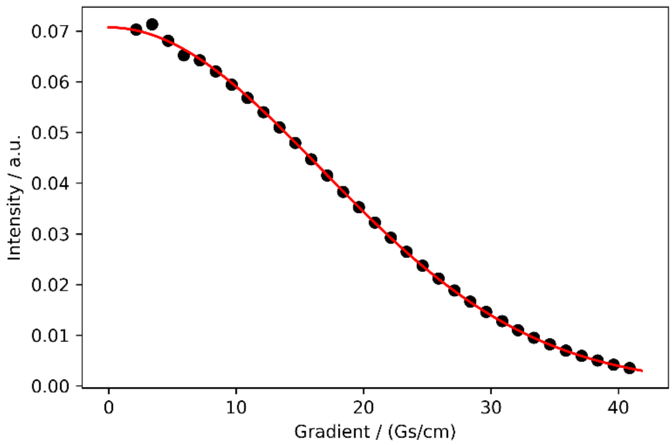

TMS

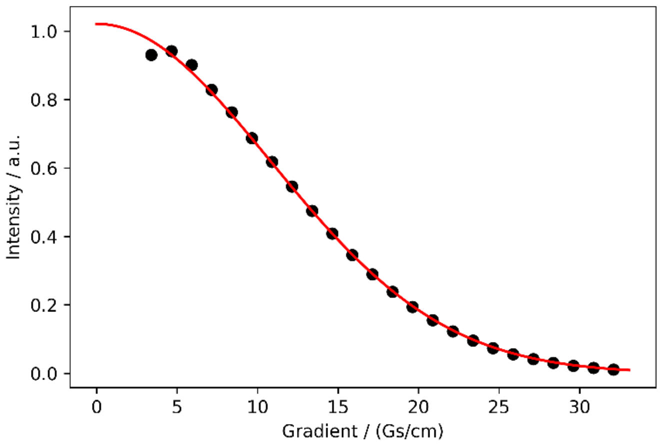

Ion Pair 5a, 5.0 mM, CD_2Cl_2

Anion

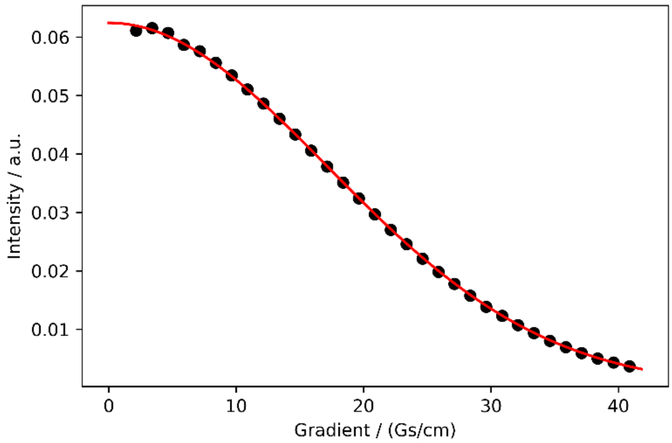

Cation

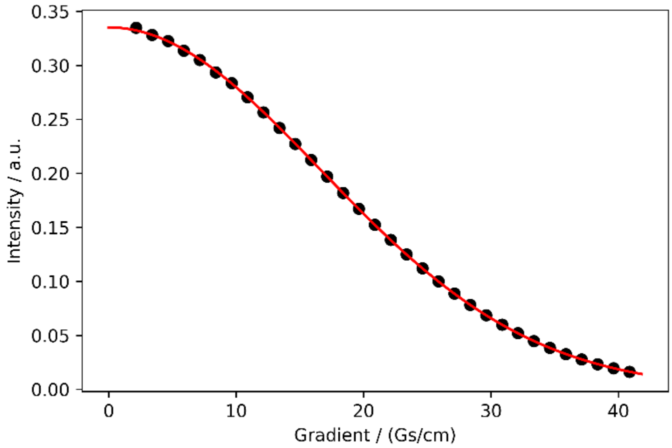

TMS

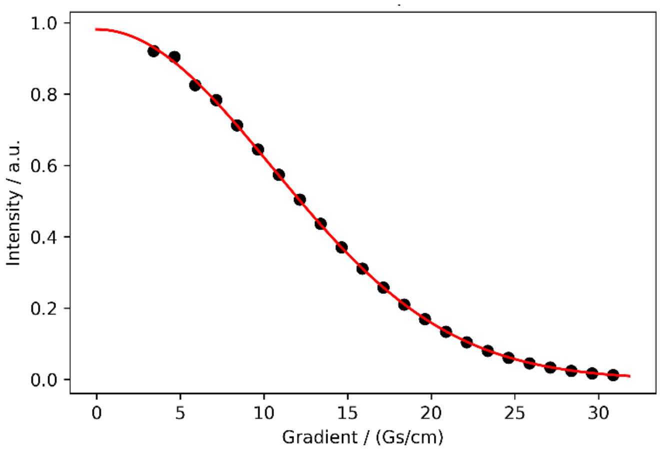

Ion Pair 6a, 0.05 mM, CD_2Cl_2

Anion

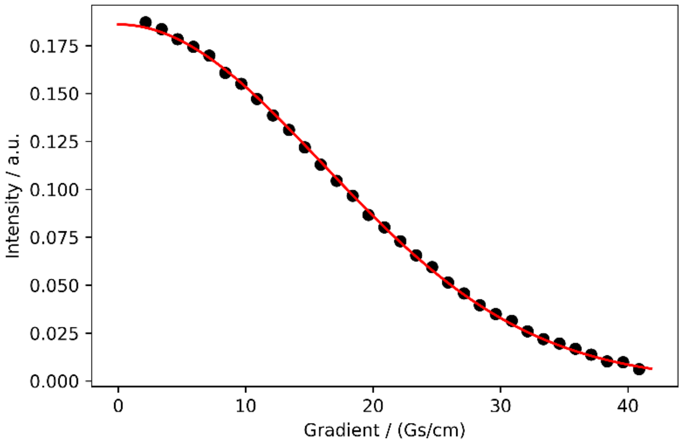

Cation

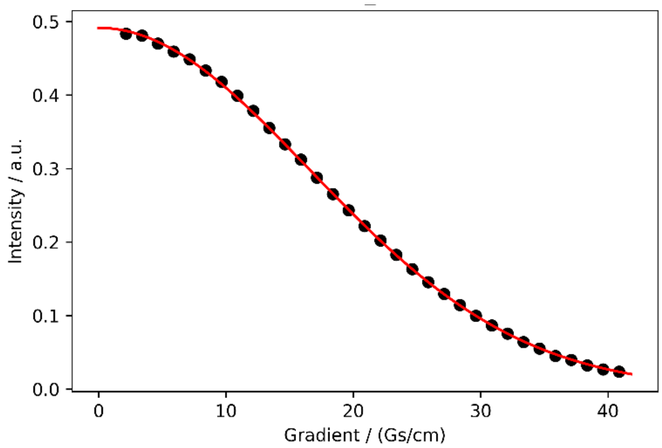

TMS

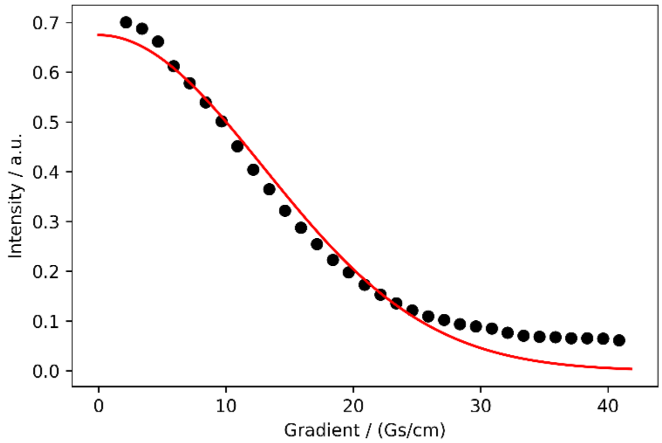

Ion Pair 6a, 0.5 mM, CD_2Cl_2

Anion

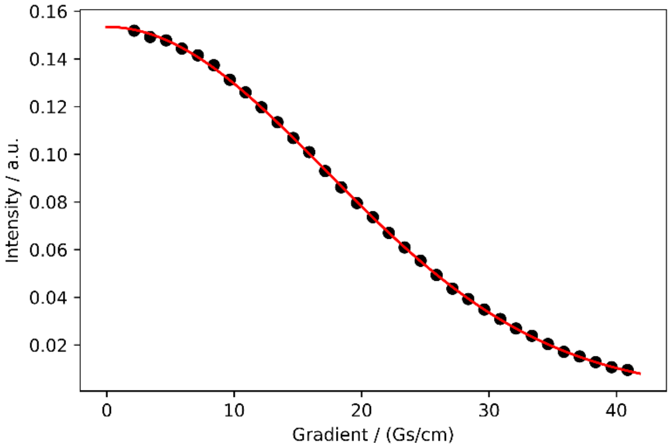

Cation

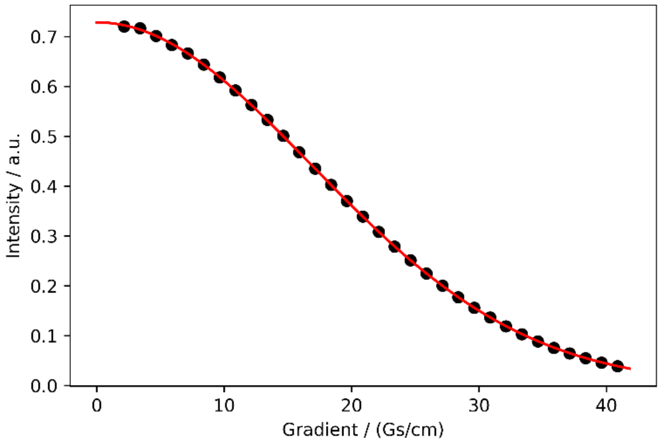

TMS

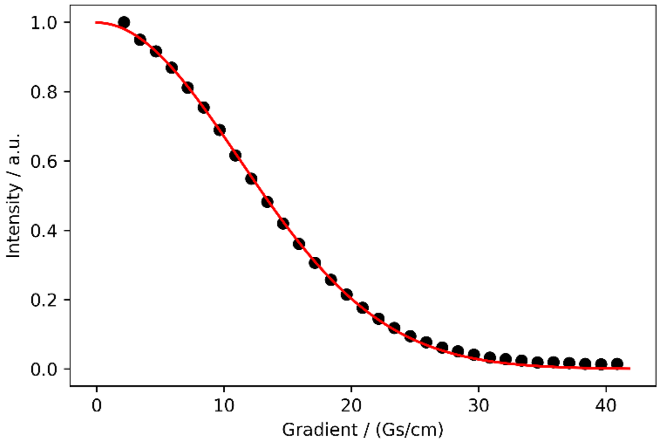

Ion Pair 6a, 1.0 mM, CD_2Cl_2

Anion

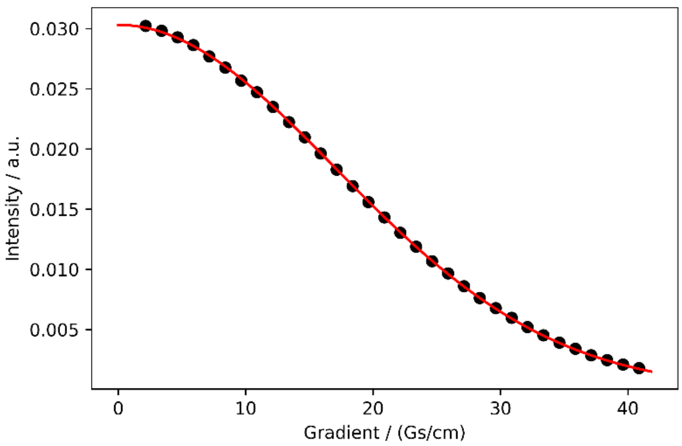

Cation

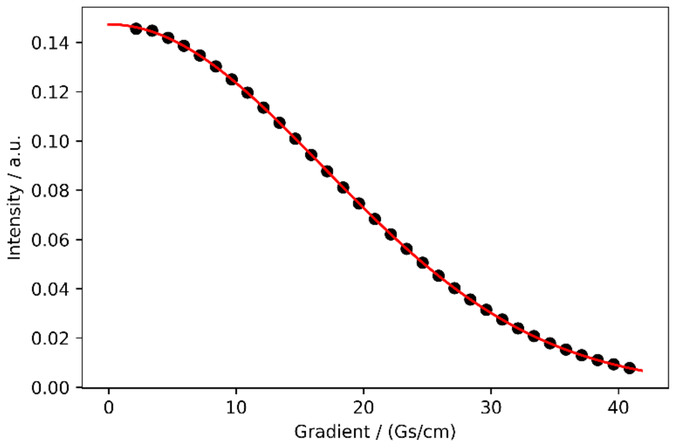

TMS

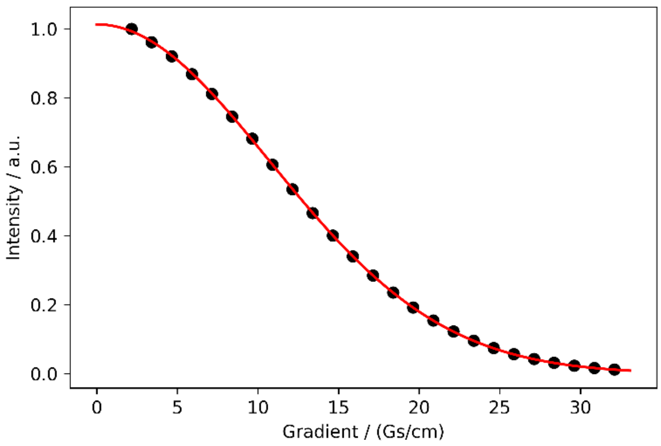

Ion Pair 6a, 5.0 mM, CD_2Cl_2

Anion

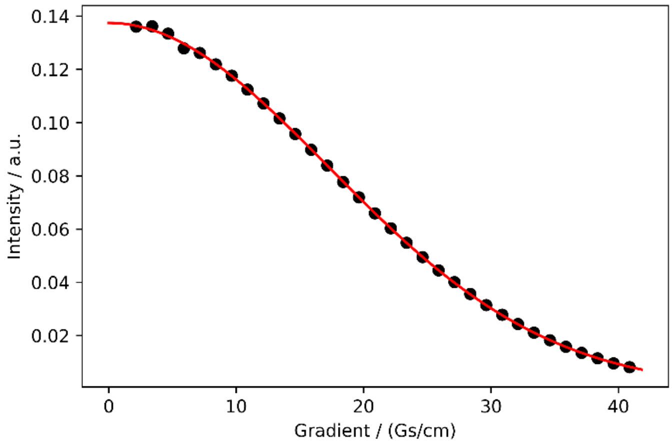

Cation

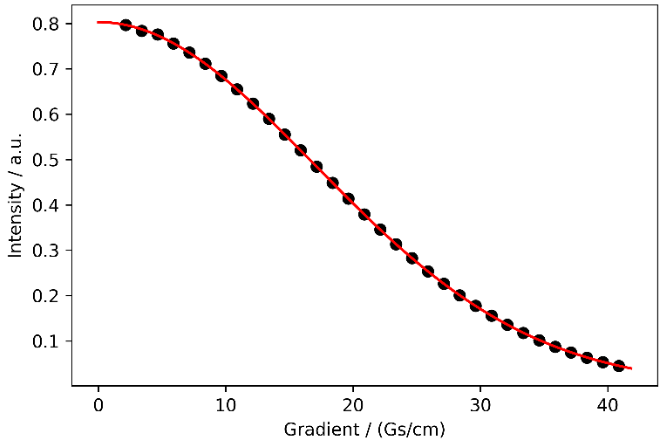

TMS

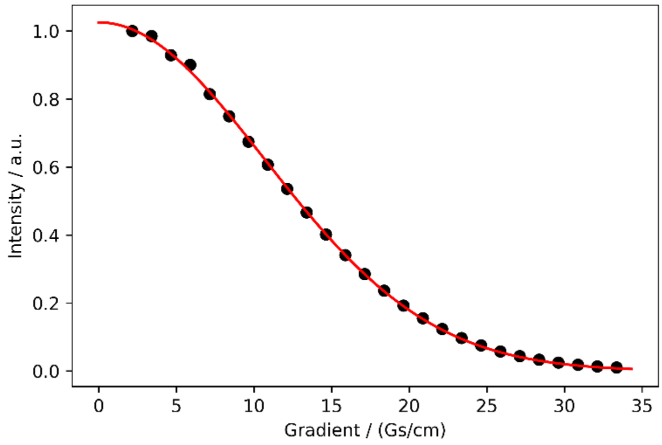

11. Computational General Information

All substrate structures were optimized with the B3LYP-D3 hybrid functional[26–28] with the 6-31+G(d) basis set.[29] Solvent effects for dichloromethane have been calculated with the SMD continuum solvation model.[30] This combination has worked well in previous studies of charge-separated intermediates.[31–34] Frequency and single point calculations were performed at the same level of theory. Thermochemical correction to 198.15 K has been applied to all found minima from unscaled vibrational frequencies obtained at the same level of theory. To lessen the impact of low-lying frequencies of large systems on entropy and enthalpy in an unpredictable manner, a free-rotor approximation for entropy as proposed by Grimme[35] and a quasi-harmonic treatment with a cutoff value of 100 cm^{-1} using Goodvibes[36] was applied. Free energies in solution have been corrected to the reference state of 1.0 mol L^{-1} at 298.15 K by adding 7.925 kJ mol^{-1} to the free energies ($G_{298,qh,corr}$). All reported calculations were done with Gaussian 16, Revision A.03[37] and B.01[38].

The conformational search was performed with Maestro-. For ion pair and additive salt systems, a set of 150 starting points was obtained by using the stochastic kick procedure invented by Saunders[40] and further developed by Sakic.[41] At a time, a set of 50 starting points was generated by a combination of the best, the second-best conformer, and the third-best conformer of the prior separately optimized anion and cation. In case on ion does not have more than two anion conformers, the last 50 starting points were obtained from the combination of the best anion conformer with the third best cation conformer. The following kick settings were used: Distance parameter: 3 Å, Minimal Distance 1.5 Å, Number of Fragments: 1, Number of Files: 50.

Starting points for the conformers of the ion pair systems were obtained from Julian Helberg. Conformers for the new systems with tetraphenyl phosphonium as cation (**3-6a**) were obtained by manual modification of the corresponding triphenyl methyl phosphonium containing systems, initially calculated by J. Helberg[3], whereby the methyl group was replaced by a phenyl group. The starting point of ion pair **6a** were obtained by manual modification of the starting points of ion pair **5a** whereas the methyl group was manually fluorinated. Starting points for the *n*-butyl chained cations were generated by manually extending the methylated ion conformers of J. Helberg. To represent tetraalkyl ammonium and tetraalkyl phosphonium cations (present in substrates **3c** and **3d**) without having to cover the entire conformational spaces of these largely flexible systems, the alkyl chains were restricted to their fully elongated, trans oriented state.

Starting points for the conformers of the sandwich cation and sandwich anion were obtained by sorting the optimized structures of the respective ion pair according to the total energy. The best six conformers were chosen and in combination with the best cation conformer the stochastic kick procedure invented by Saunders[40] and further developed by Sakic[41] was applied to generate 60 new substrate structures in total. The following kick settings were used: Distance parameter: 3 Å, Minimal Distance 1.5 Å, Number of Fragments: 1, Number of Files: 10.

The goal in calculating the respective triple ion conformers was to obtain the volumes based on the van der Waals cavities employed in the SMD continuum solvation model at the SMD(DCM)/B3LYP-D3/6-31+G(d) level of theory for the calculation of the volumes of ions based on the simulated concentrations in each respective conductivity model to compare the DOSY results with the results of the numerical simulations. Due to the heavy nature of the calculated systems (between 81 – 119 atoms) the less costly r^2SCAN-3c developed by Grimme[42] with Orca 5.0.3[43] was used for pre-optimizations, followed by single point calculations at the SMD(DCM)/B3LYP-D3/6-31+G(d) level of theory with Gaussian 16, Revision C.01[44].

The calculated ion volumes based on the van der Waals cavities employed in the SMD continuum solvation model at the SMD(DCM)/B3LYP-D3/6-31+G(d) level of theory with Gaussian 16 were used in the conductivity analysis (see Chapter 3). The calculated ion volumes of all computed species are summarized in Table S36.

Table S36. Calculated isotropic polarizabilities and ion volumes for cations, anions, and ion aggregates based on the van der Waals cavity used in the SMD solvation model at the SMD(DCM)/B3LYP-D3/6-31+G(d) level of theory.

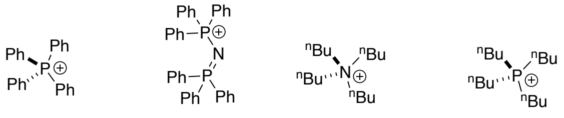

a_PPh$_4$ **b_PNP** **c_NBu$_4$** **d_PBu$_4$**

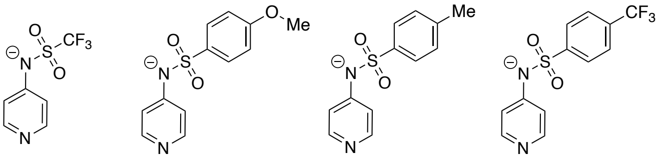

3 **4** **5** **6** **7**

Ion Volume Values									
Single Ions				1:1 Ion Pair		Triple Ion Complexes			
**Anion**	**Vol (Å^3)**	**Cation**	**Vol (Å^3)**	**Ion Pair**	**Vol (Å^3)**	**CAC**	**Vol (Å^3)**	**ACA**	**Vol (Å^3)**
**3**	215	**a_PPh4**	362	**3a**	570	**a3a**	925	**3a3**	782
**5**	266	**b_PNP**	559	**3b**	769	**b3b**	1321	**3b3**	981
**4**	285	**c_NBu4**	313	**3c**	522	**c3c**	830	**3c3**	733
**6**	295	**d_PBu4**	326	**3d**	536	**d3d**	859	**3d3**	748
**7**	69.7			**4a**	641	**a4a**	995	**4a4**	920
				**5a**	624	**a5a**	980	**5a5**	887
				**6a**	651	**a6a**	1007	**6a6**	941
				**7a**	430				
				**7b**	629				
				**7c**	382				
				**7d**	396				

Polarizability Values									
Single Ions				1:1 Ion Pair		Triple Ion Complexes			
**Anion**	**Iso Pol (Å^3)**	**Cation**	**Iso Pol (Å^3)**	**Ion Pair**	**Iso Pol (Å^3)**	**CAC**	**Iso Pol (Å^3)**	**ACA**	**Iso Pol (Å^3)**
**3**	82.8	**a_PPh4**	214	**3a**	300	**a3a**	509	**3a3**	383
**5**	137	**b_PNP**	335	**3b**	421	**b3b**	748	**3b3**	504
**4**	140	**c_NBu4**	132	**3c**	219	**c3c**	351	**3c3**	305
**6**	137	**d_PBu4**	144	**3d**	230	**d3d**	373	**3d3**	316
**7**	12.5			**4a**	354	**a4a**	563	**4a4**	490
				**5a**	351	**a5a**	559	**5a5**	484
				**6a**	350	**a6a**	557	**6a6**	481
				**7a**	227				
				**7b**	348				
				**7c**	145				
				**7d**	157				

Calculating the volume of the ion pair by summing up the respective single cation and anion volumes and comparing it to the computed ion pair volume, we check if there is a problem regarding the additivity of these ions. The results are listed in Table S37.

Table S37. Additivity analysis of ion aggregate volumes relative to the constituent ions based on SMD cavities calculated at the SMD(DCM)/B3LYP-D3/6-31+G(d) level of theory.

Ion aggregate	Constituent cation(s)	Constituent anion(s)	Sum of constituent volumes	Calculate volume	Volume difference[a]

**3a**	**a_PPh4**	**3**	577	570	-7.00
**3b**	**b_PNP**	**3**	774	769	-5.00
**3c**	**c_NBu4**	**3**	528	522	-6.00
**3d**	**d_PBu4**	**3**	541	536	-5.00
**4a**	**a_PPh4**	**4**	647	641	-6.00
**5a**	**a_PPh4**	**5**	628	624	-4.00
**6a**	**a_PPh4**	**6**	657	651	-6.00
**7a**	**a_PPh4**	**7**	432	430	-1.70
**7b**	**b_PNP**	**7**	629	629	0.30
**7c**	**c_NBu4**	**7**	383	382	-0.70
**7d**	**d_PBu4**	**7**	396	396	0.30
**a3a**	**a_PPh4**	**3**	939	925	-14.0
**a4a**	**b_PNP**	**3**	1333	1321	-12.0
**c3c**	**c_NBu4**	**3**	841	830	-11.0
**d3d**	**d_PBu4**	**3**	867	859	-8.00
**a4a**	**a_PPh4**	**4**	1009	995	-14.0
**a5a**	**a_PPh4**	**5**	990	980	-10.0
**a6a**	**a_PPh4**	**6**	1019	1007	-12.0
**3a3**	**a_PPh4**	**3**	792	782	-10.0
**4a4**	**b_PNP**	**3**	989	981	-8.00
**3c3**	**c_NBu4**	**3**	743	733	-10.0
**3d3**	**d_PBu4**	**3**	756	748	-8.00
**4a4**	**a_PPh4**	**4**	932	920	-12.0
**5a5**	**a_PPh4**	**5**	894	887	-7.00
**6a6**	**a_PPh4**	**6**	952	941	-11.0

[a] Volume difference = Calculated volume - Sum of constituent volumes.

Comparing calculated volumes with the sum of constituent ion volumes of a charged species show comparably low deviations. Therefore, the additivity for the ion volumes is given.

Another property we looked at is the isotropic polarizability ions at the SMD(DCM)/B3LYP-D3/6-31+G(d) level of theory which is given in Bohr^3 in the output file using Gaussian 16. With 1 Bohr = 0.529177 Å, we can convert these values to Å^3 by multiplying with $(0.529177)^3$ = 0.14818. The obtained isotropic polarizability values are summarized in Table S36 and correlated with the respective ion volumes in Figure S13.

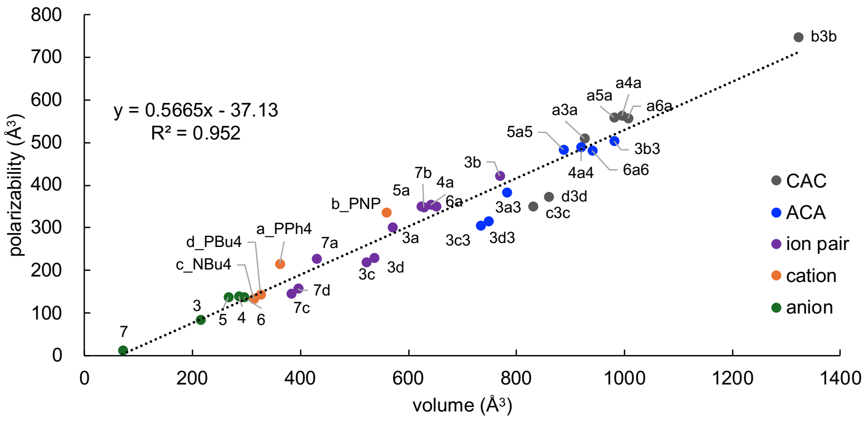

Figure S13. Correlation of calculated ion volumes (Å^3) vs. isotropic polarizability (Å^3) for all calculated single anions (green dots) and cations (orange dots), ion pairs (purple dots), cationic and anionic sandwich complexes (grey and blue dots).

Plotting the ion volumes vs. the isotropic polarizability gives a strong correlation which is remarkable considering the various type of species involved in this data set.

So far, the cation size seems to be a key factor regarding the final association pattern of the ion pair. To check whether the ion size is correlating with another association parameter, we calculated the mol fraction $\alpha/(\alpha+\beta)$ for the scaling factor α of the cationic sandwich association in the mixed model. The results are listed in Table S38. Additionally, we calculated the volume difference Δvolume (Å^3) between the cation and anion volumes.

Table S38. List of cation volumes (Å^3).

Ion aggregate	Constituent cation(s)	Volume cation (Å^3)	Constituent anion(s)	α/β	$\alpha/(\alpha+\beta)$	Volume anion (Å^3)	Volume difference[a]
**3a**	**a_PPh4**	362	**3**	44/21	0.68	215	147
**3b**	**b_PNP**	559	**3**	100/0	1.00	215	344
**3c**	**c_NBu4**	313	**3**	33/23	0.59	215	98.0
**4a**	**a_PPh4**	362	**4**	12/61	0.16	285	77.0
**5a**	**a_PPh4**	362	**5**	11/67	0.14	266	96.0
**6a**	**a_PPh4**	362	**6**	16/52	0.24	295	67.0

[a] Volume difference = cation volume - anion volume.

The mol fraction as well as the scaling factor α was plotted against the calculated volume difference Δvolume (Å^3). Both graphs in Figure S14 show a strong correlation for ion pairs **3a**, **3b**, and **3c** where cation changes while the anion remains the same. The is no correlation when comparing ion pairs with changing anions and constant cation.

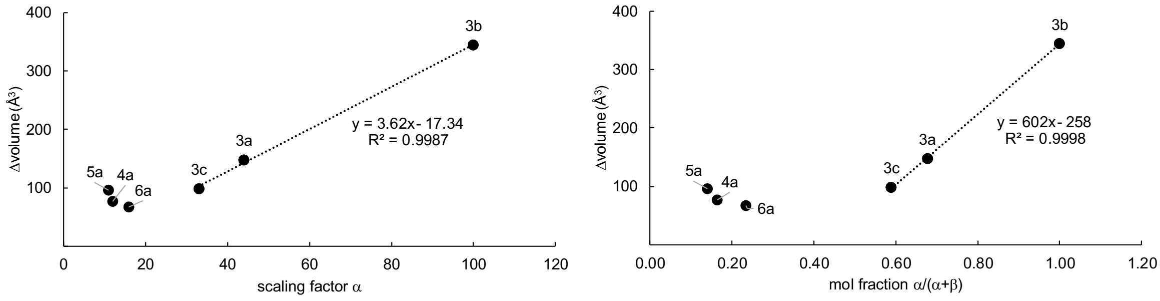

Figure S14. Left: correlation of scaling factor α with ion volume difference Δvolume (Å^3). Right: correlation of mol fraction $\alpha/(\alpha+\beta)$ with ion volume difference Δvolume (Å^3).

Assuming that other ion pairs such as **4a** would display a correlation with a similar slope when exchanging cation **a** (PPh_4^+) for cation **b** (PNP^+) we could predict the mol fraction for theoretical ion pair **4b** (see Figure S15).

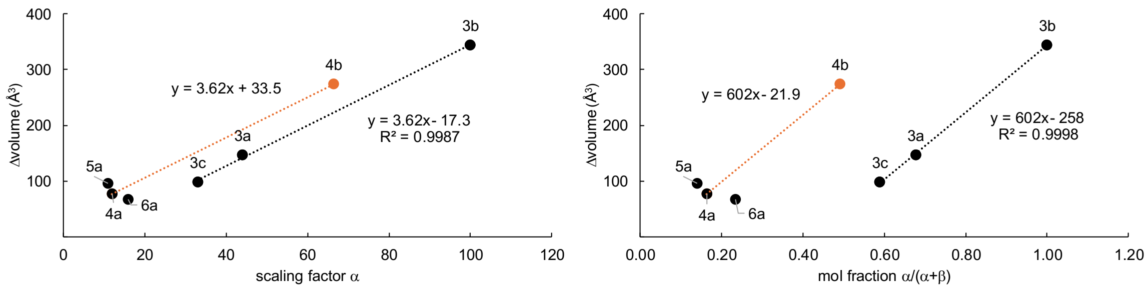

Figure S15. Left: correlation of scaling factor α with ion volume difference Δvolume (Å^3). Right: correlation of mol fraction $\alpha/(\alpha+\beta)$ with ion volume difference Δvolume (Å^3).

Thus, ion pair **4b** would have a scaling factor α of 66 and a mol fraction of 0.49 if this assumption is correct, which would indicate a predominated cationic sandwich association.

11.1 Optimized Conformers of Pyridinamide Ion Pairs in DCM

System 3b – computational data

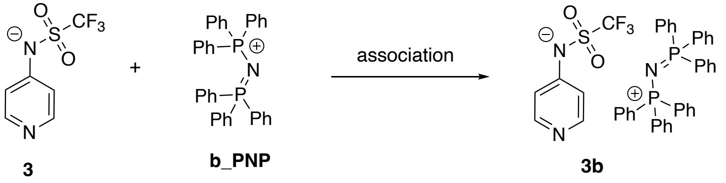

Figure S16. Ion association of pyridinamide ion pair **3b** and the single ions

The Boltzmann-averaged free reaction energy of the ion pairing of cat3b amounts to $\Delta G_{qh,298,corr}$ = 1.3 kJ mol^{-1} in DCM solution. Focusing only on the best conformers of the reactants and product the free reaction energy of the ion pairing of **3b** changes to $\Delta G_{qh,298,corr}$ = -1.8 kJ mol^{-1} in DCM solution. This result is mainly due to solvation effects since the gas phase free energy of the ion pairing amounts to $\Delta G_{qh,298}$ = -213.4 kJ mol^{-1}. In addition, we note that the contribution of the D3-dispersion correction amounts to ΔE_{disp} = -67.0 kJ mol^{-1} for the free energy in the DCM solution and in the gas phase. The blue marked cells show the Boltzmann-averaged values.

According to $\Delta G_{qh,298,corr}$ = -RT lnK and assuming R = 8.314 J K^{-1} mol^{-1} and T = 298.15 K, the respective equilibrium constant amounts to K(**3b**, DCM) = +2.06 for the best conformer of **3b** and to K(**3b**, DCM) = +0.60 for the Boltzmann-averaged free reaction energy. In this case the equilibrium constant K corresponds to the concentration of the reactants and products in the following way: K = [**3b**]/[**3**] [**b_PNP**] = [**3b**]/[**3**]^2.

Table S39. Energies for all systems shown in Figure S16.

System	E_{tot} SMD(DCM)/ B3LYP-D3/ 6-31+G(d)	H_{298} SMD(DCM)/ B3LYP-D3/ 6-31+G(d)	$G_{qh,298}$ SMD(DCM)/ B3LYP-D3/ 6-31+G(d)	$G_{qh,298,corr}$ SMD(DCM)/ B3LYP-D3/ 6-31+G(d)	Cavity Volume (Å^3)	Relative Population Parameter based on $G_{qh,298}$
**3**						
an3_001	**-1188.8384606**	**-1188.7186926**	**-1188.769809**	**-1188.766790**	**215**	0.91
an3_002	-1188.8357192	-1188.7160622	-1188.7673382	-1188.7643197	214	0.09
			**-1188.7693051**	**-1188.7662866**	**215**	
**b_PNP**						
cation_c_002	**-2127.413003**	**-2126.821082**	**-2126.913057**	**-2126.910039**	**560**	0.51
cation_c_003	-2127.412921	-2126.820643	-2126.912356	-2126.909338	559	0.24
cation_c_001	-2127.412908	-2126.820541	-2126.912386	-2126.909367	559	0.25

			**-2126.912720**	**-2126.909702**	**559**	
**3b**[a]						
cat3b_124_dcm_fr	**-3316.275809**	**-3315.561663**	**-3315.680224**	**-3315.677206**	**768**	0.29
cat3b_020_dcm_fr	-3316.275058	-3315.560851	-3315.679373	-3315.676355	770	0.12
cat3b_147_dcm_fr	-3316.274602	-3315.560287	-3315.678876	-3315.675857	768	0.07
cat3b_136m_dcm	-3316.274338	-3315.560086	-3315.678610	-3315.675591	770	0.05
cat3b_140_dcm_fr	-3316.274329	-3315.560084	-3315.678552	-3315.675533	771	0.05
cat3b_053_dcm_fr	-3316.273923	-3315.559646	-3315.677960	-3315.674942	763	0.03
cat3b_104_dcm_fr	-3316.274181	-3315.559715	-3315.677871	-3315.674853	770	0.02
cat3b_145_dcm_fr	-3316.273835	-3315.559443	-3315.677862	-3315.674844	771	0.02
cat3b_059_dcm_fr	-3316.272931	-3315.559001	-3315.677746	-3315.674727	766	0.02
cat3b_043_dcm_fr	-3316.273208	-3315.559194	-3315.677712	-3315.674693	769	0.02
cat3b_073_dcm_fr	-3316.272688	-3315.558601	-3315.677594	-3315.674576	771	0.02
cat3b_096_dcm_fr	-3316.273468	-3315.559242	-3315.677589	-3315.674570	772	0.02
cat3b_015_dcm_fr	-3316.272868	-3315.558895	-3315.677588	-3315.674570	769	0.02
cat3b_108_dcm_fr	-3316.273117	-3315.559030	-3315.677570	-3315.674552	770	0.02
cat3b_022_dcm_fr	-3316.272537	-3315.558623	-3315.677469	-3315.674450	770	0.02
cat3b_042_dcm_fr	-3316.272400	-3315.558590	-3315.677322	-3315.674304	772	0.01
cat3b_030_dcm_fr	-3316.272439	-3315.558496	-3315.677313	-3315.674295	770	0.01
cat3b_032_dcm_fr	-3316.273425	-3315.558924	-3315.677248	-3315.674230	772	0.01
cat3b_048_dcm_fr	-3316.272623	-3315.558500	-3315.677195	-3315.674177	769	0.01
cat3b_021_dcm_fr	-3316.272520	-3315.558424	-3315.677002	-3315.673984	770	0.01
**all**			**-3315.678519**	**-3315.675501**	**769**	
ΔE	-63.92	-57.47	+6.14	-1.79		
**all**			**+9.21**	**+1.28**		

[a] best 20 conformers according to $G_{qh,298}$ at SMD(DCM)/B3LYP-D3/6-31+G(d) level of theory.

Table S40. Energies of the best conformer for all systems shown in Figure S16 at different level of theory.

**System**	**E_{tot}[a] B3LYP-D3/ 6-31+G(d)**	**$G_{qh,298}$[a] B3LYP-D3/ 6-31+G(d)**	**E_{tot}[a] B3LYP/ 6-31+G(d)**	**$G_{qh,298}$[a] B3LYP/ 6-31+G(d)**	**E_{tot}[a] SMD(DCM)/ B3LYP/ 6-31+G(d)**	**$G_{qh,298,corr}$[a] SMD(DCM)/ B3LYP/ 6-31+G(d)**
**3**						
an3_001	-1188.770301	-1188.701344	-1188.753230	-1188.684273	-1188.821390	-1188.7494140

**b_PNP**						
cation_c_002	-2127.324579	-2126.824633	-2127.229977	-2126.730031	-2127.318402	-2126.815438
**3b[a]**						
cat3b_124	-3316.202835	-3315.607250	-3316.06563	-3315.470045	-3316.138605	-3315.540001
**ΔE(3b, 3, b)**	**-283.44**	**-213.38**	**-216.40**	**-146.35**	**+3.12**	**+65.25**

[a] using geometries optimized at SMD(DCM)/B3LYP-D3/6-31+G(d) level.

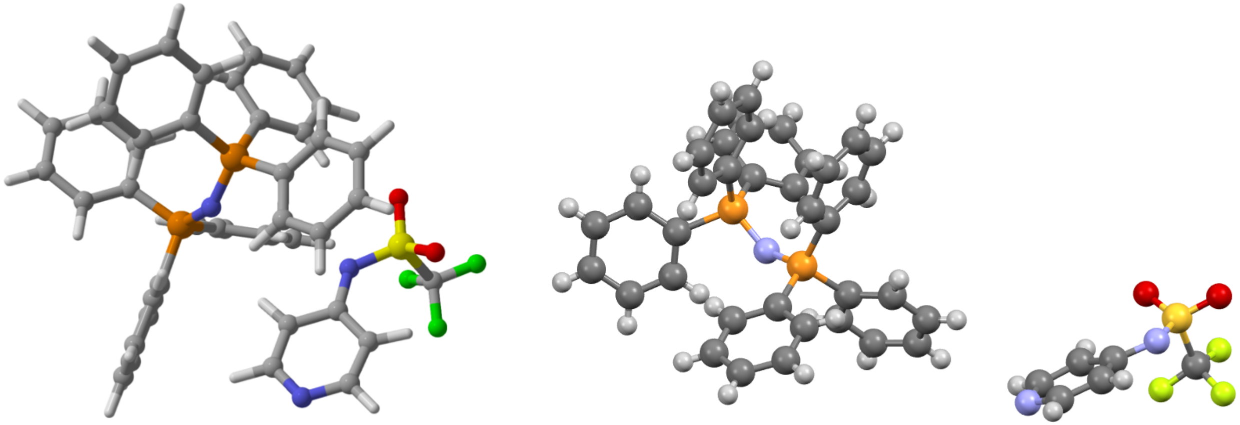

Figure S17. left: structure of conformer cat3b_124 calculated at SMD(DCM)/ B3LYP-D3/6-31+G(d) level of theory; right: crystal structure of one ion pair in catalyst **3b**.

System 3c – computational data

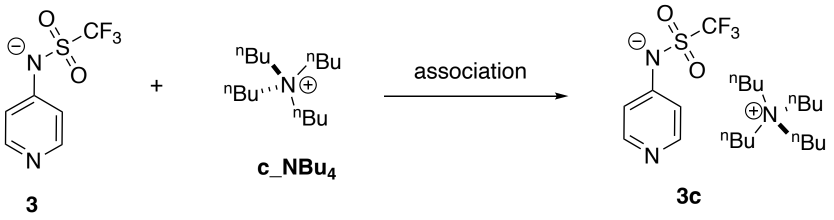

Figure S18. Ion association of pyridinamide ion pair **3c** and the single ions.

The Boltzmann-averaged free reaction energy of the ion pairing of **3c** amounts to $\Delta G_{qh,298,corr}$ = 7.3 kJ mol^{-1} in DCM solution. Focusing only on the best conformers of the reactants and product the free reaction energy of the ion pairing of **3c** changes to $\Delta G_{qh,298,corr}$ = +5.2 kJ mol^{-1} in DCM solution. This result is mainly due to solvation effects since the gas phase free energy of the ion pairing amounts to $\Delta G_{qh,298}$ = -243.8 kJ mol^{-1}. In addition, we note that the contribution of the D3-dispersion correction amounts to ΔE_{disp} = -50.0 kJ mol^{-1} for the free energy in the DCM solution and in the gas phase. The blue marked cells show the Boltzmann-averaged values.

According to $\Delta G_{qh,298,corr}$ = -RT lnK and assuming R = 8.314 J K^{-1} mol^{-1} and T = 298.15 K, the respective equilibrium constant amounts to K(**3c**, DCM) = +0.12 for the best conformer of **3c** and to K(**3c**, DCM) = +0.05 for the Boltzmann-averaged free reaction energy. In this case the equilibrium constant K corresponds to the concentration of the reactants and products in the following way: K = [**3c**]/[**3**] [**c_NBu4**] = [**3c**]/[**3**]^2.

Table S41. Energies for all systems shown in Figure S18.

System	E_{tot} SMD(DCM)/ B3LYP-D3/ 6-31+G(d)	H_{298} SMD(DCM)/ B3LYP-D3/ 6-31+G(d)	$G_{qh,298}$ SMD(DCM)/ B3LYP-D3/ 6-31+G(d)	$G_{qh,298,corr}$ SMD(DCM)/ B3LYP-D3/ 6-31+G(d)	Cavity Volume (Å^3)	Relative Population Parameter based on $G_{qh,298}$
**3**						
an3_001	**-1188.8384606**	**-1188.7186926**	**-1188.769809**	**-1188.766790**	215	0.91
an3_002	-1188.8357192	-1188.7160622	-1188.7673382	-1188.7643197	214	0.09
			**-1188.7693051**	**-1188.7662866**	**215**	
**c_NBu4**						
NBu4_002	**-686.095346**	**-685.564482**	**-685.637042**	**-685.634023**	313	0.99
NBu4_001c	-686.094074	-685.564092	-685.632815	-685.629797	313	0.01
NBu4_006	-686.090709	-685.558999	-685.630636	-685.627618	312	0.00
NBu4_005	-686.089721	-685.557688	-685.629512	-685.626493	312	0.00
NBu4_003	-686.089384	-685.557149	-685.628989	-685.625971	312	0.00

NBu4_004	-686.086662	-685.553694	-685.624302	-685.621283	312	0.00
			**-685.6369831**	**-685.6339647**	**313**	
**3c[a]**						
cat3c_001_dcm	-1874.956418	**-1874.302434**	**-1874.401539**	**-1874.398520**	522	0.10
cat3c_034_dcm	-1874.956217	-1874.302034	-1874.401302	-1874.398284	523	0.08
cat3c_028_dcm	-1874.956268	-1874.302058	-1874.401246	-1874.398228	522	0.07
cat3c_003m_dcm_fr	-1874.956347	-1874.302312	-1874.401237	-1874.398219	522	0.07
cat3c_027_dcm	**-1874.957013**	-1874.302207	-1874.401158	-1874.398140	521	0.07
cat3c_036mm_dcm_fr	-1874.956034	-1874.301936	-1874.401090	-1874.398072	523	0.06
cat3c_009_dcm	-1874.956094	-1874.301719	-1874.400973	-1874.397954	521	0.05
cat3c_031_dcm	-1874.956330	-1874.301906	-1874.400938	-1874.397920	520	0.05
cat3c_026_dcm	-1874.956044	-1874.301623	-1874.400868	-1874.397849	524	0.05
cat3c_029_dcm	-1874.955676	-1874.301445	-1874.400546	-1874.397528	523	0.03
cat3c_035bp_dcm_fr	-1874.956082	-1874.301776	-1874.400515	-1874.397497	523	0.03
cat3c_021_dcm	-1874.955681	-1874.301376	-1874.400392	-1874.397374	522	0.03
cat3c_057_dcm	-1874.955604	-1874.301212	-1874.400303	-1874.397284	522	0.03
cat3c_023_dcm	-1874.954437	-1874.300508	-1874.400231	-1874.397212	522	0.02
cat3c_033aa_dcm	-1874.955485	-1874.301102	-1874.400056	-1874.397038	522	0.02
cat3c_054_dcm	-1874.955693	-1874.301116	-1874.399912	-1874.396893	522	0.02
cat3c_072_dcm	-1874.955489	-1874.300937	-1874.399899	-1874.396880	521	0.02
cat3c_041a_dcm	-1874.955713	-1874.301023	-1874.399884	-1874.396866	521	0.02
cat3c_042_dcm	-1874.954777	-1874.300537	-1874.399813	-1874.396794	523	0.02
cat3c_002_dcm	-1874.955041	-1874.300786	-1874.399681	-1874.396663	523	0.01
all			**-1874.400652**	**-1874.397634**	**522**	
ΔE	-60.93	-52.57	+13.14	+5.22		
all			**+15.21**	**+7.28**		

[a] best 20 conformers according to $G_{qh,298}$ at SMD(DCM)/B3LYP-D3/6-31+G(d) level of theory.

Table S42. Energies of the best conformer for all systems shown in Figure S18 at different level of theory.

System	E_{tot}[a] B3LYP-D3/ 6-31+G(d)	$G_{qh,298}$[a] B3LYP-D3/ 6-31+G(d)	E_{tot}[a] B3LYP/ 6-31+G(d)	$G_{qh,298}$[a] B3LYP/ 6-31+G(d)	E_{tot}[a] SMD(DCM)/ B3LYP/ 6-31+G(d)	$G_{qh,298,corr}$[a] SMD(DCM)/ B3LYP/ 6-31+G(d)
**3**						

an3_001	-1188.770301	-1188.701344	-1188.753230	-1188.684273	-1188.821390	-1188.7494140
**c_NBu4**						
NBu4_002	-686.009748	-685.551444	-685.954640	-685.496336	-686.040238	-685.578915
**3c**[a]						
cat3c_001_dcm	-1874.900521	-1874.345642	-1874.809296	-1874.254417	-1874.865193	-1874.307295
**ΔE(3c, 3, c)**	-316.30	-243.79	-266.29	-193.78	-9.36	+55.23

[a] using geometries optimized at SMD(DCM)/B3LYP-D3/6-31+G(d) level.

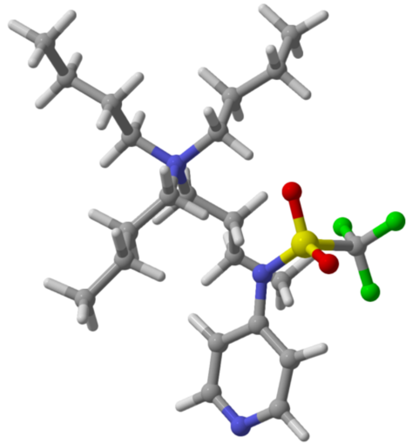

Figure S19. Structure of conformer cat3c_001 calculated at SMD(DCM)/ B3LYP-D3/6-31+G(d) level of theory

System 3d – computational data

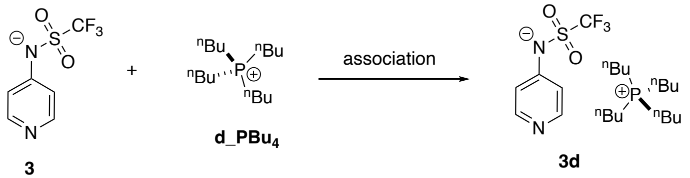

Figure S20. Ion association of pyridinamide ion pair **3d** and the single ions.

The Boltzmann-averaged free reaction energy of the ion pairing of **3d** amounts to $\Delta G_{qh,298,corr}$ = +1.1 kJ mol^{-1} in DCM solution. Focusing only on the best conformers of the reactants and product the free reaction energy of the ion pairing of **3d** changes to $\Delta G_{qh,298,corr}$ = +1.3 kJ mol^{-1} in DCM solution. This result is mainly due to solvation effects since the gas phase free energy of the ion pairing amounts to $\Delta G_{qh,298}$ = -250.4 kJ mol^{-1}. In addition, we note that the contribution of the D3-dispersion correction amounts to ΔE_{disp} = +176.3 kJ mol^{-1} for the free energy in the DCM solution and in the gas phase. The blue marked cells show the Boltzmann-averaged values.

According to $\Delta G_{qh,298,corr} = -RT \ln K$ and assuming R = 8.314 J K^{-1} mol^{-1} and T = 298.15 K, the respective equilibrium constant amounts to K(**3d**, DCM) = +0.59 for the best conformer of **3d** and to K to K(**3d**, DCM) = +0.65 for the Boltzmann-averaged free reaction energy. In this case the equilibrium constant K corresponds to the concentration of the reactants and products in the following way: K = [**3d**]/[**3**] [**d_PBu4**] = [**3d**]/[**3**]^2.

Table S43. Energies for all systems shown in Figure S20.

System	E_{tot} SMD(DCM)/ B3LYP-D3/ 6-31+G(d)	H_{298} SMD(DCM)/ B3LYP-D3/ 6-31+G(d)	$G_{qh,298}$ SMD(DCM)/ B3LYP-D3/ 6-31+G(d)	$G_{qh,298,corr}$ SMD(DCM)/ B3LYP-D3/ 6-31+G(d)	Cavity Volume (Å^3)	Relative Population Parameter based on $G_{qh,298}$
**3**						
an3_001	**-1188.8384606**	**-1188.7186926**	**-1188.769809**	**-1188.766790**	**215**	0.91
an3_002	-1188.8357192	-1188.7160622	-1188.7673382	-1188.7643197	214	0.09
			**-1188.7693051**	**-1188.7662866**	**215**	
**d_PBu4**						
PBu4_001_dcm_fr	**-972.730410**	**-972.207922**	**-972.283263**	**-972.280245**	**326**	0.71
PBu4_002b_pos	-972.729488	-972.206599	-972.282049	-972.279030	327	0.20
PBu4_003_dcm_fr	-972.727645	-972.204727	-972.280491	-972.277472	326	0.04
PBu4_005_dcm_fr	-972.728208	-972.205291	-972.280338	-972.277319	326	0.03
PBu4_004_dcm_fr	-972.727749	-972.204384	-972.279426	-972.276408	326	0.01
PBu4_006_dcm_fr	-972.726367	-972.203286	-972.278751	-972.275732	326	0.01

PBu4_007_dcm_fr	-972.725758	-972.202871	-972.278552	-972.275534	327	0.00
			**-972.282730**	**-972.279711**	**326**	
**3d[a]**						
cat3d_031_dcm_fr	-2161.591204	-2160.946455	**-2161.049248**	**-2161.046230**	536	0.05
cat3d_041a_dcm_fr	-2161.590889	-2160.946323	-2161.049170	-2161.046152	535	0.04
cat3d_027_dcm_fr	**-2161.591598**	**-2160.946633**	-2161.049140	-2161.046122	536	0.04
cat3d_029_dcm_fr	-2161.590751	-2160.946099	-2161.049124	-2161.046106	537	0.04
cat3d_001_dcm_fr	-2161.591275	-2160.946562	-2161.049074	-2161.046055	536	0.04
cat3d_013_dcm_fr	-2161.591276	-2160.946557	-2161.049068	-2161.046049	536	0.04
cat3d_034_dcm_fr	-2161.590791	-2160.946235	-2161.049054	-2161.046035	537	0.04
cat3d_057_dcm_fr	-2161.591600	-2160.946514	-2161.049037	-2161.046019	536	0.04
cat3d_004_dcm_fr	-2161.590797	-2160.946216	-2161.049006	-2161.045988	537	0.04
cat3d_020p_dcm_fr	-2161.590748	-2160.945995	-2161.048963	-2161.045945	536	0.04
cat3d_011_dcm_fr	-2161.590818	-2160.946070	-2161.048929	-2161.045911	537	0.03
cat3d_033a_dcm_fr	-2161.590646	-2160.945991	-2161.048913	-2161.045895	538	0.03
cat3d_025p_dcm_fr	-2161.591113	-2160.946250	-2161.048881	-2161.045862	537	0.03
cat3d_052_dcm_fr	-2161.590437	-2160.945840	-2161.048854	-2161.045835	535	0.03
cat3d_028_dcm_fr	-2161.590914	-2160.946146	-2161.048824	-2161.045805	536	0.03
cat3d_008_dcm_fr	-2161.590811	-2160.946001	-2161.048782	-2161.045764	537	0.03
cat3d_018_dcm_fr	-2161.591009	-2160.946197	-2161.048743	-2161.045724	537	0.03
cat3d_003_dcm_fr	-2161.591285	-2160.946372	-2161.048742	-2161.045723	536	0.03
cat3d_054_dcm_fr	-2161.591052	-2160.946085	-2161.048724	-2161.045706	535	0.03
cat3d_007p_dcm_fr	-2161.591297	-2160.946399	-2161.048721	-2161.045702	536	0.03
**all**			**-2161.048612**	**-2161.045594**	**536**	
ΔE	-59.68	-52.56	+9.24	+1.31		
**all**			**+8.99**	**+1.06**		

[a] best 20 conformers according to $G_{qh,298}$ at SMD(DCM)/B3LYP-D3/6-31+G(d) level of theory.

Table S44. Energies of the best conformer for all systems shown in Figure S20 at different level of theory.

**System**	**E_{tot}[a] B3LYP-D3/ 6-31+G(d)**	**$G_{qh,298}$[a] B3LYP-D3/ 6-31+G(d)**	**E_{tot}[a] B3LYP/ 6-31+G(d)**	**$G_{qh,298}$[a] B3LYP/ 6-31+G(d)**	**E_{tot}[a] SMD(DCM)/ B3LYP/ 6-31+G(d)**	**$G_{qh,298,corr}$[a] SMD(DCM)/ B3LYP/ 6-31+G(d)**
**3**						

an3_001	-1188.770301	-1188.701344	-1188.753230	-1188.684273	-1188.821390	-1188.7494140
**d_PBu4**						
PBu4_001	-972.643814	-972.196667	-972.679808	-972.232661	-972.593212	-972.143046
**3d**[a]						
cat3d_031	-2161.535344	-2160.993388	-2161.448224	-2160.906268	-2161.504084	-2160.959110
**ΔE(3d, 3, d)**	**-318.29**	**-250.41**	**-39.87**	**+28.00**	**-234.94**	**-174.99**

[a] using geometries optimized at SMD(DCM)/B3LYP-D3/6-31+G(d) level.

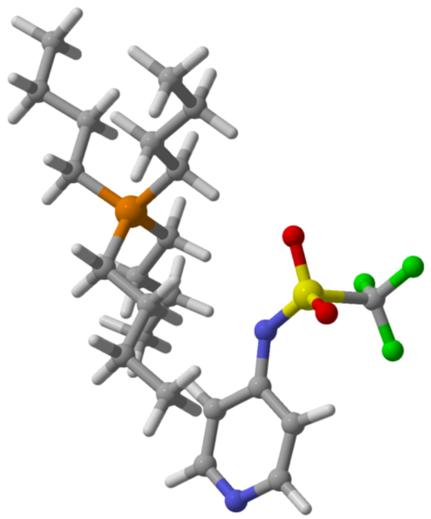

Figure S21. structure of conformer cat3d_008 calculated at SMD(DCM)/ B3LYP-D3/6-31+G(d) level of theory

System 5a – computational data

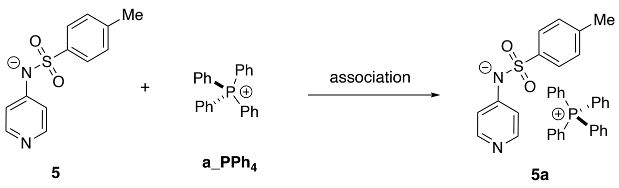

Figure S22. Ion association of pyridinamide ion pair **5a** and the single ions.

The Boltzmann-averaged free reaction energy of the ion pairing of **5a** amounts to $\Delta G_{qh,298,corr}$ = -3.8 kJ mol^{-1} in DCM solution. Focusing only on the best conformers of the reactants and product the free reaction energy of the ion pairing of **5a** changes to $\Delta G_{qh,298,corr}$ = -4.8 kJ mol^{-1} in DCM solution. This result is mainly due to solvation effects since the gas phase free energy of the ion pairing amounts to $\Delta G_{qh,298}$ = -224.4 kJ mol^{-1}. In addition, we note that the contribution of the D3-dispersion correction amounts to ΔE_{disp} = +136.5 kJ mol^{-1} for the free energy in the DCM solution and in the gas phase. The blue marked cells show the Boltzmann-averaged values.

According to $\Delta G_{qh,298,corr}$ = -RT lnK and assuming R = 8.314 J K^{-1} mol^{-1} and T = 298.15 K, the respective equilibrium constant amounts to K(**5a**, DCM) = +7.0 for the best conformer of **5a** and to K to K(**5a**, DCM) = +4.5 for the Boltzmann-averaged free reaction energy. In this case the equilibrium constant K corresponds to the concentration of the reactants and products in the following way: K = [**5a**]/[**5**] [**a_PPh4**] = [**5a**]/[**5**]^2.

Table S45. Energies for all systems shown in Figure S22.

System	E_{tot} SMD(DCM)/ B3LYP-D3/ 6-31+G(d)	H_{298} SMD(DCM)/ B3LYP-D3/ 6-31+G(d)	$G_{qh,298}$ SMD(DCM)/ B3LYP-D3/ 6-31+G(d)	$G_{qh,298,corr}$ SMD(DCM)/ B3LYP-D3/ 6-31+G(d)	Cavity Volume (Å^3)	Relative Population Parameter based on $G_{qh,298}$
**5**						
an5_001_dcm	**-1122.186986**	**-1121.960277**	**-1122.016776**	**-1122.013757**	**266**	0.82
an5_002a_dcm	-1122.184655	-1121.958007	-1122.014729	-1122.011711	267	0.09
an5_003_dcm	-1122.184683	-1121.957984	-1122.014655	-1122.011636	267	0.09
			**-1122.016400**	**-1122.013381**	**266**	
**a_PPh4**						
PPh4_003a	-1267.9099858	**-1267.5207058**	**-1267.5885298**	**-1267.5855113**	**360**	0.46
PPh4_001	-1267.9100736	-1267.5203936	-1267.5881566	-1267.5851381	364	0.31
PPh4_002f	**-1267.9100864**	-1267.5202704	-1267.5878314	-1267.5848129	362	0.22
			**-1267.5882580**	**-1267.5852395**	**362**	

**5a**[a]						
cat5a_012_dcm_fr	**-2390.123118**	**-2389.504272**	**-2389.604120**	**-2389.601102**	**625**	0.13
cat5a_078m_dcm_fr	-2390.122167	-2389.503803	-2389.603890	-2389.600871	621	0.10
cat5a_041_dcm_fr	-2390.121537	-2389.503322	-2389.603789	-2389.600771	624	0.09
cat5a_032_dcm_fr	-2390.121516	-2389.503282	-2389.603532	-2389.600514	623	0.07
cat5a_028_dcm_fr	-2390.121504	-2389.503143	-2389.603493	-2389.600474	625	0.07
cat5a_013_dcm_fr	-2390.121659	-2389.503236	-2389.603382	-2389.600363	624	0.06
cat5a_063_dcm_fr	-2390.122080	-2389.503363	-2389.603183	-2389.600164	625	0.05
cat5a_069_dcm_fr	-2390.121584	-2389.503089	-2389.603162	-2389.600143	626	0.05
cat5a_026_dcm_fr	-2390.121046	-2389.502972	-2389.603153	-2389.600135	623	0.05
cat5a_037_dcm_fr	-2390.120816	-2389.502607	-2389.602888	-2389.599869	624	0.04
cat5a_065am_dcm_fr	-2390.121449	-2389.502741	-2389.602660	-2389.599641	625	0.03
cat5a_057_dcm_fr	-2390.121561	-2389.502919	-2389.602533	-2389.599515	623	0.02
cat5a_046_dcm_fr	-2390.119762	-2389.501712	-2389.602329	-2389.599311	620	0.02
cat5a_018_dcm_fr	-2390.120212	-2389.502005	-2389.602274	-2389.599256	622	0.02
cat5a_048a_dcm_fr	-2390.120156	-2389.501910	-2389.602252	-2389.599233	625	0.02
cat5a_070a_dcm_fr	-2390.120100	-2389.501971	-2389.602233	-2389.599215	621	0.02
cat5a_061_dcm_fr	-2390.120222	-2389.501959	-2389.602151	-2389.599133	625	0.02
cat5a_083_dcm_fr	-2390.121484	-2389.502563	-2389.602024	-2389.599005	624	0.01
cat5a_003_dcm_fr	-2390.121455	-2389.502636	-2389.602022	-2389.599004	621	0.01
cat5a_020_dcm_fr	-2390.120352	-2389.501930	-2389.602002	-2389.598984	624	0.01
**all**			**-2389.603068**	**-2389.600049**	**624**	
ΔE	-68.38	-61.15	+3.11	-4.81		
**all**			**+4.18**	**-3.75**		

[a] best 20 conformers according to $G_{qh,298}$ at SMD(DCM)/B3LYP-D3/6-31+G(d) level of theory.

Table S46. Energies of the best conformer for all systems shown in Figure S22 at different level of theory.

**System**	**E_{tot}[a] B3LYP-D3/ 6-31+G(d)**	**$G_{qh,298}$[a] B3LYP-D3/ 6-31+G(d)**	**E_{tot}[a] B3LYP/ 6-31+G(d)**	**$G_{qh,298}$[a] B3LYP/ 6-31+G(d)**	**E_{tot}[a] SMD(DCM)/ B3LYP/ 6-31+G(d)**	**$G_{qh,298,corr}$[a] SMD(DCM)/ B3LYP/ 6-31+G(d)**
**5**						
an5_001	-1122.102832	-1121.932622	-1122.158865	-1121.988655	-1122.074711	-1121.901483
**a_PPh4**						

PPh4_003a	-1267.828115	-1267.506659	-1267.778739	-1267.457283	-1267.860609	-1267.5361348
**5a**[a]						
cat5a_012	-2390.043728	-2389.524730	-2389.934069	-2389.415071	-2390.013459	-2389.491443
**ΔE(5a, 5, a)**	**-285.83**	**-218.53**	**-228.13**	**-160.83**	**-0.54**	**+58.83**

[a] using geometries optimized at SMD(DCM)/B3LYP-D3/6-31+G(d) level.

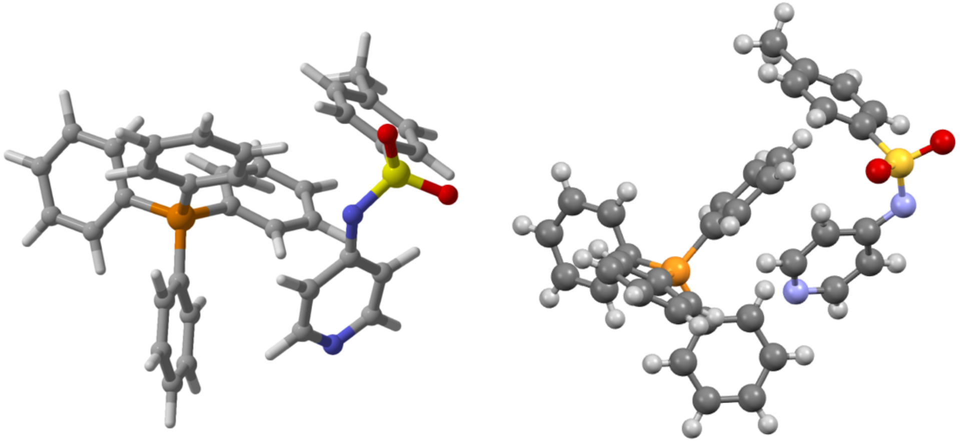

Figure S23. left: structure of conformer cat5a_012 calculated at SMD(DCM)/ B3LYP-D3/6-31+G(d) level of theory; right: crystal structure of one ion pair in catalyst **5a**.

System 6a – computational data

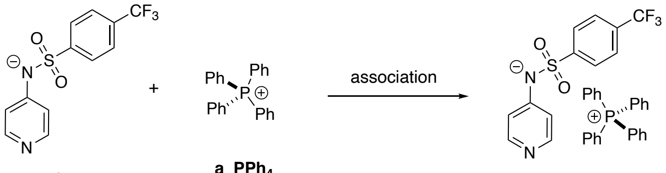

Figure S24. Ion association of pyridinamide ion pair **6a** and the single ions.

The Boltzmann-averaged free reaction energy of the ion pairing of **6a** amounts to $\Delta G_{qh,298,corr}$ = -3.8 kJ mol^{-1} in DCM solution. Focusing only on the best conformers of the reactants and product the free reaction energy of the ion pairing of **6a** changes to $\Delta G_{qh,298,corr}$ = -5.3 kJ mol^{-1} in DCM solution. This result is mainly due to solvation effects since the gas phase free energy of the ion pairing amounts to $\Delta G_{qh,298}$ = -216.2 kJ mol^{-1}. In addition, we note that the contribution of the D3-dispersion correction amounts to ΔE_{disp} = +115.8 kJ mol^{-1} for the free energy in the DCM solution and in the gas phase. The blue marked cells show the Boltzmann-averaged values.

According to $\Delta G_{qh,298,corr}$ = -RT lnK and assuming R = 8.314 J K^{-1} mol^{-1} and T = 298.15 K, the respective equilibrium constant amounts to K(**6a**, DCM) = 8.5 for the best conformer of **6a** and to K to K(**6a**, DCM) = 4.6 for the Boltzmann-averaged free reaction energy. In this case the equilibrium constant K corresponds to the concentration of the reactants and products in the following way: K = [**6a**]/[**6**] [**a_PPh4**] = [**6a**]/[**6**]^2.

Table S47. Energies for all systems shown in Figure S24.

System	E_{tot} SMD(DCM)/ B3LYP-D3/ 6-31+G(d)	H_{298} SMD(DCM)/ B3LYP-D3/ 6-31+G(d)	$G_{qh,298}$ SMD(DCM)/ B3LYP-D3/ 6-31+G(d)	$G_{qh,298,corr}$ SMD(DCM)/ B3LYP-D3/ 6-31+G(d)	Cavity Volume (Å^3)	Relative Population Parameter based on $G_{qh,298}$
**6**						
an6_001	**-1419.929810**	**-1419.724473**	**-1419.784896**	**-1419.781877**	**295**	0.89
an6_002	-1419.927369	-1419.722133	-1419.782897	-1419.779879	294	0.11
			**-1419.784681**	**-1419.781662**	**295**	
**a_PPh4**						
PPh4_003a	-1267.9099858	**-1267.5207058**	**-1267.5885298**	**-1267.5855113**	**360**	0.46
PPh4_001	-1267.9100736	-1267.5203936	-1267.5881566	-1267.5851381	364	0.31
PPh4_002f	**-1267.9100864**	-1267.5202704	-1267.5878314	-1267.5848129	362	0.22
			**-1267.5882580**	**-1267.5852395**	**362**	

**6a**[a]						
cat6a_012_dcm	-2687.866218	**-2687.269030**	**-2687.372424**	**-2687.369405**	**651**	0.16
cat6a_062_dcm	-2687.865545	-2687.268462	-2687.371852	-2687.368833	652	0.09
cat6a_077_dcm	-2687.865538	-2687.268342	-2687.371699	-2687.368681	649	0.07
cat6a_049_dcm	-2687.864657	-2687.267827	-2687.371664	-2687.368645	650	0.07
cat6a_003_dcm	**-2687.866341**	-2687.268788	-2687.371615	-2687.368597	651	0.07
cat6a_075p_dcm	-2687.865276	-2687.268285	-2687.371478	-2687.368459	651	0.06
cat6a_040_dcm	-2687.864450	-2687.267618	-2687.371469	-2687.368450	648	0.06
cat6a_056_dcm	-2687.865655	-2687.268398	-2687.371412	-2687.368394	653	0.05
cat6a_032_dcm	-2687.864713	-2687.267699	-2687.371402	-2687.368384	651	0.05
cat6a_028m_dcm	-2687.864474	-2687.267546	-2687.371337	-2687.368319	649	0.05
cat6a_002_dcm	-2687.865084	-2687.268002	-2687.371209	-2687.368190	653	0.04
cat6a_021_dcm	-2687.864480	-2687.267547	-2687.371013	-2687.367994	653	0.04
cat6a_026p_dcm	-2687.864470	-2687.267495	-2687.370956	-2687.367937	652	0.03
cat6a_068_dcm	-2687.864753	-2687.267464	-2687.370856	-2687.367837	653	0.03
cat6a_064m_dcm	-2687.864473	-2687.267166	-2687.370527	-2687.367509	652	0.02
cat6a_041_dcm	-2687.863090	-2687.266142	-2687.370069	-2687.367051	649	0.01
cat6a_047_dcm	-2687.863096	-2687.266094	-2687.369983	-2687.366965	649	0.01
cat6a_016_dcm	-2687.863649	-2687.266488	-2687.369931	-2687.366912	652	0.01
cat6a_048_dcm	-2687.862693	-2687.265908	-2687.369813	-2687.366795	651	0.01
cat6a_018_dcm	-2687.862898	-2687.266030	-2687.369766	-2687.366748	649	0.01
**all**			**-2687.371356**	**-2687.368337**	**641**	
ΔE	-69.43	-62.62	+2.63	-5.30		
**all**			**+4.16**	**-3.77**		

[a] best 20 conformers according to $G_{qh,298}$ at SMD(DCM)/B3LYP-D3/6-31+G(d) level of theory.

Table S48. Energies of the best conformer for all systems shown in Figure S24 at different level of theory.

**System**	**E_{tot}[a] B3LYP-D3/ 6-31+G(d)**	**$G_{qh,298}$[a] B3LYP-D3/ 6-31+G(d)**	**E_{tot}[a] B3LYP/ 6-31+G(d)**	**$G_{qh,298}$[a] B3LYP/ 6-31+G(d)**	**E_{tot}[a] SMD(DCM)/ B3LYP/ 6-31+G(d)**	**$G_{qh,298,corr}$[a] SMD(DCM)/ B3LYP/ 6-31+G(d)**
**6**						
an6_001	-1419.853500	-1419.708586	-1419.823428	-1419.678514	-1419.823428	-1419.675496
**a_PPh4**						

PPh4_003a	-1267.828115	-1267.506659	-1267.778739	-1267.457283	-1267.860609	-1267.5361348
**6a[a]**						
cat6a_012	-2687.791372	-2687.297578	-2687.679722	-2687.185928	-2687.754568	-2687.257755
**ΔE(6a, 6, a)**	**-288.17**	**-216.17**	**-203.62**	**-131.62**	**-185.18**	**-121.10**

[a] using geometries optimized at SMD(DCM)/B3LYP-D3/6-31+G(d) level.

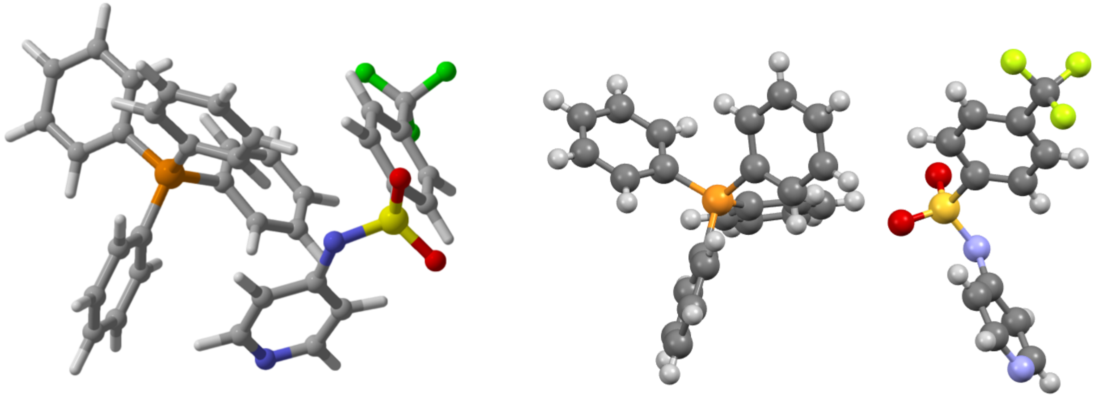

Figure S25. left: structure of conformer cat6a_012 calculated at SMD(DCM)/ B3LYP-D3/6-31+G(d) level of theory; right: crystal structure of one ion pair in catalyst **6a**.

11.2 Calculations of Methyl Cation Affinity (MCA) values

A way to describe the Lewis basicity of organocatalysts is the calculation of methyl cation affinity value (ΔMCA). Therefore, the difference in free reaction energy is determined of the methyl transfer from pyridine ma_py to the more Lewis basic ion pair **3a-d,4-6a** forming the methylated pyridinamide Me_**3a-d,4-6a** according to the equations shown in Figure S30-36.

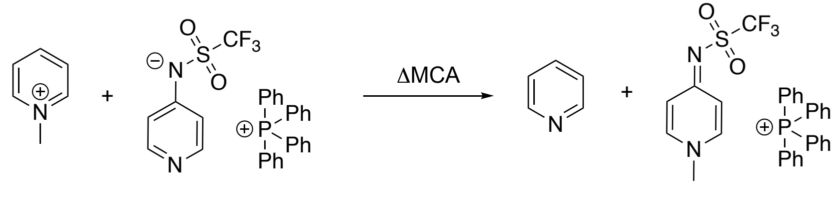

Figure S26. Equation used for calculation of relative Lewis basicity of ion pair **3a** with pyridine as reference.

Table S49. Energy for all systems shown in Figure S26.

System	E_{tot} SMD(DCM)/ B3LYP-D3/ 6-31+G(d)	H_{298} SMD(DCM)/ B3LYP-D3/ 6-31+G(d)	$G_{qh,298}$ SMD(DCM)/ B3LYP-D3/ 6-31+G(d)	$G_{qh,298,corr}$ SMD(DCM)/ B3LYP-D3/ 6-31+G(d)
**Pyridine**				
py_001_dcm_fr	-248.3117009	-248.2175679	-248.2502039	-248.2471854
ma_py_001_dcm_fr	-288.0755143	-287.9375633	-287.9743763	-287.9713578
**3a**				
cat3a_008	-2456.770628	**-2456.259420**	**-2456.354582**	**-2456.351563**
cat3a_002	-2456.770669	-2456.259399	-2456.354514	-2456.351496
cat3a_018	**-2456.770818**	-2456.259384	-2456.354496	-2456.351478
cat3a_029	-2456.770644	-2456.259350	-2456.354385	-2456.351367
cat3a_033m	-2456.770628	-2456.259315	-2456.354345	-2456.351327
		**-2456.2588145**	**-2456.353922**	**-2456.350904**
**Me_3a**				
ma_cat3a_003pp_dcm_ofr	-2496.547562	**-2495.992855**	**-2496.091673**	**-2496.088654**
ma_cat3a_033m_dcm	-2496.547742	-2495.992686	-2496.091213	-2496.088194
ma_cat3a_036ma_dcm_ofr	-2496.547841	-2495.992493	-2496.090755	-2496.087736
ma_cat3a_018_dcm	-2496.547747	-2495.992344	-2496.090674	-2496.087656

ma_cat3a_008_dcm	**-2496.548009**	-2495.992669	-2496.090602	-2496.087583
ma_cat3a_035m_dcm_ofr	-2496.547339	-2495.991834	-2496.090023	-2496.087005
		**-2495.9925766**	**-2496.0911053**	**-2496.0880868**
ΔMCA		-35.28		-33.92
**all**		**-36.14**		**-34.16**

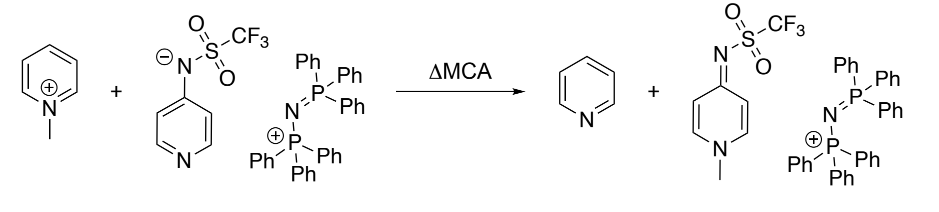

Figure S27. Equation used for calculation of relative Lewis basicity of ion pair **3b** with pyridine as reference.

Table S50. Energy for all systems shown in Figure S27.

**System**	**E_{tot} SMD(DCM)/ B3LYP-D3/ 6-31+G(d)**	**H_{298} SMD(DCM)/ B3LYP-D3/ 6-31+G(d)**	**$G_{qh,298}$ SMD(DCM)/ B3LYP-D3/ 6-31+G(d)**	**$G_{qh,298,corr}$ SMD(DCM)/ B3LYP-D3/ 6-31+G(d)**
**Pyridine**				
py_001_dcm_fr	-248.3117009	-248.2175679	-248.2502039	-248.2471854
ma_py_001_dcm_fr	-288.0755143	-287.9375633	-287.9743763	-287.9713578
**3b**				
cat3b_124_dcm_fr	**-3316.275809**	**-3315.561663**	**-3315.680224**	**-3315.677206**
cat3b_020_dcm_fr	-3316.275058	-3315.560851	-3315.679373	-3315.676355
cat3b_147_dcm_fr	-3316.274602	-3315.560287	-3315.678876	-3315.675857
cat3b_136m_dcm	-3316.274338	-3315.560086	-3315.678610	-3315.675591
cat3b_140_dcm_fr	-3316.274329	-3315.560084	-3315.678552	-3315.675533
		**-3315.559900**	**-3315.678519**	**-3315.675501**
**Me_3b**				
ma_cat3b_124_dcm	**-3356.0547111**	**-3355.2966571**	**-3355.4184931**	**-3355.4154746**

ma_cat3b_136mb_gas_dcm_ofr	-3356.0544600	-3355.2961920	-3355.4177030	-3355.4146845
ma_cat3b_027m_dcm_ofr	-3356.0531658	-3355.2950028	-3355.4165188	-3355.4135003
ma_cat3b_053_dcm_ofr	-3356.0541933	-3355.2956923	-3355.4164783	-3355.4134598
ma_cat3b_147m_dcm_ofr	-3356.0522264	-3355.2944324	-3355.4164324	-3355.4134139
ma_cat3b_147p_dcm_ofr	-3356.0522644	-3355.2940784	-3355.4160154	-3355.4129969
ma_cat3b_104b_gas_dcm_ofr	-3356.0523916	-3355.2937736	-3355.4150656	-3355.4120471
ma_cat3b_020_dcm_fr	-3356.0533789	-3355.2939939	-3355.4137099	-3355.4106914
		**-3355.2959969**	**-3355.4177759**	**-3355.4147575**
ΔMCA		-39.38		-37.01
**all**		**-42.27**		**-39.60**

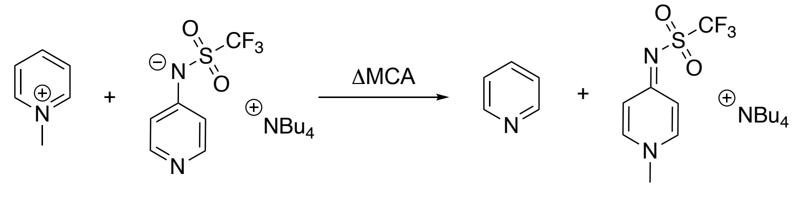

Figure S28. Equation used for calculation of relative Lewis basicity of ion pair **3c** with pyridine as reference.

Table S51. Energy for all systems shown in Figure S28.

**System**	**E_{tot} SMD(DCM)/ B3LYP-D3/ 6-31+G(d)**	**H_{298} SMD(DCM)/ B3LYP-D3/ 6-31+G(d)**	**$G_{qh,298}$ SMD(DCM)/ B3LYP-D3/ 6-31+G(d)**	**$G_{qh,298,corr}$ SMD(DCM)/ B3LYP-D3/ 6-31+G(d)**
**Pyridine**				
py_001_dcm_fr	-248.3117009	-248.2175679	-248.2502039	-248.2471854
ma_py_001_dcm_fr	-288.0755143	-287.9375633	-287.9743763	-287.9713578
**3c**				
cat3c_001_dcm	-1874.956418	**-1874.302434**	**-1874.401539**	**-1874.398520**
cat3c_034_dcm	-1874.956217	-1874.302034	-1874.401302	-1874.398284
cat3c_028_dcm	-1874.956268	-1874.302058	-1874.401246	-1874.398228
cat3c_003m_dcm_fr	-1874.956347	-1874.302312	-1874.401237	-1874.398219
cat3c_027_dcm	**-1874.957013**	-1874.302207	-1874.401158	-1874.398140

		**-1874.301555**	**-1874.400652**	**-1874.397634**
**Me_3c**				
ma_cat3c_007Ab_dcm_ofr	-1914.7331121	-1914.0350321	**-1914.1374141**	**-1914.1343956**
ma_cat3c_001m_dcm_ofr	-1914.7330948	**-1914.0350398**	-1914.1373738	-1914.1343553
ma_cat3c_003ma_gas_dcm_ofr	-1914.7332517	-1914.0347557	-1914.1367767	-1914.1337582
ma_cat3c_028f_gas_dcm_ofr	-1914.7330784	-1914.0347214	-1914.1365864	-1914.1335679
ma_cat3c_006p_dcm_ofr	**-1914.7333896**	-1914.0346546	-1914.1362436	-1914.1332251
ma_cat3c_027_dcm_fr	-1914.7317967	-1914.0332237	-1914.1350997	-1914.1320812
		**-1914.0348127**	**-1914.1370318**	**-1914.1340133**
ΔMCA		-33.11		-30.73
**all**		**-34.82**		**-32.05**

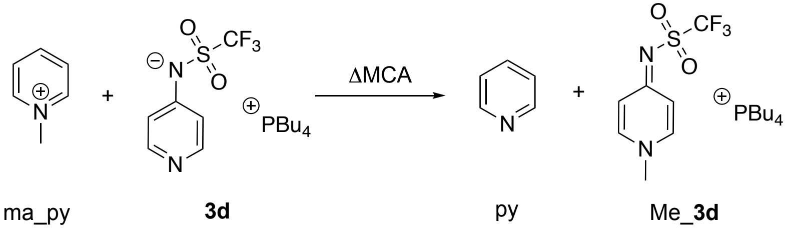

Figure S29. Equation used for calculation of relative Lewis basicity of ion pair **3d** with pyridine as reference.

Table S52. Energy for all systems shown in Figure S29.

System	E_{tot} SMD(DCM)/ B3LYP-D3/ 6-31+G(d)	H_{298} SMD(DCM)/ B3LYP-D3/ 6-31+G(d)	$G_{qh,298}$ SMD(DCM)/ B3LYP-D3/ 6-31+G(d)	$G_{qh,298,corr}$ SMD(DCM)/ B3LYP-D3/ 6-31+G(d)
**Pyridine**				
py_001_dcm_fr	-248.3117009	-248.2175679	-248.2502039	-248.2471854
ma_py_001_dcm_fr	-288.0755143	-287.9375633	-287.9743763	-287.9713578
**3d**				
cat3d_031_dcm_fr	-2161.591204	-2160.946455	**-2161.049248**	**-2161.046230**
cat3d_041a_dcm_fr	-2161.590889	-2160.946323	-2161.049170	-2161.046152
cat3d_027_dcm_fr	**-2161.591598**	**-2160.946633**	-2161.049140	-2161.046122

cat3d_029_dcm_fr	-2161.590751	-2160.946099	-2161.049124	-2161.046106
cat3d_001_dcm_fr	-2161.591275	-2160.946562	-2161.049074	-2161.046055
		**-2160.942902**	**-2161.048612**	**-2161.045594**
**Me_3d**				
ma_cat3d_034a_gas_dcm_ofr	-2201.3680129	**-2200.6794399**	**-2200.7851839**	**-2200.7821654**
ma_cat3d_013_dcm_fr	-2201.3679147	-2200.6790277	-2200.7848337	-2200.7818152
ma_cat3d_031_dcm	**-2201.3682858**	-2200.6791278	-2200.7843968	-2200.7813783
ma_cat3d_004p_dcm_ofr	-2201.3671572	-2200.6784452	-2200.7839092	-2200.7808907
ma_cat3d_057_dcm_fr	-2201.3676727	-2200.6783757	-2200.7838607	-2200.7808422
		**-2200.6790507**	**-2200.7847125**	**-2200.7816940**
ΔMCA		-33.64		-30.88
**all**		**-34.49**		**-31.32**

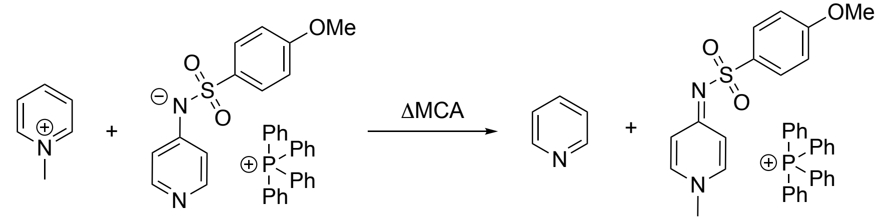

ma_py **4a** py Me_**4a**

Figure S30. Equation used for calculation of relative Lewis basicity of ion pair **4a** with pyridine as reference.

Table S53. Energy for all systems shown in Figure S30.

**System**	**E_{tot}<br>SMD(DCM)/<br>B3LYP-D3/<br>6-31+G(d)**	**H_{298}<br>SMD(DCM)/<br>B3LYP-D3/<br>6-31+G(d)**	**$G_{qh,298}$<br>SMD(DCM)/<br>B3LYP-D3/<br>6-31+G(d)**	**$G_{qh,298,corr}$<br>SMD(DCM)/<br>B3LYP-D3/<br>6-31+G(d)**
**Pyridine**				
py_001_dcm_fr	-248.3117009	-248.2175679	-248.2502039	-248.2471854
ma_py_001_dcm_fr	-288.0755143	-287.9375633	-287.9743763	-287.9713578
**4a**				

cat4a_050_dcm	**-2465.334938**	**-2464.710187**	**-2464.811281**	**-2464.808262**
cat4a_030_dcm	-2465.334896	-2464.710055	-2464.811095	-2464.808077
cat4a_026_dcm	-2465.333194	-2464.708774	-2464.810286	-2464.807268
cat4a_108_dcm	-2465.333336	-2464.708800	-2464.810174	-2464.807155
cat4a_105_dcm	-2465.332334	-2464.708102	-2464.809786	-2464.806767
		**-2464.708811**	**-2464.809896**	**-2464.806877**
**Me_4a**				
ma_cat4a_025b_gas_dcm_ofr	-2505.1179139	**-2504.4496129**	**-2504.5548899**	**-2504.5518714**
ma_cat4a_108p_dcm_ofr	-2505.1176995	-2504.4495725	-2504.5548515	-2504.5518330
ma_cat4a_030_dcm_fr	-2505.1183124	-2504.4491904	-2504.5529494	-2504.5499309
ma_cat4a_026a_gas_dcm_ofr	-2505.1168195	-2504.4477005	-2504.5521365	-2504.5491180
ma_cat4a_037_dcm_ofr	-2505.1177474	-2504.4484204	-2504.5519874	-2504.5489689
ma_cat4a_050_dcm	**-2505.1188272**	-2504.4484902	-2504.5501182	-2504.5470997
		**-2504.4492390**	**-2504.5546169**	**-2504.5515985**
ΔMCA		-51.02		-51.03
**all**		**-53.64**		**-53.95**

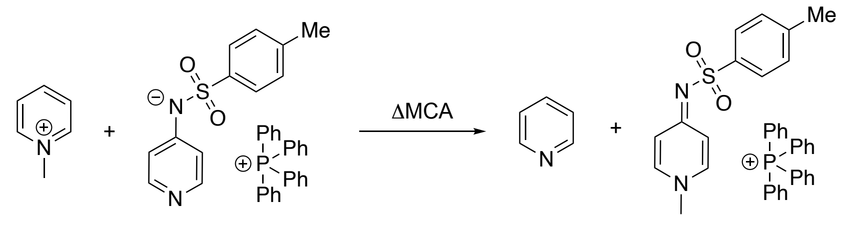

Figure S31. Equation used for calculation of relative Lewis basicity of ion pair **5a** with pyridine as reference.

Table S54. Energy for all systems shown in Figure S31.

System	E_{tot} SMD(DCM)/ B3LYP-D3/ 6-31+G(d)	H_{298} SMD(DCM)/ B3LYP-D3/ 6-31+G(d)	$G_{qh,298}$ SMD(DCM)/ B3LYP-D3/ 6-31+G(d)	$G_{qh,298,corr}$ SMD(DCM)/ B3LYP-D3/ 6-31+G(d)
**Pyridine**				
py_001_dcm_fr	-248.3117009	-248.2175679	-248.2502039	-248.2471854

ma_py_001_dcm_fr	-288.0755143	-287.9375633	-287.9743763	-287.9713578
**5a**				
cat5a_012_dcm_fr	**-2390.123118**	**-2389.504272**	**-2389.604120**	**-2389.601102**
cat5a_078m_dcm_fr	-2390.122167	-2389.503803	-2389.603890	-2389.600871
cat5a_041_dcm_fr	-2390.121537	-2389.503322	-2389.603789	-2389.600771
cat5a_032_dcm_fr	-2390.121516	-2389.503282	-2389.603532	-2389.600514
cat5a_028_dcm_fr	-2390.121504	-2389.503143	-2389.603493	-2389.600474
		**-2389.503006**	**-2389.603068**	**-2389.600049**
**Me_5a**				
ma_cat5a_032_dcm_ofr	-2429.9057838	-2429.2438118	**-2429.3477648**	**-2429.3447463**
ma_cat5a_012_dcm	**-2429.9064585**	**-2429.2438815**	-2429.3471655	-2429.3441470
ma_cat5a_041p_dcm_ofr	-2429.9060414	-2429.2438664	-2429.3471074	-2429.3440889
ma_cat5a_078m_dcm_fr	-2429.9047560	-2429.2425950	-2429.3461140	-2429.3430955
ma_cat5a_063b_gas_dcm_ofr	-2429.9040312	-2429.2417902	-2429.3456412	-2429.3426227
ma_cat5a_013_dcm_ofr	-2429.9052240	-2429.2425910	-2429.3455130	-2429.3424945
		**-2429.2436080**	**-2429.3471965**	**-2429.3441780**
ΔMCA		-51.50		-51.12
**all**		**-54.10**		**-52.40**

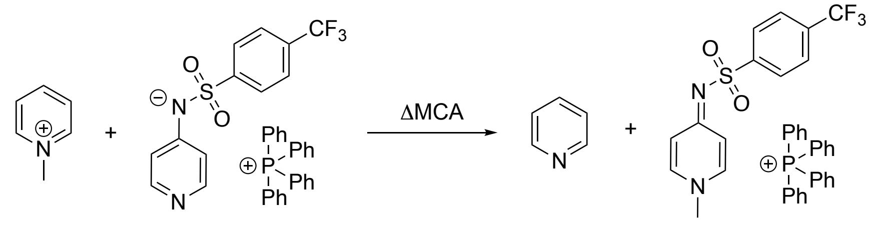

Figure S32. Equation used for calculation of relative Lewis basicity of ion pair **6a** with pyridine as reference.

Table S55. Energy for all systems shown in Figure S32.

System	E_{tot} SMD(DCM)/	H_{298} SMD(DCM)/	$G_{qh,298}$ SMD(DCM)/	$G_{qh,298,corr}$ SMD(DCM)/

	B3LYP-D3/<br>6-31+G(d)	B3LYP-D3/<br>6-31+G(d)	B3LYP-D3/<br>6-31+G(d)	B3LYP-D3/<br>6-31+G(d)
**Pyridine**				
py_001_dcm_fr	-248.3117009	-248.2175679	-248.2502039	-248.2471854
ma_py_001_dcm_fr	-288.0755143	-287.9375633	-287.9743763	-287.9713578
**6a**				
cat6a_012_dcm	-2687.866218	**-2687.269030**	**-2687.372424**	**-2687.369405**
cat6a_062_dcm	-2687.865545	-2687.268462	-2687.371852	-2687.368833
cat6a_077_dcm	-2687.865538	-2687.268342	-2687.371699	-2687.368681
cat6a_049_dcm	-2687.864657	-2687.267827	-2687.371664	-2687.368645
cat6a_003_dcm	**-2687.866341**	-2687.268788	-2687.371615	-2687.368597
		**-2687.268042**	**-2687.371356**	**-2687.368337**
**Me_6a**				
ma_cat6a_032_dcm_ofr	-2727.6473085	**-2727.0070385**	**-2727.1146215**	**-2727.1116030**
ma_cat6a_040_dcm_ofr	-2727.6472324	-2727.0066464	-2727.1141994	-2727.1111809
ma_cat6a_049_dcm_ofr	-2727.6473434	-2727.0066284	-2727.1139164	-2727.1108979
ma_cat6a_003m_dcm_ofr	**-2727.6479245**	-2727.0060155	-2727.1109755	-2727.1079570
		**-2727.0067095**	**-2727.1143026**	**-2727.1112841**
ΔMCA		-47.29		-47.33
**all**		**-49.02**		**-49.29**

Previous studies of ΔMCA indicate that the catalytic active center is situated on the pyridine N rather than the amide N. Since anion **6** is a newly designed compound ΔMCA were calculated for the pyridine N methylated and the amide N methylated conformer according to the equation shown in Figure S33. The energy values are listed in Table S56.

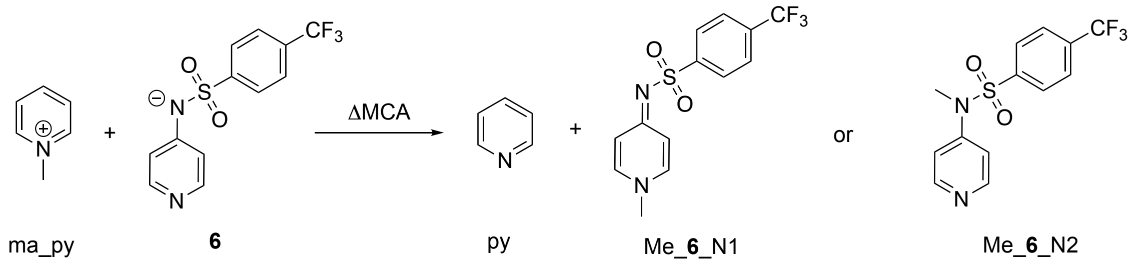

Figure S33. Comparison of MCA at pyridine-N position (N1) and amide-N position (N2) for anion **6** with pyridine as reference system.

The resulting ΔMCA show that the methylation on the pyridine N leads to the thermodynamic more stable product by 13.7 kJ mol^{-1} in reaction enthalpy and by 16.6 kJ mol^{-1} in the free reaction energy.

Table S56. Energy for all systems shown in Figure S33.

**System**	**E_{tot} SMD(DCM)/ B3LYP-D3/ 6-31+G(d)**	**H_{298} SMD(DCM)/ B3LYP-D3/ 6-31+G(d)**	**$G_{qh,298}$ SMD(DCM)/ B3LYP-D3/ 6-31+G(d)**	**$G_{qh,298,corr}$ SMD(DCM)/ B3LYP-D3/ 6-31+G(d)**
**Pyridine**				
py_001_dcm_fr	-248.3117009	-248.2175679	-248.2502039	-248.2471854
ma_py_001_dcm_fr	-288.0755143	-287.9375633	-287.9743763	-287.9713578
**6**				
an6_001	**-1419.929810**	**-1419.724473**	**-1419.784896**	**-1419.781877**
**Me_6**				
ma_an6_001_n1_dcm_fr	-1459.7173223	-1459.4683223	-1459.5325203	-1459.5295018
ma_an6_001_n2_dcm_fr	-1459.7118231	-1459.4630761	-1459.5262031	-1459.5231846
**ΔMCA**				
ma_an6_n1[a]		-62.6		-61.6
ma_an6_n2[b]		-48.9		-45.0

[a] N1 = methylation on pyridine N, [b] N2 = methylation on amide N.

11.3 Conformers of Pyridinamide Triple Ion Complexes in DCM

Starting points of pyridinamide ion triple complexes were pre-optimized using SMD(DCM)/r^{2}SCAN-3c level of theory with Orca 5.0.3[43], followed by single point calculations at SMD(DCM)/B3LYP-D3/6-31+G(d) with Gaussian 16, Revision C.01[44] to obtain calculated triple ion volumes based on the van der Waals cavities employed in the SMD continuum solvation model employed in Gaussian 16 so they are comparable to the calculated ion volumes of ion pair systems and the respective single cation and anions optimized at the SMD(DCM)/B3LYP-D3/6-31+G(d) with Gaussian 16.

Table S57. Single point energies for all triple ion systems of ion pair **3a** calculated at the SMD(DCM)/B3LYP-D3/6-31+G(d) level of theory with Gaussian 16.

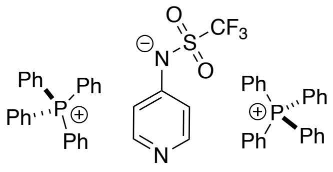

a3a

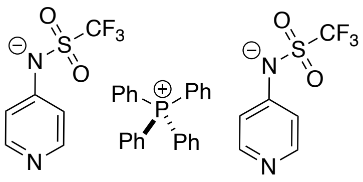

3a3

System[a]	E_{tot} SMD(DCM)/ B3LYP-D3/6-31+G(d)	Cavity Volume (Å^3)	Relative Population Parameter based on E_{298}	System[a]	E_{tot} SMD(DCM)/ B3LYP-D3/6-31+G(d)	Cavity Volume (Å^3)	Relative Population Parameter based on E_{298}
sw_a3a_new_033b_dcm_sp	**-3724.7011753**	**925**	**0.39**	asw_3a3_058b_dcm_sp	**-3645.6244701**	**781**	**0.23**
sw_a3a_new_030a_dcm_sp	-3724.7008822	923	0.28	asw_3a3_011a_dcm_sp	-3645.6233739	780	0.07
sw_a3a_new_005na_dcm_sp	-3724.6995552	925	0.07	asw_3a3_044a_dcm_sp	-3645.6233403	781	0.07
sw_a3a_new_039a_dcm_sp	-3724.6993743	926	0.06	asw_3a3_032a_dcm_sp	-3645.6231043	783	0.05
sw_a3a_new_015b_dcm_sp	-3724.6989764	926	0.04	asw_3a3_053a_dcm_sp	-3645.6231020	783	0.05
sw_a3a_new_042a_dcm_sp	-3724.6989336	927	0.04	asw_3a3_006_dcm_sp	-3645.6230698	781	0.05
sw_a3a_new_010b_dcm_sp	-3724.6985369	927	0.02	asw_3a3_047a_dcm_sp	-3645.6230374	782	0.05
sw_a3a_new_045c_dcm_sp	-3724.6984005	926	0.02	asw_3a3_039b_dcm_sp	-3645.6229589	782	0.05
sw_a3a_new_014_dcm_sp	-3724.6980441	926	0.01	asw_3a3_045b_dcm_sp	-3645.6228896	780	0.04
sw_a3a_new_047a_dcm_sp	-3724.6976338	926	0.01	asw_3a3_049a_dcm_sp	-3645.6228146	783	0.04
sw_a3a_new_018a_dcm_sp	-3724.6975821	926	0.01	asw_3a3_048b_dcm_sp	-3645.6227627	784	0.04
sw_a3a_new_038a_dcm_sp	-3724.6975740	927	0.01	asw_3a3_057b_dcm_sp	-3645.6226849	781	0.03
sw_a3a_new_020a_dcm_sp	-3724.6971852	926	0.01	asw_3a3_050_dcm_sp	-3645.6223597	781	0.02
sw_a3a_new_004a_dcm_sp	-3724.6970783	926	0.01	asw_3a3_059b_dcm_sp	-3645.6223396	782	0.02
sw_a3a_new_003_dcm_sp	-3724.6968941	928	0.00	asw_3a3_022a_dcm_sp	-3645.6220941	781	0.02
sw_a3a_new_052b_dcm_sp	-3724.6967130	925	0.00	asw_3a3_037_dcm_sp	-3645.6219701	785	0.02
sw_a3a_new_013b_dcm_sp	-3724.6964818	925	0.00	asw_3a3_008a_dcm_sp	-3645.6217232	783	0.01

sw_a3a_new_029a_dcm_sp	-3724.6964377	925	0.00	asw_3a3_051b_dcm_sp	-3645.6217229	785	0.01
sw_a3a_new_040a_dcm_sp	-3724.6963431	924	0.00	asw_3a3_033_dcm_sp	-3645.6216873	780	0.01
sw_a3a_new_055b_dcm_sp	-3724.6963343	928	0.00	asw_3a3_004a_dcm_sp	-3645.6216535	780	0.01
	**-3724.7002363**	**925**			**-3645.6230836**	**782**	

[a] best 20 conformers according to E_{tot} at SMD(DCM)/B3LYP-D3/6-31+G(d) level of theory.

Table S58. Single point energies for all triple ion systems of ion pair **3b** calculated at the SMD(DCM)/B3LYP-D3/6-31+G(d) level of theory with Gaussian 16.

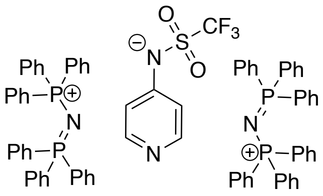 **b3b**				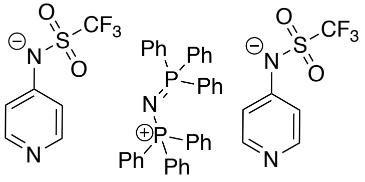 **3b3**			
**System[a]**	**E_{tot} SMD(DCM)/ B3LYP-D3/6-31+G(d)**	**Cavity Volume (Å^3)**	**Relative Population Parameter based on E_{298}**	**System[a]**	**E_{tot} SMD(DCM)/ B3LYP-D3/6-31+G(d)**	**Cavity Volume (Å^3)**	**Relative Population Parameter based on E_{298}**
sw_b3b_013a_dcm_sp	**-5443.7140126**	**1320**	**0.61**	asw_3b3_005_dcm_sp	**-4505.1275635**	**981**	**0.24**
sw_b3b_017a_dcm_sp	-5443.7127269	1321	0.16	asw_3b3_011_dcm_sp	-4505.1272391	982	0.17
sw_b3b_006a_dcm_sp	-5443.7119224	1323	0.07	asw_3b3_052_dcm_sp	-4505.1266092	978	0.09
sw_b3b_020_dcm_sp	-5443.7114798	1320	0.04	asw_3b3_059_dcm_sp	-4505.1263098	983	0.06
sw_b3b_009a_dcm_sp	-5443.7112347	1323	0.03	asw_3b3_015_dcm_sp	-4505.1262657	980	0.06
sw_b3b_032a_dcm_sp	-5443.7109708	1326	0.02	asw_3b3_021_dcm_sp	-4505.1262106	979	0.06
sw_b3b_035_dcm_sp	-5443.7109331	1320	0.02	asw_3b3_040_dcm_sp	-4505.1259597	978	0.04
sw_b3b_018_dcm_sp	-5443.7108530	1325	0.02	asw_3b3_006_dcm_sp	-4505.1258741	979	0.04
sw_b3b_007a_dcm_sp	-5443.7099513	1322	0.01	asw_3b3_017_dcm_sp	-4505.1257747	982	0.04
sw_b3b_034_dcm_sp	-5443.7099490	1322	0.01	asw_3b3_060_dcm_sp	-4505.1257439	977	0.04
sw_b3b_044_dcm_sp	-5443.7095511	1324	0.01	asw_3b3_019n_dcm_sp	-4505.1257036	977	0.03
sw_b3b_054a_dcm_sp	-5443.7085566	1325	0.00	asw_3b3_012_dcm_sp	-4505.1252592	985	0.02
sw_b3b_058a_dcm_sp	-5443.7077099	1323	0.00	asw_3b3_035_dcm_sp	-4505.1249856	980	0.02
sw_b3b_015a_dcm_sp	-5443.7077063	1322	0.00	asw_3b3_049_dcm_sp	-4505.1244954	983	0.01
sw_b3b_012a_dcm_sp	-5443.7076373	1324	0.00	asw_3b3_018_dcm_sp	-4505.1244067	980	0.01
sw_b3b_055a_dcm_sp	-5443.7070373	1322	0.00	asw_3b3_008_dcm_sp	-4505.1241883	980	0.01

sw_b3b_025_dcm_sp	-5443.7067425	1322	0.00	asw_3b3_043_dcm_sp	-4505.1241157	984	0.01
sw_b3b_008a_dcm_sp	-5443.7061014	1320	0.00	asw_3b3_038_dcm_sp	-4505.1240810	981	0.01
sw_b3b_004n_dcm_sp	-5443.7060467	1325	0.00	asw_3b3_036_dcm_sp	-4505.1240283	980	0.01
sw_b3b_014a_dcm_sp	-5443.7059537	1325	0.00	asw_3b3_025_dcm_sp	-4505.1240232	980	0.01
	**-5443.7131383**	**1321**			**-4505.1264153**	**981**	

[a] best 20 conformers according to E_{tot} at SMD(DCM)/B3LYP-D3/6-31+G(d) level of theory.

Table S59. Single point energies for all triple ion systems of ion pair **3c** calculated at the SMD(DCM)/B3LYP-D3/6-31+G(d) level of theory with Gaussian 16.

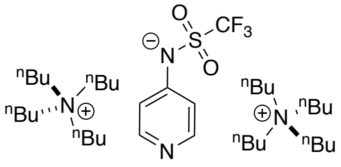

c3c

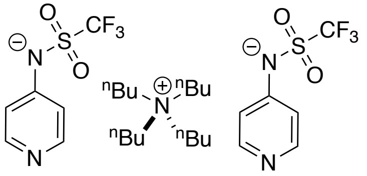

3c3

System[a]	E_{tot} SMD(DCM)/ B3LYP-D3/6-31+G(d)	Cavity Volume (Å^3)	Relative Population Parameter based on E_{298}	System[a]	E_{tot} SMD(DCM)/ B3LYP-D3/6-31+G(d)	Cavity Volume (Å^3)	Relative Population Parameter based on E_{298}
sw_c3c_052_dcm_sp	**-2561.0719269**	**830**	**0.38**	asw_3c3_007_dcm_sp	**-3063.8103134**	**733**	**0.12**
sw_c3c_028_dcm_sp	-2561.0717762	830	0.33	asw_3c3_060a_dcm_sp	-3063.8102955	733	0.12
sw_c3c_038_dcm_sp	-2561.0705711	830	0.09	asw_3c3_057_dcm_sp	-3063.8102873	733	0.12
sw_c3c_018_dcm_sp	-2561.0705085	830	0.09	asw_3c3_046_dcm_sp	-3063.8101185	735	0.10
sw_c3c_034_dcm_sp	-2561.0692660	830	0.02	asw_3c3_059_dcm_sp	-3063.8101059	732	0.10
sw_c3c_048_dcm_sp	-2561.0692481	827	0.02	asw_3c3_030_dcm_sp	-3063.8099630	733	0.09
sw_c3c_001_dcm_sp	-2561.0692174	832	0.02	asw_3c3_005_dcm_sp	-3063.8094223	734	0.05
sw_c3c_050_dcm_sp	-2561.0682378	829	0.01	asw_3c3_047_dcm_sp	-3063.8092563	734	0.04
sw_c3c_017_dcm_sp	-2561.0680673	828	0.01	asw_3c3_027_dcm_sp	-3063.8090680	733	0.03
sw_c3c_051_dcm_sp	-2561.0680099	829	0.01	asw_3c3_006_dcm_sp	-3063.8089166	732	0.03
sw_c3c_057_dcm_sp	-2561.0678695	831	0.01	asw_3c3_043a_dcm_sp	-3063.8086977	733	0.02
sw_c3c_022_dcm_sp	-2561.0676726	832	0.00	asw_3c3_045a_dcm_sp	-3063.8086730	736	0.02
sw_c3c_011_dcm_sp	-2561.0676605	830	0.00	asw_3c3_058_dcm_sp	-3063.8086506	735	0.02
sw_c3c_012_dcm_sp	-2561.0676197	829	0.00	asw_3c3_051a_dcm_sp	-3063.8086414	734	0.02
sw_c3c_037_dcm_sp	-2561.0671134	830	0.00	asw_3c3_038a_dcm_sp	-3063.8083860	732	0.02

sw_c3c_005n_dcm_sp	-2561.0661687	833	0.00	asw_3c3_012_dcm_sp	-3063.8083259	734	0.02
sw_c3c_039_dcm_sp	-2561.0659415	830	0.00	asw_3c3_018_dcm_sp	-3063.8082924	734	0.01
sw_c3c_004_dcm_sp	-2561.0657957	833	0.00	asw_3c3_010b_dcm_sp	-3063.8081661	732	0.01
sw_c3c_054_dcm_sp	-2561.0655312	831	0.00	asw_3c3_024_dcm_sp	-3063.8078301	737	0.01
sw_c3c_059_dcm_sp	-2561.0639387	828	0.00	asw_3c3_053_dcm_sp	-3063.8077583	733	0.01
	**-2561.0712698**	**830**			**-3063.8096410**	**733**	

[a] best 20 conformers according to E_{tot} at SMD(DCM)/B3LYP-D3/6-31+G(d) level of theory.

Table S60. Single point energies for all triple ion systems of ion pair **3d** calculated at the SMD(DCM)/B3LYP-D3/6-31+G(d) level of theory with Gaussian 16.

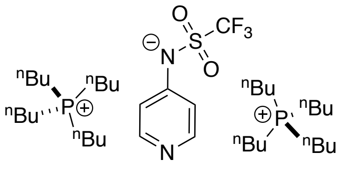 **d3d**				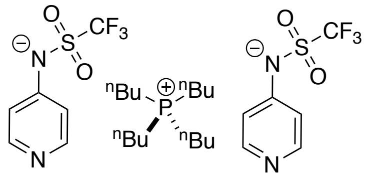 **3d3**			
**System[a]**	**E_{tot} SMD(DCM)/ B3LYP-D3/6-31+G(d)**	**Cavity Volume (Å^3)**	**Relative Population Parameter based on E_{298}**	**System[a]**	**E_{tot} SMD(DCM)/ B3LYP-D3/6-31+G(d)**	**Cavity Volume (Å^3)**	**Relative Population Parameter based on E_{298}**
sw_d3d_025a_dcm_sp	**-3134.3408192**	**860**	**0.18**	asw_3d3_012_dcm_sp	**-3350.4463795**	**749**	**0.24**
sw_d3d_006_dcm_sp	-3134.3407523	859	0.17	asw_3d3_026na_dcm_sp	-3350.4457333	748	0.12
sw_d3d_029_dcm_sp	-3134.3406891	860	0.16	asw_3d3_039a_dcm_sp	-3350.4455166	748	0.09
sw_d3d_005_dcm_sp	-3134.3405380	857	0.13	asw_3d3_057_dcm_sp	-3350.4454886	748	0.09
sw_d3d_028a_dcm_sp	-3134.3402854	861	0.10	asw_3d3_050_dcm_sp	-3350.4450766	747	0.06
sw_d3d_001_dcm_sp	-3134.3398077	861	0.06	asw_3d3_021_dcm_sp	-3350.4447109	750	0.04
sw_d3d_047a_dcm_sp	-3134.3396429	857	0.05	asw_3d3_022_dcm_sp	-3350.4446535	747	0.04
sw_d3d_011_dcm_sp	-3134.3392396	857	0.03	asw_3d3_020a_dcm_sp	-3350.4445641	748	0.03
sw_d3d_048b_dcm_sp	-3134.3389328	857	0.02	asw_3d3_052_dcm_sp	-3350.4445564	747	0.03
sw_d3d_018_dcm_sp	-3134.3382911	859	0.01	asw_3d3_030a_dcm_sp	-3350.4445371	749	0.03
sw_d3d_019_dcm_sp	-3134.3382483	859	0.01	asw_3d3_036_dcm_sp	-3350.4444546	750	0.03
sw_d3d_054b_dcm_sp	-3134.3381786	861	0.01	asw_3d3_004_dcm_sp	-3350.4441409	747	0.02
sw_d3d_060_dcm_sp	-3134.3379861	860	0.01	asw_3d3_031_dcm_sp	-3350.4438600	750	0.02
sw_d3d_041a_dcm_sp	-3134.3379353	860	0.01	asw_3d3_006_dcm_sp	-3350.4437393	746	0.01

sw_d3d_003a_dcm_sp	-3134.3378059	860	0.01	asw_3d3_003_dcm_sp	-3350.4437379	750	0.01
sw_d3d_044n_dcm_sp	-3134.3371015	858	0.00	asw_3d3_015_dcm_sp	-3350.4436902	749	0.01
sw_d3d_039a_dcm_sp	-3134.3370776	859	0.00	asw_3d3_002a_dcm_sp	-3350.4436816	747	0.01
sw_d3d_017a_dcm_sp	-3134.3369477	858	0.00	asw_3d3_011_dcm_sp	-3350.4436606	750	0.01
sw_d3d_056_dcm_sp	-3134.3369464	859	0.00	asw_3d3_017_dcm_sp	-3350.4435234	745	0.01
sw_d3d_004_dcm_sp	-3134.3367814	857	0.00	asw_3d3_019a_dcm_sp	-3350.4431721	748	0.01
	**-3134.3402174**	**859**			**-3350.4451207**	**748**	

[a] best 20 conformers according to E_{tot} at SMD(DCM)/B3LYP-D3/6-31+G(d) level of theory.

Table S61. Single point energies for all triple ion systems of ion pair **4a** calculated at the SMD(DCM)/B3LYP-D3/6-31+G(d) level of theory with Gaussian 16.

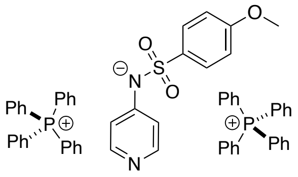

a4a

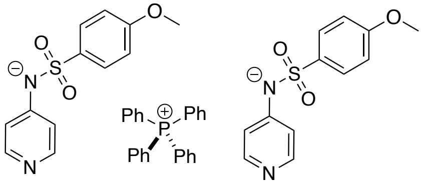

4a4

**System[a]**	**E_{tot} SMD(DCM)/ B3LYP-D3/6-31+G(d)**	**Cavity Volume (Å^3)**	**Relative Population Parameter based on E_{298}**	**System[a]**	**E_{tot} SMD(DCM)/ B3LYP-D3/6-31+G(d)**	**Cavity Volume (Å^3)**	**Relative Population Parameter based on E_{298}**
sw_a4a_018_dcm_sp	**-3733.2653541**	**993**	**0.39**	asw_4a4_005a_dcm_sp	**-3662.7548378**	**921**	**0.53**
sw_a4a_049a_dcm_sp	-3733.2653444	995	0.39	asw_4a4_016_dcm_sp	-3662.7535339	921	0.13
sw_a4a_004b_dcm_sp	-3733.2635672	998	0.06	asw_4a4_011a_dcm_sp	-3662.7534663	920	0.12
sw_a4a_040b_dcm_sp	-3733.2629560	999	0.03	asw_4a4_015a_dcm_sp	-3662.7532492	920	0.10
sw_a4a_022_dcm_sp	-3733.2629292	997	0.03	asw_4a4_006_dcm_sp	-3662.7526111	915	0.05
sw_a4a_017a_dcm_sp	-3733.2628530	997	0.03	asw_4a4_031a_dcm_sp	-3662.7516599	923	0.02
sw_a4a_005a_dcm_sp	-3733.2626049	998	0.02	asw_4a4_024a_dcm_sp	-3662.7515888	923	0.02
sw_a4a_011a_dcm_sp	-3733.2619594	998	0.01	asw_4a4_059n_dcm_sp	-3662.7514401	922	0.01
sw_a4a_008a_dcm_sp	-3733.2613398	998	0.01	asw_4a4_001_dcm_sp	-3662.7495052	924	0.00
sw_a4a_028a_dcm_sp	-3733.2612325	995	0.01	asw_4a4_034_dcm_sp	-3662.7493973	923	0.00
sw_a4a_007_dcm_sp	-3733.2611335	999	0.00	asw_4a4_021a_dcm_sp	-3662.7488765	923	0.00
sw_a4a_039a_dcm_sp	-3733.2608890	999	0.00	asw_4a4_002_dcm_sp	-3662.7486765	922	0.00
sw_a4a_016_dcm_sp	-3733.2606781	998	0.00	asw_4a4_014_dcm_sp	-3662.7485596	922	0.00

sw_a4a_047a_dcm_sp	-3733.2603097	994	0.00	asw_4a4_023a_dcm_sp	-3662.7485027	920	0.00
sw_a4a_038b_dcm_sp	-3733.2602851	997	0.00	asw_4a4_042b_dcm_sp	-3662.7484625	918	0.00
sw_a4a_044_dcm_sp	-3733.2601809	994	0.00	asw_4a4_035a_dcm_sp	-3662.7480696	923	0.00
sw_a4a_037a_dcm_sp	-3733.2601341	995	0.00	asw_4a4_050a_dcm_sp	-3662.7480102	915	0.00
sw_a4a_013a_dcm_sp	-3733.2597333	998	0.00	asw_4a4_057n_dcm_sp	-3662.7479483	923	0.00
sw_a4a_023b_dcm_sp	-3733.2595989	996	0.00	asw_4a4_019b_dcm_sp	-3662.7477695	921	0.00
sw_a4a_015a_dcm_sp	-3733.2593335	998	0.00	asw_4a4_028b_dcm_sp	-3662.7477203	922	0.00
	**-3733.2647588**	**995**			**-3662.7539922**	**920**	

[a] best 20 conformers according to E_{tot} at SMD(DCM)/B3LYP-D3/6-31+G(d) level of theory.

Table S62. Single point energies for all triple ion systems of ion pair **5a** calculated at the SMD(DCM)/B3LYP-D3/6-31+G(d) level of theory with Gaussian 16.

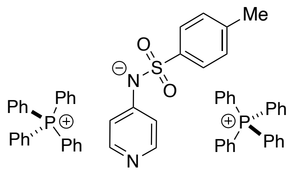

a5a

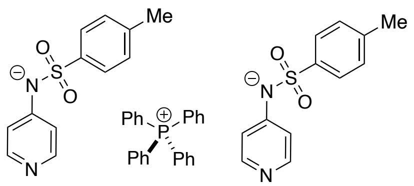

5a5

**System[a]**	**E_{tot} SMD(DCM)/ B3LYP-D3/6-31+G(d)**	**Cavity Volume (Å^3)**	**Relative Population Parameter based on E_{298}**	**System[a]**	**E_{tot} SMD(DCM)/ B3LYP-D3/6-31+G(d)**	**Cavity Volume (Å^3)**	**Relative Population Parameter based on E_{298}**
sw_a5a_002_dcm_sp	**-3658.0547888**	**979**	**0.79**	asw_5a5_002_dcm_sp	**-3512.3318260**	**886**	**0.64**
sw_a5a_042_dcm_sp	-3658.0527891	981	0.10	asw_5a5_047_dcm_sp	-3512.3306211	889	0.18
sw_a5a_039_dcm_sp	-3658.0515452	981	0.03	asw_5a5_040_dcm_sp	-3512.3299469	885	0.09
sw_a5a_057_dcm_sp	-3658.0509115	979	0.01	asw_5a5_003_dcm_sp	-3512.3289716	887	0.03
sw_a5a_007a_dcm_sp	-3658.0508619	983	0.01	asw_5a5_043_dcm_sp	-3512.3282751	889	0.01
sw_a5a_014_dcm_sp	-3658.0507259	976	0.01	asw_5a5_049_dcm_sp	-3512.3280050	884	0.01
sw_a5a_016_dcm_sp	-3658.0506566	975	0.01	asw_5a5_017_dcm_sp	-3512.3276961	885	0.01
sw_a5a_055_dcm_sp	-3658.0504648	978	0.01	asw_5a5_028_dcm_sp	-3512.3275307	890	0.01
sw_a5a_026_dcm_sp	-3658.0501218	979	0.01	asw_5a5_045_dcm_sp	-3512.3274238	888	0.01
sw_a5a_037_dcm_sp	-3658.0498951	978	0.00	asw_5a5_039_dcm_sp	-3512.3273996	880	0.01
sw_a5a_019a_dcm_sp	-3658.0497402	976	0.00	asw_5a5_038_dcm_sp	-3512.3268988	884	0.00
sw_a5a_033_dcm_sp	-3658.0497270	981	0.00	asw_5a5_011_dcm_sp	-3512.3265133	888	0.00

sw_a5a_041_dcm_sp	-3658.0490194	984	0.00	asw_5a5_033_dcm_sp	-3512.3264806	883	0.00
sw_a5a_005_dcm_sp	-3658.0489046	982	0.00	asw_5a5_058_dcm_sp	-3512.3262658	890	0.00
sw_a5a_038_dcm_sp	-3658.0488603	979	0.00	asw_5a5_013_dcm_sp	-3512.3262106	882	0.00
sw_a5a_045n_dcm_sp	-3658.0484830	982	0.00	asw_5a5_027_dcm_sp	-3512.3261552	889	0.00
sw_a5a_011_dcm_sp	-3658.0484258	980	0.00	asw_5a5_018_dcm_sp	-3512.3258251	886	0.00
sw_a5a_036a_dcm_sp	-3658.0483335	979	0.00	asw_5a5_053_dcm_sp	-3512.3255224	889	0.00
sw_a5a_010_dcm_sp	-3658.0481000	982	0.00	asw_5a5_051_dcm_sp	-3512.3250416	886	0.00
sw_a5a_006_dcm_sp	-3658.0479113	977	0.00	asw_5a5_015_dcm_sp	-3512.3246455	884	0.00
	**-3658.0541301**	**980**			**-3512.3310521**	**887**	

[a] best 20 conformers according to E_{tot} at SMD(DCM)/B3LYP-D3/6-31+G(d) level of theory.

Table S63. Single point energies for all triple ion systems of ion pair **6a** calculated at the SMD(DCM)/B3LYP-D3/6-31+G(d) level of theory with Gaussian 16.

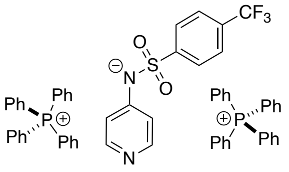 **a6a**				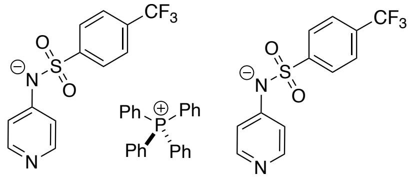 **6a6**			
**System[a]**	**E_{tot} SMD(DCM)/ B3LYP-D3/6-31+G(d)**	**Cavity Volume (Å^3)**	**Relative Population Parameter based on E_{298}**	**System[a]**	**E_{tot} SMD(DCM)/ B3LYP-D3/6-31+G(d)**	**Cavity Volume (Å^3)**	**Relative Population Parameter based on E_{298}**
sw_a6a_016a_dcm_sp	**-3955.7953706**	**1008**	**0.36**	asw_6a6_040a_dcm_sp	**-4107.8137166**	**942**	**0.39**
sw_a6a_032n_dcm_sp	-3955.7945098	1006	0.15	asw_6a6_022_dcm_sp	-4107.8123392	943	0.09
sw_a6a_057a_dcm_sp	-3955.7944884	1007	0.14	asw_6a6_050a_dcm_sp	-4107.8123121	939	0.09
sw_a6a_019a_dcm_sp	-3955.7941140	1008	0.10	asw_6a6_021b_dcm_sp	-4107.8120945	942	0.07
sw_a6a_028a_dcm_sp	-3955.7932440	1004	0.04	asw_6a6_012_dcm_sp	-4107.8120813	941	0.07
sw_a6a_007b_dcm_sp	-3955.7932155	1008	0.04	asw_6a6_017a_dcm_sp	-4107.8118183	942	0.05
sw_a6a_043a_dcm_sp	-3955.7931982	1006	0.04	asw_6a6_052_dcm_sp	-4107.8113834	942	0.03
sw_a6a_044b_dcm_sp	-3955.7928419	1002	0.02	asw_6a6_035_dcm_sp	-4107.8112172	942	0.03
sw_a6a_033b_dcm_sp	-3955.7925148	1006	0.02	asw_6a6_031a_dcm_sp	-4107.8112095	940	0.03
sw_a6a_046a_dcm_sp	-3955.7921923	1003	0.01	asw_6a6_018_dcm_sp	-4107.8109963	940	0.02
sw_a6a_009a_dcm_sp	-3955.7921595	1005	0.01	asw_6a6_027b_dcm_sp	-4107.8109782	941	0.02

sw_a6a_018a_dcm_sp	-3955.7920784	1006	0.01	asw_6a6_026b_dcm_sp	-4107.8107390	941	0.02
sw_a6a_054a_dcm_sp	-3955.7918627	1008	0.01	asw_6a6_038_dcm_sp	-4107.8106768	943	0.02
sw_a6a_050a_dcm_sp	-3955.7918518	1006	0.01	asw_6a6_043a_dcm_sp	-4107.8106361	940	0.01
sw_a6a_042a_dcm_sp	-3955.7917440	1005	0.01	asw_6a6_058_dcm_sp	-4107.8102195	936	0.01
sw_a6a_025a_dcm_sp	-3955.7914750	1006	0.01	asw_6a6_004a_dcm_sp	-4107.8101148	943	0.01
sw_a6a_027a_dcm_sp	-3955.7913930	1006	0.01	asw_6a6_036n_dcm_sp	-4107.8098943	941	0.01
sw_a6a_053a_dcm_sp	-3955.7912338	1004	0.00	asw_6a6_028_dcm_sp	-4107.8097038	939	0.01
sw_a6a_041b_dcm_sp	-3955.7911459	1002	0.00	asw_6a6_011a_dcm_sp	-4107.8096103	938	0.01
sw_a6a_049b_dcm_sp	-3955.7910002	1007	0.00	asw_6a6_054_dcm_sp	-4107.8094442	941	0.00
	**-3955.7942465**	**1007**			**-4107.8123916**	**941**	

[a] best 20 conformers according to E_{tot} at SMD(DCM)/B3LYP-D3/6-31+G(d) level of theory.

11.4 Optimized Conformers of Additive Salts in DCM

System $PNPBF_4$ – computational data

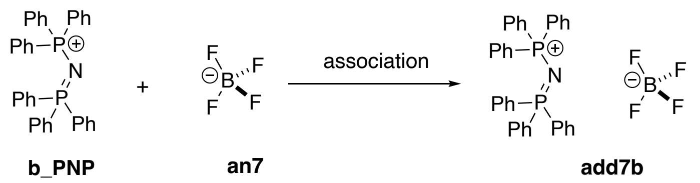

Figure S34. Ion association of additive **7b** and the single ions.

The Boltzmann-averaged free reaction energy of the ion pairing of **7b** amounts to $\Delta G_{qh,298,corr}$ = +13.9 kJ mol^{-1} in DCM solution. Focusing only on the best conformers of the reactants and product the free reaction energy of the ion pairing of **7b** changes to $\Delta G_{qh,298,corr}$ = +12.5 kJ mol^{-1} in DCM solution. The blue marked cells show the Boltzmann-averaged values.

According to $\Delta G_{qh,298,corr} = -RT \ln K$ and assuming R = 8.314 J K^{-1} mol^{-1} and T = 298.15 K, the respective equilibrium constant amounts to *K*(**7b**, DCM) = 0.007 for the best conformer of **7b** and to *K*(**7b**, DCM) = 0.004 for the Boltzmann-averaged free reaction energy. In this case the equilibrium constant *K* corresponds to the concentration of the reactants and products in the following way: *K* = [**7b**]/[$\mathbf{BF_4}$] [**b_PNP**] = [**7b**]/[$\mathbf{BF_4}$]^2.

Table S64. Energies for all systems shown in Figure S34.

System	E_{tot} SMD(DCM)/ B3LYP-D3/ 6-31+G(d)	H_{298} SMD(DCM)/ B3LYP-D3/ 6-31+G(d)	$G_{qh,298}$ SMD(DCM)/ B3LYP-D3/ 6-31+G(d)	$G_{qh,298,corr}$ SMD(DCM)/ B3LYP-D3/ 6-31+G(d)	Cavity Volume (Å^3)	Relative Population Parameter based on $G_{qh,298}$
**BF_4**						
borate_001_dcm	**-424.6525330**	**-424.6336440**	**-424.6644660**	**-424.6614475**	**69.7**	1.00
			**-424.6644660**	**-424.6614475**	**69.7**	
**b_PNP**						
cation_c_002	**-2127.413003**	**-2126.821082**	**-2126.913057**	**-2126.910039**	**560**	0.51
cation_c_003	-2127.412921	-2126.820643	-2126.912356	-2126.909338	559	0.24
cation_c_001	-2127.412908	-2126.820541	-2126.912386	-2126.909367	559	0.25
			**-2126.912720**	**-2126.909702**	**559**	
**7b**[a]						
add7b_012_dcm	**-2552.0780873**	**-2551.4650303**	**-2551.5697613**	**-2551.5667428**	**628**	0.08

add7b_112_dcm	-2552.0779330	-2551.4648320	-2551.5696060	-2551.5665875	629	0.07
add7b_145_dcm	-2552.0780671	-2551.4649881	-2551.5695071	-2551.5664886	628	0.06
add7b_080_dcm	-2552.0775340	-2551.4646350	-2551.5693790	-2551.5663605	629	0.05
add7b_040_dcm	-2552.0777464	-2551.4645434	-2551.5691644	-2551.5661459	629	0.04
add7b_025_dcm	-2552.0775889	-2551.4644939	-2551.5690929	-2551.5660744	629	0.04
add7b_008_dcm	-2552.0777622	-2551.4645002	-2551.5690812	-2551.5660627	629	0.04
add7b_031_dcm	-2552.0775467	-2551.4644087	-2551.5690747	-2551.5660562	629	0.04
add7b_142_dcm	-2552.0774378	-2551.4643418	-2551.5690398	-2551.5660213	629	0.04
add7b_044_dcm	-2552.0774876	-2551.4643126	-2551.5689506	-2551.5659321	629	0.03
add7b_075_dcm	-2552.0772426	-2551.4641026	-2551.5689176	-2551.5658991	629	0.03
add7b_124_dcm	-2552.0772946	-2551.4640796	-2551.5688946	-2551.5658761	629	0.03
add7b_004_dcm	-2552.0771107	-2551.4640887	-2551.5688207	-2551.5658022	628	0.03
add7b_056_dcm	-2552.0770696	-2551.4640306	-2551.5686996	-2551.5656811	628	0.03
add7b_024_dcm	-2552.0772159	-2551.4639719	-2551.5686949	-2551.5656764	630	0.03
add7b_107_dcm	-2552.0771499	-2551.4639409	-2551.5686399	-2551.5656214	629	0.02
add7b_058_dcm	-2552.0768889	-2551.4638929	-2551.5686269	-2551.5656084	629	0.02
add7b_027_dcm	-2552.0771886	-2551.4639656	-2551.5686256	-2551.5656071	630	0.02
add7b_010_dcm	-2552.0769587	-2551.4638597	-2551.5686037	-2551.5655852	629	0.02
add7b_147_dcm	-2552.0767477	-2551.4637577	-2551.5685927	-2551.5655742	628	0.02
**all**			**-2551.5688566**	**-2551.5658381**	**629**	
ΔE	-33.0	-27.1	+20.4	+12.5		
**all**			**+21.9**	**+13.9**		

[a] best 20 conformers according to $G_{qh,298}$ at SMD(DCM)/B3LYP-D3/6-31+G(d) level of theory.

System NBu_4BF_4 – computational data

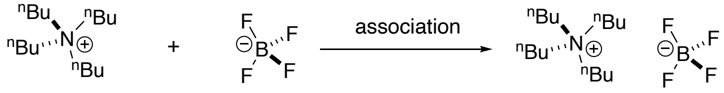

c_NBu$_4$ **an7** **add7c**

Figure S35. Ion association of additive **7c** and the single ions.

The Boltzmann-averaged free reaction energy of the ion pairing of **7c** amounts to $\Delta G_{qh,298,corr}$ = +11.2 kJ mol^{-1} in DCM solution. Focusing only on the best conformers of the reactants and product the free reaction energy of the ion pairing of **7c** changes to $\Delta G_{qh,298,corr}$ = +9.58 kJ mol^{-1} in DCM solution. The blue marked cells show the Boltzmann-averaged values.

According to $\Delta G_{qh,298,corr} = -RT \ln K$ and assuming R = 8.314 J K^{-1} mol^{-1} and T = 298.15 K, the respective equilibrium constant amounts to *K*(**7c**, DCM) = 0.021 for the best conformer of **7c** and to *K*(**7c**, DCM) = 0.011 for the Boltzmann-averaged free reaction energy. In this case the equilibrium constant *K* corresponds to the concentration of the reactants and products in the following way: *K* = [**7c**]/[**BF$_4$**] [**c_NBu4**] = [**7c**]/[**BF$_4$**]^2.

Table S65. Energies for all systems shown in Figure S35.

System	E_{tot} SMD(DCM)/ B3LYP-D3/ 6-31+G(d)	H_{298} SMD(DCM)/ B3LYP-D3/ 6-31+G(d)	$G_{qh,298}$ SMD(DCM)/ B3LYP-D3/ 6-31+G(d)	$G_{qh,298,corr}$ SMD(DCM)/ B3LYP-D3/ 6-31+G(d)	Cavity Volume (Å^3)	Relative Population Parameter based on $G_{qh,298}$
**BF$_4$**						
borate_001_dcm	**-424.6525330**	**-424.6336440**	**-424.6644660**	**-424.6614475**	**69.7**	1.00
			**-424.6644660**	**-424.6614475**	**69.7**	
**c_NBu4**						
NBu4_002	**-686.095346**	**-685.564482**	**-685.637042**	**-685.634023**	**313**	0.99
NBu4_001c	-686.094074	-685.564092	-685.632815	-685.629797	313	0.01
NBu4_006	-686.090709	-685.558999	-685.630636	-685.627618	312	0.00
NBu4_005	-686.089721	-685.557688	-685.629512	-685.626493	312	0.00
NBu4_003	-686.089384	-685.557149	-685.628989	-685.625971	312	0.00
NBu4_004	-686.086662	-685.553694	-685.624302	-685.621283	312	0.00
			**-685.6369831**	**-685.6339647**	**313**	
**7c**[a]						
add7c_054_dcm	-1110.7624593	-1110.2095593	**-1110.2948393**	**-1110.2918208**	**382**	0.08
add7c_055_dcm	**-1110.7628299**	**-1110.2095709**	-1110.2948259	-1110.2918074	381	0.08
add7c_071_dcm	-1110.7628267	-1110.2095787	-1110.2948067	-1110.2917882	381	0.08
add7c_069_dcm	-1110.7628682	-1110.2095592	-1110.2947382	-1110.2917197	381	0.07
add7c_044_dcm	-1110.7628513	-1110.2095313	-1110.2947053	-1110.2916868	381	0.07
add7c_025_dcm	-1110.7624997	-1110.2093367	-1110.2945137	-1110.2914952	383	0.06
add7c_046_dcm	-1110.7624132	-1110.2091262	-1110.2943952	-1110.2913767	381	0.05
add7c_031_dcm	-1110.7623732	-1110.2093152	-1110.2942822	-1110.2912637	380	0.05
add7c_039_dcm	-1110.7628326	-1110.2093826	-1110.2942296	-1110.2912111	382	0.04
add7c_019_dcm	-1110.7625147	-1110.2091387	-1110.2941467	-1110.2911282	381	0.04

add7c_074_dcm	-1110.7625037	-1110.2091167	-1110.2938947	-1110.2908762	381	0.03
add7c_067_dcm	-1110.7625069	-1110.2090839	-1110.2938629	-1110.2908444	382	0.03
add7c_103_dcm	-1110.7616967	-1110.2088647	-1110.2938257	-1110.2908072	382	0.03
add7c_096_dcm	-1110.7617889	-1110.2087909	-1110.2937699	-1110.2907514	381	0.03
add7c_007_dcm	-1110.7620485	-1110.2087845	-1110.2937495	-1110.2907310	383	0.03
add7c_123_dcm	-1110.7617894	-1110.2087484	-1110.2937474	-1110.2907289	382	0.03
add7c_105_dcm	-1110.7617380	-1110.2088000	-1110.2937460	-1110.2907275	382	0.03
add7c_075_dcm	-1110.7620559	-1110.2087609	-1110.2936939	-1110.2906754	383	0.02
add7c_094_dcm	-1110.7617982	-1110.2087672	-1110.2936892	-1110.2906707	381	0.02
add7c_104_dcm	-1110.7617631	-1110.2086991	-1110.2936601	-1110.2906416	382	0.02
**all**			**-1110.2941800**	**-1110.2911615**	**382**	
ΔE	-39.4	-30.1	+17.5	+9.58		
**all**			**+19.1**	**+11.2**		

[a] best 20 conformers according to $G_{qh,298}$ at SMD(DCM)/B3LYP-D3/6-31+G(d) level of theory.

System PBu_4BF_4 – computational data

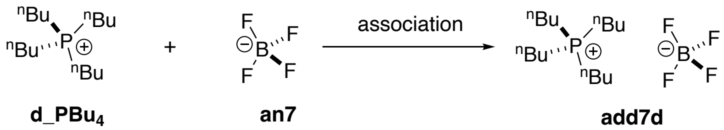

Figure S36. Ion association of additive **7d** and the single ions.

The Boltzmann-averaged free reaction energy of the ion pairing of **7d** amounts to $\Delta G_{qh,298,corr}$ = +5.97 kJ mol^{-1} in DCM solution. Focusing only on the best conformers of the reactants and product the free reaction energy of the ion pairing of **7d** changes to $\Delta G_{qh,298,corr}$ = +5.82 kJ mol^{-1} in DCM solution. The blue marked cells show the Boltzmann-averaged values.

According to $\Delta G_{qh,298,corr} = -RT \ln K$ and assuming R = 8.314 J K^{-1} mol^{-1} and T = 298.15 K, the respective equilibrium constant amounts to *K*(**7d**, DCM) = 0.096 for the best conformer of **7d** and to *K*(**7d**, DCM) = 0.090 for the Boltzmann-averaged free reaction energy. In this case the equilibrium constant *K* corresponds to the concentration of the reactants and products in the following way: *K* = [**7d**]/[$\mathbf{BF_4}$] [**d_PBu4**] = [**7d**]/[$\mathbf{BF_4}$]^2.

Table S66. Energies for all systems shown in Figure S36.

System	E_{tot} SMD(DCM)/ B3LYP-D3/ 6-31+G(d)	H_{298} SMD(DCM)/ B3LYP-D3/ 6-31+G(d)	$G_{qh,298}$ SMD(DCM)/ B3LYP-D3/ 6-31+G(d)	$G_{qh,298,corr}$ SMD(DCM)/ B3LYP-D3/ 6-31+G(d)	Cavity Volume (Å^3)	Relative Population Parameter based on $G_{qh,298}$
**BF_4**						
borate_001_dcm	**-424.6525330**	**-424.6336440**	**-424.6644660**	**-424.6614475**	**69.7**	1.00
			**-424.6644660**	**-424.6614475**	**69.7**	
**d_PBu4**						
PBu4_001_dcm_fr	**-972.730410**	**-972.207922**	**-972.283263**	**-972.280245**	**326**	0.71
PBu4_002b_pos	-972.729488	-972.206599	-972.282049	-972.279030	327	0.20
PBu4_003_dcm_fr	-972.727645	-972.204727	-972.280491	-972.277472	326	0.04
PBu4_005_dcm_fr	-972.728208	-972.205291	-972.280338	-972.277319	326	0.03
PBu4_004_dcm_fr	-972.727749	-972.204384	-972.279426	-972.276408	326	0.01
PBu4_006_dcm_fr	-972.726367	-972.203286	-972.278751	-972.275732	326	0.01
PBu4_007_dcm_fr	-972.725758	-972.202871	-972.278552	-972.275534	327	0.00
			**-972.282730**	**-972.279711**	**326**	
**7d**[a]						
add7d_062_dcm	-1397.3971810	-1396.8536460	**-1396.9424950**	**-1396.9394765**	**396**	0.06
add7d_015_dcm	**-1397.3975433**	**-1396.8537783**	-1396.9423173	-1396.9392988	396	0.05
add7d_097_dcm	-1397.3965382	-1396.8531322	-1396.9421622	-1396.9391437	396	0.04
add7d_082_dcm	-1397.3970628	-1396.8535218	-1396.9421588	-1396.9391403	395	0.04
add7d_134_dcm	-1397.3970898	-1396.8534568	-1396.9421548	-1396.9391363	396	0.04
add7d_031_dcm	-1397.3971864	-1396.8534744	-1396.9421394	-1396.9391209	396	0.04
add7d_081_dcm	-1397.3970494	-1396.8534964	-1396.9421364	-1396.9391179	395	0.04
add7d_091_dcm	-1397.3965428	-1396.8530588	-1396.9421358	-1396.9391173	396	0.04
add7d_089_dcm	-1397.3970672	-1396.8534242	-1396.9421092	-1396.9390907	396	0.04
add7d_138_dcm	-1397.3970898	-1396.8534328	-1396.9420748	-1396.9390563	396	0.04
add7d_135_dcm	-1397.3965485	-1396.8530665	-1396.9420705	-1396.9390520	396	0.04
add7d_085_dcm	-1397.3970885	-1396.8534225	-1396.9420565	-1396.9390380	396	0.04
add7d_046_dcm	-1397.3970089	-1396.8532869	-1396.9420429	-1396.9390244	396	0.04
add7d_117_dcm	-1397.3970913	-1396.8534273	-1396.9420413	-1396.9390228	396	0.04
add7d_032_dcm	-1397.3967888	-1396.8533268	-1396.9419428	-1396.9389243	396	0.03
add7d_131_dcm	-1397.3970733	-1396.8533643	-1396.9419293	-1396.9389108	396	0.03
add7d_056_dcm	-1397.3967936	-1396.8533546	-1396.9419266	-1396.9389081	396	0.03

add7d_084_dcm	-1397.3969363	-1396.8531233	-1396.9418323	-1396.9388138	397	0.03
add7d_001_dcm	-1397.3970497	-1396.8532797	-1396.9418177	-1396.9387992	396	0.03
add7d_139_dcm	-1397.3964290	-1396.8527250	-1396.9415420	-1396.9385235	395	0.02
**all**			**-1396.9419027**	**-1396.9388842**	**396**	
ΔE	-38.3	-32.1	+13.7	+5.82		
**all**			**+13.9**	**+5.97**		

[a] best 20 conformers according to $G_{qh,298}$ at SMD(DCM)/B3LYP-D3/6-31+G(d) level of theory.

11.5 Optimized Conformers and Methyl Cation Affinities of Neutral Lewis Base Catalysts in DCM

Neutral organocatalysts DMAP (**1**) and TCAP (**2**) were used as reference compounds. Their energies and methyl cation affinities were calculated at at SMD(DCM)/B3LYP-D3/6-31+G(d) level of theory. The blue marked cells show the Boltzmann-averaged values.

System DMAP – computational data

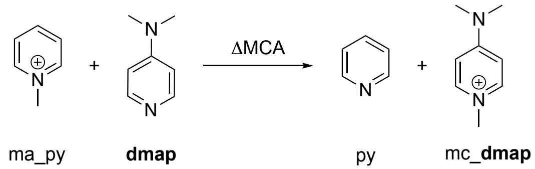

Figure S37. Equation used for calculation of relative Lewis basicity of DMAP (**1**) with pyridine as reference.

Table S67. Energy for all systems shown in Figure S37.

System	E_{tot} SMD(DCM)/ B3LYP-D3/ 6-31+G(d)	H_{298} SMD(DCM)/ B3LYP-D3/ 6-31+G(d)	$G_{qh,298}$ SMD(DCM)/ B3LYP-D3/ 6-31+G(d)	$G_{qh,298,corr}$ SMD(DCM)/ B3LYP-D3/ 6-31+G(d)
**Pyridine**				
py_001_dcm_fr	-248.3117009	-248.2175679	-248.2502039	-248.2471854
ma_py_001_dcm_fr	-288.0755143	-287.9375633	-287.9743763	-287.9713578
**DMAP (1)**				
dmap_001_dcm	-382.301346	-382.129303	-382.171771	-382.168753
mc_dmap_001_dcm_ofr	-422.0778555	-421.8616535	-421.9077655	-421.9047470

**ΔMCA**		**-32.44**		**-31.04**

System TCAP – computational data

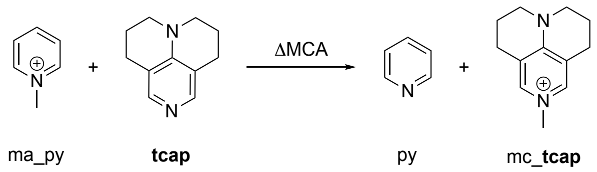

Figure S38. Equation used for calculation of relative Lewis basicity of TCAP (**2**) with pyridine as reference.

Table S68. Energy for all systems shown in Figure S38.

**System**	**E_{tot} SMD(DCM)/ B3LYP-D3/ 6-31+G(d)**	**H_{298} SMD(DCM)/ B3LYP-D3/ 6-31+G(d)**	**$G_{qh,298}$ SMD(DCM)/ B3LYP-D3/ 6-31+G(d)**	**$G_{qh,298,corr}$ SMD(DCM)/ B3LYP-D3/ 6-31+G(d)**
**Pyridine**				
py_001_dcm_fr	-248.3117009	-248.2175679	-248.2502039	-248.2471854
ma_py_001_dcm_fr	-288.0755143	-287.9375633	-287.9743763	-287.9713578
**TCAP (2)**				
tcap_001_dcm	**-537.180523**	**-536.933789**	**-536.980044**	**-536.977026**
tcap_002_dcm	-537.180206	-536.933432	-536.979826	-536.976808
		**-536.9336439**	**-536.9799476**	**-536.9769292**
**Me_TCAP**				
mc_tcap_002_dcm_ofr	**-576.9609333**	**-576.6701483**	**-576.7200573**	**-576.7170388**
mc_tcap_001_dcm_ofr	-576.9603169	-576.6692849	-576.7192719	-576.7162534
		**-576.6699013**	**-576.7198191**	**-576.7168006**
ΔMCA		-42.70		-41.59
**all**		**-42.96**		**-41.22**

11.6 XYZ-Coordinates of Most Stable Compounds

Structures of anion **3**, cation **a**, ion pair **3a** and **4a**, sandwich complexes **a3a**, **a4a**, **3a3** and **4a4**, and additive **7a** can be found in ref. 2.

Anion **5** (an5_001_dcm)

C	-3.065798	-0.473954	-1.145442
C	-3.735185	-1.637410	-0.798602
N	-3.500961	-2.349144	0.325692
C	-2.548289	-1.848417	1.134157
C	-1.818227	-0.689242	0.889526
C	-2.053752	0.056064	-0.298917
N	-1.424384	1.199663	-0.728407
S	-0.198410	1.845865	0.101644
O	0.241358	3.047548	-0.655982
O	-0.469728	2.061002	1.553988
C	1.175764	0.664434	0.022887
C	1.780435	0.197053	1.188721
C	2.840287	-0.712820	1.098584
C	3.302309	-1.166769	-0.144007
C	2.674450	-0.681660	-1.306213
C	1.619993	0.226006	-1.230766
C	4.435538	-2.159226	-0.247855
H	-3.304965	0.041239	-2.072292
H	-4.506522	-2.031151	-1.459728
H	-2.354624	-2.412096	2.046632
H	-1.087477	-0.362990	1.619582
H	1.425234	0.539215	2.155641
H	3.309589	-1.074833	2.010863
H	3.017943	-1.022019	-2.281147
H	1.141855	0.587716	-2.136797
H	5.229625	-1.785418	-0.906690
H	4.876862	-2.369850	0.732186
H	4.086264	-3.110041	-0.672048

Anion **6** (an6_001)

C	3.633271	1.094783	-1.182424
C	4.003889	2.380152	-0.817365
N	3.638564	2.983298	0.334562
C	2.868527	2.242834	1.152767
C	2.442009	0.943803	0.891709
C	2.816289	0.310727	-0.324418
N	2.476394	-0.945609	-0.773420
S	1.500259	-1.907686	0.069816
O	1.350100	-3.163954	-0.708224

O	1.855683	-2.069232	1.509040
C	-0.143761	-1.123594	0.055866
C	-0.794495	-0.826276	1.251925
C	-2.053526	-0.218731	1.220429
C	-2.639367	0.085850	-0.009566
C	-1.980677	-0.211402	-1.212010
C	-0.728470	-0.817527	-1.179042
C	-3.992741	0.733249	-0.080392
H	3.961669	0.677293	-2.130759
H	4.629894	2.969398	-1.486479
H	2.572107	2.718817	2.087157
H	1.841826	0.427906	1.631415
H	-0.322818	-1.065368	2.199095
H	-2.565102	0.014088	2.148381
H	-2.443943	0.029915	-2.164579
H	-0.207931	-1.046330	-2.103875
F	-4.524596	1.000690	1.137692
F	-4.901462	-0.046446	-0.737218
F	-3.963553	1.916390	-0.760118

Methylated anion **6** (ma_an6_001_n1_dcm_fr)

C	-3.444930	-0.204143	-1.357486
C	-4.076588	-1.388525	-1.097709
C	-3.029813	-1.544107	0.997720
C	-2.367207	-0.358708	0.801134
C	-2.540012	0.382708	-0.413577
N	-1.961074	1.542106	-0.782445
S	-0.874904	2.310368	0.172889
O	-0.542893	3.573962	-0.518567
O	-1.285289	2.403000	1.597724
C	0.617864	1.282905	0.122997
C	1.099719	0.692043	1.289515
C	2.241534	-0.112732	1.228859
C	2.876223	-0.314134	0.002247
C	2.388100	0.285214	-1.168307
C	1.253981	1.088978	-1.109047
C	4.100358	-1.181540	-0.099565
H	-3.631227	0.303790	-2.297687
H	-4.761610	-1.847717	-1.801724
H	-2.909628	-2.119522	1.908770
H	-1.731445	0.005062	1.596484
H	0.596084	0.860542	2.235416
H	2.625472	-0.574184	2.132344
H	2.892030	0.125435	-2.117287

H	0.868388	1.558122	-2.008693
F	4.482147	-1.709592	1.088331
F	5.174564	-0.498216	-0.587171
F	3.911022	-2.232263	-0.949798
N	-3.879227	-2.063832	0.072545
C	-4.530970	-3.365654	0.290217
H	-5.554275	-3.323085	-0.088044
H	-3.975956	-4.150002	-0.233394
H	-4.550969	-3.581140	1.359379

Methylated anion **6** (ma_an6_001_n2_dcm_fr)

C	1.983354	1.506284	-1.194988
C	2.245430	2.842321	-0.886688
N	3.044488	3.235900	0.116425
C	3.625873	2.268375	0.846053
C	3.444897	0.905373	0.616377
C	2.594196	0.509477	-0.423637
S	1.500378	-1.752513	0.482070
O	1.476175	-3.146255	0.021064
O	2.055391	-1.410183	1.796106
C	-0.166496	-1.088444	0.387558
C	-0.477258	0.055815	1.126316
C	-1.747654	0.617691	0.995237
C	-2.674360	0.032341	0.128044
C	-2.354268	-1.116919	-0.604905
C	-1.087703	-1.684165	-0.479273
C	-4.058770	0.607595	-0.012846
H	1.314289	1.264025	-2.012792
H	1.779798	3.632180	-1.472262
H	4.280102	2.598280	1.650016
H	3.950742	0.168727	1.228494
H	0.251350	0.498634	1.796621
H	-2.005968	1.503966	1.564428
H	-3.085874	-1.567234	-1.268925
H	-0.825264	-2.577454	-1.035691
F	-4.180033	1.840060	0.535455
F	-4.998279	-0.179387	0.585404
F	-4.433777	0.717341	-1.316299
N	2.386707	-0.881076	-0.694713
C	2.085266	-1.262373	-2.091184
H	1.066638	-0.994232	-2.398853
H	2.802823	-0.744071	-2.730801
H	2.228200	-2.337227	-2.200123

Cation **b_PNP (**cation_c_002)

C	-2.585642	-0.802779	-1.424816
P	-1.483020	0.075381	-0.277568
C	-1.649516	-0.727488	1.348546
C	-1.557442	-2.129467	1.408840
C	-1.592137	-2.778854	2.642926
C	-1.718427	-2.035166	3.822195
C	-1.805888	-0.641456	3.764863
C	-1.769230	0.015203	2.532047
C	-2.065108	1.794137	-0.130803
C	-3.362440	2.084736	0.323207
C	-3.783389	3.411462	0.422286
C	-2.916129	4.451794	0.065886
C	-1.627765	4.163292	-0.393523
C	-1.200303	2.836055	-0.493416
N	-0.006339	0.040506	-0.897333
P	1.482661	-0.047899	-0.310537
C	2.524681	1.116510	-1.240682
C	3.762682	1.541044	-0.731566
C	4.559456	2.412085	-1.477564
C	4.125343	2.862222	-2.729379
C	2.891082	2.441803	-3.236017
C	2.089557	1.569744	-2.495316
C	2.132544	-1.732115	-0.542662
C	1.261419	-2.740604	-0.977734
C	1.733728	-4.045483	-1.145569
C	3.073192	-4.344896	-0.880498
C	3.945313	-3.337647	-0.449147
C	3.479210	-2.033157	-0.279733
C	1.646831	0.378481	1.450175
C	1.516870	1.726370	1.827463
C	1.559459	2.079310	3.176463
C	1.731213	1.091605	4.153596
C	1.856865	-0.249520	3.780172
C	1.813667	-0.609878	2.430992
C	-2.180815	-1.001123	-2.753280
C	-3.037565	-1.640962	-3.652532
C	-4.296261	-2.081813	-3.230704
C	-4.699590	-1.885769	-1.905124
C	-3.847908	-1.247715	-1.000925
H	-1.460657	-2.712567	0.497325
H	-1.522171	-3.862581	2.683493
H	-1.744988	-2.542160	4.783226
H	-1.893427	-0.060738	4.679042

H	-1.825373	1.098596	2.501559
H	-4.040099	1.282210	0.602633
H	-4.786372	3.633367	0.776957
H	-3.247351	5.484119	0.143813
H	-0.954179	4.968114	-0.676027
H	-0.202976	2.611383	-0.858089
H	4.100889	1.204396	0.244716
H	5.514902	2.742278	-1.078679
H	4.746195	3.542665	-3.306457
H	2.549024	2.794435	-4.205505
H	1.127230	1.249319	-2.882648
H	0.222696	-2.505192	-1.186218
H	1.054999	-4.823932	-1.484239
H	3.439944	-5.359681	-1.011687
H	4.987724	-3.567661	-0.245405
H	4.163419	-1.258335	0.055683
H	1.384452	2.496907	1.072739
H	1.460543	3.122717	3.463798
H	1.764437	1.367904	5.204294
H	1.981538	-1.019025	4.537141
H	1.902880	-1.655020	2.151596
H	-1.201029	-0.663657	-3.077373
H	-2.719891	-1.796462	-4.680227
H	-4.960404	-2.580716	-3.931755
H	-5.673872	-2.233901	-1.572401
H	-4.163672	-1.112322	0.029611

Cation **c_NBu4** (NBu4_002_dcm_fr)

N	0.000512	0.000189	-0.000535
C	-0.851992	0.876262	0.919568
C	-1.808668	1.848921	0.237805
C	0.852397	-0.876403	0.919228
C	1.807792	-1.850105	0.237377
C	-0.875630	-0.852074	-0.920557
C	-1.854226	-1.802507	-0.238492
C	0.877856	0.851533	-0.920142
C	1.856285	1.801667	-0.237550
H	-0.147995	1.418437	1.553259
H	-1.403869	0.190051	1.563597
H	-1.260444	2.550978	-0.401410
H	-2.522856	1.312169	-0.397441
H	0.148399	-1.416566	1.554498
H	1.405885	-0.189782	1.561400
H	1.258259	-2.551411	-0.401520

H	2.522156	-1.314306	-0.398551
H	-0.188338	-1.409406	-1.558745
H	-1.412061	-0.148892	-1.559997
H	-1.322668	-2.515575	0.402253
H	-2.556640	-1.248727	0.395590
H	0.191694	1.408847	-1.559370
H	1.414251	0.147696	-1.558851
H	2.559547	1.247933	0.395573
H	1.324514	2.513643	0.404407
C	2.649090	2.584475	-1.297016
H	3.185344	1.878345	-1.946296
H	1.949626	3.135131	-1.941492
C	3.645387	3.562432	-0.666839
H	4.202476	4.109023	-1.437404
H	3.131005	4.300544	-0.037496
H	4.373457	3.034486	-0.037097
C	-2.587245	2.645009	1.297781
H	-3.136953	1.947794	1.945523
H	-1.878299	3.181783	1.943500
C	-3.565637	3.641163	0.668140
H	-4.303383	3.126678	0.038509
H	-4.112504	4.197600	1.438985
H	-3.038002	4.369832	0.038754
C	-2.649172	-2.583339	-1.297692
H	-1.951322	-3.135629	-1.942524
H	-3.184038	-1.875771	-1.946482
C	-3.647593	-3.558857	-0.667128
H	-4.203730	-4.106856	-1.437386
H	-3.135003	-4.295759	-0.034964
H	-4.376399	-3.028710	-0.040028
C	2.585838	-2.646825	1.297425
H	1.876639	-3.184616	1.942089
H	3.134385	-1.949815	1.946283
C	3.565366	-3.642071	0.668122
H	4.107194	-4.203263	1.439090
H	3.039516	-4.366645	0.032612
H	4.307483	-3.126251	0.044689

Cation **d_PBu4** (PBu4_001_dcm_fr)

P	0.000047	0.000009	0.000002
C	-1.044155	1.043394	1.071693
C	-1.989677	1.987591	0.311852
C	1.043433	1.044231	-1.071675
C	1.044251	-1.043402	1.071672

C	-1.043335	-1.044202	-1.071685
C	1.989625	-1.987743	0.311825
C	-1.988290	-1.988967	-0.311848
C	1.988305	1.989072	-0.311829
H	-0.360489	1.608071	1.718023
H	-1.607829	0.359329	1.718474
H	-1.406706	2.651837	-0.339953
H	-2.653460	1.403662	-0.339567
H	0.359322	1.608425	-1.717955
H	1.607588	0.360610	-1.718508
H	0.360588	-1.607966	1.718103
H	1.608046	-0.359343	1.718355
H	-0.359213	-1.608464	-1.717895
H	-1.607420	-0.360591	-1.718588
H	1.406540	-2.652013	-0.339850
H	2.653383	-1.403928	-0.339723
H	-1.404964	-2.652701	0.340162
H	-2.652589	-1.405396	0.339366
H	1.404918	2.652789	0.340143
H	2.652614	1.405557	0.339424
C	2.836004	-2.833374	1.272754
H	3.414198	-2.165454	1.926825
H	2.168594	-3.412201	1.926786
C	3.785253	-3.781739	0.533932
H	4.482007	-3.224099	-0.105900
H	4.380070	-4.376045	1.238403
H	3.228262	-4.478970	-0.105943
C	-2.833866	-2.835391	-1.272785
H	-3.412218	-2.168002	-1.927258
H	-2.165915	-3.414048	-1.926414
C	-3.782829	-3.784052	-0.533979
H	-4.377091	-4.378901	-1.238461
H	-3.225664	-4.480780	0.106293
H	-4.480099	-3.226598	0.105453
C	-2.836009	2.833264	1.272784
H	-2.168570	3.412184	1.926704
H	-3.414105	2.165369	1.926967
C	-3.785379	3.781512	0.533968
H	-4.380183	4.375825	1.238443
H	-3.228484	4.478741	-0.105994
H	-4.482139	3.223780	-0.105775
C	2.833866	2.835526	-1.272754
H	3.412293	2.168161	-1.927186
H	2.165903	3.414121	-1.926425

C	3.782736	3.784266	-0.533929
H	4.376999	4.379128	-1.238398
H	3.225499	4.480983	0.106294
H	4.480003	3.226871	0.105557

Ion pair **3b** (cat3b_124_dcm_fr)

C	4.341054	1.226233	-1.876473
C	4.379087	2.280470	-2.785689
N	3.403654	3.192661	-2.948225
C	2.326597	3.041409	-2.151593
C	2.183998	2.028816	-1.212618
C	3.209418	1.069672	-1.042039
N	2.975273	0.090975	-0.084553
S	3.984081	-1.065645	0.273631
O	3.313317	-2.044382	1.154779
O	4.849766	-1.604205	-0.802946
C	5.253246	-0.288735	1.454299
F	4.649601	0.255956	2.532156
F	6.134266	-1.210421	1.898553
F	5.953061	0.689531	0.839039
H	5.173245	0.534993	-1.829061
H	5.252373	2.396126	-3.426392
H	1.529423	3.771709	-2.275424
H	1.281205	1.966461	-0.615977
C	-1.514846	2.934505	-0.357417
P	-1.755350	1.333404	0.468539
C	-3.532609	1.177312	0.849692
C	-4.484243	1.929325	0.143656
C	-5.847505	1.706369	0.355100
C	-6.266282	0.733912	1.268559
C	-5.319566	-0.012949	1.979067
C	-3.956965	0.207384	1.773308
C	-0.856006	1.352425	2.048428
C	-1.438859	1.860709	3.221814
C	-0.699463	1.896234	4.406177
C	0.620041	1.429761	4.423507
C	1.201073	0.928847	3.254527
C	0.467886	0.887794	2.067097
N	-1.205163	0.223173	-0.547443
P	-1.239690	-1.374733	-0.678705
C	0.172901	-1.855521	-1.713804
C	0.735281	-0.905187	-2.577671
C	1.791086	-1.266641	-3.416196
C	2.288673	-2.572880	-3.393393

C	1.726853	-3.522481	-2.533083
C	0.667431	-3.169100	-1.695425
C	-2.750022	-1.930542	-1.539034
C	-2.987144	-3.300226	-1.743248
C	-4.130831	-3.715901	-2.426168
C	-5.041467	-2.768872	-2.911334
C	-4.802983	-1.405553	-2.716166
C	-3.657464	-0.984933	-2.034018
C	-1.131063	-2.295004	0.886877
C	0.116950	-2.398590	1.522975
C	0.210647	-3.013883	2.772166
C	-0.934365	-3.524610	3.392820
C	-2.178298	-3.423135	2.760196
C	-2.280058	-2.809656	1.509433
C	-1.462808	2.983140	-1.758559
C	-1.324459	4.212241	-2.407369
C	-1.233812	5.392600	-1.662546
C	-1.280236	5.344100	-0.264935
C	-1.422643	4.118510	0.389567
H	-4.168607	2.683273	-0.571504
H	-6.579900	2.292102	-0.194277
H	-7.327201	0.560310	1.429746
H	-5.640694	-0.766145	2.693810
H	-3.229104	-0.373537	2.330885
H	-2.463140	2.221855	3.217925
H	-1.155119	2.285031	5.313141
H	1.192571	1.455125	5.347359
H	2.223697	0.563371	3.259547
H	0.936292	0.492536	1.171815
H	0.369718	0.116053	-2.572244
H	2.238065	-0.521014	-4.067962
H	3.123687	-2.847158	-4.032771
H	2.121956	-4.534346	-2.502590
H	0.246984	-3.908814	-1.020219
H	-2.285137	-4.039825	-1.367320
H	-4.311407	-4.776697	-2.579199
H	-5.932488	-3.094970	-3.441840
H	-5.505904	-0.666813	-3.092431
H	-3.473946	0.073536	-1.887185
H	1.017086	-2.010705	1.057659
H	1.180068	-3.089237	3.257208
H	-0.857636	-4.002862	4.366096
H	-3.069881	-3.820947	3.238018
H	-3.249755	-2.732850	1.028055

H	-1.514371	2.064315	-2.334439
H	-1.276044	4.244916	-3.492580
H	-1.117304	6.347174	-2.169185
H	-1.199683	6.258410	0.316940
H	-1.451452	4.090122	1.475031

Ion pair **3c** (cat3c_001_dcm)

C	4.262325	0.945725	-0.588330
C	5.196066	1.829590	-0.052616
N	4.916118	2.799053	0.837107
C	3.626527	2.890665	1.216237
C	2.614795	2.062026	0.748846
C	2.910027	1.043700	-0.187524
N	1.832570	0.268610	-0.594486
S	1.911769	-0.940795	-1.601585
O	0.538732	-1.353177	-1.963300
O	2.917160	-0.881545	-2.689530
C	2.508788	-2.424516	-0.579060
F	1.654504	-2.696948	0.428262
F	2.611799	-3.531666	-1.343667
F	3.721002	-2.178850	-0.036764
H	4.581960	0.200834	-1.306857
H	6.237555	1.751769	-0.361251
H	3.392663	3.673546	1.936186
H	1.594371	2.190769	1.096831
N	-2.311309	0.307408	0.483112
C	-3.706699	0.896339	0.662904
C	-4.489067	1.181028	-0.614843
C	-1.436986	1.197148	-0.405003
C	-1.714948	0.181203	1.886031
C	-2.364559	-1.049930	-0.216675
C	-0.386557	-0.561488	1.984750
C	-3.186043	-2.131363	0.475636
C	-1.247885	2.642814	0.043189
H	-3.575286	1.813329	1.240804
H	-4.252655	0.191064	1.291843
H	-3.957123	1.906137	-1.241527
H	-4.612715	0.266355	-1.206007
H	-1.894517	1.164608	-1.395049
H	-0.469788	0.700396	-0.477582
H	-2.473794	-0.311361	2.497409
H	-1.613839	1.202920	2.255348
H	-1.327519	-1.362194	-0.340950
H	-2.756598	-0.858256	-1.216791

H	-0.531594	-1.627325	1.774446
H	0.332243	-0.187390	1.250236
H	-2.847515	-2.278346	1.507886
H	-4.245346	-1.850135	0.514897
H	-2.209806	3.155747	0.162754
H	-0.731798	2.684297	1.009916
C	-5.876921	1.742782	-0.267013
H	-6.411856	1.023704	0.369373
H	-5.760152	2.660876	0.325905
C	-6.710271	2.041541	-1.517051
H	-7.695220	2.443096	-1.248834
H	-6.210115	2.779060	-2.158409
H	-6.868838	1.132886	-2.112555
C	-3.047655	-3.459095	-0.286591
H	-1.987886	-3.746672	-0.322426
H	-3.368132	-3.316893	-1.328249
C	-3.865995	-4.583451	0.355333
H	-3.751062	-5.522541	-0.199796
H	-3.545072	-4.764251	1.389808
H	-4.935243	-4.333997	0.375133
C	0.203677	-0.404099	3.394814
H	0.378948	0.662238	3.596680
H	-0.525686	-0.748741	4.142327
C	1.515323	-1.178090	3.562100
H	1.927005	-1.043022	4.570076
H	1.363088	-2.253216	3.402050
H	2.269283	-0.837567	2.841875
C	-0.405281	3.394434	-1.001344
H	0.535281	2.853841	-1.165847
H	-0.938882	3.396124	-1.962212
C	-0.103022	4.833828	-0.575968
H	0.492146	5.352993	-1.337141
H	-1.027790	5.406324	-0.423337
H	0.464604	4.856409	0.363492

Ion pair **3d** (cat3d_031_dcm_fr)

C	4.197904	-0.055734	0.780408
C	5.070101	-0.793496	1.577026
N	4.871766	-2.068144	1.958557
C	3.738272	-2.642774	1.511146
C	2.800857	-2.001841	0.711473
C	3.005088	-0.658557	0.319985
N	2.003695	-0.097399	-0.461429
S	1.924838	1.424958	-0.862101

O	0.573748	1.713696	-1.390403
O	2.518426	2.420744	0.061877
C	2.999912	1.607504	-2.414365
F	2.594466	0.763860	-3.385308
F	2.941267	2.863184	-2.904679
F	4.292300	1.329351	-2.138715
H	4.438629	0.971989	0.538363
H	5.986387	-0.325659	1.934080
H	3.572623	-3.675829	1.813429
H	1.906589	-2.524129	0.385964
P	-2.323469	-0.382192	0.446657
C	-1.287821	-1.173876	-0.824254
C	-0.978832	-2.657260	-0.570395
C	-1.606801	-0.628222	2.105768
C	-2.431856	1.395405	0.064527
C	-3.989145	-1.130071	0.456916
C	-3.263837	2.213745	1.063572
C	-4.777022	-0.957029	-0.851665
C	-0.229673	0.023351	2.303747
H	-0.360575	-0.593431	-0.880455
H	-1.814118	-1.038073	-1.777638
H	-0.437545	-2.762197	0.378922
H	-1.908766	-3.234378	-0.473198
H	-1.553453	-1.712938	2.262604
H	-2.336662	-0.236072	2.825046
H	-2.848120	1.470315	-0.947878
H	-1.400440	1.760328	0.004172
H	-4.531034	-0.682961	1.299240
H	-3.850164	-2.193088	0.692368
H	-4.289718	1.823226	1.108324
H	-2.839731	2.113087	2.071665
H	-4.903732	0.111673	-1.069794
H	-4.212070	-1.388020	-1.688747
H	0.460536	-0.313865	1.523645
H	-0.315525	1.112373	2.192866
C	-6.156412	-1.625476	-0.773161
C	-6.962428	-1.457868	-2.064906
H	-6.716407	-1.197838	0.070736
H	-6.026702	-2.694815	-0.553401
H	-7.943330	-1.942933	-1.986254
H	-7.130385	-0.396510	-2.290954
H	-6.435944	-1.902522	-2.919798
C	0.358915	-0.302929	3.682564
C	1.736503	0.333553	3.892179

H	0.438398	-1.394431	3.789463
H	-0.334286	0.039684	4.464624
H	2.135333	0.097776	4.886848
H	2.454255	-0.027974	3.146389
H	1.684882	1.426527	3.799904
C	-0.125739	-3.248689	-1.701132
C	0.265542	-4.705552	-1.437047
H	0.778618	-2.638025	-1.822769
H	-0.681309	-3.177586	-2.647050
H	0.873472	-5.108197	-2.256685
H	0.851189	-4.794766	-0.512347
H	-0.622594	-5.343385	-1.332853
C	-3.301404	3.698926	0.677762
C	-4.121692	4.539252	1.661224
H	-3.720203	3.797763	-0.333831
H	-2.272874	4.082787	0.628928
H	-4.133994	5.595860	1.366211
H	-5.162573	4.191706	1.706677
H	-3.704537	4.479620	2.675335

Ion pair **5a** (cat5a_012_dcm_fr)

C	-1.175308	2.933356	0.089240
C	-0.640540	3.493246	-1.060234
N	-1.185342	3.377623	-2.291513
C	-2.333202	2.676216	-2.348571
C	-2.958572	2.073706	-1.261712
C	-2.374886	2.170152	0.031092
N	-2.797663	1.604321	1.206089
S	-4.114065	0.674696	1.300214
O	-4.258813	0.314131	2.735634
O	-5.324037	1.242039	0.636114
C	-3.763351	-0.884700	0.443148
C	-4.400232	-1.199911	-0.756779
C	-4.103284	-2.407231	-1.398810
C	-3.175322	-3.306984	-0.858858
C	-2.544959	-2.966912	0.351405
C	-2.834262	-1.770392	1.003031
C	-2.858011	-4.614526	-1.541964
H	-0.670377	3.054941	1.043395
H	0.289586	4.054677	-1.001031
H	-2.785601	2.584853	-3.335835
H	-3.889386	1.542711	-1.416203
H	-5.129030	-0.515935	-1.179513
H	-4.602601	-2.647836	-2.334890

H	-1.818782	-3.650517	0.786735
H	-2.347890	-1.528072	1.941985
H	-1.774738	-4.765483	-1.619626
H	-3.263420	-5.461780	-0.972521
H	-3.283208	-4.656181	-2.550414
P	2.204309	-0.156469	0.092490
C	2.553671	1.527872	-0.486763
C	1.073045	-0.942198	-1.092373
C	1.483072	-0.076152	1.756304
C	2.662198	1.785100	-1.863880
C	2.973107	3.072252	-2.304086
C	3.175921	4.101012	-1.378410
C	3.064924	3.845062	-0.007987
C	2.752326	2.561253	0.442628
C	-0.021017	-0.211997	-1.589532
C	-0.892346	-0.806095	-2.501720
C	-0.676907	-2.119827	-2.927129
C	0.412888	-2.845182	-2.437829
C	1.289309	-2.262490	-1.520245
C	2.322291	-0.091815	2.885525
C	1.769184	0.051836	4.158767
C	0.387848	0.219571	4.310838
C	-0.443725	0.247871	3.187860
C	0.099501	0.097780	1.910470
H	2.496617	0.993083	-2.588225
H	3.045200	3.271876	-3.369510
H	3.408087	5.104329	-1.725573
H	3.208647	4.645806	0.712153
H	2.652894	2.374329	1.507514
H	-0.193545	0.811490	-1.275163
H	-1.741284	-0.239013	-2.869328
H	-1.361002	-2.579138	-3.635032
H	0.584549	-3.865937	-2.768067
H	2.135627	-2.834775	-1.155731
H	3.395843	-0.212887	2.778065
H	2.418898	0.035596	5.029762
H	-0.038645	0.334186	5.304054
H	-1.514542	0.399291	3.285649
H	-0.564387	0.130325	1.054856
C	3.746789	-1.110872	0.178999
C	3.762889	-2.330199	0.879889
C	4.904807	-0.657845	-0.471268
C	4.932074	-3.090037	0.922095
H	2.872528	-2.681882	1.393609

C	6.071307	-1.425415	-0.422375
H	4.905737	0.285541	-1.008084
C	6.086032	-2.639127	0.270977
H	4.941410	-4.030659	1.465823
H	6.967355	-1.070271	-0.923937
H	6.996150	-3.231997	0.307807

Ion pair **6a** (cat6a_012_dcm)

C	0.028674	-3.442871	-0.297071
C	-0.564506	-3.666400	-1.529930
N	0.064228	-3.521656	-2.716645
C	1.355887	-3.150754	-2.643416
C	2.050943	-2.902012	-1.463840
C	1.385777	-3.027834	-0.215461
N	1.870239	-2.768764	1.043883
S	3.378632	-2.281795	1.315089
O	3.532196	-2.192167	2.789767
O	4.429944	-3.042182	0.581414
C	3.519263	-0.563744	0.725743
C	4.301571	-0.257727	-0.388902
C	4.395341	1.066494	-0.821592
C	3.700398	2.068378	-0.138100
C	2.916247	1.759415	0.980023
C	2.827221	0.438558	1.413080
C	3.847563	3.498250	-0.574287
H	-0.545158	-3.563392	0.617334
H	-1.610161	-3.962874	-1.576660
H	1.873583	-3.039347	-3.595732
H	3.095131	-2.620293	-1.516445
H	4.843198	-1.043370	-0.904522
H	5.005526	1.311194	-1.685574
H	2.380428	2.541079	1.508921
H	2.231504	0.192522	2.284665
P	-2.384705	0.433990	0.112361
C	-3.128723	-1.004849	-0.706451
C	-1.014816	1.034125	-0.920079
C	-1.811515	-0.056210	1.764180
C	-3.226571	-1.040248	-2.107613
C	-3.850588	-2.122124	-2.729848
C	-4.376546	-3.166149	-1.961911
C	-4.275274	-3.132374	-0.567434
C	-3.651604	-2.054975	0.064526
C	-0.134191	0.104613	-1.500977
C	0.919228	0.552265	-2.297464

C	1.097447	1.919934	-2.523788
C	0.220985	2.845111	-1.949917
C	-0.835460	2.408617	-1.148255
C	-2.666320	0.075263	2.873544
C	-2.248762	-0.380831	4.125057
C	-0.989878	-0.973913	4.273956
C	-0.145587	-1.115231	3.168839
C	-0.552142	-0.656094	1.914904
H	-2.812420	-0.237817	-2.710766
H	-3.915617	-2.152484	-3.813797
H	-4.854511	-4.010844	-2.450800
H	-4.671520	-3.948487	0.030378
H	-3.567205	-2.041441	1.146871
H	-0.271091	-0.959365	-1.343839
H	1.598211	-0.172879	-2.734268
H	1.919779	2.264686	-3.143872
H	0.357644	3.908717	-2.123772
H	-1.513345	3.138363	-0.718729
H	-3.647661	0.526897	2.767836
H	-2.909628	-0.274842	4.981166
H	-0.669745	-1.330165	5.249697
H	0.826560	-1.590199	3.261711
H	0.117606	-0.783891	1.073083
C	-3.629391	1.742500	0.298638
C	-3.362609	2.831178	1.148503
C	-4.836563	1.686719	-0.414704
C	-4.299139	3.857055	1.276127
H	-2.434180	2.876110	1.710820
C	-5.769025	2.718484	-0.279218
H	-5.057311	0.846624	-1.065498
C	-5.501892	3.801996	0.562580
H	-4.089516	4.695813	1.934202
H	-6.704383	2.670676	-0.829949
H	-6.230896	4.601359	0.666578
F	2.786950	4.269897	-0.221351
F	4.947777	4.093189	-0.020843
F	3.993784	3.625421	-1.920322

Additive **7b** (add7b_012_dcm)

C	-1.384127	1.968369	-1.318485
P	0.002524	1.463951	-0.258851
C	-0.626196	1.346400	1.443806
C	-1.853386	0.694020	1.655071
C	-2.319922	0.496391	2.955412

C	-1.568964	0.947804	4.046584
C	-0.346669	1.594339	3.837387
C	0.128863	1.792302	2.539275
C	1.260365	2.782851	-0.309520
C	0.983791	4.066442	0.189338
C	1.955586	5.065698	0.119270
C	3.204888	4.791137	-0.450775
C	3.479421	3.515940	-0.953686
C	2.509529	2.511516	-0.884449
N	0.591656	0.099890	-0.858865
P	1.349451	-1.186737	-0.278732
C	2.781280	-1.538165	-1.346727
C	3.880034	-2.275263	-0.877607
C	4.946438	-2.557866	-1.734941
C	4.920367	-2.108687	-3.059470
C	3.825990	-1.373482	-3.528203
C	2.757057	-1.087650	-2.676103
C	0.240385	-2.626296	-0.317325
C	-1.136595	-2.423322	-0.482367
C	-2.007227	-3.517174	-0.474216
C	-1.504187	-4.810300	-0.305917
C	-0.127790	-5.014648	-0.144623
C	0.745677	-3.926397	-0.148826
C	1.970081	-1.008834	1.423317
C	3.066115	-0.161115	1.660343
C	3.507462	0.059401	2.965120
C	2.856810	-0.560606	4.038597
C	1.763070	-1.398945	3.805289
C	1.317023	-1.625193	2.500537
C	-1.546434	1.370403	-2.575991
C	-2.598283	1.769448	-3.404015
C	-3.488130	2.760860	-2.979708
C	-3.327415	3.356455	-1.723586
C	-2.277526	2.963676	-0.892108
H	-2.447297	0.342642	0.816977
H	-3.268985	-0.009425	3.107016
H	-1.934868	0.793201	5.058580
H	0.243673	1.937081	4.682977
H	1.085387	2.281911	2.388513
H	0.017394	4.285191	0.635506
H	1.738727	6.056348	0.510159
H	3.960097	5.571255	-0.503979
H	4.446370	3.299888	-1.400783
H	2.721205	1.523127	-1.279916

H	3.913597	-2.618632	0.152447
H	5.797354	-3.124376	-1.365826
H	5.752417	-2.328028	-3.723698
H	3.804308	-1.019740	-4.555640
H	1.911249	-0.510209	-3.037159
H	-1.534033	-1.423100	-0.615722
H	-3.073134	-3.350282	-0.597141
H	-2.182535	-5.659823	-0.301255
H	0.264686	-6.020119	-0.016374
H	1.812186	-4.092409	-0.020886
H	3.572486	0.324375	0.830925
H	4.356146	0.714462	3.143576
H	3.200104	-0.386374	5.055149
H	1.249607	-1.873323	4.637141
H	0.460134	-2.269520	2.329342
H	-0.865744	0.587263	-2.894848
H	-2.729254	1.295497	-4.373248
H	-4.312869	3.061447	-3.620781
H	-4.025873	4.117210	-1.385376
H	-2.170077	3.417782	0.089205
F	-3.857446	-0.533923	-0.859610
F	-5.308238	-2.324666	-0.712652
F	-6.028648	-0.202328	-0.146001
F	-4.523157	-1.312245	1.209772
B	-4.930111	-1.091873	-0.127367

Additive **7c** (add7c_054_dcm)

N	-0.790810	-0.100644	0.669323
C	-0.251542	-0.350001	2.076798
C	1.259018	-0.516906	2.204044
C	-2.307787	-0.000930	0.808902
C	-3.088323	0.255126	-0.475964
C	-0.203523	1.179504	0.070213
C	-0.517103	2.476552	0.808845
C	-0.393532	-1.231001	-0.281986
C	-0.812790	-2.637669	0.128929
H	-0.763660	-1.243931	2.437986
H	-0.594395	0.489875	2.683821
H	1.606930	-1.366946	1.606890
H	1.782191	0.372648	1.836822
H	-2.490394	0.796945	1.530858
H	-2.631074	-0.935563	1.270467
H	-2.748775	1.178569	-0.958774
H	-2.935525	-0.561613	-1.190903

H	-0.570950	1.226220	-0.955881
H	0.872049	1.018064	0.013875
H	-1.597407	2.663318	0.825598
H	-0.175994	2.424806	1.849689
H	0.688879	-1.167086	-0.389251
H	-0.824168	-0.971301	-1.249552
H	-1.899946	-2.705131	0.255463
H	-0.351876	-2.916002	1.083879
C	-0.372995	-3.644351	-0.946940
H	-0.834382	-3.374522	-1.907096
H	0.713495	-3.570342	-1.091443
C	-0.748176	-5.083527	-0.580961
H	-0.429824	-5.785176	-1.361750
H	-0.271027	-5.389618	0.359472
H	-1.834033	-5.190478	-0.456118
C	1.637939	-0.749426	3.675282
H	1.289014	0.098930	4.281041
H	1.114647	-1.640190	4.050900
C	3.148381	-0.925381	3.860442
H	3.693974	-0.036030	3.519264
H	3.400745	-1.091474	4.915110
H	3.518880	-1.784361	3.286011
C	0.180637	3.656802	0.112966
H	-0.139112	3.700833	-0.937263
H	1.264679	3.482806	0.100038
C	-0.120753	4.992369	0.800109
H	0.383144	5.821376	0.287917
H	-1.198539	5.203556	0.800264
H	0.219810	4.986030	1.843992
C	-4.589164	0.373403	-0.166041
H	-4.750143	1.192630	0.548945
H	-4.933108	-0.546472	0.327734
C	-5.421606	0.621283	-1.427748
H	-6.488785	0.703736	-1.187607
H	-5.116272	1.550826	-1.925830
H	-5.301464	-0.198769	-2.147782
F	1.515650	-0.141030	-2.923909
F	3.807692	0.114510	-3.071267
F	2.826596	-0.350924	-1.032712
F	2.529764	1.720703	-2.009051
B	2.671735	0.336787	-2.262187

Additive **7d** (add7d_062_dcm)

P	-0.735614	-0.106640	0.655107

C	-2.532882	-0.006792	0.952013
C	-3.368242	0.269671	-0.307816
C	-0.132096	1.444803	-0.082801
C	0.066854	-0.398102	2.267067
C	-0.367858	-1.472308	-0.492658
C	1.593418	-0.563919	2.200333
C	-0.771132	-2.862008	0.023904
C	-0.434451	2.705766	0.742193
H	-2.684606	0.779335	1.702331
H	-2.827051	-0.955957	1.416838
H	-3.049543	1.215341	-0.765878
H	-3.193195	-0.518843	-1.051685
H	-0.578946	1.511561	-1.081850
H	0.945792	1.321354	-0.230121
H	-0.407652	-1.290853	2.693869
H	-0.207660	0.448969	2.908146
H	0.705763	-1.421511	-0.703555
H	-0.885412	-1.236028	-1.430548
H	1.848885	-1.407746	1.546926
H	2.046702	0.328762	1.750750
H	-0.247188	-3.076422	0.965081
H	-1.846982	-2.884270	0.245063
H	-1.518808	2.814753	0.879128
H	0.006371	2.611678	1.743763
C	2.197552	-0.796954	3.591579
H	1.941255	0.050667	4.243316
H	1.736553	-1.688457	4.040951
C	3.718834	-0.970524	3.544146
H	4.204264	-0.081447	3.120782
H	4.131725	-1.133396	4.547598
H	3.998594	-1.830395	2.921503
C	-0.441202	-3.957734	-0.999018
H	-0.963758	-3.739563	-1.941112
H	0.633882	-3.926794	-1.224110
C	-0.827129	-5.355245	-0.504748
H	-0.584528	-6.121246	-1.251755
H	-0.292612	-5.608950	0.420460
H	-1.903867	-5.418711	-0.297375
C	-4.867667	0.341108	0.012126
H	-5.039499	1.131043	0.757239
H	-5.182408	-0.603375	0.478459
C	-5.718952	0.610879	-1.232401
H	-6.785824	0.658526	-0.981022
H	-5.441906	1.564104	-1.701763

H	-5.585788	-0.181156	-1.981193
C	0.119562	3.965117	0.061457
H	1.203135	3.851195	-0.078338
H	-0.316026	4.051533	-0.943937
C	-0.169903	5.238278	0.862322
H	0.235822	6.124340	0.358419
H	-1.250326	5.390234	0.988946
H	0.280132	5.186901	1.862853
F	2.805185	-0.425022	-1.241123
F	3.694167	0.098092	-3.308724
F	2.775806	1.758679	-1.989513
F	1.408964	0.180847	-2.979168
B	2.672720	0.403766	-2.382845

Cationic sandwich complex **b3b** (sw_b3b_013a_dcm_sp)

C	0.437155	3.513786	-1.453749
C	0.594818	3.896761	-2.777269
N	0.258438	3.148025	-3.839484
C	-0.265108	1.941644	-3.558552
C	-0.465348	1.460950	-2.278302
C	-0.116079	2.250411	-1.162385
N	-0.390995	1.692875	0.074008
S	0.008485	2.370476	1.454306
O	-0.229606	1.394356	2.539694
O	1.265771	3.156029	1.510066
C	-1.329491	3.680958	1.823878
F	-2.547108	3.113279	1.887745
F	-1.085032	4.278740	3.005035
F	-1.363288	4.634290	0.872877
H	0.749292	4.188948	-0.666177
H	1.020713	4.876747	-2.995100
H	-0.540375	1.321662	-4.412361
H	-0.896759	0.475962	-2.121345
C	-2.253036	-2.437794	0.587825
P	-3.717837	-1.872915	-0.320998
C	-5.089821	-2.946420	0.184574
C	-5.255691	-3.220281	1.547759
C	-6.340446	-3.977408	1.973064
C	-7.261293	-4.460521	1.045082
C	-7.100359	-4.182725	-0.309693
C	-6.017202	-3.424975	-0.744386
C	-3.422732	-2.142776	-2.095073
C	-3.150402	-3.421051	-2.597619
C	-2.920656	-3.593209	-3.957369

C	-2.953279	-2.496417	-4.818296
C	-3.219727	-1.225430	-4.318504
C	-3.456724	-1.045481	-2.957906
N	-3.909076	-0.312348	-0.032908
P	-5.145514	0.653352	0.281696
C	-4.941476	2.171029	-0.690151
C	-6.026666	3.011010	-0.962008
C	-5.824862	4.184721	-1.680887
C	-4.549304	4.520068	-2.130547
C	-3.470100	3.682089	-1.860362
C	-3.661858	2.509457	-1.136901
C	-5.134450	1.113097	2.037885
C	-4.264640	0.453428	2.907671
C	-4.218047	0.820129	4.249853
C	-5.037990	1.840316	4.722735
C	-5.911106	2.495666	3.854920
C	-5.960788	2.137589	2.513070
C	-6.784657	-0.010045	-0.118122
C	-7.157763	-0.107186	-1.464371
C	-8.384667	-0.664940	-1.801866
C	-9.237950	-1.131366	-0.803078
C	-8.862766	-1.044268	0.534764
C	-7.636914	-0.484102	0.882067
C	-1.362226	-1.489195	1.094232
C	-0.218349	-1.908373	1.767306
C	0.042871	-3.266103	1.927568
C	-0.846640	-4.213062	1.420319
C	-1.996729	-3.803984	0.753973
H	-4.538509	-2.846661	2.274728
H	-6.465515	-4.192519	3.030361
H	-8.107967	-5.053366	1.380134
H	-7.821041	-4.553772	-1.032805
H	-5.898981	-3.207985	-1.801966
H	-3.127554	-4.283373	-1.936709
H	-2.714163	-4.586262	-4.346153
H	-2.770099	-2.635605	-5.880224
H	-3.245299	-0.368454	-4.985599
H	-3.665294	-0.053946	-2.566324
H	-7.027063	2.748859	-0.628464
H	-6.668251	4.834448	-1.896046
H	-4.397507	5.435043	-2.696563
H	-2.474090	3.938957	-2.211448
H	-2.820706	1.859626	-0.917093
H	-3.614849	-0.332616	2.534073

H	-3.533671	0.310901	4.922304
H	-4.996648	2.129498	5.769244
H	-6.550538	3.292669	4.223253
H	-6.640124	2.657276	1.842038
H	-6.494179	0.254902	-2.245998
H	-8.676151	-0.734506	-2.845926
H	-10.197348	-1.566243	-1.069616
H	-9.524577	-1.413253	1.313101
H	-7.347694	-0.418029	1.927048
H	-1.545562	-0.427785	0.959032
H	0.462222	-1.161169	2.163512
H	0.936752	-3.589261	2.455123
H	-0.651085	-5.273338	1.553147
H	-2.695884	-4.547278	0.380424
C	3.880830	-0.993993	2.834096
P	4.958530	-0.696142	1.407590
C	5.554791	-2.309986	0.832900
C	4.598462	-3.281731	0.511074
C	5.005057	-4.507483	-0.000397
C	6.361129	-4.768162	-0.193267
C	7.311045	-3.801356	0.123091
C	6.912430	-2.569403	0.634713
C	6.385495	0.242117	2.029386
C	7.149339	-0.256078	3.091851
C	8.227574	0.478125	3.570001
C	8.543692	1.710624	2.998979
C	7.778592	2.210236	1.949723
C	6.697899	1.478728	1.463702
N	4.118829	0.134118	0.324701
P	4.192539	0.253545	-1.274543
C	4.196897	2.015768	-1.710409
C	4.523425	2.432042	-3.006671
C	4.537562	3.787981	-3.312272
C	4.236753	4.730145	-2.329879
C	3.911080	4.315535	-1.042136
C	3.884760	2.959885	-0.729160
C	2.752359	-0.569391	-2.015530
C	1.860981	-1.254608	-1.188170
C	0.805354	-1.968315	-1.749800
C	0.632950	-1.990071	-3.130077
C	1.509924	-1.287837	-3.955244
C	2.570383	-0.579385	-3.403660
C	5.662127	-0.479232	-2.052568
C	6.878176	0.211299	-1.985014

C	8.033301	-0.370105	-2.494774
C	7.980740	-1.636500	-3.073829
C	6.771786	-2.323997	-3.141738
C	5.611692	-1.751271	-2.630524
C	2.882504	-0.062971	3.136335
C	2.098116	-0.240171	4.271447
C	2.308335	-1.336761	5.104020
C	3.303555	-2.263775	4.802525
C	4.092710	-2.096031	3.669185
H	3.540090	-3.080290	0.659497
H	4.262878	-5.261399	-0.246809
H	6.676729	-5.727959	-0.592947
H	8.367265	-4.001181	-0.033146
H	7.657847	-1.815452	0.869260
H	6.908521	-1.214751	3.544237
H	8.821373	0.088979	4.392036
H	9.385264	2.283455	3.378606
H	8.017935	3.173164	1.507638
H	6.096634	1.874391	0.651020
H	4.786440	1.706986	-3.771775
H	4.793404	4.109338	-4.317777
H	4.256165	5.789416	-2.571339
H	3.669727	5.046522	-0.275637
H	3.618888	2.638697	0.272732
H	1.995034	-1.233889	-0.110590
H	0.119529	-2.508881	-1.104011
H	-0.189558	-2.551324	-3.565680
H	1.372202	-1.296530	-5.032723
H	3.260048	-0.053790	-4.058292
H	6.923073	1.202852	-1.543030
H	8.974512	0.169773	-2.444539
H	8.884397	-2.087217	-3.474858
H	6.729541	-3.312332	-3.590487
H	4.673829	-2.296198	-2.681733
H	2.706285	0.788899	2.484792
H	1.312519	0.476154	4.490257
H	1.691635	-1.472003	5.988416
H	3.464229	-3.123423	5.446838
H	4.863813	-2.825057	3.435804

Cationic sandwich complex **c3c** (sw_c3c_052_dcm_sp)

C	-1.738697	-2.584605	-0.147975
C	-2.631821	-3.171378	-1.031288
N	-2.574803	-3.061992	-2.367852

C	-1.552917	-2.333159	-2.848778
C	-0.607675	-1.703735	-2.059677
C	-0.680827	-1.800150	-0.653833
N	0.293778	-1.111646	0.047705
S	0.339620	-1.027047	1.634307
O	1.328344	0.002378	2.020642
O	-0.946463	-1.049901	2.374596
C	1.187792	-2.631614	2.218707
F	2.449832	-2.698687	1.757891
F	1.229277	-2.684677	3.561633
F	0.523796	-3.715181	1.774039
H	-1.879002	-2.724963	0.917174
H	-3.451635	-3.769589	-0.631875
H	-1.490516	-2.243006	-3.933604
H	0.190372	-1.123825	-2.516593
N	4.385154	0.650896	-0.706353
C	4.280215	1.223258	0.701376
C	4.855685	2.614370	0.902066
C	5.828132	0.613519	-1.187345
C	3.787823	-0.749798	-0.630035
C	3.624445	1.513286	-1.703551
C	3.710888	-1.520107	-1.937145
C	2.186769	1.833424	-1.330299
C	6.765930	-0.289976	-0.405264
H	4.775820	0.506316	1.361852
H	3.215785	1.204805	0.950111
H	5.948058	2.603554	0.804302
H	4.464088	3.317948	0.157494
H	6.180169	1.648484	-1.158338
H	5.793351	0.308909	-2.237342
H	2.788920	-0.634365	-0.201204
H	4.397059	-1.291246	0.098690
H	3.666467	0.976030	-2.654663
H	4.203786	2.433054	-1.824496
H	3.012936	-1.037244	-2.631903
H	4.689384	-1.569136	-2.431006
H	1.648303	0.936264	-1.000795
H	2.160719	2.551213	-0.501088
H	6.735582	-0.055863	0.665616
H	6.477663	-1.341388	-0.522060
C	3.218077	-2.945459	-1.663500
C	3.065258	-3.752833	-2.948884
H	2.255033	-2.899954	-1.137657
H	3.922429	-3.450364	-0.988720

H	2.712755	-4.767839	-2.737191
H	2.342754	-3.282746	-3.626712
H	4.020518	-3.833054	-3.481072
C	4.483944	3.118752	2.301259
C	5.061502	4.501807	2.585775
H	4.843856	2.404185	3.053919
H	3.389403	3.146620	2.394264
H	4.781648	4.848541	3.586164
H	6.156206	4.491863	2.528486
H	4.694936	5.238416	1.861141
C	8.200036	-0.108454	-0.913902
C	9.182039	-1.028473	-0.194653
H	8.503007	0.938253	-0.776811
H	8.230978	-0.306573	-1.993925
H	10.202891	-0.881123	-0.562589
H	9.183567	-0.836653	0.884703
H	8.919063	-2.081565	-0.347641
C	1.456270	2.434504	-2.534477
C	0.028446	2.840443	-2.182480
H	1.440917	1.698824	-3.350270
H	2.010053	3.307031	-2.907347
H	-0.498192	3.237561	-3.056790
H	-0.536885	1.978588	-1.808052
H	0.018280	3.612937	-1.404170
N	-4.244321	1.035114	0.167144
C	-3.636013	2.310600	0.733914
C	-2.174115	2.227380	1.139412
C	-5.721381	1.328179	-0.056268
C	-6.529304	0.216466	-0.703068
C	-3.562558	0.631694	-1.135698
C	-3.665309	1.631151	-2.275602
C	-4.051484	-0.133840	1.125389
C	-4.627807	0.042419	2.519441
H	-4.254867	2.584639	1.593472
H	-3.780298	3.078172	-0.031547
H	-2.035212	1.521290	1.966075
H	-1.552923	1.877681	0.306448
H	-5.763845	2.236252	-0.663877
H	-6.132339	1.577937	0.925341
H	-6.150967	-0.009946	-1.707284
H	-6.470685	-0.706023	-0.113145
H	-4.007331	-0.325404	-1.423941
H	-2.516325	0.436605	-0.885357
H	-4.710419	1.896704	-2.473945

H	-3.129263	2.557123	-2.033608
H	-2.973040	-0.306834	1.179096
H	-4.496742	-1.003236	0.634553
H	-5.703503	0.251944	2.482667
H	-4.144082	0.879107	3.038024
C	-4.405541	-1.241817	3.326938
H	-4.892416	-2.080197	2.810200
H	-3.332671	-1.469004	3.357417
C	-4.950528	-1.125077	4.747130
H	-4.792467	-2.052746	5.307469
H	-4.453647	-0.315684	5.294680
H	-6.026799	-0.915755	4.742751
C	-1.678480	3.610816	1.573243
H	-1.800418	4.317245	0.740811
H	-2.305018	3.982709	2.395402
C	-0.217592	3.578701	2.013219
H	0.425044	3.206711	1.206344
H	0.135075	4.578113	2.290360
H	-0.080074	2.921179	2.879177
C	-3.068296	1.029077	-3.551659
H	-3.588329	0.090155	-3.785345
H	-2.016869	0.766634	-3.376231
C	-3.173211	1.984882	-4.736145
H	-2.729985	1.547302	-5.637007
H	-4.220122	2.221777	-4.959469
H	-2.654330	2.928722	-4.531437
C	-7.996834	0.643282	-0.811958
H	-8.062255	1.576690	-1.387398
H	-8.386788	0.863875	0.190860
C	-8.855823	-0.430135	-1.473556
H	-9.901982	-0.113006	-1.539139
H	-8.506191	-0.644274	-2.490233
H	-8.824446	-1.366558	-0.904522

Cationic sandwich complex **d3d** (sw_d3d_025a_dcm_sp)

C	1.724575	-3.123238	0.171968
C	2.301515	-3.992042	1.086704
N	1.848720	-4.210749	2.330964
C	0.747984	-3.522818	2.679255
C	0.092987	-2.632162	1.849026
C	0.573345	-2.395492	0.542831
N	-0.146697	-1.479211	-0.204182
S	0.179271	-1.127444	-1.721150
O	-0.642748	0.036140	-2.116717

O	1.596002	-1.142720	-2.160203
C	-0.590301	-2.537936	-2.747534
F	-1.921590	-2.593871	-2.562200
F	-0.358321	-2.349612	-4.058998
F	-0.069106	-3.727945	-2.393941
H	2.168197	-3.014406	-0.810647
H	3.189736	-4.552865	0.793876
H	0.366778	-3.695844	3.686057
H	-0.795607	-2.110958	2.195772
P	-4.152393	0.391733	0.546668
C	-5.416073	1.507589	1.229712
C	-5.120822	2.995115	1.015245
C	-2.517404	0.796658	1.223909
C	-4.610657	-1.313940	0.977264
C	-4.039100	0.573414	-1.256373
C	-3.735600	-2.366486	0.287492
C	-5.336310	0.261414	-2.006449
C	-2.368341	0.584787	2.731871
H	-5.502789	1.272817	2.299051
H	-6.369092	1.224591	0.762688
H	-4.178105	3.263234	1.510185
H	-4.989195	3.201718	-0.055170
H	-2.333842	1.846729	0.959306
H	-1.788479	0.189795	0.666521
H	-5.670065	-1.444565	0.721485
H	-4.535719	-1.389672	2.070361
H	-3.223582	-0.088074	-1.579851
H	-3.702468	1.600094	-1.454464
H	-3.973142	-2.387174	-0.783486
H	-2.674206	-2.095788	0.367455
H	-5.694785	-0.740741	-1.736186
H	-6.120280	0.971250	-1.711675
H	-3.126228	1.163936	3.276687
H	-2.535315	-0.471770	2.978776
C	-6.245466	3.874729	1.564239
H	-7.186216	3.611760	1.062018
H	-6.386709	3.654511	2.631016
C	-5.956222	5.361145	1.376292
H	-5.837082	5.608708	0.314884
H	-6.769907	5.977010	1.773918
H	-5.033656	5.651325	1.892988
C	-0.974926	0.998172	3.208522
H	-0.223367	0.428438	2.643735
H	-0.811039	2.057585	2.967690

C	-0.783090	0.771025	4.704514
H	0.216726	1.078765	5.028986
H	-1.513879	1.345279	5.286463
H	-0.908240	-0.287409	4.961815
C	-5.129690	0.329042	-3.520566
H	-4.342952	-0.383034	-3.805664
H	-4.760406	1.327913	-3.789899
C	-6.409303	0.023178	-4.293121
H	-7.201747	0.739215	-4.045144
H	-6.240954	0.072753	-5.374237
H	-6.780624	-0.981480	-4.058575
C	-3.940513	-3.759840	0.881566
H	-3.685977	-3.734636	1.950202
H	-5.002832	-4.032704	0.817136
C	-3.089699	-4.810759	0.173672
H	-3.227957	-5.801068	0.620989
H	-3.352938	-4.883281	-0.888132
H	-2.023282	-4.561077	0.238901
P	3.859426	1.480125	0.170592
C	3.989124	1.374133	1.980930
C	3.249813	0.179787	2.590710
C	2.142646	1.800391	-0.329779
C	4.938889	2.834560	-0.382901
C	4.369240	-0.092262	-0.581405
C	4.900574	3.090109	-1.892653
C	5.781334	-0.547243	-0.202949
C	1.556676	3.120759	0.177279
H	3.607683	2.322340	2.383006
H	5.060433	1.334799	2.219548
H	2.185611	0.218816	2.322118
H	3.649224	-0.755609	2.177068
H	1.547285	0.949808	0.028435
H	2.125649	1.753846	-1.426626
H	5.955134	2.581419	-0.052405
H	4.635333	3.729790	0.176497
H	4.268168	0.027380	-1.667551
H	3.619801	-0.836759	-0.281272
H	5.198434	2.182318	-2.433253
H	3.875619	3.326617	-2.207035
H	6.517449	0.203719	-0.518887
H	5.866123	-0.640861	0.887715
H	1.518256	3.111012	1.274710
H	2.198261	3.962640	-0.114168
C	5.827994	4.240827	-2.287315

H	5.527765	5.146918	-1.743769
H	6.851217	4.005311	-1.964772
C	5.807473	4.508017	-3.789561
H	4.799069	4.771658	-4.129728
H	6.476442	5.333641	-4.054783
H	6.129037	3.623485	-4.351979
C	6.116575	-1.891670	-0.851419
H	5.368046	-2.633810	-0.541458
H	6.030052	-1.796263	-1.942175
C	7.514188	-2.379715	-0.482278
H	7.735788	-3.342303	-0.955363
H	8.279560	-1.664097	-0.805206
H	7.612840	-2.509094	0.601965
C	3.387726	0.156244	4.113812
H	2.954743	1.076644	4.528922
H	4.453938	0.163479	4.378117
C	2.710834	-1.063257	4.731078
H	2.793444	-1.055130	5.823390
H	1.645406	-1.093211	4.475853
H	3.166115	-1.991441	4.365661
C	0.149211	3.333725	-0.383052
H	0.209901	3.429296	-1.475587
H	-0.448311	2.434416	-0.187874
C	-0.544278	4.557269	0.207248
H	-1.547509	4.684256	-0.215064
H	-0.649248	4.467833	1.295307
H	0.022158	5.473352	0.001748

Cationic sandwich complex **a5a** (sw_a5a_002_dcm_sp)

C	-3.145264	-2.754961	1.981760
C	-4.451142	-3.039811	1.638020
N	-4.814004	-3.837675	0.613465
C	-3.797778	-4.383219	-0.075653
C	-2.452969	-4.171513	0.184301
C	-2.068319	-3.311879	1.244156
N	-0.808428	-2.935684	1.622121
S	0.501268	-3.528920	0.871083
O	1.669488	-2.943269	1.582588
O	0.517264	-5.006441	0.694270
C	0.548889	-2.820918	-0.797003
C	0.556285	-3.640878	-1.918483
C	0.628821	-3.065448	-3.186568
C	0.692666	-1.679967	-3.347660
C	0.672881	-0.874460	-2.201481

C	0.605058	-1.434798	-0.934229
C	0.810314	-1.057716	-4.709796
H	-2.932181	-2.093111	2.817607
H	-5.263507	-2.596366	2.214834
H	-4.076793	-5.039217	-0.901692
H	-1.713458	-4.674213	-0.428788
H	0.513053	-4.719486	-1.801163
H	0.637361	-3.708753	-4.063595
H	0.716473	0.207446	-2.305748
H	0.606533	-0.797644	-0.054548
H	0.195175	-0.155113	-4.787444
H	1.847979	-0.758845	-4.908207
H	0.509969	-1.755157	-5.496945
P	-3.897711	1.401170	-0.003640
C	-5.286857	0.567069	0.796121
C	-3.422109	0.449876	-1.467530
C	-2.523382	1.530728	1.167021
C	-6.238734	-0.102096	0.018316
C	-7.335561	-0.690273	0.637143
C	-7.485717	-0.609201	2.019511
C	-6.536696	0.057561	2.790695
C	-5.432664	0.646150	2.184624
C	-3.353962	-0.947017	-1.385078
C	-3.001616	-1.682455	-2.510159
C	-2.724352	-1.035909	-3.712211
C	-2.793987	0.352061	-3.792698
C	-3.142394	1.100428	-2.673497
C	-2.387274	2.670203	1.969046
C	-1.359424	2.730705	2.903989
C	-0.480100	1.660009	3.048774
C	-0.628735	0.521180	2.261588
C	-1.645206	0.453041	1.316204
H	-6.126639	-0.165652	-1.060161
H	-8.071605	-1.215965	0.036378
H	-8.342732	-1.074228	2.498607
H	-6.649431	0.112657	3.869358
H	-4.689510	1.155091	2.791476
H	-3.585903	-1.458880	-0.454845
H	-2.946826	-2.764419	-2.442165
H	-2.455958	-1.617207	-4.589944
H	-2.586967	0.858015	-4.731095
H	-3.211873	2.180691	-2.751067
H	-3.076240	3.503815	1.870954
H	-1.250430	3.616770	3.522498

H	0.322463	1.711392	3.779896
H	0.041858	-0.325823	2.376168
H	-1.745363	-0.442408	0.710865
C	-4.387478	3.068839	-0.502650
C	-3.401682	3.998212	-0.860094
C	-5.739892	3.414779	-0.564821
C	-3.776566	5.267424	-1.281966
H	-2.348775	3.734864	-0.807264
C	-6.102771	4.689719	-0.987529
H	-6.507090	2.700707	-0.281156
C	-5.125204	5.613327	-1.345542
H	-3.012991	5.988475	-1.558297
H	-7.153459	4.960420	-1.031173
H	-5.413587	6.608508	-1.671970
P	4.442900	0.740081	0.235838
C	4.094159	0.718310	2.008211
C	4.560182	-0.968285	-0.343085
C	3.120413	1.624394	-0.628073
C	5.993741	1.613208	-0.084083
C	4.100149	-1.314168	-1.616651
C	4.216835	-2.628567	-2.054589
C	4.786745	-3.593270	-1.228262
C	5.253627	-3.245071	0.037001
C	5.142770	-1.934911	0.486403
C	1.911113	1.881071	0.022055
C	0.898485	2.558498	-0.649385
C	1.091839	2.980537	-1.961747
C	2.300376	2.726708	-2.608726
C	3.319763	2.052650	-1.947224
C	5.997686	3.013396	-0.113588
C	7.195362	3.690382	-0.309113
C	8.381363	2.978971	-0.478767
C	8.373537	1.586913	-0.453870
C	7.181769	0.897581	-0.256557
C	4.708796	1.635891	2.864788
C	4.388753	1.626877	4.218424
C	3.464346	0.710550	4.713287
C	2.854648	-0.203245	3.855952
C	3.164280	-0.205765	2.500896
H	3.638872	-0.572992	-2.260595
H	3.849782	-2.898920	-3.040026
H	4.864725	-4.621552	-1.569865
H	5.696308	-3.996959	0.683374
H	5.499495	-1.672012	1.478254

H	1.755793	1.563361	1.047925
H	-0.040809	2.754737	-0.139169
H	0.300860	3.511579	-2.484333
H	2.453886	3.060039	-3.630829
H	4.265459	1.871557	-2.451285
H	5.073814	3.570895	0.014046
H	7.200239	4.776060	-0.332997
H	9.314634	3.512467	-0.635634
H	9.296500	1.032031	-0.593050
H	7.181194	-0.188381	-0.245476
H	5.437671	2.346587	2.487124
H	4.869896	2.334207	4.887337
H	3.222341	0.704303	5.772501
H	2.137924	-0.923246	4.240561
H	2.689842	-0.933654	1.847004

Cationic sandwich complex **a6a** (sw_a6a_016a_dcm_sp)

C	2.777401	0.118513	3.364564
C	1.615656	0.771855	3.720787
N	0.391757	0.206028	3.718364
C	0.358120	-1.089146	3.363495
C	1.459726	-1.843583	2.994086
C	2.743709	-1.242311	2.963586
N	3.933541	-1.793441	2.575914
S	4.037619	-3.353454	2.152269
O	5.474237	-3.620728	1.905070
O	3.323894	-4.304169	3.046096
C	3.250204	-3.524906	0.518447
C	2.186700	-4.401964	0.338702
C	1.648493	-4.572141	-0.933061
C	2.175021	-3.856422	-2.005655
C	3.234079	-2.966449	-1.819909
C	3.773379	-2.801278	-0.551705
C	1.629174	-4.093164	-3.382561
H	3.728737	0.644280	3.380651
H	1.661297	1.819056	4.020244
H	-0.625791	-1.562380	3.370824
H	1.321249	-2.887795	2.737677
H	1.795939	-4.961798	1.182346
H	0.828689	-5.267725	-1.084400
H	3.640663	-2.410188	-2.658581
H	4.607575	-2.122947	-0.399217
P	2.271471	2.518948	-0.558160
C	1.518374	3.210018	0.933103

C	1.372753	1.018140	-1.014616
C	4.015835	2.158625	-0.249288
C	0.142980	3.071627	1.149712
C	-0.433696	3.653270	2.273131
C	0.351662	4.373183	3.170126
C	1.719646	4.513638	2.948419
C	2.309745	3.930308	1.833200
C	0.994305	0.116719	-0.011674
C	0.305707	-1.039339	-0.355812
C	-0.015693	-1.293973	-1.686658
C	0.364557	-0.399201	-2.682477
C	1.059666	0.758951	-2.352479
C	4.967150	3.173221	-0.415074
C	6.300790	2.914579	-0.122074
C	6.684828	1.655804	0.335029
C	5.735768	0.651402	0.505562
C	4.398161	0.896381	0.214172
H	-0.475487	2.516079	0.450644
H	-1.499968	3.540618	2.446851
H	-0.102993	4.821344	4.049112
H	2.333731	5.068384	3.651510
H	3.379618	4.028371	1.673705
H	1.229951	0.314939	1.030302
H	0.022360	-1.740285	0.422569
H	-0.563343	-2.195171	-1.945234
H	0.111882	-0.595039	-3.720445
H	1.337996	1.458151	-3.134681
H	4.672374	4.155704	-0.772512
H	7.040386	3.698798	-0.253790
H	7.728853	1.456867	0.560417
H	6.032521	-0.327196	0.871087
H	3.666776	0.105934	0.352553
C	2.166595	3.721193	-1.903347
C	2.960388	3.548545	-3.044596
C	1.270125	4.789402	-1.818849
C	2.844392	4.444681	-4.099854
H	3.665567	2.724023	-3.107927
C	1.164378	5.680888	-2.881759
H	0.663626	4.930958	-0.929192
C	1.947625	5.508557	-4.019492
H	3.459359	4.313209	-4.985130
H	0.472178	6.515110	-2.815247
H	1.863839	6.208811	-4.845816
F	1.819357	-3.038466	-4.208659

F	2.219966	-5.163869	-3.982157
F	0.297268	-4.352456	-3.372685
P	-4.329170	-0.112431	0.129056
C	-3.464125	1.341634	-0.512784
C	-4.296886	-0.028399	1.932193
C	-6.038703	-0.096704	-0.460720
C	-3.584751	-1.660097	-0.441669
C	-5.464584	-0.229243	2.673177
C	-5.410786	-0.175374	4.062309
C	-4.202417	0.074395	4.706152
C	-3.039028	0.276747	3.965601
C	-3.080750	0.231448	2.577635
C	-6.618807	1.078587	-0.944813
C	-7.948014	1.066473	-1.355288
C	-8.692802	-0.107241	-1.281985
C	-8.111330	-1.278749	-0.800459
C	-6.783630	-1.280883	-0.391537
C	-3.788984	-2.059166	-1.770083
C	-3.266945	-3.268289	-2.211356
C	-2.553314	-4.083765	-1.334329
C	-2.351908	-3.686338	-0.015975
C	-2.866873	-2.475630	0.437141
C	-2.709028	1.273339	-1.686565
C	-2.151509	2.435326	-2.211164
C	-2.349787	3.657283	-1.574174
C	-3.092302	3.721528	-0.397190
C	-3.649427	2.567440	0.140201
H	-6.411089	-0.418821	2.176316
H	-6.318264	-0.326381	4.639306
H	-4.165303	0.114931	5.791234
H	-2.089767	0.467594	4.458184
H	-2.170637	0.396932	2.004401
H	-6.041311	1.995684	-1.010613
H	-8.398921	1.977479	-1.737291
H	-9.729905	-0.111530	-1.605392
H	-8.690892	-2.195528	-0.746159
H	-6.331488	-2.197523	-0.021922
H	-4.364208	-1.438748	-2.452412
H	-3.427192	-3.578825	-3.239521
H	-2.158588	-5.034775	-1.680511
H	-1.799126	-4.321919	0.669748
H	-2.718760	-2.181856	1.471695
H	-2.548481	0.325590	-2.190406
H	-1.562830	2.382574	-3.122234

H	-1.917526	4.562001	-1.992199
H	-3.237817	4.672039	0.107537
H	-4.229438	2.623000	1.057400

Anionic sandwich complex **3b3** (asw_3b3_005_dcm_sp)

C	4.049136	-3.506034	1.273295
C	3.263197	-4.500512	1.835065
N	1.961837	-4.694488	1.570303
C	1.418211	-3.845044	0.681360
C	2.108741	-2.821504	0.059916
C	3.473458	-2.608882	0.348777
N	4.069799	-1.543459	-0.306865
S	5.601623	-1.157472	-0.126237
O	5.837514	0.157869	-0.759555
O	6.246756	-1.402799	1.186227
C	6.550416	-2.332294	-1.288839
F	6.130577	-2.190957	-2.559412
F	7.872113	-2.074569	-1.255489
F	6.367483	-3.618411	-0.931100
H	5.089384	-3.425722	1.564566
H	3.715131	-5.186110	2.552909
H	0.361012	-3.990249	0.457546
H	1.598573	-2.167249	-0.641575
C	-1.135177	-0.557302	-1.842232
P	-0.389342	1.000985	-1.300783
C	-1.662718	2.287469	-1.427590
C	-3.015406	1.933725	-1.405999
C	-3.989148	2.926287	-1.341792
C	-3.618732	4.267606	-1.299579
C	-2.270399	4.621920	-1.318360
C	-1.292056	3.636935	-1.376610
C	0.918967	1.355584	-2.511970
C	0.742723	2.248719	-3.572666
C	1.760078	2.416585	-4.508766
C	2.942367	1.689589	-4.397234
C	3.110895	0.786664	-3.349183
C	2.103627	0.617352	-2.407767
N	0.152744	0.768356	0.192621
P	0.966787	1.656710	1.254710
C	1.811490	0.505433	2.369793
C	1.174810	-0.703739	2.670206
C	1.752993	-1.581069	3.580171
C	2.967644	-1.262208	4.182999
C	3.601643	-0.058812	3.884255

C	3.023739	0.831984	2.984698
C	-0.128774	2.661653	2.303910
C	0.408028	3.354943	3.395588
C	-0.424069	4.119024	4.204278
C	-1.790393	4.192064	3.932418
C	-2.324620	3.496792	2.852297
C	-1.495396	2.729674	2.037224
C	2.192838	2.795090	0.555008
C	3.384692	2.281442	0.031876
C	4.290241	3.132338	-0.590283
C	4.007689	4.492171	-0.702323
C	2.819793	5.005032	-0.186286
C	1.911323	4.160969	0.444587
C	-1.237939	-1.643280	-0.973211
C	-1.773434	-2.843229	-1.435936
C	-2.213724	-2.953935	-2.750586
C	-2.119777	-1.863486	-3.614858
C	-1.574993	-0.667447	-3.167050
H	-3.318298	0.890453	-1.424740
H	-5.038131	2.644756	-1.321921
H	-4.381491	5.039898	-1.249562
H	-1.979187	5.667865	-1.283018
H	-0.242566	3.920070	-1.373920
H	-0.181281	2.808782	-3.677643
H	1.623925	3.116293	-5.328407
H	3.733415	1.824618	-5.129919
H	4.032014	0.218567	-3.253147
H	2.252272	-0.084205	-1.590958
H	0.234277	-0.963386	2.192670
H	1.258633	-2.521696	3.805383
H	3.424119	-1.956280	4.883398
H	4.552367	0.188117	4.347833
H	3.522874	1.769915	2.759740
H	1.471861	3.300910	3.614420
H	-0.006344	4.656585	5.050740
H	-2.438155	4.789344	4.568229
H	-3.389039	3.546808	2.640372
H	-1.914436	2.182411	1.200152
H	3.609946	1.220603	0.099870
H	5.211730	2.722016	-0.991870
H	4.716443	5.154560	-1.192065
H	2.598675	6.065129	-0.271518
H	0.988113	4.567260	0.847626
H	-0.901196	-1.552012	0.055298

H	-1.855054	-3.688826	-0.760067
H	-2.636934	-3.890322	-3.103309
H	-2.467339	-1.947082	-4.640429
H	-1.492214	0.176985	-3.847757
C	-4.194023	-2.424651	1.092138
C	-3.199830	-2.831613	1.968790
N	-2.426988	-2.006944	2.693895
C	-2.681185	-0.694376	2.546115
C	-3.648390	-0.181077	1.703453
C	-4.440332	-1.045951	0.917810
N	-5.347651	-0.425200	0.079407
S	-6.274532	-1.212063	-0.946198
O	-6.880671	-0.239711	-1.878502
O	-5.774028	-2.486550	-1.515654
C	-7.779956	-1.777345	0.075265
F	-8.385797	-0.729727	0.663415
F	-8.684358	-2.395184	-0.709095
F	-7.410624	-2.640012	1.043120
H	-4.748432	-3.170474	0.535236
H	-3.007048	-3.898494	2.088184
H	-2.067230	-0.012051	3.135292
H	-3.804431	0.891217	1.630769

Anionic sandwich complex **3c3** (asw_3c3_007_dcm_sp)

C	3.220617	-1.366429	-1.893004
C	3.565620	-0.786069	-3.103932
N	4.441836	0.220065	-3.253246
C	5.018170	0.660572	-2.120832
C	4.750378	0.152582	-0.863343
C	3.812864	-0.890932	-0.703873
N	3.595104	-1.310136	0.596453
S	2.435197	-2.324470	0.996307
O	2.272938	-2.307290	2.463742
O	1.203353	-2.322375	0.167007
C	3.139479	-4.071653	0.701713
F	4.263516	-4.258915	1.416052
F	2.247055	-5.009319	1.070629
F	3.436043	-4.265725	-0.598127
H	2.488648	-2.164685	-1.880004
H	3.099086	-1.157147	-4.017185
H	5.737766	1.472836	-2.228075
H	5.252525	0.556805	0.011354
N	0.849562	2.208087	0.315860
C	2.208081	2.050411	0.980943

C	2.429120	2.843150	2.257371
C	0.634996	3.636421	-0.158916
C	0.839574	1.229234	-0.853517
C	-0.279709	1.909593	1.292774
C	-0.387207	1.270439	-1.746319
C	-0.239552	0.546647	1.959934
C	1.634903	4.156552	-1.177961
H	2.948245	2.327098	0.225423
H	2.332305	0.980419	1.172854
H	2.295811	3.918651	2.087557
H	1.716872	2.540473	3.034227
H	0.645338	4.261820	0.737861
H	-0.379853	3.670531	-0.565572
H	0.958915	0.233412	-0.416289
H	1.744755	1.440265	-1.430763
H	-1.214284	2.028349	0.736533
H	-0.241607	2.704314	2.044154
H	-1.291260	1.026708	-1.175572
H	-0.525546	2.265227	-2.187011
H	-0.361100	-0.251779	1.219406
H	0.717190	0.372847	2.466515
H	2.642860	4.202433	-0.748343
H	1.680893	3.500663	-2.055508
C	3.852311	2.591641	2.767066
C	4.130195	3.323082	4.076816
H	4.573497	2.912550	2.003274
H	4.001274	1.511966	2.905523
H	5.151787	3.136045	4.424689
H	4.009727	4.406373	3.957578
H	3.442981	2.994254	4.865096
C	-1.375825	0.458202	2.984609
C	-1.522053	-0.947011	3.559112
H	-2.314684	0.756682	2.502099
H	-1.189917	1.177134	3.794440
H	-2.338033	-0.992760	4.288786
H	-1.741972	-1.670854	2.765012
H	-0.602345	-1.270154	4.061117
C	-0.231743	0.250946	-2.880014
C	-1.466773	0.203961	-3.774357
H	-0.047991	-0.744004	-2.452994
H	0.653733	0.504898	-3.478965
H	-1.346233	-0.532026	-4.576825
H	-2.357782	-0.072489	-3.198160
H	-1.656949	1.179571	-4.237639

C	1.223741	5.561411	-1.630659
C	2.209648	6.149215	-2.635680
H	1.149858	6.220092	-0.754639
H	0.221021	5.517342	-2.076530
H	1.898264	7.148973	-2.956821
H	3.212743	6.233687	-2.201438
H	2.284253	5.518013	-3.528921
C	-4.384174	-1.867636	-1.592791
C	-4.257612	-3.056213	-2.295994
N	-3.161999	-3.831363	-2.306126
C	-2.132643	-3.392368	-1.559898
C	-2.150744	-2.225736	-0.818388
C	-3.298942	-1.405251	-0.818661
N	-3.212673	-0.251500	-0.059351
S	-4.316497	0.889769	-0.066984
O	-3.746226	2.104662	0.555914
O	-5.133395	1.072161	-1.290753
C	-5.605568	0.354020	1.229771
F	-5.028604	0.167384	2.431514
F	-6.564484	1.289692	1.368610
F	-6.198635	-0.801472	0.870597
H	-5.309336	-1.307839	-1.659930
H	-5.099743	-3.406232	-2.894295
H	-1.236191	-4.013315	-1.556938
H	-1.277833	-1.932760	-0.241931

Anionic sandwich complex **3d3** (asw_3d3_012_dcm_sp)

C	-2.462849	-2.678142	-0.557202
C	-1.381603	-3.530331	-0.393200
N	-0.778917	-3.790479	0.778095
C	-1.301885	-3.172243	1.851427
C	-2.379933	-2.307584	1.802103
C	-2.998227	-2.014878	0.567180
N	-4.051428	-1.120352	0.617816
S	-4.665367	-0.428409	-0.674522
O	-5.484867	0.726556	-0.252978
O	-3.775121	-0.241973	-1.847987
C	-5.994987	-1.625246	-1.332587
F	-6.915067	-1.880537	-0.385238
F	-6.627325	-1.098632	-2.398620
F	-5.450415	-2.798687	-1.709868
H	-2.865191	-2.521516	-1.550836
H	-0.970508	-4.035421	-1.267997
H	-0.823856	-3.378837	2.809675

H	-2.750398	-1.839427	2.709887
P	-0.414212	1.652273	0.585970
C	-2.019235	1.642950	1.434928
C	-2.169358	2.696753	2.534106
C	0.935463	1.472447	1.786830
C	-0.412229	0.274826	-0.598454
C	-0.181086	3.227650	-0.290738
C	0.751023	0.296662	-1.593930
C	-1.260359	3.518259	-1.338070
C	0.810211	0.232976	2.677663
H	-2.154801	0.632394	1.841997
H	-2.786987	1.773018	0.660875
H	-1.387429	2.561611	3.293124
H	-2.037535	3.702661	2.113741
H	0.950677	2.392267	2.387281
H	1.873691	1.446339	1.215468
H	-1.381523	0.299415	-1.115382
H	-0.399484	-0.648111	-0.002348
H	0.816320	3.186379	-0.749704
H	-0.148516	4.017141	0.471513
H	0.707183	1.211808	-2.199143
H	1.706550	0.309074	-1.054227
H	-1.322381	2.688970	-2.055137
H	-2.241218	3.589799	-0.850247
H	-0.134983	0.261864	3.236535
H	0.783224	-0.670985	2.055190
C	-3.543889	2.599363	3.199061
C	-3.728049	3.633075	4.306163
H	-3.672107	1.587608	3.607457
H	-4.322491	2.727475	2.434921
H	-4.716655	3.545273	4.769596
H	-2.976254	3.505473	5.094154
H	-3.631605	4.652503	3.913924
C	-0.972876	4.818054	-2.091108
C	-2.048040	5.133249	-3.127030
H	0.006557	4.739228	-2.582227
H	-0.897175	5.642830	-1.369428
H	-1.826166	6.064217	-3.659975
H	-2.124675	4.331505	-3.870934
H	-3.030667	5.244385	-2.653511
C	0.706641	-0.921146	-2.518523
C	1.847286	-0.922008	-3.532177
H	-0.259950	-0.942840	-3.041452
H	0.753750	-1.834570	-1.910106

H	1.784027	-1.789171	-4.198679
H	1.825610	-0.018336	-4.153247
H	2.821580	-0.958705	-3.030595
C	1.976258	0.126631	3.661411
C	1.890083	-1.136712	4.512742
H	1.986234	1.015578	4.306905
H	2.919044	0.132361	3.098822
H	2.728584	-1.201942	5.214647
H	0.961465	-1.159182	5.095997
H	1.911413	-2.034630	3.882995
C	5.231630	-2.257478	-0.024642
C	5.274448	-3.637417	-0.153161
N	4.213403	-4.453253	-0.052213
C	3.037159	-3.850039	0.194324
C	2.880421	-2.484152	0.341028
C	3.996089	-1.625737	0.232125
N	3.732832	-0.275492	0.378470
S	4.849728	0.852902	0.321077
O	4.251357	2.128323	0.771790
O	6.210426	0.526410	0.809954
C	5.143367	1.196162	-1.531428
F	4.008800	1.599894	-2.133284
F	6.066612	2.163394	-1.694016
F	5.580701	0.091383	-2.166749
H	6.147734	-1.686649	-0.118650
H	6.232869	-4.117817	-0.353457
H	2.164582	-4.498485	0.277948
H	1.896678	-2.065007	0.535732

Anionic sandwich complex **5a5** (asw_5a5_002_dcm_sp)

C	4.340492	0.307524	-1.873277
C	5.154443	1.257400	-1.291224
N	5.906848	1.049098	-0.191533
C	5.836001	-0.190509	0.322539
C	5.058341	-1.221048	-0.180534
C	4.246562	-0.998157	-1.322741
N	3.380200	-1.859491	-1.934426
S	3.200551	-3.384213	-1.398071
O	2.245190	-4.031021	-2.333484
O	4.473895	-4.116023	-1.149089
C	2.339937	-3.302542	0.195009
C	2.887830	-3.869371	1.340020
C	2.167459	-3.829772	2.532853
C	0.906768	-3.229526	2.596626

C	0.384164	-2.651081	1.433649
C	1.089635	-2.689297	0.239170
C	0.112277	-3.220191	3.871455
H	3.756752	0.555938	-2.756343
H	5.206336	2.255232	-1.728368
H	6.445884	-0.376987	1.208013
H	5.086755	-2.188630	0.307763
H	3.864345	-4.342482	1.297312
H	2.595349	-4.275257	3.428291
H	-0.588431	-2.164448	1.465094
H	0.663550	-2.252795	-0.657946
H	-0.540109	-2.343042	3.924083
H	-0.531863	-4.107528	3.928075
H	0.762401	-3.231517	4.751631
P	0.646612	2.500459	0.188481
C	2.070866	3.444157	-0.404046
C	1.198770	1.448146	1.555758
C	-0.014987	1.538873	-1.196857
C	3.032664	3.895847	0.506378
C	4.087071	4.680732	0.054610
C	4.181783	5.016121	-1.294129
C	3.222809	4.565441	-2.197894
C	2.164502	3.777851	-1.758465
C	2.418782	0.765889	1.449270
C	2.855926	-0.023887	2.504972
C	2.090582	-0.133044	3.663599
C	0.879722	0.544279	3.767299
C	0.428334	1.336296	2.716990
C	-1.135322	1.993793	-1.901039
C	-1.567784	1.297614	-3.025346
C	-0.884476	0.162199	-3.452018
C	0.235599	-0.283723	-2.754372
C	0.673391	0.398873	-1.625670
H	2.961956	3.636488	1.558897
H	4.838133	5.026299	0.758655
H	5.010653	5.625675	-1.643175
H	3.300530	4.820346	-3.250705
H	1.421860	3.422610	-2.467069
H	3.031074	0.856390	0.555396
H	3.798769	-0.554245	2.416589
H	2.440958	-0.748832	4.487226
H	0.279376	0.461361	4.668442
H	-0.515981	1.860114	2.812376
H	-1.664460	2.886830	-1.584687

H	-2.440606	1.648210	-3.568214
H	-1.226556	-0.378200	-4.330451
H	0.777087	-1.166942	-3.080872
H	1.551153	0.037597	-1.099130
C	-0.627804	3.642718	0.765686
C	-1.897342	3.142132	1.080695
C	-0.346689	5.005126	0.899326
C	-2.879787	4.014174	1.533750
H	-2.133539	2.085770	0.973915
C	-1.339728	5.866002	1.355943
H	0.634142	5.396364	0.646648
C	-2.602590	5.372536	1.673137
H	-3.865381	3.624827	1.773600
H	-1.124237	6.925468	1.458776
H	-3.375312	6.050008	2.026089
C	-5.430967	1.553223	-1.404987
C	-6.457491	1.467231	-2.323560
N	-7.259899	0.394972	-2.472268
C	-7.006107	-0.631399	-1.643773
C	-6.006315	-0.653158	-0.684617
C	-5.155250	0.470452	-0.532302
N	-4.102181	0.625815	0.328423
S	-3.729302	-0.537593	1.391602
O	-2.527660	-0.050959	2.125527
O	-4.858593	-0.999364	2.246203
C	-3.178613	-1.989771	0.458687
C	-3.398706	-3.262842	0.976226
C	-2.888920	-4.368830	0.303922
C	-2.171327	-4.223197	-0.888557
C	-1.986536	-2.934634	-1.399672
C	-2.476601	-1.818463	-0.730332
C	-1.574762	-5.417127	-1.577056
H	-4.821620	2.451931	-1.343755
H	-6.653235	2.310720	-2.986958
H	-7.650873	-1.504969	-1.750970
H	-5.897996	-1.531494	-0.057577
H	-3.962595	-3.384557	1.896451
H	-3.052702	-5.363982	0.711686
H	-1.438642	-2.801871	-2.329637
H	-2.314243	-0.821400	-1.131467
H	-0.614037	-5.680991	-1.115729
H	-2.225942	-6.293228	-1.495766
H	-1.384955	-5.214920	-2.635154

Anionic sandwich complex **6a6** (asw_6a6_040a_dcm_sp)

C	3.892592	3.814544	0.684774
C	2.649588	3.866455	1.282388
N	2.225730	3.014110	2.237770
C	3.119380	2.083628	2.611828
C	4.391194	1.938694	2.079580
C	4.829000	2.814905	1.054843
N	6.016121	2.808993	0.370332
S	7.138112	1.666219	0.625225
O	8.319093	2.051075	-0.182359
O	7.392309	1.313461	2.048519
C	6.478125	0.162688	-0.156002
C	5.990173	-0.882506	0.622723
C	5.419129	-1.987428	0.001507
C	5.343574	-2.036402	-1.389350
C	5.857887	-0.999563	-2.167659
C	6.427841	0.103922	-1.546818
C	4.641491	-3.193956	-2.031784
H	4.158538	4.530190	-0.088939
H	1.938863	4.633757	0.972432
H	2.796153	1.394566	3.393034
H	5.038305	1.162079	2.468576
H	6.065665	-0.838495	1.704048
H	5.034652	-2.806942	0.601573
H	5.807924	-1.044584	-3.250394
H	6.824887	0.920336	-2.142592
P	0.111600	-0.374014	-0.036994
C	-0.791751	-1.830745	-0.613035
C	-1.051736	0.980337	0.239139
C	1.327138	0.098802	-1.288282
C	-0.335462	-3.109877	-0.281259
C	-0.989594	-4.224373	-0.794194
C	-2.093182	-4.066240	-1.628517
C	-2.547015	-2.791081	-1.955779
C	-1.898840	-1.668484	-1.453550
C	-2.272533	0.715785	0.872934
C	-3.127255	1.768365	1.175081
C	-2.773791	3.074940	0.845695
C	-1.565523	3.333404	0.204326
C	-0.699160	2.289493	-0.101757
C	2.376129	0.958720	-0.939075
C	3.258417	1.394474	-1.919612
C	3.107630	0.965111	-3.236649
C	2.074274	0.097588	-3.578161

C	1.177455	-0.337462	-2.607694
H	0.517471	-3.242072	0.377779
H	-0.639730	-5.218377	-0.531647
H	-2.607163	-4.939926	-2.019182
H	-3.411977	-2.669170	-2.600680
H	-2.254030	-0.675560	-1.714341
H	-2.555932	-0.301449	1.131357
H	-4.073636	1.560631	1.664926
H	-3.446991	3.893587	1.084005
H	-1.293529	4.350363	-0.062634
H	0.241662	2.498103	-0.601464
H	2.499597	1.290112	0.088232
H	4.071837	2.060143	-1.646755
H	3.806807	1.301634	-3.997238
H	1.963724	-0.246417	-4.602346
H	0.373961	-1.015080	-2.879832
C	0.964520	-0.756844	1.511234
C	2.198003	-1.416911	1.473161
C	0.360298	-0.455298	2.735160
C	2.815566	-1.781434	2.663709
H	2.673530	-1.644267	0.523146
C	0.986219	-0.828961	3.918946
H	-0.590887	0.065461	2.774176
C	2.210244	-1.491470	3.884391
H	3.774372	-2.290481	2.637035
H	0.515281	-0.593707	4.868787
H	2.698705	-1.776744	4.811972
F	4.950623	-4.376059	-1.443246
F	3.284206	-3.071111	-1.939853
F	4.926038	-3.319101	-3.346776
C	-3.449052	-2.863435	1.848879
C	-2.658694	-2.529440	2.929588
N	-2.945449	-1.549497	3.810034
C	-4.094951	-0.893568	3.583294
C	-4.965746	-1.147327	2.534383
C	-4.657110	-2.164600	1.596921
N	-5.355791	-2.538264	0.479286
S	-6.734551	-1.794504	0.072683
O	-7.230504	-2.471715	-1.149772
O	-7.715936	-1.615479	1.176946
C	-6.283604	-0.115109	-0.468474
C	-6.825361	0.998936	0.160592
C	-6.487312	2.272243	-0.289684
C	-5.607643	2.412548	-1.359491

C	-5.064009	1.290769	-1.989509
C	-5.402752	0.022867	-1.540427
C	-5.267448	3.779483	-1.874394
H	-3.146597	-3.659666	1.174029
H	-1.729529	-3.075146	3.100576
H	-4.341639	-0.100157	4.290587
H	-5.877661	-0.566491	2.455672
H	-7.511121	0.872921	0.992313
H	-6.907242	3.146217	0.197964
H	-4.378303	1.405539	-2.823480
H	-4.987390	-0.856094	-2.024041
F	-5.395103	4.739540	-0.928818
F	-6.078274	4.148604	-2.906148
F	-3.998776	3.853095	-2.345203

Pyridine **py** (py_001_dcm_fr)

N	0.000007	1.421246	-0.000002
C	1.147847	0.723139	-0.000006
C	1.201143	-0.673568	-0.000003
C	-0.000010	-1.386862	0.000000
C	-1.201150	-0.673557	0.000002
C	-1.147836	0.723155	0.000012
H	2.065266	1.308634	-0.000009
H	2.160711	-1.182956	-0.000006
H	-0.000010	-2.473970	-0.000002
H	-2.160728	-1.182927	-0.000004
H	-2.065253	1.308655	-0.000001

DMAP (dmap_001_dcm)

C	-1.958350	-1.137929	-0.009938
C	-0.570530	-1.202079	-0.010741
C	0.187236	0.000003	-0.000966
C	-0.570497	1.202089	0.009878
C	-1.958328	1.137947	0.010423
N	-2.678886	0.000021	0.000574
H	-2.530304	-2.064672	-0.019033
H	-0.091619	-2.173998	-0.022052
H	-0.091602	2.174017	0.020994
H	-2.530251	2.064704	0.020268
N	1.552507	-0.000027	-0.001973
C	2.285520	1.261309	-0.017266
H	3.355224	1.051700	-0.054684
H	2.025382	1.864351	-0.897490
H	2.082245	1.856879	0.883504

C	2.285452	-1.261330	0.018811
H	2.080960	-1.861119	-0.878790
H	3.355232	-1.051660	0.053612
H	2.026358	-1.860217	0.902259

TCAP (tcap_001_dcm)

N	-0.000231	2.928293	0.025472
C	1.139090	2.212933	0.001229
C	1.215414	0.823935	-0.041996
C	-0.000028	0.086785	-0.044146
C	-1.215547	0.823783	-0.042253
C	-1.139445	2.212787	0.001040
N	0.000092	-1.291603	-0.016196
C	-1.245780	-2.021791	-0.252390
C	-2.424390	-1.323255	0.423809
C	2.550759	0.112040	-0.097051
C	2.424587	-1.323214	0.423375
C	1.246039	-2.021347	-0.253362
H	2.064984	2.788417	0.016926
H	-2.065435	2.788125	0.016572
H	-1.433442	-2.117501	-1.335909
H	-1.119676	-3.033075	0.148551
H	-2.260771	-1.316171	1.509568
H	-3.342883	-1.888547	0.229119
H	2.910616	0.085944	-1.136279
H	3.299880	0.670168	0.477591
H	2.260925	-1.317024	1.509137
H	3.343158	-1.888240	0.228282
H	1.120191	-3.033069	0.146518
H	1.433533	-2.115870	-1.337014
C	-2.550687	0.111592	-0.097719
H	-3.300370	0.669933	0.475977
H	-2.909794	0.084599	-1.137185

Methylated compounds

Methylated pyridine **ma_py**

N	0.000000	0.861058	0.000000
C	1.173183	0.182354	0.000000
C	1.189497	-1.201404	0.000000
C	-0.021863	-1.899251	0.000000
C	-1.219273	-1.182569	0.000000
C	-1.182296	0.203831	0.000000
C	0.044115	2.342475	0.000000
H	2.074299	0.784801	0.000000

H	2.142951	-1.717619	0.000000
H	-0.030391	-2.984920	0.000000
H	-2.181522	-1.682129	0.000000
H	-2.078753	0.812124	0.000000
H	-0.971774	2.734836	0.000000
H	0.572506	2.676444	0.895300
H	0.572506	2.676444	-0.895300

Methylated DMAP (mc_dmap_001_dcm_ofr)

C	1.436900	-1.172220	-0.000231
C	0.067058	-1.210849	0.009714
C	-0.692865	0.000057	0.001376
C	0.066819	1.211015	-0.015690
C	1.436722	1.172390	-0.026508
H	2.033925	-2.077030	0.007453
H	-0.410693	-2.181079	0.024455
H	-0.411323	2.181091	-0.029997
H	2.033747	2.077101	-0.042295
N	-2.038024	-0.000078	0.005578
C	-2.780034	1.264252	0.027784
H	-3.846362	1.046769	0.077080
H	-2.508721	1.860939	0.906408
H	-2.583672	1.850580	-0.878143
C	-2.780144	-1.264416	-0.018414
H	-2.577782	-1.855545	0.882939
H	-3.846765	-1.046715	-0.059459
H	-2.514428	-1.856370	-0.902055
C	3.600093	-0.000116	0.027417
H	3.974874	-0.889817	-0.480553
H	3.932283	0.000073	1.069564
H	3.974705	0.889618	-0.480463
N	2.127012	0.000037	-0.023808

Methylated TCAP (mc_tcap_002_dcm_ofr)

C	-1.805243	-1.170217	-0.132624
C	-0.433827	-1.219653	-0.143878
C	0.304916	-0.000212	-0.000585
C	-0.437622	1.218876	0.131986
C	-1.808280	1.167264	0.100864
N	1.652222	0.001162	0.005690
C	2.438183	1.245939	0.044978
C	1.639002	2.445453	-0.452371
C	0.308217	-2.524299	-0.298724
C	1.638475	-2.444727	0.456909

C	2.440902	-1.242154	-0.027783
H	-2.404098	-2.070095	-0.221671
H	-2.410814	2.065531	0.183075
H	2.784387	1.408263	1.075382
H	3.323407	1.088430	-0.580454
H	1.461420	2.348483	-1.531179
H	2.227254	3.354622	-0.291467
H	-0.305758	-3.355784	0.063265
H	0.507565	-2.709511	-1.363966
H	2.229983	-3.352336	0.299091
H	1.449346	-2.351908	1.534115
H	2.799313	-1.400685	-1.054583
H	3.318580	-1.084732	0.608256
C	0.300746	2.525393	0.288971
H	-0.310813	3.354360	-0.082575
H	0.488713	2.714850	1.355530
C	-3.965635	-0.001787	0.034341
H	-4.345127	0.889200	-0.469533
H	-4.294156	-0.004095	1.077982
H	-4.345223	-0.890262	-0.472365
N	-2.494077	-0.001673	-0.025891

Methylated ion pair **3a**: Me_**3a** (ma_cat3a_003pp_dcm_ofr)

C	3.329141	1.341407	-0.842250
C	3.569765	2.669388	-1.100525
C	2.908279	3.282631	1.064554
C	2.653504	1.976109	1.386156
C	2.845294	0.932634	0.433636
N	2.533814	-0.320828	0.865238
S	2.640739	-1.636018	-0.045521
O	1.967941	-2.740578	0.654567
O	2.392565	-1.464439	-1.492550
C	4.471103	-2.119646	0.038431
F	4.868666	-2.277558	1.312361
F	4.678028	-3.273994	-0.619021
F	5.238919	-1.165867	-0.527447
H	3.499432	0.627407	-1.636026
H	3.932961	3.005256	-2.064588
H	2.761318	4.093737	1.768529
H	2.291285	1.732480	2.378614
P	-2.316873	-0.053681	0.013469
C	-1.268116	1.296243	-0.597681
C	-2.003248	-1.557960	-0.950963
C	-4.066532	0.388449	-0.188064

C	-0.685123	1.224889	-1.872250
C	0.063372	2.302539	-2.349735
C	0.230173	3.446507	-1.564102
C	-0.343078	3.513360	-0.289983
C	-1.089616	2.440736	0.198646
C	-2.665914	-1.737774	-2.179121
C	-2.385903	-2.861652	-2.956744
C	-1.448216	-3.803666	-2.517633
C	-0.788548	-3.621344	-1.299347
C	-1.063690	-2.502008	-0.511119
C	-4.455847	1.714774	-0.431532
C	-5.812092	2.025677	-0.557901
C	-6.778176	1.021932	-0.442485
C	-6.390460	-0.301152	-0.202553
C	-5.038682	-0.622104	-0.077173
H	-0.798906	0.336695	-2.485464
H	0.523156	2.240685	-3.332032
H	0.815609	4.281300	-1.939487
H	-0.205578	4.396657	0.327315
H	-1.525842	2.495030	1.191911
H	-3.394877	-1.011795	-2.526748
H	-2.900221	-3.000363	-3.903808
H	-1.231254	-4.677401	-3.126570
H	-0.051919	-4.342240	-0.957836
H	-0.535507	-2.376905	0.426910
H	-3.715270	2.501999	-0.529952
H	-6.109983	3.052577	-0.751147
H	-7.831823	1.268266	-0.544008
H	-7.138126	-1.084880	-0.117181
H	-4.744067	-1.652936	0.098027
C	-1.939193	-0.320194	1.768705
C	-0.595067	-0.330122	2.180439
C	-2.964738	-0.540227	2.702421
C	-0.283521	-0.575087	3.517907
H	0.212943	-0.162147	1.474963
C	-2.641371	-0.775817	4.040688
H	-4.006520	-0.522929	2.399899
C	-1.303893	-0.796361	4.448946
H	0.758263	-0.595520	3.825708
H	-3.436726	-0.941029	4.762262
H	-1.057582	-0.983610	5.490831
N	3.356398	3.635576	-0.171814
C	3.627910	5.053345	-0.469140
H	2.777680	5.657510	-0.145393

H	4.530248	5.371221	0.060514
H	3.771427	5.173063	-1.543467

Methylated ion pair **3b**: Me_**3b** (ma_cat3b_124_dcm)

C	4.413845	1.223138	-1.274686
C	4.555062	2.466318	-1.842834
C	2.406892	3.104775	-1.149278
C	2.204515	1.882832	-0.565064
C	3.211376	0.874708	-0.591672
N	2.896308	-0.292586	0.033720
S	3.881237	-1.551144	0.125961
O	3.121926	-2.707270	0.627327
O	4.815603	-1.761927	-0.999874
C	5.030332	-1.129312	1.573233
F	4.331168	-0.934365	2.705858
F	5.906227	-2.127570	1.783119
F	5.725237	-0.003132	1.311011
H	5.231595	0.521834	-1.368207
H	5.460507	2.757032	-2.363671
H	1.653042	3.883211	-1.135021
H	1.256960	1.681749	-0.084459
C	-1.351377	2.944559	0.309773
P	-1.781258	1.230263	0.743601
C	-3.581137	1.159763	1.018924
C	-4.433829	2.159611	0.526191
C	-5.818841	2.016605	0.648784
C	-6.356581	0.879954	1.260322
C	-5.507756	-0.114996	1.759184
C	-4.124562	0.023548	1.641690
C	-0.975723	0.809778	2.317943
C	-1.578752	1.101731	3.552922
C	-0.910695	0.798448	4.741409
C	0.356105	0.204724	4.702581
C	0.956244	-0.085344	3.473347
C	0.294438	0.215108	2.281575
N	-1.262037	0.329521	-0.476953
P	-1.384502	-1.196294	-0.960742
C	0.026985	-1.506775	-2.063751
C	0.647384	-0.418083	-2.693509
C	1.709969	-0.634737	-3.572265
C	2.157576	-1.935105	-3.824801
C	1.536413	-3.022257	-3.201372
C	0.469852	-2.812633	-2.324896
C	-2.903585	-1.467175	-1.932846

C	-3.186850	-2.740712	-2.454548
C	-4.342721	-2.943855	-3.209152
C	-5.219734	-1.878717	-3.449597
C	-4.934451	-0.609121	-2.939421
C	-3.776160	-0.400518	-2.184752
C	-1.355689	-2.450745	0.358304
C	-0.129796	-2.789658	0.954696
C	-0.101243	-3.681839	2.027248
C	-1.290222	-4.235779	2.513455
C	-2.512340	-3.901238	1.920279
C	-2.548346	-3.011912	0.843829
C	-1.308537	3.316903	-1.043463
C	-1.029106	4.639624	-1.394060
C	-0.781826	5.592021	-0.399274
C	-0.815965	5.220462	0.948995
C	-1.104009	3.900590	1.306305
H	-4.026732	3.045258	0.047052
H	-6.475394	2.793857	0.266609
H	-7.434092	0.770568	1.352204
H	-5.921305	-0.997333	2.240548
H	-3.471371	-0.750463	2.032516
H	-2.564173	1.556850	3.591761
H	-1.382108	1.021209	5.695023
H	0.871654	-0.035331	5.629095
H	1.935143	-0.553906	3.435008
H	0.769329	-0.024420	1.336682
H	0.314369	0.591946	-2.479352
H	2.197436	0.213156	-4.046459
H	2.995295	-2.101335	-4.497037
H	1.889441	-4.033311	-3.385404
H	0.004324	-3.663128	-1.836096
H	-2.511643	-3.571993	-2.267750
H	-4.559614	-3.931586	-3.607248
H	-6.121839	-2.040507	-4.034039
H	-5.611917	0.220452	-3.124120
H	-3.560079	0.586504	-1.791395
H	0.803069	-2.371685	0.592068
H	0.851789	-3.938876	2.481543
H	-1.264661	-4.929233	3.350297
H	-3.437965	-4.332420	2.292479
H	-3.501514	-2.758820	0.390231
H	-1.486587	2.575104	-1.816050
H	-0.999431	4.923132	-2.442930
H	-0.560508	6.620247	-0.673723

H	-0.618166	5.955773	1.724225
H	-1.126215	3.623043	2.356310
N	3.571532	3.401522	-1.788863
C	3.783255	4.744694	-2.356723
H	2.828283	5.134941	-2.712524
H	4.190702	5.407747	-1.587972
H	4.481980	4.673179	-3.191445

Methylated ion pair **3c**: Me_**3c** (ma_cat3c_007Ab_dcm_ofr)

C	-4.149867	-0.184379	-0.785095
C	-5.231551	-0.914111	-0.352508
C	-3.859124	-2.323907	0.926306
C	-2.741340	-1.635276	0.534349
C	-2.836570	-0.518298	-0.346907
N	-1.657782	0.088955	-0.653539
S	-1.510911	1.340384	-1.640677
O	-0.082050	1.519254	-1.947403
O	-2.486514	1.441826	-2.746031
C	-1.909282	2.842885	-0.557752
F	-1.043683	2.941047	0.465181
F	-1.839536	3.972101	-1.283472
F	-3.153729	2.739496	-0.049001
H	-4.325353	0.637804	-1.465448
H	-6.241226	-0.677070	-0.667249
H	-3.812998	-3.174617	1.596893
H	-1.768773	-1.948240	0.896736
N	2.499184	-0.522908	0.492472
C	2.771783	0.833115	-0.161081
C	3.700748	1.776987	0.594795
C	1.903319	-0.346882	1.888384
C	1.523104	-1.244655	-0.438778
C	3.786112	-1.325183	0.675135
C	1.077311	-2.640559	-0.015839
C	4.524674	-1.724363	-0.598925
C	0.669676	0.544827	1.981472
H	1.794160	1.291852	-0.313347
H	3.176923	0.612928	-1.150043
H	3.318767	1.979587	1.602126
H	4.699500	1.338212	0.704225
H	2.707853	0.048013	2.511540
H	1.681616	-1.353286	2.247471
H	2.016279	-1.279603	-1.411491
H	0.656404	-0.594006	-0.539331
H	3.507443	-2.213762	1.244453

H	4.430939	-0.719946	1.314378
H	1.934901	-3.303024	0.151206
H	0.510531	-2.596720	0.922203
H	3.900940	-2.376943	-1.220296
H	4.773926	-0.840870	-1.198485
H	0.937688	1.586954	1.774770
H	-0.082540	0.258130	1.241231
C	3.818262	3.106462	-0.168676
H	4.192928	2.912407	-1.183463
H	2.818448	3.547571	-0.283759
C	4.742350	4.101855	0.539061
H	5.757387	3.695864	0.641952
H	4.812635	5.042155	-0.021383
H	4.372350	4.338240	1.545436
C	0.055110	0.453849	3.386755
H	-0.236262	-0.586925	3.588429
H	0.812422	0.717637	4.138758
C	-1.164597	1.367460	3.545000
H	-1.948264	1.110241	2.821250
H	-1.593413	1.281500	4.551134
H	-0.894958	2.418694	3.382895
C	0.179953	-3.245172	-1.108282
H	0.756113	-3.338950	-2.039211
H	-0.646895	-2.555366	-1.320567
C	-0.378592	-4.613136	-0.707556
H	-0.983387	-4.541325	0.206219
H	-1.015765	-5.027146	-1.498585
H	0.429606	-5.331477	-0.516613
C	5.821021	-2.469504	-0.241622
H	6.455216	-1.821658	0.379637
H	5.577365	-3.350127	0.369505
C	6.597336	-2.907919	-1.487136
H	7.516402	-3.440184	-1.212983
H	5.994219	-3.579089	-2.112619
H	6.880402	-2.042744	-2.100928
N	-5.099757	-1.971362	0.489032
C	-6.282553	-2.706690	0.974905
H	-6.078474	-3.778467	0.936160
H	-6.499841	-2.406527	2.003752
H	-7.134806	-2.475531	0.335607

Methylated ion pair **3d**: Me_**3d** (ma_cat3d_034a_gas_dcm_ofr)

C	4.053507	0.315685	0.514387
C	5.079023	-0.428387	1.046757

C	4.027075	-2.437696	0.445289
C	2.975262	-1.753071	-0.104687
C	2.937338	-0.328368	-0.092672
N	1.835242	0.235183	-0.659118
S	1.527204	1.805712	-0.652504
O	0.119321	2.001630	-1.037741
O	2.053167	2.588731	0.485123
C	2.479523	2.481633	-2.143346
F	2.123097	1.840288	-3.269443
F	2.230541	3.792813	-2.302183
F	3.806197	2.318948	-1.966528
H	4.116632	1.392994	0.580844
H	5.936607	0.038170	1.517447
H	4.076022	-3.520675	0.452705
H	2.160353	-2.305033	-0.557899
P	-2.508211	-0.578552	0.417637
C	-1.482911	-1.068577	-1.005960
C	-0.928208	-2.499072	-0.930541
C	-1.666367	-0.981212	1.984720
C	-2.819198	1.212542	0.307264
C	-4.083779	-1.500362	0.396635
C	-3.725615	1.775650	1.413044
C	-4.984014	-1.204162	-0.813487
C	-0.271413	-0.356920	2.139551
H	-0.667217	-0.341812	-1.075672
H	-2.113105	-0.935271	-1.894347
H	-0.244469	-2.578958	-0.074951
H	-1.739354	-3.220248	-0.760383
H	-1.612241	-2.076078	2.033171
H	-2.336332	-0.657010	2.791004
H	-3.252786	1.390828	-0.684998
H	-1.835768	1.695335	0.310697
H	-4.600031	-1.261094	1.334451
H	-3.819557	-2.564588	0.436498
H	-4.700352	1.269825	1.395518
H	-3.279674	1.576896	2.396892
H	-5.226556	-0.133798	-0.842180
H	-4.447881	-1.435341	-1.743341
H	0.368660	-0.669253	1.307433
H	-0.343207	0.736868	2.084126
C	-3.935663	3.287115	1.248669
C	-4.830308	3.876346	2.343362
H	-4.378850	3.483099	0.261972
H	-2.958090	3.789128	1.257608

H	-4.968317	4.955781	2.204416
H	-5.823752	3.408225	2.337470
H	-4.392560	3.720156	3.338321
C	-6.285199	-2.016600	-0.763492
C	-7.196651	-1.732470	-1.961425
H	-6.818068	-1.786619	0.170090
H	-6.041142	-3.087974	-0.729546
H	-8.120568	-2.321351	-1.905978
H	-7.477181	-0.671584	-2.001302
H	-6.696381	-1.981696	-2.906596
C	-0.175667	-2.875186	-2.213945
C	0.449166	-4.271245	-2.135546
H	0.605543	-2.126464	-2.403826
H	-0.868230	-2.826853	-3.065857
H	0.989377	-4.517593	-3.058012
H	1.161251	-4.341066	-1.302200
H	-0.318862	-5.040793	-1.981062
C	0.380397	-0.758052	3.469771
C	1.794254	-0.188877	3.624469
H	0.415706	-1.855225	3.537099
H	-0.252071	-0.414191	4.300443
H	2.227686	-0.462720	4.594555
H	2.459516	-0.566777	2.838489
H	1.790261	0.906528	3.553881
N	5.076429	-1.785989	1.017964
C	6.163357	-2.559870	1.645936
H	7.038233	-1.919538	1.761101
H	5.835918	-2.918871	2.625727
H	6.415159	-3.407213	1.005586

Methylated ion pair **4a**: Me_**4a** (ma_cat4a_025b_gas_dcm_ofr)

C	-5.082331	-0.155622	0.295555
C	-5.938558	-1.217883	0.166592
C	-4.318958	-2.473266	-0.985613
C	-3.415914	-1.452259	-0.893027
C	-3.752320	-0.217839	-0.241382
N	-2.797993	0.720986	-0.198571
S	-3.064653	2.163612	0.546665
O	-3.301755	2.000480	2.007364
O	-4.057668	2.992680	-0.188161
C	-1.450355	2.906113	0.331822
C	-1.142826	3.551702	-0.865419
C	0.134922	4.077657	-1.068470
C	1.103331	3.953102	-0.057943

C	0.779426	3.311458	1.150865
C	-0.492644	2.788712	1.345792
O	2.373749	4.422809	-0.150143
C	2.811120	5.000077	-1.384834
H	-5.436889	0.728164	0.811541
H	-6.948269	-1.188258	0.560278
H	-4.086017	-3.410790	-1.478449
H	-2.427678	-1.571795	-1.323902
H	-1.893823	3.644821	-1.643825
H	0.358973	4.577419	-2.003674
H	1.540227	3.226932	1.920690
H	-0.740693	2.286832	2.274579
H	2.250340	5.914677	-1.614304
H	3.864126	5.245928	-1.237347
H	2.712136	4.285471	-2.210906
P	1.896388	-1.169739	0.052891
C	3.106295	0.183247	0.094427
C	0.839443	-0.930048	-1.402176
C	2.762779	-2.762229	-0.065124
C	0.902030	-1.184990	1.570188
C	0.491562	-2.015043	-2.223647
C	-0.322902	-1.803783	-3.338094
C	-0.789603	-0.518868	-3.634463
C	-0.446165	0.558767	-2.813097
C	0.366468	0.359670	-1.697532
C	4.083585	-2.825362	-0.535713
C	4.717276	-4.064425	-0.656226
C	4.040047	-5.237400	-0.309499
C	2.724640	-5.174708	0.163713
C	2.083865	-3.941521	0.289197
C	1.444007	-1.731594	2.748711
C	0.695574	-1.712612	3.925719
C	-0.588500	-1.153421	3.933630
C	-1.126158	-0.614571	2.761949
C	-0.385823	-0.630374	1.577214
C	3.546477	0.703798	1.321198
C	4.517285	1.707064	1.338908
C	5.046283	2.194332	0.140526
C	4.606879	1.677607	-1.082402
C	3.638883	0.673059	-1.111389
H	0.857003	-3.014321	-2.009236
H	-0.588220	-2.643341	-3.974839
H	-1.424706	-0.358416	-4.501652
H	-0.819666	1.555289	-3.027208

H	0.619246	1.203087	-1.062013
H	4.621486	-1.920875	-0.801787
H	5.741204	-4.109523	-1.016772
H	4.537517	-6.199072	-0.403171
H	2.197299	-6.083936	0.439128
H	1.065884	-3.899691	0.665593
H	2.436617	-2.172914	2.749113
H	1.114778	-2.135823	4.834454
H	-1.167375	-1.139575	4.853518
H	-2.114334	-0.163508	2.758281
H	-0.830025	-0.206507	0.682391
H	3.131569	0.343394	2.257165
H	4.848545	2.115168	2.289709
H	5.792634	2.984033	0.159018
H	5.011176	2.059762	-2.015579
H	3.299098	0.279629	-2.064837
N	-5.576045	-2.372176	-0.458731
C	-6.539123	-3.469735	-0.641319
H	-6.025011	-4.424950	-0.514481
H	-7.327627	-3.384241	0.108110
H	-6.977239	-3.417075	-1.642784

Methylated ion pair **5a**: Me_**5a** (ma_cat5a_032_dcm_ofr)

C	3.447556	-1.388645	-1.518152
C	4.131353	-2.570023	-1.579324
C	5.170124	-2.135012	0.484111
C	4.513108	-0.939399	0.608604
C	3.581739	-0.507442	-0.394716
N	2.820549	0.596614	-0.395029
S	2.837025	1.618204	0.895521
O	2.222176	0.997130	2.101144
O	4.170820	2.245370	1.100285
C	1.710524	2.878789	0.295999
C	2.133729	3.745673	-0.716952
C	1.249675	4.707238	-1.199812
C	-0.053742	4.822259	-0.682435
C	-0.443566	3.949447	0.341503
C	0.428848	2.974211	0.833699
C	-1.005014	5.852848	-1.238641
H	2.770093	-1.123295	-2.322581
H	4.029062	-3.256500	-2.412125
H	5.869852	-2.484225	1.235359
H	4.704943	-0.339080	1.489257
H	3.136695	3.664990	-1.125620

H	1.572386	5.379222	-1.992115
H	-1.444269	4.024251	0.758674
H	0.112040	2.298266	1.620893
H	-1.941544	5.883259	-0.672043
H	-0.559292	6.855120	-1.219525
H	-1.250291	5.629059	-2.285390
P	-2.059950	-0.850250	0.032928
C	-0.698986	-2.002083	0.384247
C	-3.546247	-1.774303	-0.446407
C	-1.623827	0.266009	-1.329840
C	-0.735424	-3.322362	-0.093676
C	0.321611	-4.190420	0.189165
C	1.407769	-3.750257	0.952329
C	1.442028	-2.436252	1.428425
C	0.396800	-1.557740	1.144513
C	-3.649660	-2.281928	-1.754030
C	-4.772065	-3.024773	-2.121270
C	-5.792476	-3.260279	-1.192848
C	-5.691571	-2.751340	0.105419
C	-4.570932	-2.007757	0.483608
C	-0.277010	0.547792	-1.602818
C	0.047564	1.447190	-2.619826
C	-0.961705	2.067864	-3.360127
C	-2.305722	1.792710	-3.082749
C	-2.642343	0.892620	-2.070955
H	-1.581109	-3.681871	-0.671070
H	0.288317	-5.211932	-0.179797
H	2.226159	-4.429834	1.175973
H	2.283962	-2.082954	2.014130
H	0.453380	-0.539613	1.514340
H	-2.864689	-2.095379	-2.481123
H	-4.850353	-3.414859	-3.132328
H	-6.667496	-3.835714	-1.483372
H	-6.486051	-2.926716	0.825498
H	-4.505500	-1.611702	1.492211
H	0.526815	0.097646	-1.030335
H	1.092162	1.668035	-2.815946
H	-0.703567	2.770691	-4.147904
H	-3.093561	2.276428	-3.653738
H	-3.688156	0.682995	-1.865291
C	-2.382535	0.111387	1.538171
C	-2.268744	-0.503514	2.797453
C	-2.767979	1.457237	1.443850
C	-2.538890	0.231454	3.952453

H	-1.962013	-1.541784	2.879545
C	-3.035184	2.184398	2.605197
H	-2.844798	1.944767	0.478118
C	-2.920326	1.574427	3.857927
H	-2.443723	-0.243732	4.924817
H	-3.322215	3.229494	2.529167
H	-3.122053	2.145586	4.760251
N	4.979884	-2.960969	-0.582413
C	5.741981	-4.213684	-0.707404
H	6.660488	-4.036681	-1.275586
H	5.992279	-4.582119	0.288783
H	5.129133	-4.956886	-1.220870

Methylated ion pair **6a**: Me_**6a** (ma_cat6a_032_dcm_ofr)

C	-3.995853	-0.677482	-1.612999
C	-5.239355	-0.143805	-1.807607
C	-5.914248	-0.798860	0.346032
C	-4.687286	-1.351360	0.606254
C	-3.641839	-1.304165	-0.374048
N	-2.382363	-1.757891	-0.259325
S	-1.865411	-2.422973	1.147496
O	-1.722697	-1.411560	2.229725
O	-2.606053	-3.659978	1.509737
C	-0.205687	-2.904614	0.638239
C	-0.055980	-3.941480	-0.287159
C	1.222894	-4.294560	-0.707185
C	2.333101	-3.611312	-0.192340
C	2.175747	-2.595837	0.751458
C	0.893007	-2.234119	1.168818
C	3.706287	-4.002953	-0.663542
H	-3.257079	-0.609175	-2.404360
H	-5.523192	0.344647	-2.733064
H	-6.717503	-0.819574	1.074057
H	-4.529822	-1.813805	1.572791
H	-0.924511	-4.455284	-0.686487
H	1.355281	-5.087391	-1.437264
H	3.038229	-2.076557	1.152765
H	0.755621	-1.435033	1.888669
P	0.969168	2.147814	0.019247
C	-0.808154	2.491516	0.149161
C	1.855025	3.650816	-0.479552
C	1.281669	0.857418	-1.219392

C	-1.353242	3.642928	-0.442612
C	-2.724504	3.888939	-0.344198
C	-3.550097	2.997432	0.347707
C	-3.005278	1.854245	0.940428
C	-1.638373	1.593358	0.842247
C	1.846529	4.048967	-1.828048
C	2.497383	5.223661	-2.206624
C	3.156333	6.001411	-1.247666
C	3.165024	5.604931	0.093078
C	2.516032	4.430796	0.482304
C	0.285310	-0.082311	-1.524647
C	0.557663	-1.108257	-2.430928
C	1.816906	-1.204894	-3.028522
C	2.811977	-0.269974	-2.722291
C	2.549526	0.763262	-1.821781
H	-0.721126	4.351126	-0.968359
H	-3.142270	4.781598	-0.801664
H	-4.616125	3.194612	0.427700
H	-3.640813	1.156593	1.476027
H	-1.238482	0.696690	1.305474
H	1.341517	3.446203	-2.577849
H	2.492041	5.528251	-3.249479
H	3.666406	6.913281	-1.546807
H	3.680045	6.204751	0.838379
H	2.532861	4.127083	1.524550
H	-0.692355	-0.044838	-1.057343
H	-0.214919	-1.838538	-2.650886
H	2.027368	-2.011489	-3.725596
H	3.793637	-0.344561	-3.181979
H	3.327076	1.486074	-1.592875
C	1.580828	1.598499	1.639052
C	0.958163	2.036035	2.819843
C	2.709921	0.766180	1.705700
C	1.457995	1.627493	4.057919
H	0.087089	2.682433	2.781223
C	3.204313	0.366698	2.947572
H	3.196718	0.419118	0.800162
C	2.577547	0.792127	4.123369
H	0.967747	1.959371	4.968899
H	4.072184	-0.285356	2.994403
H	2.959315	0.470297	5.088557

F	3.753916	-4.181134	-2.012356
F	4.124219	-5.179782	-0.113855
F	4.653158	-3.079859	-0.359163
N	-6.198198	-0.187618	-0.836364
C	-7.544116	0.344607	-1.102570
H	-8.140069	-0.401844	-1.636615
H	-8.025694	0.588375	-0.154348
H	-7.458850	1.249129	-1.707661

References

(1) Harris, R. K.; Becker, E. D.; Menezes, S. M. C. de; Goodfellow, R.; Granger, P. NMR Nomenclature. Nuclear Spin Properties and Conventions for Chemical Shifts(IUPAC Recommendations 2001). *Pure Appl. Chem.* **2001**, *73* (11), 1795–1818. https://doi.org/10.1351/pac200173111795.

(2) Burger, V.; Franta, M.; Ofial, A. R.; Gschwind, R. M.; Zipse, H. Highly Nucleophilic Pyridinamide Anions in Apolar Organic Solvents Due to Asymmetric Ion Pair Association. *J. Am. Chem. Soc.* **2025**, https://doi.org/10.1021/jacs.4c14825.

(3) Helberg, J.; Ampßler, T.; Zipse, H. Pyridinyl Amide Ion Pairs as Lewis Base Organocatalysts. *J. Org. Chem.* **2020**, *85* (8), 5390–5402. https://doi.org/10.1021/acs.joc.0c00114.

(4) Mayer, R. J.; Ofial, A. R.; Mayr, H.; Legault, C. Y. Lewis Acidity Scale of Diaryliodonium Ions toward Oxygen, Nitrogen, and Halogen Lewis Bases. *J. Am. Chem. Soc.* **2020**, *142* (11), 5221–5233. https://doi.org/10.1021/jacs.9b12998.

(5) Wu, Y. C.; Koch, W. F.; Pratt, K. W. Proposed New Electrolytic Conductivity Primary Standards for KCl Solutions. *J. Res. Natl. Inst. Stand. Technol.* **1991**, *96* (2), 191–201. https://doi.org/10.6028/jres.096.008.

(6) Brown, A. M.; Fuoss, R. M. Conductance of Tetrabutylammonium Tetraphenylboride in Nitriles. *J. Am. Chem. Soc.* **1960**, *64*, 1341–1342. https://doi.org/10.1021/j100838a510.

(7) Fuoss, R. M.; Hirsch, E. Single Ion Conductances in Non-Aqueous Solvents. *J. Am. Chem. Soc.* **1960**, *82* (5), 1013–1017. https://doi.org/10.1021/ja01490a001.

(8) Hoops, S.; Sahle, S.; Gauges, R.; Lee, C.; Pahle, J.; Simus, N.; Singhal, M.; Xu, L.; Mendes, P.; Kummer, U. COPASI—a COmplex PAthway SImulator. *Bioinformatics* **2006**, *22* (24), 3067–3074. https://doi.org/10.1093/bioinformatics/btl485.

(9) Zott, F. *fabianzott/steadystate_analysis*. Github.com. https://github.com/fabianzott/steadystate_analysis.

(10) Jerschow, A.; Müller, N. Diffusion-Separated Nuclear Magnetic Resonance Spectroscopy of Polymer Mixtures. *Macromolecules* **1998**, *31* (19), 6573–6578. https://doi.org/10.1021/ma9801772.

(11) M. Barbosa, T.; Rittner, R.; F. Tormena, C.; A. Morris, G.; Nilsson, M. Convection in Liquid-State NMR: Expect the Unexpected. *RSC Adv.* **2016**, *6* (97), 95173–95176. https://doi.org/10.1039/C6RA23427E.

(12) Stejskal, E. O.; Tanner, J. E. Spin Diffusion Measurements: Spin Echoes in the Presence of a Time-Dependent Field Gradient. *J. Chem. Phys.* **1965**, *42* (1), 288–292. https://doi.org/10.1063/1.1695690.

(13) Macchioni, A.; Ciancaleoni, G.; Zuccaccia, C.; Zuccaccia, D. Determining Accurate Molecular Sizes in Solution through NMR Diffusion Spectroscopy. *Chem. Soc. Rev.* **2008**, *37* (3), 479–489. https://doi.org/10.1039/b615067p.

(14) Zuccaccia and, D.; Alceo Macchioni; Macchioni, A. An Accurate Methodology to Identify the Level of Aggregation in Solution by PGSE NMR Measurements: The Case of Half-Sandwich Diamino Ruthenium(II) Salts. *Organometallics* **2005**, *24* (14), 3476–3486. https://doi.org/10.1021/om050145k.

(15) Chen, H.-C.; Chen, S.-H. Diffusion of Crown Ethers In Alcohols. *J. Phys. Chem.* **1984**, *88* (21), 5118–5121. https://doi.org/10.1021/j150665a063.

(16) Ben-Amotz, D.; Willis, K. G. Molecular Hard-Sphere Volume Increments. *J. Phys. Chem.* **1993**, *97* (29), 7736–7742. https://doi.org/10.1021/j100131a051.

(17) Mayr, H.; Bug, T.; Gotta, M. F.; Hering, N.; Irrgang, B.; Janker, B.; Kempf, B.; Loos, R.; Ofial, A. R.; Remennikov, G.; Schimmel, H. Reference Scales for the Characterization of Cationic Electrophiles

and Neutral Nucleophiles. *J. Am. Chem. Soc.* **2001**, *123* (39), 9500–9512. https://doi.org/10.1021/ja010890y.

(18) Brotzel, F.; Kempf, B.; Singer, T.; Zipse, H.; Mayr, H. Nucleophilicities and Carbon Basicities of Pyridines. *Chem. - Eur. J.* **2007**, *13* (1), 336–345. https://doi.org/10.1002/chem.200600941.

(19) Rycke, N. De; Berionni, G.; Couty, F.; Mayr, H.; Goumont, R.; David, O. R. P. Synthesis and Reactivity of Highly Nucleophilic Pyridines. *Org. Lett.* **2011**, *13* (3), 530–533. https://doi.org/10.1021/ol1029589.

(20) Sooväli, L.; Rodima, T.; Kaljurand, I.; Kütt, A.; Koppel, I. A.; Leito, I. Basicity of Some P1 Phosphazenes in Water and in Aqueous Surfactant Solution. *Org. Biomol. Chem.* **2006**, *4* (11), 2100–2105. https://doi.org/10.1039/B602797K.

(21) Gagliardi, L. G.; Castells, C. B.; Ràfols, C.; Rosés, M.; Bosch, E. δ Conversion Parameter between pH Scales in Acetonitrile/Water Mixtures at Various Compositions and Temperatures. *Anal. Chem.* **2007**, *79* (8), 3180–3187. https://doi.org/10.1021/ac062372h.

(22) Folda, A.; Scalcon, V.; Ghazzali, M.; Jaafar, M. H.; Khan, R. A.; Casini, A.; Citta, A.; Bindoli, A.; Rigobello, M. P.; Al-Farhan, K.; Alsalme, A.; Reedijk, J. Insights into the Strong In-Vitro Anticancer Effects for Bis(Triphenylphosphane)Iminium Compounds Having Perchlorate, Tetrafluoridoborate and Bis(Chlorido)Argentate Anions. *J. Inorg. Biochem.* **2015**, *153*, 346–354. https://doi.org/10.1016/j.jinorgbio.2015.08.030.

(23) Bertocco, P.; Bolli, C.; Correia Bicho, B. A.; Jenne, C.; Erken, B.; Laitinen, R. S.; Seeger, H. A.; Takaluoma, T. T. Theoretical and Synthetic Study on the Existence, Structures, and Bonding of the Halide-Bridged [B2X7]− (X = F, Cl, Br, I) Anions. *Inorg. Chem.* **2016**, *55* (7), 3599–3604. https://doi.org/10.1021/acs.inorgchem.6b00118.

(24) Denny, J. A.; Darensbourg, M. Y. *CSD Commun.* **2016**.

(25) Sinnaeve, D. The Stejskal-Tanner Equation Generalized for Any Gradient Shape-An Overview of Most Pulse Sequences Measuring Free Diffusion. *Concepts Magn. Reson.* **2012**, *40 A* (2), 39–65. https://doi.org/10.1002/cmr.a.21223.

(26) Becke, A. D. A New Mixing of Hartree-Fock and Local Density-Functional Theories. *J. Chem. Phys.* **1993**, *98* (2), 1372–1377. https://doi.org/10.1063/1.464304.

(27) Grimme, S. Semiempirical Hybrid Density Functional with Perturbative Second-Order Correlation. *J. Chem. Phys.* **2006**, *124* (3), 034108. https://doi.org/034108.

(28) Lee, C.; Yang, W.; Parr, R. G. Development of the Colic-Salvetti Correlation-Energy Formula into a Functional of the Electron Density. *Phys. Rev.* **1988**, *37* (2), 785–789.

(29) Spitznagel, G. W.; Clark, T.; Chandrasekhar, J.; Schleyer, P. V. R. Stabilization of Methyl Anions by First-row Substituents. The Superiority of Diffuse Function-augmented Basis Sets for Anion Calculations. *J. Comput. Chem.* **1982**, *3* (3), 363–371. https://doi.org/10.1002/jcc.540030311.

(30) Marenich, A. V.; Cramer, C. J.; Truhlar, D. G. Universal Solvation Model Based on Solute Electron Density and on a Continuum Model of the Solvent Defined by the Bulk Dielectric Constant and Atomic Surface Tensions. *J. Phys. Chem. B* **2009**, *113* (18), 6378–6396. https://doi.org/10.1021/jp810292n.

(31) Marin-Luna, M.; Patschinski, P.; Zipse, H. Substituent Effects in the Silylation of Secondary Alcohols: A Mechanistic Study. *Chem. Eur. J.* **2018**, *24* (56), 15052–15058. https://doi.org/10.1002/chem.201803014.

(32) Marin-Luna, M.; Pölloth, B.; Zott, F.; Zipse, H. Size-Dependent Rate Acceleration in the Silylation of Secondary Alcohols: The Bigger the Faster. *Chem. Sci.* **2018**, *9* (31), 6509–6515. https://doi.org/10.1039/c8sc01889h.

(33) Pölloth, B.; Sibi, M. P.; Zipse, H. The Size-Accelerated Kinetic Resolution of Secondary Alcohols. *Angew. Chem. - Int. Ed.* **2021**, *60* (2), 774–778. https://doi.org/10.1002/anie.202011687.

(34) Mayr, S.; Marin-Luna, M.; Zipse, H. Size-Driven Inversion of Selectivity in Esterification Reactions: Secondary Beat Primary Alcohols. *J. Org. Chem.* **2021**, *86* (4), 3456–3489. https://doi.org/10.1021/acs.joc.0c02848.

(35) Grimme, S. Supramolecular Binding Thermodynamics by Dispersion-Corrected Density Functional Theory. *Chem. - Eur. J.* **2012**, *18* (32), 9955–9964. https://doi.org/10.1002/chem.201200497.

(36) Luchini, G.; Alegre-Requena, J. V.; Funes-Ardoiz, I.; Paton, R. S. GoodVibes: Automated Thermochemistry for Heterogeneous Computational Chemistry Data [Version 1; Peer Review: 2 Approved with Reservations]. *F1000Research* **2020**, *9* (291), 1–14. https://doi.org/10.12688/f1000research.22758.1.

(37) Gaussian 16; Revision A.03; Frisch, M. J.; Trucks, G. W.; Schlegel, H. B.; Scuseria, G. E.; Robb, M. A.; Cheeseman, J. R.; Scalmani, G.; Barone, V.; Petersson, G. A.; Nakatsuji, H.; Li, X.; Caricato, M.; Marenich, A. V.; Bloino, J.; Janesko, B. G.; Gomperts, R.; Mennucci, B.; Hratchian, H. P.; Ortiz, J. V.; Izmaylov, A. F.; Sonnenberg, J. L.; Williams-Young, D.; Ding, F.; Lipparini, F.; Egidi, F.; Goings, J.; Peng, B.; Petrone, A.; Henderson, T.; Ranasinghe, D.; Zakrzewski, V. G.; Gao, J.; Rega, N.; Zheng, G.; Liang, W.; Hada, M.; Ehara, M.; Toyota, K.; Fukuda, R.; Hasegawa, J.; Ishida, M.; Nakajima, T.; Honda, Y.; Kitao, O.; Nakai, H.; Vreven, T.; Throssell, K.; Montgomery, J. A. Jr.; Peralta, J. E.; Ogliaro, F.; Bearpark, M. J.; Heyd, J. J.; Brothers, E. N.; Kudin, K. N.; Staroverov, V. N.; Keith, T. A.; Kobayashi, R.; Normand, J.; Raghavachari, K.; Rendell, A. P.; Burant, J. C.; Iyengar, S. S.; Tomasi, J.; Cossi, M.; Millam, J. M.; Klene, M.; Adamo, C.; Cammi, R.; Ochterski, J. W.; Martin, R. L.; Morokuma, K.; Farkas, O.; Foresman, J. B.; Fox, D. J. Gaussian 16, Revision A.03, **2016**, p Gaussian, Inc, Wallingford CT.

(38) Gaussian 16; Revision B.01; Frisch, M. J.; Trucks, G. W.; Schlegel, H. B.; Scuseria, G. E.; Robb, M. A.; Cheeseman, J. R.; Scalmani, G.; Barone, V.; Petersson, G. A.; Nakatsuji, H.; Li, X.; Caricato, M.; Marenich, A. V.; Bloino, J.; Janesko, B. G.; Gomperts, R.; Mennucci, B.; Hratchian, H. P.; Ortiz, J. V.; Izmaylov, A. F.; Sonnenberg, J. L.; Williams-Young, D.; Ding, F.; Lipparini, F.; Egidi, F.; Goings, J.; Peng, B.; Petrone, A.; Henderson, T.; Ranasinghe, D.; Zakrzewski, V. G.; Gao, J.; Rega, N.; Zheng, G.; Liang, W.; Hada, M.; Ehara, M.; Toyota, K.; Fukuda, R.; Hasegawa, J.; Ishida, M.; Nakajima, T.; Honda, Y.; Kitao, O.; Nakai, H.; Vreven, T.; Throssell, K.; Montgomery, J. A. Jr.; Peralta, J. E.; Ogliaro, F.; Bearpark, M. J.; Heyd, J. J.; Brothers, E. N.; Kudin, K. N.; Staroverov, V. N.; Keith, T. A.; Kobayashi, R.; Normand, J.; Raghavachari, K.; Rendell, A. P.; Burant, J. C.; Iyengar, S. S.; Tomasi, J.; Cossi, M.; Millam, J. M.; Klene, M.; Adamo, C.; Cammi, R.; Ochterski, J. W.; Martin, R. L.; Morokuma, K.; Farkas, O.; Foresman, J. B.; Fox, D. J. Gaussian 16, Revision B.01, **2016**, p Gaussian, Inc, Wallingford CT.

(39) Maestro, rev. 12.2.012; Schrödinger, New York, **2019**.

(40) Martin Saunders. Stochastic Search for Isomers on a Quantum Mechanical Surface. *J. Comput. Chem.* **2004**, *25* (5), 621–626.

(41) Šakić, D.; Hanževački, M.; Smith, D. M.; Vrček, V. A Computational Study of the Chlorination and Hydroxylation of Amines by Hypochlorous Acid. *Org. Biomol. Chem.* **2015**, *13* (48), 11740–11752. https://doi.org/10.1039/c5ob01823d.

(42) Grimme, S.; Hansen, A.; Ehlert, S.; Mewes, J. M. R2SCAN-3c: A "Swiss Army Knife" Composite Electronic-Structure Method. *J. Chem. Phys.* **2021**, *154* (6). https://doi.org/10.1063/5.0040021.

(43) Neese, F. Software Update: The ORCA Program System—Version 5.0. *WIREs Comput. Mol. Sci.* **2022**, *12* (5), e1606. https://doi.org/10.1002/wcms.1606.

(44) Gaussian 16; Revision C.01; Frisch, M. J.; Trucks, G. W.; Schlegel, H. B.; Scuseria, G. E.; Robb, M. A.; Cheeseman, J. R.; Scalmani, G.; Barone, V.; Petersson, G. A.; Nakatsuji, H.; Li, X.; Caricato, M.; Marenich, A. V.; Bloino, J.; Janesko, B. G.; Gomperts, R.; Mennucci, B.; Hratchian, H. P.; Ortiz, J. V.; Izmaylov, A. F.; Sonnenberg, J. L.; Williams-Young, D.; Ding, F.; Lipparini, F.; Egidi, F.; Goings, J.; Peng, B.; Petrone, A.; Henderson, T.; Ranasinghe, D.; Zakrzewski, V. G.; Gao, J.; Rega, N.; Zheng, G.; Liang, W.; Hada, M.; Ehara, M.; Toyota, K.; Fukuda, R.; Hasegawa, J.; Ishida, M.; Nakajima, T.; Honda,

Y.; Kitao, O.; Nakai, H.; Vreven, T.; Throssell, K.; Montgomery, J. A. Jr.; Peralta, J. E.; Ogliaro, F.; Bearpark, M. J.; Heyd, J. J.; Brothers, E. N.; Kudin, K. N.; Staroverov, V. N.; Keith, T. A.; Kobayashi, R.; Normand, J.; Raghavachari, K.; Rendell, A. P.; Burant, J. C.; Iyengar, S. S.; Tomasi, J.; Cossi, M.; Millam, J. M.; Klene, M.; Adamo, C.; Cammi, R.; Ochterski, J. W.; Martin, R. L.; Morokuma, K.; Farkas, O.; Foresman, J. B.; Fox, D. J. Gaussian 16, Revision C.01, **2016**, p Gaussian, Inc, Wallingford CT.
